# Asymmetric construction of allylicstereogenic carbon center featuring atrifluoromethyl group via enantioselective reductive fluoroalkylation

**DOI:** 10.1038/s41467-022-34841-1

**Published:** 2022-11-17

**Authors:** Ruo-Xing Jin, Bing-Bing Wu, Kang-Jie Bian, Jian-Liang Yu, Jing-Cheng Dai, Ya-Wen Zuo, Yi-Fan Zhang, Xi-Sheng Wang

**Affiliations:** 1 Department of Chemistry, University of Science and Technology of China, 230026 Hefei, China; 2 State Key Laboratory of Elemento-Organic Chemistry, Nankai University, 300071 Tianjin, China

**Keywords:** Synthetic chemistry methodology, Stereochemistry

## Abstract

Emerging as a powerful tool for lead optimization in pharmaceutical research and development, to develop the facile, general protocols that allows the incorporation of fluorine-containing motif in drug candidates has accumulated enormous research interest in recent years. Among these important motifs, the incorporation of strategic motif CF_3_ on aliphatic chain especially with the concomitant construction of trifluoromethylated alkanes bearing a CF_3_-substituted stereogenic carbon, is of paramount importance. Herein, we disclose an asymmetric nickel-catalyzed reductive trifluoroalkylation of alkenyl halides for enantioselective syntheses of diverse *α*-trifluoromethylated allylic alkanes, offering a general protocol to access the trifluoromethyl analogue to chiral *α*-methylated allylic alkanes, one of the most prevalent key components among natural products and pharmaceuticals. Utilities of the method including the application of the asymmetric trifluoroalkylation on multiple biologically active complex molecules, derivatization of transformable alkenyl functionality were demonstrated, providing a facile method in the diversity-oriented syntheses of CF_3_-containing chiral drugs and bioactive-molecules.

## Abstract (web summary)

The efficient construction of trifluoromethylated alkanes bearing a CF_3_ chiral center is of great importance in organic synthesis. Here, the authors disclose the enantioselective syntheses of α-trifluoromethylated allylic alkanes via reductive trifluoroalkylation.

## Introduction

Fluorine-containing drugs have been emerging as one of the most important therapeutic candidates on global pharmaceutical market over the past few years, as the selective incorporation of fluorine atom(s) or fluorine-containing motifs could significantly affect the therapeutic profiles including enhancement of the lipophilic, bioavailability and metabolic stability of parent molecules^1–3^. Therefore, fluorine incorporation or fluorine-scan has been widely used as a routine and powerful strategy for lead optimization in drug discovery, diverse synthesis of the fluorinated derivatives of biologically important compounds^4–12^. Among these fluorinated functionalities, trifluoromethyl group (CF_3_), widely known as a bulky and highly electronegative group that has remarkable effects on the adjacent functional groups, has been triggering the interest of synthetic and medicinal chemists for decades, while most efforts were focused on aromatic trifluoromethylation^13–20^. Given that the enantiomers of the chiral drug could demonstrate distinctive differentiation in activities like pharmacokinetics, efficacy and toxicity, drug chirality has long been recognized as a major theme in drug research and development^21–24^. Under these contexts, the incorporation of strategic motif CF_3_ on aliphatic chain especially with the concomitant construction of trifluoromethylated alkanes bearing a CF_3_-substituted stereogenic carbon, is of high importance while it still remains largely underdeveloped^25–34^.

**Fig. 1:**
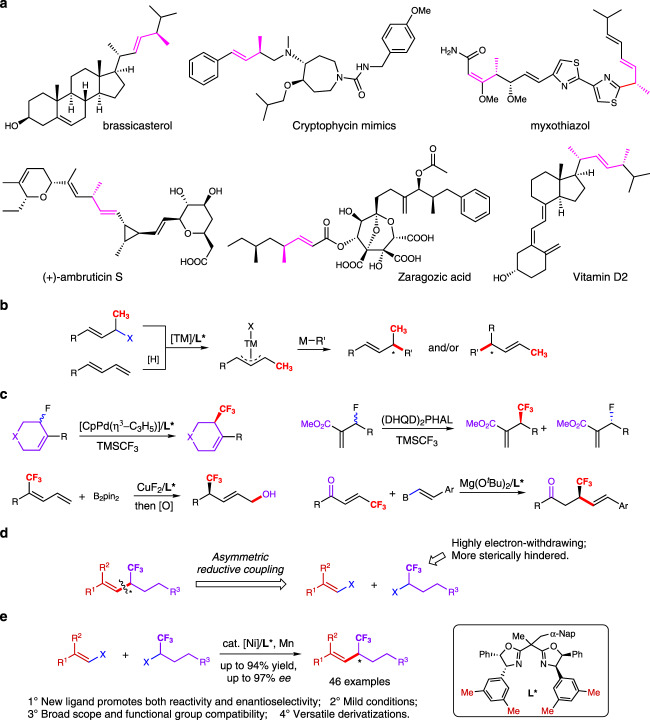
Asymmetric synthesis of enantioenriched olefins featuring allylic (trifluoro)methyl stereocenters. **a** Stereochemically complex biologically important molecules bearing allylic methyl stereocenters. **b** Strategies for accessing enantioenriched olefins bearing allylic methyl stereocenters. **c** Known strategies for synthesis of chiral olefins featuring allylic trifluoromethyl stereogenic carbon centers. **d** Our strategy to enantioenriched a-trifluoromethyl olefins. **e** This work: Nickel-catalyzed asymmetric reductive coupling to construct allylic trifluoromethyl stereocenters.

Allylic stereogenic carbon center bearing a methyl group come forward as a pivotal structural element, which has seen its prevalence in diverse biologically important natural products and drug-like molecules (Fig. 1a)^35–39^. Pioneering works for asymmetric construction of such structural units were mostly demonstrated via conventional asymmetric alkylation of electrophilic *π*-allyl complexes, formed through oxidation addition of transition metal to allylic (pseudo)halides or hydrometallation of 1,3-dienes despite the simultaneous control of both regio- and enantioselectivity could be a problematic issue in these AAA-type (asymmetric allylic alkylation) reactions (R = 1^o^ alkyl, Fig. 1b)^40–54^. Regarding to trifluoromethylation, such strategy was utilized to construct the internal alkenes featuring allylic trifluoromethyl-substituted stereocenters via asymmetric allylic trifluoromethylation in 2019 by Trost, while the protocol was only applicable to cyclic allylic fluorides^55^. It is worth mentioning that Shibata reported the asymmetric introduction of nucleophilic trifluoromethyl group onto allylic site via (DHQD)_2_PHAL-catalzyed kinetic resolution of electron-deficient allyl fluorides^56^. Apart from the direct nucleophilic trifluoromethylation, with the usage of CF_3_-preinstalled 1,3-dienes or enones, copper-catalyzed enantioselective protoboration and hydroxytetraphenylene-catalyzed asymmetric conjugate addition of vinyl boronic acids were demonstrated by Zhang^57^ and Chang^58^ respectively (Fig. 1c). Undoubtedly, the limited substrate sets in these works have rendered the lack of generality in construction of allylic trifluoromethylated stereocenters, which would inevitably hamper their application in diversity-oriented synthesis (DOS) of chiral trifluoromethylated molecules.

We are intrigued by the possibility that conducive effect could be brought to these drug-like derivatives in their physiological and biological properties by replacing methyl group (CH_3_-) of allylic methylated alkanes with its bio-isostere, trifluoromethyl group (CF_3_-). Empowered by retrosynthetic analysis, we reckoned that the enantioselective construction of the allylic trifluoromethylated alkanes could be accessed through enantioconvergent coupling of readily prepared trifluoroalkylated halides and diverse alkenyl halides. Indeed, the enantioconvergent differentiation would be more effective between the sterically hindered trifluoromethyl group and the alkyl substituents, thus enabling an asymmetric reductive radical cross-coupling (Fig. 1d)^59,60^.

Herein, we report an efficient strategy for the general and efficient asymmetric construction of allylic stereocenters featuring a trifluoromethyl group via a nickel-catalyzed enantioselective reductive trifluoroalkylation (Fig. 1e). The transformation demonstrated broad functional group compatibility, mild conditions and excellent enantioselective control; the corresponding product, *α*-trifluoromethyl olefins, could be further utilized as a versatile CF_3_-containing chiral building blocks, allowing facile syntheses of various chiral drug-like molecules featuring trifluoromethylated stereogenic carbon center through simple derivatizations of C = C bonds. As enantiopure *α*-methylated olefins represent a key structural unit in tons of bioactive molecules, this method could provide a highly efficient and selective synthetic route to trifluoromethylated chiral analogs in fluorine-containing pharmaceutical design and development.

## Results

### Optimization of reaction conditions

As a step- and atom-economic alternative strategy to traditional coupling reactions, however, only few enantioselective reductive coupling reactions have been developed by alkenylation at the benzyl and/or *α*-position of heteroatoms, possibly due to the difficulty to match the reaction rate of elementary steps two electrophiles involved^61–64^. Meanwhile, it would be ideal if the chiral allylic trifluoromethylated alkanes could be generated from the direct cross-coupling of the commercially available and abundant starting material such as alkenyl halide and trifluoromethyl halide with exceptional functional group compatibility. Directed by this hypothesis-driven design, we sought to commence our initial studies with methyl (*E*)−4-(2-iodovinyl) benzoate (**1a**) as the pilot substrate, racemic trifluoromethylated alkyl bromide (**2a**) as the trifluoroalkylating agent and Mn as the reductant in the presence of a catalytic amount of NiBr_2_•DME (10 mol%) in THF (Table 1). Satisfyingly, the reaction proceeded smoothly in 52% yield with 64% *ee* values with **L1** used as the ligand. Interestingly, both frameworks such as pyridyl-substituted oxazoliine (**L2**) with stronger coordinating moiety or tridentate ligand (**L3**) with extended aromatic plane indicated inefficiency on enantioselective control or sluggish transformation (entries 2–3). Excitingly, spacing oxazoline coordinating units with gem-disubstitutents could greatly improve the enantioselectivity substantially (**L4**-**L6**) (entries 4–6). Furthermore, modification of gem-disubstituted showed that increasing control of enantiomeric excess (**L7-L8**) (entries 7–8) and 51% of yield and 93% of *ee* were obtained with the usage of **L8** (entry 9). TBAI was also found to be helpful in reaction turnover, possibly through in situ formation of more reactive trifluoromethylated alkyl iodide (entry 10). Of note is that co-solvent could help with reaction efficiency greatly (entries11-12), improving the yield to 79% when using 9:1 of THF/N,N-Dimethylpropionamide solvent mixture. Finally, benefited from the hypothesis-driven design of the ligand, we reasoned that increasing steric effect on oxazoline ring could improve the differentiation of aliphatic and alkenyl coupling partners in the enantioconvergent step. Indeed, switching from phenyl to 3,5-methyl-phenyl (**L9**) of oxazoline substituents, the trifluoromethylated allylic alkane could be afforded in 90% yields with 95% enantiomeric excess values (entry 13). Control experiment showed that vinyl bromide could be converted to the expected product, albeit with relatively lower yield and enantioselectivity (entry 14).

**Table 1:** Nickel-catalyzed asymmetric reductive trifluoroalkylation of vinyl iodides: optimization of conditions^a^


Entry	Ligand	Solvent	Yield (%)^b^	*ee* (%)^c^
1	**L1**	THF	52	64
2	**L2**	THF	17	–
3	**L3**	THF	0	–
4	**L4**	THF	24	77
5	**L5**	THF	27	85
6	**L6**	THF	20	88
7	**L7**	THF	27	90
8	**L8**	THF	37	93
9^d^	**L8**	THF	51	93
10^d,e^	**L8**	THF	59	93
11^d,e^	**L8**	THF/DMAc (9/1)	78	91
12^d,e^	**L8**	THF/*N*,*N*-Dimethylpropionamide (9/1)	79	92
13^d,e^	**L9**	THF/*N*,*N*-Dimethylpropionamide (9/1)	90	95
14^d,e,f^	**L9**	THF/*N*,*N*-Dimethylpropionamide (9/1)	81	94

*THF* tetrahydrofuran, *DMAc* dimethylacetamide, *ee* enantiomer excess, *TBAI* tetrabutylammonium iodide.

^a^Reaction conditions (unless otherwise specified): **1a** (0.10 mmol, 1.0 equiv), **2a** (0.10 mmol, 1.0 equiv), NiBr_2_•DME (10 mol%), Ligand (13 mol%), Mn (0.20 mmol, 2.0 equiv), TBAI (0.05 mmol, 0.5 equiv), THF (0.5 mL), −4 °C, 20 h.

^b^Isolated yields.

^c^The *ee* values were determined by HPLC on a chiral stationary phase.

^d^2.0 equiv of **2a** was used.

^e^1.0 equiv of TBAI was used.

^f^Vinyl bromide was used instead.

### Asymmetric reductive cross-coupling trifluoroalkylation of alkenyl halides

With the optimized reaction condition in hands, we next started to investigate the reaction scope of vinyl iodides and trifluoroalkyl bromides. Firstly, a series of vinyl iodides were well compatible with this catalytic system. Both withdrawing groups, such as ester (**3**, **4**), ketone (**5**), cyanate (**6**), fluoro (**7**), chloride (**8**, **9**), and trifluoromethyl (**10**), and electron-donating groups such as methoxy (**11**, **12**) were well tolerated under the standard conditions. Besides, fused ring derivatives such as naphthyl vinyl iodide (**14**), phenyl vinyl iodide was also smoothly trifluoroalkylated to afford the desired chiral olefin (**13**) with 92% *ee* in 65% yield. Meanwhile, methyl group on the *ortho*- (**15**), *meta*- (**16**), and *para*- (**17**) site on the phenyl rings of vinyl iodide furnished excellent enantioselectivities (93–95% *ee*) and good yields, indicating less dependence on steric environment of alkenyl halides. The *para*- *tert*-butyl substituted substrate (**18**) also provides 68% yield with 92% *ee*. Noteworthily, alkyl-substituted vinyl iodides (**19**–**21**) and other conjugated system such as enoate (**22**), acrylamide (**23**) which are seldom reported in the asymmetric reductive cross-coupling also behave well in this asymmetric catalytic system, furnishing the coupling product with up to 97% *ee*. Tri-substituted vinyl iodides (**24**–**25**) also functioned well in this reaction, providing the coupling product with 93% *ee* in 45% yield and 81% *ee* in 32% yield respectively. However, (*Z*)-type or tetra-substituted vinyl iodides behave sluggishly in the asymmetric trifluoromethylation reaction, only providing trace amount of product, which might be due to the steric hindrance effect. (Supplementary Fig. 1)

**Fig. 2:**
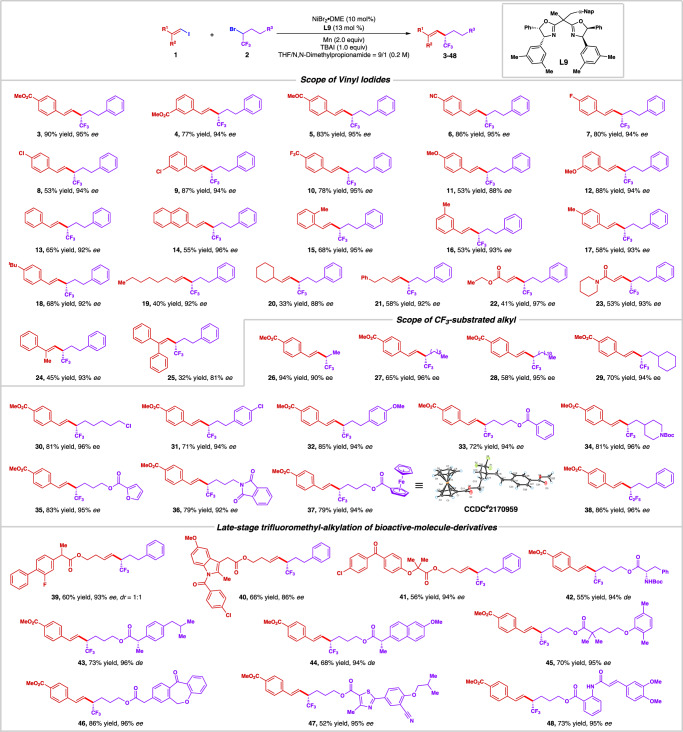
Nickel-catalyzed asymmetric reductive trifluoroalkylation of vinyl iodides. Reaction conditions: **1** (0.10 mmol, 1.0 equiv), **2** (0.20 mmol, 2.0 equiv), NiBr_2_•DME (10 mol%), **L9** (13 mol%), Mn (0.20 mmol, 2.0 equiv), TBAI (0.10 mmol, 1.0 equiv), THF (0.45 mL), *N*,*N*-Dimethylpropionamide (0.05 mL), −4 °C, 20 h; all reported yields are isolated yields; the *ee* values were determined by HPLC on a chiral stationary phase.

Next, we moved on to explore the scope of trifluoroalkylating reagents. To our delight, almost all readily available trifluoroalkyl bromides shown in Fig. 2 could be alkenylated smoothly under the standard conditions, affording the desired chiral trifluoroalkylated olefins in excellent enantioselectivities. Simple aliphatic alkyl trifluoroalkyl bromides (**26**–**29**) could furnish corresponding products in 58–94% of yields with excellent *ee* values (90–96%). Reactive handle such as chloro-tethered alkyl trifluoroalkylated bromides (**30**) also behave well, providing further derivatization potential through simple substitution reactions. Moreover, both electron-withdrawing group such as chloro and electron-donating group such as methoxy on the aryl rings (**31** and **32**) could provide the enantioselective coupling product in good yields with 94% *ee*’s. Interestingly, simple arene (**33**) as well as heterocycles such as N-Boc substituted piperidine (**34**), furan (**35**), N-Phalimide (**36**), ferrocene (**37**) were all well compatible with this asymmetric transformation, affording good yields with high *ee* values of expected products. Apart from the CF_3_ group, other fluorine-containing functionality such as C_2_F_5_ (**38**) also served as a suitable choice for this asymmetric catalytic system. Encouraged by the tolerance of the broad functionalities to our nickel-catalyzed system, we next sought out to test the late-stage asymmetric trifluoroalkylation of biologically active molecule derivatives. Excitingly, moderate to good yields and excellent enantioselectivities were afforded in all cases, including flurbiprofen (**39**), indometacin (**40**), fenofibrate (**41**), L-PHE (**42**), (S)-ibuprofen (**43**), naproxen (**44**), gemfibrozil (**45**), isoxepac (**46**), febuxostat (**47**), and (*E*) -tranilast (**48**), which demonstrates applicational potential of incorporating CF_3_-substituted stereogenic center in commercially available drugs and natural products.

### Synthetic applications

**Fig. 3:**
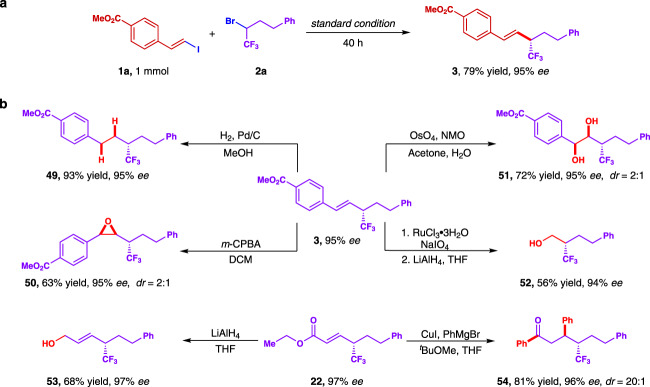
Synthetic utility. **a** 1 mmol scale reaction. **b** Synthesis of diverse trifluoromethylated chiral analogs.

To evaluate the practicability of the strategy, we carried out a 1 mmol scale reaction under the standard condition. By prolonging the reaction time to 40 h, the coupling product **3** was obtained without apparent loss of yield or enantioselectivity (Fig. 3a). Relatedly, we then carried out multiple derivatization probes of the chiral *α*-CF_3_-substituted olefins (Fig. 3b). First, the coupling product **3** could be easily transformed into a chiral asymmetric trifluoromethyl alkane (**49**) in almost quantitative yield with 95% *ee*. Epoxidation of the allylictrifluoromethyl was next proceeded, affording corresponding epoxides (**50**) in total yield of 63% while the diastereoisomers could be separated and have 95% *ee* respectively. Furthermore, we also tested dehydroxylating protocol on the standard product and we were able to access both isomers (**51**) in 72% yield with 95% *ee*. The oxidation with RuCl_3_ and sodium periodate provided the *α*-CF_3_ acid which was transformed to *β*-CF_3_ alcohol (**52**) by the reduction of LiAlH_4_ in total 56% yield with 94% *ee*. As for the enoate coupling product **22**, it could be reduced to allylic alcohol (**53**) by LiAlH_4_ in 68% yield without erosion in enantioselectivity. While the acrylate derivative also served as a typical Michael acceptor in conjugate addition, the asymmetric addition of organometallic reagents to **22** was performed and the phenyl substituted ketone (**54**) was determined to be the major product with 81% yield and 96% *ee*. Overall, these preliminary synthetic applications have indicated almost exclusive optic retention of CF_3_-substituted stereogenic carbon and we believe that since alkene motif has represented one of most transformable functionalities, further elevation of molecular complexity would be useful via alkene difunctionalization in accessing diverse chiral trifluoroalkylated molecules.

## Discussion

To gain more insights into the mechanism of this reaction, series of control experiments were carried out (Supplementary Figs. 2–6). The coupling product **3** was subjected to the standard condition, and no isomerized (*Z*)-type alkene was detected according to the crude ^19^F NMR spectrum. It is noteworthy that the transformation of (*E*)-type alkene to (*Z*)-type alkene requires high energy for its enormous reaction barrier^65^. Monitoring experiments showed that the trifluoromethyl bromide substrate would be converted to trifluoromethyl iodide partly during the reaction process. Lastly, both of the trifluoromethyl species remained racemic while the coupling product **32** was obtained with 94% *ee*, which indicates that a kinetic resolution process is less likely.

In summary, we have developed a general and efficient nickel-catalyzed reductive trifluoroalkylation of vinyl iodides for diverse synthesis of allylic alkanes featuring *α*-trifluoromethylated stereogenic carbon. This protocol exhibits high catalytic reactivity and enantioselectivity, mild conditions and excellent functional group compatibility, enabling efficient late-stage asymmetric trifluoroalkylation of commercially available drugs and natural products. The easily transformable alkenyl motif equally offers further elaboration of these sets of chiral trifluoroalkylated molecules. This method has thus provided a powerful synthetic route to CF_3_-containing analogs to prevalent chiral *α*-methylated allylic alkanes, empowering the design and diversity-oriented synthesis of fluorine-containing chiral pharmaceuticals. The utilization of enantioconvergent transformation in accessing fluorine-containing molecules and its application in modification of bioactive complex molecules are currently ongoing in our laboratory and will be disclosed in due course.

## Methods

### General procedure for nickel-catalyzed asymmetric reductive trifluoroalkylation of vinyl iodides

NiBr_2_•DME (0.01 mmol), **L9** (0.013 mmol), Mn powder (0.20 mmol), TBAI (0.10 mmol), and vinyl iodide **1** (0.10 mmol) were firstly combined in a 10 mL oven-dried sealing tube. The vessel was evacuated and backfilled with nitrogen (repeated for 3 times). Alkyl bromide **2** (0.20 mmol), THF (0.45 mL) and N,N-Dimethylpropionamide (0.05 mL) were added via syringe. The tube was sealed with a Teflon lined cap and stirred at −4 °C for 20 h. The reaction mixture was then diluted with ethyl acetate and filtered through a pad of celite. The filtrate was added brine and extracted with ethyl acetate (three times), the combined organic layer was dried over Na_2_SO_4_, filtrated and concentrated under vacuum. The residue was then purified by flash column chromatography to give corresponding products.

## Supplementary information

Supplementary Information

## Data Availability

All data needed to support the conclusions of this manuscript are included in the main text or supplementary information. X-ray crystallographic data for **35** (CCDC 2170959) has been deposited at the Cambridge Crystallographic Data Center. Copies of the data can be obtained free of charge via www.ccdc.cam.ac.uk/data_request/cif.
