# Supplementary material for: Asymmetric construction of allylicstereogenic carbon center featuring atrifluoromethyl group via enantioselective reductive fluoroalkylation
Source: Nat Commun. 2022 Nov 17;13:7035. doi: 10.1038/s41467-022-34841-1 (PMC9672039; doi:10.1038/s41467-022-34841-1)
Supplement: Supplementary file 1 — Supplementary Information [file 41467_2022_34841_MOESM1_ESM.pdf]

Supplementary Information

For

Asymmetric Construction of Allylic Stereogenic Carbon Center Featuring a Trifluoromethyl Group via Enantioselective Reductive Fluoroalkylation

Ruo-Xing Jin[1†], Bing-Bing Wu[1†], Kang-Jie Bian[1], Jian-Liang Yu[1], Jing-Cheng Dai[1], Ya-Wen Zuo[1], Yi-Fan Zhang[1], Xi-Sheng Wang[1,2*]

[1]Department of Chemistry, University of Science and Technology of China, Hefei 230026, China.

[2]State Key Laboratory of Elemento-Organic Chemistry, Nankai University, Tianjin 300071, China

Contents

I. Supplementary Notes 3

General Information 3

II. Supplementary Methods 4

Optimization of conditions 4

Supplementary Table S1. Optimization of Ligands 4

Supplementary Table S2. Optimization of Solvents 5

Supplementary Table S3. Optimization of Ni-Catalysts 6

Supplementary Table S4. Quant of **2a** 7

Supplementary Table S5. Quant of additive 8

Supplementary Table S6. Co-solvent screening and adjustment of ligand 9

General Procedures 10

Synthesis of new chiral bisoxazoline ligands 10

Synthesis of secondary alkyl bromides, iodides and alkyl vinyl iodides 13

General procedure for Nickel-catalyzed asymmetric reductive trifluoroalkylation of vinyl iodides 17

Synthetic Utility 39

Control experiments 45

X-ray Crystallographic Data 49

III. Supplementary Figures 50

NMR Spectra of New Compounds 50

HPLC Data 139

IV. Supplementary References 192

I. Supplementary Notes

General Information

NMR spectra were recorded on Bruker-500 (500 MHz for ^{1}H; 126 MHz for ^{13}C and 471 MHz for ^{19}F). All ^{1}H NMR, ^{13}C NMR and ^{19}F NMR spectra were recorded at room temperature. ^{1}H NMR spectra were referenced relative to $CDCl_3$ at δ 7.26 ppm. ^{13}C NMR spectra were referenced relative to $CDCl_3$ at δ 77.16 ppm. The ^{13}C NMR spectra were obtained with ^{1}H decoupling. Data for ^{1}H, ^{13}C, ^{19}F NMR are recorded as follows: chemical shift (δ, ppm), multiplicity (s = singlet, d = doublet, t = triplet, m = multiplet, q = quartet et al.), integration, and coupling constant (Hz). {note: some of the NMR spectra were recorded on Bruker-400 (400 MHz for ^{1}H; 101 MHz for ^{13}C and 376 MHz for ^{19}F) and Bruker-600 (600 MHz for ^{1}H; 151 MHz for ^{13}C and 565 MHz for ^{19}F) }. High resolution mass spectra were recorded on P-SIMS-Gly of Bruker Daltonics Inc. using ESI-TOF (electrospray ionization-time of flight). High performance liquid chromatography was performed on SHIMADZU DGU-20A3R, using IA, IC, AYH, ADH, OJH, ODH, ASH chiral columns eluted with a mixture of hexane/isopropyl alcohol. $NiBr_2{\cdot}DME$ and TBAI were obtained from Sigma-Aldrich Chemicals. Mn powder was purchased from Adamas. Anhydrous THF was purchased from *J&K* Chemicals. N,N-Dimethylpropionamide was obtained from HEOWNS Chemicals. Chiral bisoxazoline ligand, secondary alkyl bromides and vinyl iodides were synthesized via following method described in this supplementary information.

II. Supplementary Methods

Optimization of conditions

Supplementary Table S1. Optimization of Ligands

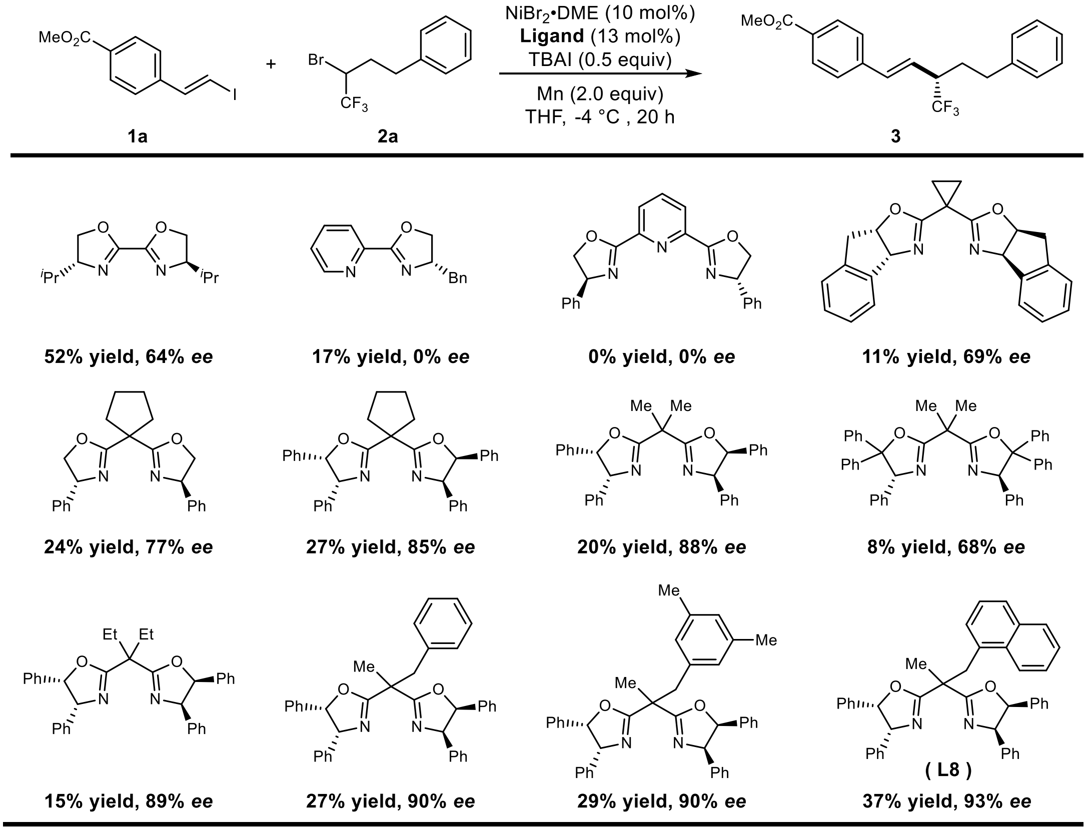

Unless otherwise noted, the reaction conditions were as follows: **1a** (0.10 mmol, 1.0 equiv), **2a** (0.10 mmol, 1.0 equiv), $NiBr_2$·DME (10 mol%), Ligand (13 mol%), Mn (0.20 mmol, 2.0 equiv), TBAI (0.05 mmol, 0.5 equiv), THF (0.5 mL), -4 °C, 20 h. Isolated yield was given. The *ee* values were determined by HPLC on a chiral stationary phase.

Supplementary Table S2. Optimization of Solvents

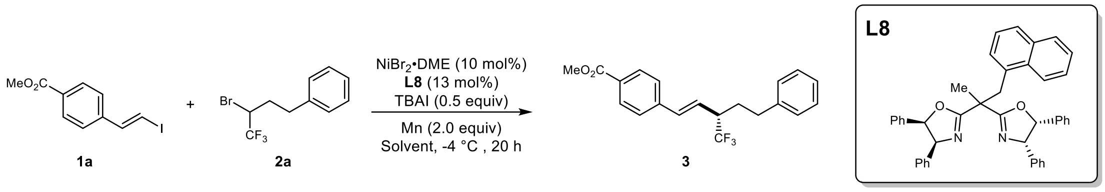

Entry	Solvent	Yield (%)[a]	ee (%)[b]
1	DMA	35	56
2	DMF	n. d.	--
3	CH_3CN	trace	--
4	NMP	n. d.	--
5	DME	8	82
6[c]	1,4-Dioxane	n. d.	--
7	THF	37	93

Unless otherwise noted, the reaction conditions were as follows: **1a** (0.10 mmol, 1.0 equiv), **2a** (0.10 mmol, 1.0 equiv), $NiBr_2$·DME (10 mol%), **L8** (13 mol%), Mn (0.20 mmol, 2.0 equiv), TBAI (0.05 mmol, 0.5 equiv), solvent (0.50 mL), -4 °C, 20 h. [a]Isolated yield. [b]The *ee* values were determined by HPLC on a chiral stationary phase. [c]The reaction was performed under room temperature.

Supplementary Table S3. Optimization of Ni-Catalysts

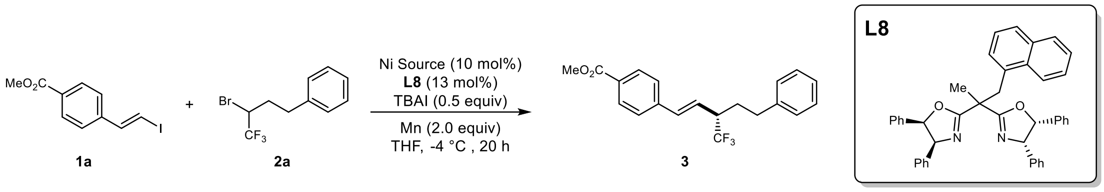

Entry	Ni Source	Yield (%)[a]	ee (%)[b]
1	$NiBr_2$·DME	37	93
2	$NiCl_2$·DME	31	90
3	$NiBr_2$	trace	--
4	$NiCl_2$	n. d.	--
5	NiI_2	21	89
6	$Ni(OTf)_2$	trace	--
7	$Ni(OAc)_2$	n. d.	--

Unless otherwise noted, the reaction conditions were as follows: **1a** (0.10 mmol, 1.0 equiv), **2a** (0.10 mmol, 1.0 equiv), Ni Source (10 mol%), **L8** (13 mol%), Mn (0.20 mmol, 2.0 equiv), TBAI (0.05 mmol, 0.5 equiv), THF (0.50 mL), -4 °C, 20 h. [a]Isolated yield. [b]The *ee* values were determined by HPLC on a chiral stationary phase.

Supplementary Table S4. Quant of **2a**

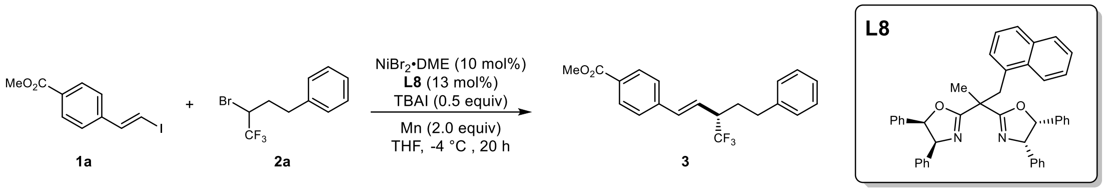

Entry	Quant of 2a (equiv)	Yield (%)[a]	ee (%)[b]
1	1.00	37	93
2	1.25	41	93
3	1.50	45	93
4	1.75	46	92
5	2.00	51	93
6	2.25	51	93
7	2.50	50	92
8	3.00	48	92

Unless otherwise noted, the reaction conditions were as follows: **1a** (0.10 mmol, 1.0 equiv), **2a** (0.x mmol, x equiv), $NiBr_2$·DME (10 mol%), **L8** (13 mol%), Mn (0.20 mmol, 2.0 equiv), TBAI (0.05 mmol, 0.5 equiv), THF (0.50 mL), -4 °C, 20 h. [a]Isolated yield. [b]The *ee* values were determined by HPLC on a chiral stationary phase.

Supplementary Table S5. Quant of additive

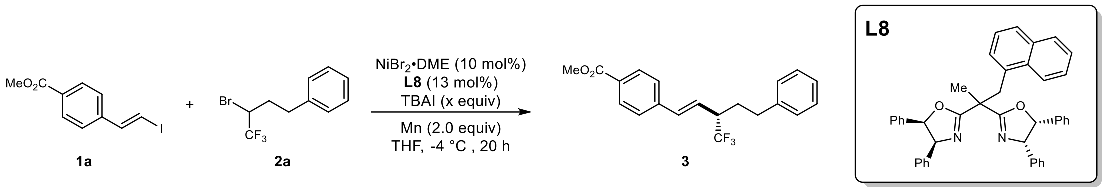

Entry	Quant of TBAI (equiv)	Yield (%)[a]	ee (%)[b]
1	0.25	33	91
2	0.50	51	93
3	1.00	59	93
4	1.50	55	92

Unless otherwise noted, the reaction conditions were as follows: **1a** (0.10 mmol, 1.0 equiv), **2a** (0.20 mmol, 2.0 equiv), $NiBr_2$·DME (10 mol%), **L8** (13 mol%), Mn (0.20 mmol, 2.0 equiv), TBAI (0.x mmol, x equiv), THF (0.50 mL), -4 °C, 20 h. [a]Isolated yield. [b]The *ee* values were determined by HPLC on a chiral stationary phase.

Supplementary Table S6. Co-solvent screening and adjustment of ligand

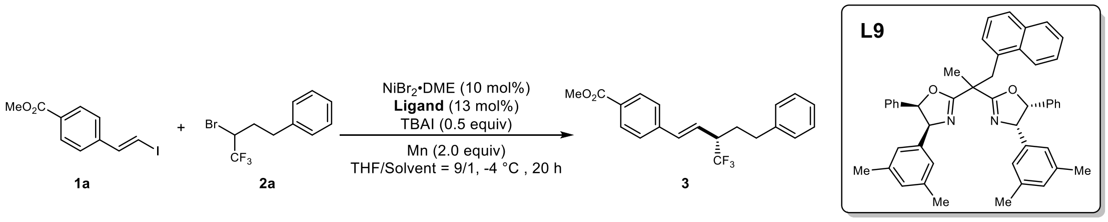

Entry	Solvent	Ligand	Yield (%)[a]	ee (%)[b]
1	DMAc	**L8**	78	91
2	DMF	**L8**	n. d.	--
3	NMP	**L8**	65	89
4	N,N-Dimethylpropionamide	**L8**	79	92
5	N,N-Diethylformamide	**L8**	n. d.	--
6	N-Acetylmorpholine	**L8**	64	92
7	N,N-Dimethylpropionamide	**L9**	90	95

Unless otherwise noted, the reaction conditions were as follows: **1a** (0.10 mmol, 1.0 equiv), **2a** (0.20 mmol, 2.0 equiv), $NiBr_2$·DME (10 mol%), Ligand (13 mol%), Mn (0.20 mmol, 2.0 equiv), TBAI (0.10 mmol, 1.0 equiv), THF (0.45 mL), co-solvent (0.05 ml), -4 °C, 20 h. [a]Isolated yield. [b]The *ee* values were determined by HPLC on a chiral stationary phase.

General procedure

Synthesis of new chiral bisoxazoline ligands

The ligand **L8** was synthesized according to the reported literature with modifications. [1]

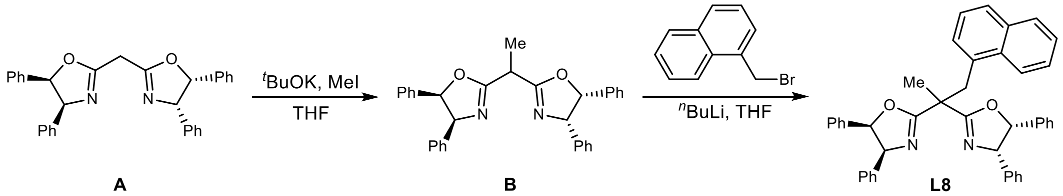

The ligand skeleton **A** (458 mg, 1.0 mmol, 1 equiv.) and ^{t}BuOK (112mg, 1.0 mmol, 1 equiv.) were firstly combined in a 25 mL oven-dried sealing tube. The vessel was evacuated and backfilled with N_2 (repeated for 3 times). THF (4 mL) was added to the reaction tube and the mixture was stirred at 40 ˚C for 1 hour. Then a solution of MeI (62 μL, 1.0 mmol, 1 equiv.) in THF (5 mL) was added dropwise (over 20 min). The resulting mixture was heated to 60 ˚C and stirred for 4 hours (TLC monitored). The mixture was quenched with sat. NH_4Cl solution and extracted with ethyl acetate for three times. The organic layer was conbined and washed with brine, dried over Na_2SO_4, filtered and concentrated in vacuo. The residue was purified by chromatography on silica gel (PE/EA = 3/2) to give the desired product **B**.

The mono methylated ligand **B** (370 mg, 0.8 mmol, 1 equiv.) was firstly added to a 25 mL oven-dried sealing tube. The vessel was evacuated and backfilled with N_2 (repeated for 3 times). THF (8 mL) was added to the reaction tube and the vessel was cooled to -78 ˚C. n-butyllithium (0.47 mL, 1.12 mmol, 2.4 M in hexane, 1.4 equiv.) was added to the reaction tube dropwise. After that, the reaction was stirred at room temperature for 30 min. Then the reaction tube was transferred to -78 ˚C and a solution of 1-(Bromomethyl)naphthalene (354 mg, 1.6 mmol, 2 equiv.) in THF (2 mL) was added dropwise. The resulting mixture was warmed to room temperature and stirred for 8 hours. The mixture was quenched with sat. NH_4Cl solution and extracted with ethyl acetate for three times. The organic layer was washed with brine, dried over Na_2SO_4, filtered and concentrated in vacuo. The residue was purified by chromatography on silica gel (PE/EA = 3/1) to give the desired product **L8**.

(4R,4'R,5S,5'S)-2,2'-(1-(naphthalen-1-yl)propane-2,2-diyl)bis(4,5-diphenyl-4,5-dihydrooxazole)

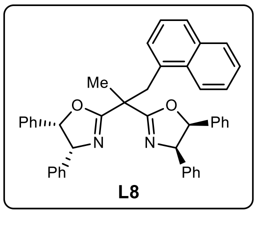

The Ligand **L8** was purified with silica gel chromatography as a white solid (318 mg, 52% yield over

two steps); mp: 157-159 °C. ^{1}H NMR (500 MHz, $CDCl_3$) δ 8.40 (d, J = 8.5 Hz, 1H), 7.93 (d, J = 8.0 Hz, 1H), 7.85 (d, J = 8.1 Hz, 1H), 7.67 (d, J = 6.9 Hz, 1H), 7.62 – 7.50 (m, 3H), 7.07 – 6.90 (m, 20H), 5.84 (dd, J = 10.2, 5.4 Hz, 2H), 5.64 (d, J = 10.1 Hz, 1H), 5.34 (d, J = 10.3 Hz, 1H), 4.31 – 4.18 (m, 2H), 1.89 (s, 3H). ^{13}C NMR (126 MHz, $CDCl_3$) δ 170.3, 168.7, 137.6, 137.5, 136.3, 136.0, 134.1, 133.4, 133.2, 129.1, 129.0, 128.1, 128.0, 127.8, 127.72, 127.70, 127.65, 127.6, 127.5, 127.1, 127.0, 126.9, 126.8, 125.9, 125.5, 125.4, 124.6, 86.6, 86.3, 73.9, 73.8, 45.1, 37.8, 22.7. HRMS (ESI): m/z calcd. for $C_{43}H_{37}N_2O_2^+$ [M + H$^+$]: 613.2850, found: 613.2857.

The ligand **L9** was synthesized according to the reported literature with modifications. [2]

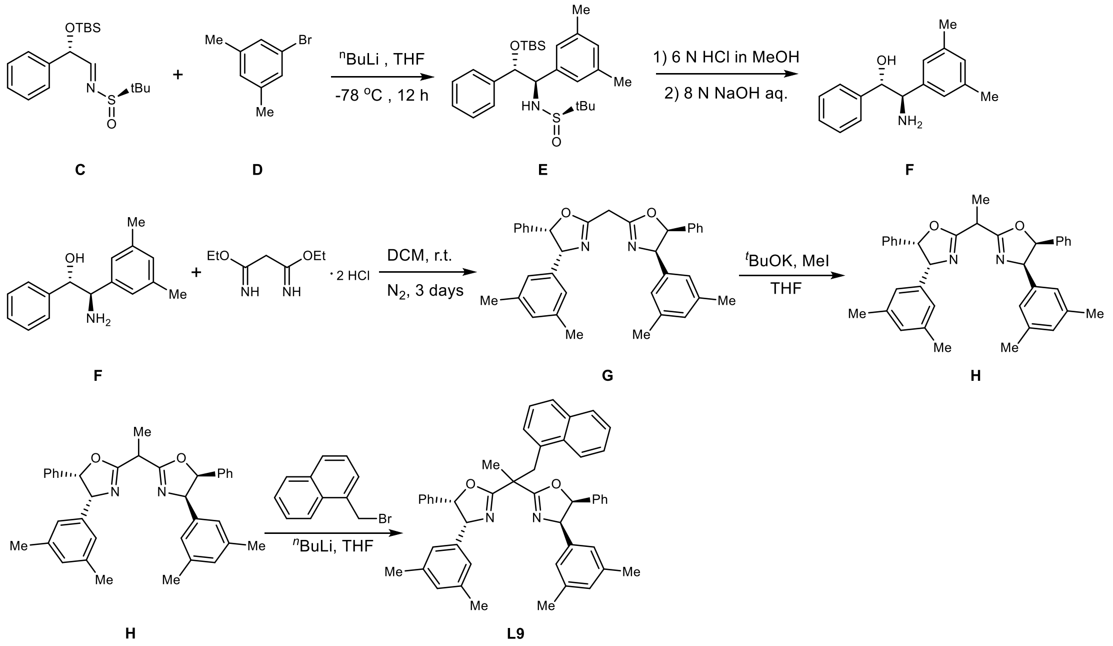

One 100 mL, one-necked Schlenk flask was charged with **D** (3.68 g, 20.0 mmol, 2.0 equiv.) and THF (40 mL) under nitrogen. The solution was cooled to -78 °C, and n-butyllithium (8.3 mL, 2.4 M in hexane, 20.0 mmol, 2.0 equiv.) was added dropwise by syringe. The resulting solution was stirred at -78 °C for 1.5 h. Then compound **C** (4.35 g, 10.0 mmol, 1.0 equiv.) in THF (15 mL) was added to the organolithium solution using a syringe dropwise over 30 min. The resulting mixture was stirred at -78 °C for 12 h. After that the reaction was quenched by the addition of sat. NH_4Cl solution (20 mL) at -78 °C and slowly warmed to 25 °C. The mixture was transferred to a 250 mL separatory funnel. The aqueous layer was extracted with ethyl acetate (3 × 50 mL), and the organic layers were combined, washed with brine, dried over Na_2SO_4, filtered and concentrated in vacuo. The residue was purified by chromatography on silica gel (PE/EA = 10/1) to give the desired product **E** (8.0 mmol, 80% yield).

A 250 mL, one-necked Schlenk flask with an eggshaped stir bar was charged with **E** (8.0 mmol) and methanol (10 mL). HCl solution (60 mL, 6 M in MeOH) was added dropwise by syringe. The reaction was stirred for 2 h at 25 °C, and then the solvent was removed by rotary evaporation. The crude solid was suspended in dichloromethane (50 mL) and 80 mL of NaOH solution (8 M in H_2O) was added while

the solution was stirred vigorously. The solution was stirred for 1 h and then was transferred to a 500 mL separatory funnel. The organic layer was separated, and the aqueous layer was extracted with dichloromethane (3 × 50 mL). The organic layers were combined, dried over Na_2SO_4, filtered and concentrated in vacuo. The residue was purified by recrystallization (hexane) to give the desired amino alcohol **F** (6.0 mmol, 75% yield).

The amino alcohol **F** (6.0 mmol, 2.0 equiv.) and the hydrochloride (3.0 mmol, 1.0 equiv.) was firstly added to a 100 mL oven-dried sealing tube. The vessel was evacuated and backfilled with N_2 (repeated for 3 times). Dichloromethane (30 mL) was added to the reaction tube and stirred at room temperature for 2 days. After that, the reaction was quenched with 20 mL H_2O and extracted with dichloromethane (3 × 20 mL). The organic layers were combined, dried over Na_2SO_4, filtered and concentrated in vacuo. The residue was purified by recrystallization (hexane and ethyl acetate) to give the desired skeleton **G** (2 mmol, 67% yield).

The following steps to synthesize **L9** was similar to **L8**.

(4R,4'R,5S,5'S)-2,2'-(1-(naphthalen-1-yl)propane-2,2-diyl)bis(4-(3,5-dimethylphenyl)-5-phenyl-4,5-dihydrooxazole)

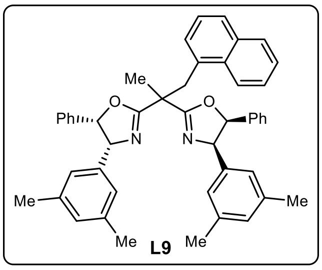

The Ligand **L9** was purified with silica gel chromatography (PE/EA = 3/1) as a white solid (361 mg, 54% yield over two steps); mp: 161-163 °C. ^{1}H NMR (500 MHz, $CDCl_3$) δ 8.40 (d, *J* = 8.4 Hz, 1H), 7.91 (d, *J* = 8.1 Hz, 1H), 7.82 (d, *J* = 8.1 Hz, 1H), 7.69 (d, *J* = 7.3 Hz, 1H), 7.60 – 7.57 (m, 1H), 7.55 – 7.52 (m, 1H), 7.50 – 7.47 (m, 1H), 7.07 – 6.49 (m, 10H), 6.60 (s, 3H), 6.54 (s, 1H), 6.49 (s, 2H), 5.80 (d, *J* = 9.9 Hz, 1H), 5.66 (d, *J* = 10.2 Hz, 1H), 5.54 (d, *J* = 10.1 Hz, 1H), 5.19 (d, *J* = 10.1 Hz, 1H), 4.32 (d, *J* = 14.3 Hz, 1H), 4.15 (d, *J* = 14.4 Hz, 1H), 1.96 (s, 6H), 1.92 (s, 6H), 1.87 (s, 3H). ^{13}C NMR (126 MHz, $CDCl_3$) δ 170.1, 168.3, 137.2, 137.0, 136.8, 136.3, 136.0, 134.0, 133.44, 133.36, 129.1, 128.9, 128.6, 128.4, 127.8, 127.43, 127.36, 127.3, 126.81, 126.79, 125.9, 125.8, 125.7, 125.4, 125.3, 124.6, 86.6, 86.0, 73.7, 73.6, 44.8, 37.9, 22.8, 21.0, 20.9. HRMS (ESI): m/z calcd. for $C_{47}H_{45}N_2O_2^+$ [M + H$^+$]: 669.3476, found: 669.3469.

Synthesis of Secondary Alkyl Bromides and Alkyl Vinyl Iodides

The substrate **S1** was synthesized according to the reported literature. [3]

4-bromo-5,5,5-trifluoropentyl (*E*)-2-(3-(3,4-dimethoxyphenyl)acrylamido)benzoate

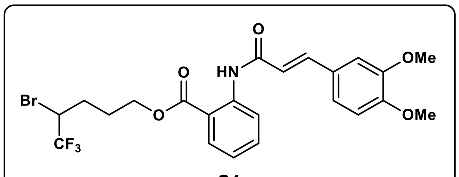

S1

The substrate **S1** was purified with silica gel chromatography (PE/EA = 7/1) as a white solid (688 mg, 65% yield); mp: 103-104 °C. ^{1}H NMR (500 MHz, $CDCl_3$) δ 11.20 (s, 1H), 8.86 (dd, *J* = 8.5, 1.2 Hz, 1H), 7.98 (dd, *J* = 8.1, 1.7 Hz, 1H), 7.66 (d, *J* = 15.5 Hz, 1H), 7.54 (ddd, *J* = 8.7, 7.3, 1.7 Hz, 1H), 7.11 (dd, *J* = 8.3, 2.0 Hz, 1H), 7.08 – 7.04 (m, 2H), 6.83 (d, *J* = 8.3 Hz, 1H), 6.44 (d, *J* = 15.4 Hz, 1H), 4.40– 4.32 (m, 2H), 4.21– 4.14 (m, 1H), 3.91 (s, 3H), 3.87 (s, 3H), 2.26 – 2.19 (m, 1H), 2.17 – 2.10 (m, 1H), 2.05 – 1.99 (m, 1H), 1.98 – 1.89 (m, 1H). ^{19}F NMR (471 MHz, $CDCl_3$) δ -72.14 (d, *J* = 7.6 Hz). ^{13}C NMR (126 MHz, $CDCl_3$) δ 168.3, 164.7, 151.0, 149.2, 142.3, 142.2, 134.9, 130.7, 127.6, 123.9 (q, *J* = 277.5 Hz), 122.6, 122.5, 120.7, 119.5, 114.6, 111.0, 109.7, 63.9, 56.0, 55.9, 46.8 (q, *J* = 32.8 Hz), 28.4, 26.1. HRMS (ESI): m/z calcd. for $C_{23}H_{24}BrF_3NO_5^+$ [M + H^+]: 530.0784, found: 530.0791.

The substrate **S2** was synthesized according to the reported literature with modifications. [4]

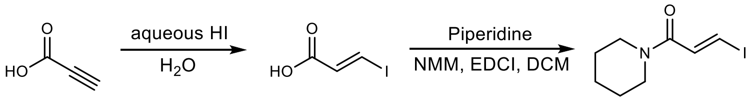

A mixture of propiolic acid (0.80 ml, 20 mmol, 1.0 equiv.) and aqueous HI (12.0 mL ,7 M aqueous solution, 80 mmol, 4.0 equiv.) was heated in three foil-wrapped Ace tubes at 95 °C for 24 hours. The resulting mixtures were allowed to cool to ambient temperature to afford a suspension of the acid in excess aqueous HI. The pressure was released (CARE), then the mixtures were extracted with dichloromethane (3 × 20 mL). The organic layers were combined, dried over Na_2SO_4, filtered and concentrated in vacuo. The crude (*E*)-3-iodoacrylic acid was used directly without further purification.

To an ice-water cooled solution of (*E*)-3-iodoacrylic acid (0.8 g, 4 mmol, 1.0 equiv.), piperidine (0.43 mL, 4.4 mmol, 1.1 equiv.), 4-methyl morpholine (0.5 mL, 4.4 mmol, 1.1 equiv.) in DCM (8 mL) was added EDCI (0.9 g, 4.4 mmol, 1.1 equiv.) in small portions. The reaction was stirred at 0 °C for 2 hours. After completion, the solution was washed with diluted hydrochloric acid and water, and then dried with Na_2SO_4. The residue after concentration in vacuo was purified by flash silica gel chromatography (PE/EA = 5/1) to afford **S2**.

(*E*)-3-iodo-1-(piperidin-1-yl)prop-2-en-1-one

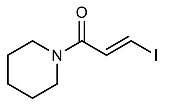

S2

The substrate **S2** was purified with silica gel chromatography as a yellow oil (763 mg, 72% yield). ^{1}H NMR (500 MHz, $CDCl_3$) δ 7.56 (d, *J* = 14.2 Hz, 1H), 7.25 (d, *J* = 14.2 Hz, 1H), 3.56 – 3.43 (m, 4H), 1.65 – 1.54 (m, 6H). ^{13}C NMR (126 MHz, $CDCl_3$) δ 163.6, 136.1, 95.1, 47.0, 43.3, 26.7, 25.5, 24.5. HRMS (EI): m/z calcd. for $C_8H_{12}INO$ [M]: 264.9964, found: 264.9966.

The substrate **S3, S4, S5** was synthesized according to the reported literature with modifications. [5]

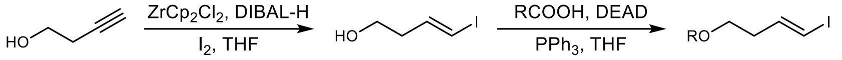

$ZrCp_2Cl_2$ (3.2 g, 11 mmol, 1.1 equiv.) in THF (25 mL) cooled to 0 °C was added slowly a solution of DIBAL-H (1.6 g, 11 mmol, 1.1 equiv.) in THF (5 mL) under N_2. The resultant suspension was stirred for 30 min at 0 °C, followed by addition of a solution of 3-Butyn-1-ol (0.76 mL, 10 mmol, 1.0 equiv.) in THF (5 mL). The mixture was warmed to room temperature and stirred until a homogeneous solution resulted (ca. 1 h) and then cooled to -78 °C, followed by addition of I_2 (3.3 g, 13 mmol, 1.3 equiv.) in THF (15 mL). After 8 h at -78 °C, the reaction mixture was quenched with 1N HCl, extracted with ethyl acetate, washed successively with saturated $Na_2S_2O_3$, $NaHCO_3$ and brine, dried over Na_2SO_4, filtered, and concentrated. The residue was purified by flash silica gel chromatography (PE/EA = 10/1) to give the desired alcohol (3.1 mmol, 31% yield).

To an anhydrous THF (12 mL) solution of acid (3.0 mmol, 1.0 equiv.), (E)-4-iodo-3-buten-1-ol (0.59 g, 3.0 mmol, 1.0 equiv.) and triphenylphosphine (0.87 g, 3.3 mmol, 1.1 equiv.) was slowly added a THF solution (5 mL) of diethyl azodicarboxylate (0.52 mL, 3.3 mmol, 1.1 equiv.) over 30 min at 0 °C. The resulting mixture was then stirred at room temperature for 12 hours. The reaction was quenched with saturated NaCl solution (15 mL) and extracted with ethyl acetate (3 × 20 mL). The combined organic layers were dried over anhydrous Na_2SO_4, filtered, and concentrated. The residue was purified with flash chromatography on silica gel to give the desired product.

(*E*)-4-iodobut-3-en-1-yl 2-(4-(4-chlorobenzoyl)phenoxy)-2-methylpropanoate

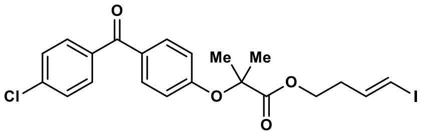

S3

The substrate **S3** was purified with silica gel chromatography (PE/EA = 10/1) as a white solid (926 mg, 62% yield); mp: 105-107 °C. ^{1}H NMR (500 MHz, $CDCl_3$) δ 7.76 – 7.69 (m, 4H), 7.45 – 7.43 (m, 2H), 6.86 – 6.83 (m, 2H), 6.33 (dt, *J* = 14.4, 7.2 Hz, 1H), 6.06 (dt, *J* = 14.4, 1.4 Hz, 1H), 4.20 (t, *J* = 6.3 Hz, 2H), 2.35 (td, *J* = 7.6, 7.0, 5.6 Hz, 2H), 1.67 (s, 6H). ^{13}C NMR (126 MHz, $CDCl_3$) δ 194.3, 173.7, 159.7, 141.2, 138.5, 136.5, 132.3, 131.3, 130.6, 128.7, 117.3, 79.4, 78.0, 63.5, 35.1, 25.6. HRMS (ESI): m/z calcd. for $C_{21}H_{21}ClIO_4^+$ [M + H^+]: 499.0168, found: 499.0180.

(*E*)-7-phenyl-5-(trifluoromethyl)hept-3-en-1-yl 2-(2-fluoro-[1,1'-biphenyl]-4-yl)propanoate

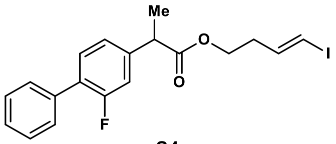

S4

The substrate **S4** was purified with silica gel chromatography (PE/EA = 20/1) as a colorless oil (623 mg, 49% yield). ^{1}H NMR (500 MHz, $CDCl_3$) δ 7.57 – 7.54 (m, 2H), 7.46 – 7.40 (m, 3H), 7.38 – 7.35 (m, 1H), 7.16 – 7.10 (m, 2H), 6.42 (dt, *J* = 14.4, 7.2 Hz, 1H), 6.05 (dt, *J* = 14.4, 1.4 Hz, 1H), 4.19 – 4.09 (m, 2H), 3.75 (q, *J* = 7.1 Hz, 1H), 2.37 – 2.33 (m, 2H), 1.54 (d, *J* = 7.2 Hz, 3H). ^{19}F NMR (471 MHz, $CDCl_3$) δ -117.38 – -117.43 (m). ^{13}C NMR (126 MHz, $CDCl_3$) δ 173.9, 159.8 (d, *J* = 248.3 Hz), 141.7 (d, *J* = 8.1 Hz), 141.6, 135.6, 131.0 (d, *J* = 3.8 Hz), 129.1 (d, *J* = 2.8 Hz), 128.6, 128.0 (d, *J* = 13.8 Hz), 127.8, 123.7 (d, *J* = 3.6 Hz), 115.4 (d, *J* = 23.7 Hz), 77.7, 62.8, 45.1, 35.3, 18.3. HRMS (ESI): m/z calcd. for $C_{19}H_{18}FINaO_2^+$ [M + Na^+]: 447.0228, found: 447.0232.

(*E*)-4-iodobut-3-en-1-yl 2-(1-(4-chlorobenzoyl)-5-methoxy-2-methyl-1H-indol-3-yl)acetate

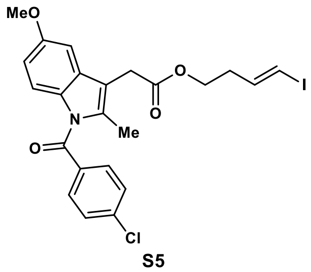

S5

The substrate **S5** was purified with silica gel chromatography (PE/EA = 3/1) as a yellow solid (1.03 g, 64% yield); mp: 60-62 °C. ^{1}H NMR (500 MHz, $CDCl_3$) δ 7.69 – 7.65 (m, 2H), 7.48 – 7.45 (m, 2H), 6.95 (d, *J* = 2.6 Hz, 1H), 6.86 (d, *J* = 9.0 Hz, 1H), 6.67 (dd, *J* = 9.0, 2.6 Hz, 1H), 6.42 (dt, *J* = 14.4, 7.1 Hz, 1H), 6.05 (dt, *J* = 14.5, 1.4 Hz, 1H), 4.13 (t, *J* = 6.4 Hz, 2H), 3.84 (s, 3H), 3.66 (s, 2H), 2.39 – 2.33 (m, 5H). ^{13}C NMR (126 MHz, $CDCl_3$) δ 170.8, 168.4, 156.2, 141.6, 139.3, 136.1, 134.0, 131.3, 130.9, 130.7, 129.2, 115.1, 112.5, 111.7, 101.4, 77.6, 62.9, 55.9, 35.3, 30.4, 13.5. HRMS (ESI): m/z calcd. for $C_{23}H_{21}ClINNaO_4^+$ [M + Na^+]: 560.0096, found: 560.0107.

The substrate **S6** was synthesized according to the reported literature. [6]

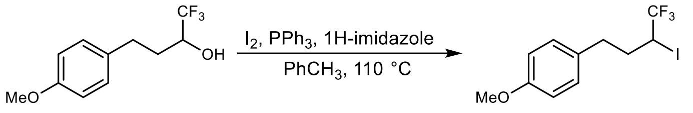

Iodine (685 mg, 2.7 mmol, 2.7 equiv) was added to a solution of alcohol (234 mg, 1.0 mmol, 1.0 equiv), PPh_3 (917mg, 3.5 mmol, 3.5 equiv) and imidazole (184 mg, 2.7 mmol, 2.7 equiv) in toluene (10 mL). The resulting mixture was stirred at 110 °C for 3 h. After cooling to room temperature, the reaction was quenched with water. The aqueous phase was extracted with ethyl acetate for three times. The combined organic layers were washed with saturated $Na_2S_2O_3$ and brine, dried over Na_2SO_4 and concentrated. The crude product was purified by flash chromatography on silica gel to give desired fluorinated alkyl iodide.

1-methoxy-4-(4,4,4-trifluoro-3-iodobutyl)benzene

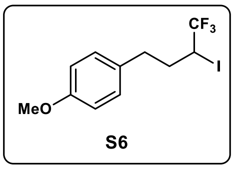

The substrate **S6** was purified with silica gel chromatography (PE/EA = 50/1) as a colorless oil (210 mg, 61% yield). ^{1}H NMR (500 MHz, $CDCl_3$) δ 7.16 – 7.13 (m, 2H), 6.89 – 6.85 (m, 2H), 4.05 – 3.98 (m, 1H), 3.81 (s, 3H), 2.93 (dt, J = 13.3, 6.3 Hz, 1H), 2.64 (dt, J = 14.0, 8.2 Hz, 1H), 2.14 (td, J = 8.1, 6.5 Hz, 2H). ^{19}F NMR (471 MHz, $CDCl_3$) δ -68.70 (d, J = 7.2 Hz). ^{13}C NMR (126 MHz, $CDCl_3$) δ 158.5, 131.2, 129.6, 124.8 (q, J = 276.5 Hz), 114.3, 55.4, 34.5, 33.6, 23.8 (q, J = 31.0 Hz). HRMS (ESI): m/z calcd. for $C_{11}H_{13}F_3IO^+$ [M + H$^+$]: 344.9958, found: 344.9945.

General Procedure for Nickel-catalyzed Asymmetric Reductive Trifluoroalkylation of Vinyl Iodides

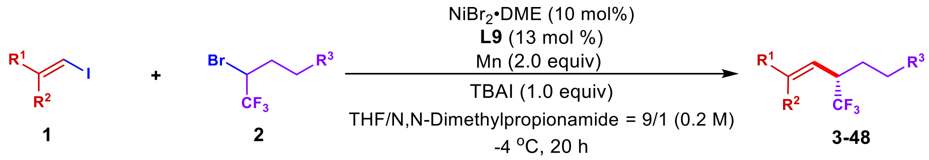

$NiBr_2$•DME (10 mol%, 0.01 mmol, 3.1 mg), **L9** (13 mol%, 0.013 mmol, 8.7 mg), Mn powder (2.0 equiv, 0.20 mmol, 11.0 mg), TBAI (1.0 equiv, 0.10 mmol, 36.9 mg) and vinyl iodide **1** (1.0 equiv, 0.10 mmol) were firstly combined in a 10 mL oven-dried sealing tube. The vessel was evacuated and backfilled with N_2 (repeated for 3 times). Alkyl bromide **2** (2.0 equiv, 0.20 mmol), THF (0.45 mL) and N,N-Dimethylpropionamide (0.05 mL) were added via syringe. The tube was sealed with a Teflon lined cap and stirred at -4 ˚C for 20 h. The reaction mixture was then diluted with ethyl acetate (~20 mL) and filtered through a pad of celite. The filtrate was added brine (20 mL) and extracted with ethyl acetate (3 × 15 mL), the combined organic layer was dried over Na_2SO_4, filtrated and concentrated under vacuum. The residue was then purified by flash column chromatography to give desired products.

methyl (*E*)-4-(5-phenyl-3-(trifluoromethyl)pent-1-en-1-yl)benzoate

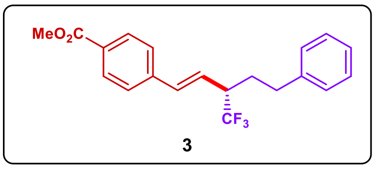

The product **3** was purified with silica gel chromatography (PE/EA = 30/1) as a colorless oil (31.2 mg, 90% yield). ^{1}H NMR (500 MHz, $CDCl_3$) δ 8.04 (d, *J* = 8.3 Hz, 2H), 7.47 (d, *J* = 8.3 Hz, 2H), 7.32 (t, *J* = 7.5 Hz, 2H), 7.23 (t, *J* = 7.4 Hz, 1H), 7.19 (d, *J* = 6.8 Hz, 2H), 6.61 (d, *J* = 15.8 Hz, 1H), 6.15 (dd, *J* = 15.9, 9.3 Hz, 1H), 3.94 (s, 3H), 2.94 – 2.87 (m, 1H), 2.84 – 2.78 (m, 1H), 2.64 – 2.58 (m, 1H), 2.25 – 2.18 (m, 1H), 1.98 – 1.90 (m, 1H). ^{19}F NMR (471 MHz, $CDCl_3$) δ -70.43 (d, *J* = 8.6 Hz). ^{13}C NMR (126 MHz, $CDCl_3$) δ 166.9, 140.6, 140.6, 135.6, 130.1, 129.7, 128.7, 128.5, 126.9 (q, *J* = 280.7 Hz) 126.5, 126.4, 125.4 (q, *J* = 2.7 Hz), 52.2, 47.3 (q, *J* = 26.8 Hz), 32.5, 29.4. HRMS (ESI): m/z calcd. for $C_{20}H_{20}F_3O_2^+$ [M + H$^+$]: 349.1410, found: 349.1410. $[\alpha]_D^{20}$ = 1.2 (c = 1.0, $CHCl_3$), HPLC chiralcel OD-H column (2% isopropanol in hexanes, 0.5 mL/min, λ = 254 nm), t_R = 35.1 min (minor), 39.5 min (major), 95% *ee*.

methyl (*E*)-3-(5-phenyl-3-(trifluoromethyl)pent-1-en-1-yl)benzoate

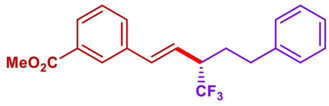

The product **4** was purified with silica gel chromatography (PE/EA = 30/1) as a colorless oil (26.9 mg, 77% yield). ^{1}H NMR (400 MHz, $CDCl_3$) δ 8.10 (t, J = 1.8 Hz, 1H), 7.96 (dt, J = 7.7, 1.4 Hz, 1H), 7.59 (dt, J = 7.7, 1.6 Hz, 1H), 7.43 (t, J = 7.7 Hz, 1H), 7.31 (t, J = 7.4 Hz, 2H), 7.24 – 7.17 (m, 3H), 6.60 (d, J = 15.9 Hz, 1H), 6.10 (dd, J = 15.9, 9.4 Hz, 1H), 3.95 (s, 3H), 2.94 – 2.76 (m, 2H), 2.63 – 2.56 (m, 1H), 2.23 – 2.15 (m, 1H), 1.97 – 1.87 (m, 1H). ^{19}F NMR (376 MHz, $CDCl_3$) δ -70.58 (d, J = 8.8 Hz). ^{13}C NMR (101 MHz, $CDCl_3$) δ 167.0, 140.7, 136.6, 135.5, 131.0, 130.8, 129.3, 128.9, 128.7, 128.6, 127.6, 126.9 (q, J = 280.8 Hz),126.4, 124.1 (q, J = 2.6 Hz), 52.4, 47.3 (q, J = 26.8 Hz), 32.6, 29.4 (q, J = 2.0 Hz). HRMS (EI): m/z calcd. for $C_{20}H_{19}F_3O_2$ [M]: 348.1337, found: 348.1330. $[\alpha]_D^{20}$ = 0.3 (c = 1.0, $CHCl_3$), HPLC chiralcel AD-H column (4% isopropanol in hexanes, 1.0 mL/min, λ = 254 nm), t_R = 7.0 min (minor), 8.4 min (major), 94% *ee*.

(*E*)-1-(4-(5-phenyl-3-(trifluoromethyl)pent-1-en-1-yl)phenyl)ethan-1-one

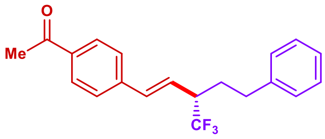

The product **5** was purified with silica gel chromatography (PE/EA = 30/1) as a colorless oil (27.6 mg, 83% yield). ^{1}H NMR (400 MHz, $CDCl_3$) δ 7.95 (d, J = 8.3 Hz, 2H), 7.49 (d, J = 8.4 Hz, 2H), 7.31 (t, J = 7.5 Hz, 2H), 7.25 – 7.15 (m, 3H), 6.61 (d, J = 15.9 Hz, 1H), 6.14 (dd, J = 15.9, 9.4 Hz, 1H), 2.94 – 2.86 (m, 1H), 2.83 – 2.76 (m, 1H), 2.65 – 2.56 (m, 4H), 2.24 – 2.16 (m, 1H), 1.98 – 1.88 (m, 1H). ^{19}F NMR (376 MHz, $CDCl_3$) δ -70.44 (d, J = 8.4 Hz). ^{13}C NMR (101 MHz, $CDCl_3$) δ 197.6, 140.8, 140.6, 136.7, 135.5, 129.0, 128.7, 128.5, 126.8 (q, J = 280.5 Hz), 126.8, 126.5, 125.7 (q, J = 2.4 Hz), 47.3 (q, J = 27.0 Hz), 32.6, 29.4, 26.8. HRMS (ESI): m/z calcd. for $C_{20}H_{20}F_3O^+$ [M + H^+]: 333.1461, found: 333.1461. $[\alpha]_D^{20}$ = 2.4 (c = 2.0, $CHCl_3$), HPLC chiralcel AD-H column (10% isopropanol in hexanes, 1.0 mL/min, λ = 254 nm), t_R = 10.1 min (minor), 15.4 min (major), 95% *ee*.

(*E*)-4-(5-phenyl-3-(trifluoromethyl)pent-1-en-1-yl)benzonitrile

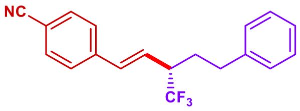

The product **6** was purified with silica gel chromatography (PE/EA = 30/1) as a colorless oil (27.1 mg, 86% yield). ^{1}H NMR (500 MHz, $CDCl_3$) δ 7.64 (d, *J* = 8.4 Hz, 2H), 7.48 (d, *J* = 8.4 Hz, 2H), 7.31 (t, *J* = 7.5 Hz, 2H), 7.22 (t, *J* = 7.4 Hz, 1H), 7.17 (d, *J* = 6.7 Hz, 2H), 6.57 (d, *J* = 15.9 Hz, 1H), 6.14 (dd, *J* = 15.9, 9.4 Hz, 1H), 2.98 – 2.83 (m, 1H), 2.81 – 2.75 (m, 1H), 2.62 – 2.56 (m, 1H), 2.24– 2.17 (m, 1H), 1.96– 1.88 (m, 1H). ^{19}F NMR (471 MHz, $CDCl_3$) δ -70.40 (d, *J* = 8.5 Hz). ^{13}C NMR (126 MHz, $CDCl_3$) δ 140.6, 140.4, 134.9, 132.7, 128.8, 128.5, 127.2, 126.8 (q, *J* = 2.9 Hz), 126.7 (q, *J* = 280.7 Hz) 126.5, 118.9, 111.6, 47.3 (q, *J* = 26.9 Hz)., 32.5, 29.3. HRMS (ESI): m/z calcd. for $C_{19}H_{17}F_3N^+$ [M + H^+]: 316.1308, found: 316.1335. $[\alpha]_D^{20}$ = 1.0 (c = 1.0, $CHCl_3$), HPLC chiralcel OD-H column (4% isopropanol in hexanes, 1.0 mL/min, λ = 254 nm), t_R = 15.0 min (minor), 20.3 min (major), 95% *ee*.

(*E*)-1-fluoro-4-(5-phenyl-3-(trifluoromethyl)pent-1-en-1-yl)benzene

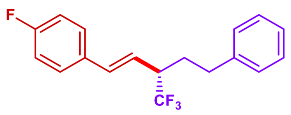

7

The product **7** was purified with silica gel chromatography (PE/EA = 100/1) as a colorless oil (24.6 mg, 80% yield). ^{1}H NMR (400 MHz, $CDCl_3$) δ 7.42 – 7.35 (m, 2H), 7.33 – 7.28 (m, 2H), 7.25 – 7.14 (m, 3H), 7.10 – 7.00 (m, 2H), 6.53 (d, *J* = 15.9 Hz, 1H), 5.92 (dd, *J* = 15.8, 9.4 Hz, 1H), 2.91 – 2.76 (m, 2H), 2.63 – 2.55 (m, 1H), 2.22 – 2.13 (m, 1H), 1.94 – 1.85 (m, 1H). ^{19}F NMR (376 MHz, $CDCl_3$) δ -70.67 (d, *J* = 9.4 Hz), -113.50 – -113.58 (m). ^{13}C NMR (151 MHz, $CDCl_3$) δ 162.8 (d, *J* = 247.6 Hz), 140.7, 135.3, 132.5 (q, *J* = 3.2 Hz), 128.7, 128.6, 128.2 (d, *J* = 7.9 Hz), 127.0 (d, *J* = 279.9 Hz), 126.4, 122.5, 115.8 (d, *J* = 21.6 Hz), 47.2 (q, *J* = 26.7 Hz), 32.6, 29.4. HRMS (EI): m/z calcd. for $C_{18}H_{16}F_4$ [M]: 308.1188, found: 308.1187. $[\alpha]_D^{20}$ = 1.3 (c = 1.0, $CHCl_3$), HPLC chiralcel OD-H column (1% isopropanol in hexanes, 1.0 mL/min, λ = 254 nm), t_R = 7.1 min (minor), 8.5 min (major), 94% *ee*.

(*E*)-1-chloro-4-(5-phenyl-3-(trifluoromethyl)pent-1-en-1-yl)benzene

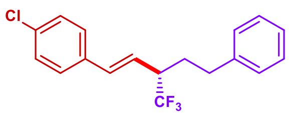

8

The product **8** was purified with silica gel chromatography (PE/EA = 100/1) as a colorless oil (17.3 mg, 53% yield). ^{1}H NMR (400 MHz, $CDCl_3$) δ 7.36 – 7.27 (m, 6H), 7.24 – 7.20 (m, 1H), 7.19 – 7.16 (m, 2H), 6.52 (d, *J* = 15.8 Hz, 1H), 5.98 (dd, *J* = 15.9, 9.3 Hz, 1H), 2.91 – 2.75 (m, 2H), 2.63 – 2.55 (m, 1H), 2.22 – 2.14 (m, 1H), 1.95 – 1.85 (m, 1H). ^{19}F NMR (376 MHz, $CDCl_3$) δ -70.60 (d, *J* = 8.2 Hz). ^{13}C NMR (101 MHz, $CDCl_3$) δ 140.7, 135.3, 134.8, 134.0, 129.0, 128.7, 128.5, 127.9, 126.9 (q, *J* = 280.8

Hz), 126.4, 123.4 (q, J = 2.7 Hz), 47.3 (q, J = 26.8 Hz), 32.6, 29.4 (q, J = 2.1 Hz). HRMS (EI): m/z calcd. for $C_{18}H_{16}ClF_3$ [M]: 324.0893, found: 324.0891. $[\alpha]_D^{20}$ = 1.8 (c = 1.0, $CHCl_3$), HPLC chiralcel OD-H column (1% isopropanol in hexanes, 1.0 mL/min, λ = 254 nm), t_R = 8.0 min (minor), 9.3 min (major), 94% *ee*.

(*E*)-1-chloro-3-(5-phenyl-3-(trifluoromethyl)pent-1-en-1-yl)benzene

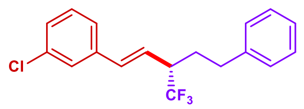

9

The product **9** was purified with silica gel chromatography (PE/EA = 100/1) as a colorless oil (28.1 mg, 87% yield). ^{1}H NMR (500 MHz, $CDCl_3$) δ 7.41 – 7.40 (m, 1H), 7.33 – 7.29 (m, 2H), 7.28 – 7.25 (m, 3H), 7.26 – 7.19 (m, 1H), 7.21 – 7.15 (m, 2H), 6.51 (d, J = 15.8 Hz, 1H), 6.03 (dd, J = 15.9, 9.3 Hz, 1H), 2.90 – 2.76 (m, 2H), 2.62 – 2.55 (m, 1H), 2.22 – 2.15 (m, 1H), 1.94 – 1.87 (m, 1H). ^{19}F NMR (471 MHz, $CDCl_3$) δ -70.57 (d, J = 8.7 Hz). ^{13}C NMR (126 MHz, $CDCl_3$) δ 140.6, 138.1, 135.2, 134.8, 130.1, 128.7, 128.6, 128.3, 126.9 (q, J = 280.1 Hz), 126.6, 126.5, 124.9, 124.3 (q, J = 2.7 Hz), 47.2 (q, J = 26.8 Hz), 32.5, 29.4. HRMS (EI): m/z calcd. for $C_{18}H_{16}ClF_3$ [M]: 324.0893, found: 324.0893. $[\alpha]_D^{20}$ = 1.9 (c = 1.0, $CHCl_3$), HPLC chiralcel AD-H column (1% isopropanol in hexanes, 0.5 mL/min, λ = 254 nm), t_R = 9.7 min (minor), 11.2 min (major), 94% *ee*.

(*E*)-1-(5-phenyl-3-(trifluoromethyl)pent-1-en-1-yl)-4-(trifluoromethyl)benzene

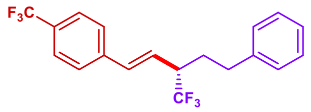

10

The product **10** was purified with silica gel chromatography (PE/EA = 100/1) as a colorless oil (28.0 mg, 78% yield). ^{1}H NMR (500 MHz, $CDCl_3$) δ 7.61 (d, J = 8.1 Hz, 2H), 7.51 (d, J = 8.1 Hz, 2H), 7.32 (t, J = 7.5 Hz, 2H), 7.25 – 7.21 (m, 1H), 7.19 – 7.18 (m, 2H), 6.60 (d, J = 15.8 Hz, 1H), 6.12 (dd, J = 15.9, 9.4 Hz, 1H), 2.95 – 2.85 (m, 1H), 2.83 – 2.77 (m, 1H), 2.64 – 2.58 (m, 1H), 2.25 – 2.18 (m, 1H), 1.97 – 1.89 (m, 1H). ^{19}F NMR (471 MHz, $CDCl_3$) δ -62.58, -70.51 (d, J = 8.2 Hz). ^{13}C NMR (126 MHz, $CDCl_3$) δ 140.6, 139.7, 135.2, 130.2 (q, J = 32.3 Hz), 128.8, 128.6, 126.9, 126.9 (q, J = 280.4 Hz), 126.5, 125.8 (q, J = 3.7 Hz), 125.5 (q, J = 2.7 Hz), 124.2 (q, J = 271.8 Hz), 47.3 (q, J = 27.0 Hz), 32.6, 29.4. HRMS (EI): m/z calcd. for $C_{19}H_{16}F_6$ [M]: 358.1156, found: 358.1154. $[\alpha]_D^{20}$ = 0.8 (c = 1.0, $CHCl_3$), HPLC chiralcel OD-H column (1% isopropanol in hexanes, 1.0 mL/min, λ = 254 nm), t_R = 6.6 min (minor), 8.1 min (major), 95% *ee*.

(*E*)-1-methoxy-4-(5-phenyl-3-(trifluoromethyl)pent-1-en-1-yl)benzene

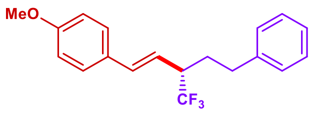

11

The product **11** was purified with silica gel chromatography (PE/EA = 30/1) as a colorless oil (17.1 mg, 53% yield). ^{1}H NMR (500 MHz, $CDCl_3$) δ 7.38 – 7.33 (m, 2H), 7.30 (t, *J* = 7.5 Hz, 2H), 7.25 – 7.13 (m, 3H), 6.95 – 6.83 (m, 2H), 6.51 (d, *J* = 15.8 Hz, 1H), 5.86 (dd, *J* = 15.8, 9.3 Hz, 1H), 3.83 (s, 3H), 2.85 – 2.76 (m, 2H), 2.69 – 2.50 (m, 1H), 2.19 – 2.12 (m, 1H), 1.92 – 1.84 (m, 1H). ^{19}F NMR (471 MHz, $CDCl_3$) δ -70.74. ^{13}C NMR (126 MHz, $CDCl_3$) δ 159.7, 140.9, 135.9, 129.1, 128.7, 128.6, 127.9, 127.1 (q, *J* = 280.5 Hz), 126.3, 120.4 (q, *J* = 2.5 Hz), 114.2, 55.5, 47.3 (q, *J* = 26.5 Hz), 32.6, 29.6. HRMS (EI): m/z calcd. for $C_{19}H_{19}F_3O$ [M]: 320.1388, found: 320.1383. $[\alpha]_D^{20}$ = 1.2 (c = 1.0, $CHCl_3$), HPLC chiralcel OD-H column (2% isopropanol in hexanes, 0.5 mL/min, λ = 254 nm), t_R = 15.1 min (minor), 20.7 min (major), 88% *ee*.

(*E*)-1-methoxy-3-(5-phenyl-3-(trifluoromethyl)pent-1-en-1-yl)benzene

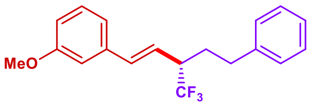

12

The product **12** was purified with silica gel chromatography (PE/EA = 30/1) as a colorless oil (28.3 mg, 88% yield). ^{1}H NMR (500 MHz, $CDCl_3$) δ 7.33 – 7.26 (m, 3H), 7.24 – 7.16 (m, 3H), 7.02 (d, *J* = 7.6 Hz, 1H), 6.95 (t, *J* = 2.0 Hz, 1H), 6.86 (ddd, *J* = 8.2, 2.6, 0.9 Hz, 1H), 6.55 (d, *J* = 15.8 Hz, 1H), 6.01 (dd, *J* = 15.8, 9.3 Hz, 1H), 3.85 (s, 3H), 2.90 – 2.77 (m, 2H), 2.62 – 2.56 (m, 1H), 2.21 – 2.14 (m, 1H), 1.94 – 1.86 (m, 1H). ^{19}F NMR (471 MHz, $CDCl_3$) δ -70.62 (d, *J* = 8.7 Hz). ^{13}C NMR (126 MHz, $CDCl_3$) δ 160.0, 140.8, 137.7, 136.4, 129.8, 128.7, 128.6, 127.0 (q, *J* = 280.1 Hz), 126.4, 123.0 (q, *J* = 2.8 Hz), 119.3, 113.9, 112.0, 55.4, 47.2 (q, *J* = 26.7 Hz), 32.5, 29.5. HRMS (ESI): m/z calcd. for $C_{19}H_{20}F_3O^+$ [M + H$^+$]: 321.1461, found: 321.1438. $[\alpha]_D^{20}$ = 0.9 (c = 1.0, $CHCl_3$), HPLC chiralcel AD-H column (2% isopropanol in hexanes, 1.0 mL/min, λ = 254 nm), t_R = 5.6 min (minor), 6.7 min (major), 94% *ee*.

(*E*)-(3-(trifluoromethyl)pent-1-ene-1,5-diyl)dibenzene

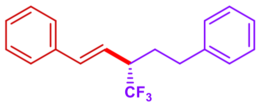

13

The product **13** was purified with silica gel chromatography (PE/EA = 100/1) as a colorless oil (18.9 mg, 65% yield). ^{1}H NMR (400 MHz, $CDCl_3$) δ 7.45 – 7.40 (m, 2H), 7.41 – 7.34 (m, 2H), 7.34 – 7.29 (m, 3H), 7.25 – 7.22 (m, 1H), 7.21 – 7.17 (m, 2H), 6.58 (d, J = 15.9 Hz, 1H), 6.02 (dd, J = 15.9, 9.4 Hz, 1H), 2.92 – 2.77 (m, 2H), 2.64 – 2.56 (m, 1H), 2.25 – 2.14 (m, 1H), 1.96 – 1.86 (m, 1H). ^{19}F NMR (376 MHz, $CDCl_3$) δ -70.65 (d, J = 8.8 Hz). ^{13}C NMR (101 MHz, $CDCl_3$) δ 140.8, 136.5, 136.3, 128.8, 128.7, 128.6, 128.3, 127.0 (q, J = 280.9 Hz), 126.7, 126.4, 122.7 (q, J = 2.6 Hz), 47.3 (q, J = 26.6 Hz), 32.6, 29.5 (q, J = 2.0 Hz). HRMS (EI): m/z calcd. for $C_{18}H_{17}F_3$ [M]: 290.1282, found: 290.1283. $[\alpha]_D^{20}$ = 0.4 (c = 1.0, $CHCl_3$), HPLC chiralcel OD-H column (1% isopropanol in hexanes, 1.0 mL/min, λ = 254 nm), t_R = 7.1 min (major), 9.4 min (minor), 92% *ee*.

(*E*)-2-(5-phenyl-3-(trifluoromethyl)pent-1-en-1-yl)naphthalene

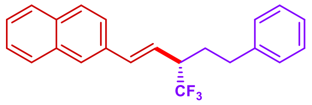

14

The product **14** was purified with silica gel chromatography (PE/EA = 60/1) as a white solid (18.7 mg, 55% yield); mp: 49-50 °C. ^{1}H NMR (500 MHz, $CDCl_3$) δ 7.85 – 7.82 (m, 3H), 7.77 (s, 1H), 7.63 (dd, J = 8.6, 1.7 Hz, 1H), 7.52 – 7.46 (m, 2H), 7.32 (t, J = 7.5 Hz, 2H), 7.25 – 7.21 (m, 3H), 6.74 (d, J = 15.8 Hz, 1H), 6.14 (dd, J = 15.8, 9.4 Hz, 1H), 2.97 – 2.89 (m, 1H), 2.88 – 2.81 (m, 1H), 2.66 – 2.60 (m, 1H), 2.25 – 2.18 (m, 1H), 1.99 – 1.94 (m, 1H). ^{19}F NMR (471 MHz, $CDCl_3$) δ -70.56 (d, J = 8.8 Hz). ^{13}C NMR (126 MHz, $CDCl_3$) δ 140.8, 136.6, 133.7, 133.6, 133.3, 128.7, 128.6, 128.5, 128.2, 127.9, 127.1 (q, J = 280.5 Hz), 126.8, 126.6, 126.4, 126.3, 123.5, 123.0 (q, J = 2.7 Hz), 47.4 (q, J = 26.7 Hz), 32.6, 29.5. HRMS (ESI): m/z calcd. for $C_{22}H_{20}F_3^+$ [M + H$^+$]: 341.1512, found: 341.1519. $[\alpha]_D^{20}$ = 0.2 (c = 1.0, $CHCl_3$), HPLC chiralcel OD-H column (2% isopropanol in hexanes, 1.0 mL/min, λ = 254 nm), t_R = 11.7 min (minor), 21.6 min (major), 96% *ee*.

(*E*)-1-methyl-2-(5-phenyl-3-(trifluoromethyl)pent-1-en-1-yl)benzene

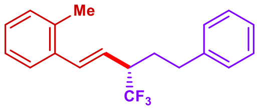

15

The product **15** was purified with silica gel chromatography (PE/EA = 100/1) as a colorless oil (20.6 mg, 68% yield). ^{1}H NMR (500 MHz, $CDCl_3$) δ 7.49 – 7.46 (m, 1H), 7.33 (t, J = 7.5 Hz, 2H), 7.26 – 7.18 (m, 6H), 6.80 (d, J = 15.8 Hz, 1H), 5.90 (dd, J = 15.7, 9.4 Hz, 1H), 2.94 – 2.81 (m, 2H), 2.66 – 2.60 (m, 1H), 2.39 (s, 3H), 2.24 – 2.16 (m, 1H), 1.96 – 1.89 (m, 1H). ^{19}F NMR (471 MHz, $CDCl_3$) δ -70.71 (d, J = 9.5

Hz). ^{13}C NMR (126 MHz, $CDCl_3$) δ 140.8, 135.7, 135.6, 134.6, 130.5, 128.7, 128.6, 128.2, 127.0 (q, *J* = 280.4 Hz), 126.40, 126.35, 126.1, 124.2 (q, *J* = 2.6 Hz), 47.4 (q, *J* = 26.7 Hz), 32.6, 29.4 (q, *J* = 1.9 Hz), 19.9. HRMS (EI): m/z calcd. for $C_{19}H_{19}F_3$ [M]: 304.1439, found: 304.1439. $[\alpha]_D^{20}$ = 1.2 (c = 1.0, $CHCl_3$), HPLC chiralcel OD-H column (1% isopropanol in hexanes, 0.5 mL/min, λ = 254 nm), t_R = 14.3 min (major), 15.7 min (minor), 95% *ee*.

(*E*)-1-methyl-3-(5-phenyl-3-(trifluoromethyl)pent-1-en-1-yl)benzene

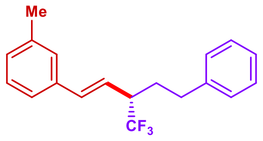

16

The product **16** was purified with silica gel chromatography (PE/EA = 100/1) as a colorless oil (16.0 mg, 53% yield). ^{1}H NMR (500 MHz, $CDCl_3$) δ 7.33 – 7.31 (m, 2H), 7.27 – 7.21 (m, 4H), 7.19 (d, *J* = 6.9 Hz, 2H), 7.12 (d, *J* = 7.2 Hz, 1H), 6.55 (d, *J* = 15.8 Hz, 1H), 6.00 (dd, *J* = 15.8, 9.3 Hz, 1H), 2.90 – 2.77 (m, 2H), 2.62 – 2.56 (m, 1H), 2.38 (s, 3H), 2.21 – 2.14 (m, 1H), 1.94 – 1.86 (m, 1H). ^{19}F NMR (471 MHz, $CDCl_3$) δ -70.68 (d, *J* = 8.7 Hz). ^{13}C NMR (126 MHz, $CDCl_3$) δ 140.9, 138.4, 136.6, 136.3, 129.1, 128.72, 128.68, 128.6, 127.3, 127.1 (q, *J* = 280.4 Hz), 126.4, 123.9, 122.5 (q, *J* = 2.6 Hz), 47.3 (q, *J* = 26.7 Hz), 32.6, 29.5 (q, *J* = 2.4 Hz), 21.5. HRMS (EI): m/z calcd. for $C_{19}H_{19}F_3$ [M]: 304.1439, found: 304.1438. $[\alpha]_D^{20}$ = 0.8 (c = 1.0, $CHCl_3$), HPLC chiralcel AD-H column (1% isopropanol in hexanes, 0.5 mL/min, λ = 254 nm), t_R = 8.6 min (minor), 9.7 min (major), 93% *ee*.

(*E*)-1-methyl-4-(5-phenyl-3-(trifluoromethyl)pent-1-en-1-yl)benzene

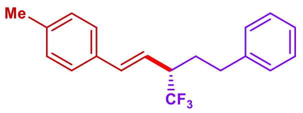

17

The product **17** was purified with silica gel chromatography (PE/EA = 100/1) as a colorless oil (17.7 mg, 58% yield). ^{1}H NMR (500 MHz, $CDCl_3$) δ 7.37 – 7.27 (m, 4H), 7.22 (t, *J* = 7.4 Hz, 1H), 7.18 (t, *J* = 6.9 Hz, 4H), 6.54 (d, *J* = 15.8 Hz, 1H), 5.96 (dd, *J* = 15.8, 9.3 Hz, 1H), 2.89 – 2.77 (m, 2H), 2.62 – 2.56 (m, 1H), 2.37 (s, 3H), 2.20 – 2.14 (m, 1H), 1.93 – 1.85 (m, 1H). ^{19}F NMR (376 MHz, $CDCl_3$) δ -70.71 (d, *J* = 8.8 Hz). ^{13}C NMR (126 MHz, $CDCl_3$) δ 140.9, 138.2, 136.4, 133.6, 129.5, 128.7, 128.6, 127.1 (q, J = 280.4 Hz), 126.6, 126.3, 121.7 (q, *J* = 2.6 Hz), 47.3 (q, *J* = 26.7 Hz), 32.6, 29.6 (q, *J* = 2.3 Hz), 21.4. HRMS (EI): m/z calcd. for $C_{19}H_{19}F_3$ [M]: 304.1439, found: 304.1437. $[\alpha]_D^{20}$ = 1.7 (c = 1.0, $CHCl_3$), HPLC chiralcel AD-H column (0.4% isopropanol in hexanes, 0.3 mL/min, λ = 254 nm), t_R = 21.2 min (major), 23.6 min (minor), 93% *ee*.

(*E*)-1-(tert-butyl)-4-(5-phenyl-3-(trifluoromethyl)pent-1-en-1-yl)benzene

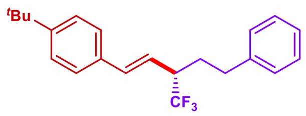

18

The product **18** was purified with silica gel chromatography (PE/EA = 100/1) as a colorless oil (23.5 mg, 68% yield). ^{1}H NMR (500 MHz, $CDCl_3$) δ 7.42 – 7.34 (m, 4H), 7.31 (t, *J* = 7.5 Hz, 2H), 7.22 (t, *J* = 7.4 Hz, 1H), 7.18 (d, *J* = 6.7 Hz, 2H), 6.56 (d, *J* = 15.8 Hz, 1H), 5.98 (dd, *J* = 15.9, 9.3 Hz, 1H), 2.89 – 2.76 (m, 2H), 2.61 – 2.55 (m, 1H), 2.20 – 2.13 (m, 1H), 1.93 – 1.85 (m, 1H), 1.34 (s, 9H). ^{19}F NMR (471 MHz, $CDCl_3$) δ -70.73 (d, *J* = 8.7 Hz). ^{13}C NMR (126 MHz, $CDCl_3$) δ 151.5, 140.9, 136.3, 133.6, 128.7, 128.6, 127.1 (q, *J* = 280.6 Hz), 126.40, 126.35, 125.8, 121.9 (q, *J* = 2.7 Hz), 47.3 (q, *J* = 26.8 Hz), 34.8, 32.5, 31.4, 29.5. HRMS (EI): m/z calcd. for $C_{22}H_{25}F_3$ [M]: 346.1908, found: 346.1907. $[\alpha]_D^{20}$ = 0.7 (c = 1.0, $CHCl_3$), HPLC chiralcel AD-H column (1% isopropanol in hexanes, 1.0 mL/min, λ = 254 nm), t_R = 4.8 min (minor), 5.4 min (major), 92% *ee*.

(*E*)-(3-(trifluoromethyl)undec-4-en-1-yl)benzene

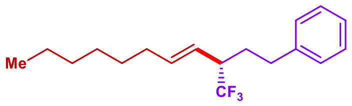

19

The product **19** was purified with silica gel chromatography (Hexane) as a colorless oil (12.0 mg, 40% yield). ^{1}H NMR (500 MHz, $CDCl_3$) δ 7.31 (t, *J* = 7.5 Hz, 2H), 7.25 – 7.15 (m, 3H), 5.68 (dt, *J* = 15.3, 6.8 Hz, 1H), 5.27 (dd, *J* = 15.3, 9.2 Hz, 1H), 2.79 – 2.73 (m, 1H), 2.69 – 2.59 (m, 1H), 2.56 – 2.50 (m, 1H), 2.13 – 2.02 (m, 3H), 1.80 – 1.72 (m, 1H), 1.47 – 1.39 (m, 2H), 1.37 – 1.28 (m, 6H), 0.91 (t, *J* = 6.7 Hz, 3H). ^{19}F NMR (471 MHz, $CDCl_3$) δ -71.17 (d, *J* = 8.6 Hz). ^{13}C NMR (126 MHz, $CDCl_3$) δ 141.2, 138.4, 128.6, 128.6, 127.2 (q, *J* = 280.0 Hz), 126.3, 123.1 (q, *J* = 2.7 Hz), 47.0 (q, *J* = 26.3 Hz), 32.7, 32.5, 31.8, 29.4, 29.1, 28.9, 22.8, 14.2. HRMS (EI): m/z calcd. for $C_{18}H_{25}F_3$ [M]: 298.1908, found: 298.1904. $[\alpha]_D^{20}$ = 0.1 (c = 1.0, $CHCl_3$), HPLC chiralcel OD-H column (0.5% isopropanol in hexanes, 0.5 mL/min, λ = 214 nm), t_R = 9.1 min (minor), 9.8 min (major), 92% *ee*.

(*E*)-(5-cyclohexyl-3-(trifluoromethyl)pent-4-en-1-yl)benzene

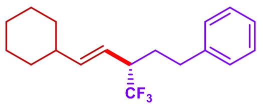

20

The product **20** was purified with silica gel chromatography (Hexane) as a colorless oil (9.8 mg, 33% yield). ^{1}H NMR (500 MHz, $CDCl_3$) δ 7.30 (t, *J* = 7.5 Hz, 2H), 7.23 – 7.19 (m, 1H), 7.17 (d, *J* = 7.5 Hz, 2H), 5.62 (dd, *J* = 15.5, 6.7 Hz, 1H), 5.22 (ddd, *J* = 15.5, 9.3, 1.4 Hz, 1H), 2.77 – 2.72 (m, 1H), 2.65 – 2.49 (m, 2H), 2.09 – 2.00 (m, 2H), 1.80 – 1.65 (m, 6H), 1.36 – 1.26 (m, 2H), 1.23 – 1.08 (m, 3H). ^{19}F NMR (376 MHz, $CDCl_3$) δ -71.21 (d, *J* = 8.9 Hz). ^{13}C NMR (101 MHz, $CDCl_3$) δ 144.1, 141.1, 128.61, 128.58, 127.2 (q, *J* = 280.6 Hz), 126.3, 120.5 (q, *J* = 2.5 Hz), 46.9 (q, *J* = 26.3 Hz), 40.9, 32.93, 32.85, 32.5, 29.4 (q, *J* = 1.9 Hz), 26.2, 26.1. HRMS (EI): m/z calcd. for $C_{18}H_{23}F_3$ [M]: 296.1752, found: 296.1749. $[\alpha]_D^{20}$ = 0.1 (c = 1.0, $CHCl_3$), HPLC chiralcel OD-H column (0.4% isopropanol in hexanes, 0.3 mL/min, λ = 214 nm), t_R = 17.0 min (minor), 17.9 min (major), 88% *ee*.

(*E*)-(5-(trifluoromethyl)hept-3-ene-1,7-diyl)dibenzene

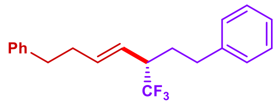

21

The product **21** was purified with silica gel chromatography (Hexane) as a colorless oil (18.5 mg, 58% yield). ^{1}H NMR (500 MHz, $CDCl_3$) δ 7.32 – 7.28 (m, 4H), 7.23 – 7.18 (m, 4H), 7.13 (d, *J* = 6.9 Hz, 2H), 5.70 (dt, *J* = 15.4, 6.8 Hz, 1H), 5.28 (ddt, *J* = 15.4, 9.2, 1.5 Hz, 1H), 2.81 – 2.71 (m, 2H), 2.69 – 2.57 (m, 2H), 2.51 – 2.40 (m, 3H), 2.06 – 1.99 (m, 1H), 1.76 – 1.68 (m, 1H). ^{19}F NMR (471 MHz, $CDCl_3$) δ -71.09 (d, *J* = 8.7 Hz). ^{13}C NMR (126 MHz, $CDCl_3$) δ 141.5, 141.1, 137.1, 128.63, 128.60, 128.56, 128.5, 127.1 (q, *J* = 280.0 Hz), 126.3, 126.1, 124.1 (q, *J* = 2.5 Hz), 46.9 (q, *J* = 26.7 Hz), 35.6, 34.3, 32.5, 29.4 (q, *J* = 2.2 Hz). HRMS (EI): m/z calcd. for $C_{20}H_{21}F_3$ [M]: 318.1595, found: 318.1590. $[\alpha]_D^{20}$ = 0.3 (c = 1.0, $CHCl_3$), HPLC chiralcel OD-H column (1% isopropanol in hexanes, 1.0 mL/min, λ = 214 nm), t_R = 8.0 min (minor), 9.6 min (major), 92% *ee*.

ethyl (*E*)-6-phenyl-4-(trifluoromethyl)hex-2-enoate

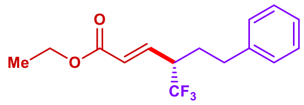

22

The product **22** was purified with silica gel chromatography (PE/EA = 30/1) as a colorless oil (11.7 mg, 41% yield). ^{1}H NMR (500 MHz, $CDCl_3$) δ 7.31 (dd, *J* = 8.1, 6.9 Hz, 2H), 7.22 (t, *J* = 7.4 Hz, 1H), 7.16 (d, *J* = 6.9 Hz, 2H), 6.75 (dd, *J* = 15.7, 9.5 Hz, 1H), 6.01 (d, *J* = 15.7 Hz, 1H), 4.24 (q, *J* = 7.1 Hz, 2H), 2.92 – 2.82 (m, 1H), 2.77 – 2.72 (m, 1H), 2.54 (dt, *J* = 13.9, 8.3 Hz, 1H), 2.19 – 2.12 (m, 1H), 1.93 – 1.86 (m, 1H), 1.33 (t, *J* = 7.1 Hz, 3H). ^{19}F NMR (376 MHz, $CDCl_3$) δ -70.02 (d, *J* = 8.2 Hz). ^{13}C NMR

(101 MHz, $CDCl_3$) δ 165.5, 140.2, 140.1 (q, J = 2.5 Hz), 128.8, 128.5, 127.5, 126.6, 126.2 (q, J = 280.6 Hz), 61.0, 46.2 (q, J = 27.1 Hz), 32.4, 29.0 (q, J = 2.3 Hz), 14.3. HRMS (ESI): m/z calcd. for $C_{15}H_{17}F_3NaO_2^+$ [M + Na^+]: 309.1073, found: 309.1083. $[\alpha]_D^{20}$ = 0.2 (c = 0.5, $CHCl_3$), HPLC chiralcel OD-H column (5% isopropanol in hexanes, 1.0 mL/min, λ = 214 nm), t_R = 6.1 min (minor), 6.7 min (major), 97% *ee*.

(*E*)-6-phenyl-1-(piperidin-1-yl)-4-(trifluoromethyl)hex-2-en-1-one

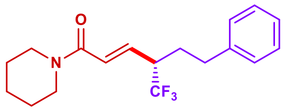

23

The product **23** was purified with silica gel chromatography (PE/EA = 30/1) as a colorless oil (17.2 mg, 53% yield). ^{1}H NMR (400 MHz, $CDCl_3$) δ 7.32 – 7.27 (m, 2H), 7.23 – 7.19 (m, 1H), 7.17 – 7.14 (m, 2H), 6.62 (dd, J = 15.2, 9.4 Hz, 1H), 6.42 (d, J = 15.2 Hz, 1H), 3.63 (t, J = 5.4 Hz, 2H), 3.47 (t, J = 5.4 Hz, 2H), 2.91 – 2.73 (m, 2H), 2.58 – 2.50 (m, 1H), 2.19 – 2.10 (m, 1H), 1.95 – 1.85 (m, 1H), 1.75 – 1.65 (m, 3H), 1.62 – 1.57 (m, 3H). ^{19}F NMR (376 MHz, $CDCl_3$) δ -70.12 (d, J = 8.3 Hz). ^{13}C NMR (151 MHz, $CDCl_3$) δ 164.1, 140.4, 136.8, 128.7, 128.5, 126.9, 126.47, 126.47 (q, J = 280.0 Hz), 47.1, 46.5 (q, J = 27.0 Hz), 43.3, 32.4, 29.0, 26.8, 25.6, 24.6. HRMS (EI): m/z calcd. for $C_{18}H_{22}F_3NO$ [M]: 325.1653, found: 325.1656. $[\alpha]_D^{20}$ = 0.4 (c = 1.0, $CHCl_3$), HPLC chiralcel OD-H column (10% isopropanol in hexanes, 1.0 mL/min, λ = 214 nm), t_R = 7.5 min (major), 8.4 min (minor), 93% *ee*.

(*E*)-(3-(trifluoromethyl)hex-4-ene-1,5-diyl)dibenzene

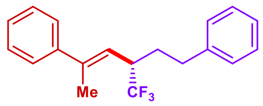

24

The product **24** was purified with silica gel chromatography (Hexane) as a colorless oil (13.7 mg, 45% yield). ^{1}H NMR (400 MHz, $CDCl_3$) δ 7.42 – 7.40 (m, 2H), 7.37 – 7.33 (m, 2H), 7.31 – 7.27 (m, 3H), 7.22 – 7.15 (m, 3H), 5.57 (dd, J = 10.0, 1.7 Hz, 1H), 3.17 – 3.04 (m, 1H), 2.82 – 2.75 (m, 1H), 2.61 – 2.54 (m, 1H), 2.23 – 2.15 (m, 1H), 1.99 (s, 1H), 1.92 – 1.83 (m, 1H). ^{19}F NMR (376 MHz, $CDCl_3$) δ -70.44 (d, J = 9.0 Hz). ^{13}C NMR (101 MHz, $CDCl_3$) δ 143.1, 141.9, 140.9, 128.7, 128.6, 128.5, 127.7, 127.4 (q, J = 280.7 Hz), 126.4, 126.1, 121.5 (q, J = 2.4 Hz), 42.6 (q, J = 26.7 Hz), 32.6, 30.2 (q, J = 1.9 Hz), 16.9. HRMS (EI): m/z calcd. for $C_{19}H_{19}F_3$ [M]: 304.1439, found: 304.1431. $[\alpha]_D^{20}$ = 1.6 (c = 1.0, $CHCl_3$), HPLC chiralcel OD-H column (1% isopropanol in hexanes, 0.5 mL/min, λ = 214 nm), t_R = 10.6 min (major), 11.3 min (minor), 93% *ee*.

(3-(trifluoromethyl)pent-1-ene-1,1,5-triyl)tribenzene

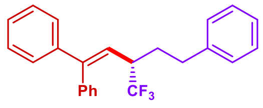

25

The product **25** was purified with silica gel chromatography (Hexane) as a colorless oil (11.7 mg, 32% yield). ^{1}H NMR (400 MHz, $CDCl_3$) δ 7.40 – 7.34 (m, 3H), 7.32 – 7.13 (m, 10H), 7.06 – 7.04 (m, 2H), 5.96 (d, J = 10.6 Hz, 1H), 3.08 – 2.96 (m, 1H), 2.75 – 2.67 (m, 1H), 2.43 – 2.35 (m, 1H), 2.09 – 2.00 (m, 1H), 1.88 – 1.79 (m, 1H). ^{19}F NMR (376 MHz, $CDCl_3$) δ -69.75 (d, J = 9.3 Hz). ^{13}C NMR (101 MHz, $CDCl_3$) δ 147.9, 141.5, 141.2, 139.1, 129.8, 128.7, 128.6, 128.4, 128.4, 128.1, 127.7, 127.5, 127.3 (q, J = 281.2 Hz), 126.2, 122.0 (q, J = 2.5 Hz), 44.0 (q, J = 26.4 Hz), 32.8, 30.8 (q, J = 2.0 Hz). HRMS (EI): m/z calcd. for $C_{24}H_{21}F_3$ [M]: 366.1595, found: 366.1596. $[\alpha]_D^{20}$ = 0.6 (c = 1.0, $CHCl_3$), HPLC chiralcel OD-H column (1% isopropanol in hexanes, 1.0 mL/min, λ = 214 nm), t_R = 4.9 min (major), 5.5 min (minor), 81% *ee*.

methyl (*E*)-4-(4,4,4-trifluoro-3-methylbut-1-en-1-yl)benzoate

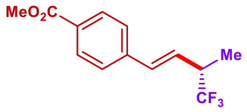

26

The product **26** was purified with silica gel chromatography (PE/EA = 30/1) as a colorless oil (24.2 mg, 94% yield). ^{1}H NMR (500 MHz, $CDCl_3$) δ 8.00 (d, J = 8.4 Hz, 2H), 7.44 (d, J = 8.4 Hz, 2H), 6.61 (d, J = 15.9 Hz, 1H), 6.23 (dd, J = 15.9, 8.0 Hz, 1H), 3.92 (s, 3H), 3.12 – 3.01 (m, 1H), 1.34 (d, J = 7.0 Hz, 3H). ^{19}F NMR (376 MHz, $CDCl_3$) δ -72.38 (d, J = 8.3 Hz). ^{13}C NMR (126 MHz, $CDCl_3$) δ 166.9, 140.8, 133.4, 130.1, 129.6, 127.1 (q, J = 280.4 Hz), 127.0 (q, J = 2.7 Hz), 126.5, 52.3, 42.1 (q, J = 27.9 Hz), 13.7 (q, J = 2.7 Hz). HRMS (EI): m/z calcd. for $C_{13}H_{13}F_3O_2$ [M]: 258.0868, found: 258.0867. $[\alpha]_D^{20}$ = 0.1 (c = 1.0, $CHCl_3$), HPLC chiralcel AD-H column (2% isopropanol in hexanes, 0.5 mL/min, λ = 254 nm), t_R = 14.9 min (minor), 15.9 min (major), 90% *ee*.

methyl (*E*)-4-(3-(trifluoromethyl)non-1-en-1-yl)benzoate

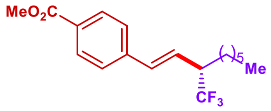

27

The product **27** was purified with silica gel chromatography (PE/EA = 30/1) as a colorless oil (21.3 mg, 65% yield). ^{1}H NMR (400 MHz, $CDCl_3$) δ 8.00 (d, J = 8.4 Hz, 2H), 7.44 (d, J = 8.4 Hz, 2H), 6.59 (d, J = 15.9 Hz, 1H), 6.08 (dd, J = 15.9, 9.4 Hz, 1H), 3.92 (s, 3H), 2.91 – 2.79 (m 1H), 1.85 – 1.77 (m, 1H), 1.61 – 1.52 (m, 1H), 1.43 – 1.23 (m, 8H), 0.87 (t, J = 6.5 Hz, 3H). ^{19}F NMR (376 MHz, $CDCl_3$) δ -70.65 (d, J = 8.7 Hz). ^{13}C NMR (101 MHz, $CDCl_3$) δ 166.9, 140.8, 134.9, 130.1, 129.6, 127.0 (q, J = 281.0 Hz), 126.5, 126.0 (q, J = 2.7 Hz), 52.3, 48.1 (q, J = 26.5 Hz), 31.7, 29.1, 28.0, 26.7, 22.7, 14.2. HRMS (ESI): m/z calcd. for $C_{18}H_{24}F_3O_2^+$ [M + H$^+$]: 329.1723, found: 329.1727. $[\alpha]_D^{20}$ = 1.1 (c = 1.0, $CHCl_3$), HPLC chiralcel OD-H column (2% isopropanol in hexanes, 0.2 mL/min, λ = 254 nm), t_R = 21.4 min (minor), 23.0 min (major), 96% *ee*.

methyl (*E*)-4-(3-(trifluoromethyl)tetradec-1-en-1-yl)benzoate

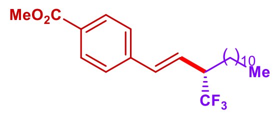

28

The product **28** was purified with silica gel chromatography (PE/EA = 30/1) as a colorless oil (23.0 mg, 58% yield). ^{1}H NMR (500 MHz, $CDCl_3$) δ 8.00 (d, J = 8.5 Hz, 2H), 7.44 (d, J = 8.5 Hz, 2H), 6.59 (d, J = 15.8 Hz, 1H), 6.08 (dd, J = 15.9, 9.3 Hz, 1H), 3.92 (s, 3H), 2.90 – 2.80 (m, 1H), 1.84 – 1.78 (m, 1H), 1.41 – 1.24 (m, 19H), 0.87 (t, J = 6.9 Hz, 3H). ^{19}F NMR (376 MHz, $CDCl_3$) δ -70.64 (d, J = 8.7 Hz). ^{13}C NMR (126 MHz, $CDCl_3$) δ 166.9, 140.8, 134.9, 130.1, 129.6, 127.0 (q, J = 280.4 Hz), 126.5, 126.1 (q, J = 2.6 Hz), 52.3, 48.1 (q, J = 26.7 Hz), 32.0, 29.7, 29.7, 29.49, 29.47, 29.4, 28.0 (d, J = 2.1 Hz), 26.7, 22.8, 14.3. HRMS (ESI): m/z calcd. for $C_{23}H_{34}F_3O_2^+$ [M + H$^+$]: 399.2505, found: 399.2506. $[\alpha]_D^{20}$ = 1.2 (c = 1.0, $CHCl_3$), HPLC chiralcel OD-H column (2% isopropanol in hexanes, 0.2 mL/min, λ = 254 nm), t_R = 20.9 min (minor), 23.0 min (major), 95% *ee*.

methyl (*E*)-4-(3-(cyclohexylmethyl)-4,4,4-trifluorobut-1-en-1-yl)benzoate

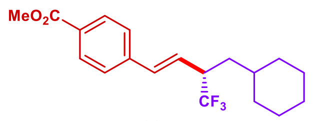

29

The product **29** was purified with silica gel chromatography (PE/EA = 30/1) as a colorless oil (23.7 mg, 70% yield). ^{1}H NMR (500 MHz, $CDCl_3$) δ 8.00 (d, J = 8.4 Hz, 2H), 7.45 (d, J = 8.4 Hz, 2H), 6.59 (d, J = 15.9 Hz, 1H), 6.06 (dd, J = 15.9, 9.3 Hz, 1H), 3.92 (s, 3H), 3.04 – 2.94 (m, 1H), 1.78 – 1.62 (m, 5H), 1.61 – 1.57 (m, 1H), 1.55 – 1.49 (m, 1H), 1.34 – 1.25 (m, 1H), 1.23 – 1.10 (m, 3H), 1.04 – 0.96 (m, 1H), 0.89 – 0.81 (m, 1H). ^{19}F NMR (471 MHz, $CDCl_3$) δ -70.71 (d, J = 9.0 Hz). ^{13}C NMR (126 MHz, $CDCl_3$)

δ 166.9, 140.8, 134.7, 130.1, 129.5, 127.1 (q, J = 280.4 Hz), 126.5, 126.2 (q, J = 2.8 Hz), 52.3, 45.3 (q, J = 26.7 Hz), 35.3, 34.3, 34.1, 31.9, 26.5, 26.2, 26.0. HRMS (ESI): m/z calcd. for $C_{19}H_{24}F_3O_2^+$ [M + H$^+$]: 341.1723, found: 341.1731. $[\alpha]_D^{20}$ = 0.7 (c = 1.0, $CHCl_3$), HPLC chiralcel OD-H column (1% isopropanol in hexanes, 0.3 mL/min, λ = 254 nm), t_R = 16.1 min (minor), 17.0 min (major), 94% *ee*.

methyl (*E*)-4-(7-chloro-3-(trifluoromethyl)hept-1-en-1-yl)benzoate

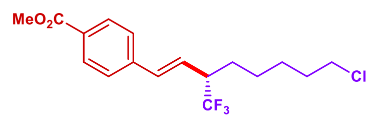

30

The product **30** was purified with silica gel chromatography (PE/EA = 30/1) as a colorless oil (28.1 mg, 81% yield). ^{1}H NMR (500 MHz, $CDCl_3$) δ 8.00 (d, J = 8.4 Hz, 2H), 7.45 (d, J = 8.4 Hz, 2H), 6.60 (d, J = 15.9 Hz, 1H), 6.08 (dd, J = 15.9, 9.4 Hz, 1H), 3.92 (s, 3H), 3.52 (t, J = 6.6 Hz, 2H), 2.91 – 2.81 (m, 1H), 1.87 – 1.74 (m, 3H), 1.63 – 1.55 (m, 1H), 1.54 – 1.39 (m, 3H), 1.36 – 1.28 (m, 1H). ^{19}F NMR (471 MHz, $CDCl_3$) δ -70.63 (d, J = 8.8 Hz). ^{13}C NMR (126 MHz, $CDCl_3$) δ 166.9, 140.6, 135.1, 130.1, 129.6, 126.9 (q, J = 280.2 Hz), 126.5, 125.6 (q, J = 2.7 Hz), 52.3, 48.0 (q, J = 26.6 Hz), 45.0, 32.4, 27.8, 26.6, 26.0. HRMS (EI): m/z calcd. for $C_{17}H_{20}ClF_3O_2$ [M]: 348.1104, found: 348.1098. $[\alpha]_D^{20}$ = 0.7 (c = 1.0, $CHCl_3$), HPLC chiralcel OD-H column (2% isopropanol in hexanes, 0.5 mL/min, λ = 254 nm), t_R = 14.3 min (minor), 20.9 min (major), 96% *ee*.

methyl (*E*)-4-(5-(4-chlorophenyl)-3-(trifluoromethyl)pent-1-en-1-yl)benzoate

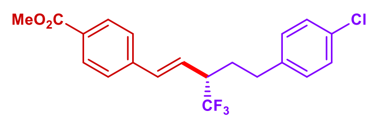

31

The product **31** was purified with silica gel chromatography (PE/EA = 30/1) as a colorless oil (27.2 mg, 71% yield). ^{1}H NMR (400 MHz, $CDCl_3$) δ 8.02 (d, J = 8.4 Hz, 2H), 7.45 (d, J = 8.4 Hz, 2H), 7.27 (d, J = 8.4 Hz, 2H), 7.10 (d, J = 8.4 Hz, 2H), 6.59 (d, J = 15.9 Hz, 1H), 6.11 (dd, J = 15.9, 9.4 Hz, 1H), 3.93 (s, 3H), 2.91 – 2.82 (m, 1H), 2.79 – 2.72 (m, 1H), 2.61 – 2.53 (m, 1H), 2.20 – 2.11 (m, 1H), 1.94 – 1.85 (m, 1H) ^{19}F NMR (376 MHz, $CDCl_3$) δ -70.49 (d, J = 8.5 Hz). ^{13}C NMR (101 MHz, $CDCl_3$) δ 166.9, 140.5, 139.0, 135.7, 132.3, 130.2, 129.9, 129.8, 128.9, 126.8 (q, J = 280.6 Hz), 126.6, 125.2 (d, J = 2.6 Hz), 52.3, 47.3 (q, J = 26.8 Hz), 32.0, 29.3. HRMS (ESI): m/z calcd. for $C_{20}H_{19}ClF_3O_2^+$ [M + H$^+$]: 383.1020, found: 383.1030. $[\alpha]_D^{20}$ = 1.0 (c = 1.0, $CHCl_3$), HPLC chiralcel AS-H column (4% isopropanol in hexanes, 1.0 mL/min, λ = 254 nm), t_R = 8.2 min (major), 21.5 min (minor), 94% *ee*.

methyl (*E*)-4-(5-(4-methoxyphenyl)-3-(trifluoromethyl)pent-1-en-1-yl)benzoate

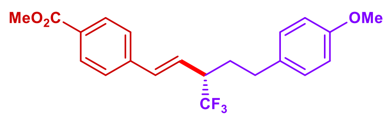

32

The product **32** was purified with silica gel chromatography (PE/EA = 10/1) as a colorless oil (32.1 mg, 85% yield). ^{1}H NMR (400 MHz, $CDCl_3$) δ 8.02 (d, *J* = 8.3 Hz, 2H), 7.46 (d, *J* = 8.3 Hz, 2H), 7.09 (d, *J* = 8.6 Hz, 2H), 6.84 (d, *J* = 8.6 Hz, 2H), 6.59 (d, *J* = 15.9 Hz, 1H), 6.12 (dd, *J* = 15.9, 9.4 Hz, 1H), 3.93 (s, 3H), 3.79 (s, 3H), 2.93 – 2.81 (m, 1H), 2.77 – 2.70 (m, 1H), 2.58 – 2.50 (m, 1H), 2.19 – 2.11 (m, 1H), 1.93 – 1.84 (m, 1H). ^{19}F NMR (376 MHz, $CDCl_3$) δ -70.46 (d, *J* = 8.8 Hz). ^{13}C NMR (101 MHz, $CDCl_3$) δ 166.9, 158.2, 140.7, 135.5, 132.6, 130.1, 129.7, 129.5, 126.9 (q, *J* = 280.8 Hz),126.5, 125.5 (q, *J* = 2.5 Hz), 114.1, 55.4, 52.3, 47.2 (q, *J* = 26.8 Hz), 31.6, 29.6. HRMS (ESI): m/z calcd. for $C_{21}H_{22}F_3O_3^+$ [M + H$^+$]: 379.1516, found: 379.1517. $[\alpha]_D^{20}$ = 1.4 (c = 1.0, $CHCl_3$), HPLC chiralcel OD-H column (5% isopropanol in hexanes, 0.5 mL/min, λ = 254 nm), t_R = 23.8 min (major), 26.4 min (minor), 94% *ee*.

methyl (*E*)-4-(6-(benzoyloxy)-3-(trifluoromethyl)hex-1-en-1-yl)benzoate

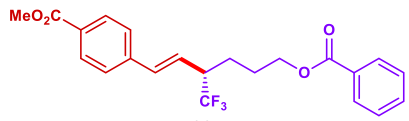

33

The product **33** was purified with silica gel chromatography (PE/EA = 10/1) as a colorless oil (29.2 mg, 72% yield). ^{1}H NMR (500 MHz, $CDCl_3$) δ 8.04 – 7.99 (m, 4H), 7.56 (t, *J* = 7.5 Hz, 1H), 7.46 – 7.43 (m, 4H), 6.64 (d, *J* = 15.9 Hz, 1H), 6.11 (dd, *J* = 15.9, 9.3 Hz, 1H), 4.35 (t, *J* = 6.2 Hz, 2H), 3.92 (s, 3H), 3.00 – 2.91 (m, 1H), 2.06 – 1.99 (m, 1H), 1.95 – 1.87 (m, 1H), 1.84 – 1.70 (m, 2H). ^{19}F NMR (376 MHz, $CDCl_3$) δ -70.61 (d, *J* = 8.2 Hz). ^{13}C NMR (126 MHz, $CDCl_3$) δ 166.8, 166.6, 140.5, 135.6, 133.2, 130.3, 130.1, 129.8, 129.7, 128.6, 126.8 (q, *J* = 280.1 Hz), 126.6, 125.1 (d, *J* = 2.8 Hz), 64.2, 52.2, 47.8 (q, *J* = 27.1 Hz), 26.1, 24.8. HRMS (ESI): m/z calcd. for $C_{22}H_{21}F_3NaO_4^+$ [M + Na$^+$]: 429.1284, found: 429.1283. $[\alpha]_D^{20}$ = 0.4 (c = 1.0, $CHCl_3$), HPLC chiralcel AD-H column (10% isopropanol in hexanes, 1.0 mL/min, λ = 254 nm), t_R = 13.0 min (minor), 15.0 min (major), 94% *ee*.

tert-butyl (*E*)-4-(4-(4-(methoxycarbonyl)phenyl)-2-(trifluoromethyl)but-3-en-1-yl)piperidine-1-carboxylate

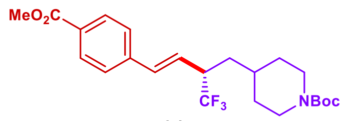

34

The product **34** was purified with silica gel chromatography (PE/EA = 10/1) as a colorless oil (35.6 mg, 81% yield). ^{1}H NMR (400 MHz, $CDCl_3$) δ 8.01 (d, *J* = 8.4 Hz, 2H), 7.45 (d, *J* = 8.4 Hz, 2H), 6.61 (d, *J* = 15.9 Hz, 1H), 6.07 (dd, *J* = 15.9, 9.3 Hz, 1H), 4.13 – 4.07 (m, 2H), 3.92 (s, 3H), 3.05 – 2.93 (m, 1H), 2.70 – 2.58 (m, 2H), 1.71 – 1.56 (m, 5H), 1.44 (s, 9H), 1.28 – 1.02 (m, 2H). ^{19}F NMR (376 MHz, $CDCl_3$) δ -70.72 (d, *J* = 8.7 Hz). ^{13}C NMR (101 MHz, $CDCl_3$) δ 166.8, 154.9, 140.5, 135.1, 130.2, 129.8, 126.9 (q, *J* = 280.9 Hz), 126.6, 125.6 (q, *J* = 2.5 Hz)., 79.6, 52.3, 45.2 (q, *J* = 26.8 Hz), 34.6, 33.0, 32.8, 31.0, 28.6. HRMS (ESI): m/z calcd. for $C_{23}H_{30}F_3NNaO_4^+$ [M + Na$^+$]: 464.2019, found: 464.2025. $[\alpha]_D^{20}$ = 1.7 (c = 1.0, $CHCl_3$), HPLC chiralcel AD-H column (10% isopropanol in hexanes, 1.0 mL/min, λ = 254 nm), t_R = 16.9 min (major), 18.9 min (minor), 96% *ee*.

(*E*)-6-(4-(methoxycarbonyl)phenyl)-4-(trifluoromethyl)hex-5-en-1-yl furan-2-carboxylate

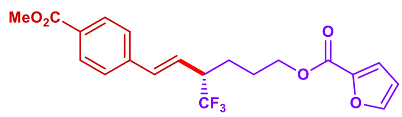

35

The product **35** was purified with silica gel chromatography (PE/EA = 10/1) as a colorless oil (32.8 mg, 83% yield). ^{1}H NMR (400 MHz, $CDCl_3$) δ 7.99 (d, *J* = 8.4 Hz, 2H), 7.58 (dd, *J* = 1.8, 0.9 Hz, 1H), 7.43 (d, *J* = 8.4 Hz, 2H), 7.18 (dd, *J* = 3.5, 0.9 Hz, 1H), 6.63 (d, *J* = 15.9 Hz, 1H), 6.51 (dd, *J* = 3.5, 1.7 Hz, 1H), 6.09 (dd, *J* = 15.9, 9.3 Hz, 1H), 4.33 (t, *J* = 6.2 Hz, 2H), 3.91 (s, 3H), 3.01 – 2.89 (m, 1H), 2.12 – 1.65 (m, 4H). ^{19}F NMR (376 MHz, $CDCl_3$) δ -70.64 (d, *J* = 8.9 Hz). ^{13}C NMR (151 MHz, $CDCl_3$) δ 166.9, 158.8, 146.6, 144.6, 140.4, 135.6, 130.1, 129.7, 126.7 (q, *J* = 280.5 Hz), 126.6, 125.0, 118.2, 112.0, 64.3, 52.3, 47.8 (q, *J* = 27.1 Hz), 26.0, 24.6. HRMS (ESI): m/z calcd. for $C_{20}H_{19}F_3NaO_5^+$ [M + Na$^+$]: 419.1077, found: 419.1083. $[\alpha]_D^{20}$ = 0.8 (c = 1.0, $CHCl_3$), HPLC chiralcel AD-H column (10% isopropanol in hexanes, 1.0 mL/min, λ = 254 nm), t_R = 16.1 min (minor), 19.4 min (major), 95% *ee*.

methyl (*E*)-4-(6-(1,3-dioxoisoindolin-2-yl)-3-(trifluoromethyl)hex-1-en-1-yl)benzoate

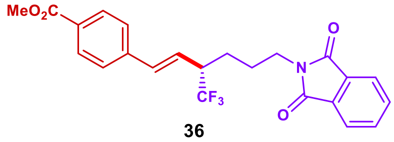

36

The product **36** was purified with silica gel chromatography (PE/EA = 10/1) as a colorless oil (34.0 mg,

79% yield). ^{1}H NMR (500 MHz, $CDCl_3$) δ 7.97 (d, *J* = 6.7 Hz, 2H), 7.91 – 7.76 (m, 2H), 7.71 – 7.69 (m, 2H), 7.42 (d, *J* = 6.6 Hz, 2H), 6.61 (d, *J* = 15.9 Hz, 1H), 6.06 (dd, *J* = 15.8, 9.4 Hz, 1H), 3.90 (s, 3H), 3.71 (t, *J* = 6.2 Hz, 2H), 2.99 – 2.90 (m, 1H), 1.89 – 1.60 (m, 4H). ^{19}F NMR (471 MHz, $CDCl_3$) δ -70.60 (d, *J* = 8.7 Hz). ^{13}C NMR (126 MHz, $CDCl_3$) δ 168.4, 166.8, 140.4, 135.6, 134.2, 132.1, 130.1, 129.6, 126.7 (q, *J* = 280.2 Hz), 126.6, 125.0 (q, *J* = 2.8 Hz), 123.4, 52.2, 47.7 (q, *J* = 26.9 Hz), 37.4, 25.8, 25.2. HRMS (ESI): m/z calcd. for $C_{23}H_{21}F_3NO_4^+$ [M + H$^+$]: 432.1417, found: 432.1432. $[\alpha]_D^{20}$ = 3.2 (c = 2.0, $CHCl_3$), HPLC chiralcel OD-H column (10% isopropanol in hexanes, 1.0 mL/min, λ = 254 nm), t_R = 15.5 min (minor), 24.8 min (major), 92% *ee*.

(S,*E*)-6-(4-(methoxycarbonyl)phenyl)-4-(trifluoromethyl)hex-5-en-1-yl ferrocene carboxylate

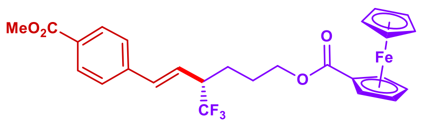

37

The product **37** was purified with silica gel chromatography (PE/EA = 10/1) as an orange solid (40.6 mg, 79% yield); mp: 80-81 °C. ^{1}H NMR (400 MHz, $CDCl_3$) δ 8.00 (d, *J* = 8.4 Hz, 2H), 7.45 (d, *J* = 8.4 Hz, 2H), 6.65 (d, *J* = 15.8 Hz, 1H), 6.12 (dd, *J* = 15.9, 9.4 Hz, 1H), 4.85 – 4.77 (m, 2H), 4.40 (t, *J* = 1.9 Hz, 2H), 4.29 – 4.15 (m, 7H), 3.92 (s, 3H), 3.00 – 2.93 (m, 1H), 2.05 – 1.99 (m, 1H), 1.89 – 1.83 (m, 1H), 1.79 – 1.68 (m, 2H). ^{19}F NMR (376 MHz, $CDCl_3$) δ -70.59 (d, *J* = 8.5 Hz). ^{13}C NMR (151 MHz, $CDCl_3$) δ 171.9, 166.9, 140.4, 135.6, 130.1, 129.7, 126.8 (q, *J* = 280.2 Hz), 126.6, 125.1, 71.5, 71.1, 70.23, 70.21, 69.9, 63.4, 52.3, 47.8 (q, *J* = 27.0 Hz), 26.2, 24.8. HRMS (ESI): m/z calcd. for $C_{26}H_{26}F_3FeO_4^+$ [M + H$^+$]: 515.1127, found: 515.1121. $[\alpha]_D^{20}$ = 1.2 (c = 1.0, $CHCl_3$), HPLC chiralcel AD-H column (5% isopropanol in hexanes, 0.5 mL/min, λ = 254 nm), t_R = 52.8 min (major), 56.8 min (minor), 94% *ee*.

methyl (*E*)-4-(4,4,5,5,5-pentafluoro-3-phenethylpent-1-en-1-yl)benzoate

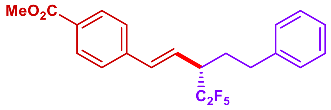

38

The product **38** was purified with silica gel chromatography (PE/EA = 30/1) as a colorless oil (34.3 mg, 86% yield). ^{1}H NMR (400 MHz, $CDCl_3$) δ 8.03 (d, *J* = 8.4 Hz, 2H), 7.46 (d, *J* = 8.4 Hz, 2H), 7.31 (dd, *J* = 8.0, 6.7 Hz, 2H), 7.25 – 7.14 (m, 3H), 6.56 (d, *J* = 15.8 Hz, 1H), 6.09 (dd, *J* = 15.9, 9.8 Hz, 1H), 3.93 (s, 3H), 3.97 – 2.77 (m, 2H), 2.60 – 2.53 (m, 1H), 2.60 – 2.21 (m, 1H), 1.96 – 1.87 (m, 1H). ^{19}F NMR (376 MHz, $CDCl_3$) δ -81.47 (s), -116.30 (dd, *J* = 269.6, 9.4 Hz), -122.11 (dd, *J* = 269.9, 20.4 Hz).^{13}C NMR (126 MHz, $CDCl_3$) δ 166.9, 140.6, 140.5, 135.4, 130.2, 129.8, 128.7, 128.5, 126.6, 126.5, 125.1

(dd, *J* = 6.3, 2.7 Hz), 119.4 (qt, *J* = 287.3, 36.7 Hz), 115.8 (tq, *J* = 256.8, 35.7 Hz), 52.3, 45.6 (t, *J* = 21.5 Hz), 32.6, 28.1. HRMS (ESI): m/z calcd. for $C_{21}H_{20}F_5O_2^+$ [M + H$^+$]: 399.1378, found: 399.1384. $[\alpha]_D^{20}$ = 0.7 (c = 1.0, $CHCl_3$), HPLC chiralcel OD-H column (2% isopropanol in hexanes, 0.5 mL/min, λ = 254 nm), t_R = 29.6 min (minor), 31.0 min (major), 96% *ee*.

(*E*)-7-phenyl-5-(trifluoromethyl)hept-3-en-1-yl 2-(2-fluoro-[1,1'-biphenyl]-4-yl)propanoate

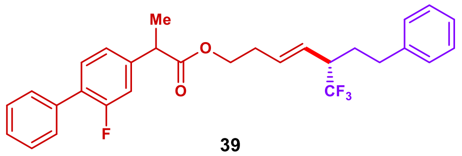

39

The product **39** was purified with silica gel chromatography (PE/EA = 20/1) as a colorless oil (29.0 mg, 60% yield). ^{1}H NMR (500 MHz, $CDCl_3$) δ 7.54 – 7.51 (m, 2H), 7.44 (t, *J* = 7.5 Hz, 2H), 7.40 – 7.36 (m, 2H), 7.29 (t, *J* = 7.5 Hz, 2H), 7.21 (t, *J* = 7.4 Hz, 1H), 7.15 – 7.11 (m, 4H), 5.61 (dt, *J* = 14.4, 6.8 Hz, 1H), 5.36 (dt, *J* = 15.6, 8.4 Hz, 1H), 4.25 – 4.13 (m, 2H), 3.75 (q, *J* = 7.1 Hz, 1H), 2.74 – 2.60 (m, 2H), 2.52 – 2.41 (m, 3H), 2.08 – 2.01 (m, 1H), 1.79 – 1.69 (m, 1H), 1.54 (dd, *J* = 7.2, 3.3 Hz, 3H). ^{19}F NMR (471 MHz, $CDCl_3$) δ -70.99 (d, *J* = 8.7 Hz), -117.29 – -117.85 (m). ^{13}C NMR (126 MHz, $CDCl_3$) δ 174.1, 159.8 (d, *J* = 248.3 Hz), 141.9 (d, *J* = 7.5 Hz), 140.8, 135.6, 133.1, 130.9 (d, *J* = 4.2 Hz), 129.1 (d, *J* = 3.2 Hz), 128.7, 128.6, 128.5, 128.0 (d, *J* = 13.5 Hz), 127.8, 127.0 (q, *J* = 280.1 Hz), 126.4, 126.2, 123.7, 115.4 (d, *J* = 23.8 Hz), 63.9 (d, *J* = 3.8 Hz), 46.9 (q, *J* = 26.7 Hz), 45.2, 32.5, 32.0, 29.2, 18.5. HRMS (ESI): m/z calcd. for $C_{29}H_{28}F_4NaO_2^+$ [M + Na$^+$]: 507.1918, found: 507.1922. $[\alpha]_D^{20}$ = 0.3 (c = 1.0, $CHCl_3$), HPLC chiralcel AS-H column contacted with another AS-H column (1.5% isopropanol in hexanes, 0.3 mL/min, λ = 254 nm), t_R = 36.2 min (major), 38.2 min (minor), 42.6 min (major), 51.1 min (minor), 93% *ee*, dr = 1:1.

(*E*)-7-phenyl-5-(trifluoromethyl)hept-3-en-1-yl 2-(1-(4-chlorobenzoyl)-5-methoxy-2-methyl-1*H*-indol-3-yl)acetate

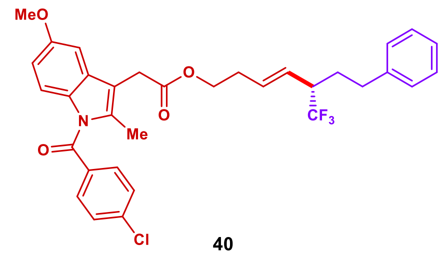

40

The product **40** was purified with silica gel chromatography (PE/EA = 3/1) as a yellow oil (39.5 mg, 66% yield). ^{1}H NMR (500 MHz, $CDCl_3$) δ 7.65 (d, *J* = 8.5 Hz, 2H), 7.46 (d, *J* = 8.4 Hz, 2H), 7.29 (t, *J* = 7.7 Hz, 2H), 7.22 – 7.19 (m, 1H), 7.15 (d, *J* = 7.5 Hz, 2H), 6.97 (s, 1H), 6.87 (d, *J* = 9.0 Hz, 1H), 6.68 (d, *J* = 9.2 Hz, 1H), 5.61 (dt, *J* = 14.4, 6.9 Hz, 1H), 5.37 (dd, *J* = 15.5, 9.1 Hz, 1H), 4.23 – 4.15 (m, 2H), 3.83

(s, 3H), 3.67 (s, 2H), 2.73 – 2.59 (m, 2H), 2.51 – 2.43 (m, 3H), 2.38 (s, 3H), 2.08 – 2.01 (m, 1H), 1.78 – 1.70 (m, 1H). ^{19}F NMR (471 MHz, $CDCl_3$) δ -70.96 (d, J = 8.8 Hz). ^{13}C NMR (126 MHz, $CDCl_3$) δ 170.9, 168.4, 156.2, 140.8, 139.4, 136.1, 134.0, 133.0, 131.3, 130.9, 130.8, 129.2, 128.7, 128.5, 127.0 (q, J = 280.1 Hz), 126.4, 126.3 (q, J = 2.7 Hz), 115.1, 112.6, 111.6, 101.5, 64.0, 55.8, 46.9 (q, J = 26.5 Hz), 32.4, 32.1, 30.4, 29.1, 13.4. HRMS (ESI): m/z calcd. for $C_{33}H_{32}ClF_3NO_4^+$ [M + H$^+$]: 598.1966, found: 598.1976. $[\alpha]_D^{20}$ = 0.3 (c = 1.0, $CHCl_3$), HPLC chiralcel OD-H column (5% isopropanol in hexanes, 1.0 mL/min, λ = 254 nm), t_R = 35.9 min (major), 44.0 min (minor), 86% *ee*.

(*E*)-7-phenyl-5-(trifluoromethyl)hept-3-en-1-yl 2-(4-(4-chlorobenzoyl)phenoxy)-2-methylpropanoate

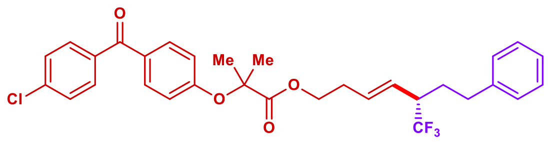

41

The product **41** was purified with silica gel chromatography (PE/EA = 10/1) as a colorless oil (31.3 mg, 56% yield). ^{1}H NMR (500 MHz, $CDCl_3$) δ 7.71 (dd, J = 19.3, 6.7 Hz, 4H), 7.44 (d, J = 6.5 Hz, 2H), 7.29 (t, J = 7.1 Hz, 2H), 7.22 – 7.19 (m, 1H), 7.14 (d, J = 7.4 Hz, 2H), 6.85 (d, J = 6.7 Hz, 2H), 5.55 (dt, J = 14.6, 6.7 Hz, 1H), 5.33 (dd, J = 15.5, 9.1 Hz, 1H), 4.29 – 4.20 (m, 2H), 2.73 – 2.57 (m, 2H), 2.51 – 2.39 (m, 3H), 2.07 – 2.00 (m, 1H), 1.75 – 1.67 (m, 7H). ^{19}F NMR (471 MHz, $CDCl_3$) δ -70.95 (d, J = 8.7 Hz). ^{13}C NMR (126 MHz, $CDCl_3$) δ 194.3, 173.8, 159.7, 140.8, 138.5, 136.5, 132.7, 132.2, 131.3, 130.5, 128.7, 128.5, 126.9 (q, J = 280.1 Hz), 126.43 (q, J = 2.6 Hz), 126.37, 117.4, 79.5, 64.6, 46.9 (q, J = 26.7 Hz), 32.5, 31.9, 29.2, 25.5. HRMS (ESI): m/z calcd. for $C_{31}H_{31}ClF_3O_4^+$ [M + H$^+$]: 559.1857, found: 559.1859. $[\alpha]_D^{20}$ = 0.3 (c = 1.0, $CHCl_3$), HPLC chiralcel AD-H column (2% isopropanol in hexanes, 0.5 mL/min, λ = 254 nm), t_R = 43.1 min (major), 45.6 min (minor), 94% *ee*.

Methyl 4-((*E*)-6-(((tert-butoxycarbonyl)-L-phenylalanyl)oxy)-3-(trifluoromethyl)hex-1-en-1-yl)benzoate

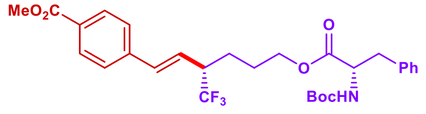

42

The product **42** was purified with silica gel chromatography (PE/EA = 5/1) as a colorless oil (30.1 mg, 55% yield). ^{1}H NMR (400 MHz, $CDCl_3$) δ 8.01 (d, J = 8.3 Hz, 2H), 7.45 (d, J = 8.4 Hz, 2H), 7.28 – 7.18 (m, 3H), 7.13 – 7.11 (m, 2H), 6.60 (d, J = 15.9 Hz, 1H), 6.05 (dd, J = 15.9, 9.3 Hz, 1H), 4.97 – 4.95 (m, 1H), 4.58 – 4.53 (m, 1H), 4.15 – 4.02 (m, 2H), 3.92 (s, 3H), 3.05 (d, J = 6.4 Hz, 2H), 2.88 – 2.81 (m,

1H), 1.83 – 1.76 (m, 1H), 1.73 – 1.61 (m, 1H), 1.58 – 1.48 (m, 2H), 1.41 (s, 9H). ^{19}F NMR (376 MHz, $CDCl_3$) δ -70.58 (d, *J* = 8.7 Hz). ^{13}C NMR (101 MHz, $CDCl_3$) δ 172.2, 166.9, 155.2, 140.4, 136.1, 135.6, 130.2, 129.8, 129.4, 128.7, 127.2, 126.7 (q, *J* = 281.1 Hz), 126.6, 125.1, 80.2, 64.6, 54.7, 52.3, 47.8 (q, *J* = 27.2 Hz), 38.7, 28.4, 25.8, 24.6. HRMS (ESI): m/z calcd. for $C_{29}H_{35}F_3NO_6^+$ [M + H$^+$]: 550.2411, found: 550.2416. $[\alpha]_D^{20}$ = 0.2 (c = 1.0, $CHCl_3$), HPLC chiralcel AS-H column (2% isopropanol in hexanes, 1.0 mL/min, λ = 254 nm), t_R = 85.1 min (major), 115.8 min (minor), 94% *de*.

methyl 4-((*E*)-6-(((S)-2-(4-isobutylphenyl)propanoyl)oxy)-3-(trifluoromethyl)hex-1-en-1-yl)benzoate

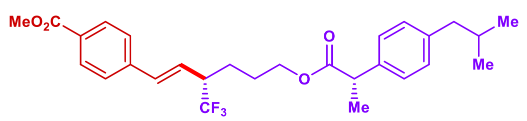

43

The product **43** was purified with silica gel chromatography (PE/EA = 10/1) as a colorless oil (35.7 mg, 73% yield). ^{1}H NMR (500 MHz, $CDCl_3$) δ 8.00 (d, *J* = 8.4 Hz, 2H), 7.41 (d, *J* = 8.5 Hz, 2H), 7.19 (d, *J* = 8.1 Hz, 2H), 7.08 (d, *J* = 8.1 Hz, 2H), 6.54 (d, *J* = 15.9 Hz, 1H), 5.99 (dd, *J* = 15.9, 9.4 Hz, 1H), 4.14 – 4.03 (m, 2H), 3.92 (s, 3H), 3.69 (q, *J* = 7.2 Hz, 1H), 2.86 – 2.75 (m, 1H), 2.42 (d, *J* = 7.2 Hz, 2H), 1.86 – 1.76 (m, 2H), 1.73 – 1.65 (m, 1H), 1.60 – 1.47 (m, 5H), 0.88 (d, *J* = 6.6 Hz, 6H). ^{19}F NMR (471 MHz, $CDCl_3$) δ -70.69 (d, *J* = 8.6 Hz). ^{13}C NMR (126 MHz, $CDCl_3$) δ 174.9, 166.9, 140.8, 140.5, 137.8, 135.4, 130.1, 129.7, 129.5, 127.2, 126.7 (q, *J* = 280.7 Hz), 126.5, 125.2 (q, *J* = 2.8 Hz), 63.8, 52.3, 47.6 (q, *J* = 27.0 Hz), 45.3, 45.1, 30.3, 25.8, 24.4, 22.5, 18.5. HRMS (ESI): m/z calcd. for $C_{28}H_{33}F_3NaO_4^+$ [M + Na$^+$]: 513.2223, found: 513.2226. $[\alpha]_D^{20}$ = 0.6 (c = 1.0, $CHCl_3$), HPLC chiralcel AD-H column (10% isopropanol in hexanes, 1.0 mL/min, λ = 254 nm), t_R = 7.1 min (major), 7.9 min (minor), 96% *de*.

methyl 4-((*E*)-6-(((S)-2-(6-methoxynaphthalen-2-yl)propanoyl)oxy)-3-(trifluoromethyl)hex-1-en-1-yl)benzoate

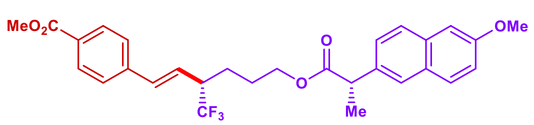

44

The product **44** was purified with silica gel chromatography (PE/EA = 10/1) as a colorless oil (34.8 mg, 68% yield). ^{1}H NMR (400 MHz, $CDCl_3$) δ 7.97 (d, *J* = 8.4 Hz, 2H), 7.68 (t, *J* = 8.3 Hz, 3H), 7.39 (dd, *J* = 8.4, 1.8 Hz, 1H), 7.31 (d, *J* = 8.1 Hz, 2H), 7.19 – 7.02 (m, 2H), 6.45 (d, *J* = 15.9 Hz, 1H), 5.90 (dd, *J* = 15.8, 9.3 Hz, 1H), 4.17 – 4.05 (m, 2H), 3.92 – 3.82 (m, 7H), 2.83 – 2.72 (m, 1H), 1.81 – 1.75 (m, 1H), 1.72 – 1.65 (m, 1H), 1.60 – 1.44 (m, 5H). ^{19}F NMR (376 MHz, $CDCl_3$) δ -70.66 (d, *J* = 8.8 Hz). ^{13}C

NMR (151 MHz, $CDCl_3$) δ 174.8, 166.9, 157.8, 140.4, 135.7, 135.4, 133.8, 130.1, 129.6, 129.4, 129.0, 127.3, 126.6 (q, J = 280.3 Hz), 126.5, 126.3, 126.1, 125.1, 119.2, 105.6, 64.0, 55.4, 52.3, 47.6 (q, J = 27.1 Hz), 45.6, 25.8, 24.5, 18.5. HRMS (ESI): m/z calcd. for $C_{29}H_{30}F_3O_5^+$ [M + H$^+$]: 515.2040, found: 515.2047. $[\alpha]_D^{20}$ = 1.4 (c = 1.0, $CHCl_3$), HPLC chiralcel AD-H column (10% isopropanol in hexanes, 1.0 mL/min, λ = 254 nm), t_R = 19.6 min (major), 22.2 min (minor), 94% *de*.

methyl (*E*)-4-(6-((5-(2,5-dimethylphenoxy)-2,2-dimethylpentanoyl)oxy)-3-(trifluoromethyl)hex-1-en-1-yl)benzoate

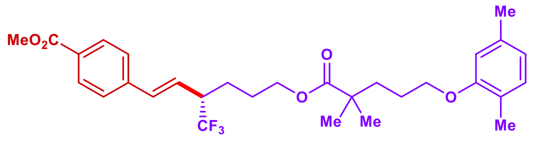

45

The product **45** was purified with silica gel chromatography (PE/EA = 10/1) as a colorless oil (37.4 mg, 70% yield). ^{1}H NMR (500 MHz, $CDCl_3$) δ 7.99 (d, J = 8.5 Hz, 2H), 7.43 (d, J = 8.4 Hz, 2H), 6.99 (d, J = 7.4 Hz, 1H), 6.79 – 6.52 (m, 3H), 6.08 (dd, J = 15.9, 9.3 Hz, 1H), 4.09 (t, J = 6.1 Hz, 2H), 3.92 (s, 3H), 3.91 – 3.89 (m, 2H), 2.93 – 2.86 (m, 1H), 2.30 (s, 3H), 2.16 (s, 3H), 1.96 – 1.89 (m, 1H), 1.82– 1.72 (m, 5H), 1.67– 1.62 (m, 2H), 1.22 (s, 6H). ^{19}F NMR (376 MHz, $CDCl_3$) δ -70.68 (d, J = 9.2 Hz). ^{13}C NMR (126 MHz, $CDCl_3$) δ 177.9, 166.8, 157.0, 140.4, 136.6, 135.5, 130.4, 130.1, 129.8, 126.7 (q, J = 280.0 Hz), 126.6, 125.1, 123.7, 120.9, 112.1, 68.0, 63.6, 52.3, 47.7 (q, J= 26.7 Hz), 42.3, 37.2, 25.9, 25.33, 25.30, 24.6, 21.5, 15.9. HRMS (ESI): m/z calcd. for $C_{30}H_{37}F_3NaO_5^+$ [M + Na$^+$]: 557.2485, found: 557.2491. $[\alpha]_D^{20}$ = 1.0 (c = 1.0, $CHCl_3$), HPLC chiralcel AD-H column (3% isopropanol in hexanes, 1.0 mL/min, λ = 254 nm), t_R = 12.9 min (major), 14.0 min (minor), 95% *ee*.

methyl (*E*)-4-(6-(2-(11-oxo-6,11-dihydrodibenzo[b,e]oxepin-8-yl)acetoxy)-3-(trifluoromethyl)hex-1-en-1-yl)benzoate

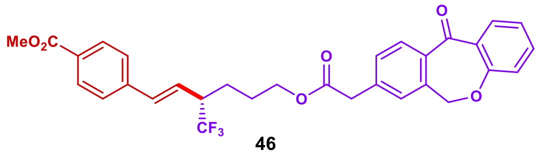

46

The product **46** was purified with silica gel chromatography (PE/EA = 5/1) as a colorless oil (47.3 mg, 86% yield). ^{1}H NMR (400 MHz, $CDCl_3$) δ 8.12 (d, J = 2.4 Hz, 1H), 7.98 (d, J = 8.3 Hz, 2H), 7.87 (dd, J = 7.7, 1.4 Hz, 1H), 7.57 – 7.53 (m, 1H), 7.48 – 7.39 (m, 4H), 7.35 (d, J = 7.4 Hz, 1H), 6.58 (d, J = 15.9 Hz, 1H), 6.05 (dd, J = 15.9, 9.3 Hz, 1H), 5.15 (s, 2H), 4.13 (t, J = 6.0 Hz, 2H), 3.91 (s, 3H), 3.64 (s, 2H), 2.90 – 2.81 (m, 1H), 1.93 – 1.55 (m, 5H). ^{19}F NMR (376 MHz, $CDCl_3$) δ -70.59 (d, J = 8.3 Hz). ^{13}C NMR (151 MHz, $CDCl_3$) δ 190.9, 171.5, 166.8, 160.6, 140.5, 140.4, 136.4, 135.6, 135.5, 132.9, 132.5,

130.1, 129.7 (q, J = 280.2 Hz), 129.6, 129.4, 127.9, 127.8, 126.7, 126.6, 125.2, 125.1, 121.2, 73.7, 64.3, 52.3, 47.7 (q, J = 26.7 Hz), 40.4, 25.8, 24.6. HRMS (ESI): m/z calcd. for $C_{31}H_{27}F_3NaO_6^+$ [M + Na$^+$]: 575.1652, found: 575.1658. $[\alpha]_D^{20}$ = 1.2 (c = 1.0, $CHCl_3$), HPLC chiralcel AD-H column (40% isopropanol in hexanes, 0.5 mL/min, λ = 254 nm), t_R = 30.7 min (minor), 34.2 min (major), 96% *ee*.

(*E*)-6-(4-(methoxycarbonyl)phenyl)-4-(trifluoromethyl)hex-5-en-1-yl 2-(3-cyano-4-isobutoxyphenyl)-4-methylthiazole-5-carboxylate

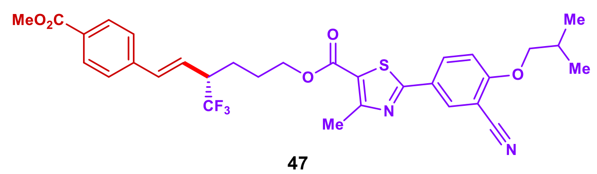

47

The product **47** was purified with silica gel chromatography (PE/EA = 5/1) as a white solid (31.4 mg, 52% yield); mp: 95-97 °C. ^{1}H NMR (500 MHz, $CDCl_3$) δ 8.17 (d, J = 2.3 Hz, 1H), 8.07 (dd, J = 8.9, 2.3 Hz, 1H), 8.00 (d, J = 8.5 Hz, 2H), 7.45 (d, J = 8.4 Hz, 2H), 7.01 (d, J = 8.9 Hz, 1H), 6.65 (d, J = 15.9 Hz, 1H), 6.11 (dd, J = 15.9, 9.3 Hz, 1H), 4.32 (t, J = 6.1 Hz, 2H), 4.03 – 3.77 (m, 5H), 3.04 – 2.89 (m, 1H), 2.75 (s, 3H), 2.24 – 2.16 (m, 1H), 2.03 – 1.97 (m, 1H), 1.93 – 1.86 (m, 1H), 1.84 – 1.69 (m, 2H), 1.09 (d, J = 6.7 Hz, 6H). ^{19}F NMR (471 MHz, $CDCl_3$) δ -70.60 (d, J = 8.7 Hz). ^{13}C NMR (126 MHz, $CDCl_3$) δ 167.5, 166.8, 162.7, 162.1, 161.7, 140.4, 135.7, 132.7, 132.3, 130.2, 129.8, 126.7 (q, J = 280.5 Hz), 126.6, 126.1, 125.1, 121.5, 115.5, 112.8, 103.2, 75.9, 64.6, 52.3, 47.8 (q, J = 26.7 Hz), 29.8, 28.3, 26.0, 24.7, 19.2, 17.6. HRMS (ESI): m/z calcd. for $C_{31}H_{32}F_3N_2O_5S^+$ [M + H$^+$]: 601.1979, found: 601.1982. $[\alpha]_D^{20}$ = 0.3 (c = 1.0, $CHCl_3$), HPLC chiralcel AD-H column (10% isopropanol in hexanes, 1.0 mL/min, λ = 254 nm), t_R = 48.5 min (minor), 60.5 min (major), 95% *ee*.

(*E*)-6-(4-(methoxycarbonyl)phenyl)-4-(trifluoromethyl)hex-5-en-1-yl 2-((*E*)-3-(3,4-dimethoxyphenyl)acrylamido)benzoate

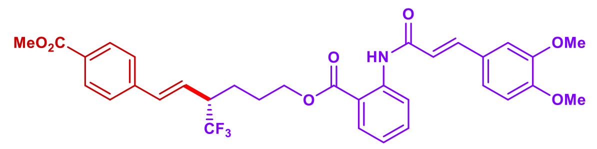

48

The product **48** was purified with silica gel chromatography (PE/EA = 5/1) as a white solid (44.6 mg, 73% yield); mp: 134-136 °C. ^{1}H NMR (500 MHz, $CDCl_3$) δ 11.24 (s, 1H), 8.88 (dd, J = 8.5, 1.2 Hz, 1H), 8.04 – 7.98 (m, 3H), 7.69 (d, J = 15.4 Hz, 1H), 7.60 – 7.56 (m, 1H), 7.44 (d, J = 8.3 Hz, 2H), 7.15 (dd, J = 8.2, 2.0 Hz, 1H), 7.11 – 7.07 (m, 2H), 6.87 (d, J = 8.3 Hz, 1H), 6.64 (d, J = 15.8 Hz, 1H), 6.46 (d, J = 15.5 Hz, 1H), 6.11 (dd, J = 15.9, 9.3 Hz, 1H), 4.38 (t, J = 6.2 Hz, 2H), 3.94 (s, 3H), 3.92 (s, 3H), 3.90 (s, 3H), 2.99 – 2.92 (m, 1H), 2.07 – 2.00 (m, 1H), 1.99 – 1.91 (m, 1H), 1.87 – 1.72 (m, 2H). ^{19}F NMR (471

MHz, $CDCl_3$) δ -70.56 (d, J = 8.7 Hz). ^{13}C NMR (126 MHz, $CDCl_3$) δ 168.6, 166.8, 164.9, 151.1, 149.4, 142.5, 142.3, 140.3, 135.7, 135.0, 130.8, 130.1, 129.9, 127.8, 126.7 (q, J = 279.6 Hz), 126.6, 125.0, 122.7, 122.6, 120.8, 119.7, 114.8, 111.2, 109.9, 64.7, 56.12, 56.09, 52.3, 47.8 (q, J = 27.3 Hz), 26.0, 24.8. HRMS (ESI): m/z calcd. for $C_{33}H_{33}F_3NO_7^+$ [M + H^+]: 612.2204, found: 612.2209. $[\alpha]_D^{20}$ = 1.2 (c = 1.0, $CHCl_3$), HPLC chiralcel OD-H column (50% isopropanol in hexanes, 0.5 mL/min, λ = 254 nm), t_R = 21.9 min (minor), 41.5 min (major), 95% *ee*.

Unsuccessful substrates

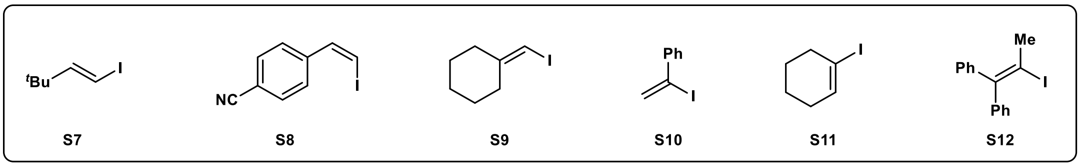

Supplementary Figure 1. Unsuccessful substrates

Among these unsuccessful substrates, **S7-S11** only gave trace amount of coupling products while **S12** failed to proceed the coupling reaction with starting material remained.

Synthetic Utility

1 mmol scale reaction

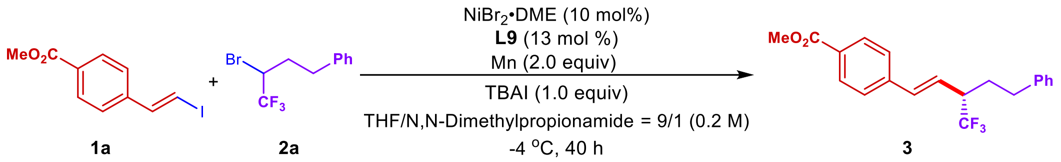

$NiBr_2$•DME (10 mol%, 0.1 mmol, 31 mg), **L9** (13 mol%, 0.13 mmol, 87 mg), Mn powder (2.0 equiv, 2.0 mmol, 110 mg), TBAI (1.0 equiv, 1.0 mmol, 369 mg) and vinyl iodide **1a** (1.0 equiv, 1.0 mmol, 288 mg) were firstly combined in a 25 mL oven-dried sealing tube. The vessel was evacuated and backfilled with N_2 (repeated for 3 times). Alkyl bromide **2a** (2.0 equiv, 2.0 mmol, 532 mg), THF (4.5 mL) and N,N-Dimethylpropionamide (0.5 mL) were added via syringe. The tube was sealed with a Teflon lined cap and stirred at -4 ˚C for 40 h. The reaction mixture was then diluted with ethyl acetate (~20 mL) and filtered through a pad of celite. The filtrate was added brine (20 mL) and extracted with ethyl acetate (3 × 15 mL), the combined organic layer was dried over Na_2SO_4, filtrated and concentrated under vacuum. The residue was then purified by flash column chromatography to give coupling product **3** in 79% yield (275 mg) with 95% *ee*.

Synthesis of diverse trifluoromethylated chiral analogues

Preparation of chiral trifluoromethylated alkane

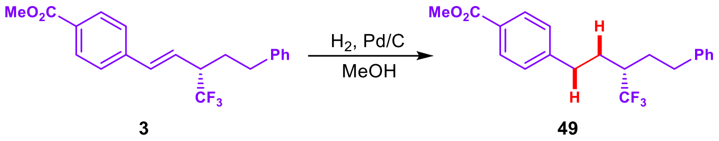

The chiral α-CF_3-substituted olefin **3** (34.8 mg, 0.1 mmol, 1.0 equiv.), Pd/C (5 mg) was added to a 25 mL oven-dried sealing tube. After that, the system was evacuated and a hydrogen-filled balloon was attached. The reaction was stirred at room temperature for 12 hours. The reaction mixture was then passed through a short pad of silica gel, with ethyl acetate as the eluent (~35 mL). The resulting mixture was concentrated, and the residue was purified by chromatography on silica gel to give the desired product **49**.

methyl (S)-4-(5-phenyl-3-(trifluoromethyl)pentyl)benzoate

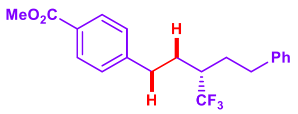

49

The product **49** was purified with silica gel chromatography (PE/EA = 30/1) as a colorless oil (32.6 mg, 93% yield). ^{1}H NMR (400 MHz, $CDCl_3$) δ 8.00 (d, *J* = 8.0 Hz, 2H), 7.34 (t, *J* = 7.3 Hz, 2H), 7.29 – 7.26 (m, 1H), 7.23 (d, *J* = 8.0 Hz, 2H), 7.20 – 7.18 (m, 2H), 3.95 (s, 3H), 2.79 – 2.71 (m, 4H), 2.18 – 1.97 (m, 3H), 1.88 – 1.78 (m, 2H). ^{19}F NMR (376 MHz, $CDCl_3$) δ -69.59 (d, *J* = 9.2 Hz). ^{13}C NMR (126 MHz, $CDCl_3$) δ 167.1, 146.7, 141.1, 130.0, 128.7, 128.62 (q, *J* = 281.0 Hz), 128.55, 128.5, 128.4, 126.4, 52.2, 41.3 (q, *J* = 24.9 Hz), 33.0, 29.8, 29.5. HRMS (ESI): m/z calcd. for $C_{20}H_{21}F_3NaO_2^+$ [M + Na$^+$]: 373.1386, found: 373.1388. $[\alpha]_D^{20}$ = 0.2 (c = 1.0, $CHCl_3$), HPLC chiralcel OD-H column (2% isopropanol in hexanes, 1.0 mL/min, λ = 214 nm), t_R = 24.9 min (minor), 28.0 min (major), 95% *ee*.

Preparation of chiral trifluoromethylated styrene oxide derivates

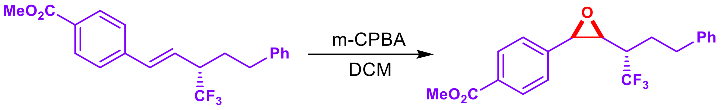

3 **50**

To a test tube, the chiral *α*-CF_3-substituted olefin **3** (34.8 mg, 0.1 mmol, 1.0 equiv.) and m-CPBA (138.1 mg, 0.8 mmol, 8.0 equiv.) in 1.5 mL of dichloromethane were stirred for 5 days at room temperature. After completion, the residue was purified by chromatography on silica gel to give the desired product **50**.

methyl 4-(3-((S)-1,1,1-trifluoro-4-phenylbutan-2-yl)oxiran-2-yl)benzoate

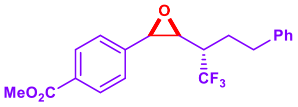

50

The product **50** was purified with silica gel chromatography (PE/EA = 20/1) as a colorless oil (23.1 mg, 63% yield), *dr* = 2:1. **50-major**: ^{1}H NMR (500 MHz, $CDCl_3$) δ 8.04 (d, *J* = 8.4 Hz, 2H), 7.36– 7.31 (m, 4H), 7.28 – 7.25 (m, 2H), 7.24 – 7.22 (m, 1H), 3.92 (s, 3H), 3.84 (d, *J* = 1.9 Hz, 1H), 3.01 – 2.93 (m, 2H), 2.90– 2.84 (m, 1H), 2.22 – 2.02 (m, 3H). ^{19}F NMR (471 MHz, $CDCl_3$) δ -68.69 (d, *J* = 8.4 Hz). ^{13}C NMR (126 MHz, $CDCl_3$) δ 166.8, 141.3, 140.7, 130.4, 130.0, 128.7, 128.6, 126.7 (q, *J* = 280.9 Hz), 126.5, 125.5, 60.7 (q, *J* = 3.6 Hz), 57.1, 52.3, 46.4 (q, *J* = 25.5 Hz), 32.6, 28.9. $[\alpha]_D^{20}$ = 0.6 (c = 1.6, $CHCl_3$), HPLC chiralcel OD-H column (5% isopropanol in hexanes, 1.0 mL/min, λ = 240 nm), t_R = 9.8 min (minor), 16.8 min (major), 95% *ee*. **50-minor**: ^{1}H NMR (400 MHz, $CDCl_3$) δ 8.04 (d, *J* = 8.4 Hz, 2H), 7.35 – 7.30 (m, 4H), 7.25 – 7.21 (m, 1H), 7.20 – 7.17 (m, 2H), 3.93 (s, 3H), 3.65 (d, *J* = 2.0 Hz,

1H), 3.05 (dd, J = 7.9, 2.0 Hz, 1H), 2.85 – 2.71 (m, 2H), 2.25 – 2.05 (m, 2H), 1.94 – 1.84 (m, 1H). ^{19}F NMR (471 MHz, $CDCl_3$) δ -69.35 (d, J = 8.6 Hz). ^{13}C NMR (126 MHz, $CDCl_3$) δ 166.8, 141.3, 140.2, 130.6, 130.1, 128.9, 128.4, 127.1 (q, J = 281.7 Hz), 126.8, 125.6, 59.8, 56.1, 52.4, 45.6 (q, J = 25.7 Hz), 33.2, 26.7. $[\alpha]_D^{20}$ = 0.9 (c = 0.8, $CHCl_3$), HPLC chiralcel OD-H column (10% isopropanol in hexanes, 1.0 mL/min, λ = 240 nm), t_R = 10.4 min (minor), 16.1 min (major), 95% *ee*. HRMS (ESI): m/z calcd. for $C_{20}H_{20}F_3O_3^+$ [M + H$^+$]: 365.1359, found: 365.1373.

Preparation of chiral trifluoromethylated dihydroxylated alkane

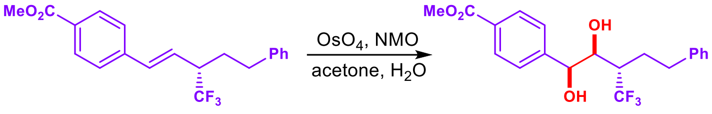

3 **51**

To a solution of the chiral *α*-CF_3-substituted olefin **3** (34.8 mg, 0.1 mmol, 1.0 equiv.) in acetone (0.4 mL) and water (0.1 mL) was added OsO_4 (32 µL, 5.0 mol %, 4.0 wt% in H_2O) and then N-methyl-morpholineN-oxide (17.6 mg, 0.15 mmol, 1.5 equiv). The mixture was stirred for 24 h at room temperature and then quenched by the addition of an aqueous saturated solution of Na_2SO_3 (5.0 mL). The organic phase was extracted with ethyl acetate (3 × 20 mL), the combined organic phases were washed with brine, dried over Na_2SO_4, and concentrated in vacuo. The residue was purified by chromatography on silica gel to give the desired product **51**.

.

methyl 4-((3S)-1,2-dihydroxy-5-phenyl-3-(trifluoromethyl)pentyl)benzoate

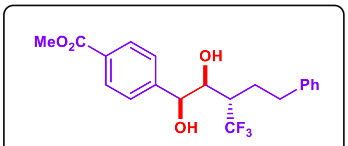

51

The product **51** was purified with silica gel chromatography (PE/EA = 3/1) as a colorless oil (27.5 mg, 72% yield), *dr* = 2:1. ^{1}H NMR (500 MHz, $CDCl_3$) δ 7.99 (d, J = 8.4 Hz, 4H), 7.88 (d, J = 8.4 Hz, 2H), 7.36 – 7.28 (m, 7H), 7.21 – 7.13 (m, 8H), 6.98 – 6.96 (m, 6H), 4.88 (d, J = 6.6 Hz, 2H), 4.44 (d, J = 7.9 Hz, 1H), 4.03 (d, J = 8.0 Hz, 1H), 3.93 (s, 6H), 3.91 – 3.88 (m, J = 13.1 Hz, 5H), 3.02 – 2.82 (m, 7H), 2.70 – 2.59 (m, 3H), 2.53 – 2.47 (m, 2H), 2.14 – 1.93 (m, 9H). ^{19}F NMR (471 MHz, $CDCl_3$) δ -63.70 (d, J = 8.7 Hz), -67.87 (d, J = 10.0 Hz). ^{13}C NMR (126 MHz, $CDCl_3$) δ 166.84, 166.80, 145.4, 144.5, 141.0, 140.2, 130.2, 130.10, 130.05, 130.0, 128.84, 128.82, 128.6, 128.3, 128.0 (q, J = 281.7 Hz), 127.6 (q, J = 282.1 Hz), 127.0, 126.8, 126.5, 126.3, 74.6, 74.5, 74.3, 73.0, 52.31, 52.28, 43.0 (q, J = 24.0 Hz), 42.3 (q, J = 24.0 Hz), 33.5, 32.8, 27.6, 25.2. HRMS (ESI): m/z calcd. for $C_{20}H_{21}F_3NaO_4^+$ [M + Na$^+$]: 405.1284, found: 405.1290. $[\alpha]_D^{20}$ = -0.2 (c = 1.0, $CHCl_3$), HPLC chiralcel AY-H column contacted with another OD-H column (20% isopropanol in hexanes, 0.5 mL/min, λ = 254 nm), t_R = 27.1 min (major), 29.7 min

(major), 35.5 min (minor), 40.7 min (minor), 95% *ee*, *dr* = 2:1.

Preparation of chiral β-trifluoromethylated alcohol

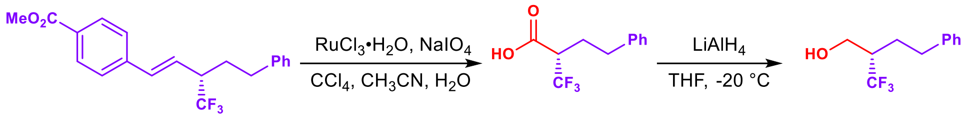

3 **52**

To a solution of chiral *α*-CF_3-substituted olefin **3** (34.8 mg, 0.1 mmol, 1.0 equiv.) in CH_3CN (0.2 mL) was added CCl_4 (0.2 mL), H_2O (0.3 mL), and $NaIO_4$ (88.5 mg, 0.41 mmol, 4.1 equiv.). The biphasic mixture was vigorously stirred, and 5 mg of $RuCl_3 \cdot H_2O$ was added in one portion. After 60 min the mixture was passed through a short pad of silica gel, with dichloromethane as the eluent (~60 mL). The combined organic layers were treated with Na_2SO_4 and concentrated under reduced pressure to afford the crude acid which was used directly.

To a solution of crude trifluoromethylated acid in THF (2 mL) was added $LiAlH_4$ (32 mg, 0.8 mmol, 8.0 equiv.) at -20 °C. Then, the mixture was stirred at room temperature for 4 h. After that, 1N HCl solution was added to quench the reaction. The organic phase was extracted with ethyl acetate (3 × 20 mL), the combined organic phases were washed with brine, dried over Na_2SO_4, and concentrated in vacuo. The residue was purified by chromatography on silica gel to give the desired product **52**.

(S)-4-phenyl-2-(trifluoromethyl)butan-1-ol

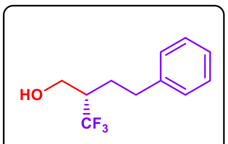

52

The product **52** was purified with silica gel chromatography (PE/EA = 10:1) as a colorless oil (12.2 mg, 56% yield). ^{1}H NMR (400 MHz, $CDCl_3$) δ 7.33 – 7.29 (m, 2H), 7.23 – 7.20 (m, 3H), 3.89 – 3.81 (m, 2H), 2.85 – 2.69 (m, 2H), 2.32 – 2.20 (m, 1H), 2.02 – 1.88 (m, 2H). ^{19}F NMR (376 MHz, $CDCl_3$) δ -68.89 (d, *J* = 9.5 Hz). ^{13}C NMR (126 MHz, $CDCl_3$) δ 141.0, 128.7, 128.5, 128.0 (q, *J* = 281.0 Hz), 126.4, 59.9 (q, *J* = 2.9 Hz), 44.8 (q, *J* = 24.2 Hz), 32.9, 26.4. HRMS (EI): m/z calcd. for $C_{11}H_{13}F_3O$ [M]: 218.0918, found: 218.0909. $[\alpha]_D^{20}$ = 0.4 (c = 0.5, $CHCl_3$), HPLC chiralcel AD-H column (2% isopropanol in hexanes, 1.0 mL/min, λ = 214 nm), t_R = 14.9 min (minor), 15.8 min (major), 94% *ee*.

Preparation of chiral trifluoromethylated allyl alcohol

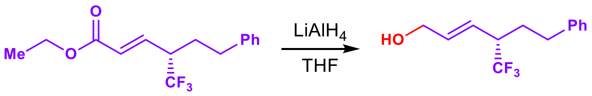

22 **53**

To a solution of chiral α-CF_3-substituted olefin **22** (14.3 mg, 0.05 mmol, 1.0 equiv.) in THF (1 mL) was added $LiAlH_4$ (4 mg, 0.1 mmol, 2.0 equiv.) at 0 °C. Then, the mixture was stirred at room temperature for 4 h. After that, 1N HCl solution was added to quench the reaction. The organic phase was extracted with ethyl acetate (3 × 20 mL), the combined organic phases were washed with brine, dried over Na_2SO_4, and concentrated in vacuo. The residue was purified by chromatography on silica gel to give the desired product **53**.

(S,E)-6-phenyl-4-(trifluoromethyl)hex-2-en-1-ol

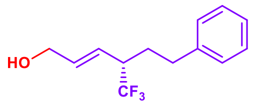

53

The product **53** was purified with silica gel chromatography (PE/EA = 10:1) as a colorless oil (8.3 mg, 68% yield). ^{1}H NMR (400 MHz, $CDCl_3$) δ 7.32 – 7.28 (m, 2H), 7.24 – 7.16 (m, 3H), 5.86 (dt, J = 15.5, 5.1 Hz, 1H), 5.60 – 5.53 (m, 1H), 4.20 (dd, J = 5.1, 1.7 Hz, 2H), 2.79 – 2.67 (m, 2H), 2.60 – 2.52 (m, 1H), 2.13 – 2.05 (m, 1H), 1.86 – 1.76 (m, 1H), 1.65 – 1.56 (m, 1H). ^{19}F NMR (376 MHz, $CDCl_3$) δ -70.87 (d, J = 9.6 Hz). ^{13}C NMR (101 MHz, $CDCl_3$) δ 140.8, 136.3, 128.7, 128.6, 126.9 (q, J = 280.7 Hz), 126.4, 124.4 (q, J = 2.5 Hz), 63.0, 46.6 (q, J = 26.5 Hz), 32.6, 29.3. HRMS (EI): m/z calcd. for $C_{13}H_{15}F_3O$ [M]: 244.1075, found: 244.1076. $[\alpha]_D^{20}$ = 0.5 (c = 0.25, $CHCl_3$), HPLC chiralcel AD-H column (1% isopropanol in hexanes, 1.0 mL/min, λ = 214 nm), t_R = 27.4 min (minor), 30.5 min (major), 97% *ee*.

Preparation of chiral trifluoromethylated ketone

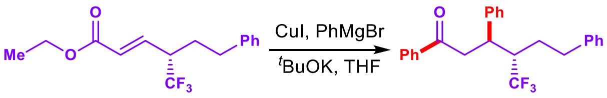

22 **54**

CuI (4.0 mg, 0.021 mmol, 0.3 equiv.) was firstly added to a 10 mL oven-dried sealing tube. The vessel was evacuated and backfilled with N_2 (repeated for 3 times). 0.1 mL of tert-Butyl methyl ether was added to the system and the tube was transferred to -50 °C. Then, PhMgBr solution (0.35 mL, 1M in THF, 5.0 equiv.) was added under N_2 atmosphere. After stirring at this temperature for 10 min, chiral α-CF_3-substituted olefin **22** (20.0 mg, 0.07 mmol, 1.0 equiv.) in tert-Butyl methyl ether (0.2 mL) was added dropwise. The reaction was stirred at -50 °C for additional 8 h. After that, MeOH (0.5 mL) and sat. NH_4Cl solution (1.0 mL) was added to quench the reaction. The organic phase was extracted with ethyl acetate (3 × 20 mL), the combined organic phases were washed with brine, dried over Na_2SO_4, and concentrated in vacuo. The residue was purified by chromatography on silica gel to give the desired product **54**.

(4S)-1,3,6-triphenyl-4-(trifluoromethyl)hexan-1-one

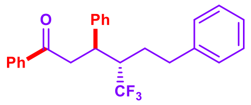

54

The product **54** was purified with silica gel chromatography (PE/EA = 30:1) as a colorless oil (22.5 mg, 81% yield), *dr* = 20:1 according to the ^{19}F NMR. ^{1}H NMR (400 MHz, $CDCl_3$) δ 7.93 – 7.91 (m, 2H), 7.59 – 7.54 (m, 1H), 7.45 (t, *J* = 7.7 Hz, 2H), 7.30 – 7.26 (m, 2H), 7.25 – 7.16 (m, 6H), 7.06 – 7.04 (m, 2H), 4.04 – 3.99 (m, 1H), 3.65 (dd, *J* = 17.7, 9.6 Hz, 1H), 3.40 (dd, *J* = 17.7, 4.8 Hz, 1H), 2.80 – 2.73 (m, 1H), 2.64 – 2.48 (m, 2H), 2.02 – 1.83 (m, 2H). ^{19}F NMR (376 MHz, $CDCl_3$) δ -64.50 (d, *J* = 9.5 Hz), -65.22 (d, *J* = 9.7 Hz). ^{13}C NMR (126 MHz, $CDCl_3$) δ 197.7, 141.0, 140.6, 137.0, 133.4, 128.8, 128.64, 128.62, 128.5, 128.4 (q, *J* = 282.6 Hz), 128.3, 128.1, 127.1, 126.3, 47.5 (q, *J* = 23.6 Hz), 39.0, 38.6, 33.9, 27.2. HRMS (ESI): m/z calcd. for $C_{25}H_{24}F_3O^+$ [M + H$^+$]: 397.1774, found: 397.1779. $[\alpha]_D^{20}$ = 0.2 (c = 0.5, $CHCl_3$), HPLC chiralcel IC column (1% isopropanol in hexanes, 0.5 mL/min, λ = 214 nm), t_R = 13.9 min (minor), 14.6 min (major), 16.5 min (minor), 18.0 min (major), 96% *ee*.

Control experiments

Vinyl bromide insdead of vinyl iodide

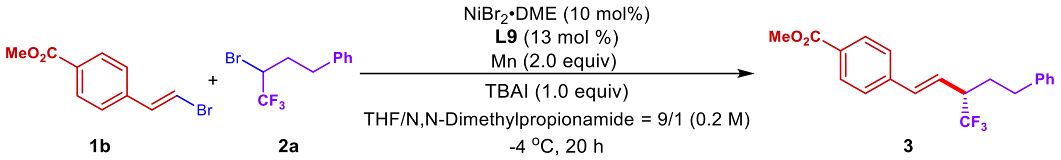

NiBr$_2$•DME (10 mol%, 0.01 mmol, 3.1 mg), **L9** (13 mol%, 0.013 mmol, 8.7 mg), Mn powder (2.0 equiv, 0.2 mmol, 11.0 mg), TBAI (1.0 equiv, 0.1 mmol, 36.9 mg) and vinyl bromide **1b** (1.0 equiv, 0.1 mmol, 24.0 mg) were firstly combined in a 10 mL oven-dried sealing tube. The vessel was evacuated and backfilled with N$_2$ (repeated for 3 times). Alkyl bromide **2a** (2.0 equiv, 0.2 mmol, 53.2 mg), THF (0.45 mL) and N,N-Dimethylpropionamide (0.05 mL) were added via syringe. The tube was sealed with a Teflon lined cap and stirred at -4 ˚C for 20 h. The reaction mixture was then diluted with ethyl acetate (~20 mL) and filtered through a pad of celite. The filtrate was added brine (20 mL) and extracted with ethyl acetate (3 × 15 mL), the combined organic layer was dried over Na$_2$SO$_4$, filtrated and concentrated under vacuum. The residue was then purified by flash column chromatography to give coupling product **3** in 81% yield (28.2 mg) with 94% *ee*.

HPLC DATA:

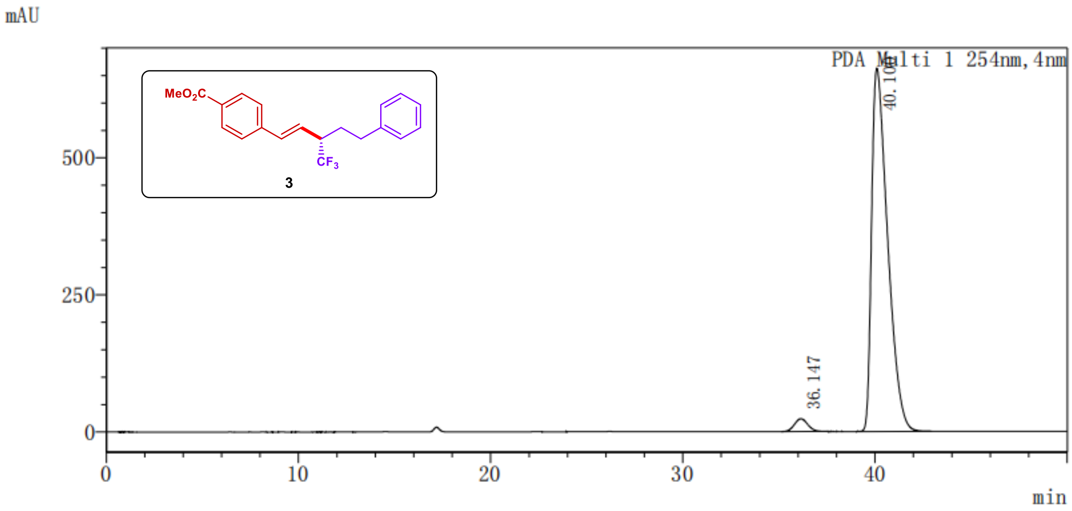

Peak#	Ret. Time	Area	Area#
1	36.147	1116706	2.828
2	40.100	38364196	97.172
Total		39480902	100.000

Supplementary Figure 2. Chiral HPLC analysis of Compound **3**

Isomerization of product 3

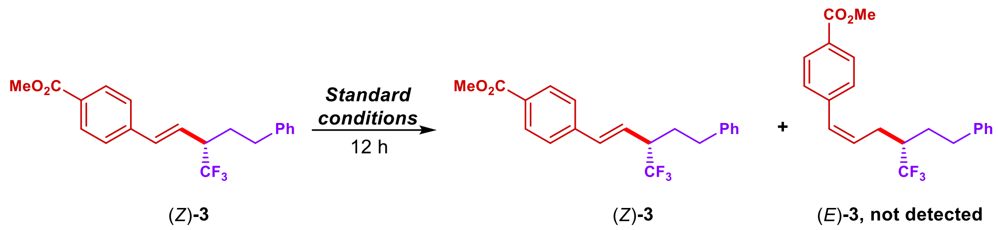

NiBr$_2$•DME (10 mol%, 0.01 mmol, 3.1 mg), **L9** (13 mol%, 0.013 mmol, 8.7 mg), Mn powder (2.0 equiv, 0.2 mmol, 11.0 mg), TBAI (1.0 equiv, 0.1 mmol, 36.9 mg) and coupling product (*Z*)-**3** (1.0 equiv, 0.1 mmol, 34.8 mg) were firstly combined in a 10 mL oven-dried sealing tube. The vessel was evacuated and backfilled with N_2 (repeated for 3 times). THF (0.45 mL) and N,N-Dimethylpropionamide (0.05 mL) were added via syringe. The tube was sealed with a Teflon lined cap and stirred at -4 ˚C for 12 h. The crude ^{19}F NMR spectrum showed that no isomerized product (*E*)-**3** was detected.

Monitoring experiment

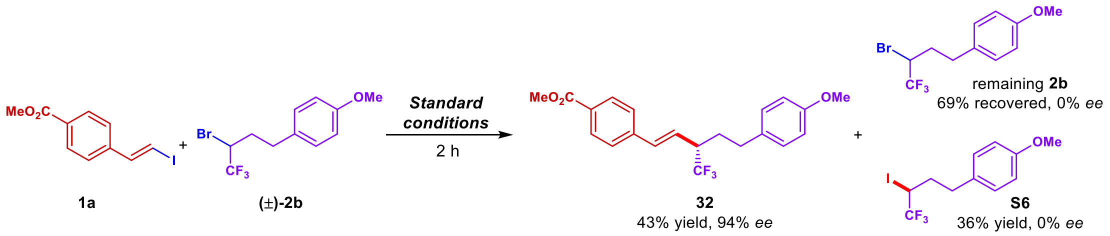

NiBr$_2$•DME (10 mol%, 0.01 mmol, 3.1 mg), **L9** (13 mol%, 0.013 mmol, 8.7 mg), Mn powder (2.0 equiv, 0.2 mmol, 11.0 mg), TBAI (1.0 equiv, 0.1 mmol, 36.9 mg) and vinyl iodide **1a** (1.0 equiv, 0.1 mmol, 28.8 mg) were firstly combined in a 10 mL oven-dried sealing tube. The vessel was evacuated and backfilled with N_2 (repeated for 3 times). Alkyl bromide **2b** (2.0 equiv, 0.2 mmol, 59.2 mg), THF (0.45 mL) and N,N-Dimethylpropionamide (0.05 mL) were added via syringe. The tube was sealed with a Teflon lined cap and stirred at -4 ˚C for 2 h. The crude ^{19}F NMR spectrum showed that trifluoromethyl iodide **S6** and coupling product **32** were obtained in 36% and 43% yields respectively while 69% of **2b** was remaining. The HPLC data showed that trifluoromethyl iodide **S6** and trifluoromethyl bromide **2b** were racemic while **32** had 94% *ee* value.

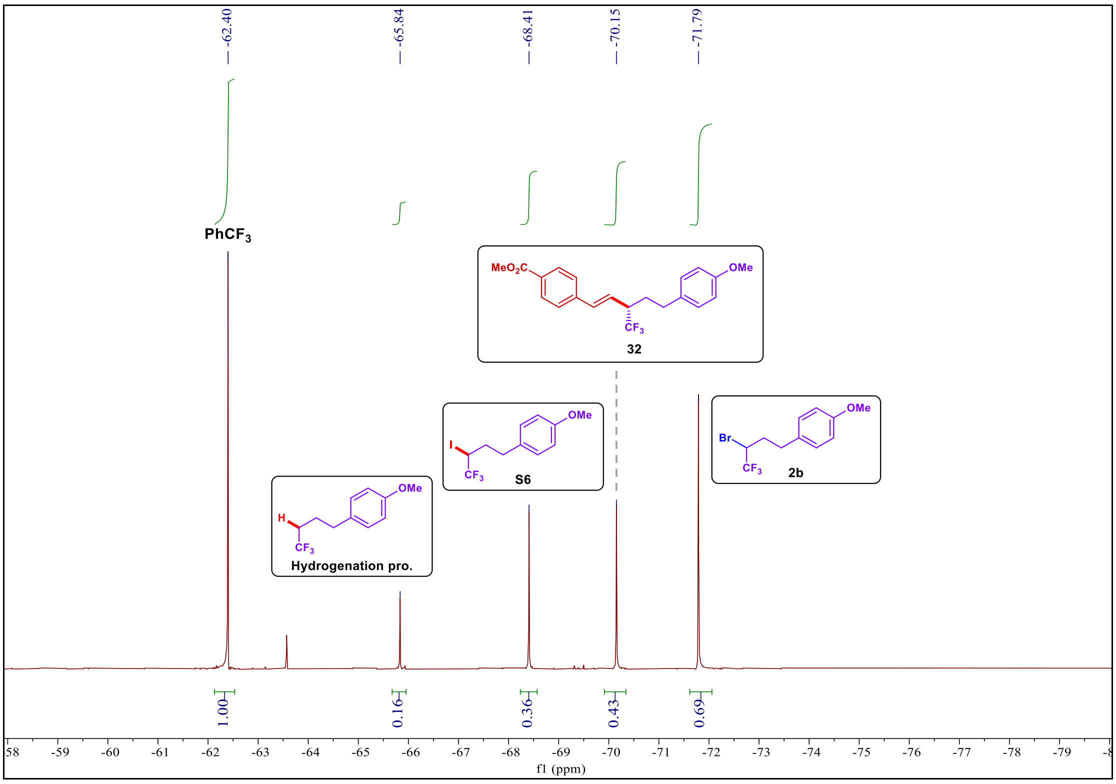

Supplementary Figure 3. Crude ^{19}F NMR Spectrum (376 MHz, $CDCl_3$)

HPLC DATA:

HPLC chiralcel ODH column contacted with another ADH column (0.3% isopropanol in hexanes, 0.3 mL/min, λ = 220 nm).

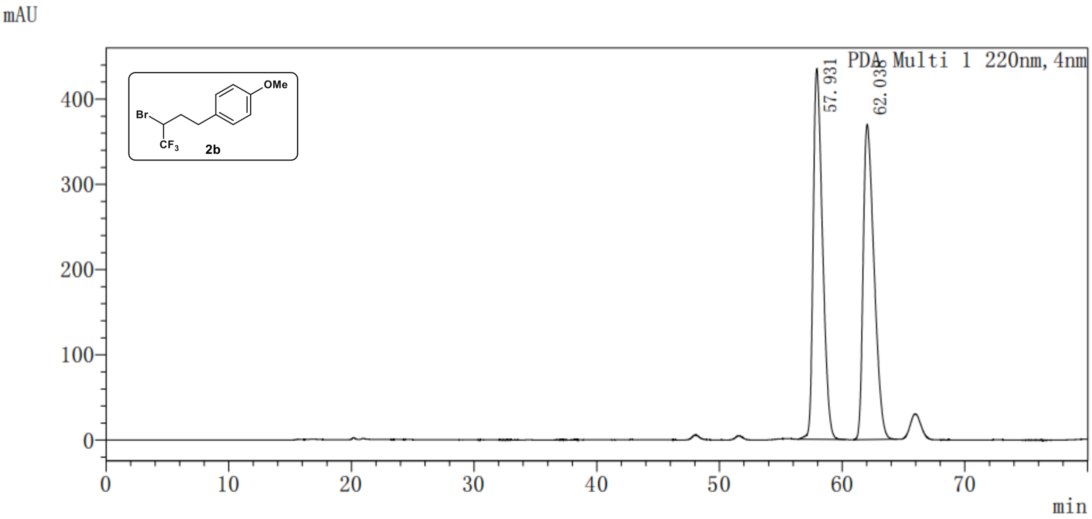

Peak#	Ret. Time	Area	Area#
1	57.931	22930617	50.100
2	62.038	22839077	49.900
Total		45769693	100.000

Supplementary Figure 4. HPLC analysis of Compound **2b**

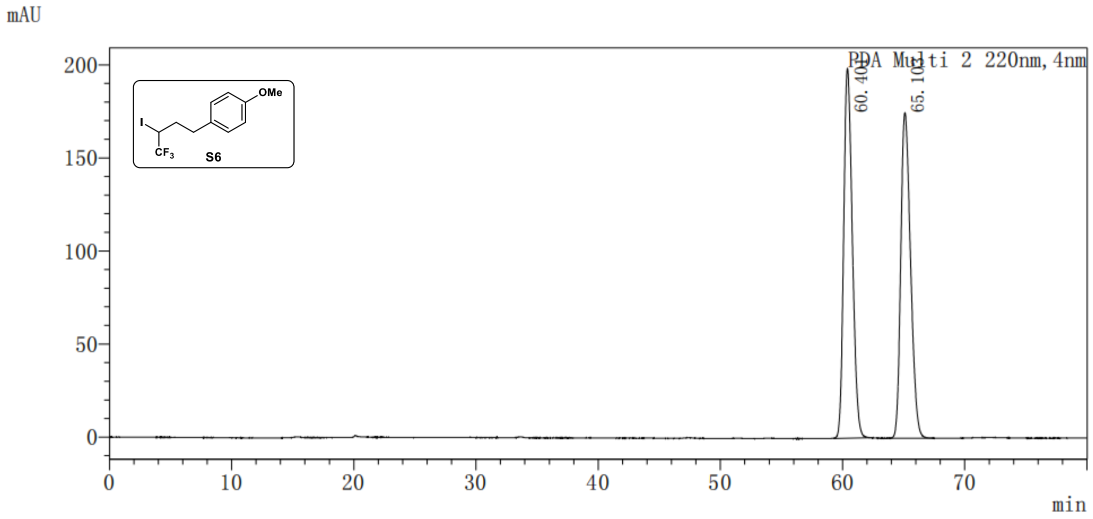

Peak#	Ret. Time	Area	Area#
1	60.401	9763785	50.187
2	65.103	9690953	49.813
Total		19454738	100.000

Supplementary Figure 5. HPLC analysis of Compound **S6**

Since **2b** and **S6** could not be separated by silica gel column chromatography after reaction, the mixture was analyzed by HPLC directly.

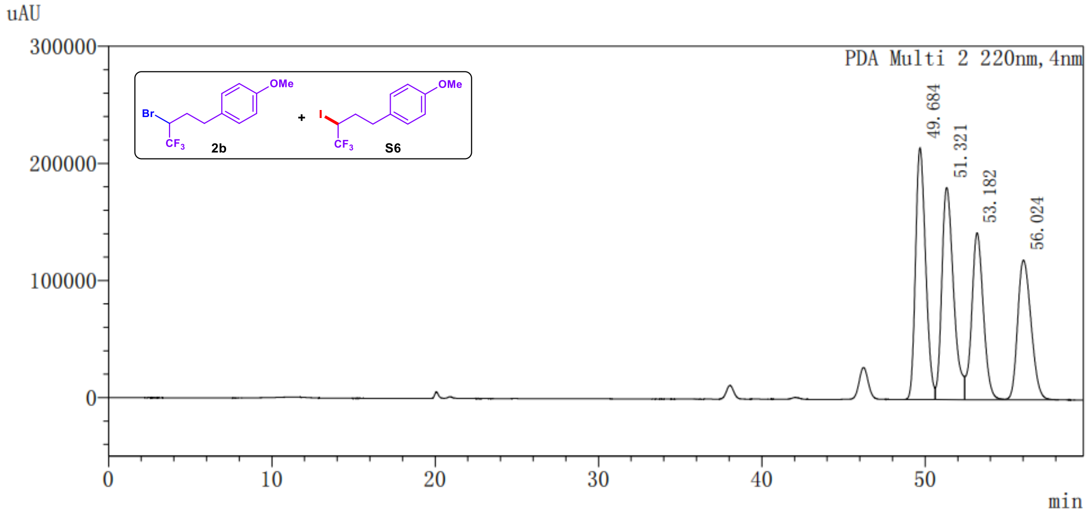

Peak#	Ret. Time	Area	Area#
1	49.684	9265113	28.753
2	51.321	9076915	28.169
3	53.182	7061746	21.915
4	56.024	6819100	21.162
Total		32222874	100.000

Supplementary Figure 6. HPLC analysis of the reaction mixture

X-ray Crystallographic Data

Compound 35: (The crystal structure of compound **35** has been deposited at the Cambridge Crystallographic Data Centre (**CCDC 2170959**). Copies of the data can be obtained free of charge via www.ccdc.cam.ac.uk/data_request/cif.)

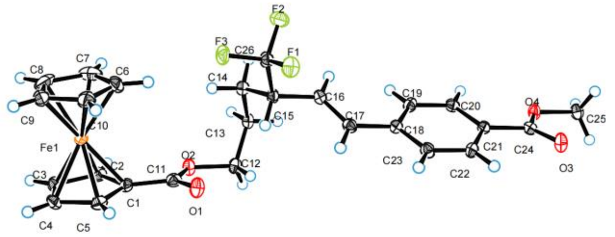

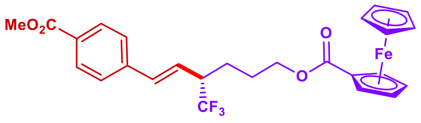

35

Supplementary Table S7. Crystal data and structure refinement for compound **35**.

Identification code	jrx-171-4-100K_auto
Empirical formula	$C_{26}H_{25}F_3FeO_4$
Formula weight	514.31
Temperature/K	99.99(10)
Crystal system	orthorhombic
Space group	$P2_12_12_1$
a/Å	5.93025(10)
b/Å	10.42585(19)
c/Å	36.1580(6)
α/°	90
β/°	90
γ/°	90
Volume/Å^3	2235.57(7)
Z	4
ρ_{calc}g/cm^3	1.528
μ/mm^{-1}	5.916
F(000)	1064.0
Crystal size/mm^3	0.21 × 0.17 × 0.15
Radiation	Cu Kα (λ = 1.54184)
2Θ range for data collection/°	8.828 to 145.808
Index ranges	$-4 \leq h \leq 7$, $-12 \leq k \leq 10$, $-42 \leq l \leq 43$
Reflections collected	5918
Independent reflections	3639 [R_{int} = 0.0288, R_{sigma} = 0.0446]
Data/restraints/parameters	3639/0/309
Goodness-of-fit on F^2	1.035
Final R indexes [I>=2σ (I)]	R_1 = 0.0335, wR_2 = 0.0812
Final R indexes [all data]	R_1 = 0.0377, wR_2 = 0.0836
Largest diff. peak/hole / e Å^{-3}	0.24/-0.36
Flack parameter	-0.006(4)

III. Supplementary Figures

NMR Spectra of New Compounds

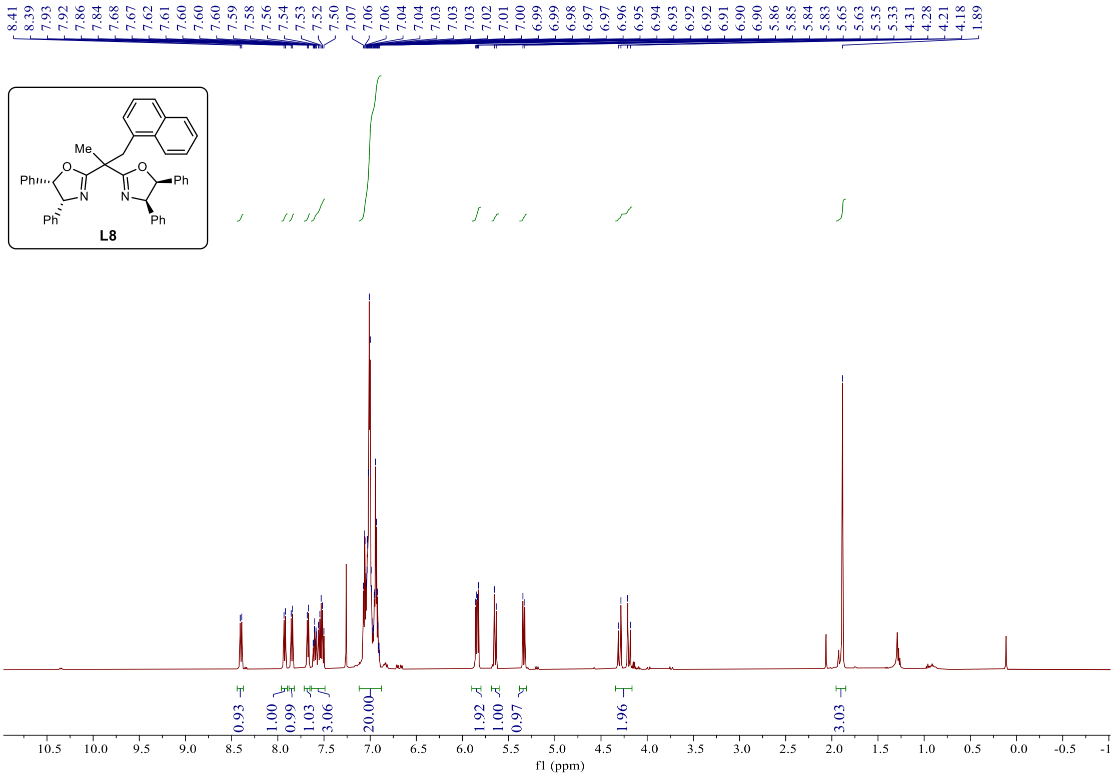

Supplementary Figure 7. ^{1}H NMR Spectrum of Compound **L8** (500 MHz, $CDCl_3$)

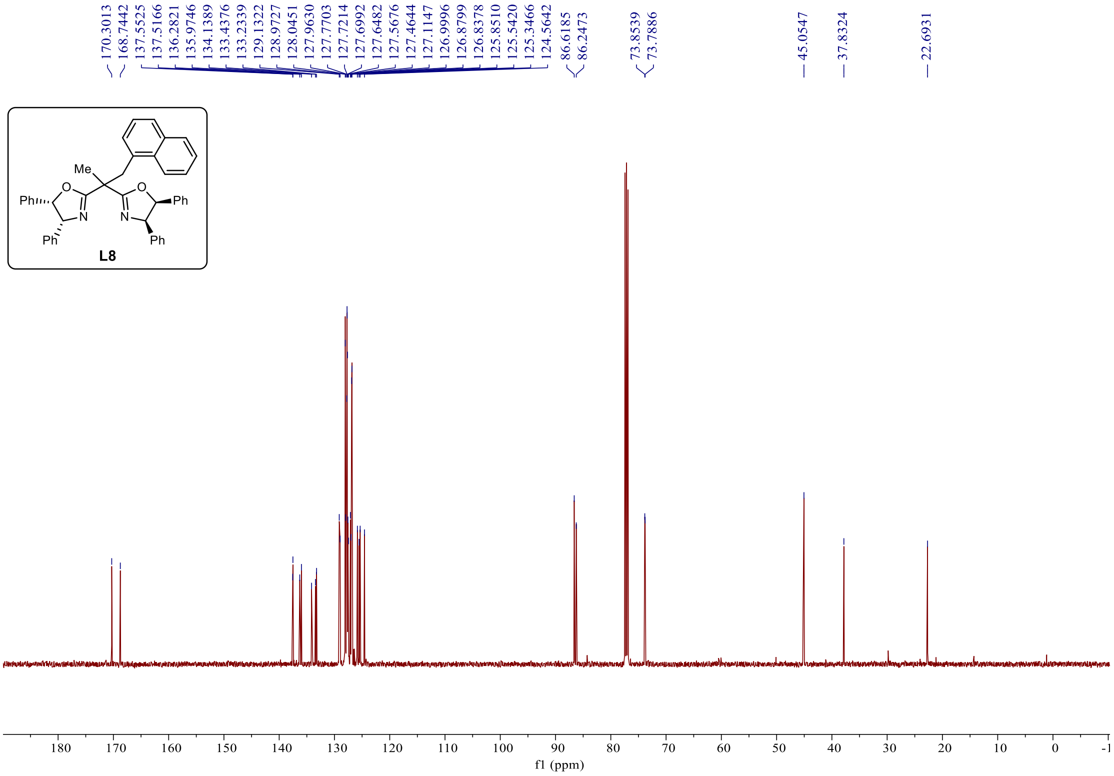

Supplementary Figure 8. ^{13}C NMR Spectrum of Compound **L8** (126 MHz, $CDCl_3$)

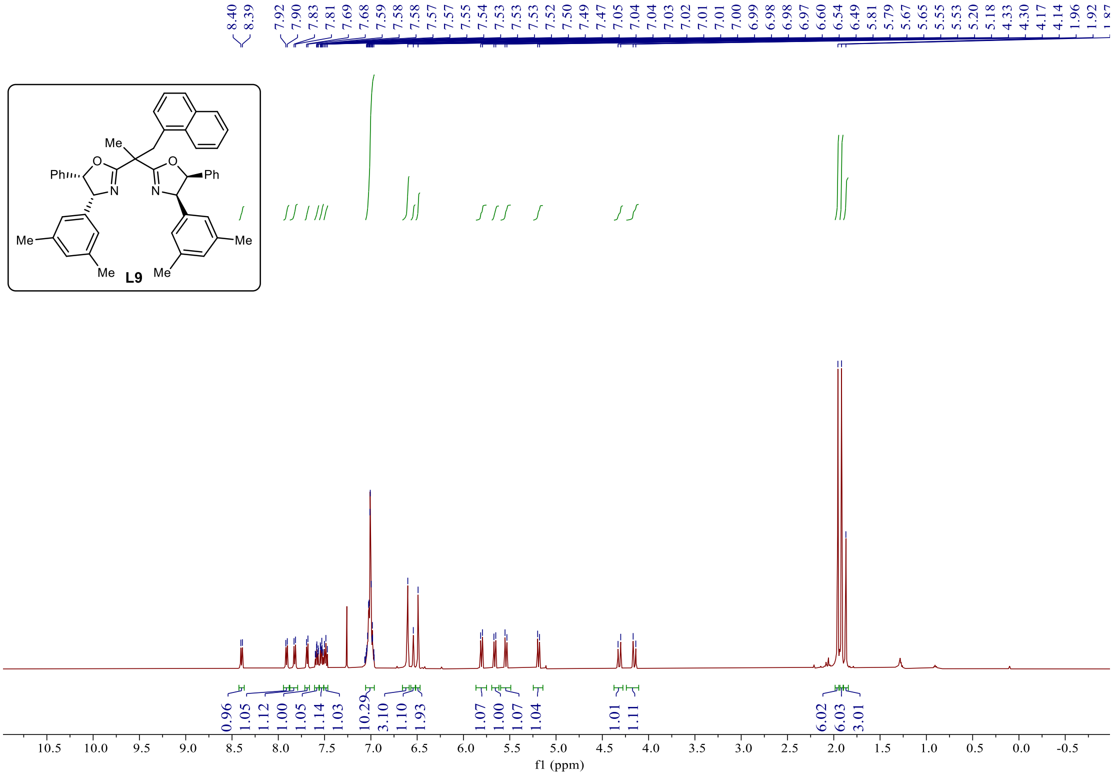

Supplementary Figure 9. ^{1}H NMR Spectrum of Compound **L9** (500 MHz, $CDCl_3$)

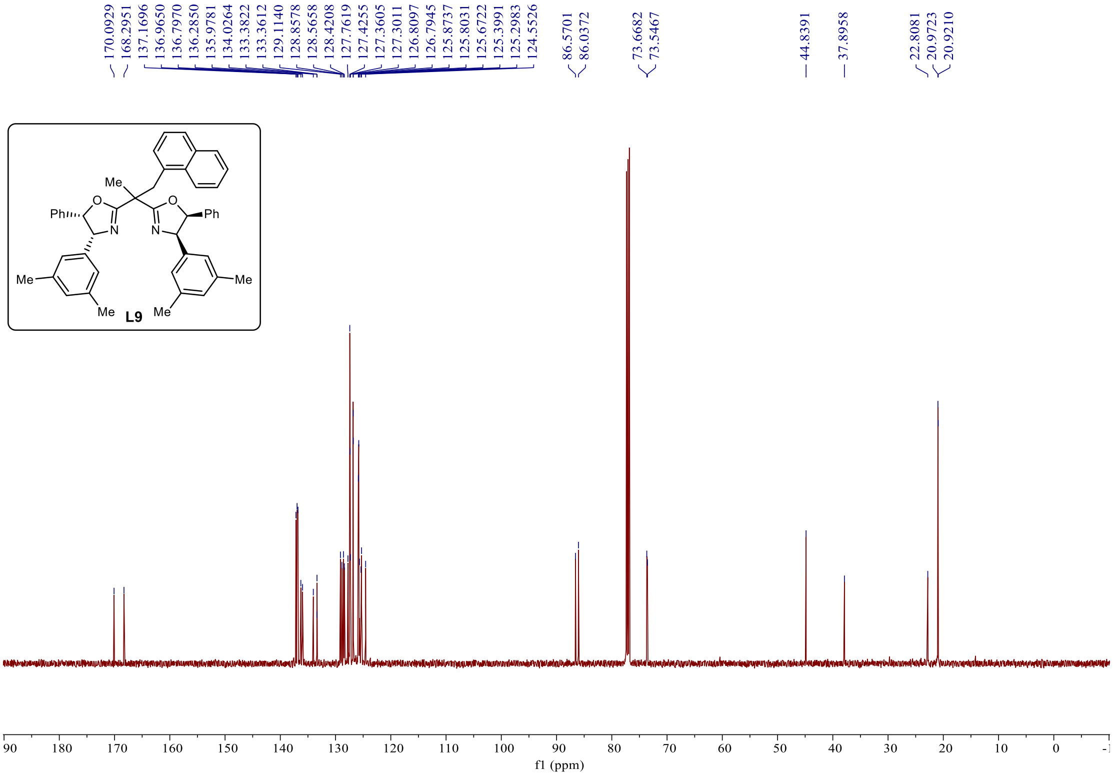

Supplementary Figure 10. ^{13}C NMR Spectrum of Compound **L9** (126 MHz, $CDCl_3$)

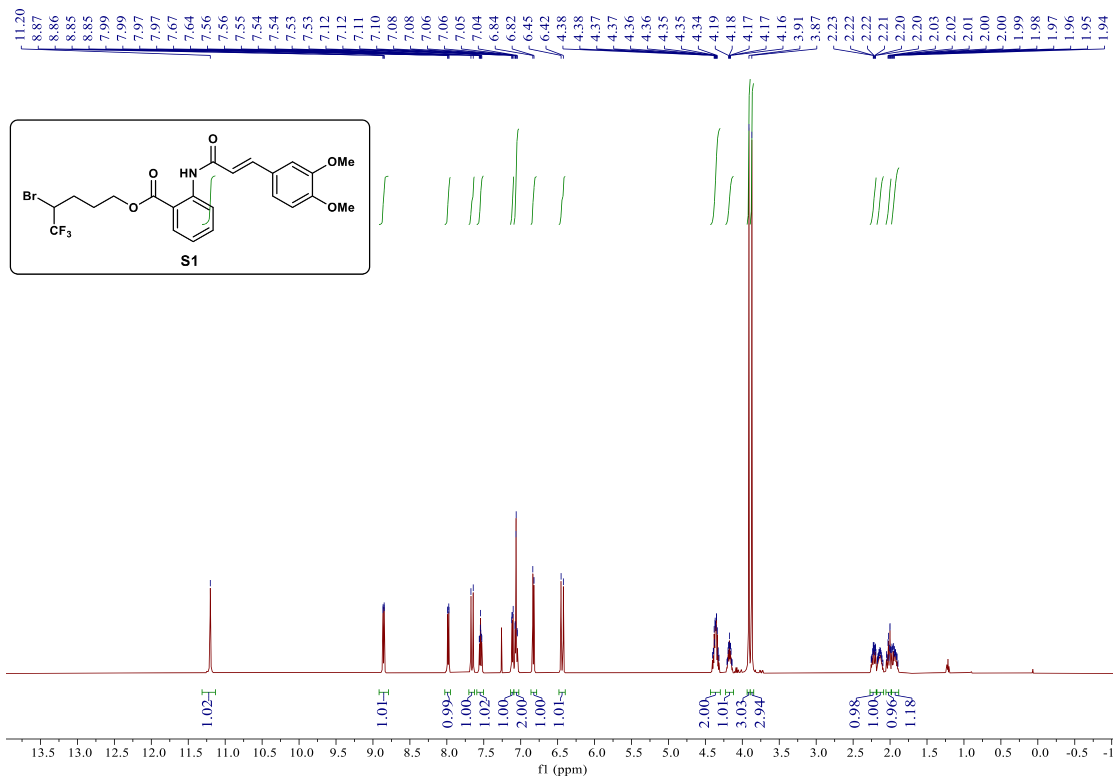

Supplementary Figure 11. ^{1}H NMR Spectrum of Compound **S1** (500 MHz, $CDCl_3$)

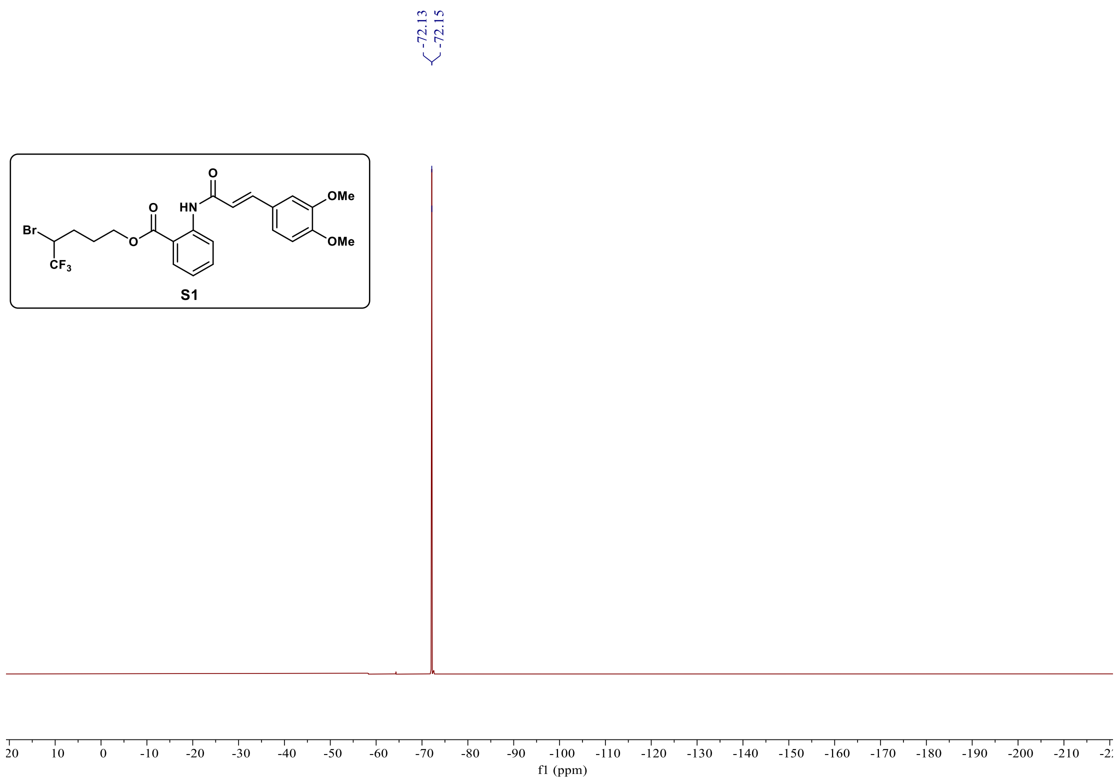

Supplementary Figure 12. ^{19}F NMR Spectrum of Compound **S1** (471 MHz, $CDCl_3$)

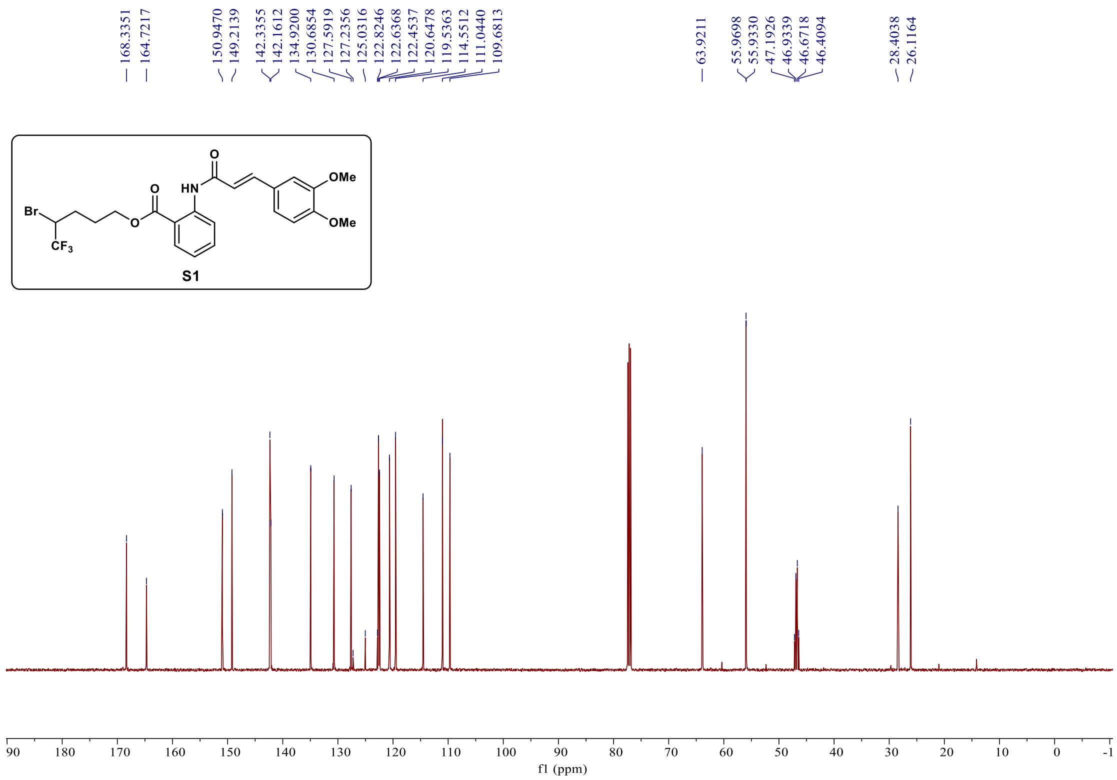

Supplementary Figure 13. ^{13}C NMR Spectrum of Compound **S1** (126 MHz, $CDCl_3$)

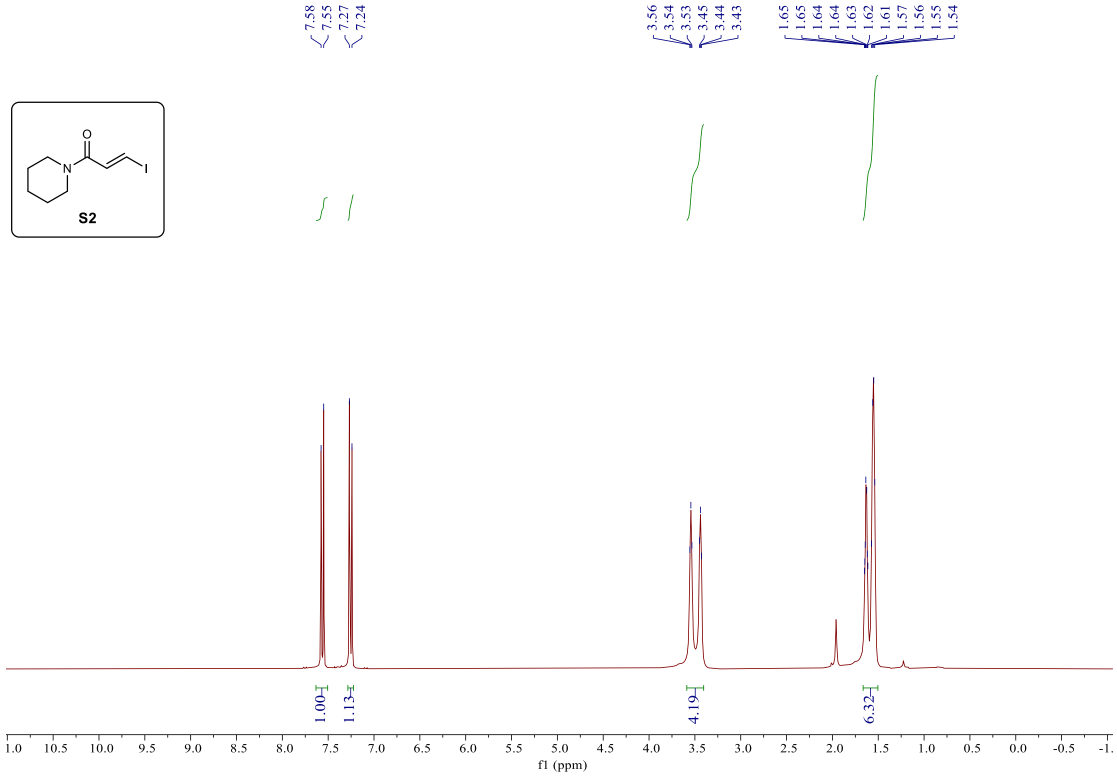

Supplementary Figure 14. ^{1}H NMR Spectrum of Compound **S2** (500 MHz, $CDCl_3$)

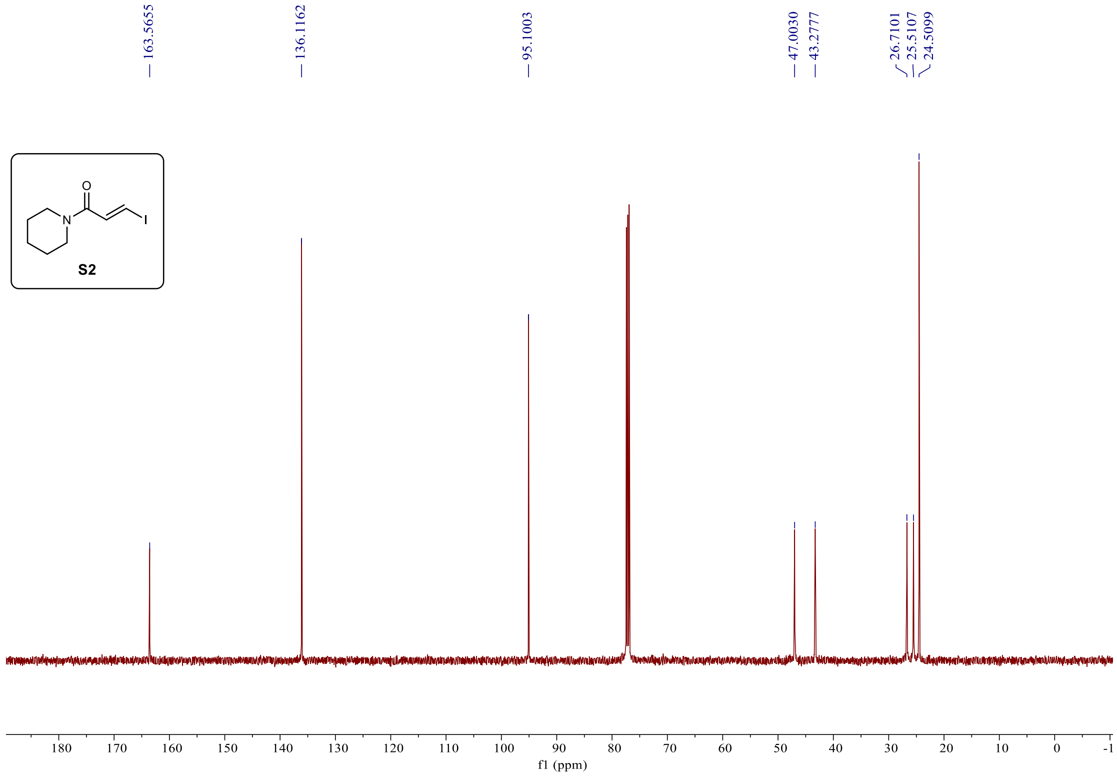

Supplementary Figure 15. ^{13}C NMR Spectrum of Compound **S2** (126 MHz, $CDCl_3$)

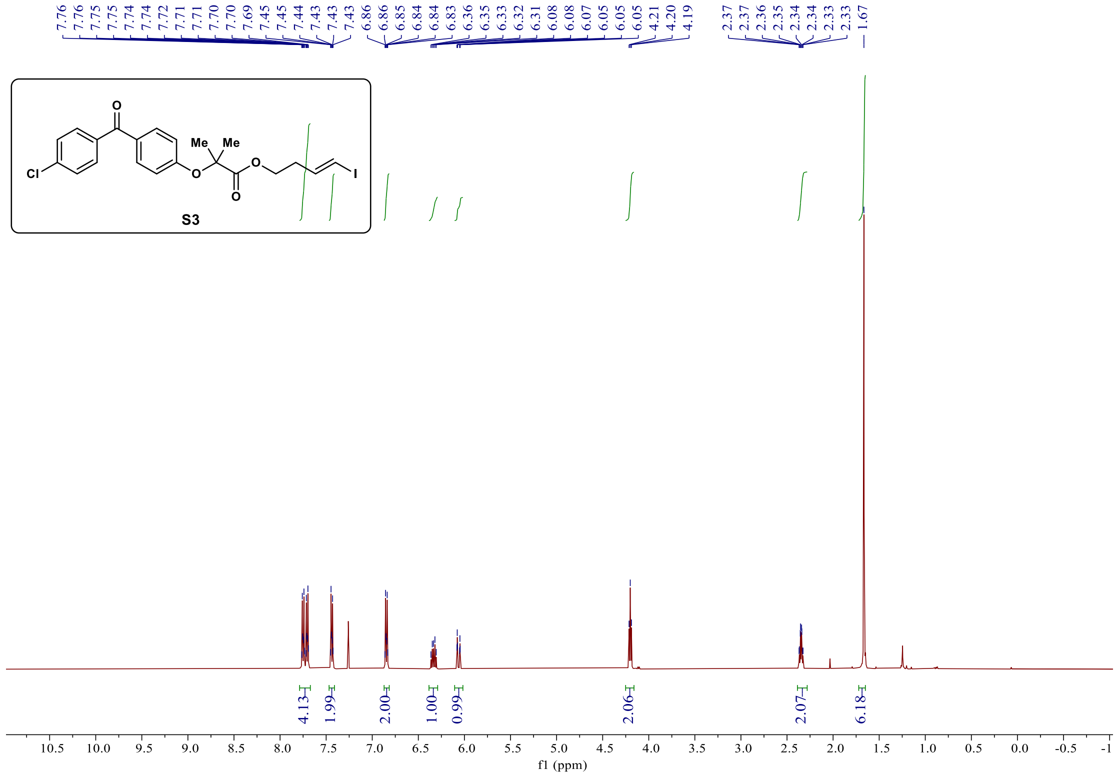

Supplementary Figure 16. ^{1}H NMR Spectrum of Compound **S3** (500 MHz, $CDCl_3$)

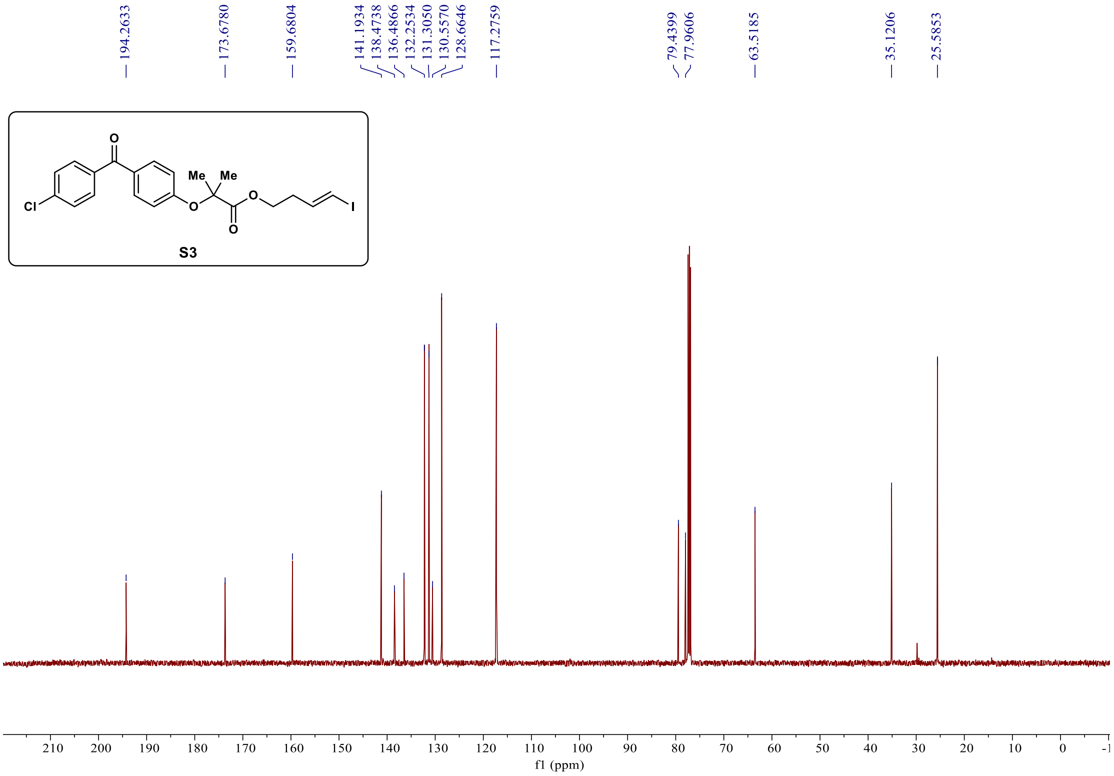

Supplementary Figure 17. ^{13}C NMR Spectrum of Compound **S3** (126 MHz, $CDCl_3$)

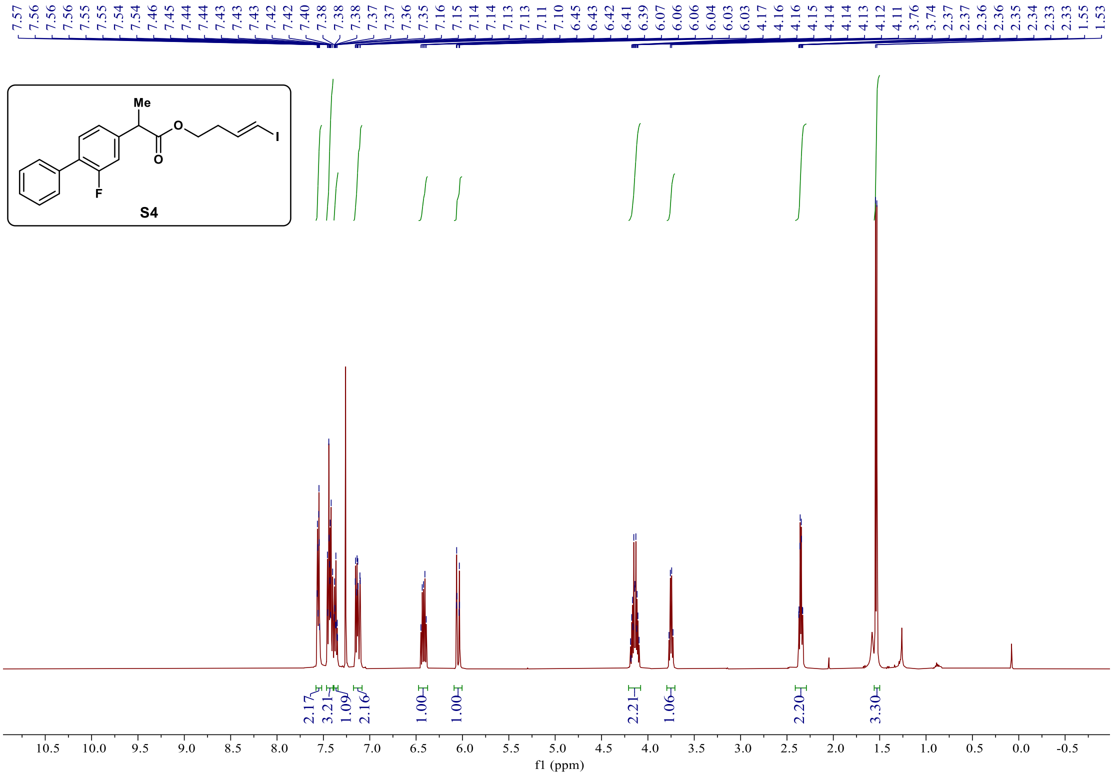

Supplementary Figure 18. ^{1}H NMR Spectrum of Compound **S4** (500 MHz, $CDCl_3$)

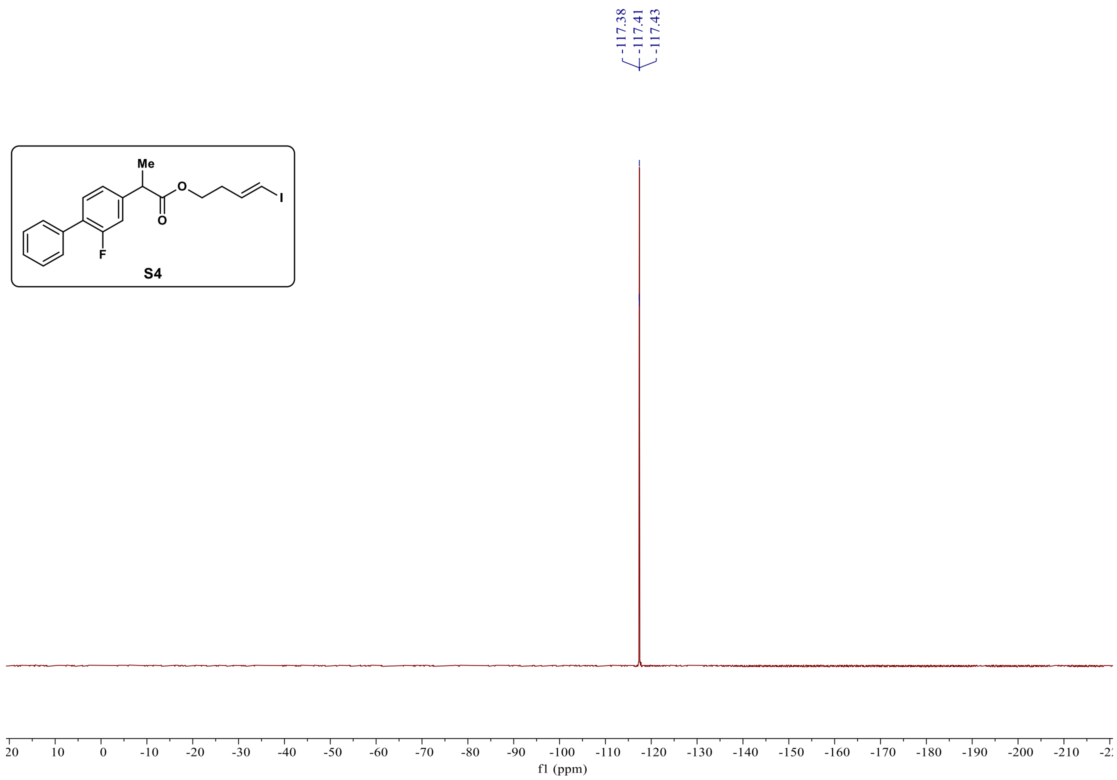

Supplementary Figure 19. ^{19}F NMR Spectrum of Compound **S4** (471 MHz, $CDCl_3$)

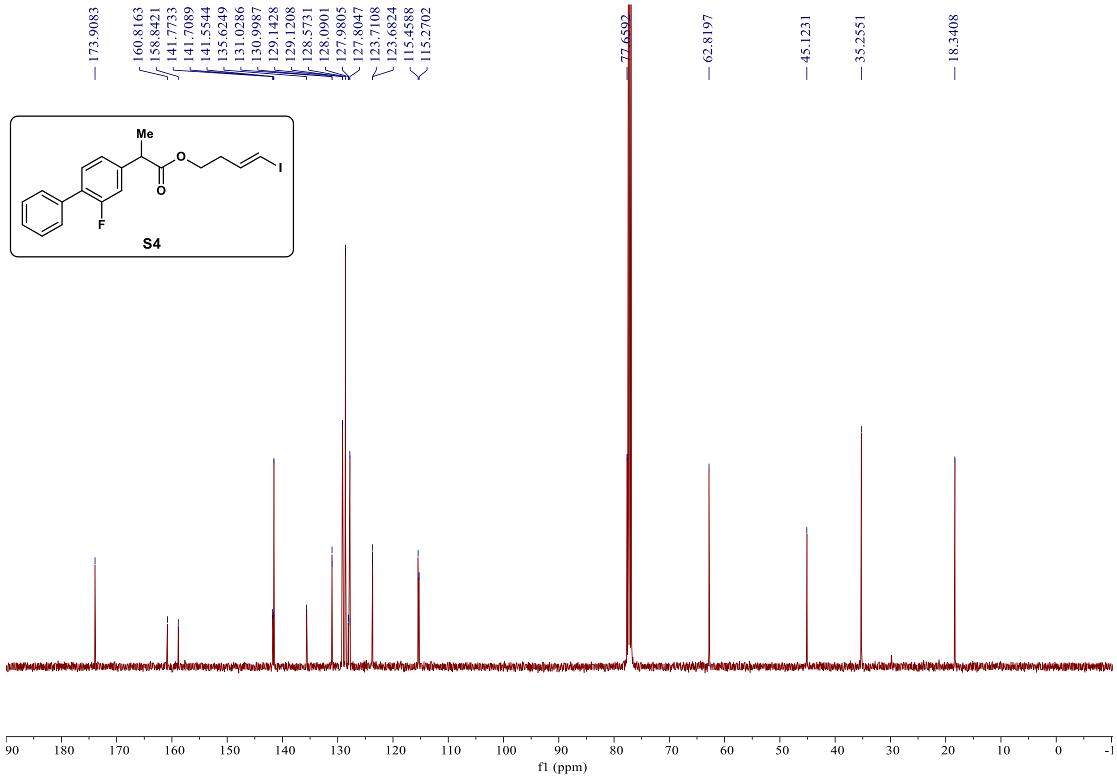

Supplementary Figure 20. ^{13}C NMR Spectrum of Compound **S4** (126 MHz, $CDCl_3$)

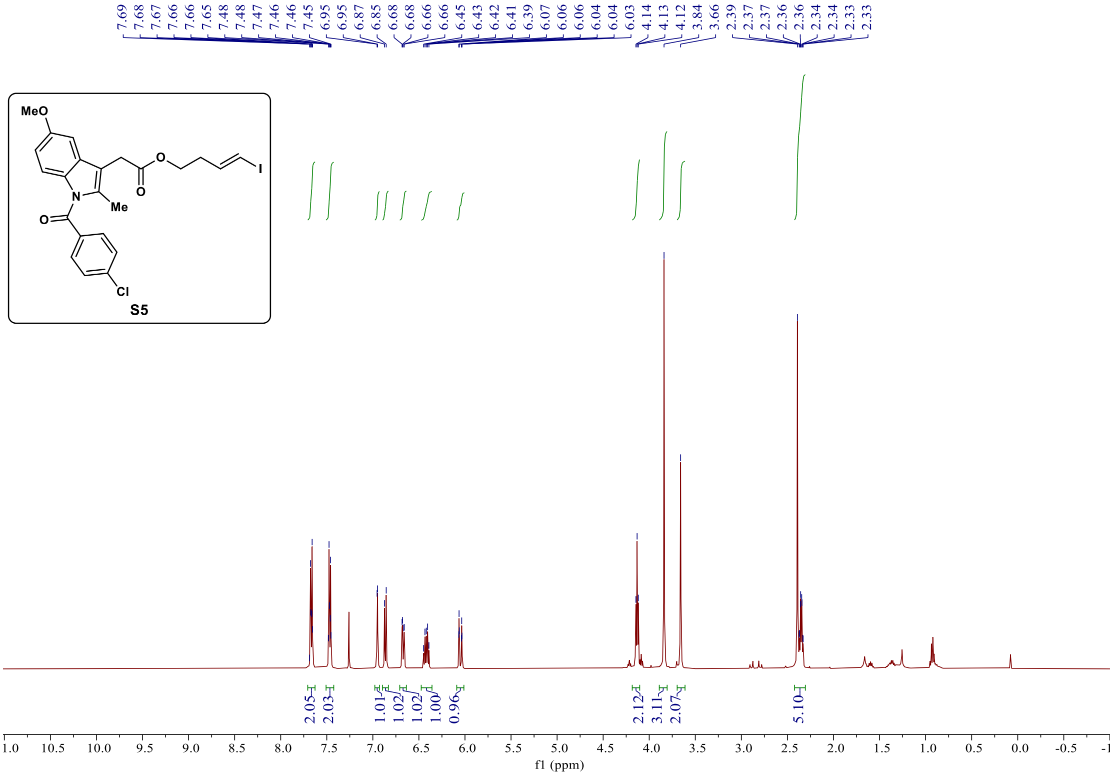

Supplementary Figure 21. ^{1}H NMR Spectrum of Compound **S5** (500 MHz, $CDCl_3$)

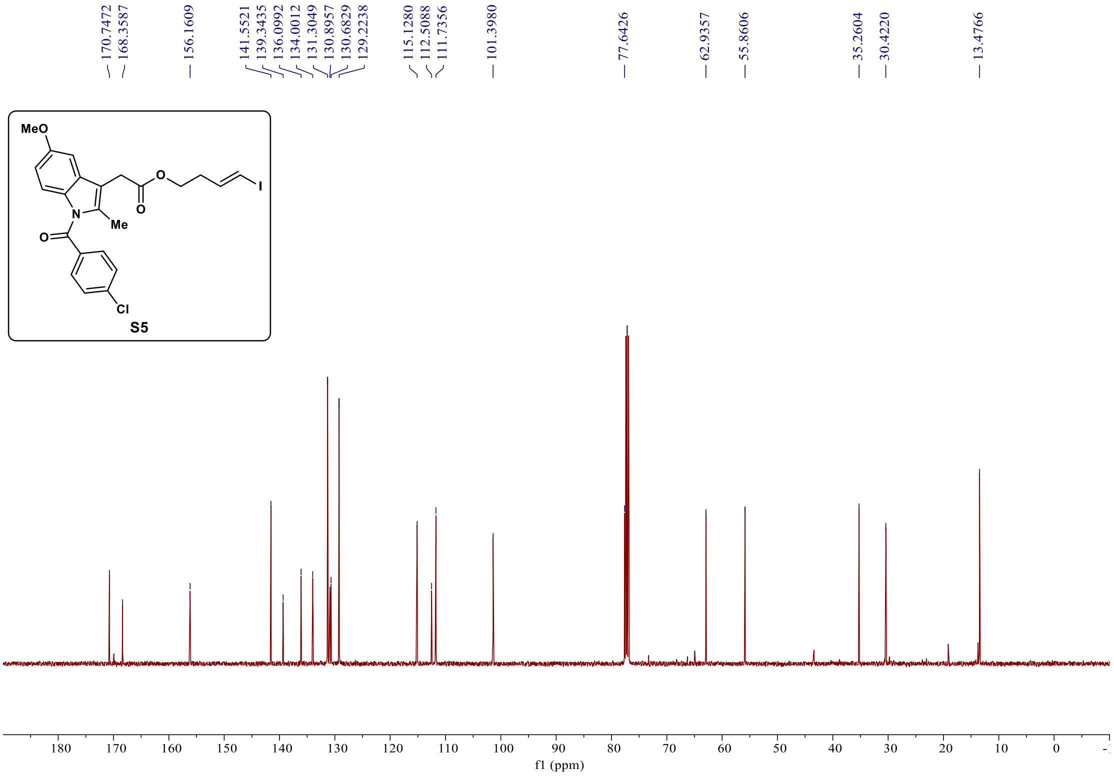

Supplementary Figure 22. ^{13}C NMR Spectrum of Compound **S5** (126 MHz, $CDCl_3$)

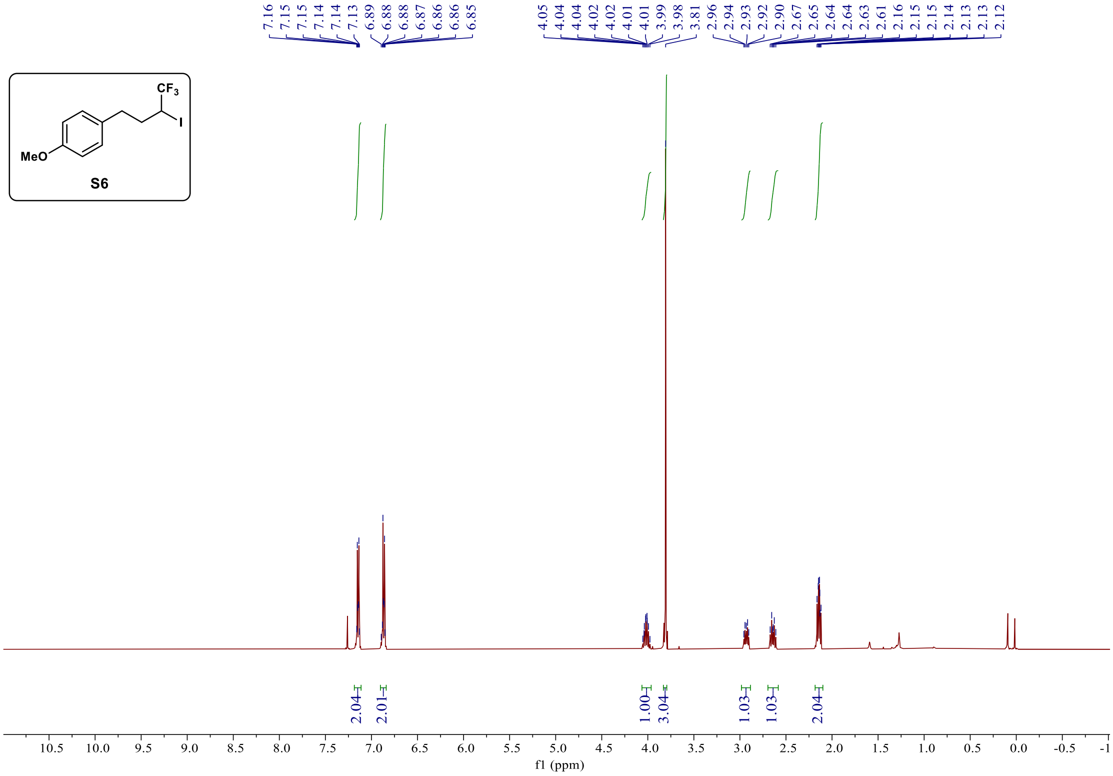

Supplementary Figure 23. ^{1}H NMR Spectrum of Compound **S6** (500 MHz, $CDCl_3$)

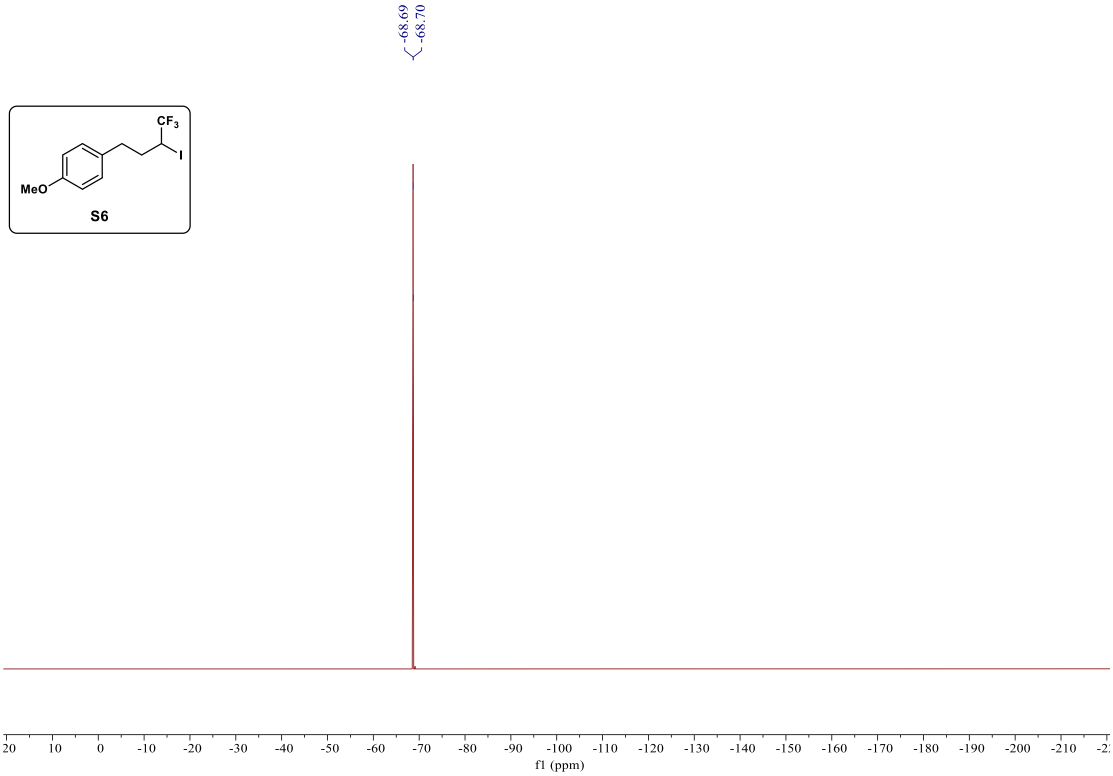

Supplementary Figure 24. ^{19}F NMR Spectrum of Compound **S6** (471 MHz, $CDCl_3$)

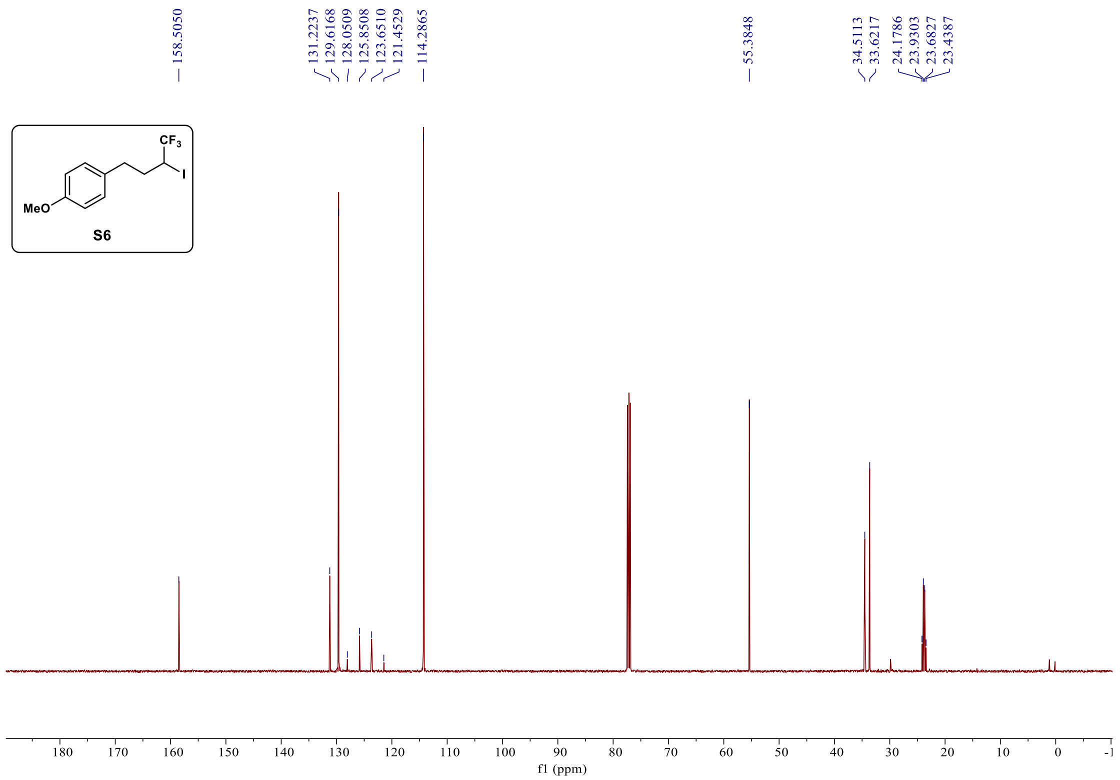

Supplementary Figure 25. ^{13}C NMR Spectrum of Compound **S6** (126 MHz, $CDCl_3$)

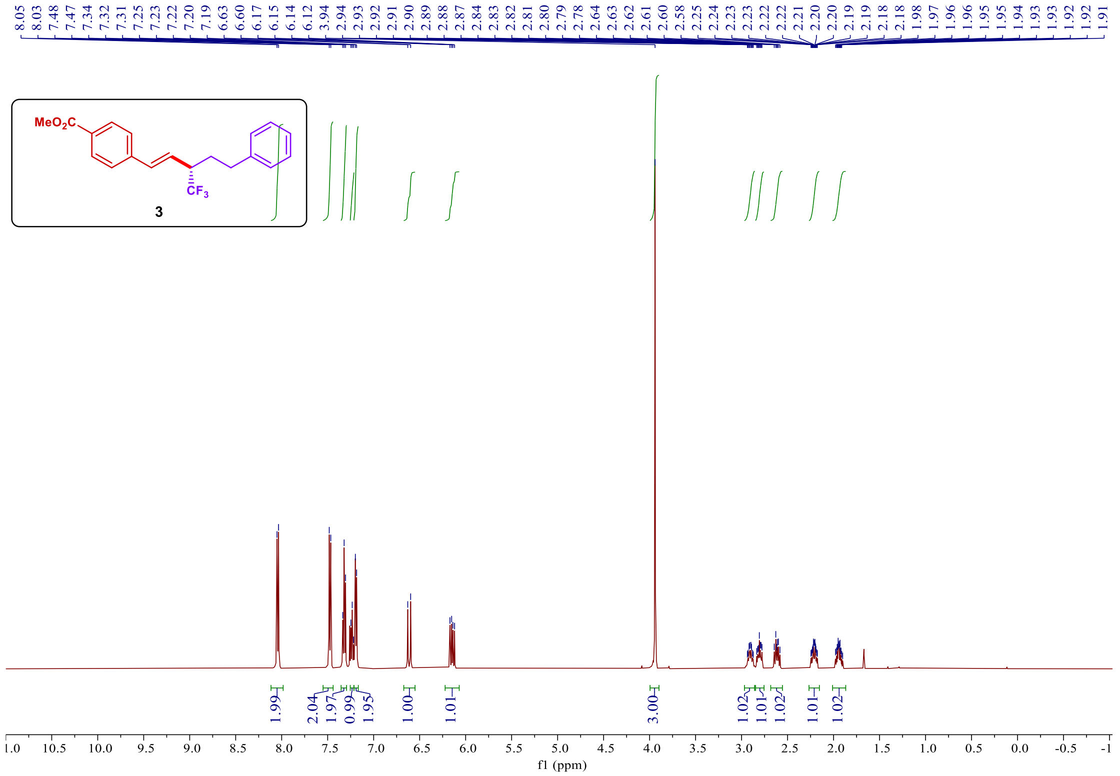

Supplementary Figure 26. ^{1}H NMR Spectrum of Compound **3** (500 MHz, $CDCl_3$)

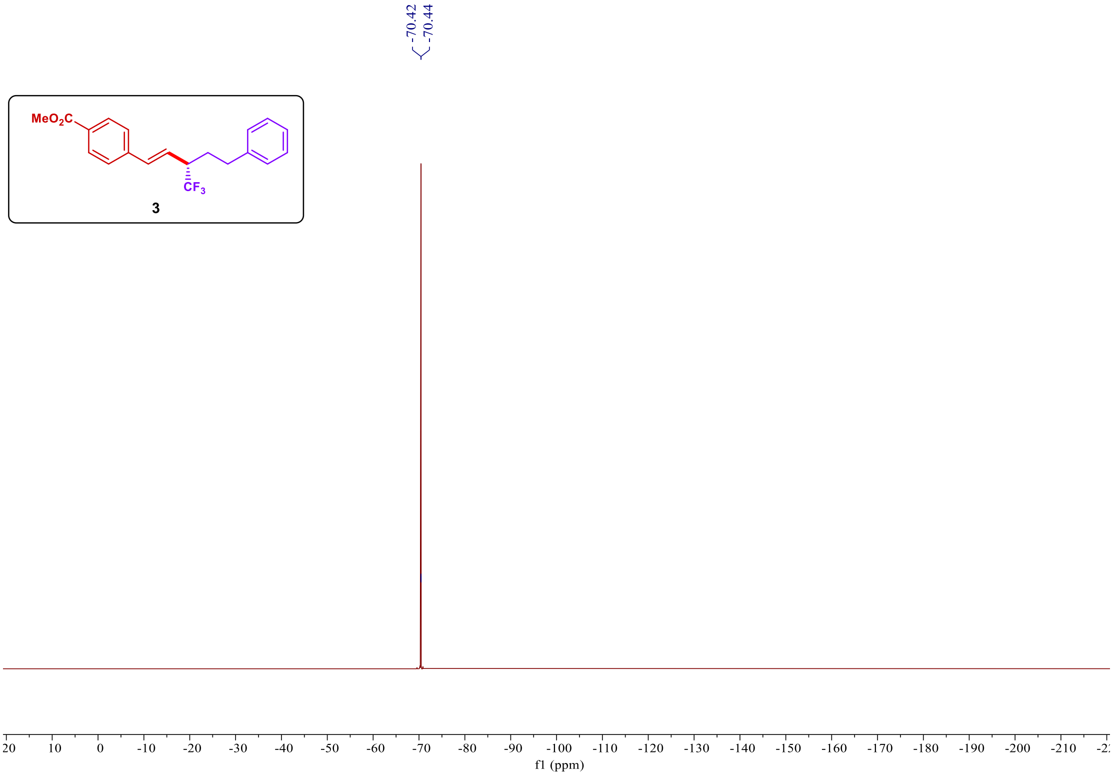

Supplementary Figure 27. ^{19}F NMR Spectrum of Compound **3** (471 MHz, $CDCl_3$)

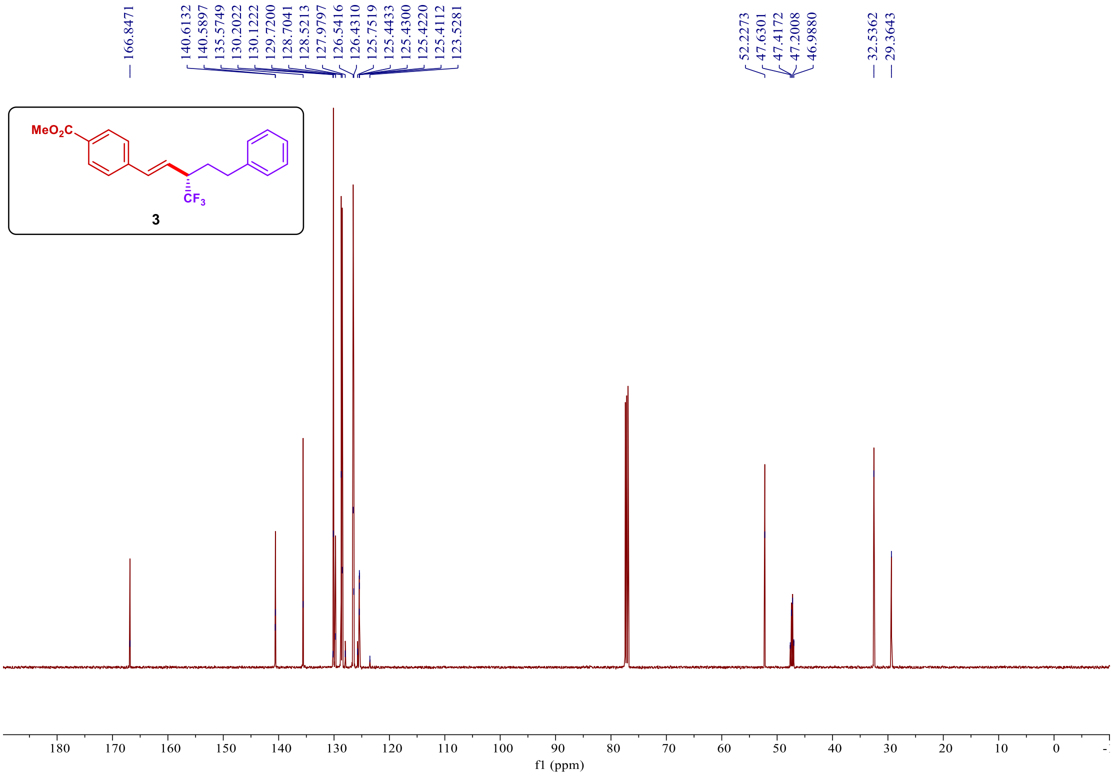

Supplementary Figure 28. ^{13}C NMR Spectrum of Compound **3** (126 MHz, $CDCl_3$)

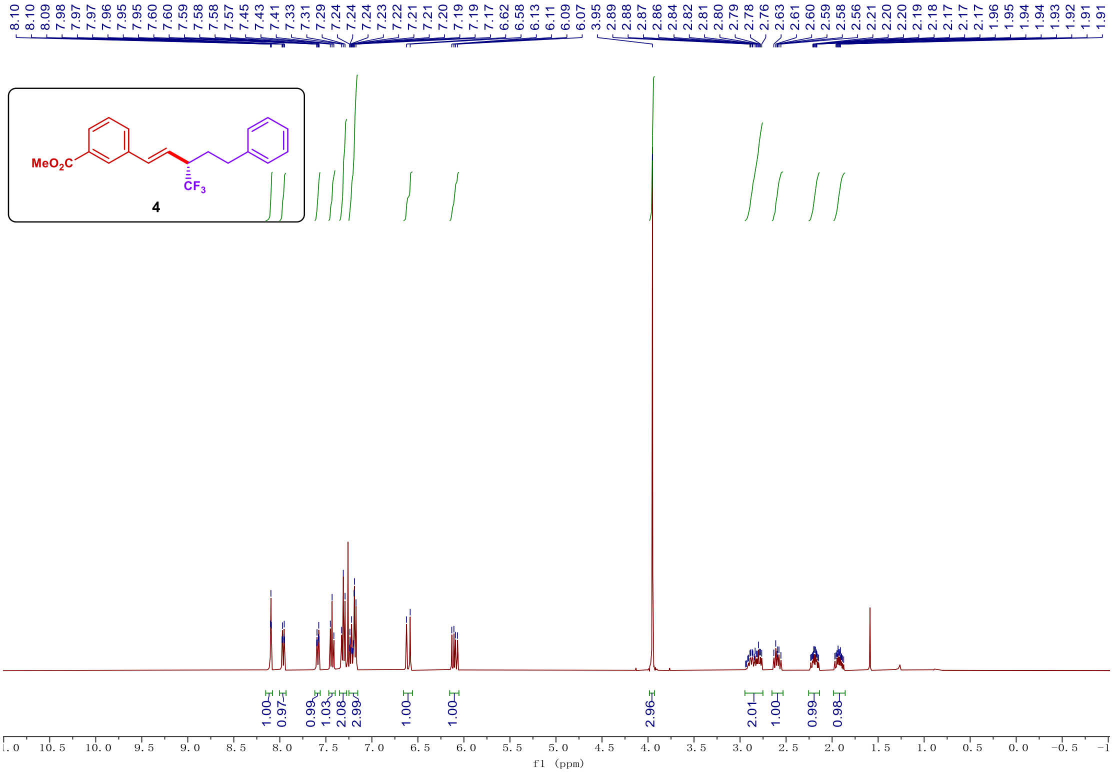

Supplementary Figure 29. ^{1}H NMR Spectrum of Compound **4** (400 MHz, $CDCl_3$)

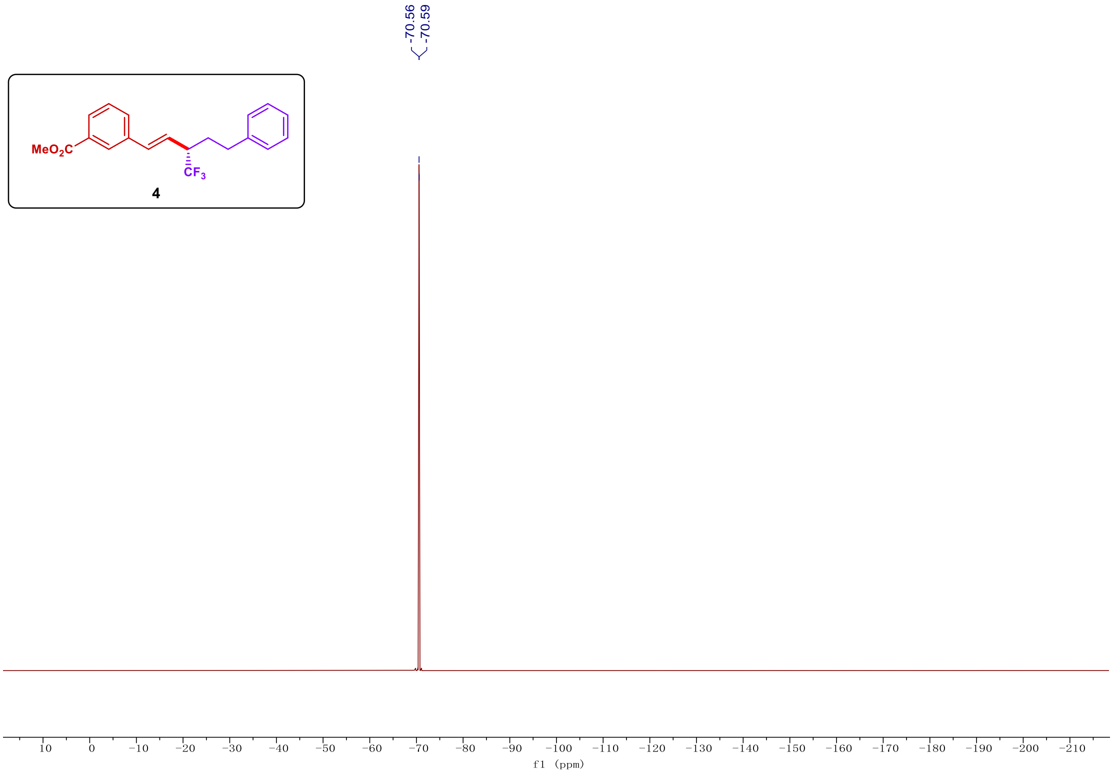

Supplementary Figure 30. ^{19}F NMR Spectrum of Compound **4** (376 MHz, $CDCl_3$)

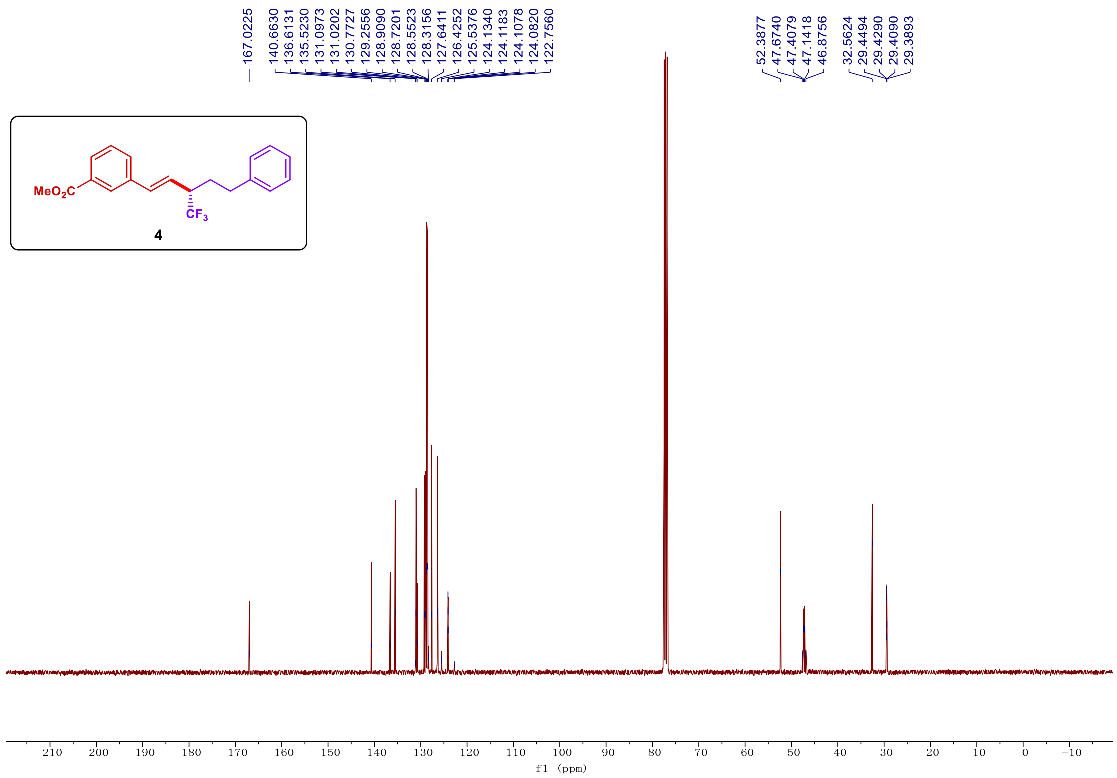

Supplementary Figure 31. ^{13}C NMR Spectrum of Compound **4** (101 MHz, $CDCl_3$)

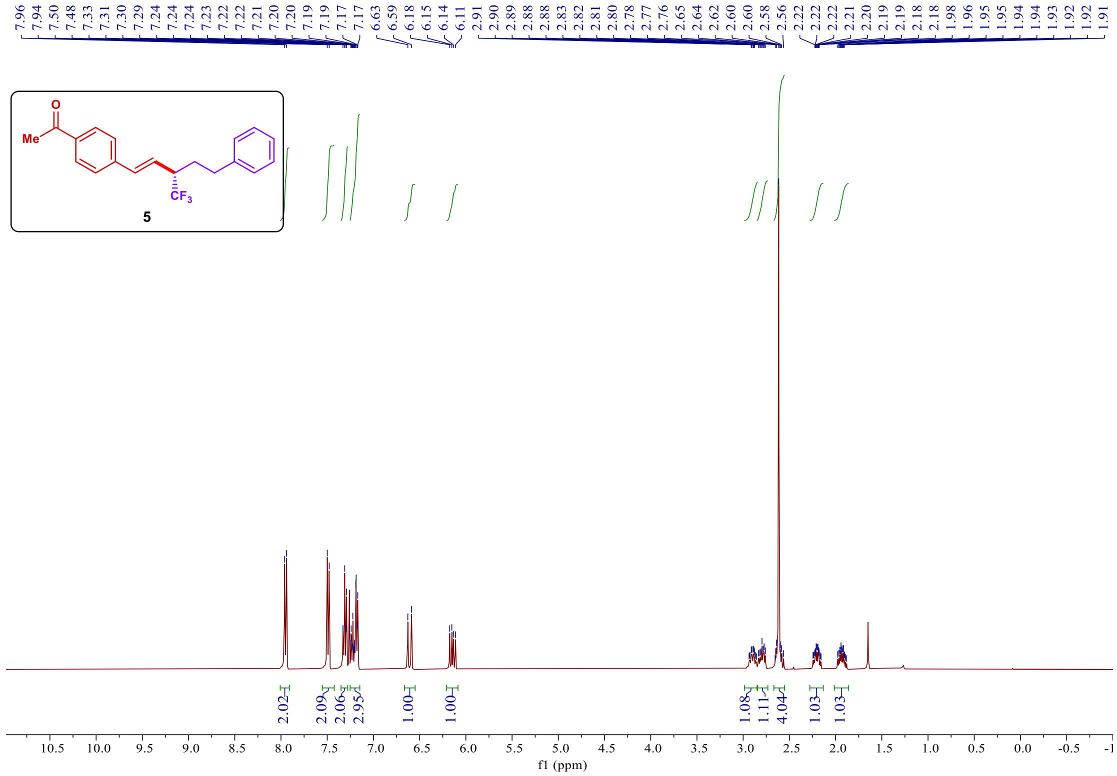

Supplementary Figure 32. ^{1}H NMR Spectrum of Compound **5** (400 MHz, $CDCl_3$)

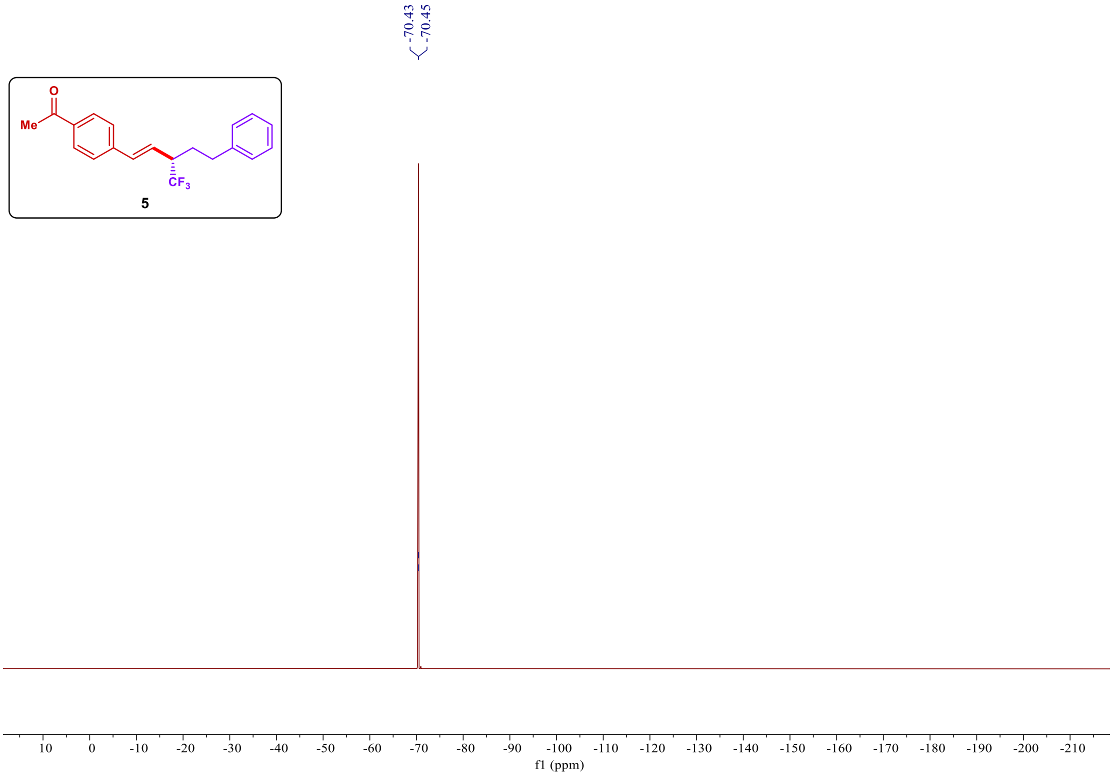

Supplementary Figure 33. ^{19}F NMR Spectrum of Compound **5** (376 MHz, $CDCl_3$)

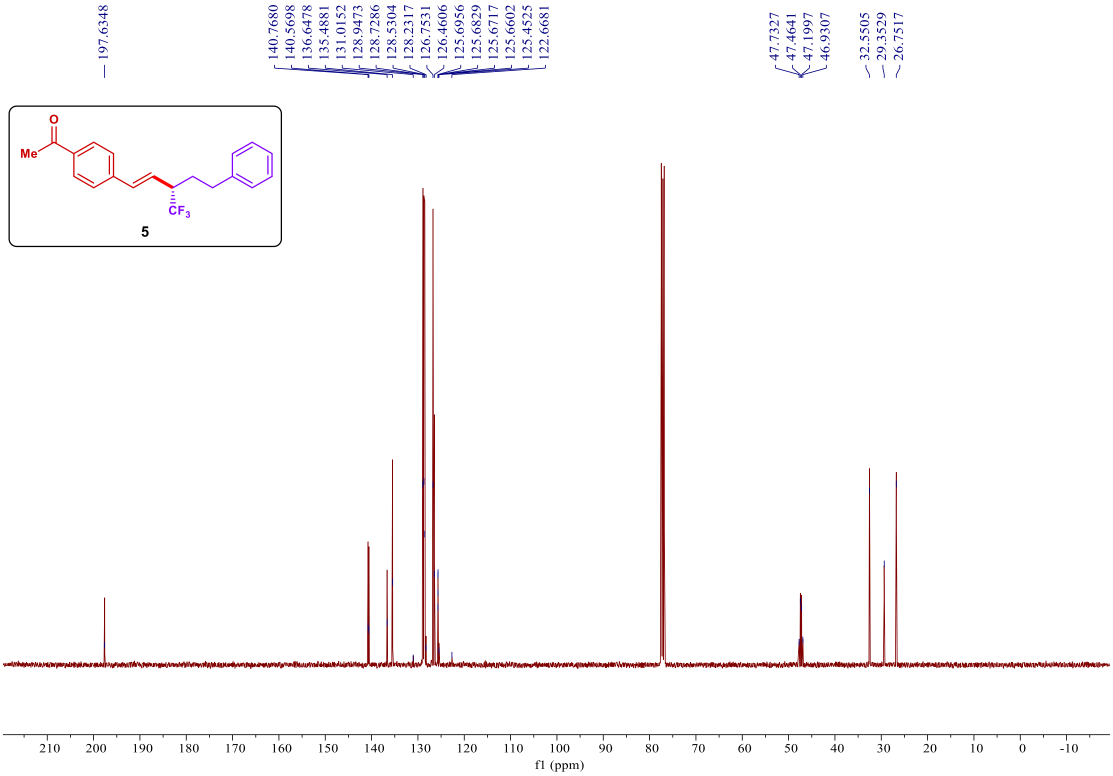

Supplementary Figure 34. ^{13}C NMR Spectrum of Compound **5** (101 MHz, $CDCl_3$)

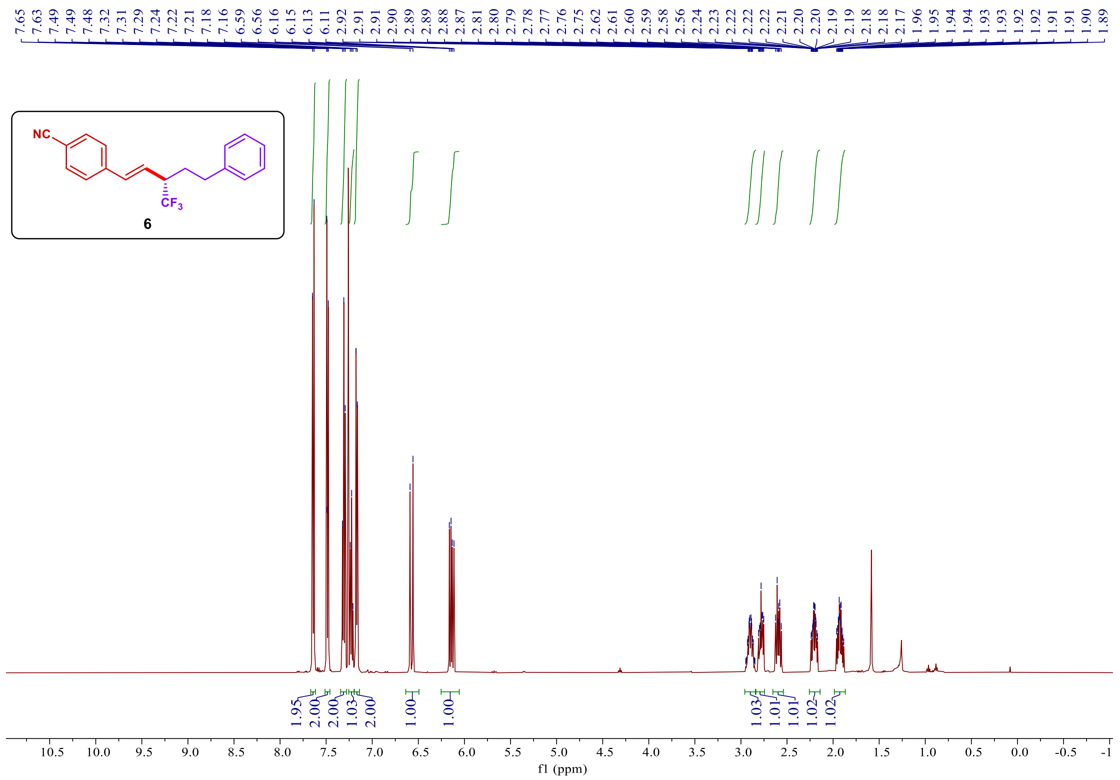

Supplementary Figure 35. ^{1}H NMR Spectrum of Compound **6** (500 MHz, $CDCl_3$)

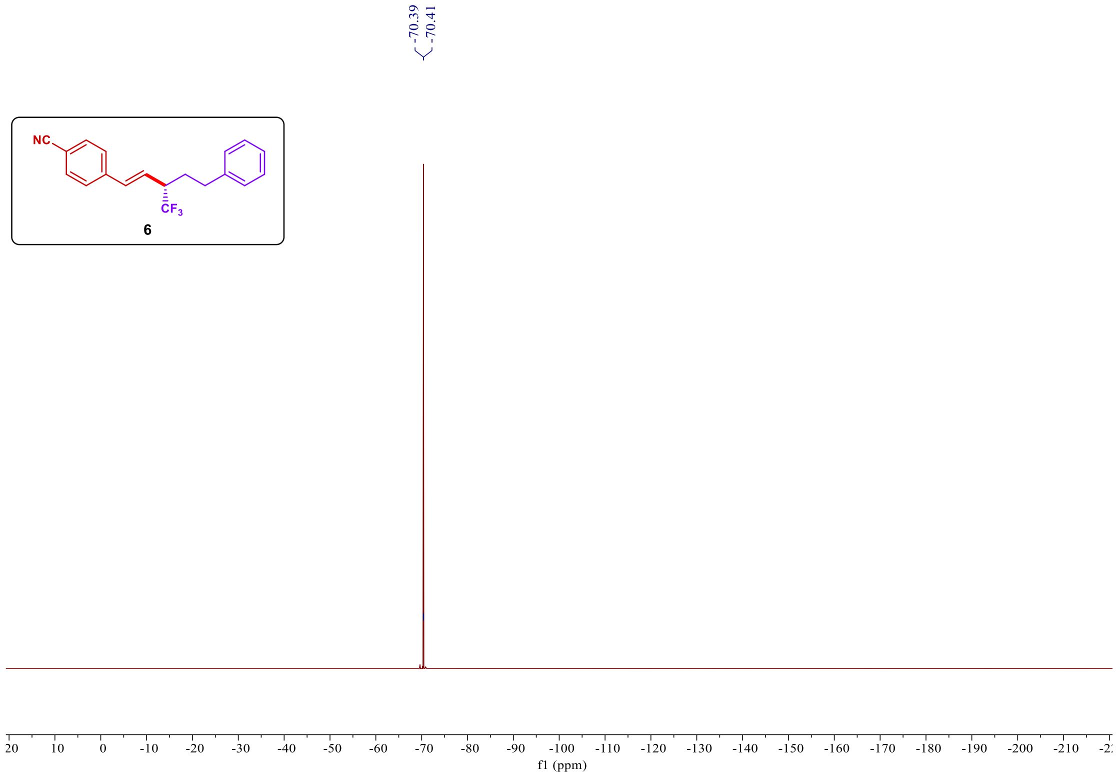

Supplementary Figure 36. ^{19}F NMR Spectrum of Compound **6** (471 MHz, $CDCl_3$)

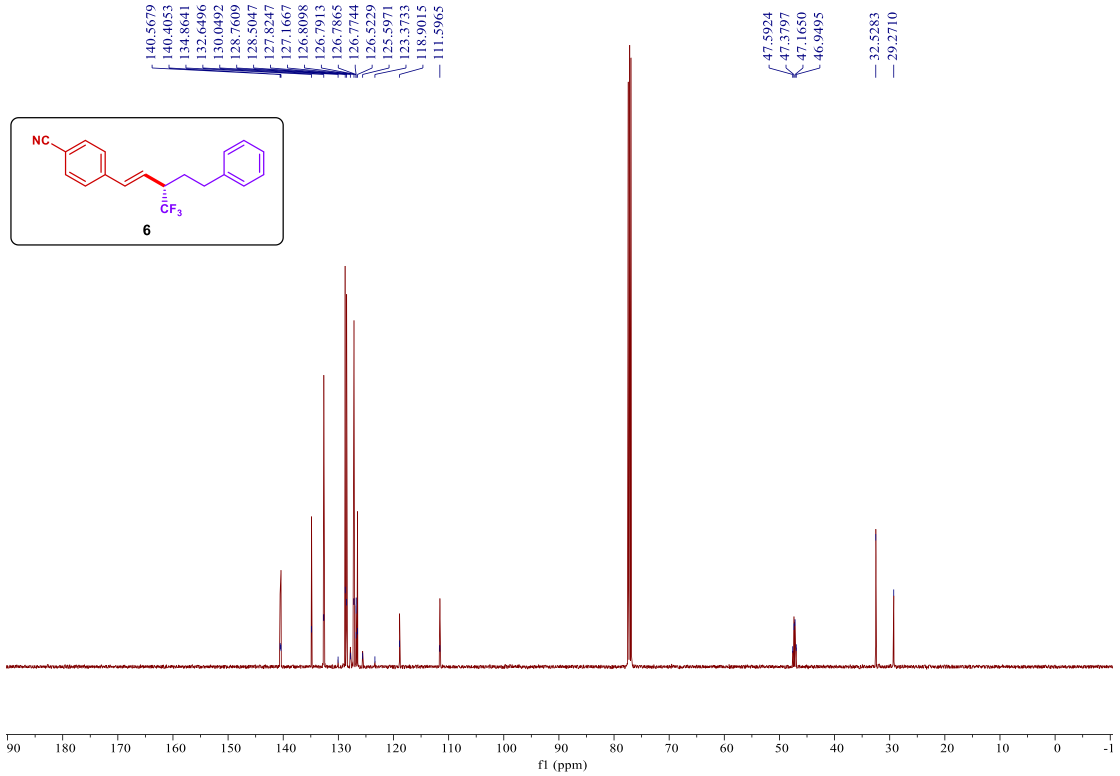

Supplementary Figure 37. ^{13}C NMR Spectrum of Compound **6** (126 MHz, $CDCl_3$)

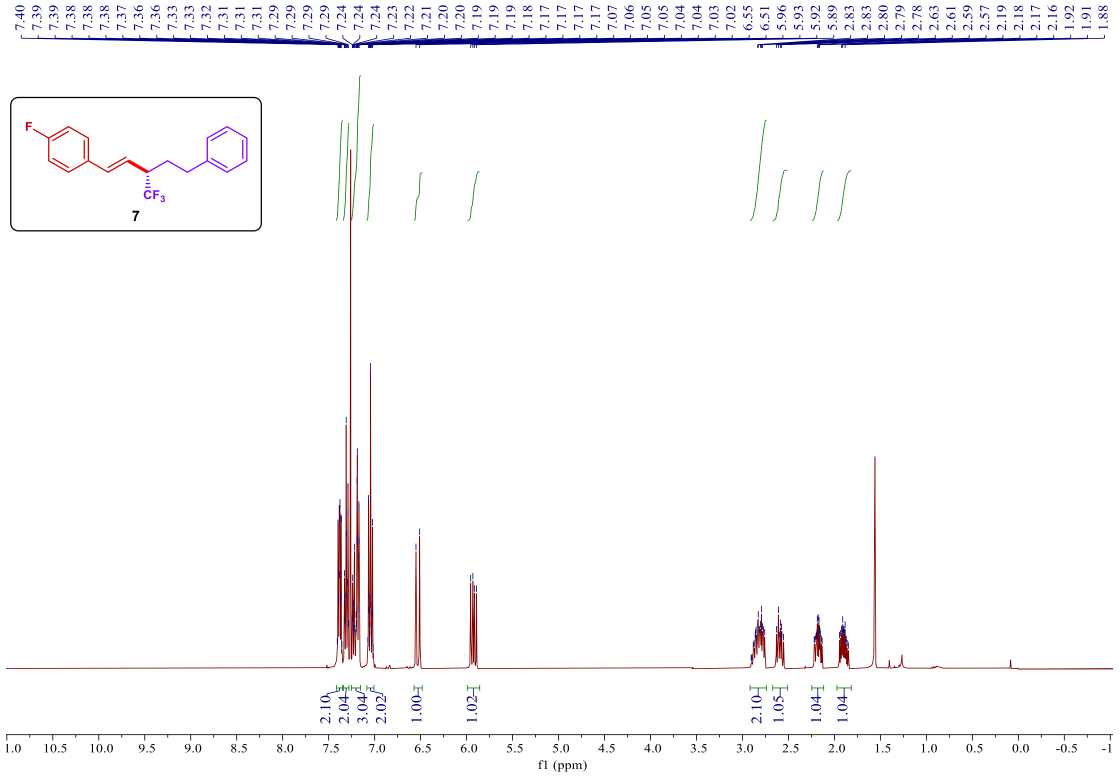

Supplementary Figure 38. ^{1}H NMR Spectrum of Compound **7** (400 MHz, $CDCl_3$)

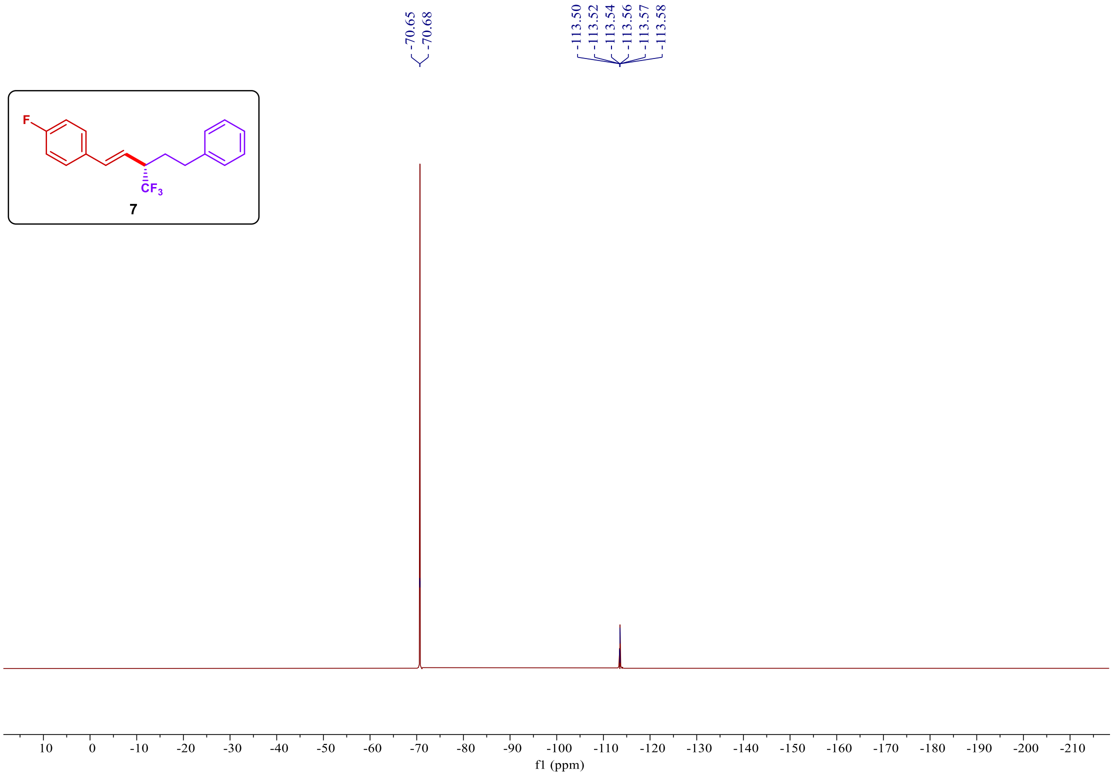

Supplementary Figure 39. ^{19}F NMR Spectrum of Compound **7** (376 MHz, $CDCl_3$)

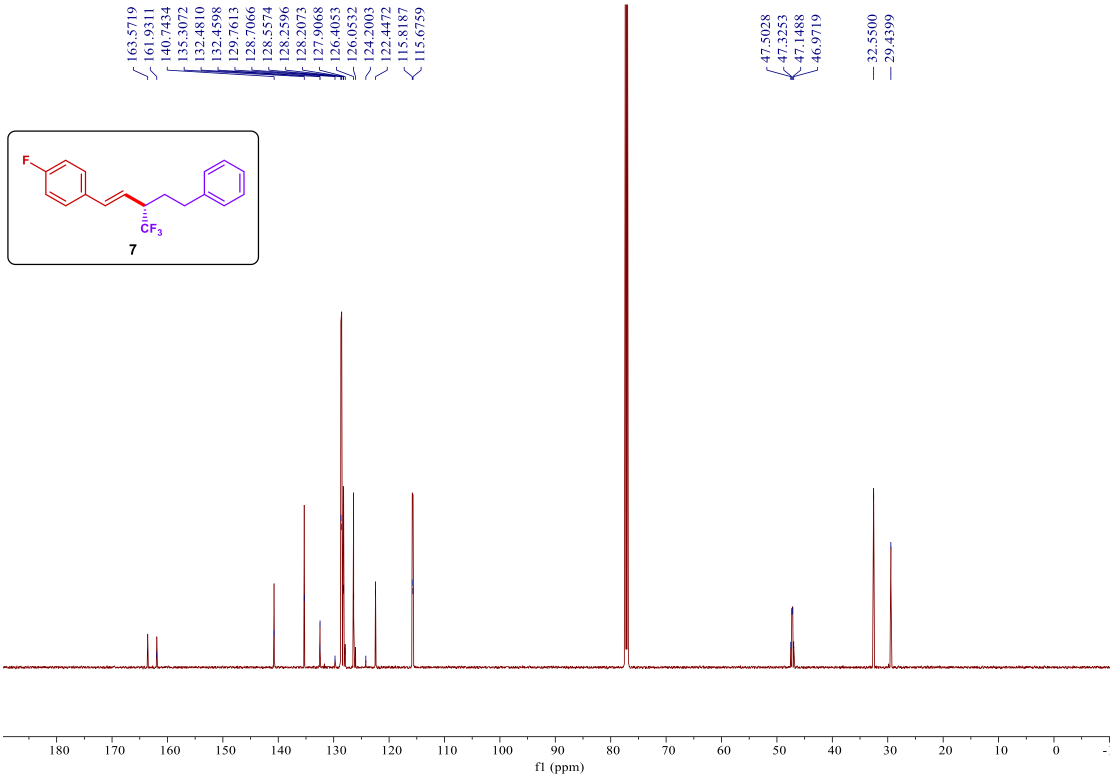

Supplementary Figure 40. ^{13}C NMR Spectrum of Compound **7** (151 MHz, $CDCl_3$)

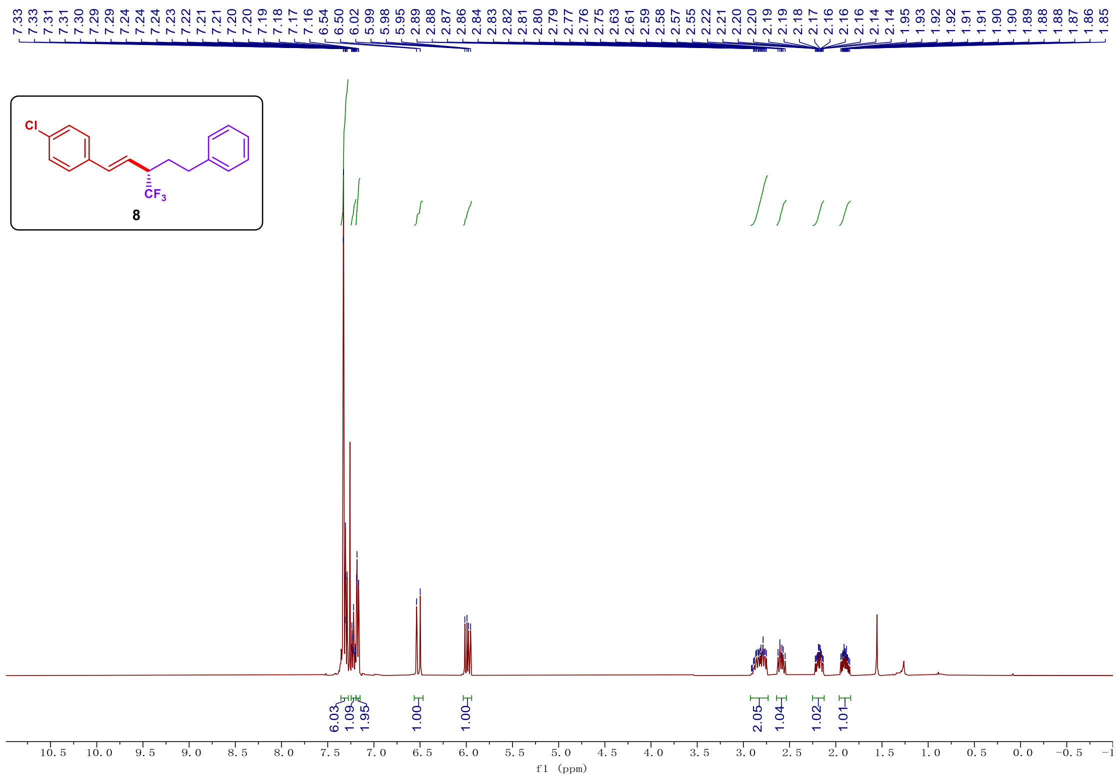

Supplementary Figure 41. ^{1}H NMR Spectrum of Compound **8** (400 MHz, $CDCl_3$)

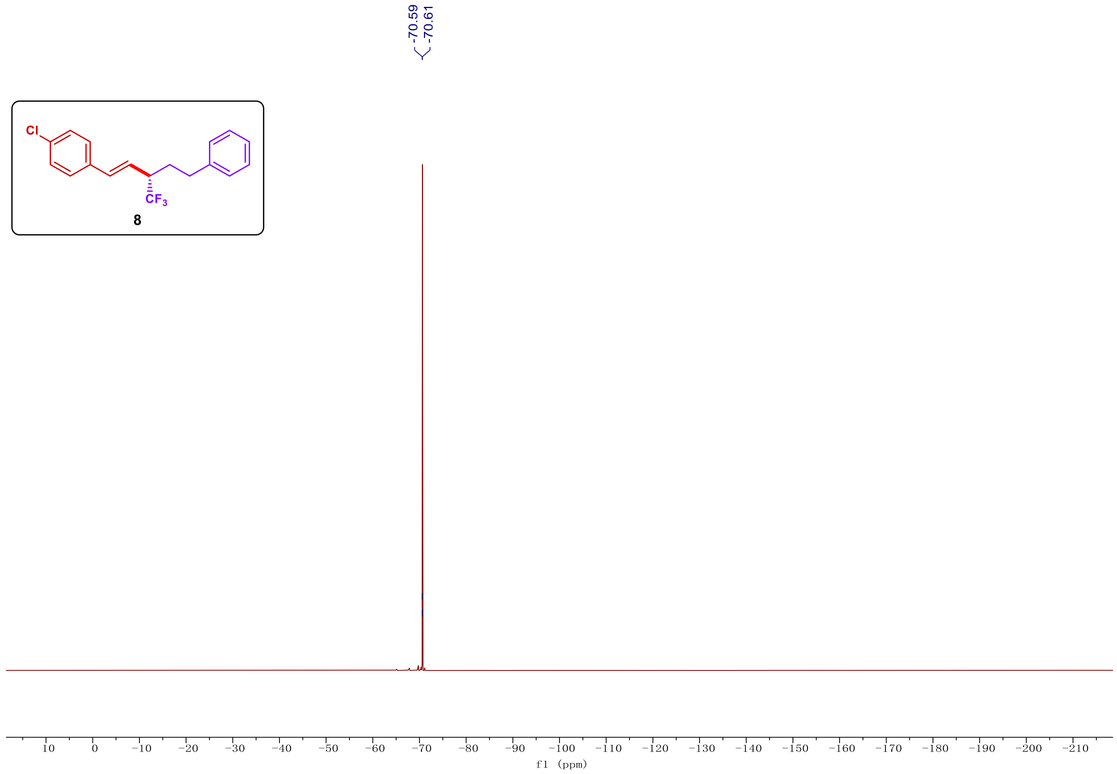

Supplementary Figure 42. ^{19}F NMR Spectrum of Compound **8** (376 MHz, $CDCl_3$)

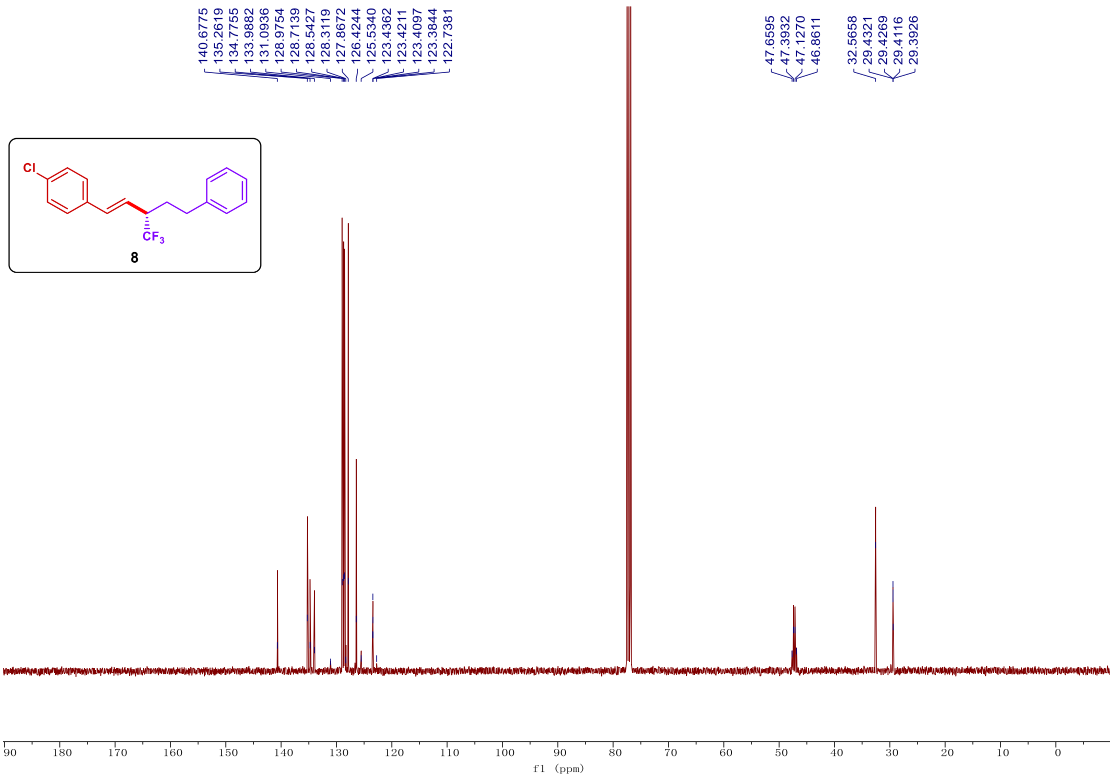

Supplementary Figure 43. ^{13}C NMR Spectrum of Compound **8** (101 MHz, $CDCl_3$)

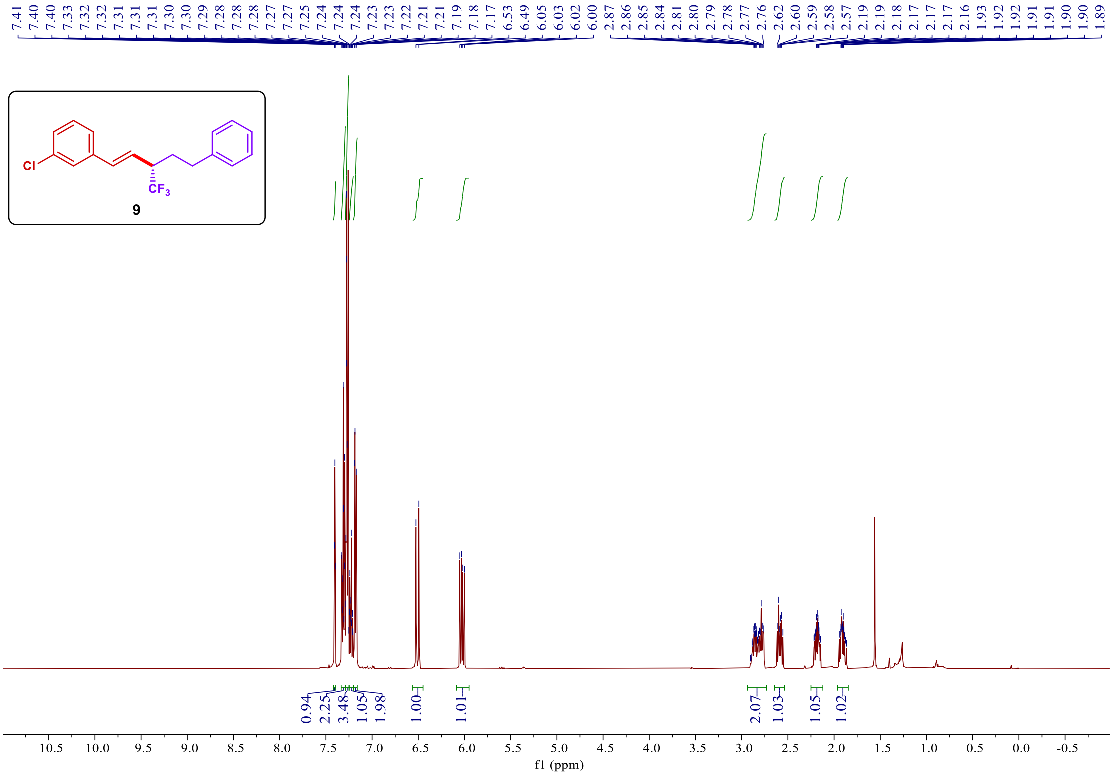

Supplementary Figure 44. ^{1}H NMR Spectrum of Compound **9** (500 MHz, $CDCl_3$)

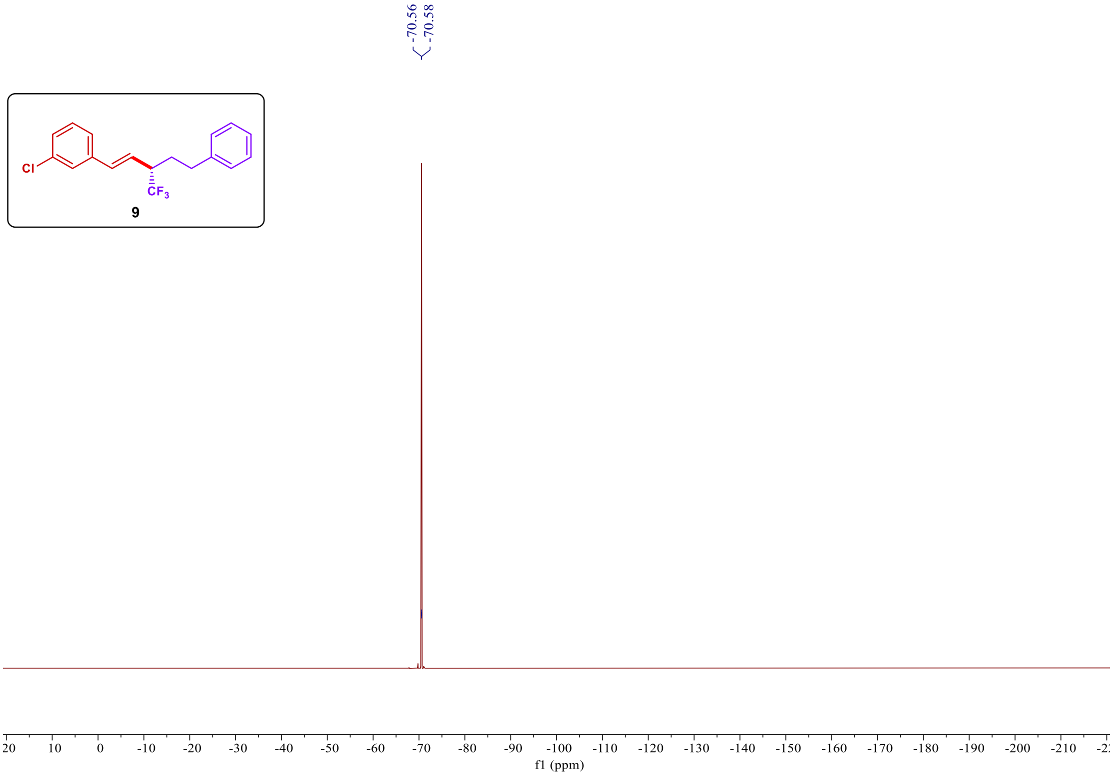

Supplementary Figure 45. ^{19}F NMR Spectrum of Compound **9** (471 MHz, $CDCl_3$)

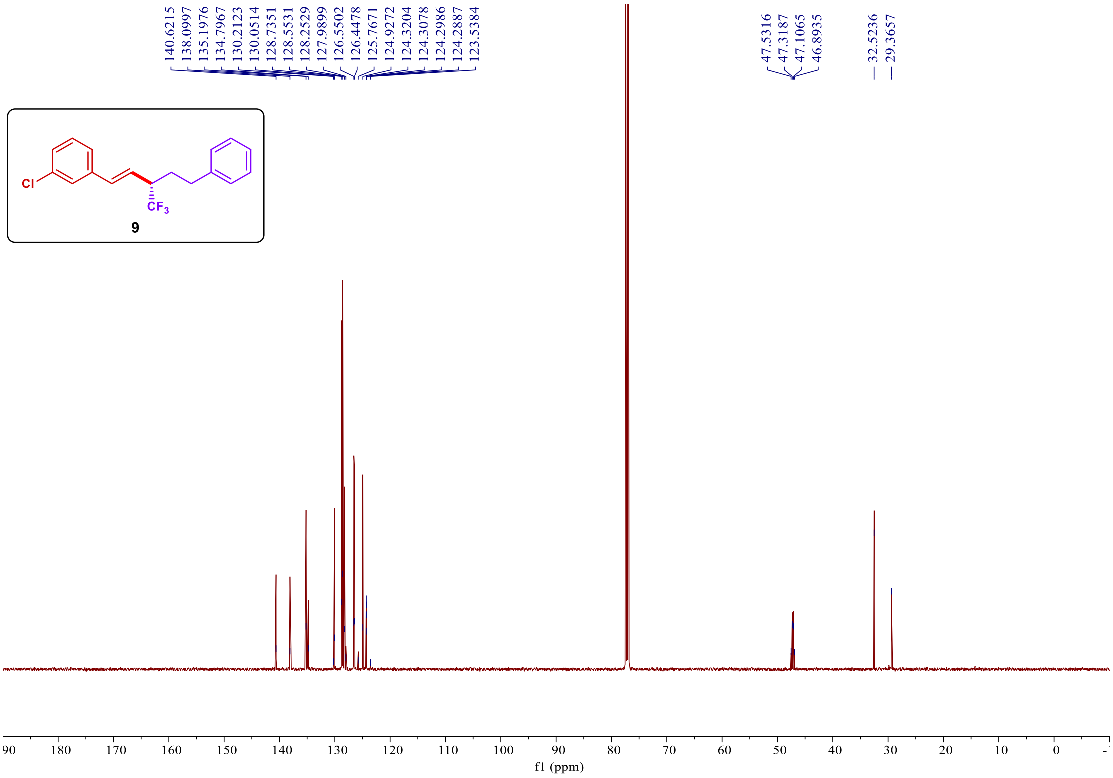

Supplementary Figure 46. ^{13}C NMR Spectrum of Compound **9** (126 MHz, $CDCl_3$)

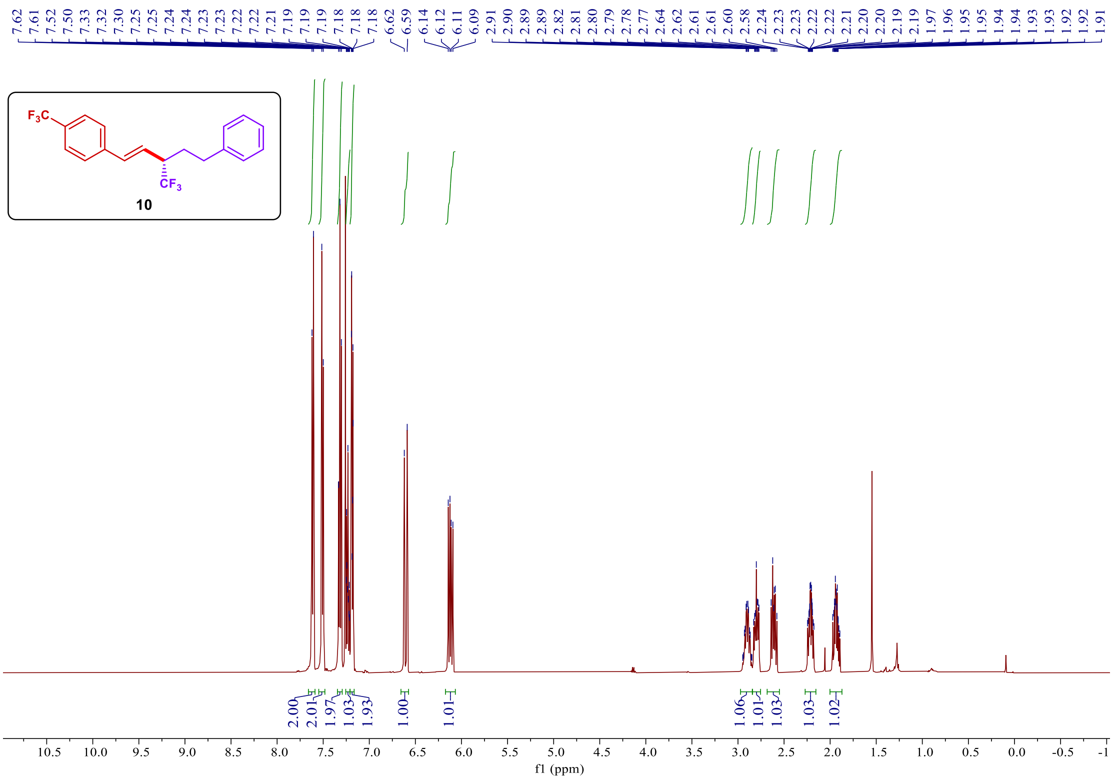

Supplementary Figure 47. ^{1}H NMR Spectrum of Compound **10** (500 MHz, $CDCl_3$)

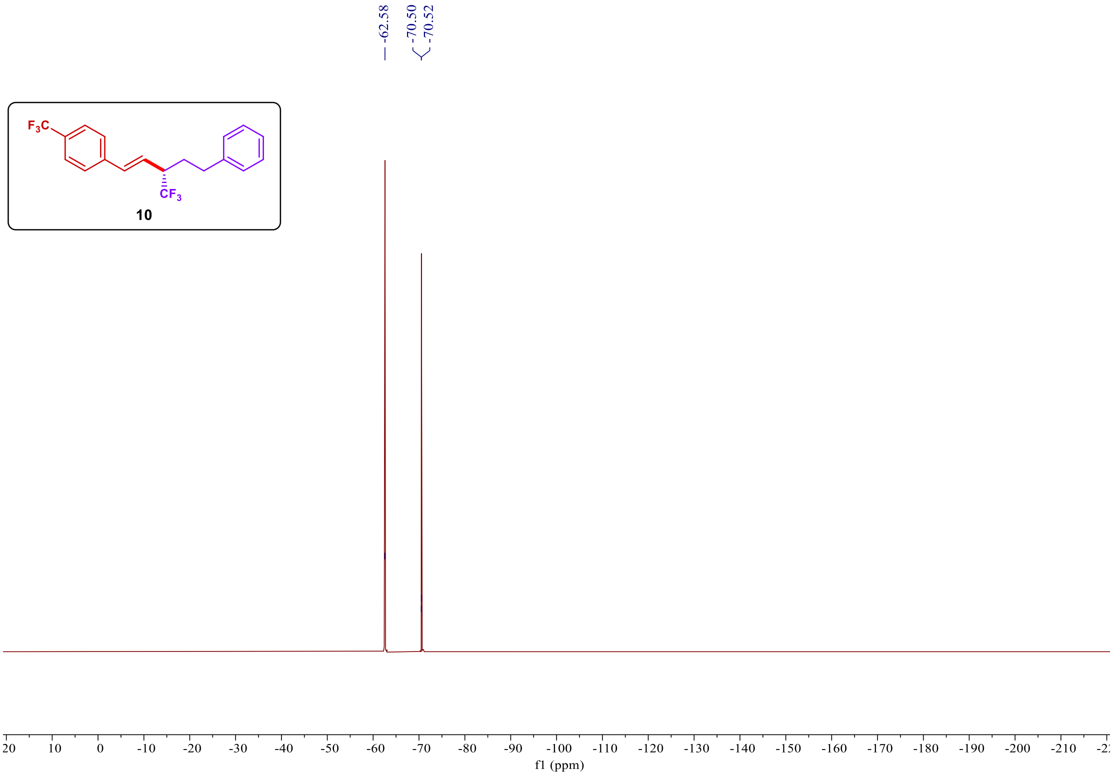

Supplementary Figure 48. ^{19}F NMR Spectrum of Compound **10** (471 MHz, $CDCl_3$)

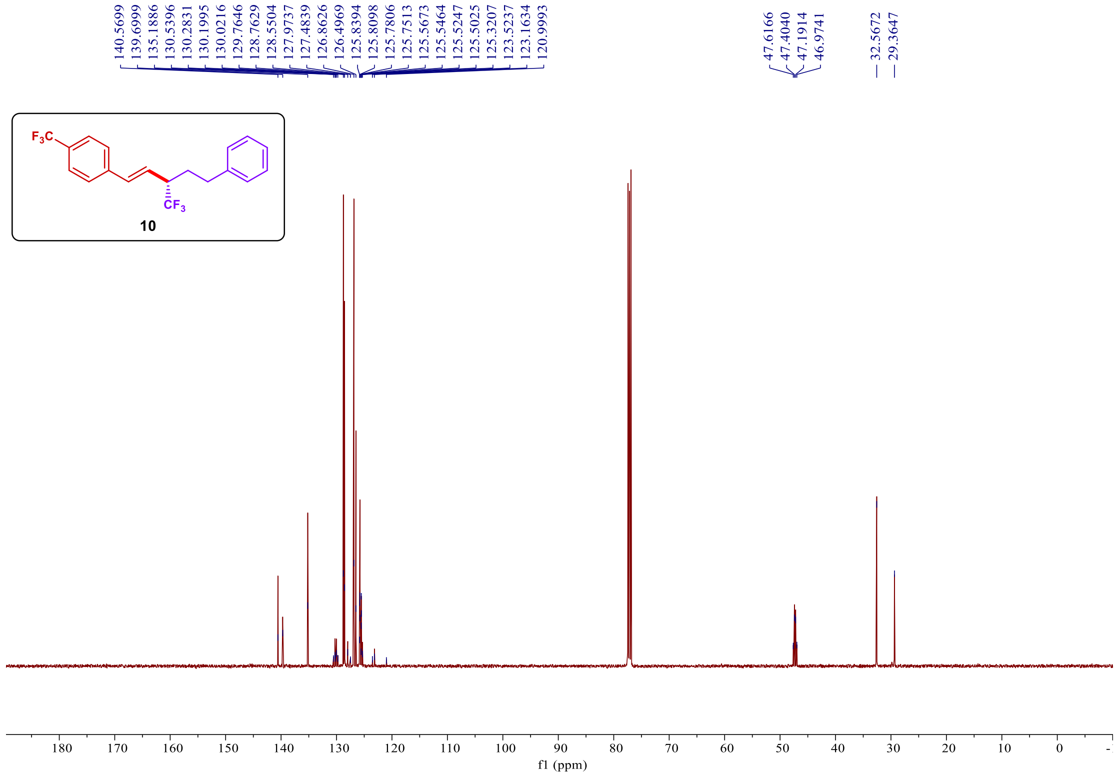

Supplementary Figure 49. ^{13}C NMR Spectrum of Compound **10** (126 MHz, $CDCl_3$)

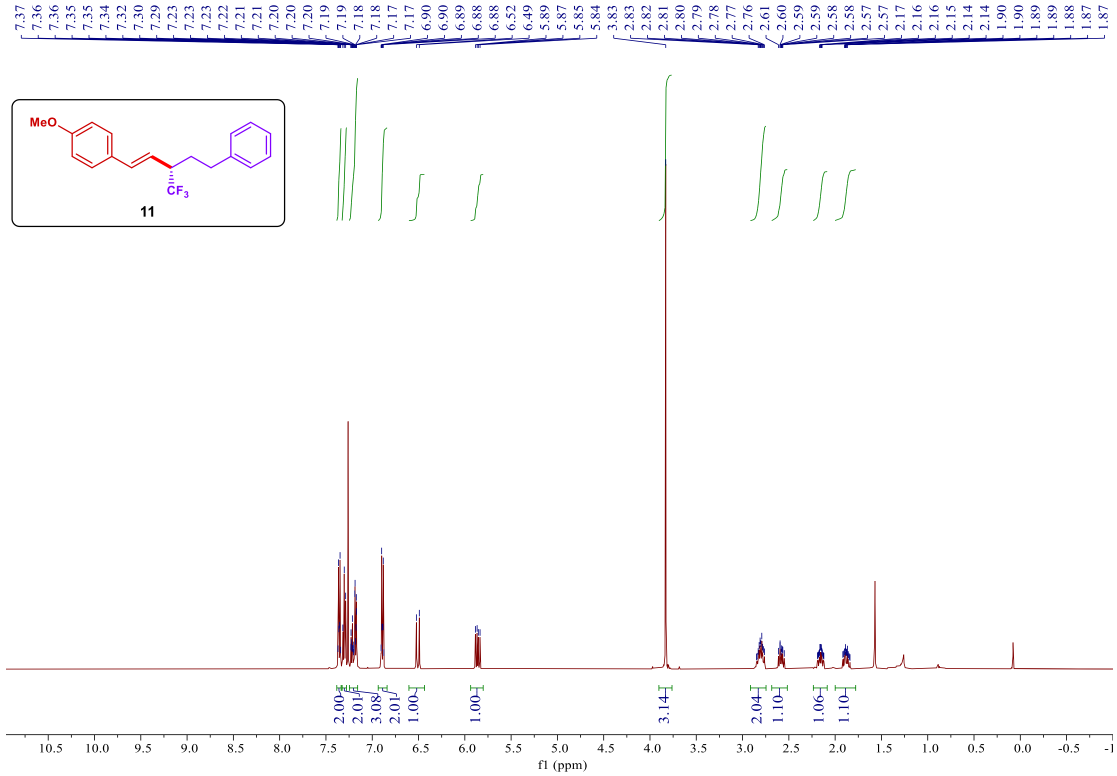

Supplementary Figure 50. ^{1}H NMR Spectrum of Compound **11** (500 MHz, $CDCl_3$)

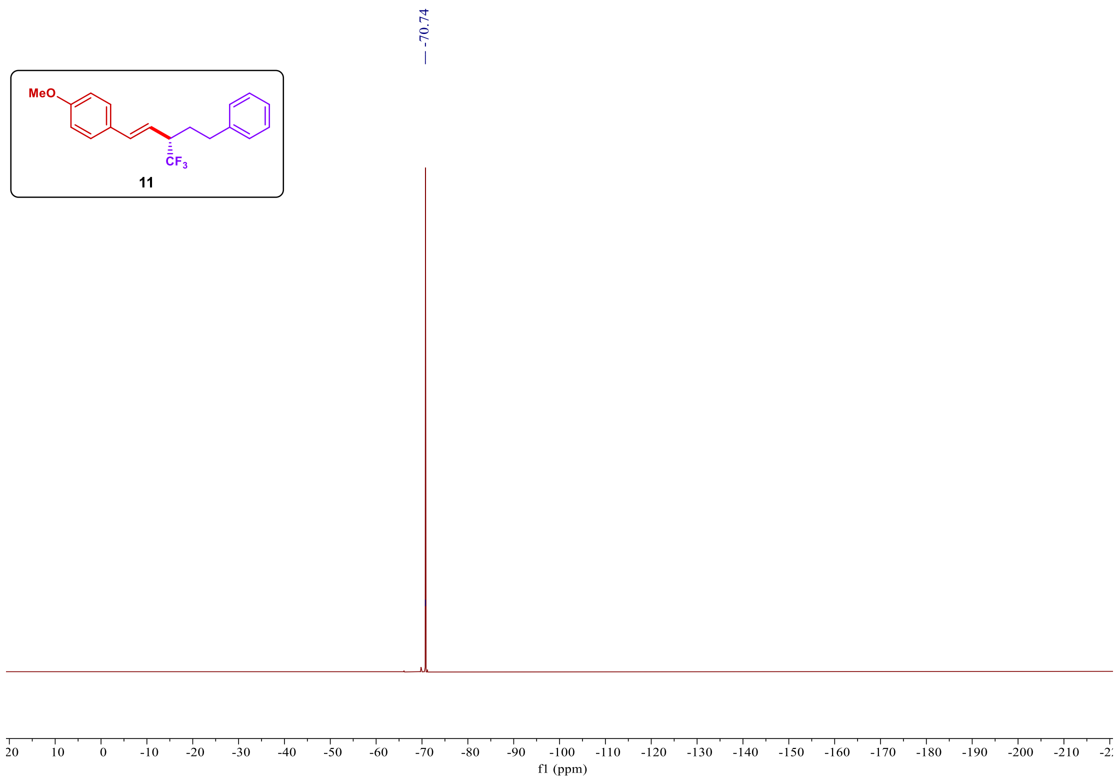

Supplementary Figure 51. ^{19}F NMR Spectrum of Compound **11** (471 MHz, $CDCl_3$)

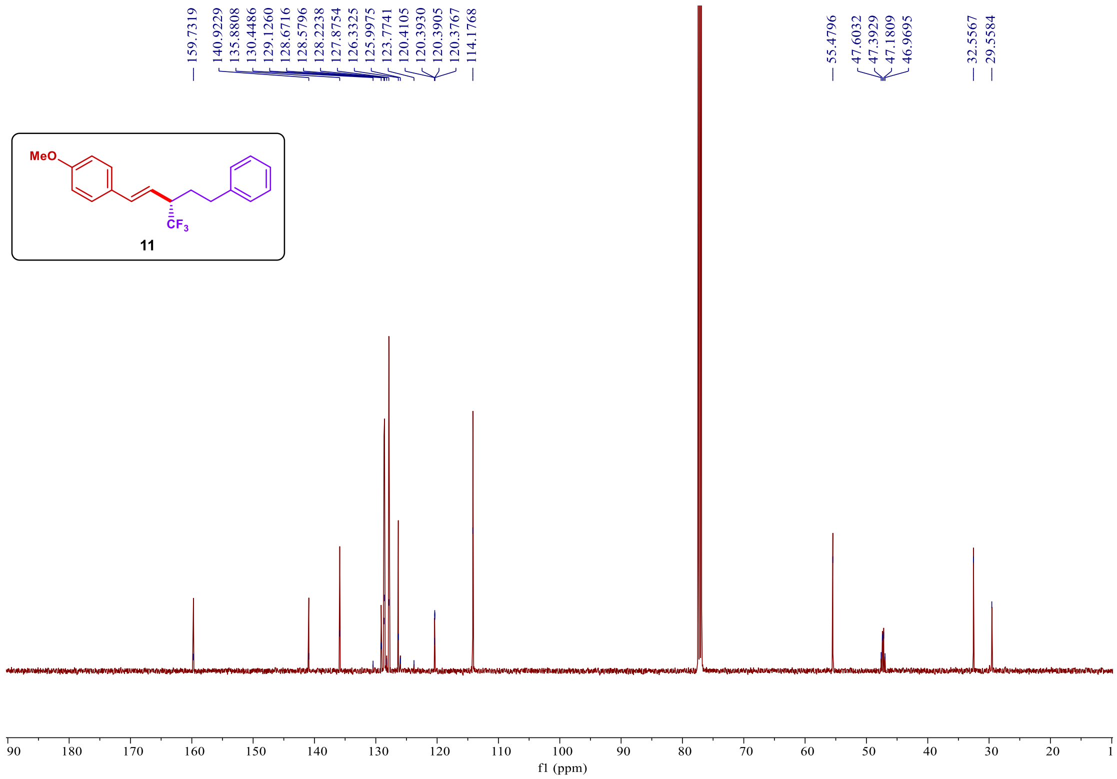

Supplementary Figure 52. ^{13}C NMR Spectrum of Compound **11** (126 MHz, $CDCl_3$)

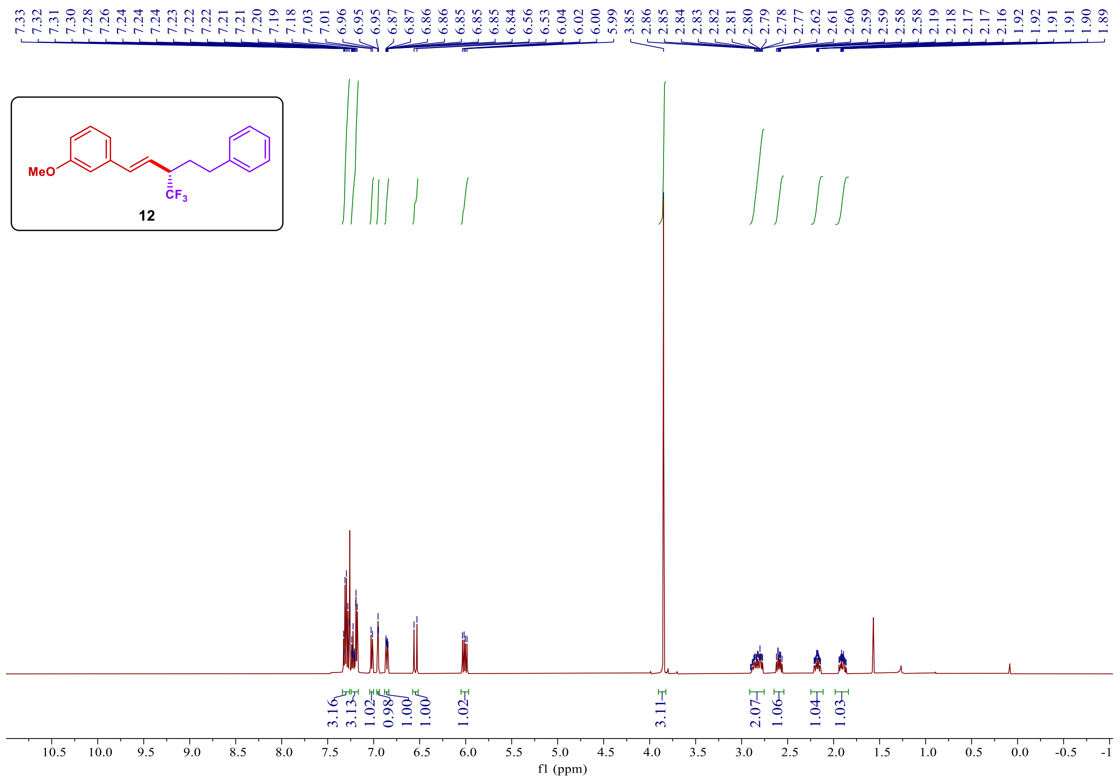

Supplementary Figure 53. ^{1}H NMR Spectrum of Compound **12** (500 MHz, $CDCl_3$)

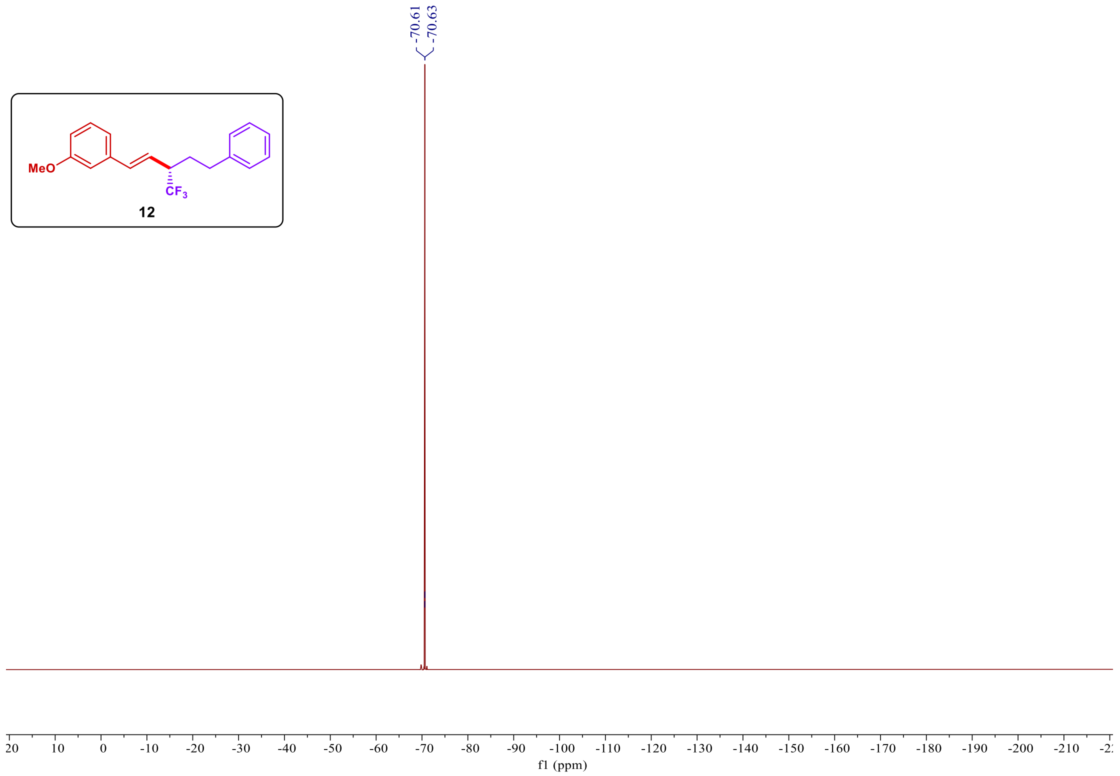

Supplementary Figure 54. ^{19}F NMR Spectrum of Compound **12** (471 MHz, $CDCl_3$)

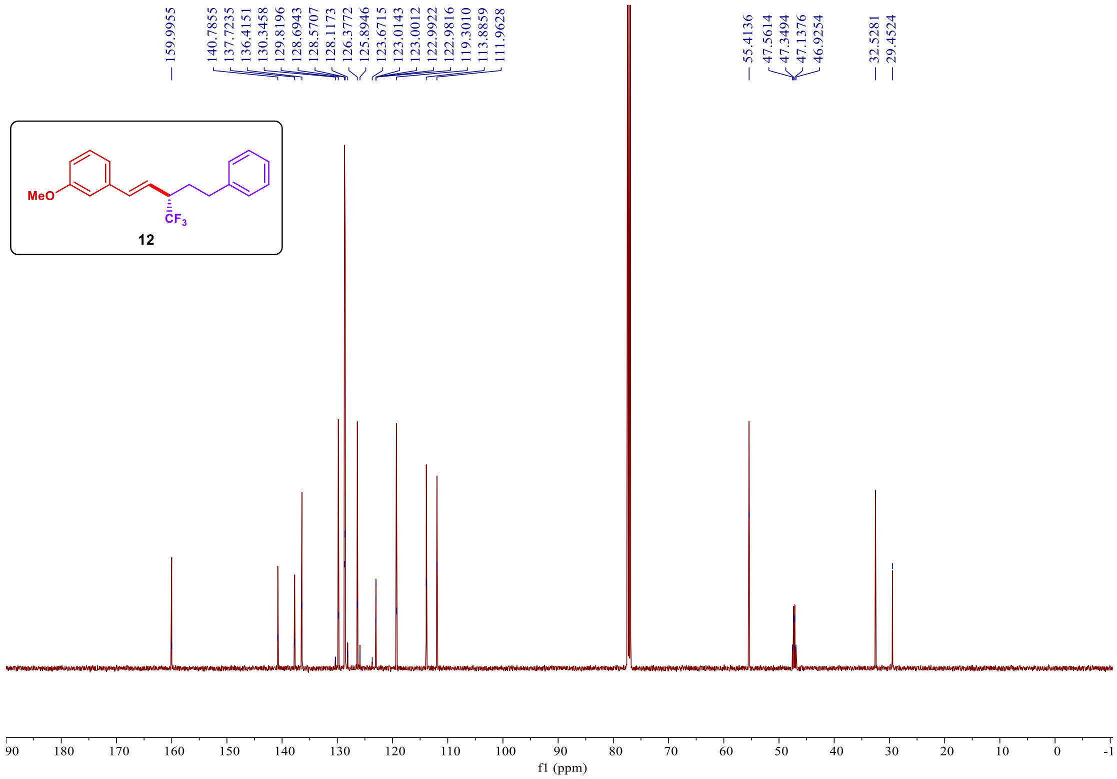

Supplementary Figure 55. ^{13}C NMR Spectrum of Compound **12** (126 MHz, $CDCl_3$)

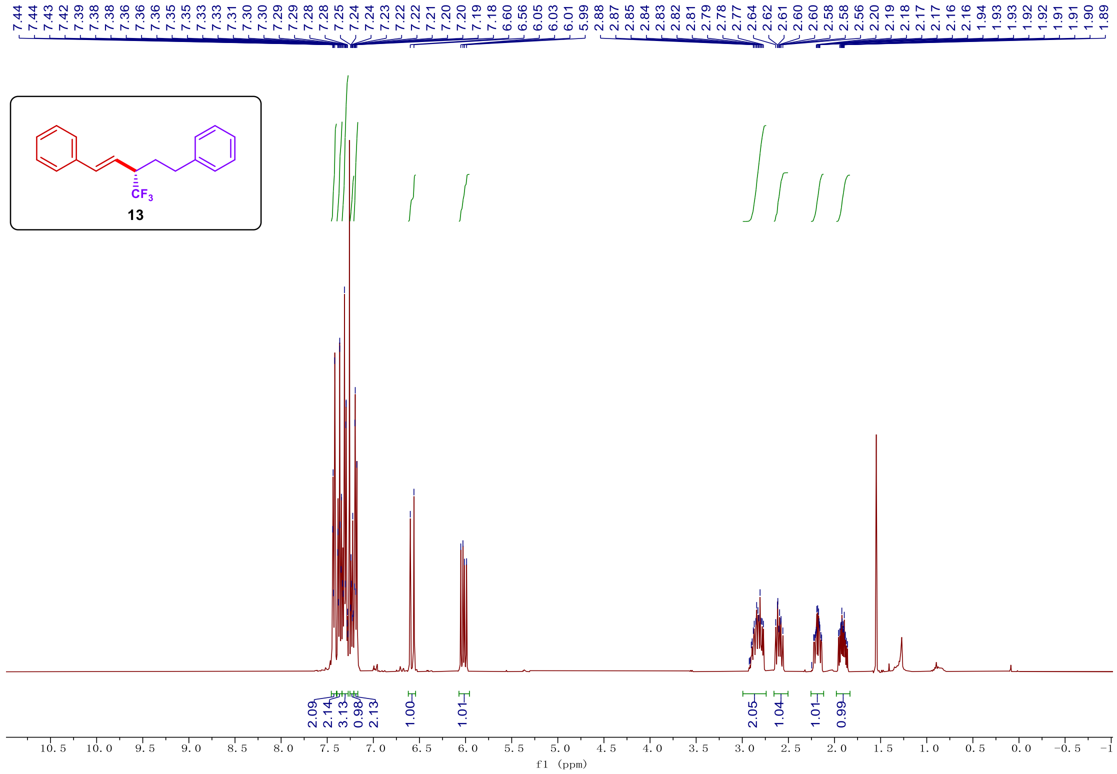

Supplementary Figure 56. ^{1}H NMR Spectrum of Compound **13** (400 MHz, $CDCl_3$)

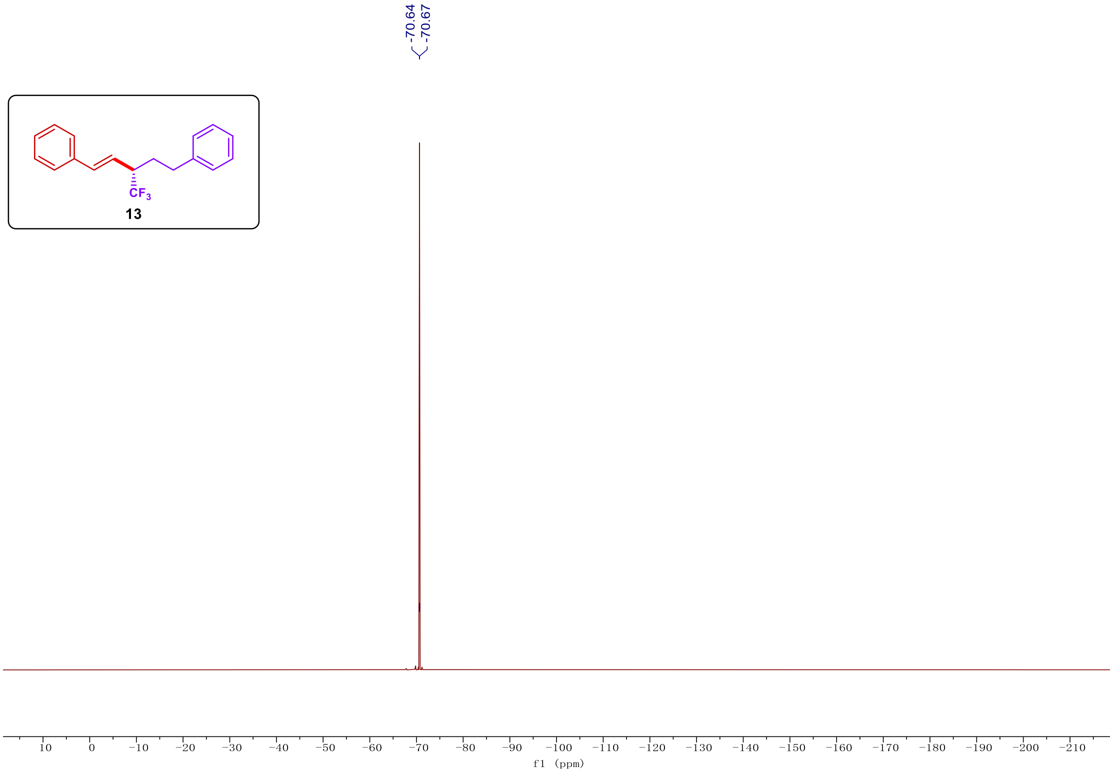

Supplementary Figure 57. ^{19}F NMR Spectrum of Compound **13** (376 MHz, $CDCl_3$)

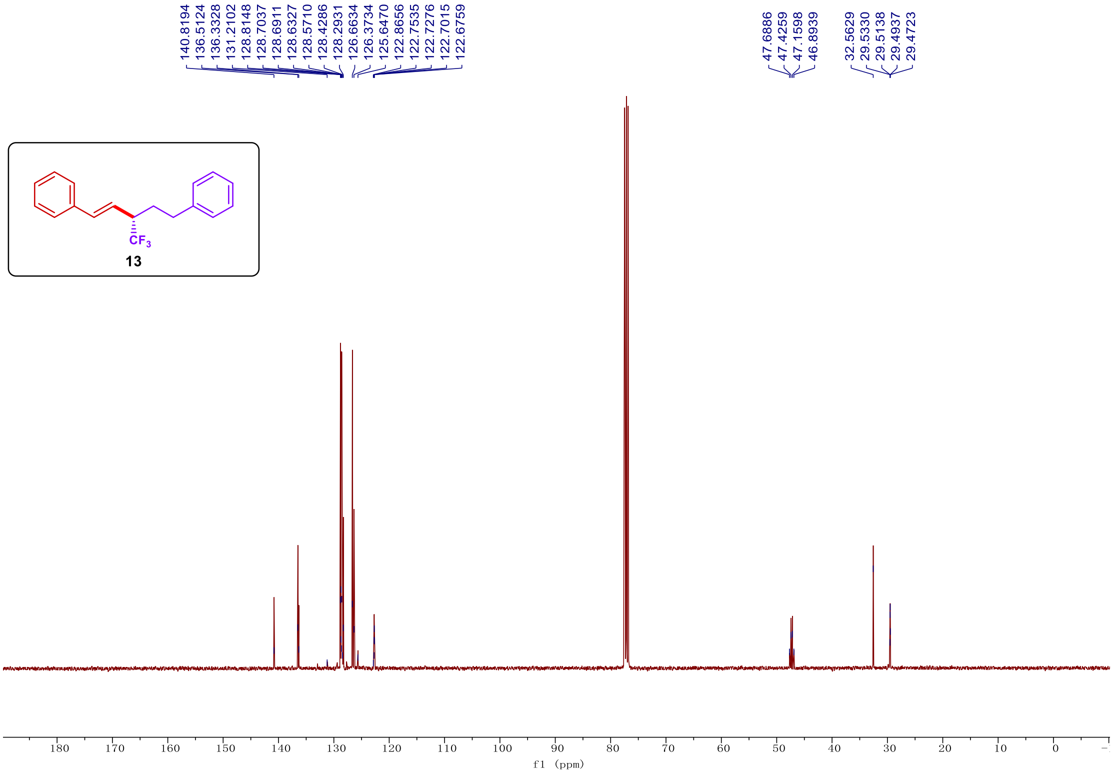

Supplementary Figure 58. ^{13}C NMR Spectrum of Compound **13** (101 MHz, $CDCl_3$)

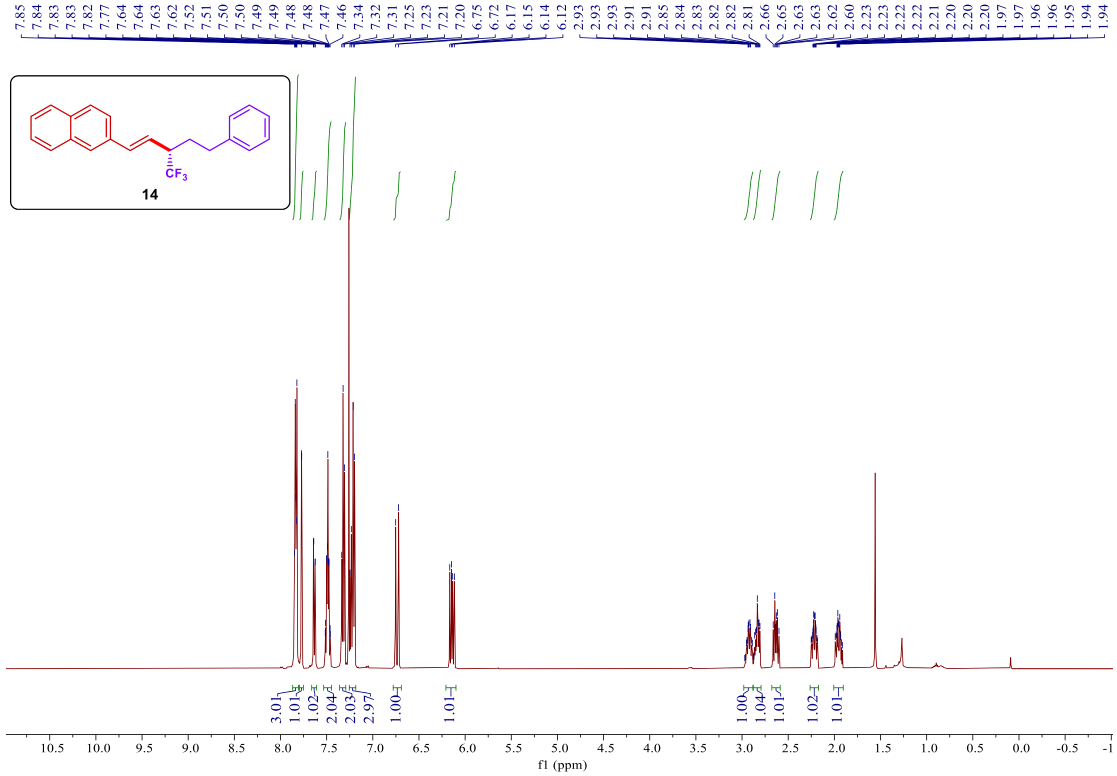

Supplementary Figure 59. ^{1}H NMR Spectrum of Compound **14** (500 MHz, $CDCl_3$)

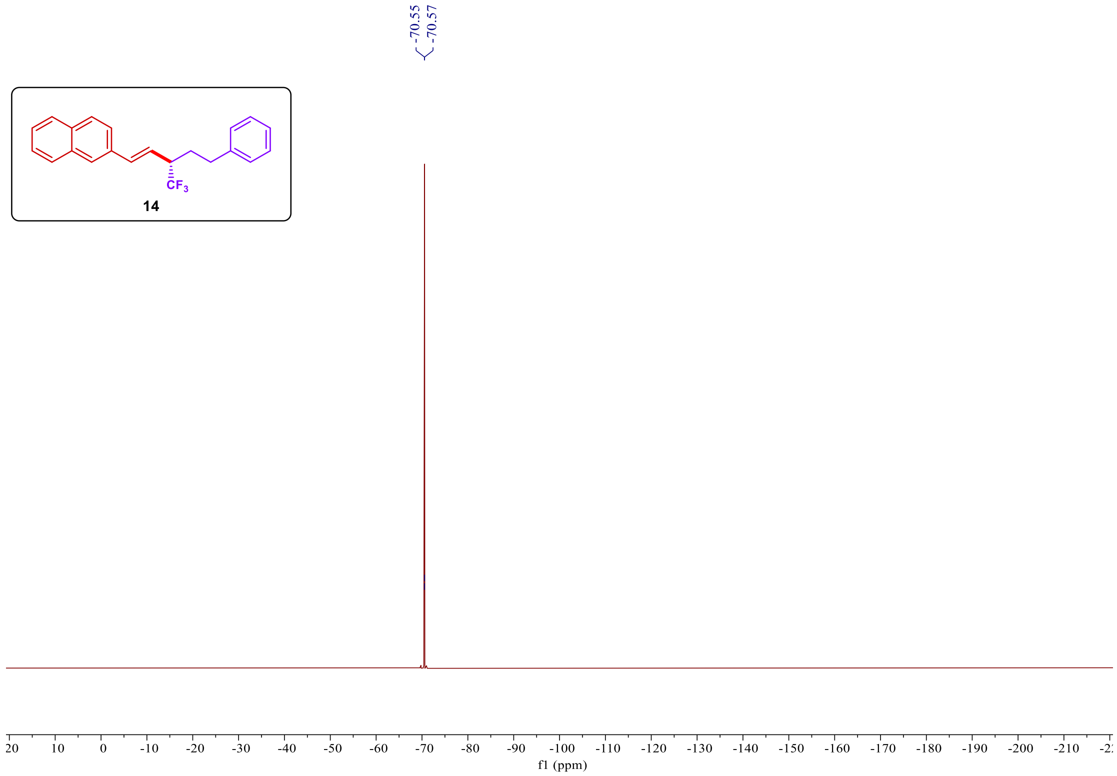

Supplementary Figure 60. ^{19}F NMR Spectrum of Compound **14** (471 MHz, $CDCl_3$)

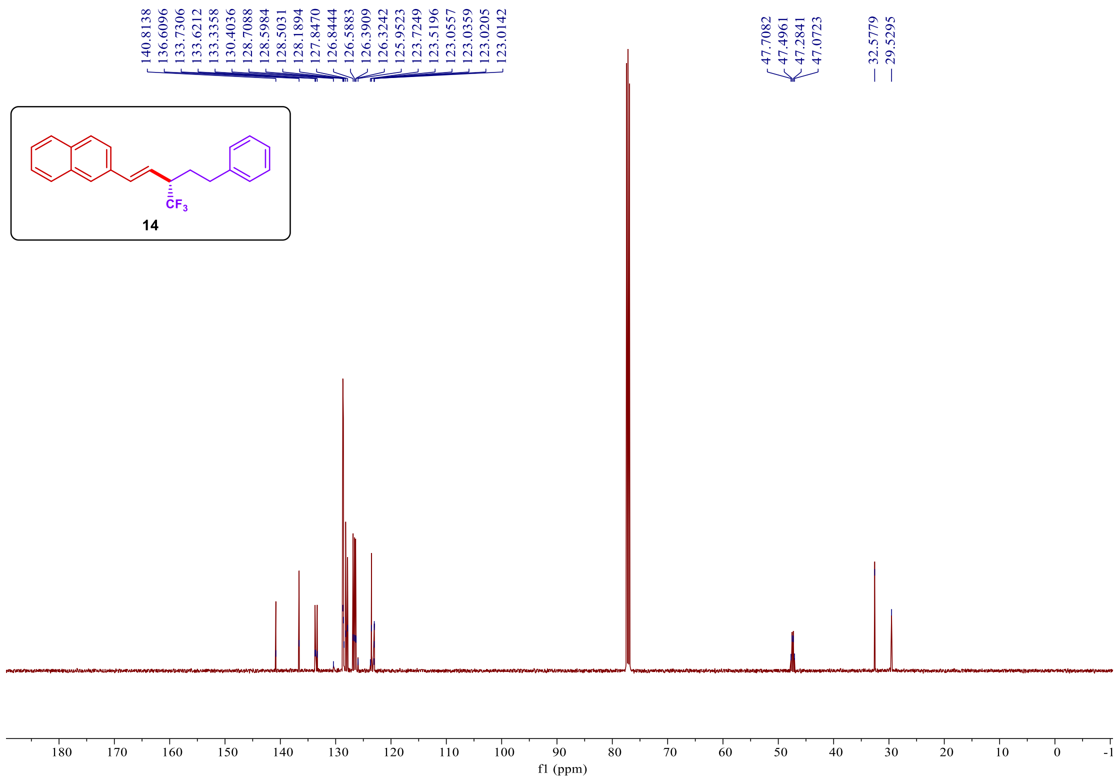

Supplementary Figure 61. ^{13}C NMR Spectrum of Compound **14** (126 MHz, $CDCl_3$)

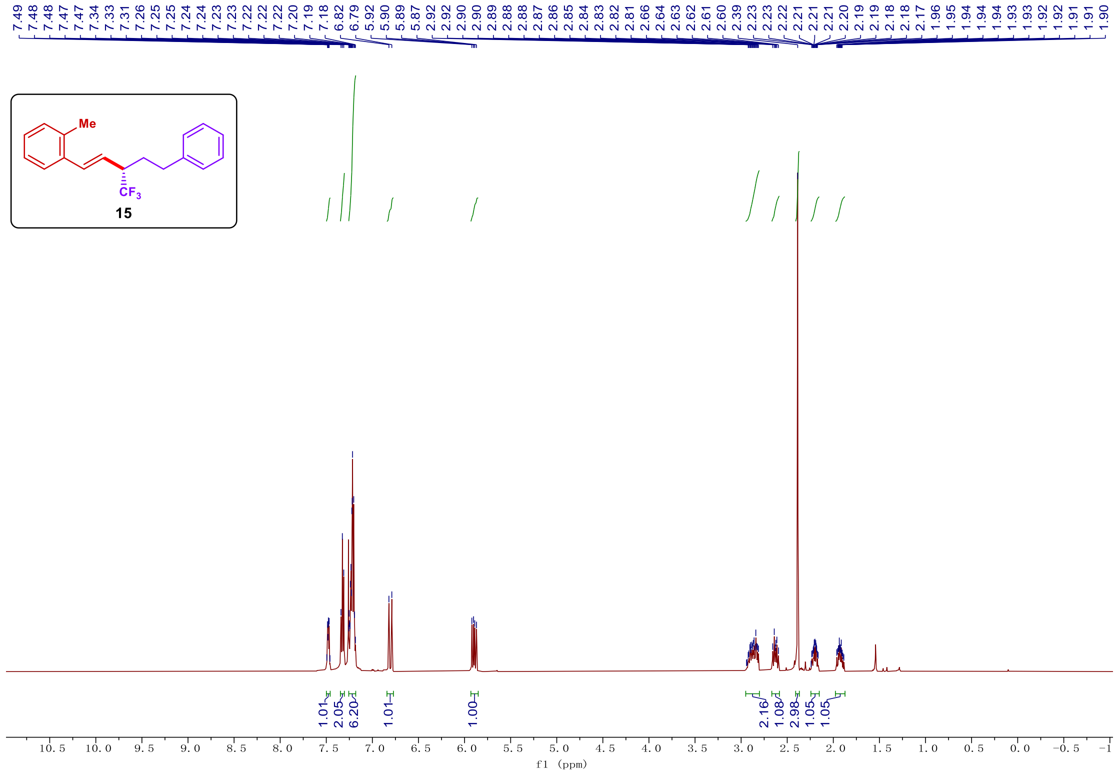

Supplementary Figure 62. ^{1}H NMR Spectrum of Compound **15** (500 MHz, $CDCl_3$)

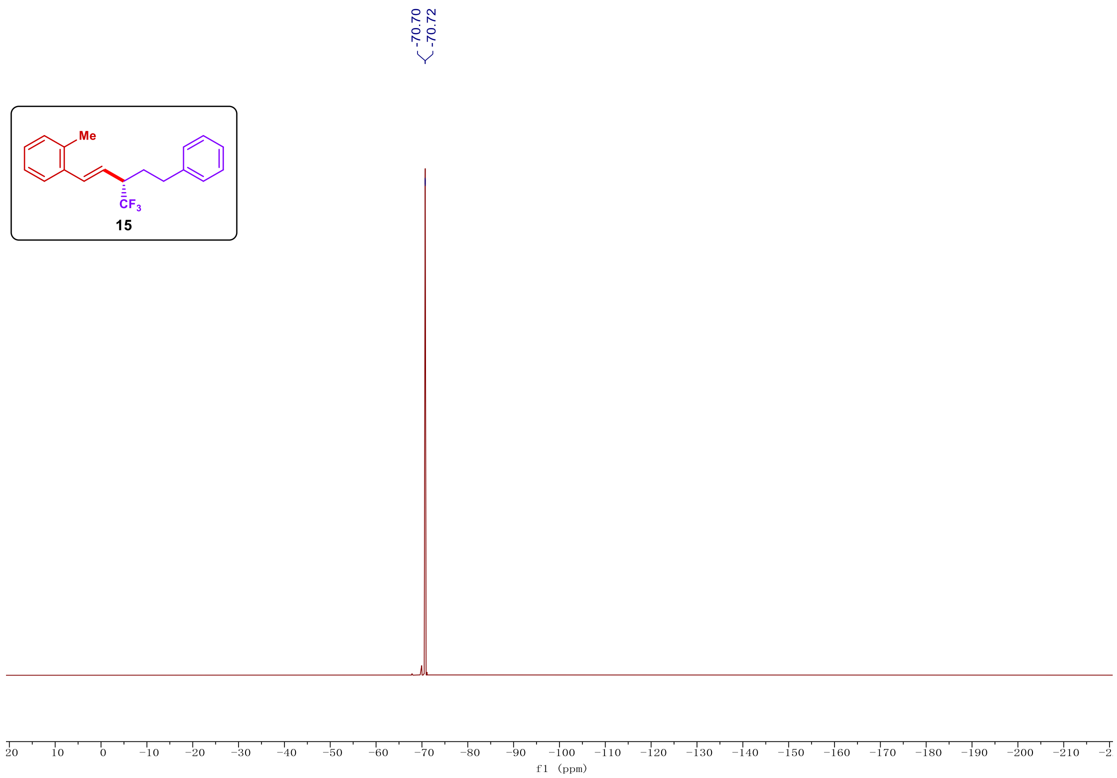

Supplementary Figure 63. ^{19}F NMR Spectrum of Compound **15** (471 MHz, $CDCl_3$)

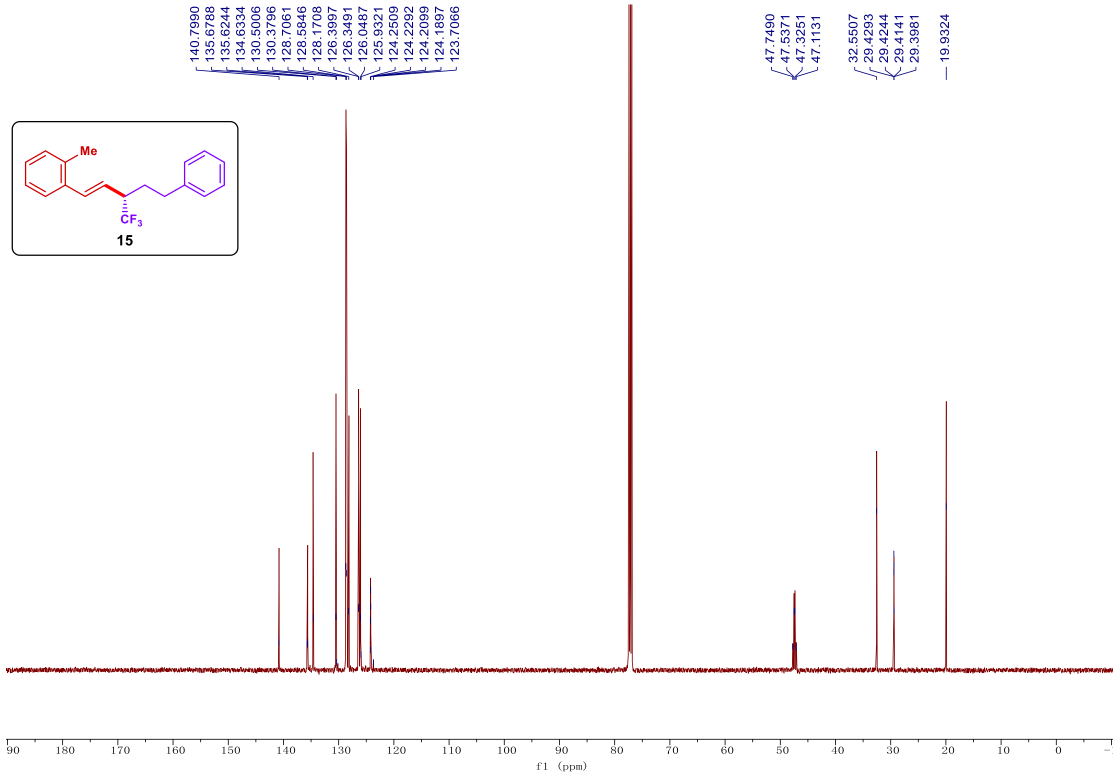

Supplementary Figure 64. ^{13}C NMR Spectrum of Compound **15** (126 MHz, $CDCl_3$)

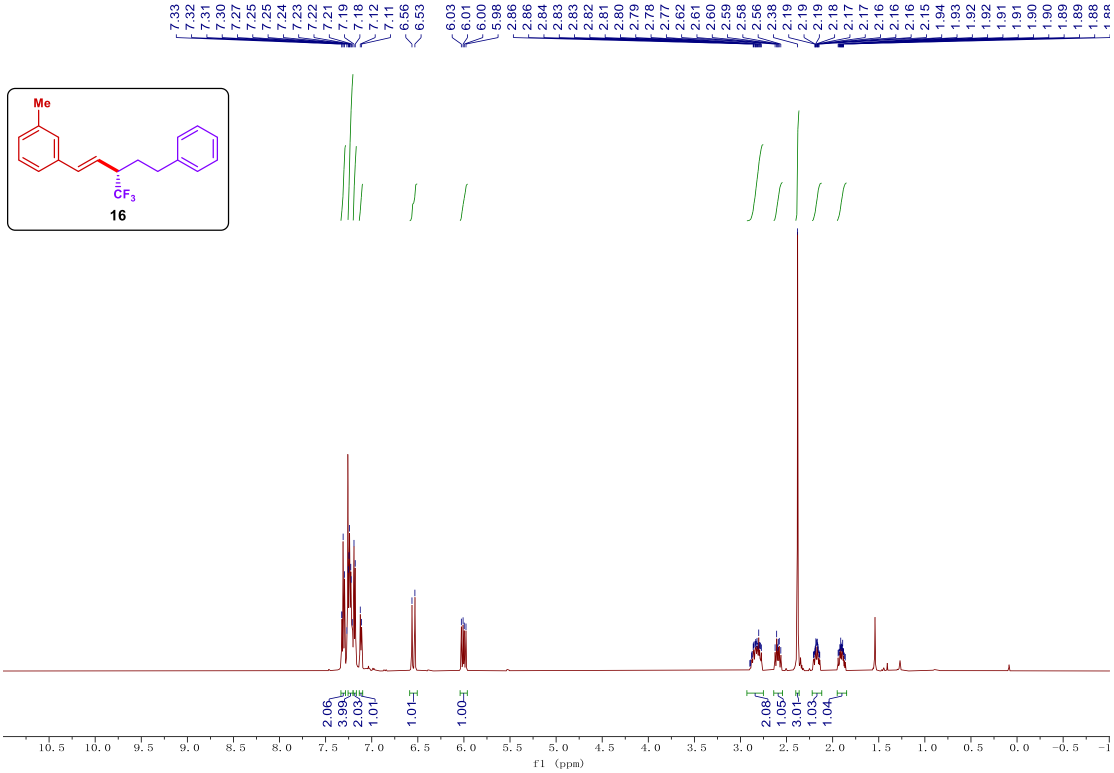

Supplementary Figure 65. ^{1}H NMR Spectrum of Compound **16** (500 MHz, $CDCl_3$)

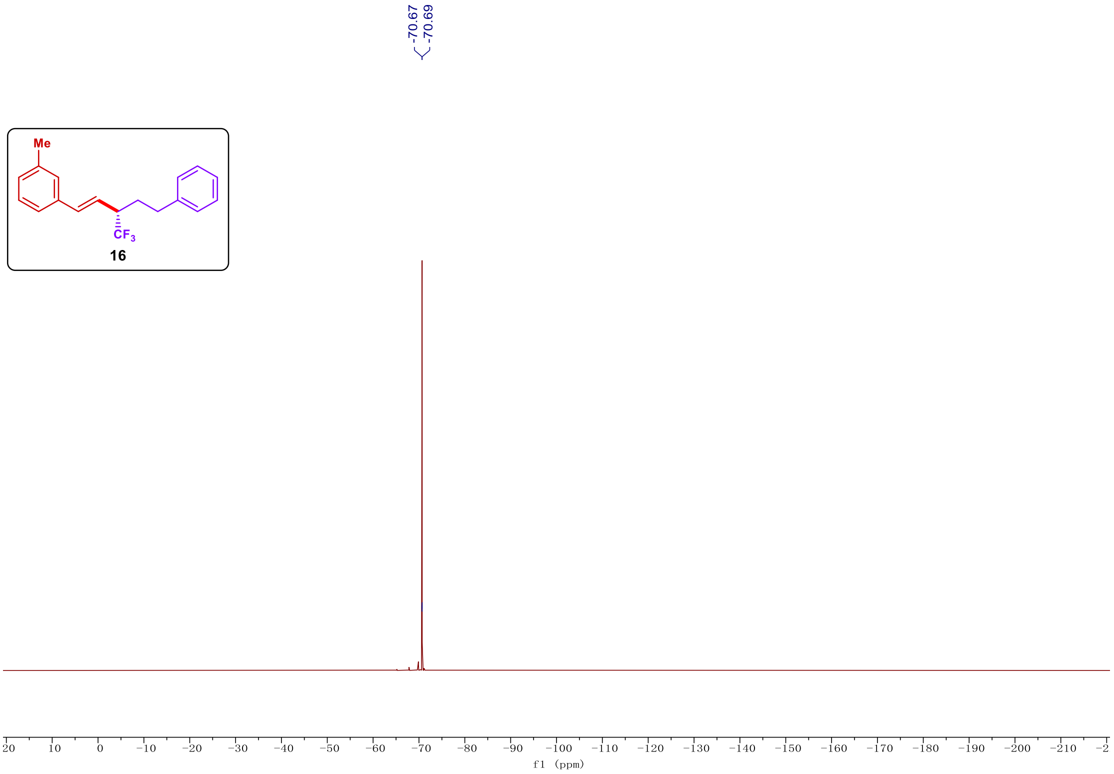

Supplementary Figure 66. ^{19}F NMR Spectrum of Compound **16** (471 MHz, $CDCl_3$)

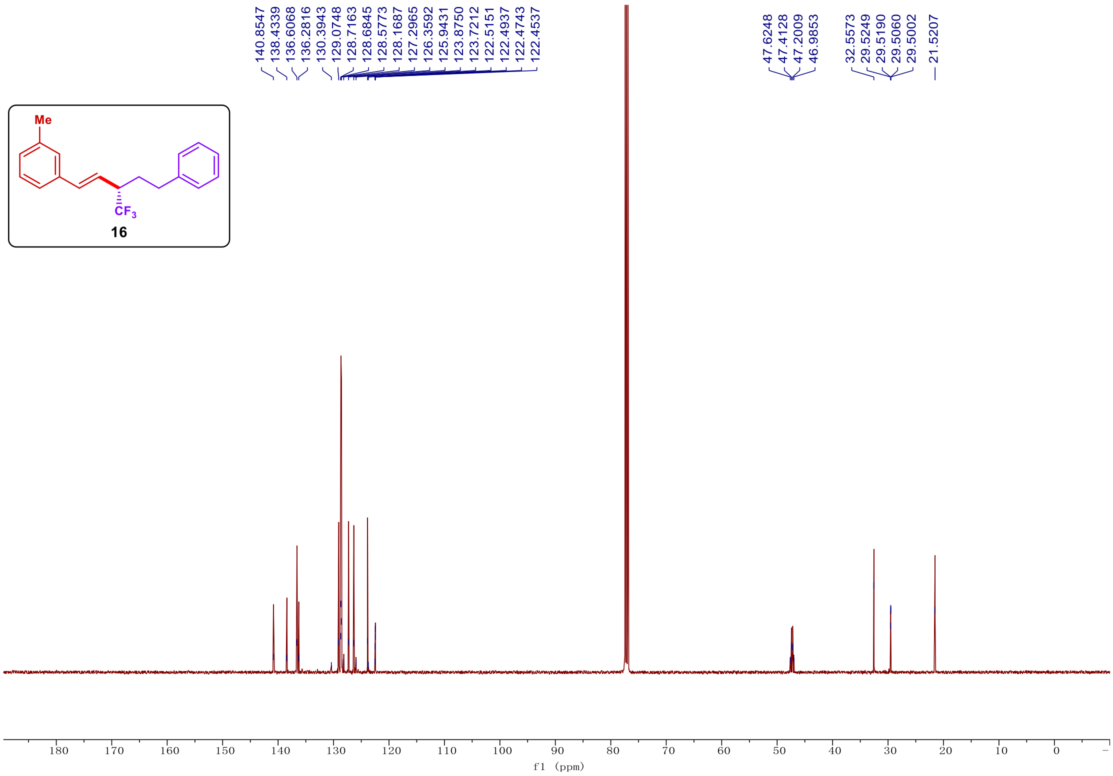

Supplementary Figure 67. ^{13}C NMR Spectrum of Compound **16** (126 MHz, $CDCl_3$)

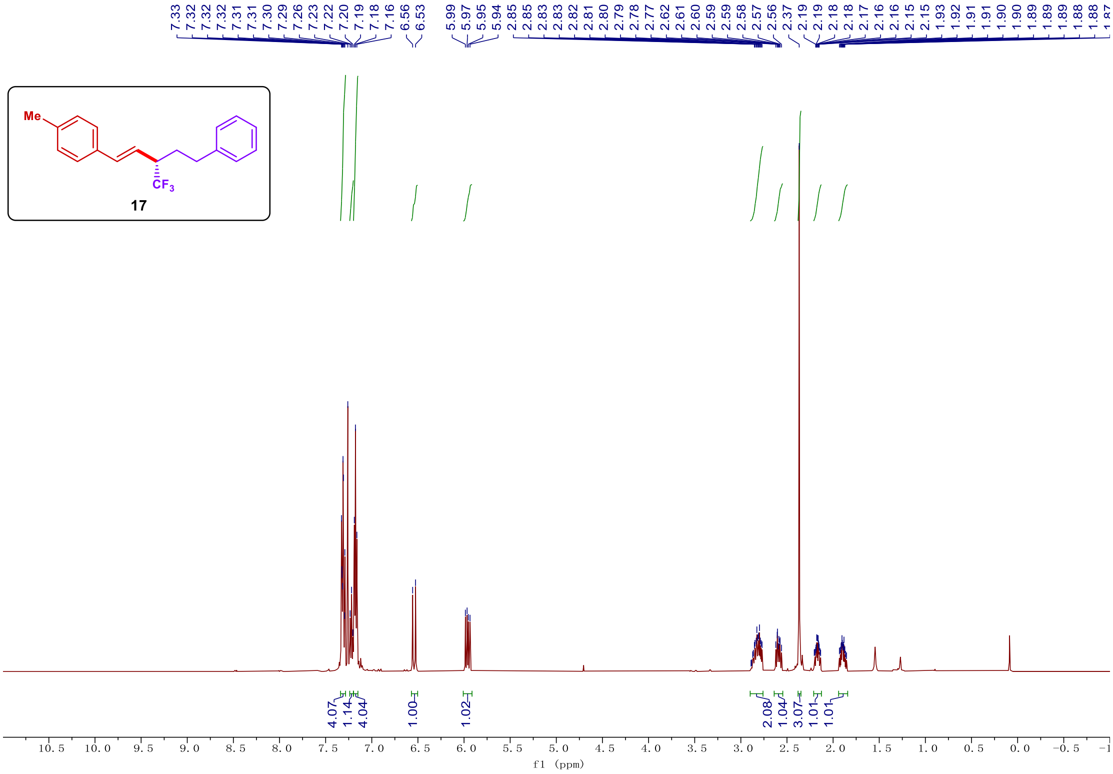

Supplementary Figure 68. ^{1}H NMR Spectrum of Compound **17** (500 MHz, $CDCl_3$)

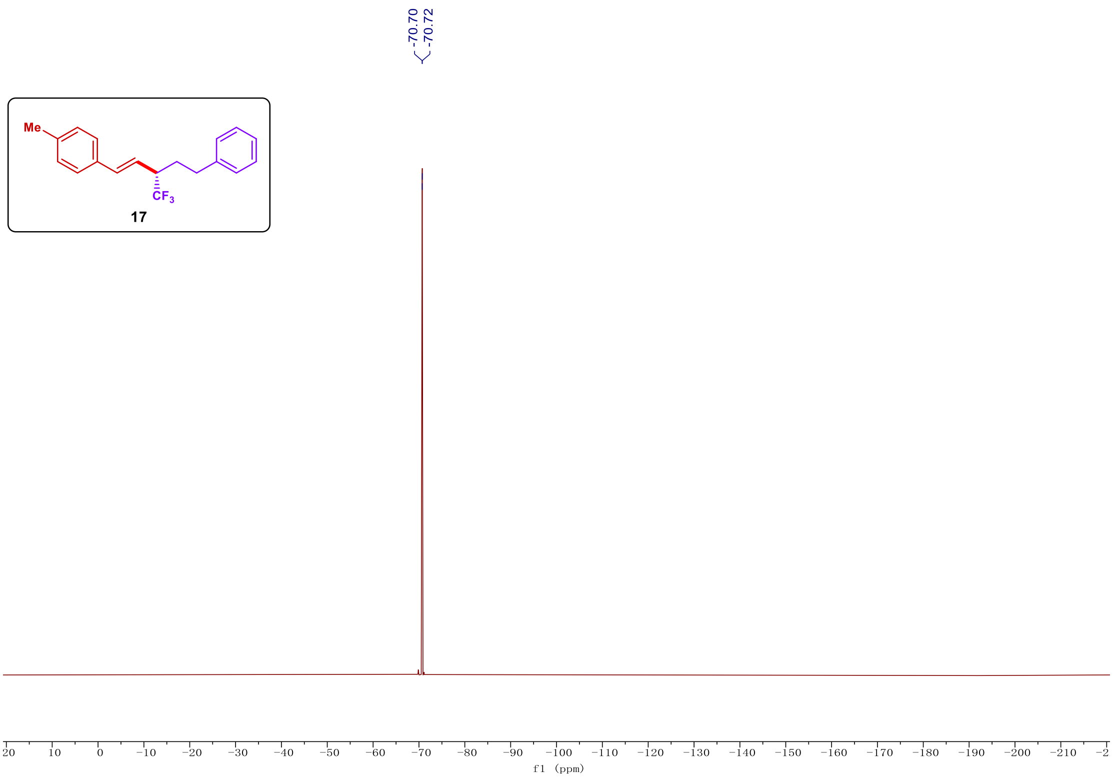

Supplementary Figure 69. ^{19}F NMR Spectrum of Compound **17** (376 MHz, $CDCl_3$)

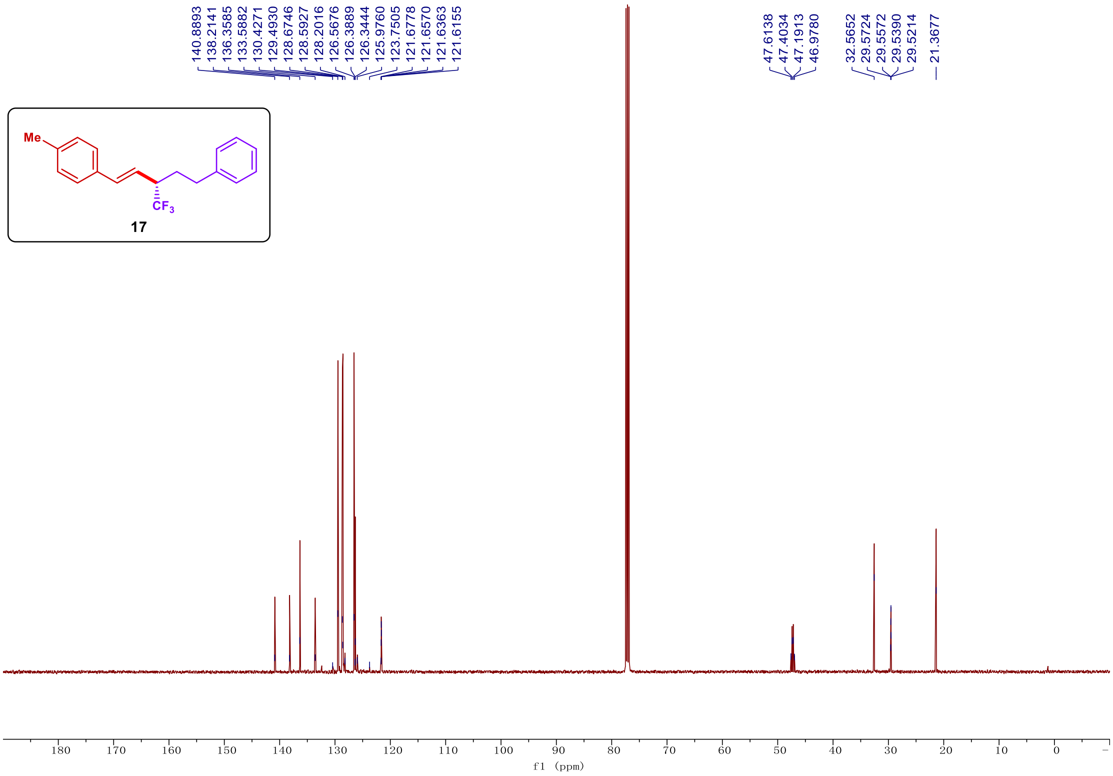

Supplementary Figure 70. ^{13}C NMR Spectrum of Compound **17** (126 MHz, $CDCl_3$)

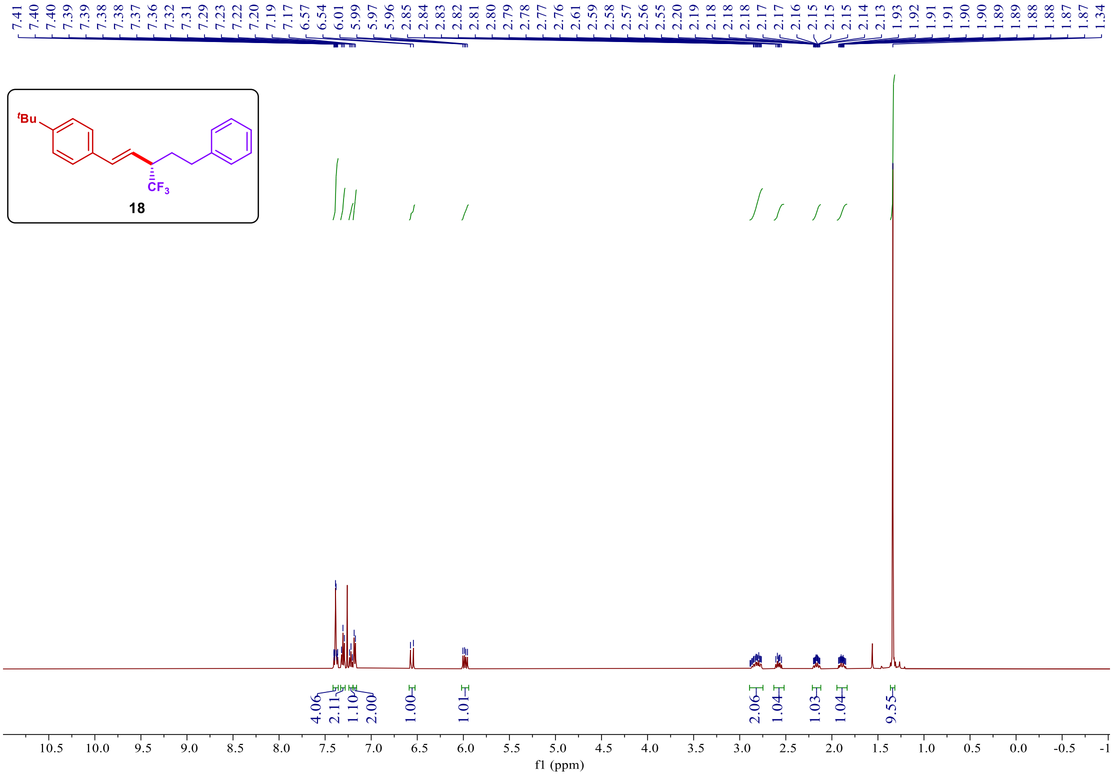

Supplementary Figure 71. ^{1}H NMR Spectrum of Compound **18** (500 MHz, $CDCl_3$)

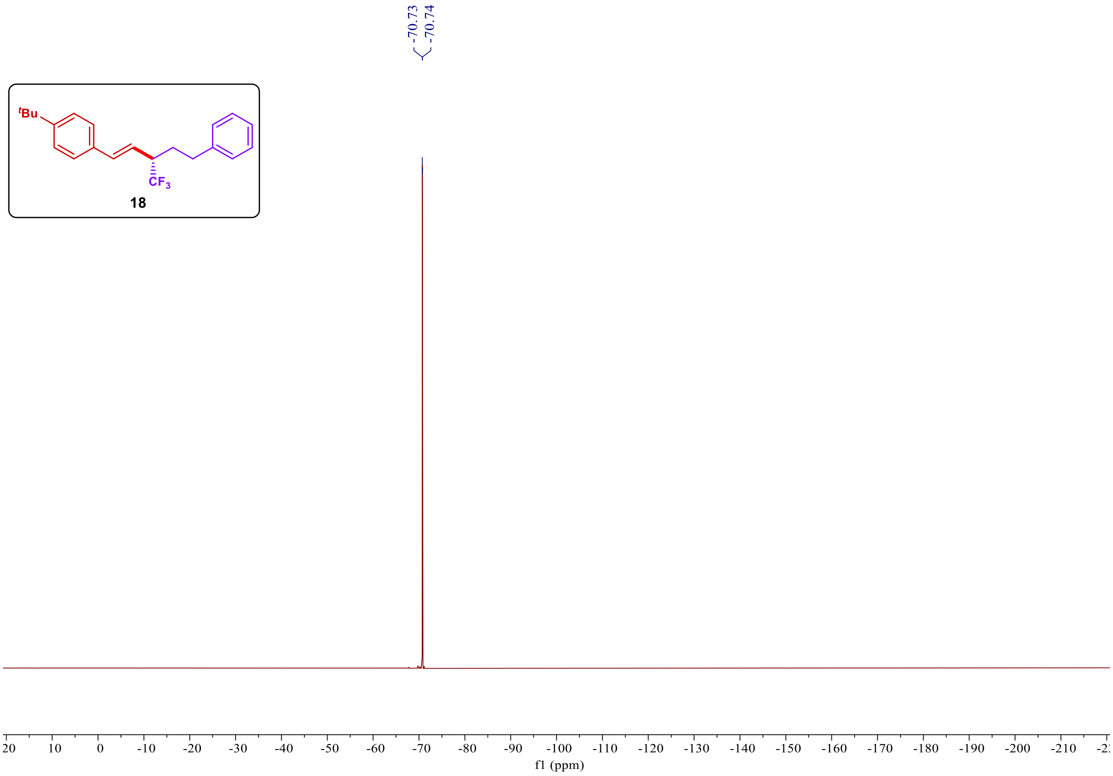

Supplementary Figure 72. ^{19}F NMR Spectrum of Compound **18** (471 MHz, $CDCl_3$)

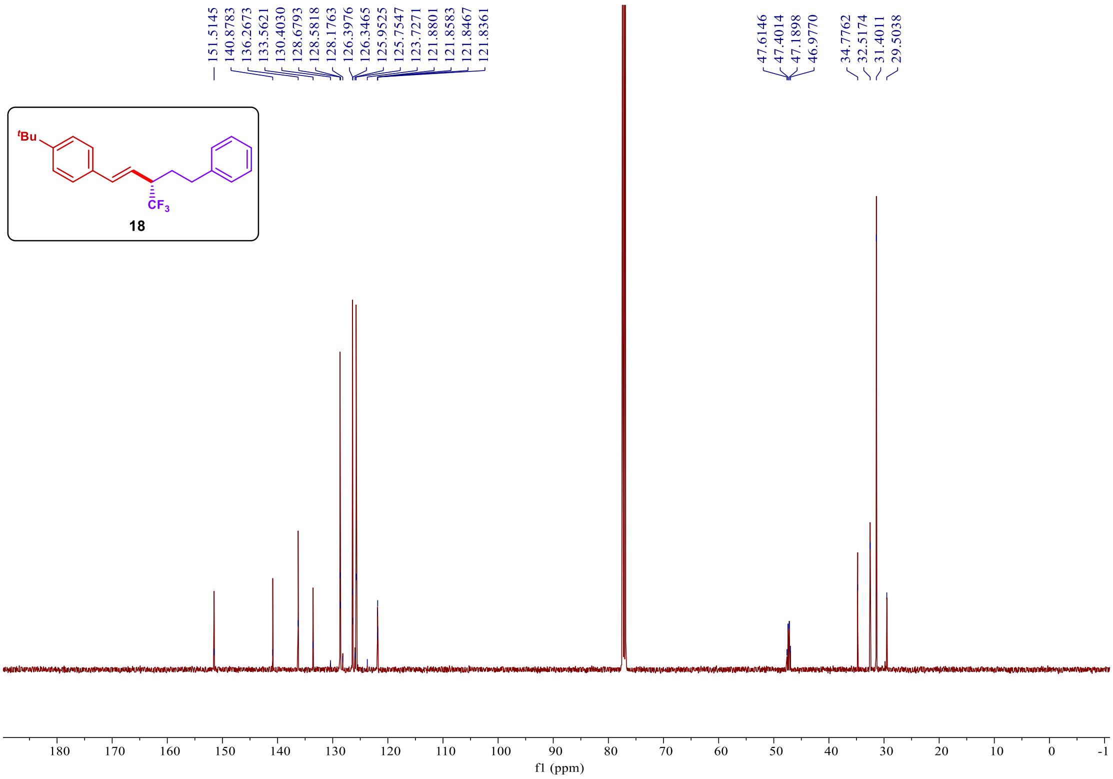

Supplementary Figure 73. ^{13}C NMR Spectrum of Compound **18** (126 MHz, $CDCl_3$)

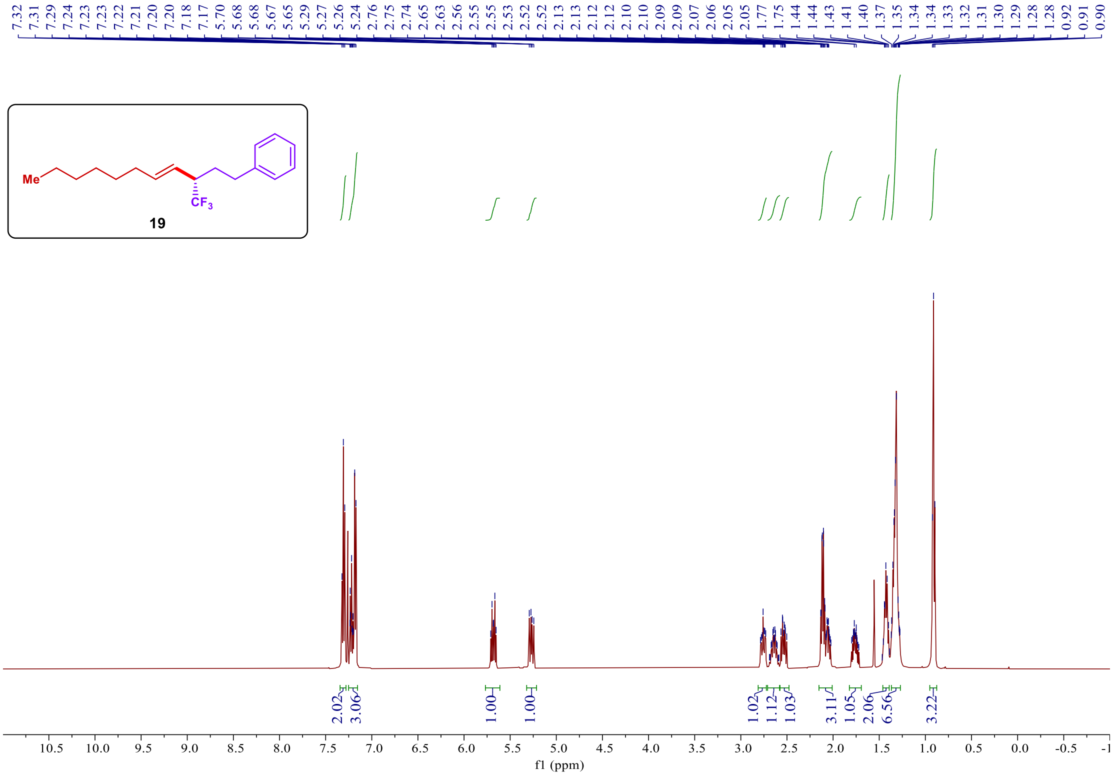

Supplementary Figure 74. ^{1}H NMR Spectrum of Compound **19** (500 MHz, $CDCl_3$)

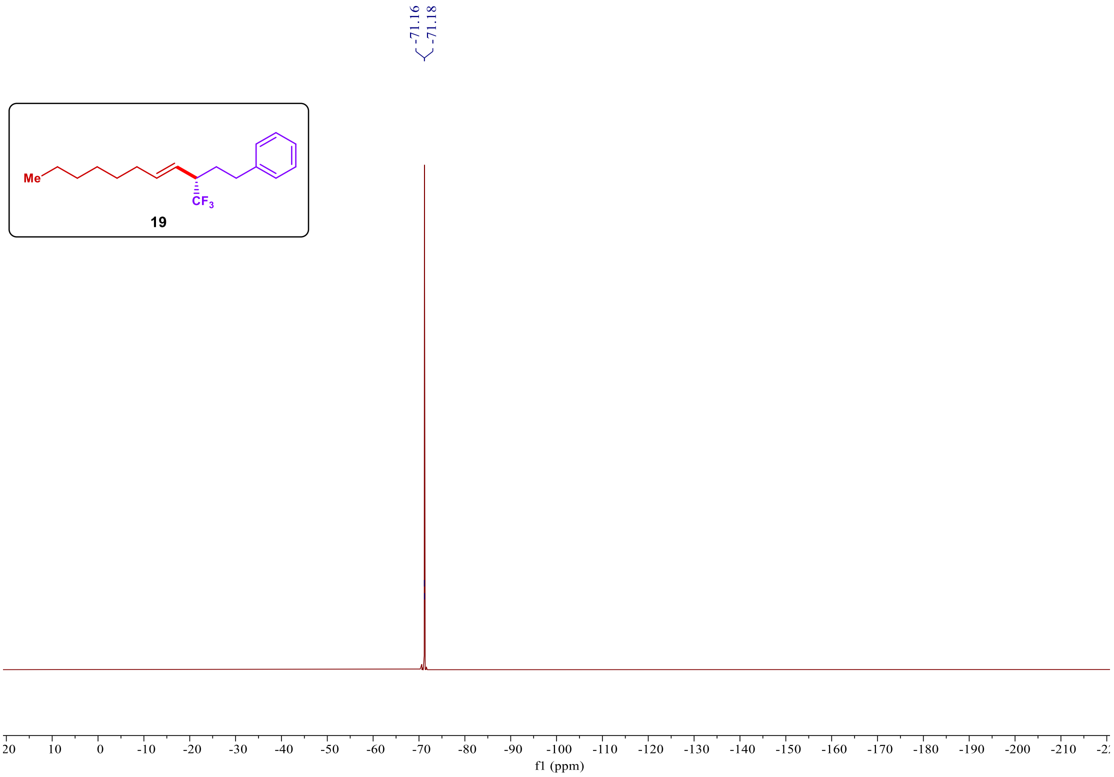

Supplementary Figure 75. ^{19}F NMR Spectrum of Compound **19** (471 MHz, $CDCl_3$)

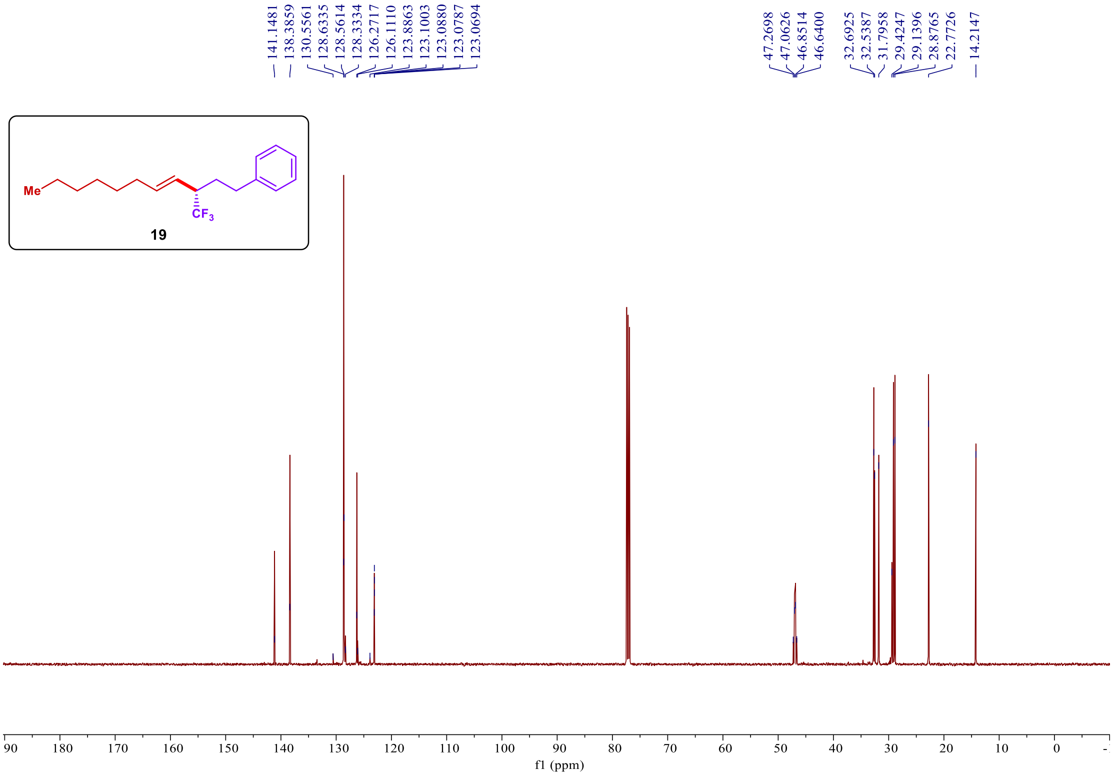

Supplementary Figure 76. ^{13}C NMR Spectrum of Compound **19** (126 MHz, $CDCl_3$)

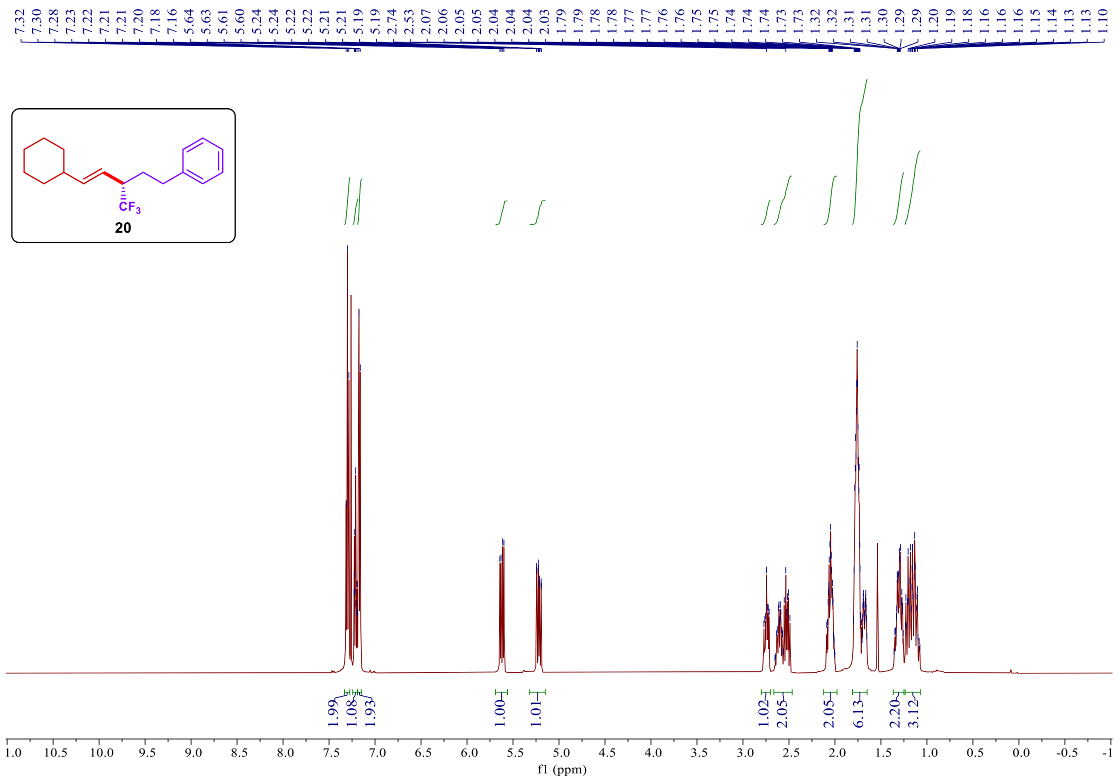

Supplementary Figure 77. ^{1}H NMR Spectrum of Compound **20** (500 MHz, $CDCl_3$)

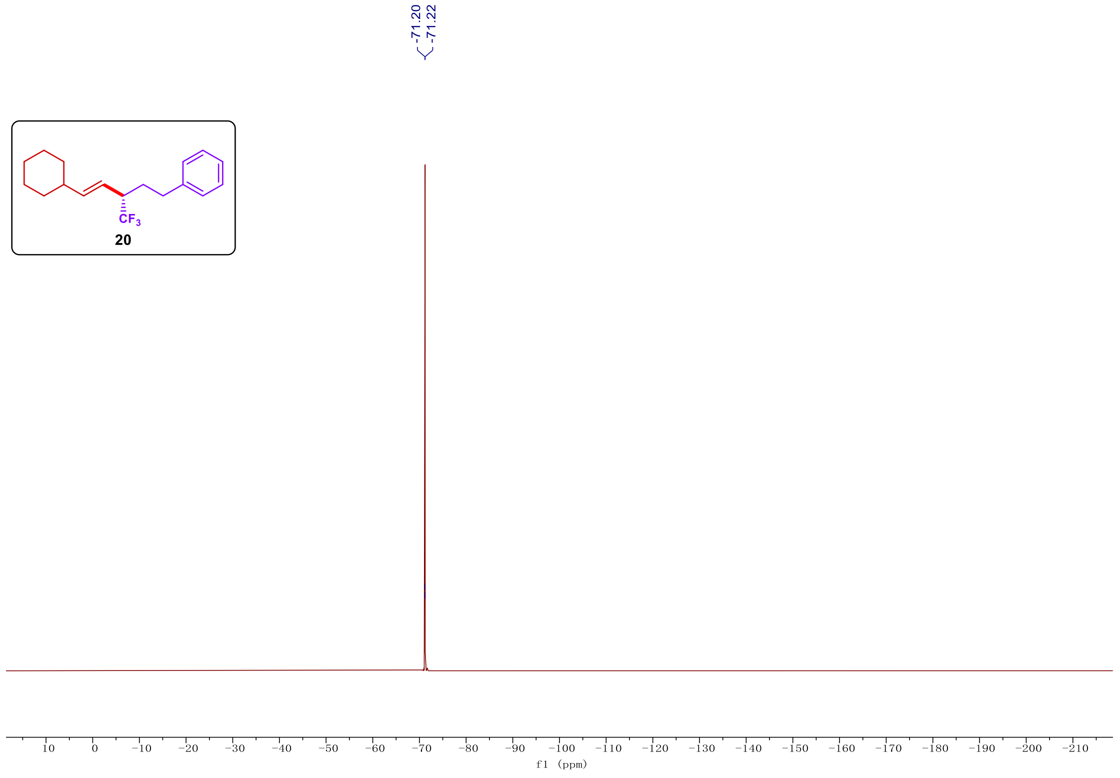

Supplementary Figure 78. ^{19}F NMR Spectrum of Compound **20** (376 MHz, $CDCl_3$)

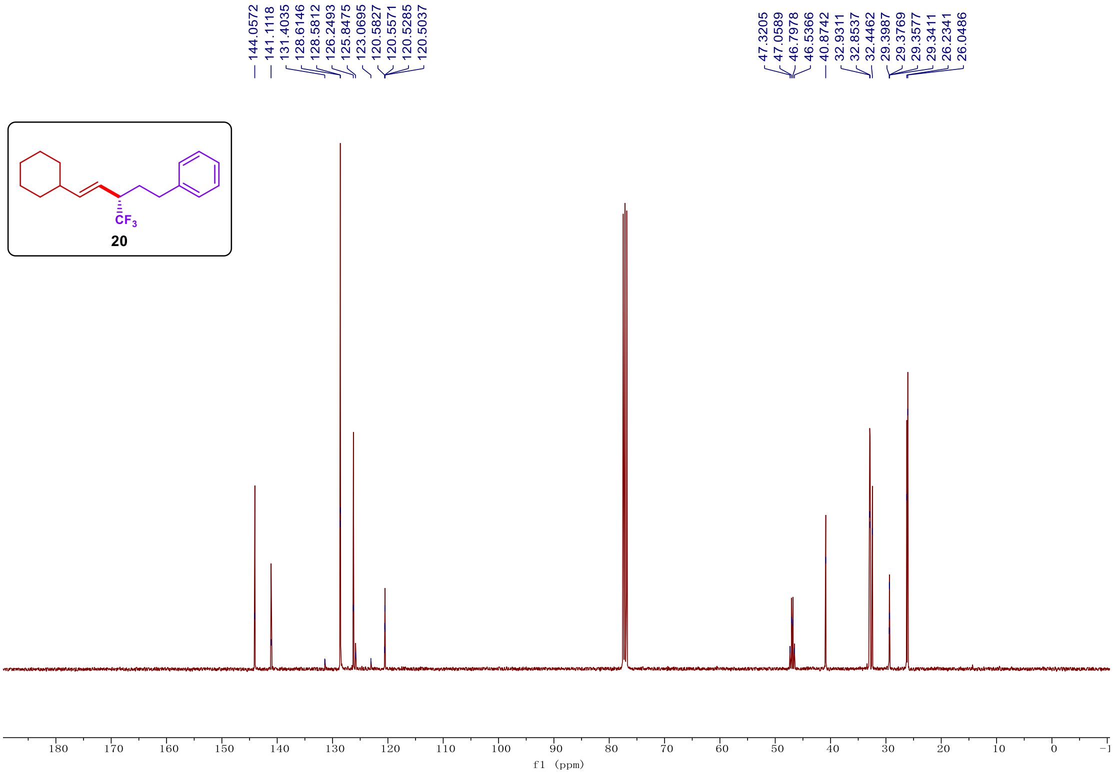

Supplementary Figure 79. ^{13}C NMR Spectrum of Compound **20** (101 MHz, $CDCl_3$)

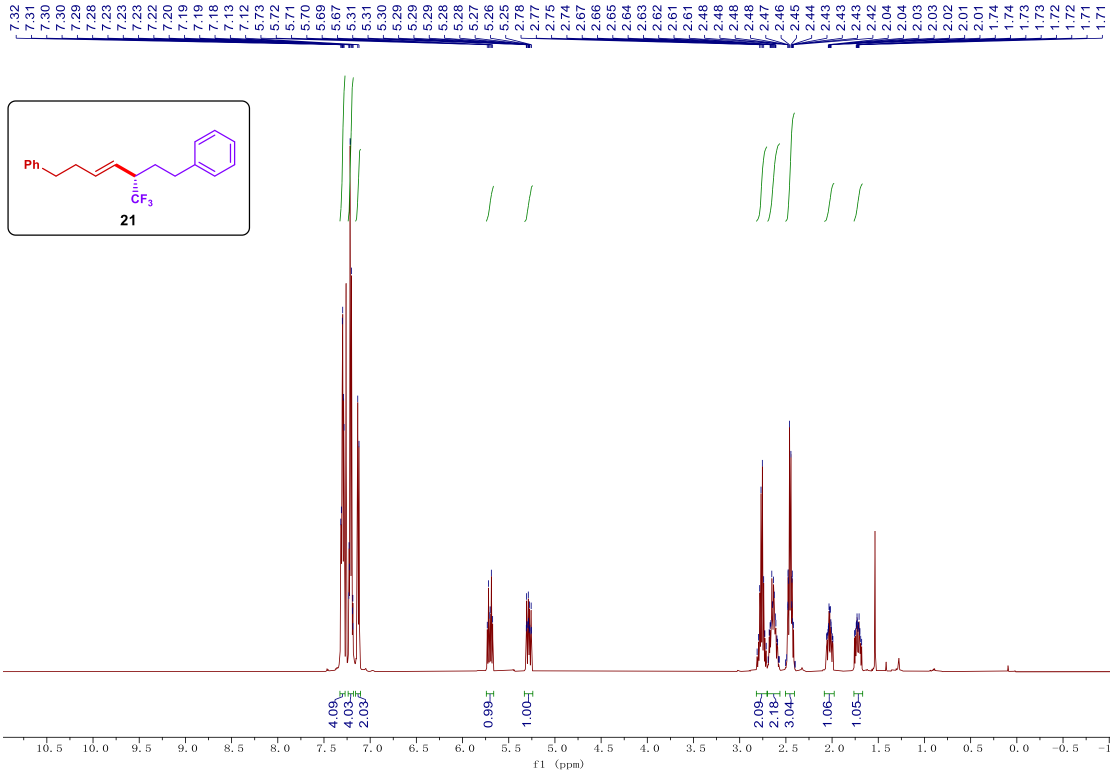

Supplementary Figure 80. ^{1}H NMR Spectrum of Compound **21** (500 MHz, $CDCl_3$)

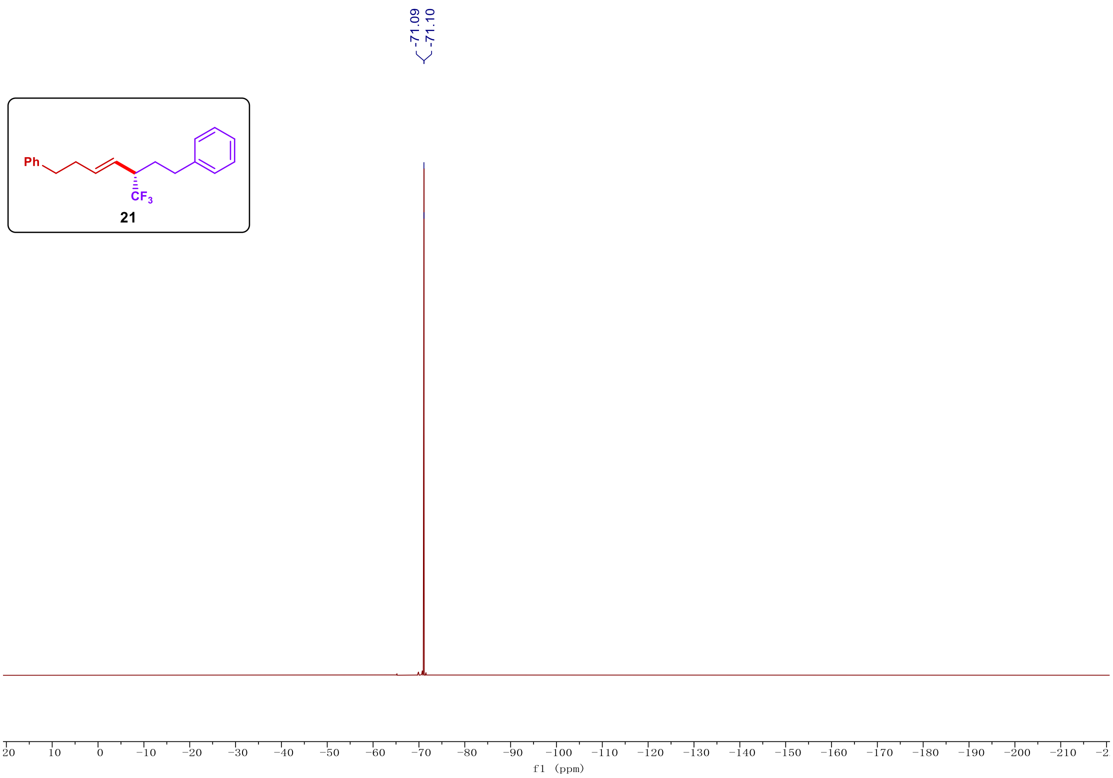

Supplementary Figure 81. ^{19}F NMR Spectrum of Compound **21** (471 MHz, $CDCl_3$)

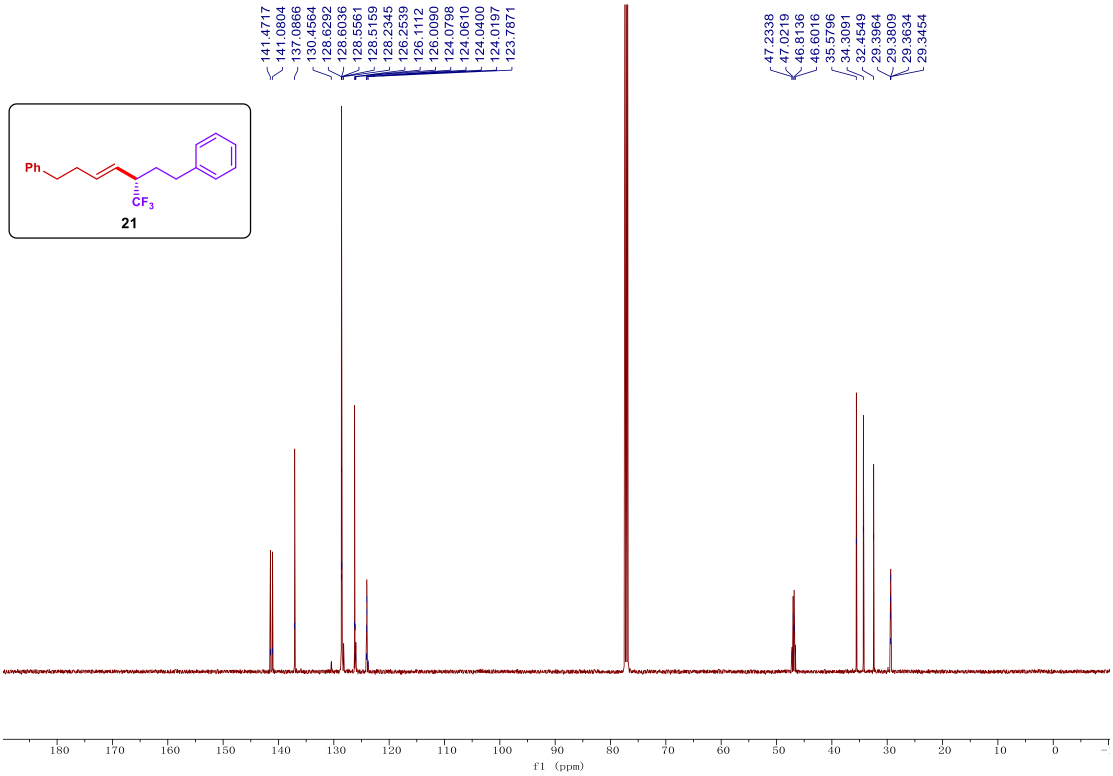

Supplementary Figure 82. ^{13}C NMR Spectrum of Compound **21** (126 MHz, $CDCl_3$)

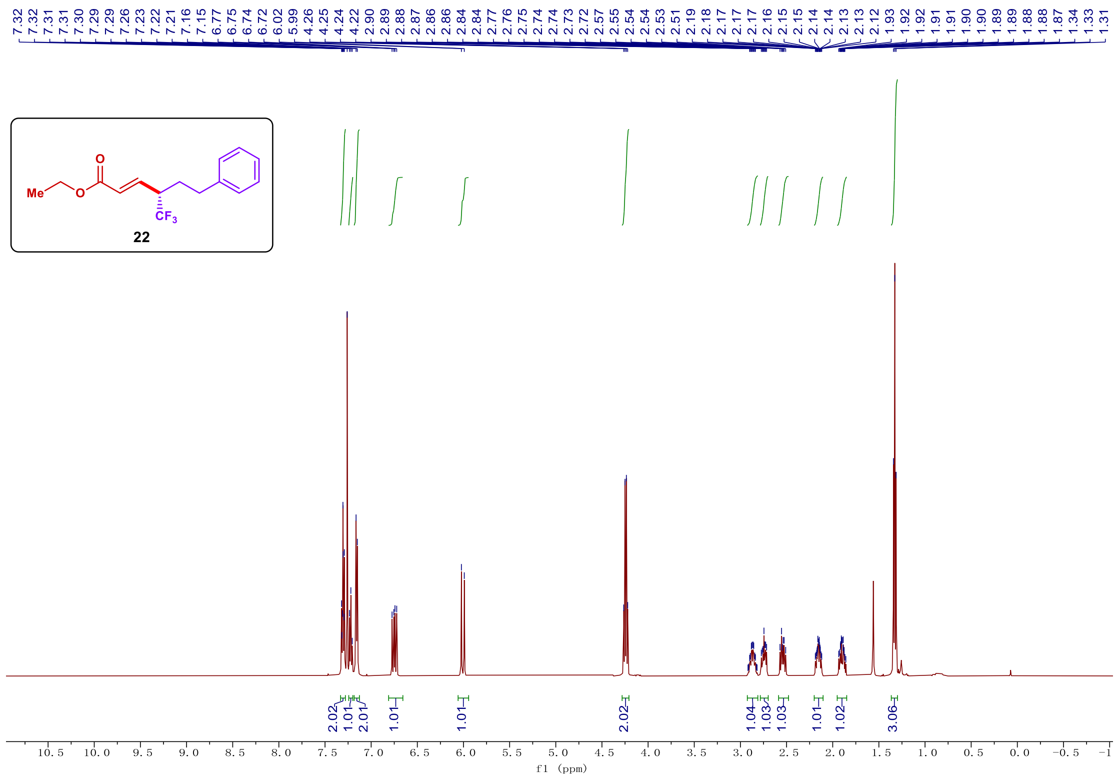

Supplementary Figure 83. ^{1}H NMR Spectrum of Compound **22** (500 MHz, $CDCl_3$)

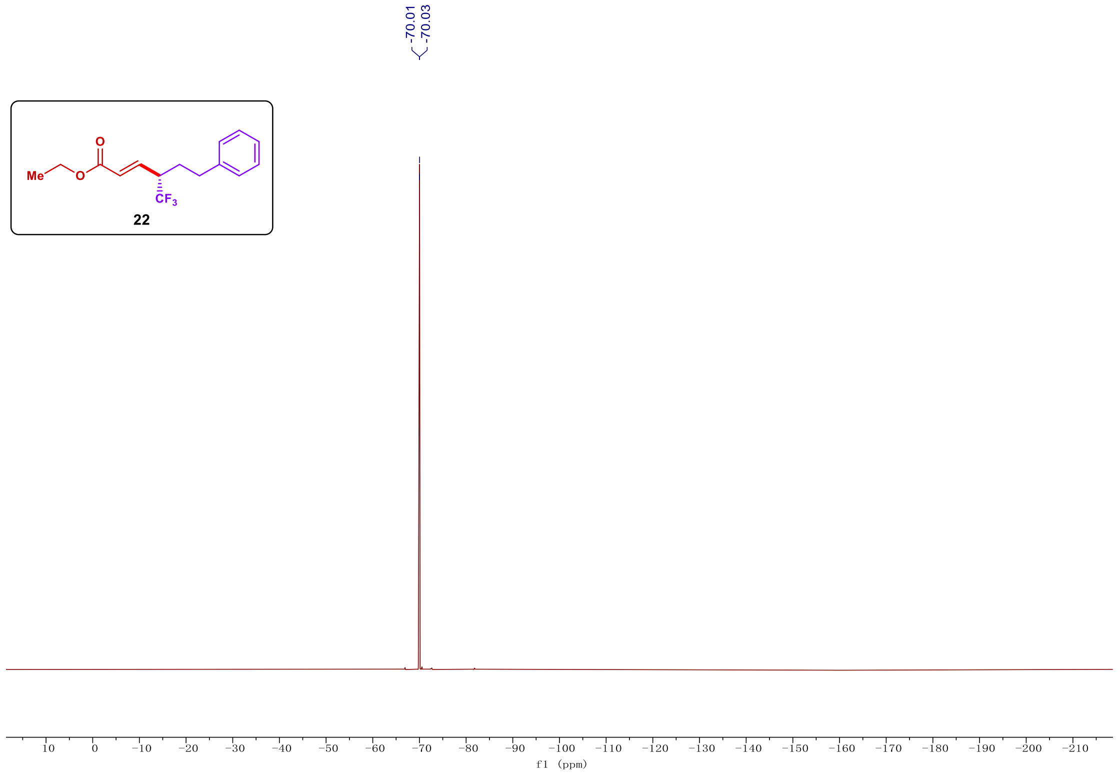

Supplementary Figure 84. ^{19}F NMR Spectrum of Compound **22** (376 MHz, $CDCl_3$)

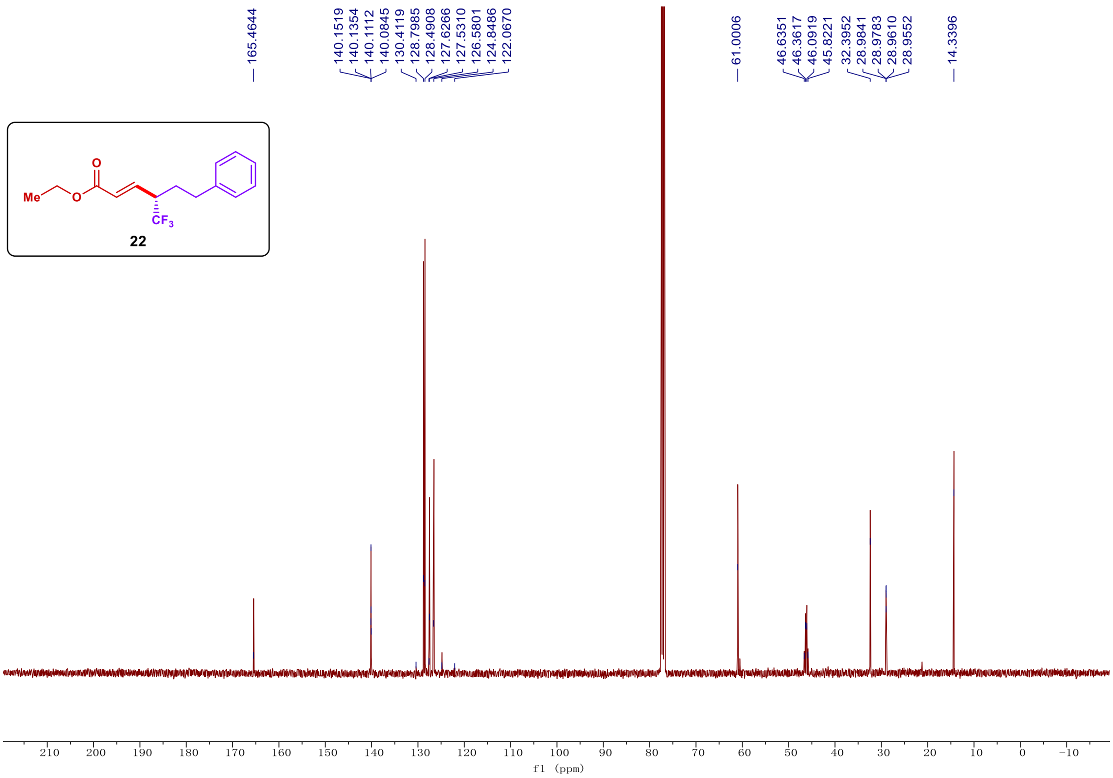

Supplementary Figure 85. ^{13}C NMR Spectrum of Compound **22** (101 MHz, $CDCl_3$)

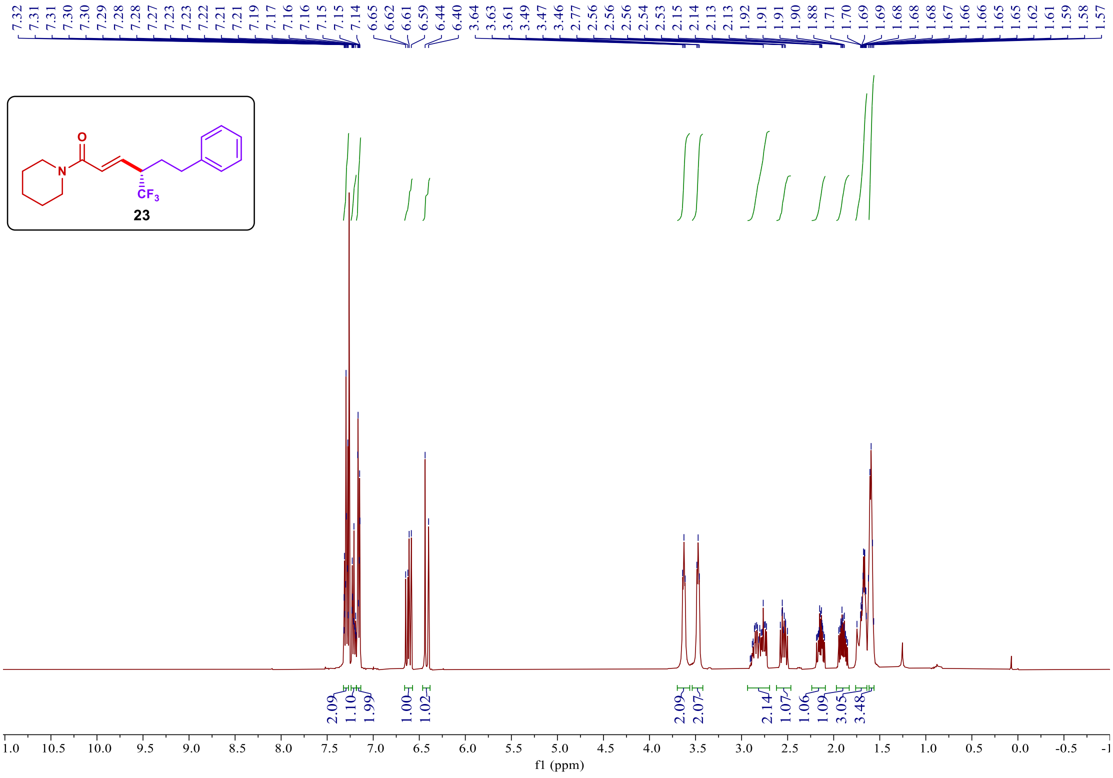

Supplementary Figure 86. ^{1}H NMR Spectrum of Compound **23** (400 MHz, $CDCl_3$)

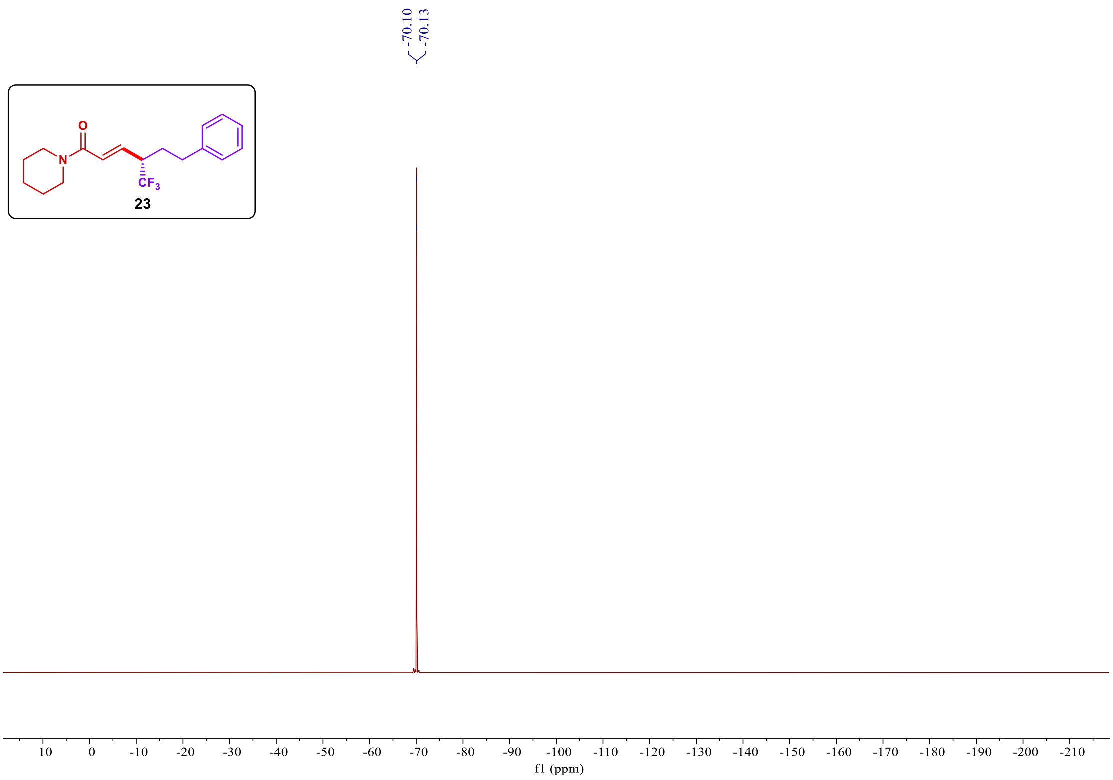

Supplementary Figure 87. ^{19}F NMR Spectrum of Compound **23** (376 MHz, $CDCl_3$)

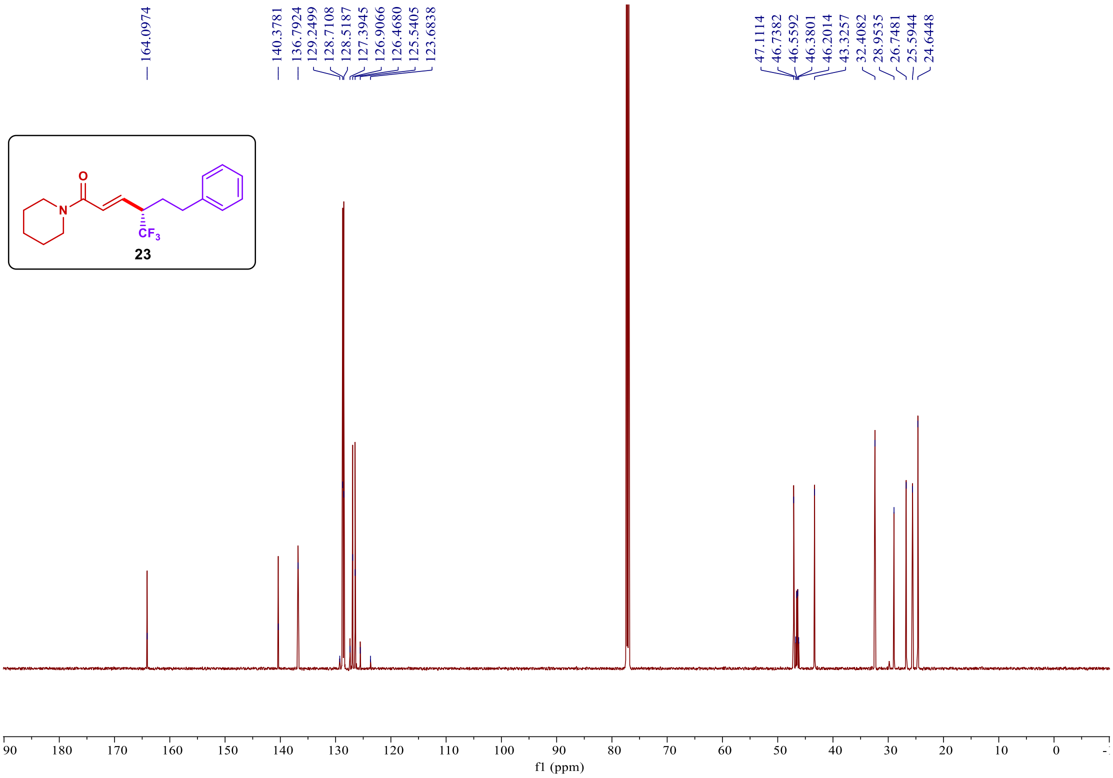

Supplementary Figure 88. ^{13}C NMR Spectrum of Compound **23** (151 MHz, $CDCl_3$)

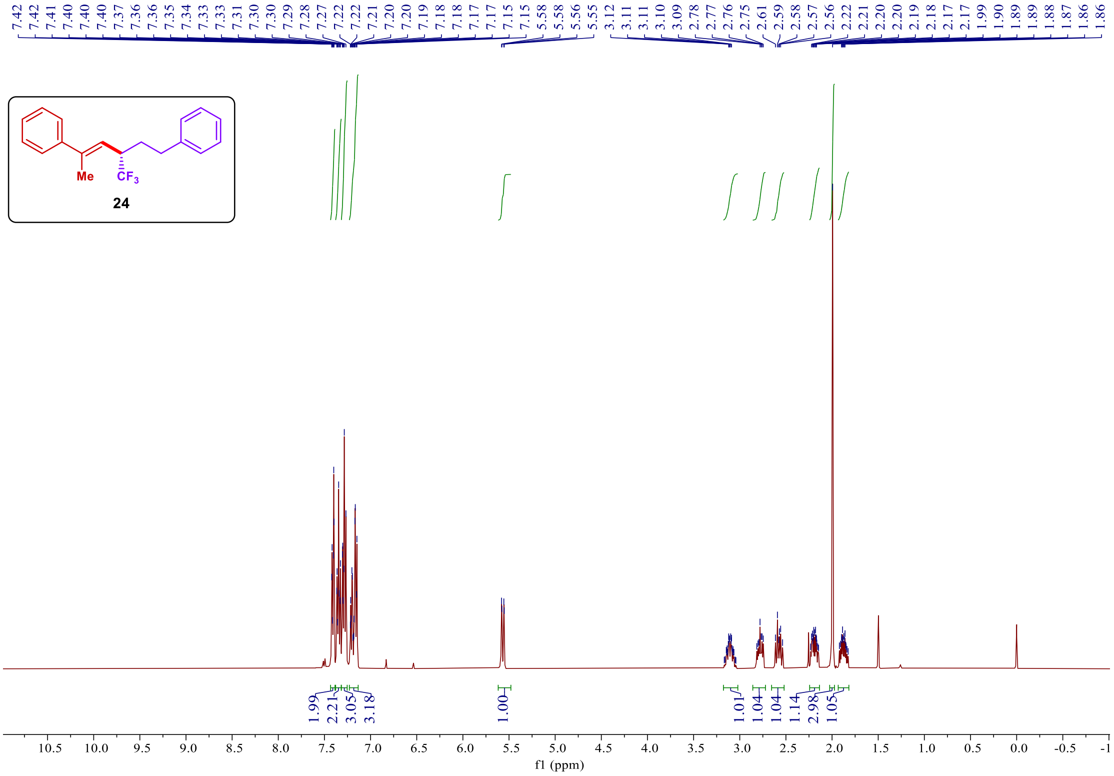

Supplementary Figure 89. ^{1}H NMR Spectrum of Compound **24** (400 MHz, $CDCl_3$)

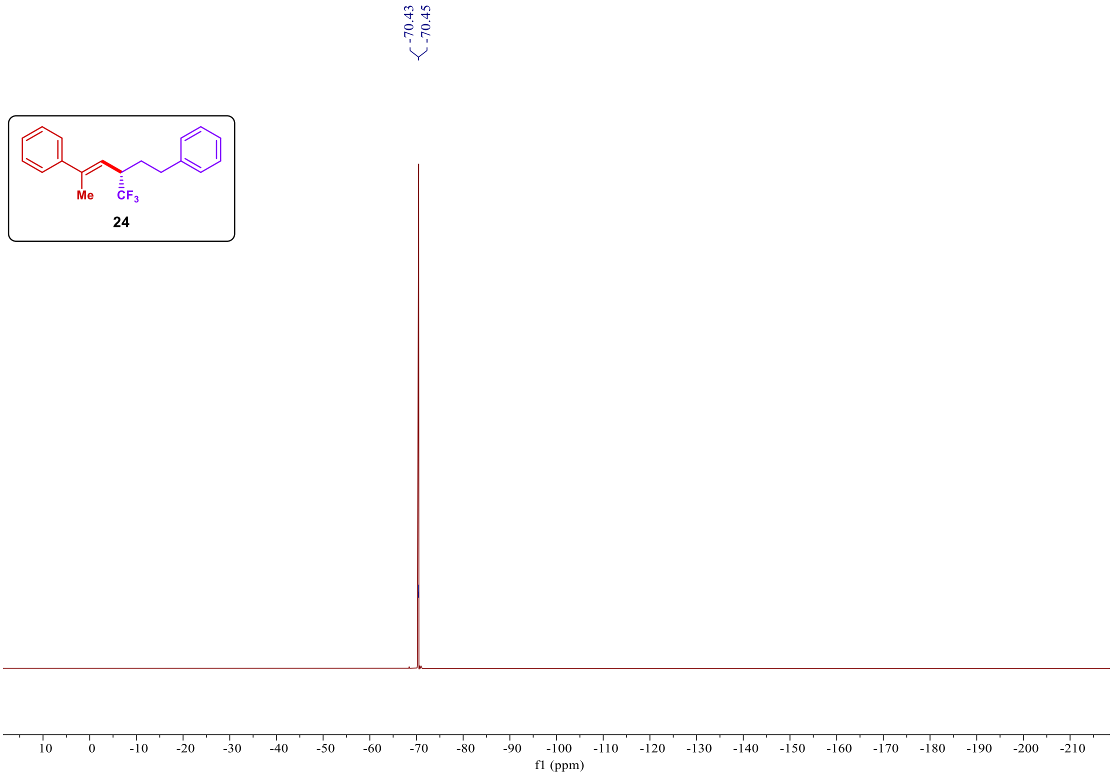

Supplementary Figure 90. ^{19}F NMR Spectrum of Compound **24** (376 MHz, $CDCl_3$)

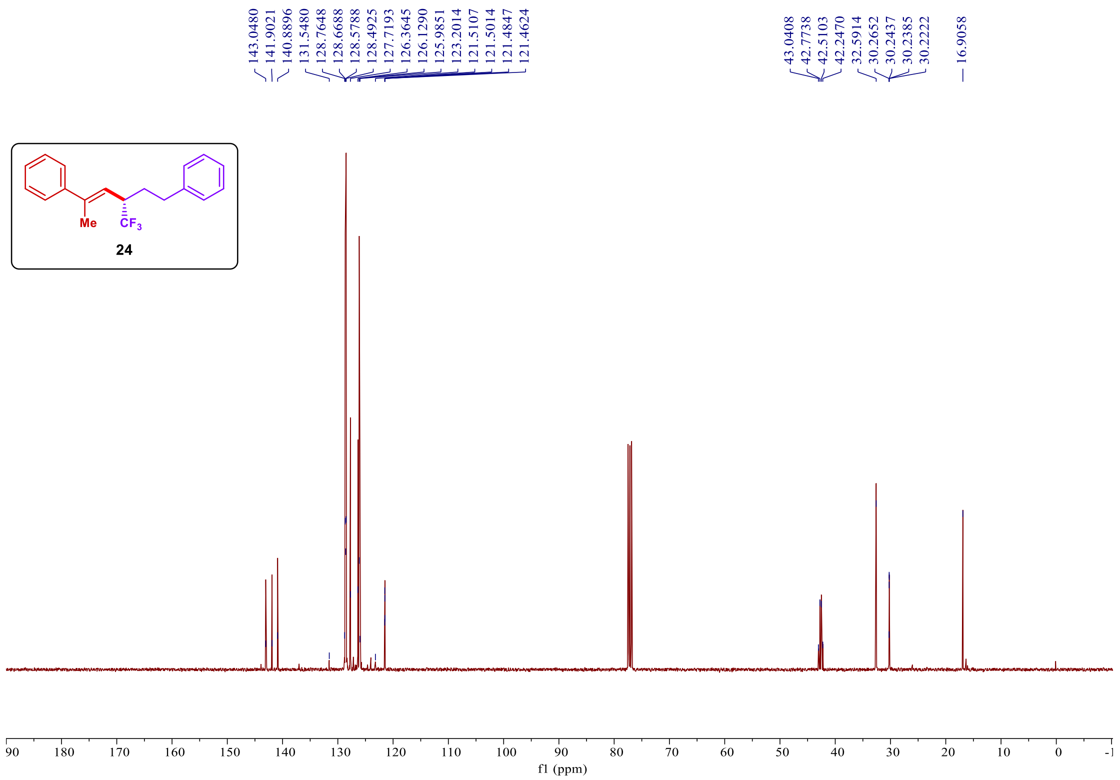

Supplementary Figure 91. ^{13}C NMR Spectrum of Compound **24** (101 MHz, $CDCl_3$)

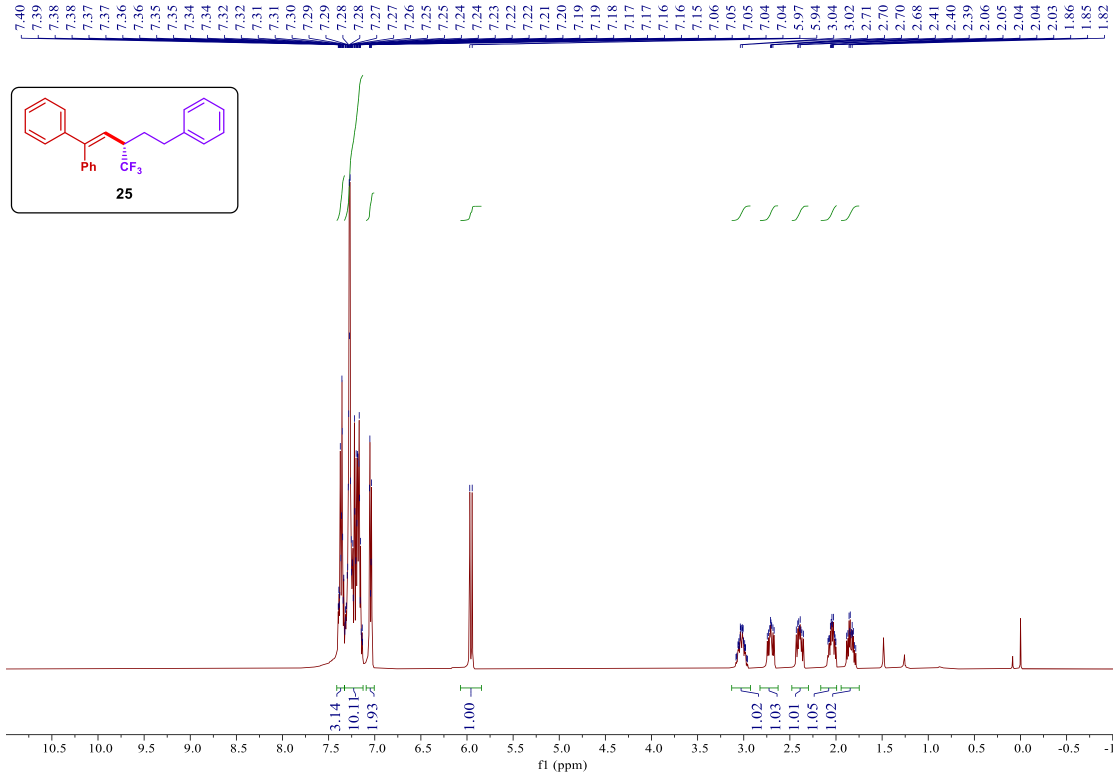

Supplementary Figure 92. ^{1}H NMR Spectrum of Compound **25** (400 MHz, $CDCl_3$)

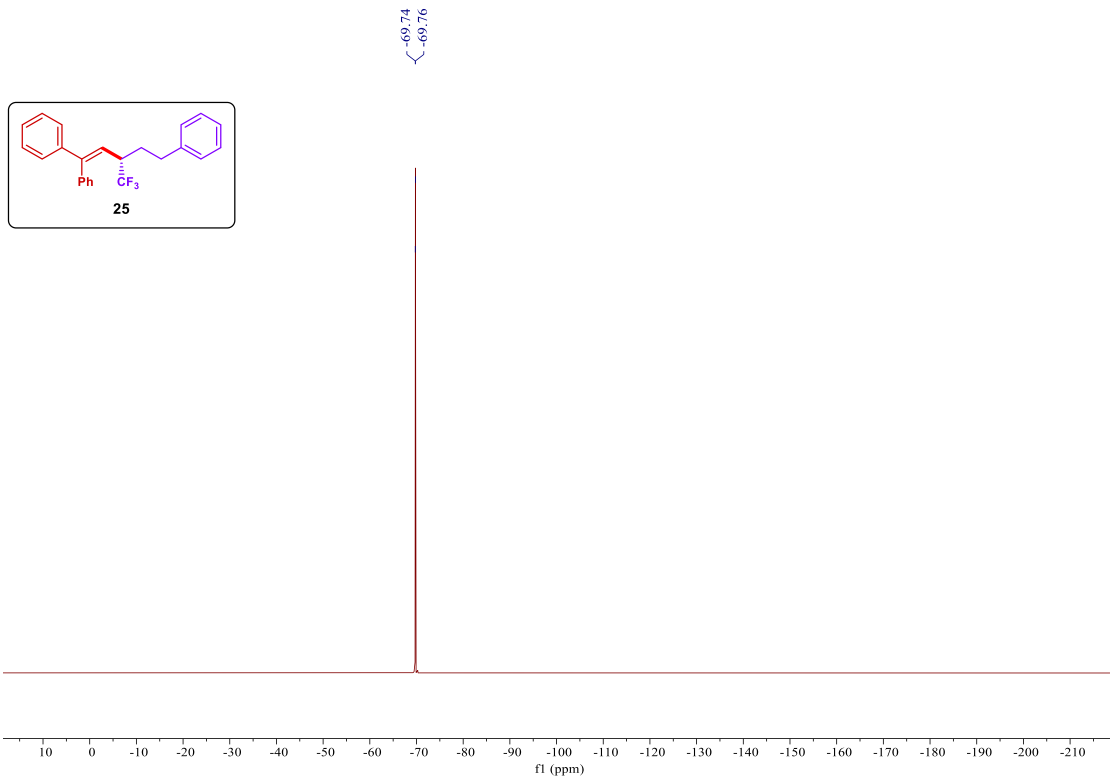

Supplementary Figure 93. ^{19}F NMR Spectrum of Compound **25** (376 MHz, $CDCl_3$)

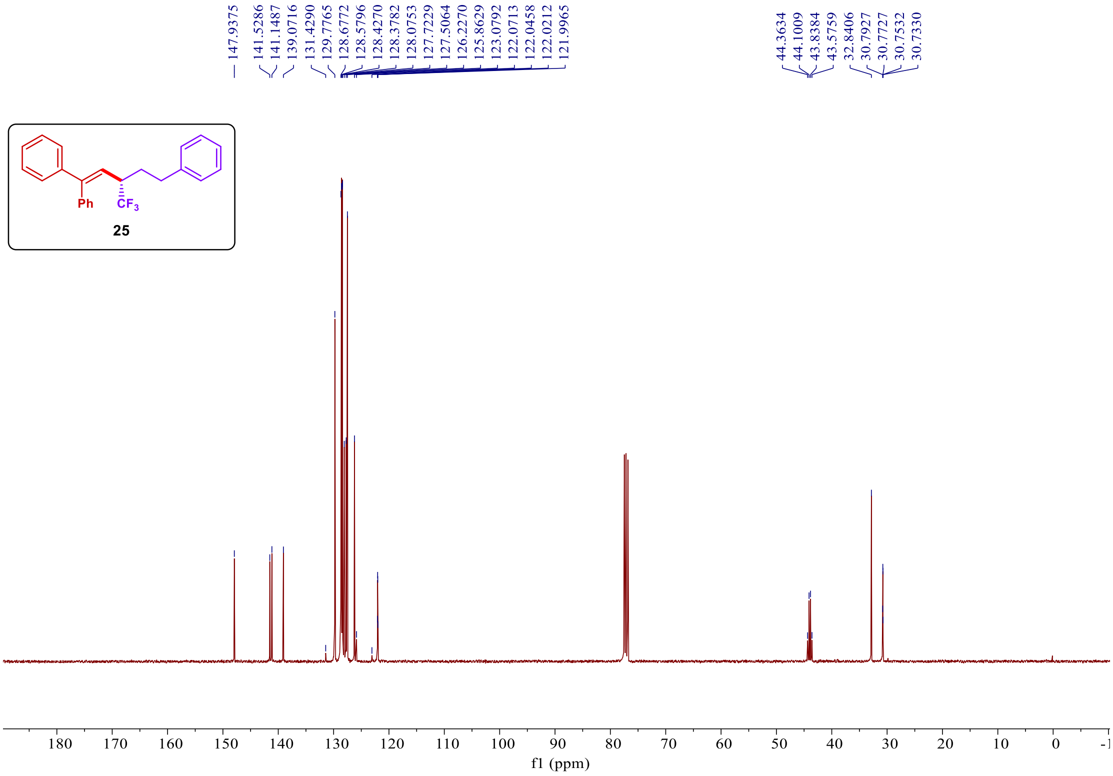

Supplementary Figure 94. ^{13}C NMR Spectrum of Compound **25** (101 MHz, $CDCl_3$)

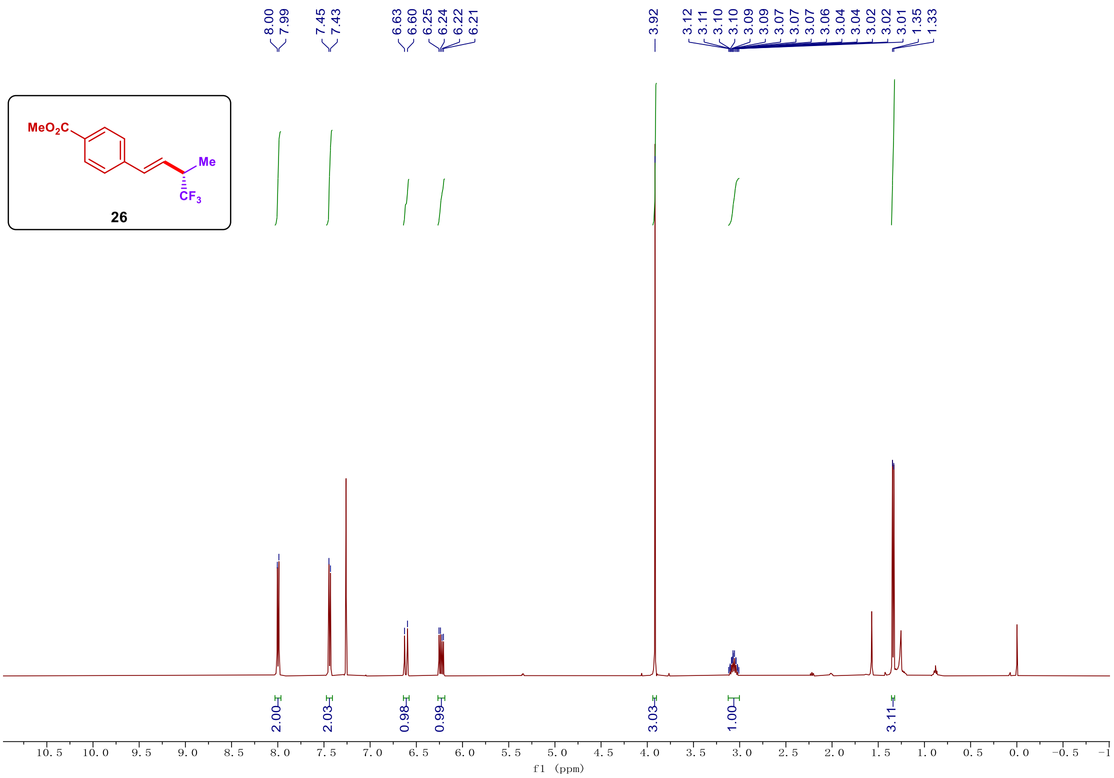

Supplementary Figure 95. ^{1}H NMR Spectrum of Compound **26** (500 MHz, $CDCl_3$)

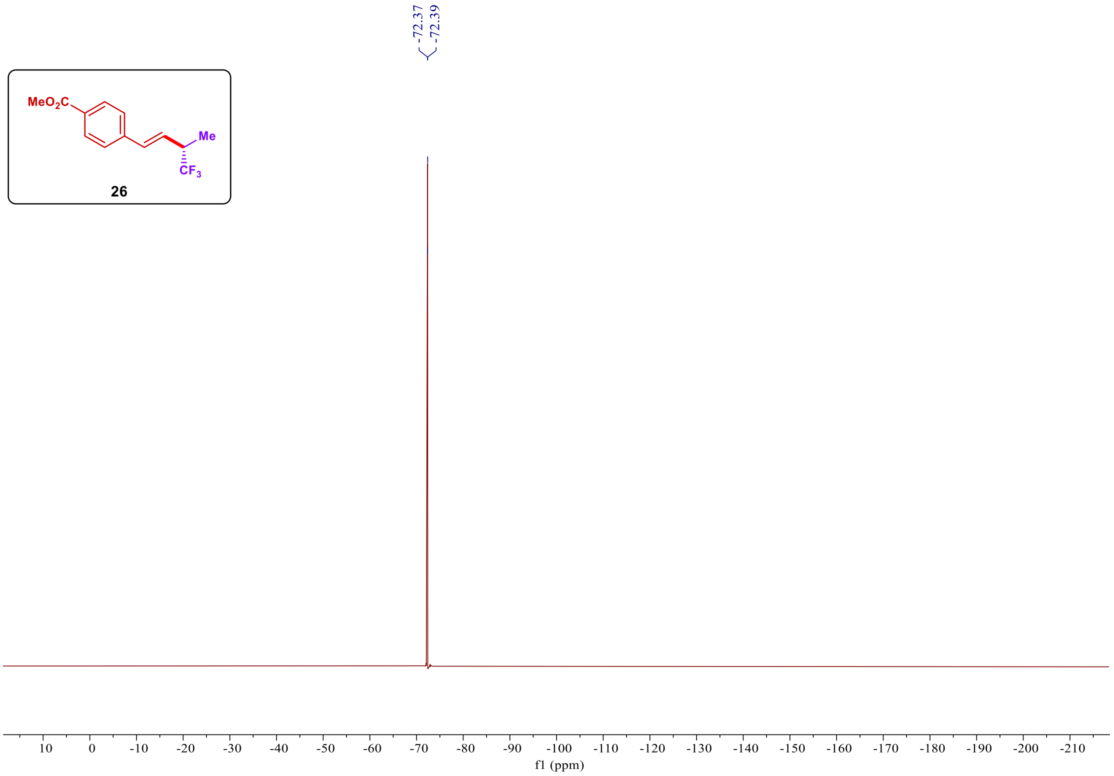

Supplementary Figure 96. ^{19}F NMR Spectrum of Compound **26** (376 MHz, $CDCl_3$)

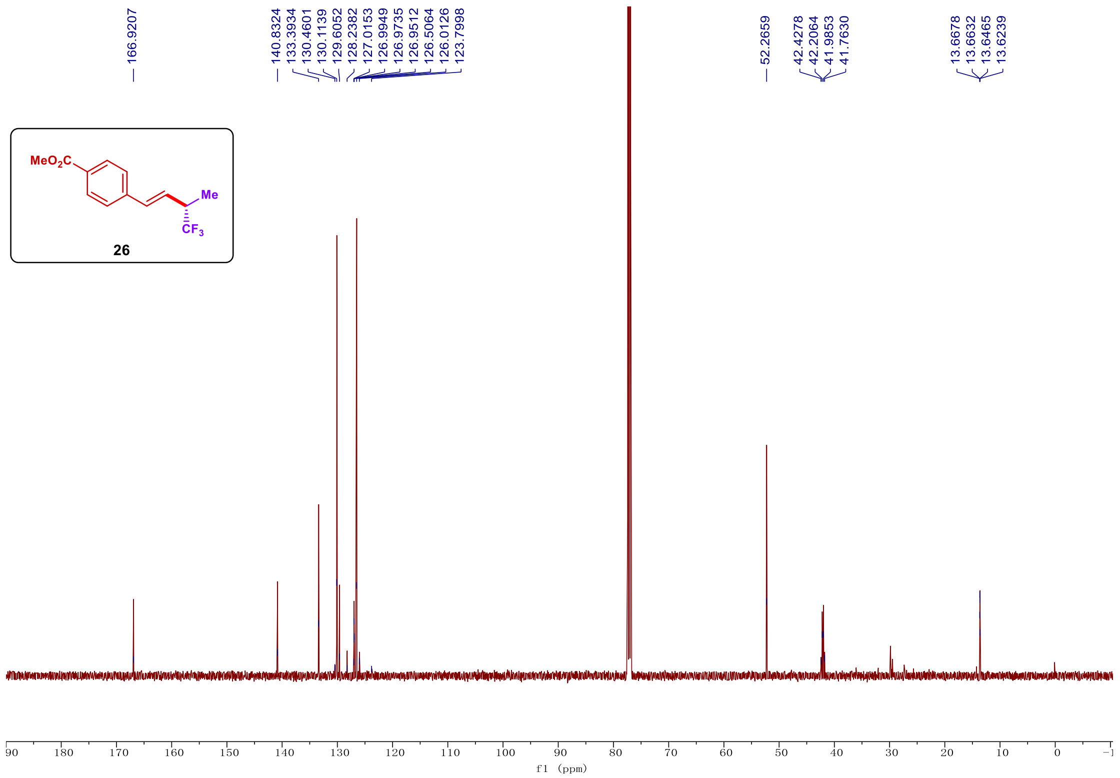

Supplementary Figure 97. ^{13}C NMR Spectrum of Compound **26** (126 MHz, $CDCl_3$)

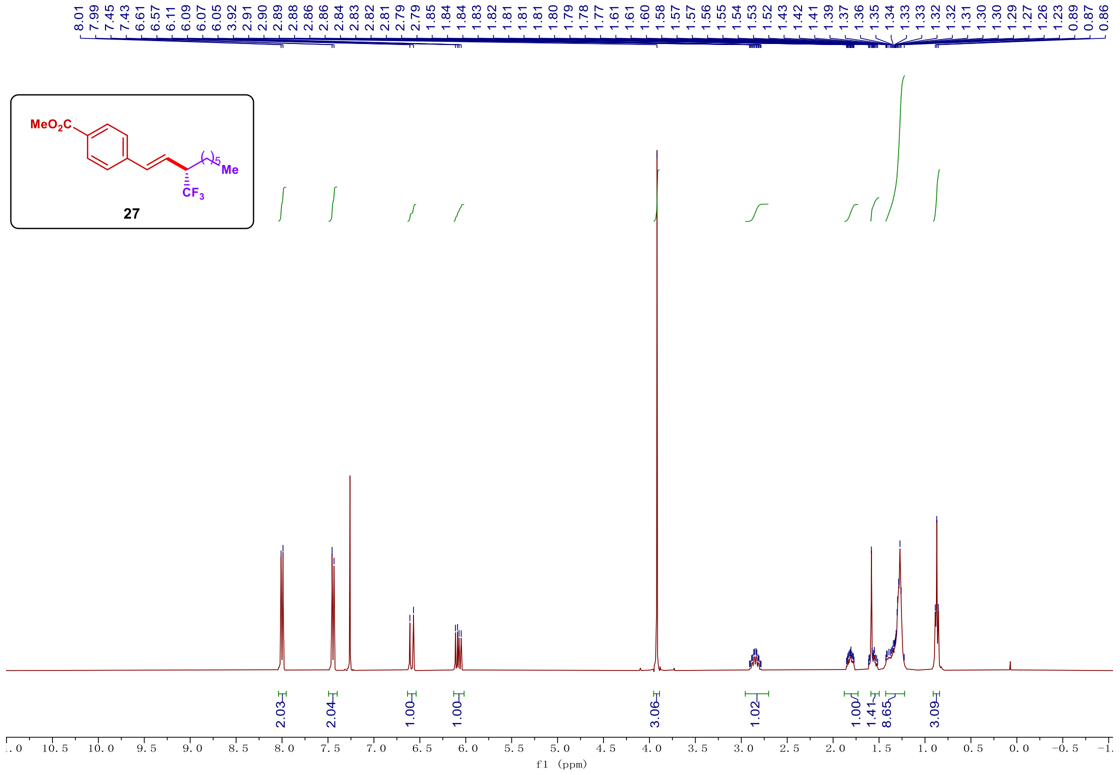

Supplementary Figure 98. ^{1}H NMR Spectrum of Compound **27** (400 MHz, $CDCl_3$)

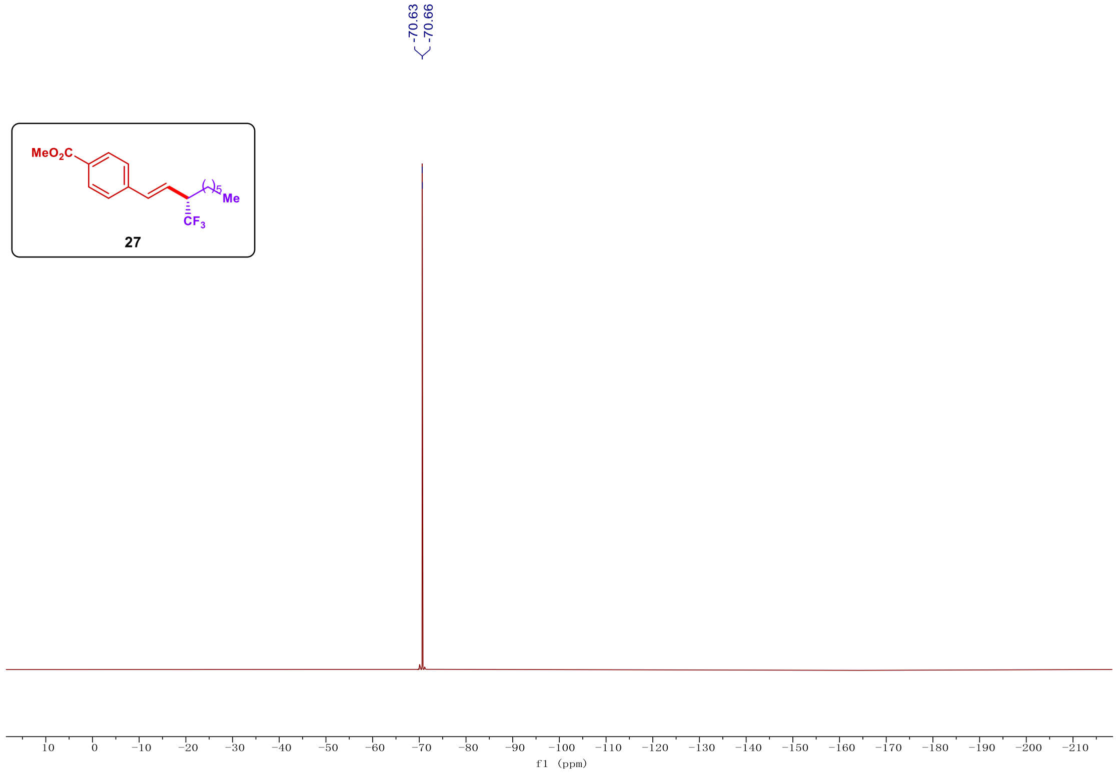

Supplementary Figure 99. ^{19}F NMR Spectrum of Compound **27** (376 MHz, $CDCl_3$)

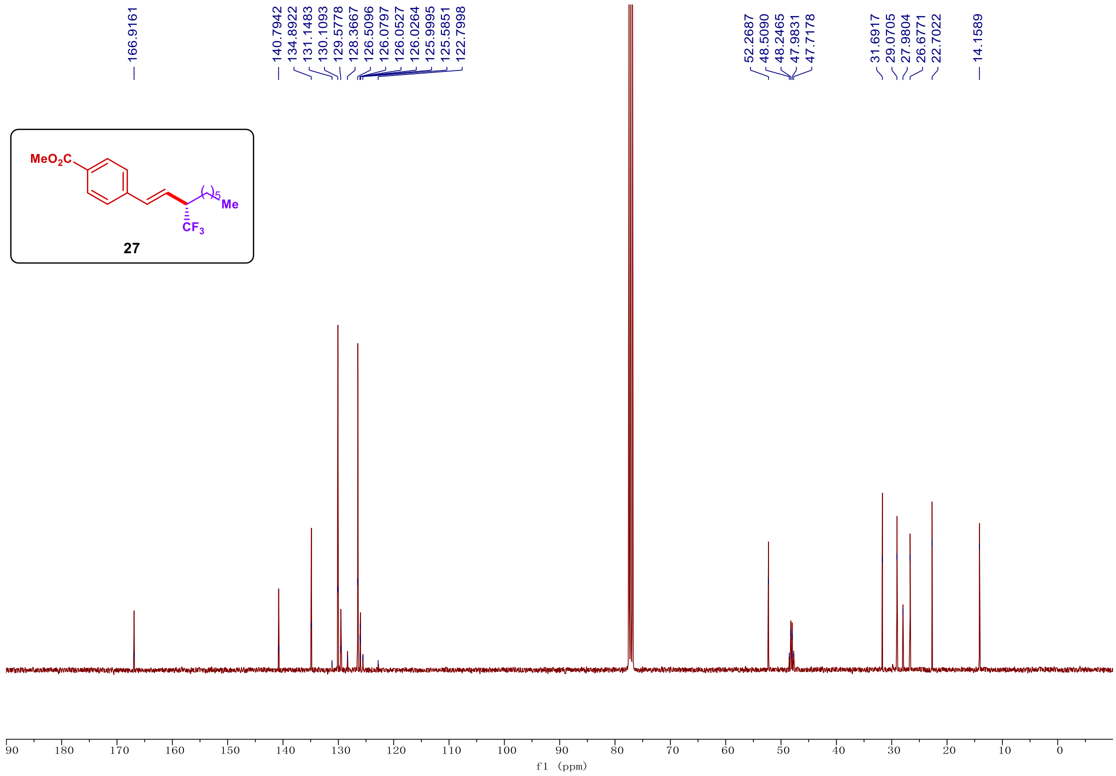

Supplementary Figure 100. ^{13}C NMR Spectrum of Compound **27** (101 MHz, $CDCl_3$)

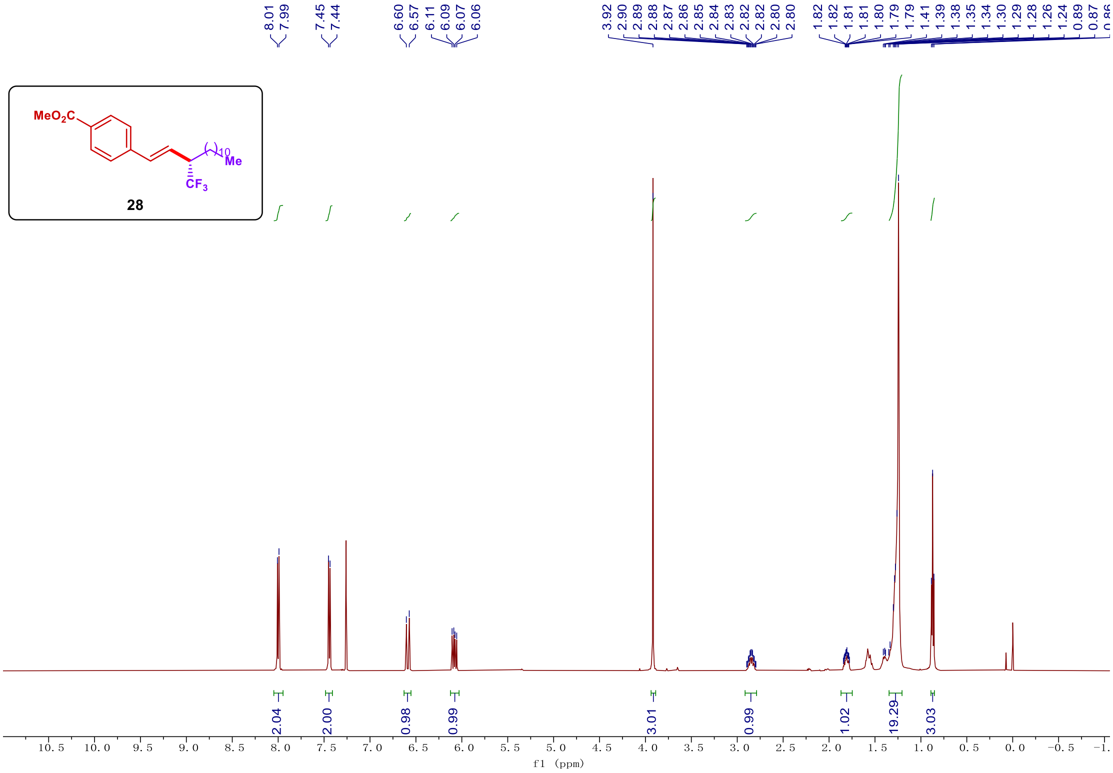

Supplementary Figure 101. ^{1}H NMR Spectrum of Compound **28** (500 MHz, $CDCl_3$)

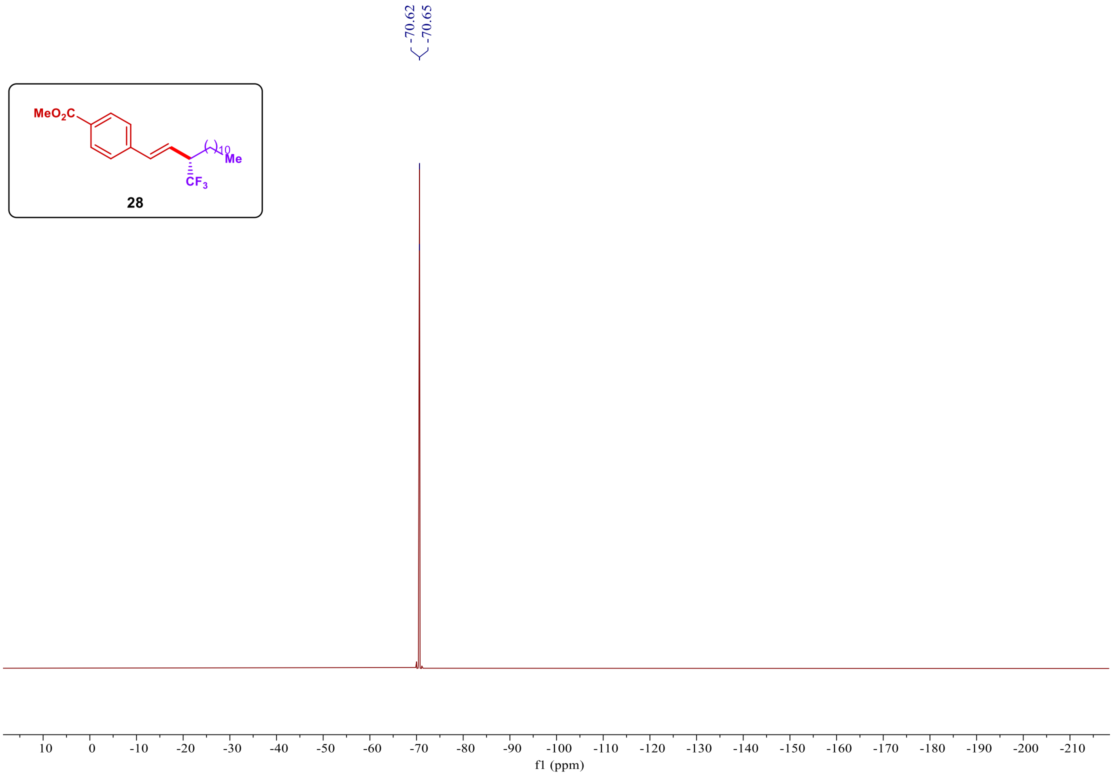

Supplementary Figure 102. ^{19}F NMR Spectrum of Compound **28** (376 MHz, $CDCl_3$)

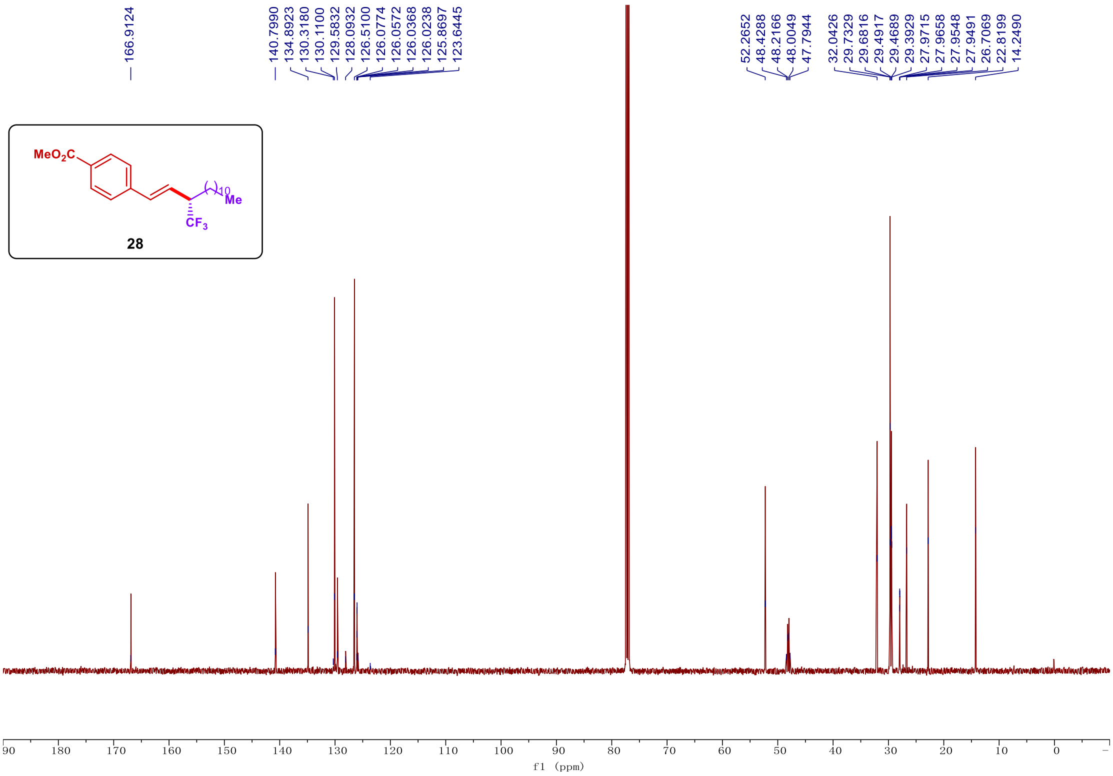

Supplementary Figure 103. ^{13}C NMR Spectrum of Compound **28** (126 MHz, $CDCl_3$)

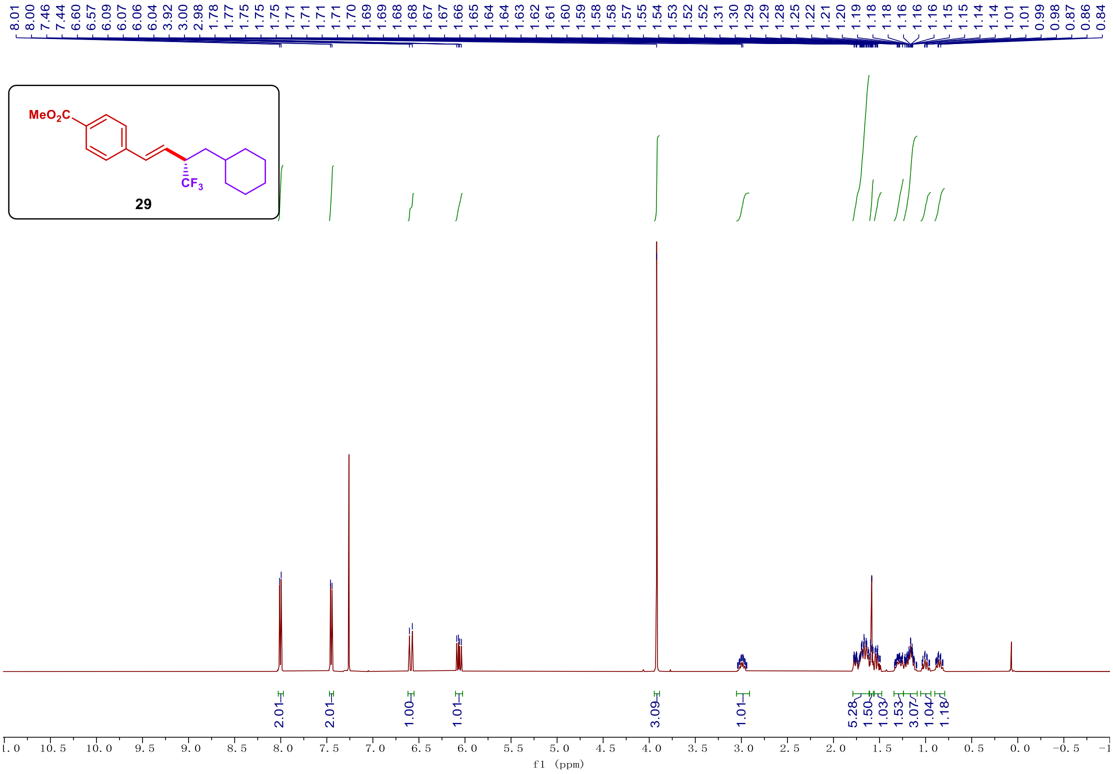

Supplementary Figure 104. ^{1}H NMR Spectrum of Compound **29** (500 MHz, $CDCl_3$)

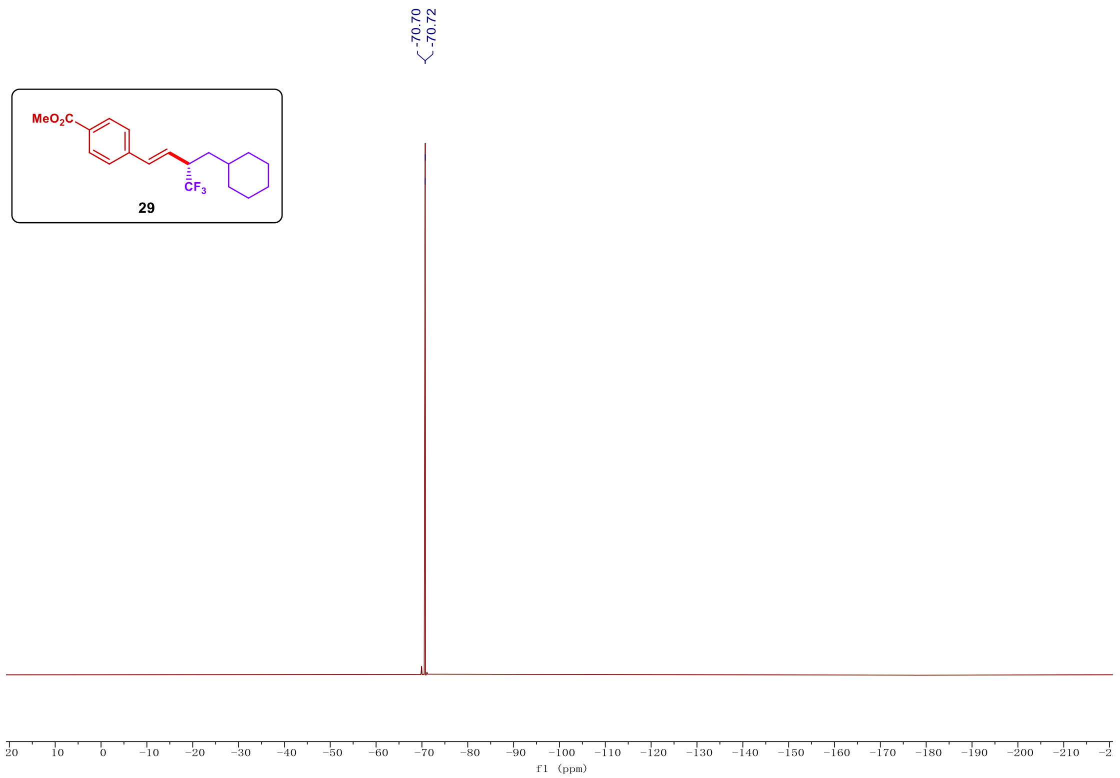

Supplementary Figure 105. ^{19}F NMR Spectrum of Compound **29** (471 MHz, $CDCl_3$)

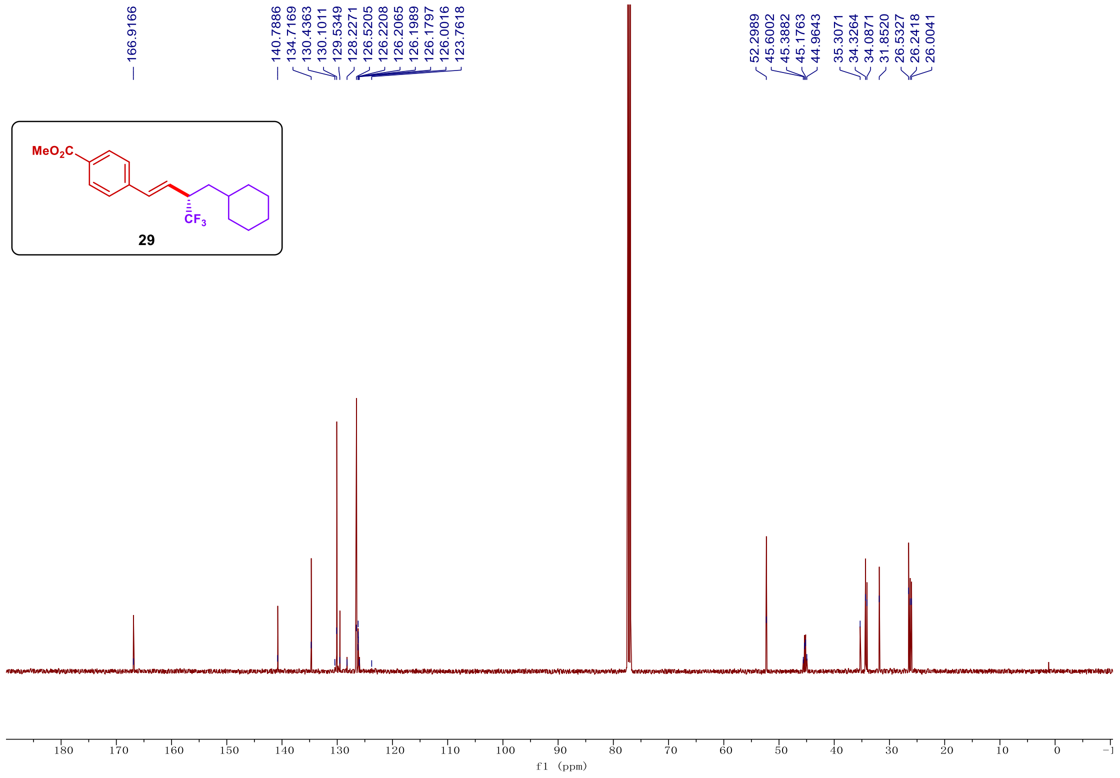

Supplementary Figure 106. ^{13}C NMR Spectrum of Compound **29** (126 MHz, $CDCl_3$)

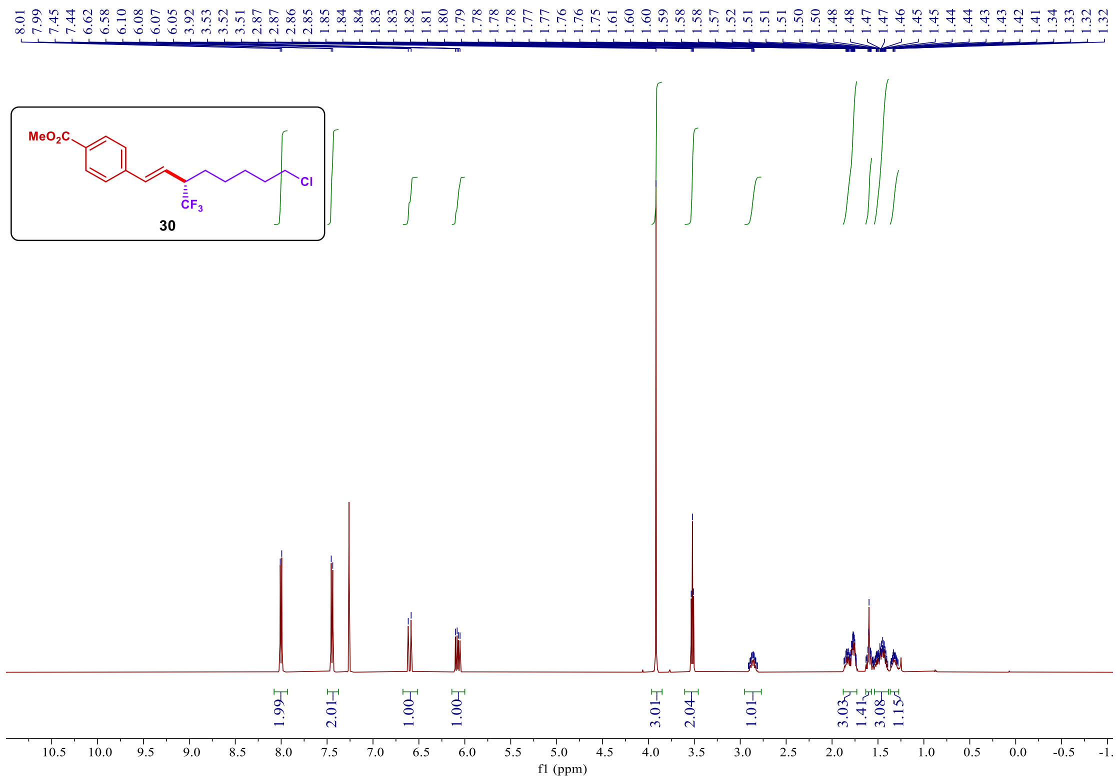

Supplementary Figure 107. ^{1}H NMR Spectrum of Compound **30** (500 MHz, $CDCl_3$)

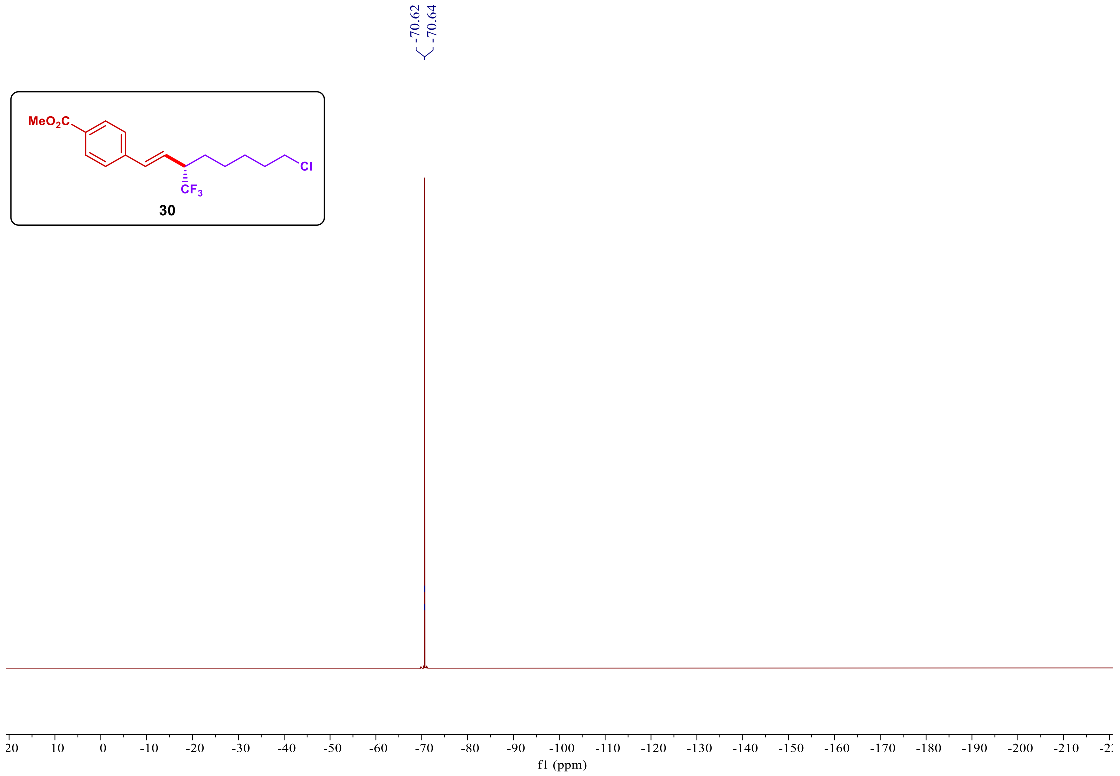

Supplementary Figure 108. ^{19}F NMR Spectrum of Compound **30** (471 MHz, $CDCl_3$)

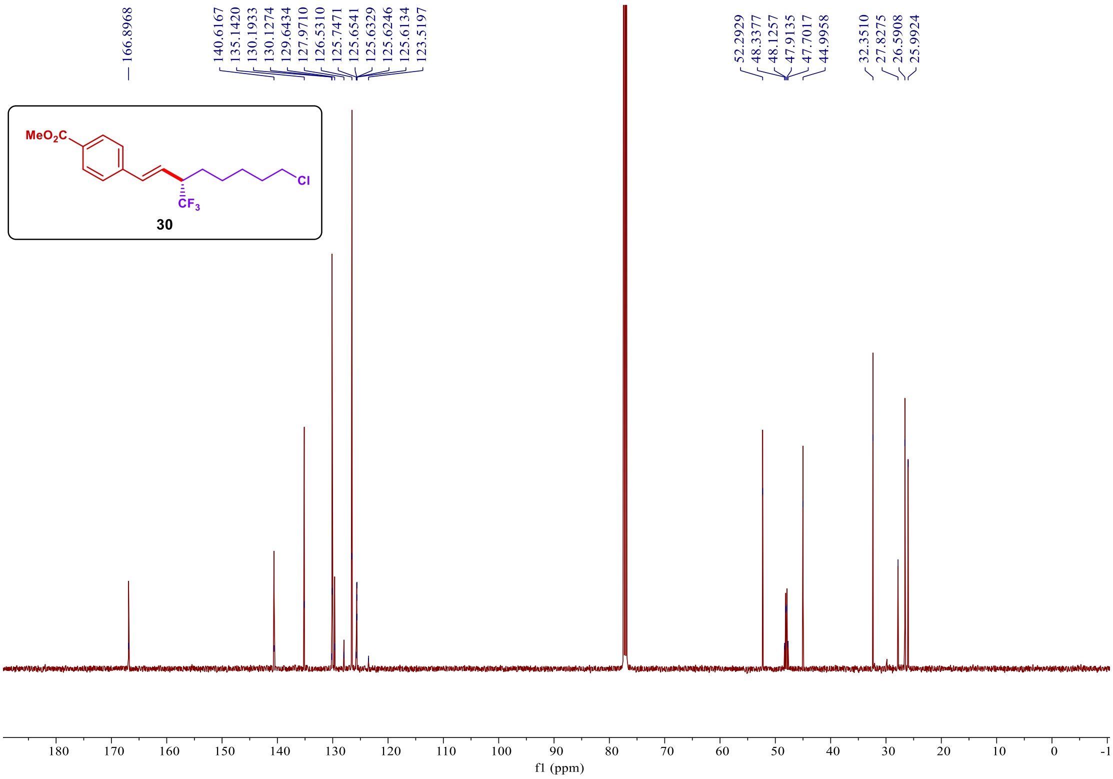

Supplementary Figure 109. ^{13}C NMR Spectrum of Compound **30** (126 MHz, $CDCl_3$)

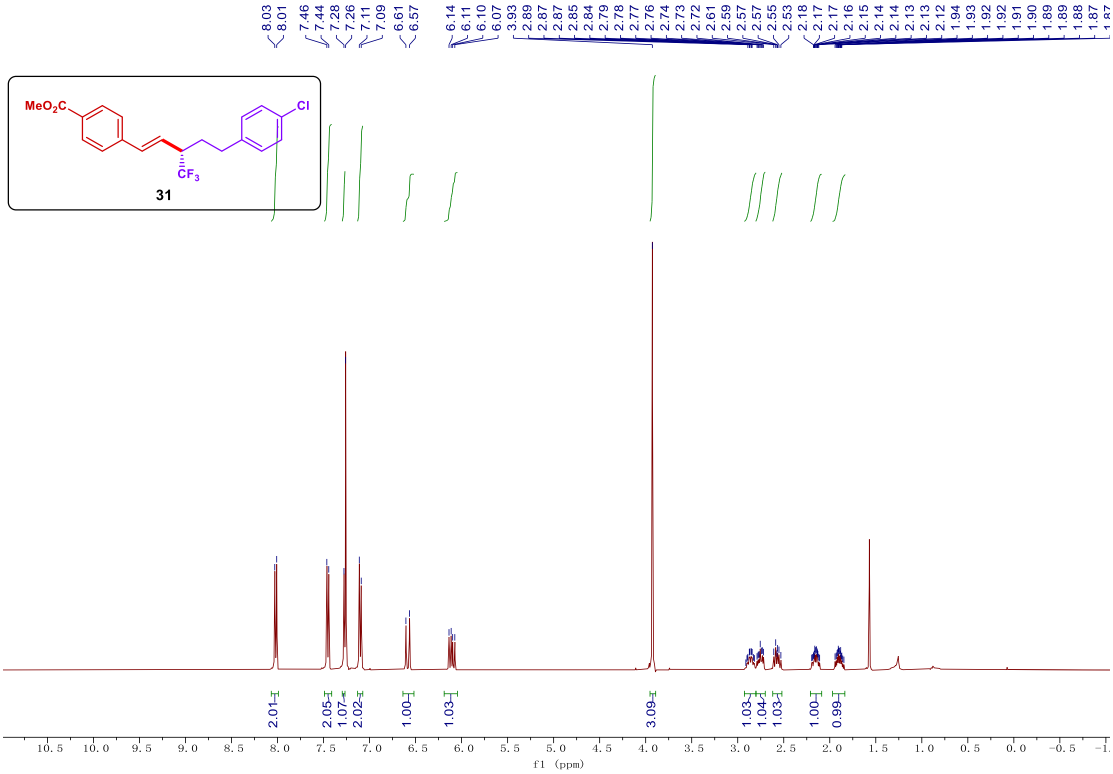

Supplementary Figure 110. ^{1}H NMR Spectrum of Compound **31** (400 MHz, $CDCl_3$)

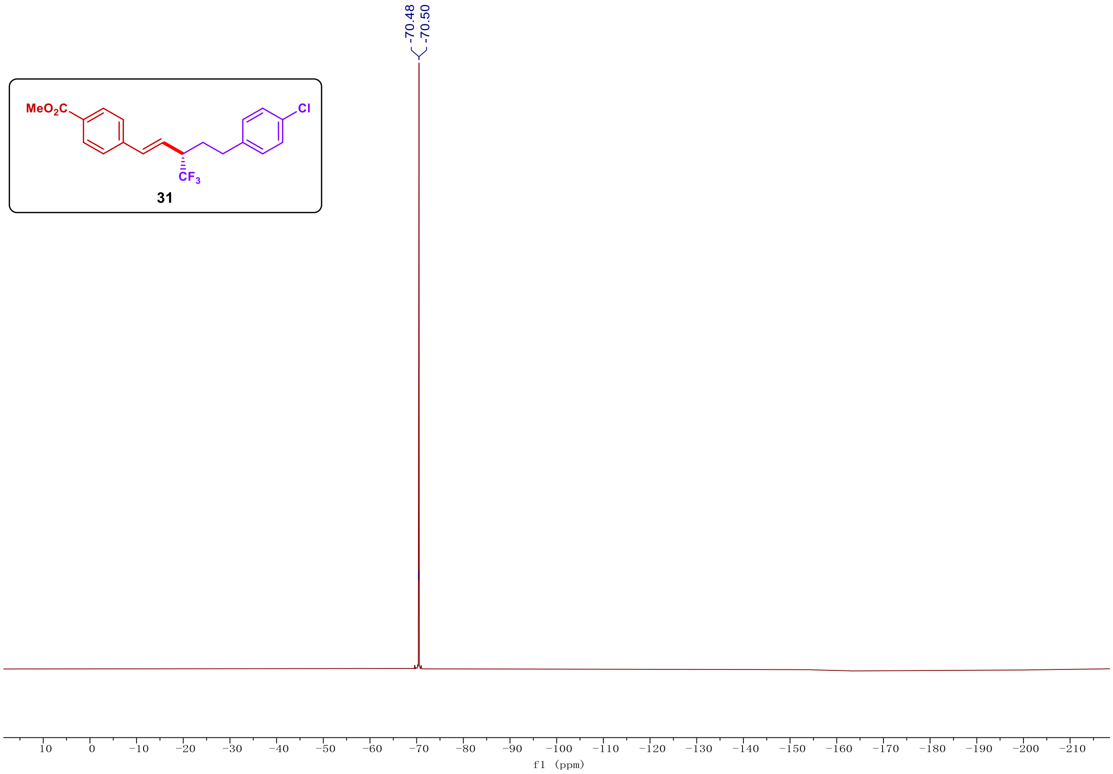

Supplementary Figure 111. ^{19}F NMR Spectrum of Compound **31** (376 MHz, $CDCl_3$)

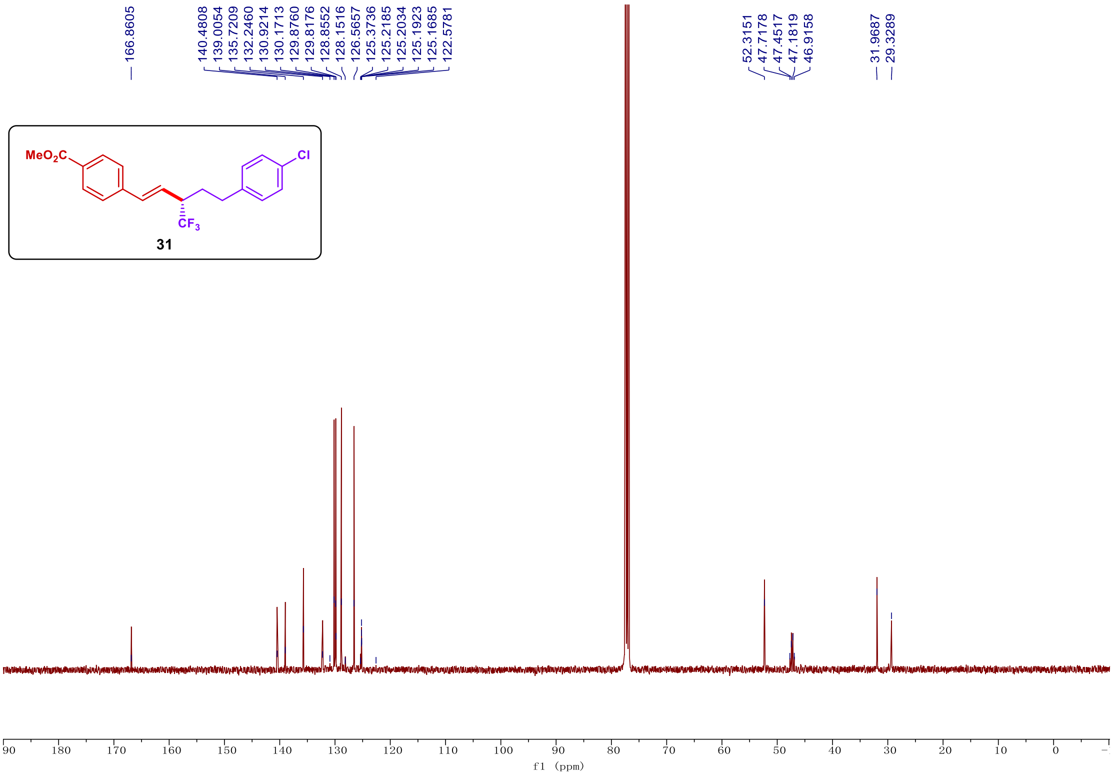

Supplementary Figure 112. ^{13}C NMR Spectrum of Compound **31** (101 MHz, $CDCl_3$)

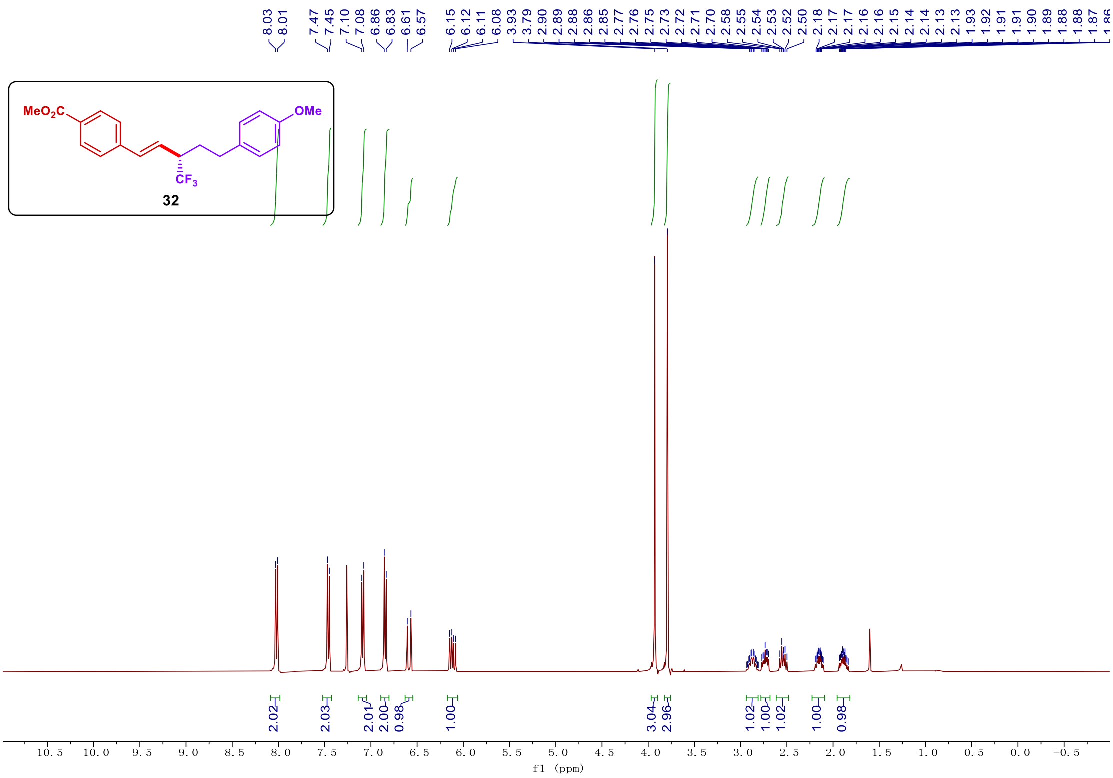

Supplementary Figure 113. ^{1}H NMR Spectrum of Compound **32** (400 MHz, $CDCl_3$)

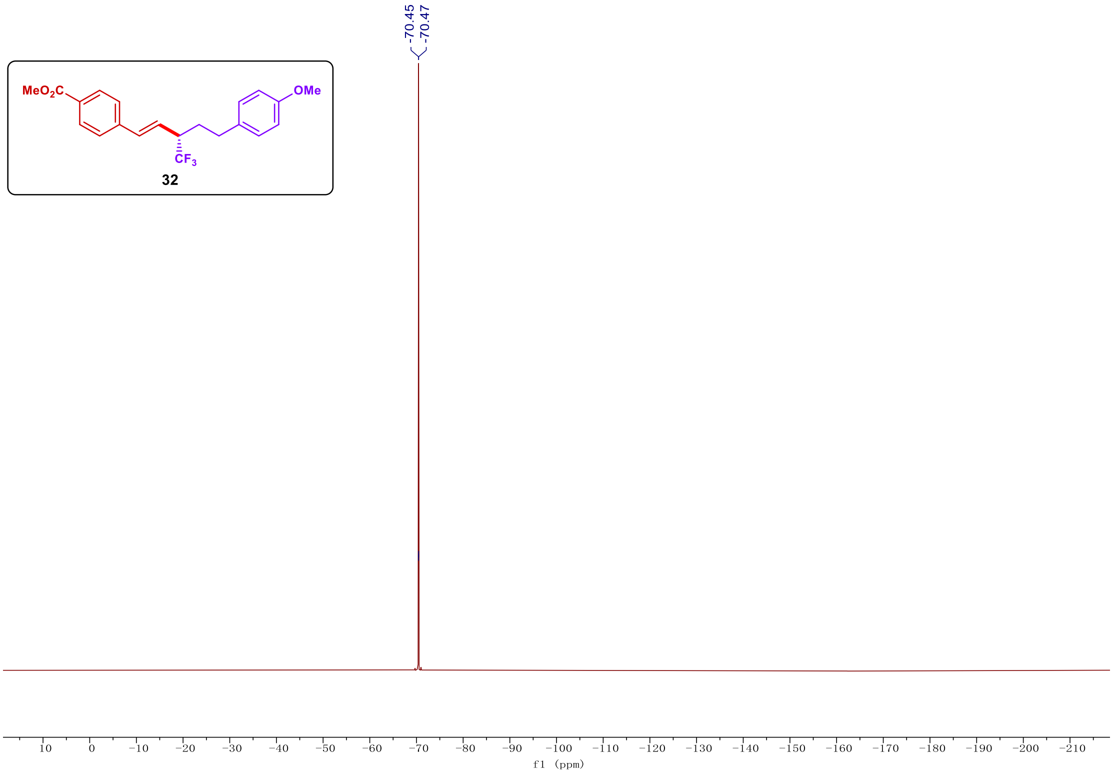

Supplementary Figure 114. ^{19}F NMR Spectrum of Compound **32** (376 MHz, $CDCl_3$)

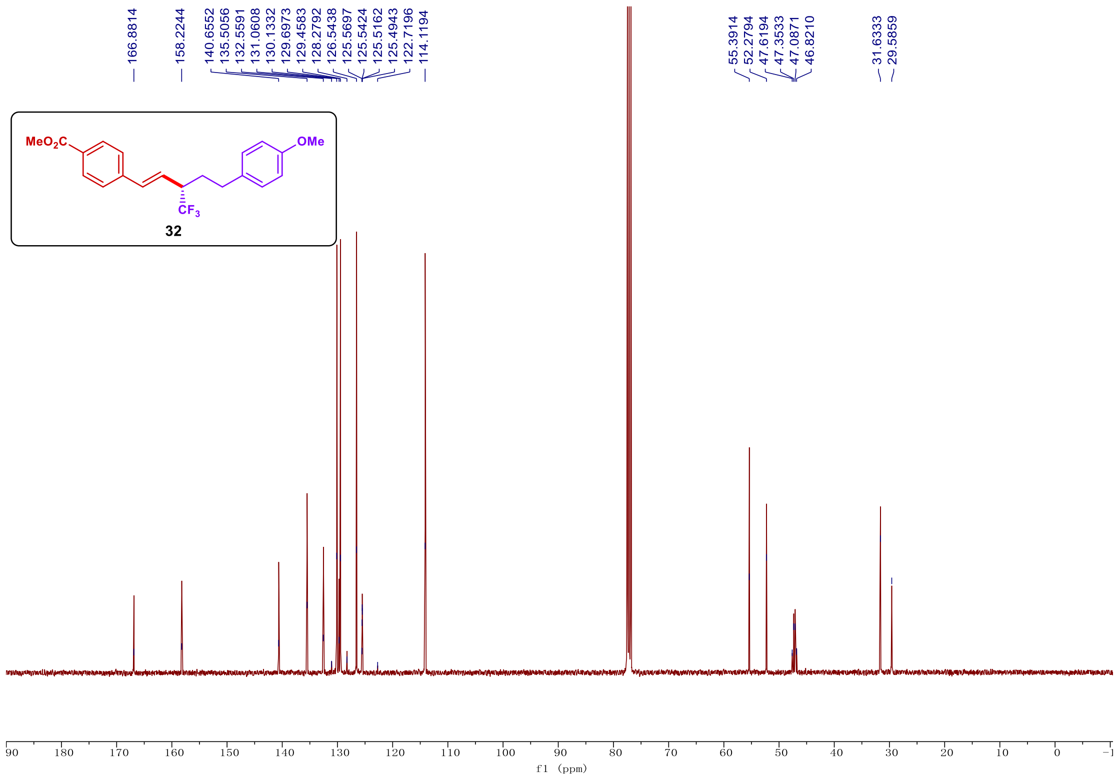

Supplementary Figure 115. ^{13}C NMR Spectrum of Compound **32** (101 MHz, $CDCl_3$)

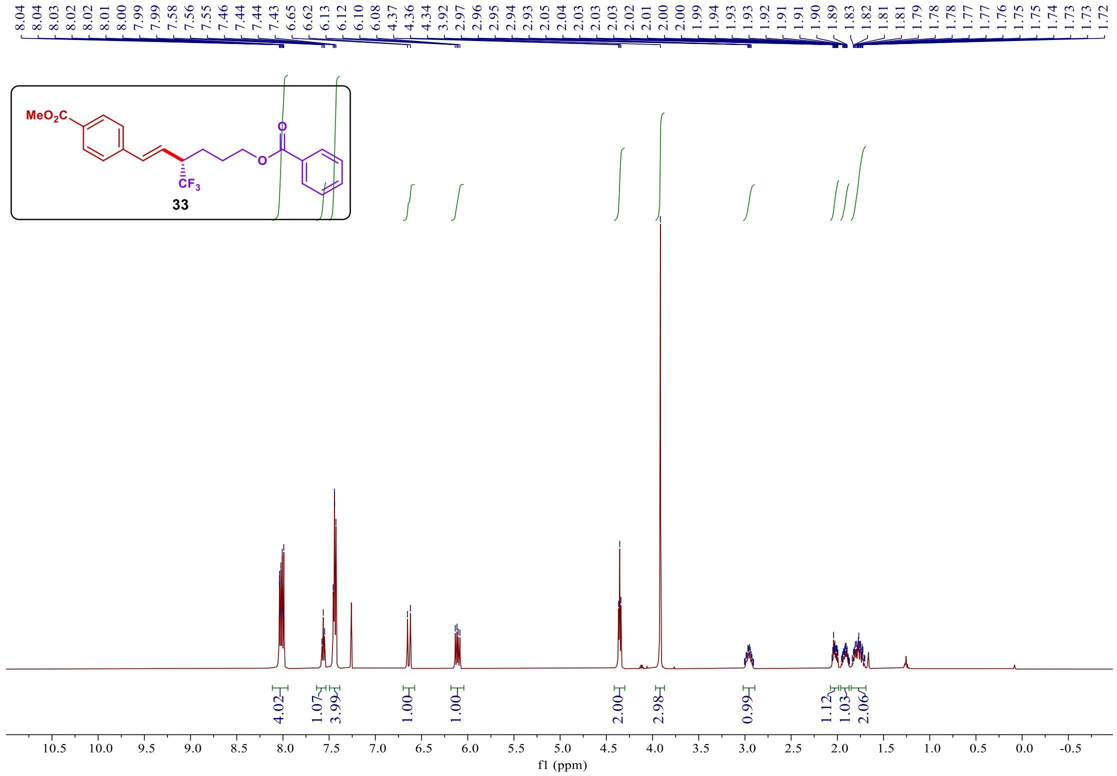

Supplementary Figure 116. ^{1}H NMR Spectrum of Compound **33** (500 MHz, $CDCl_3$)

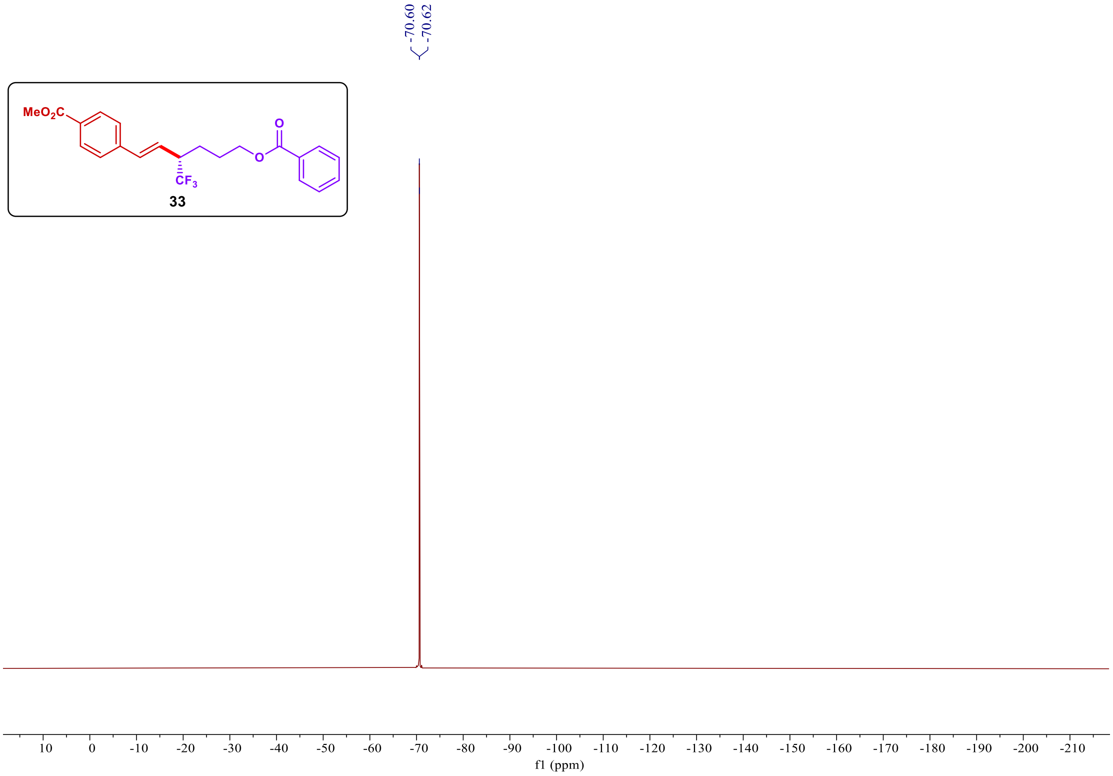

Supplementary Figure 117. ^{19}F NMR Spectrum of Compound **33** (376 MHz, $CDCl_3$)

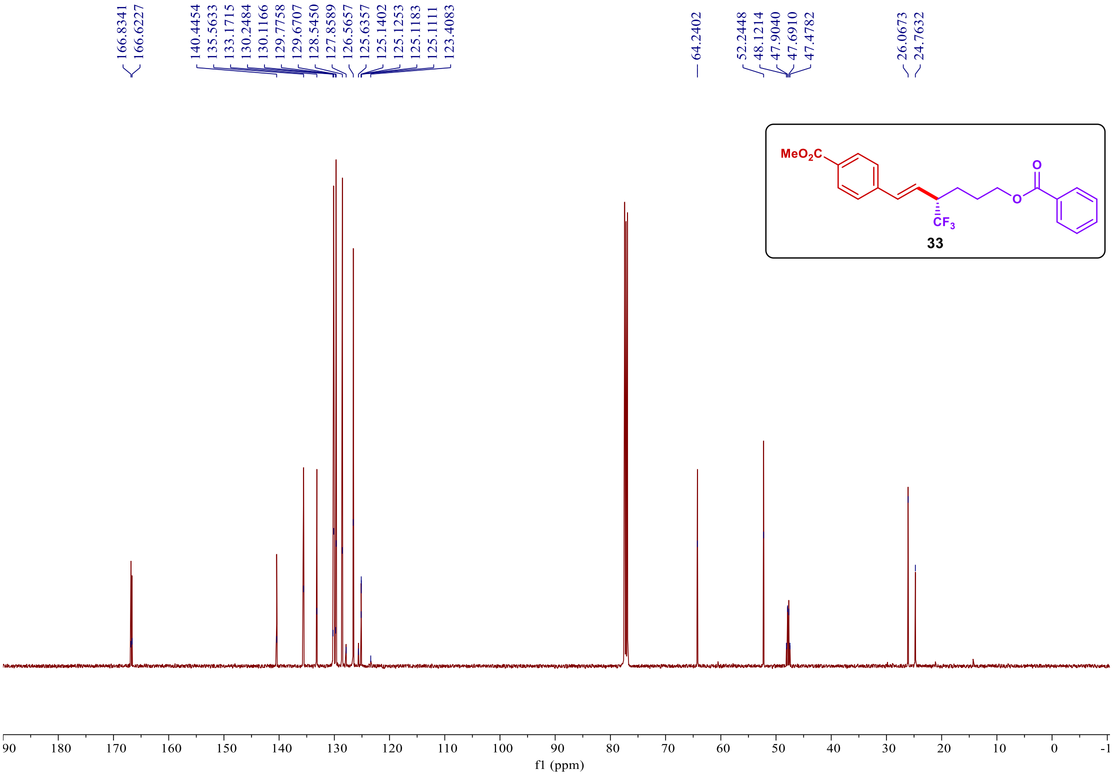

Supplementary Figure 118. ^{13}C NMR Spectrum of Compound **33** (126 MHz, $CDCl_3$)

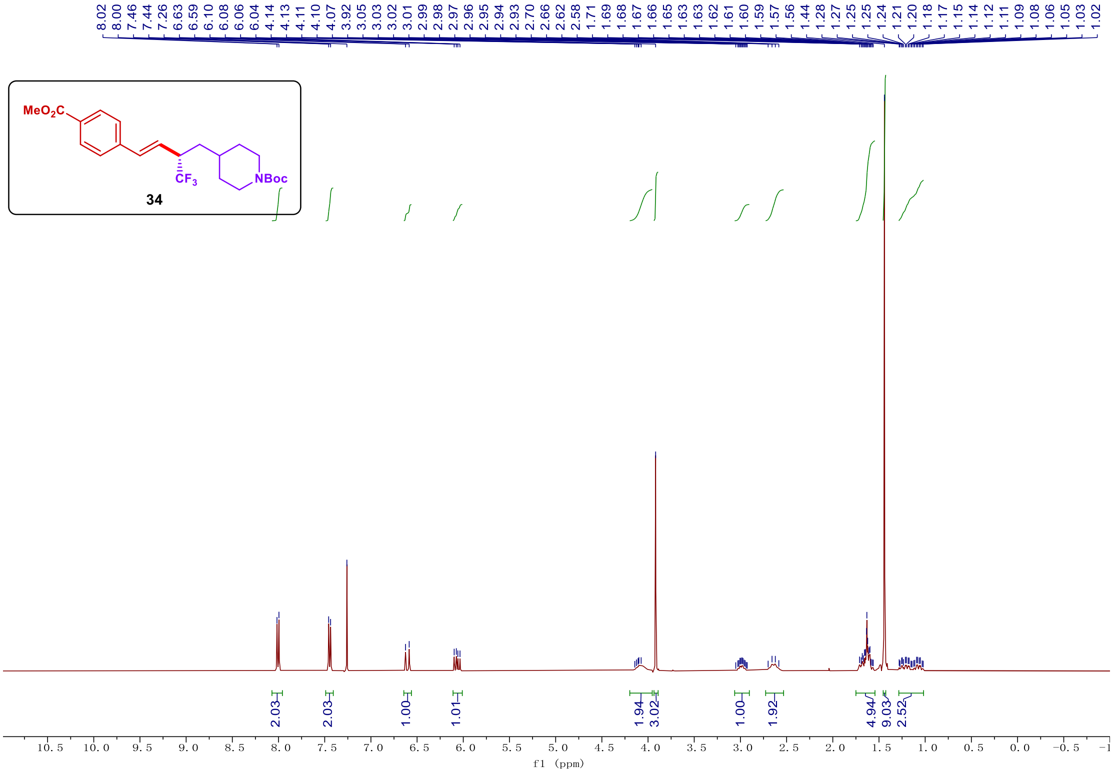

Supplementary Figure 119. ^{1}H NMR Spectrum of Compound **34** (400 MHz, $CDCl_3$)

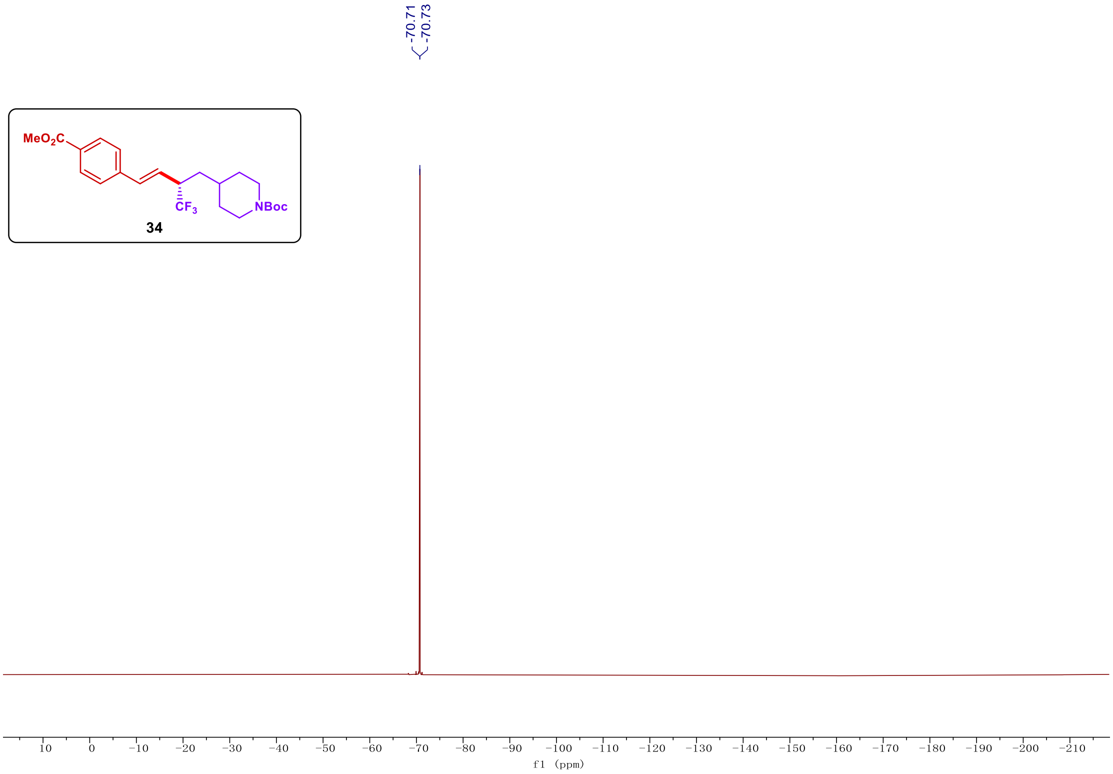

Supplementary Figure 120. ^{19}F NMR Spectrum of Compound **34** (376 MHz, $CDCl_3$)

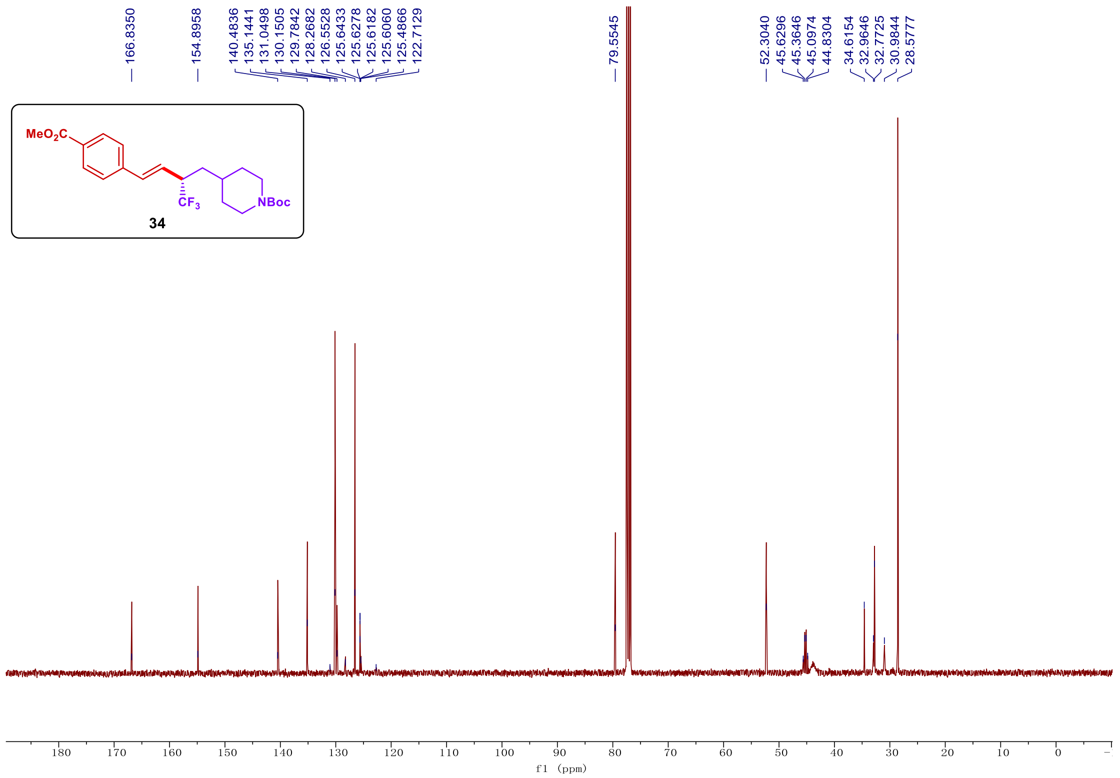

Supplementary Figure 121. ^{13}C NMR Spectrum of Compound **34** (101 MHz, $CDCl_3$)

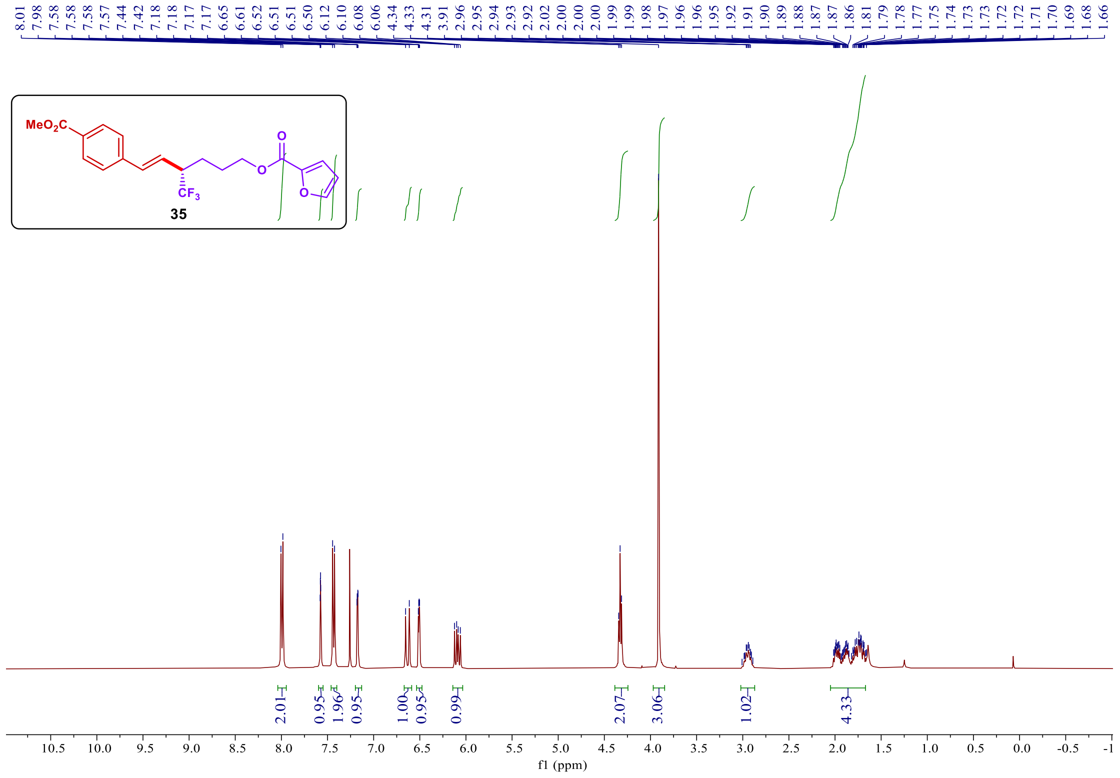

Supplementary Figure 122. ^{1}H NMR Spectrum of Compound **35** (400 MHz, $CDCl_3$)

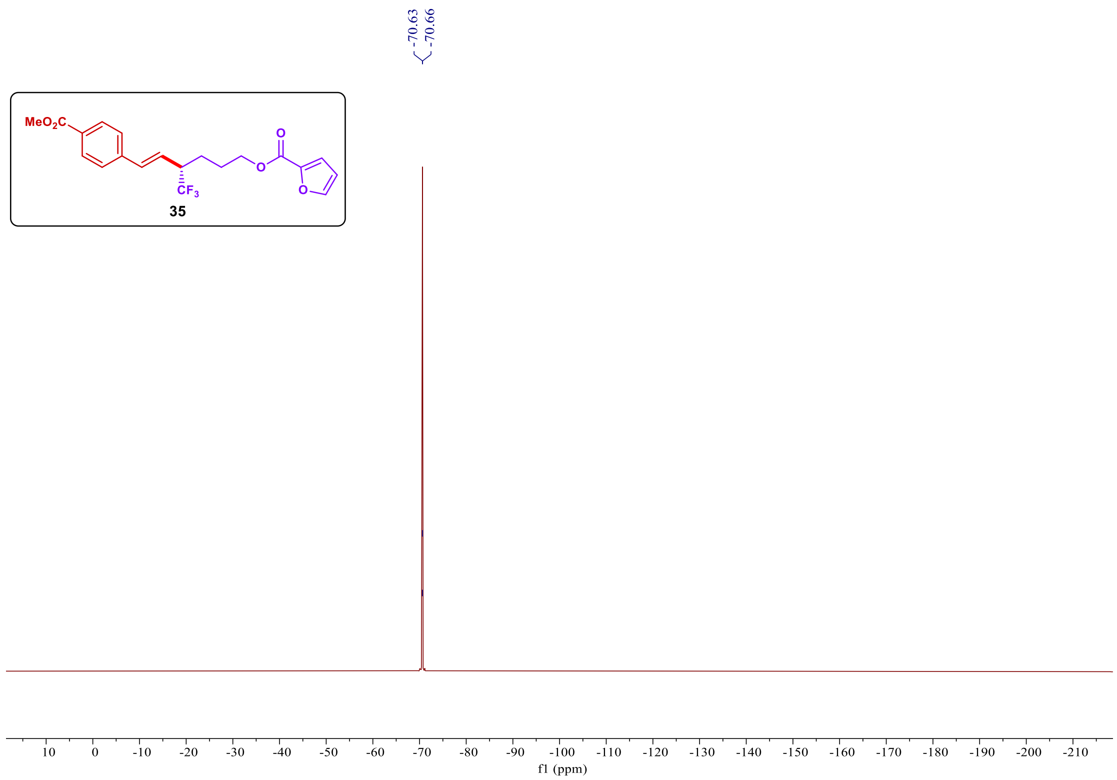

Supplementary Figure 123. ^{19}F NMR Spectrum of Compound **35** (376 MHz, $CDCl_3$)

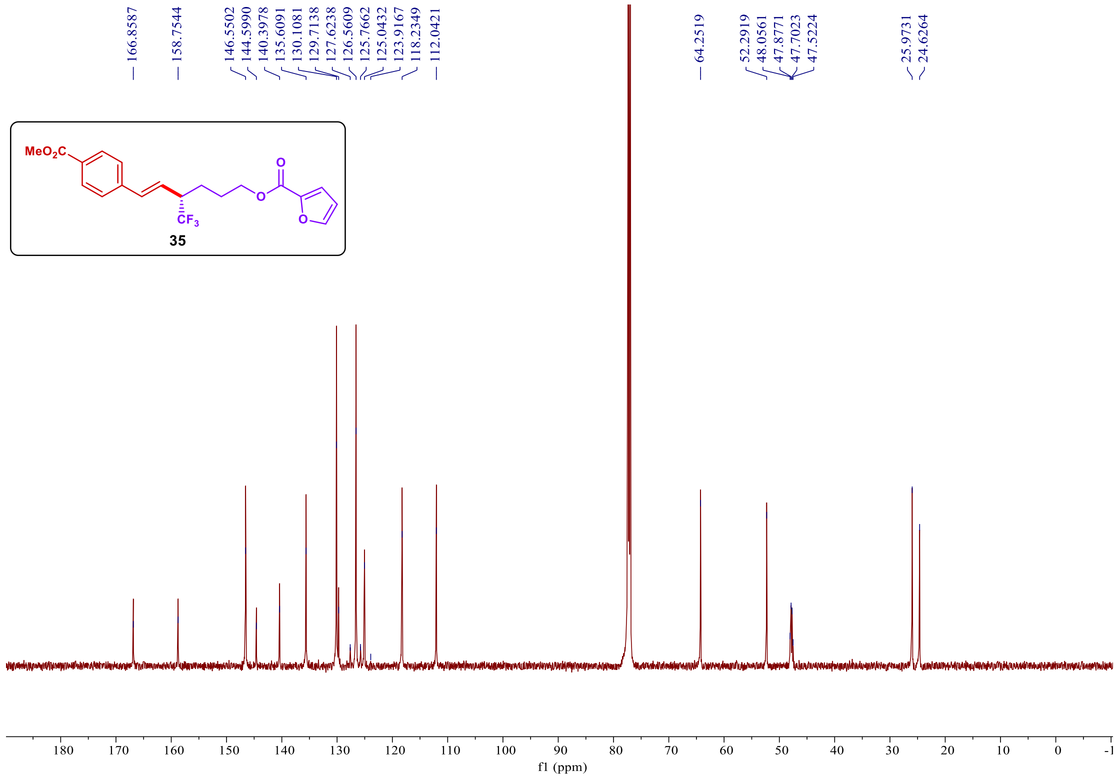

Supplementary Figure 124. ^{13}C NMR Spectrum of Compound **35** (151 MHz, $CDCl_3$)

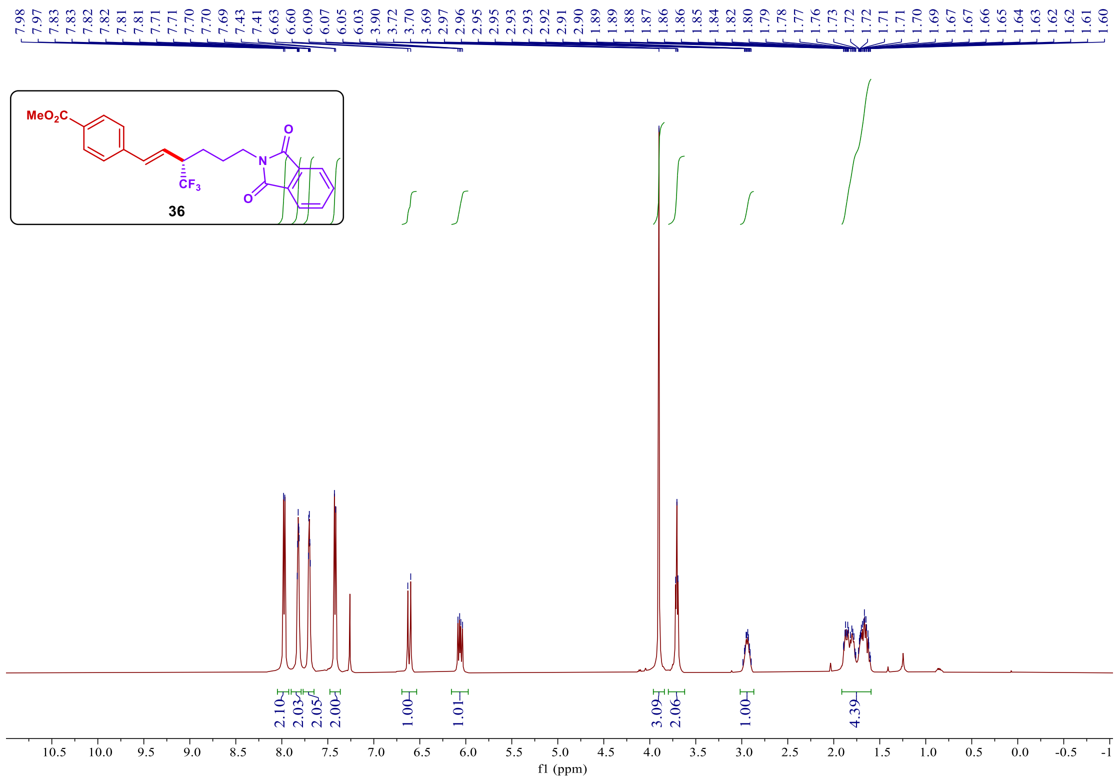

Supplementary Figure 125. ^1H NMR Spectrum of Compound **36** (500 MHz, $CDCl_3$)

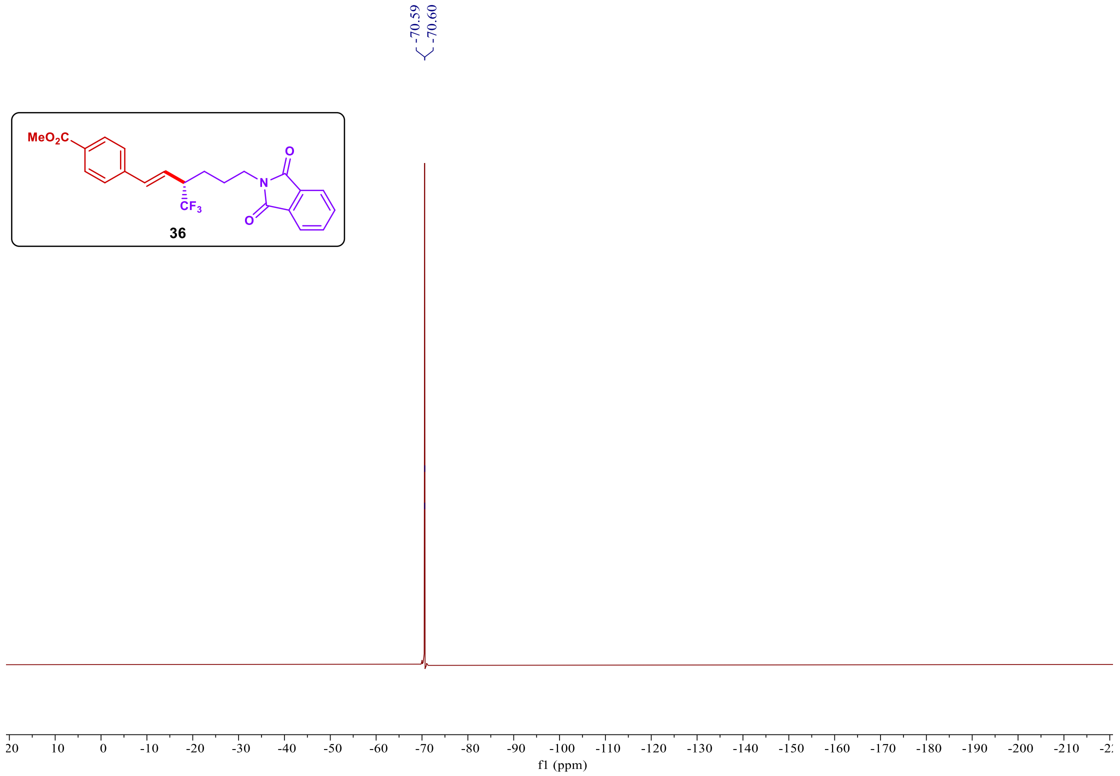

Supplementary Figure 126. ^{19}F NMR Spectrum of Compound **36** (471 MHz, $CDCl_3$)

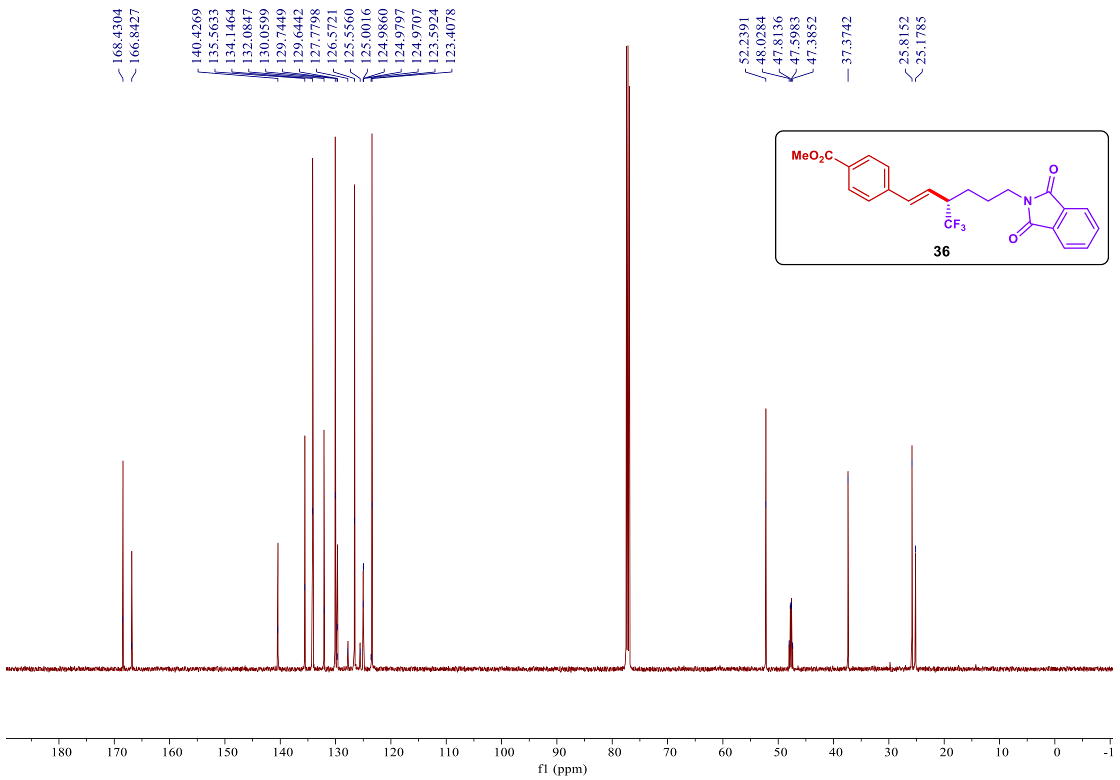

Supplementary Figure 127. ^{13}C NMR Spectrum of Compound **36** (126 MHz, $CDCl_3$)

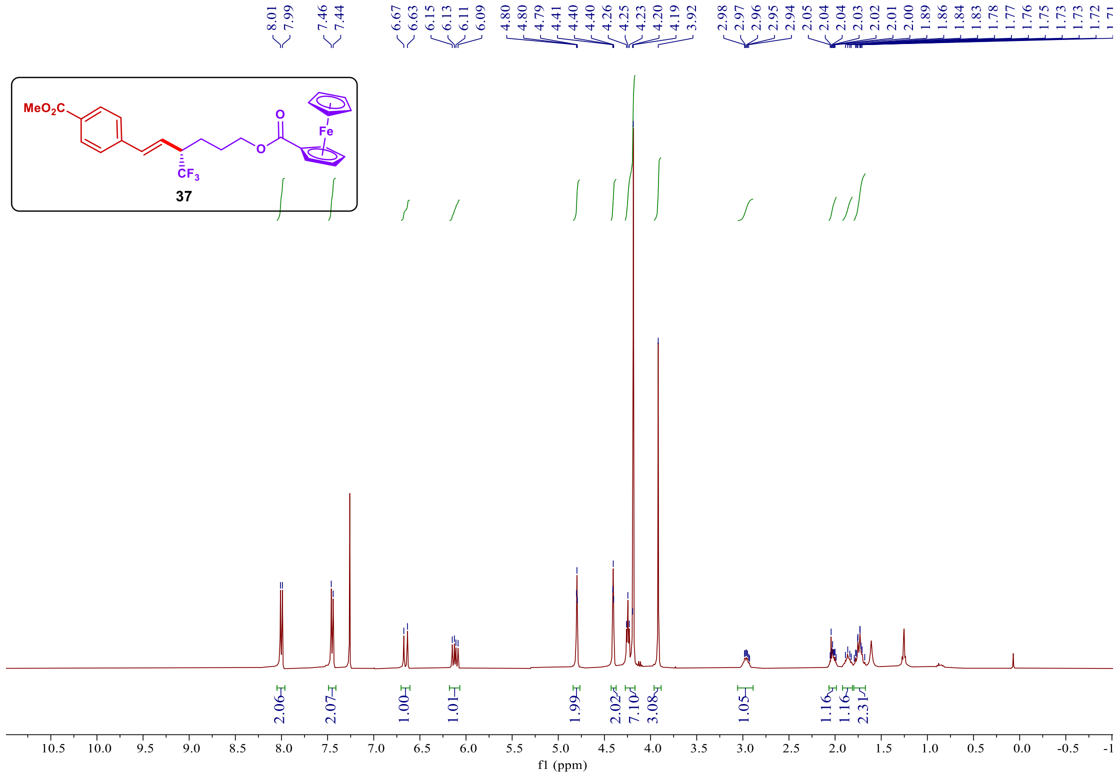

Supplementary Figure 128. ^{1}H NMR Spectrum of Compound **37** (400 MHz, $CDCl_3$)

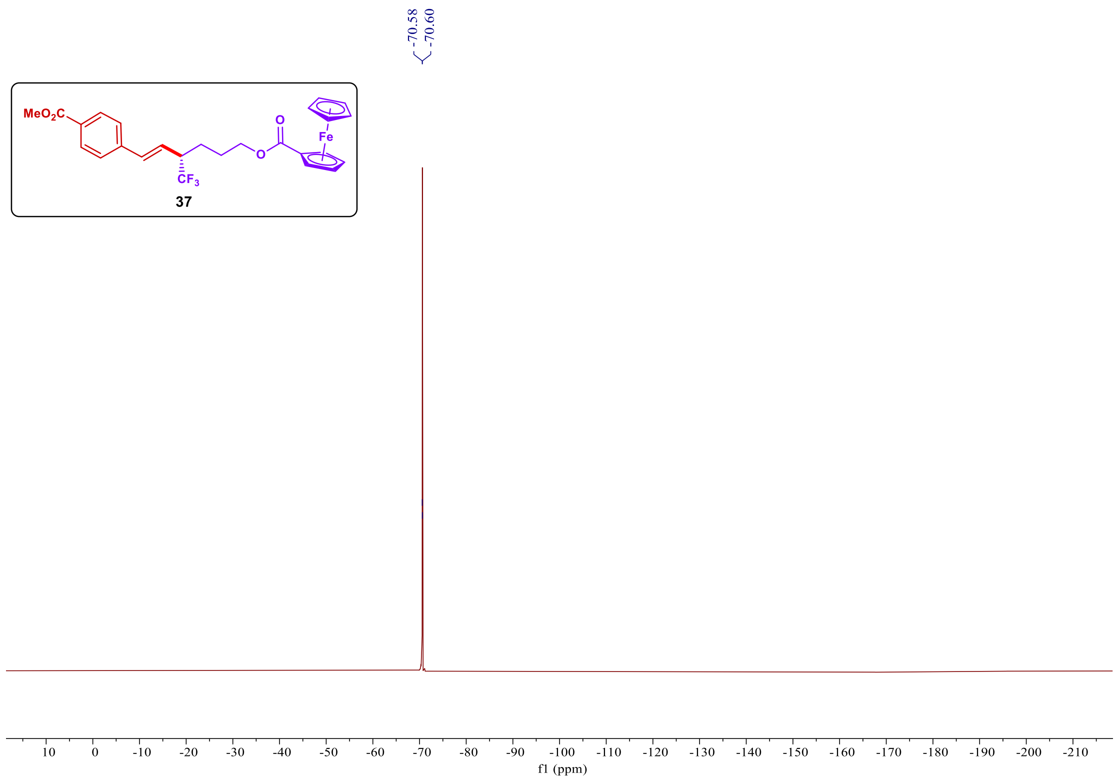

Supplementary Figure 129. ^{19}F NMR Spectrum of Compound **37** (376 MHz, $CDCl_3$)

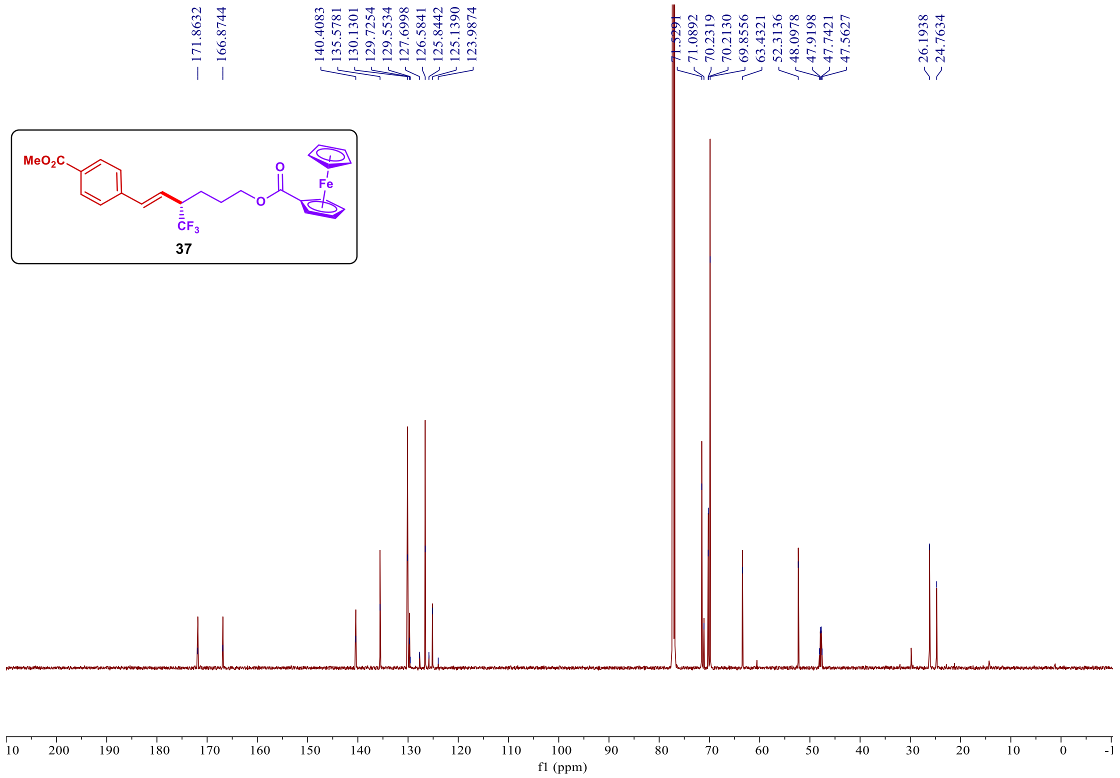

Supplementary Figure 130. ^{13}C NMR Spectrum of Compound **37** (151 MHz, $CDCl_3$)

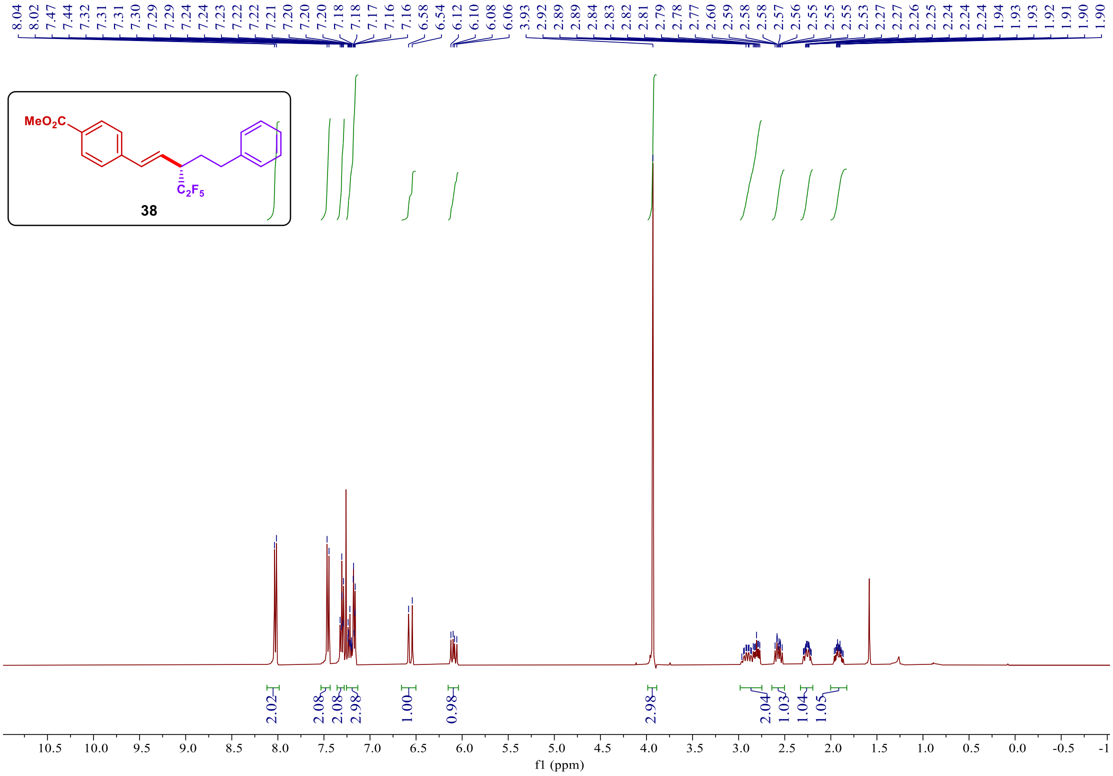

Supplementary Figure 131. ^{1}H NMR Spectrum of Compound **38** (400 MHz, $CDCl_3$)

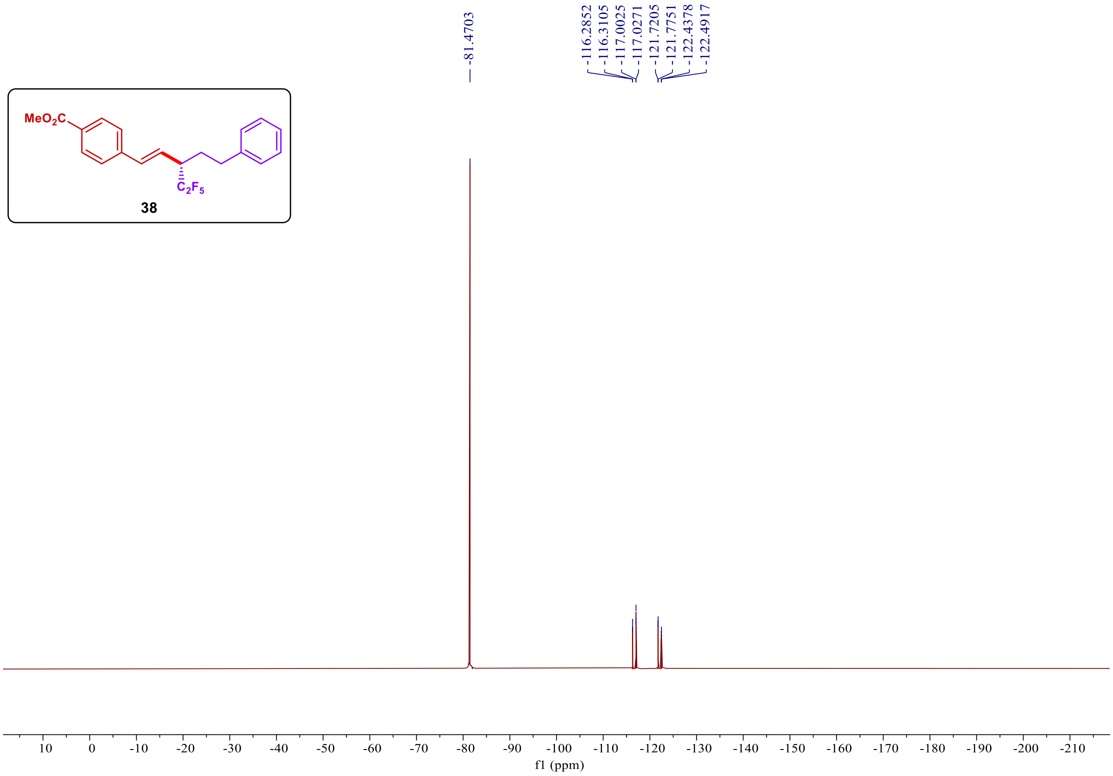

Supplementary Figure 132. ^{19}F NMR Spectrum of Compound **38** (376 MHz, $CDCl_3$)

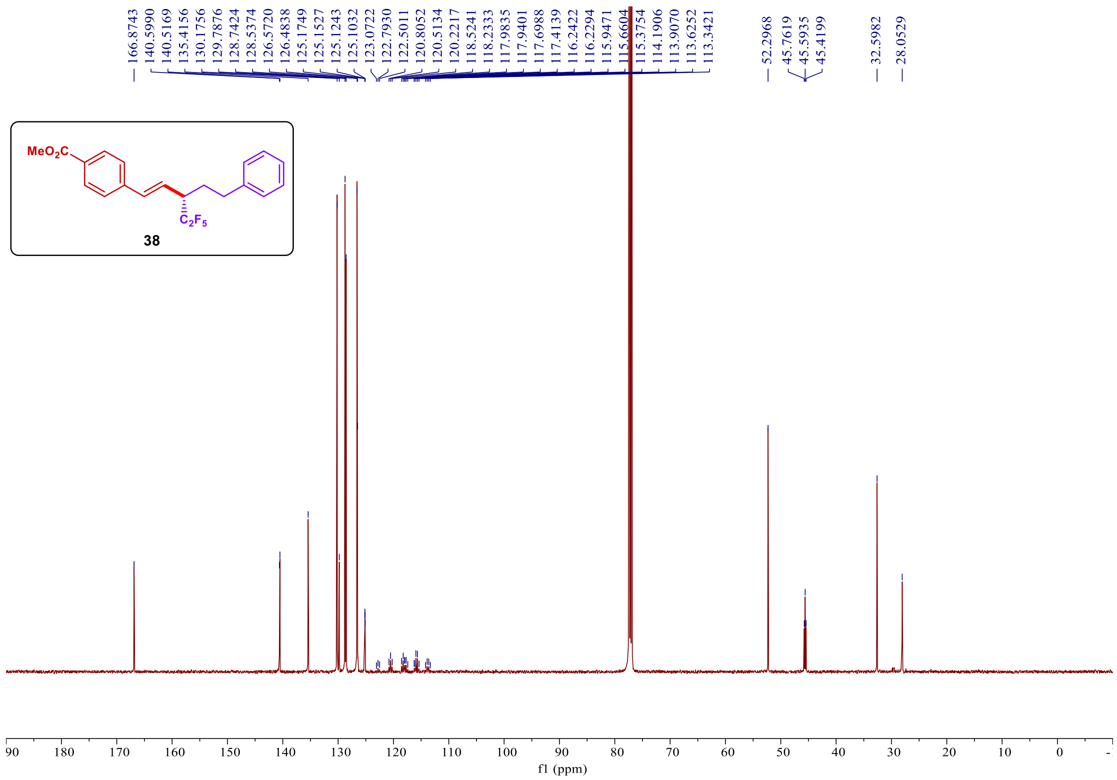

Supplementary Figure 133. ^{13}C NMR Spectrum of Compound **38** (126 MHz, $CDCl_3$)

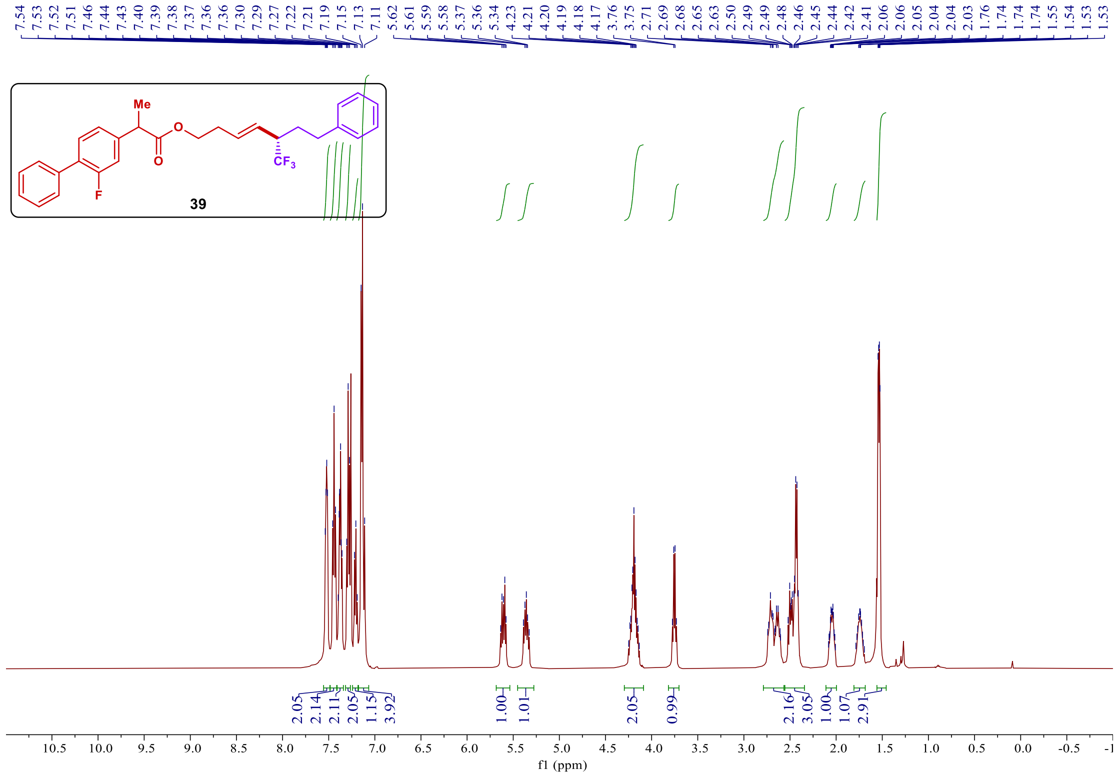

Supplementary Figure 134. ^{1}H NMR Spectrum of Compound **39** (500 MHz, $CDCl_3$)

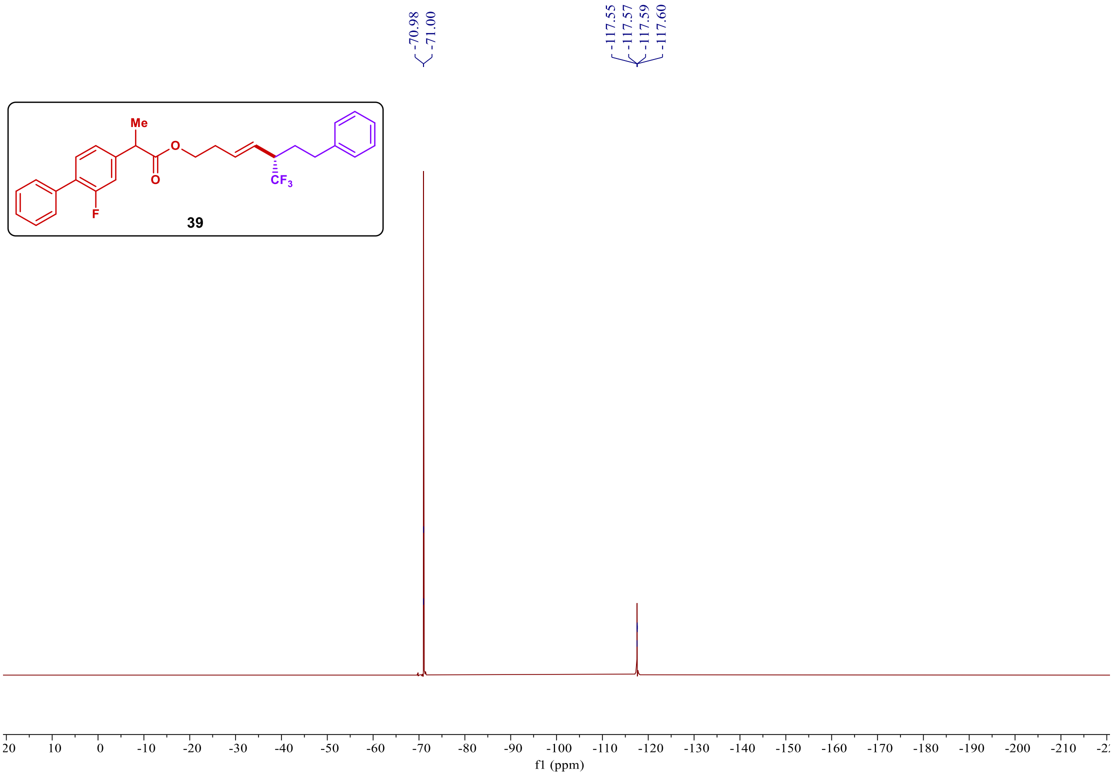

Supplementary Figure 135. ^{19}F NMR Spectrum of Compound **39** (471 MHz, $CDCl_3$)

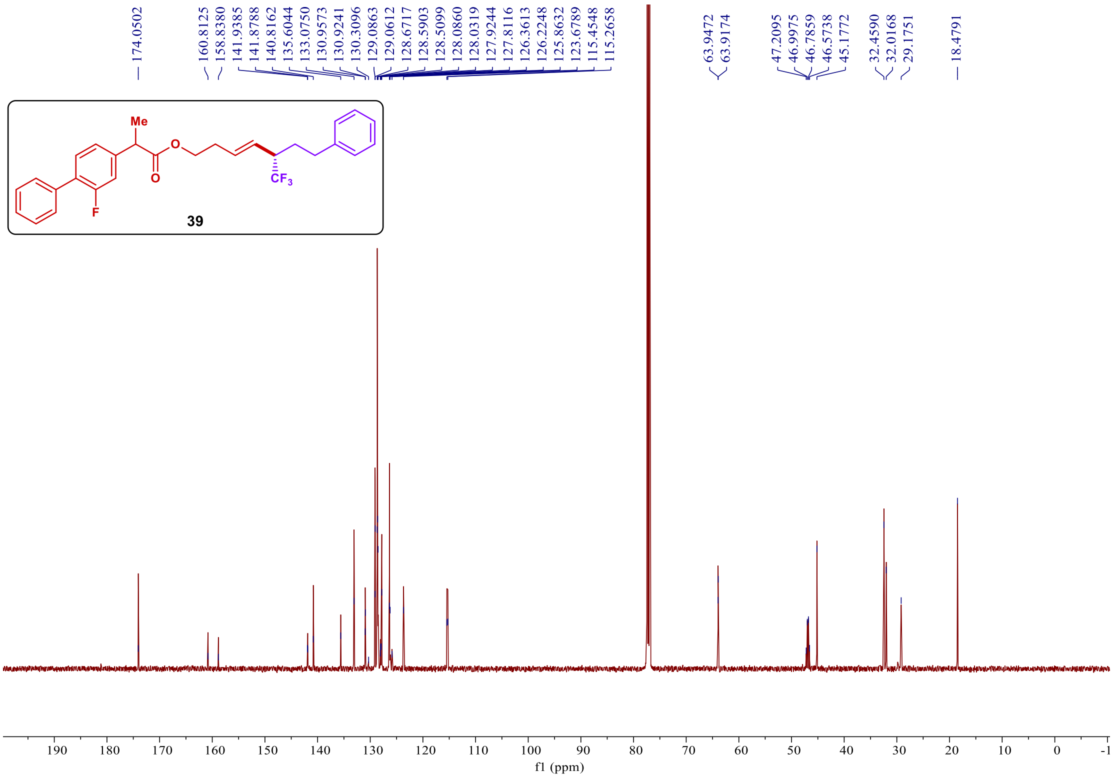

Supplementary Figure 136. ^{13}C NMR Spectrum of Compound **39** (126 MHz, $CDCl_3$)

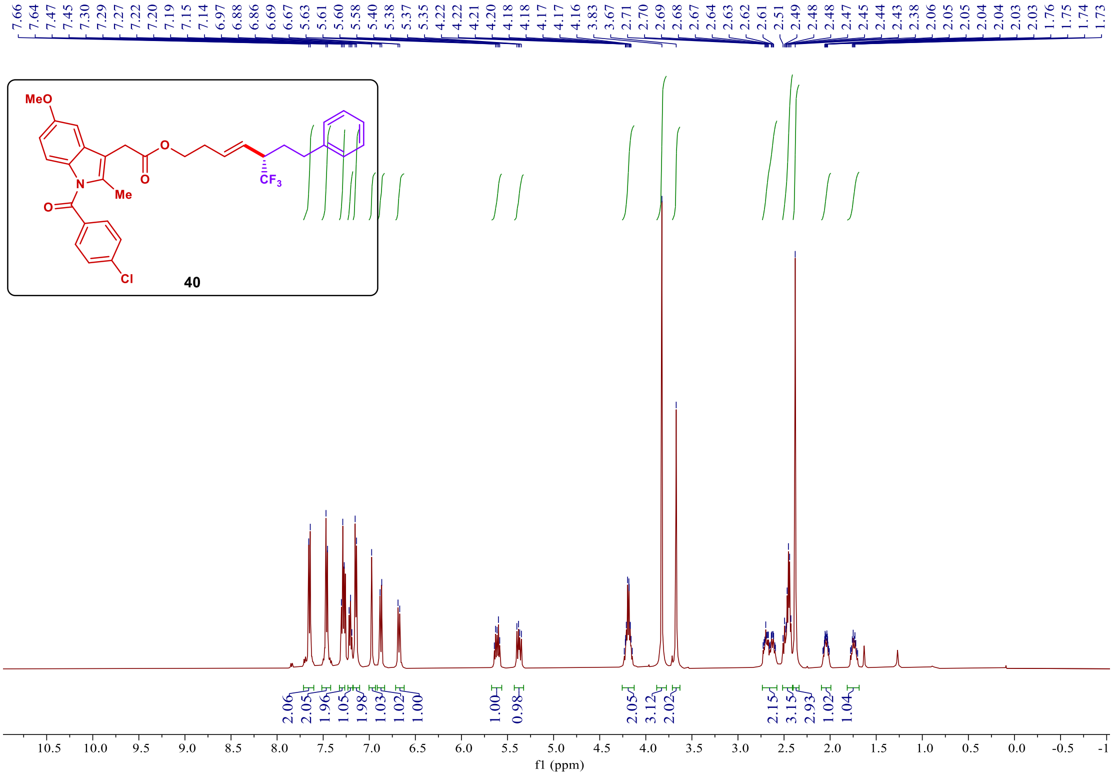

Supplementary Figure 137. ^{1}H NMR Spectrum of Compound **40** (500 MHz, $CDCl_3$)

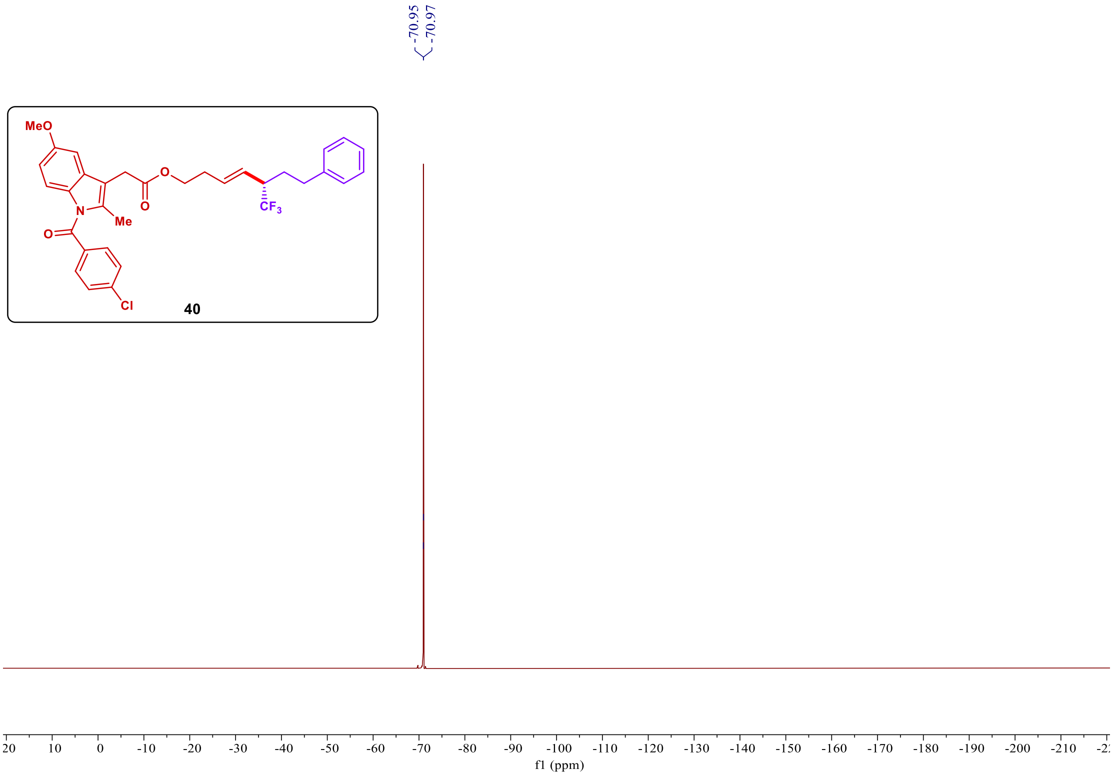

Supplementary Figure 138. ^{19}F NMR Spectrum of Compound **40** (471 MHz, $CDCl_3$)

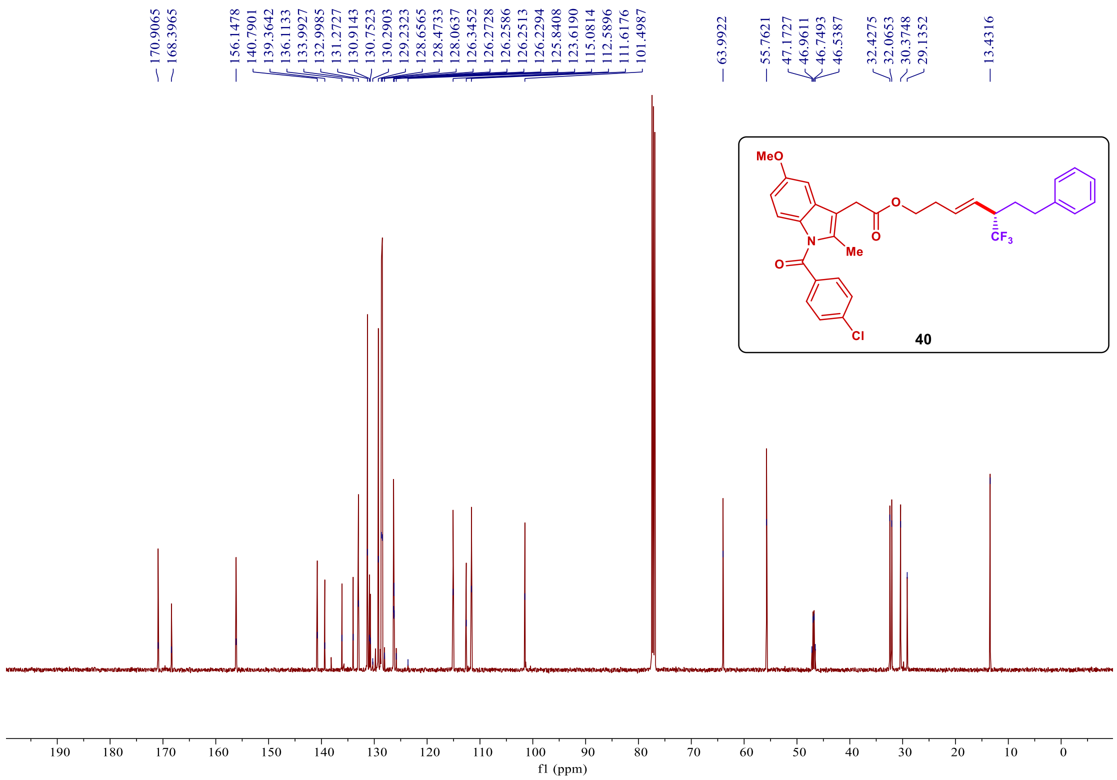

Supplementary Figure 139. ^{13}C NMR Spectrum of Compound **40** (126 MHz, $CDCl_3$)

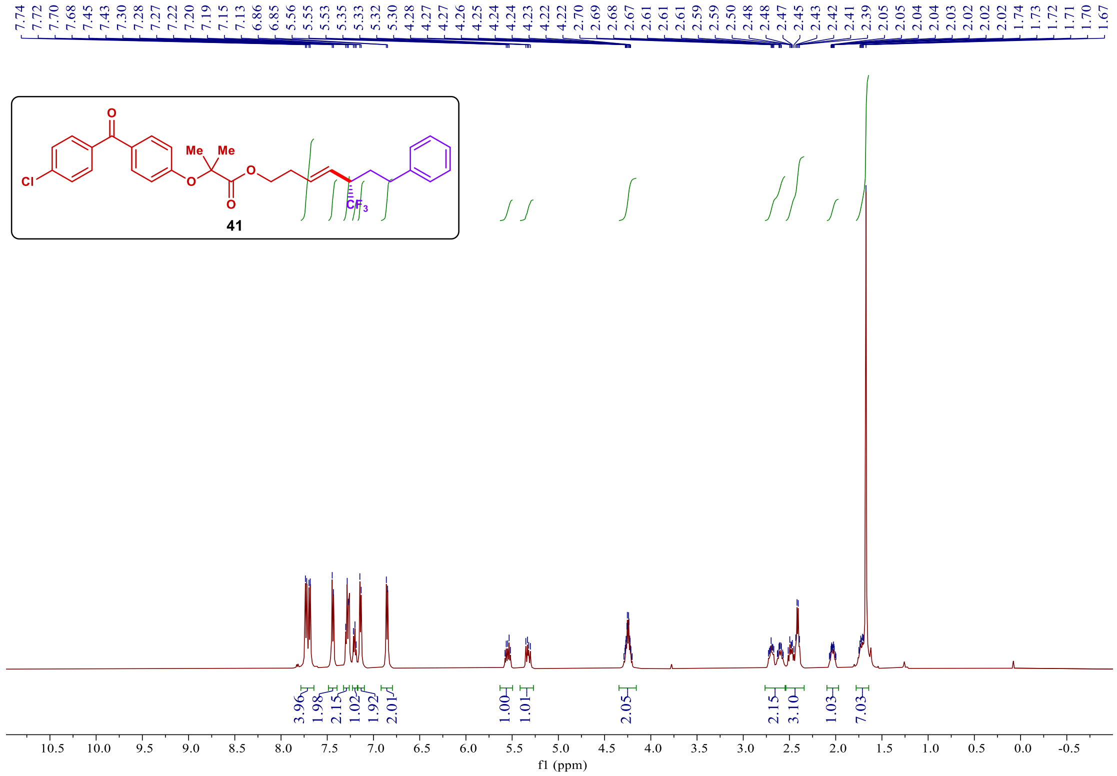

Supplementary Figure 140. ^{1}H NMR Spectrum of Compound **41** (500 MHz, $CDCl_3$)

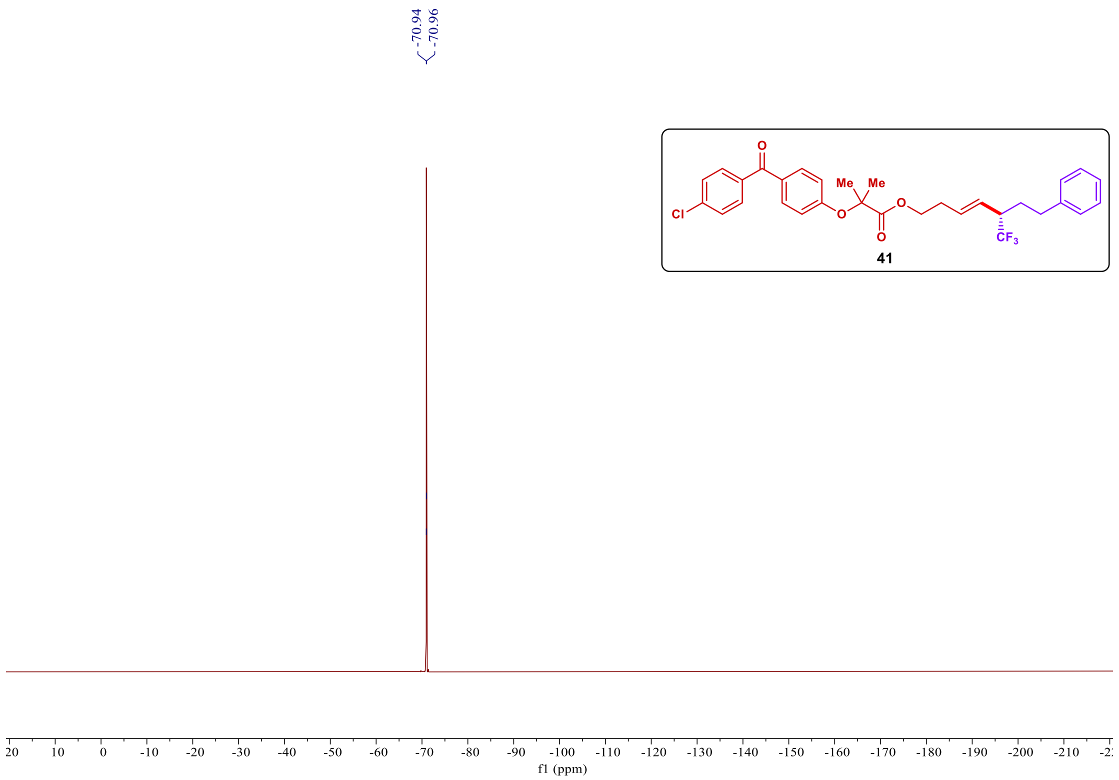

Supplementary Figure 141. ^{19}F NMR Spectrum of Compound **41** (471 MHz, $CDCl_3$)

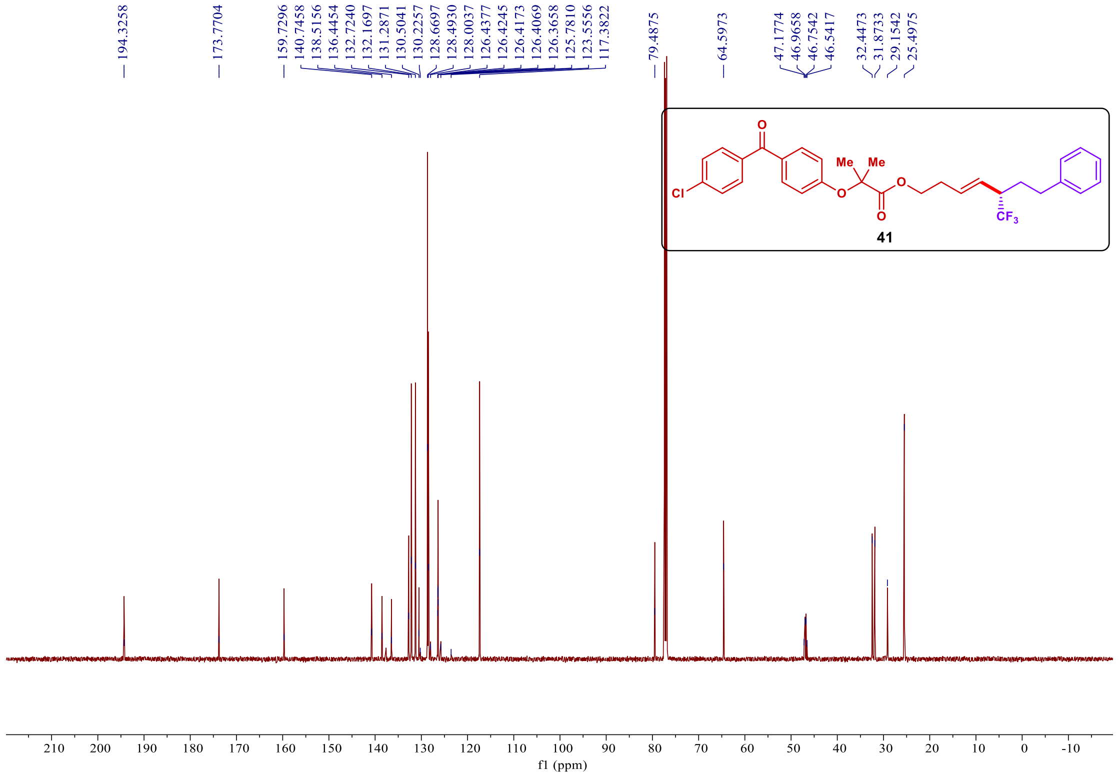

Supplementary Figure 142. ^{13}C NMR Spectrum of Compound **41** (126 MHz, $CDCl_3$)

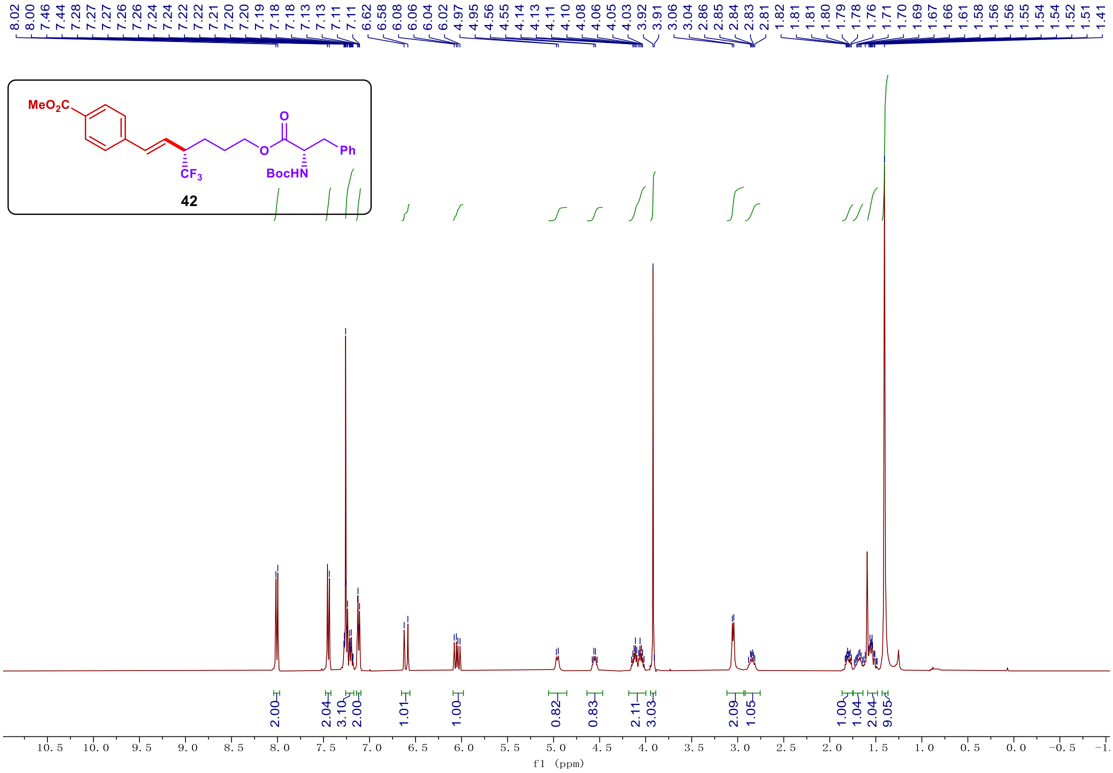

Supplementary Figure 143. ^{1}H NMR Spectrum of Compound **42** (400 MHz, $CDCl_3$)

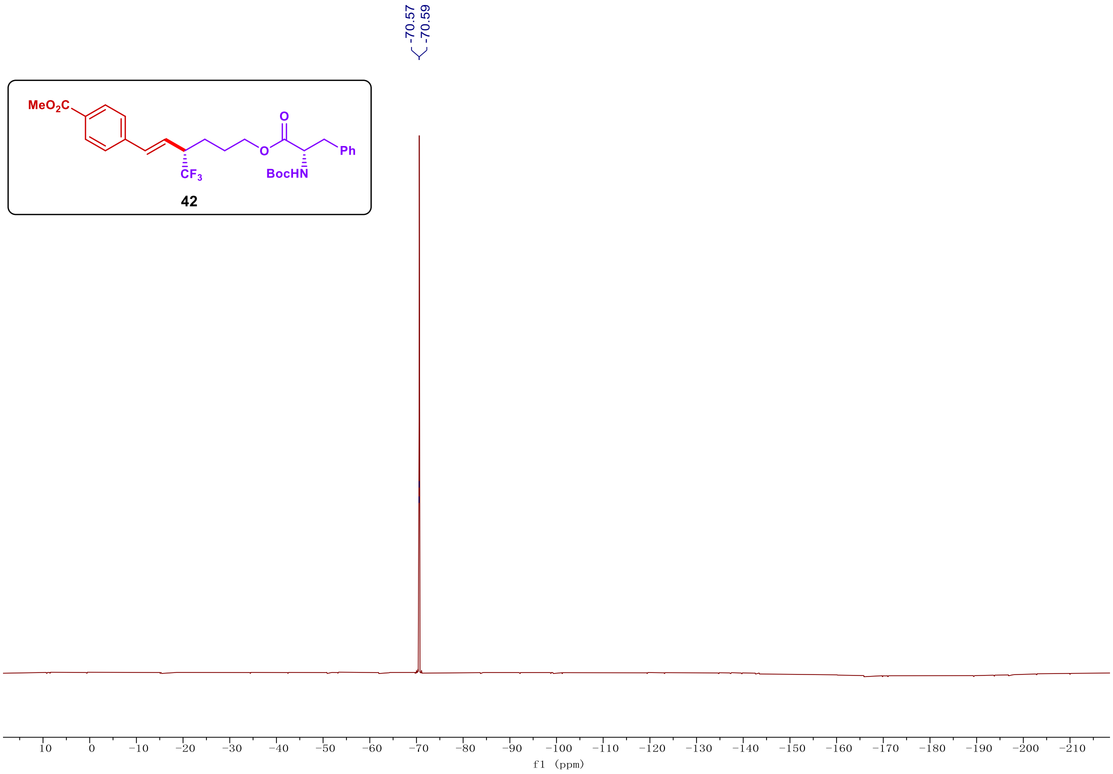

Supplementary Figure 144. ^{19}F NMR Spectrum of Compound **42** (376 MHz, $CDCl_3$)

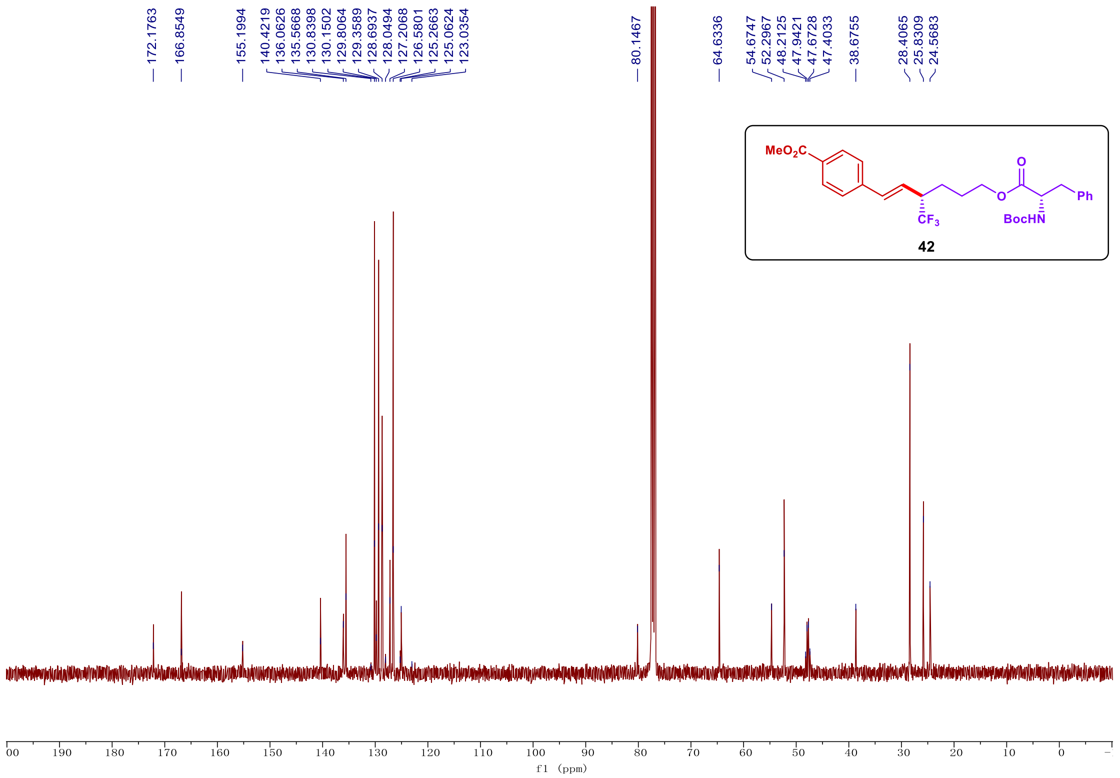

Supplementary Figure 145. ^{13}C NMR Spectrum of Compound **42** (101 MHz, $CDCl_3$)

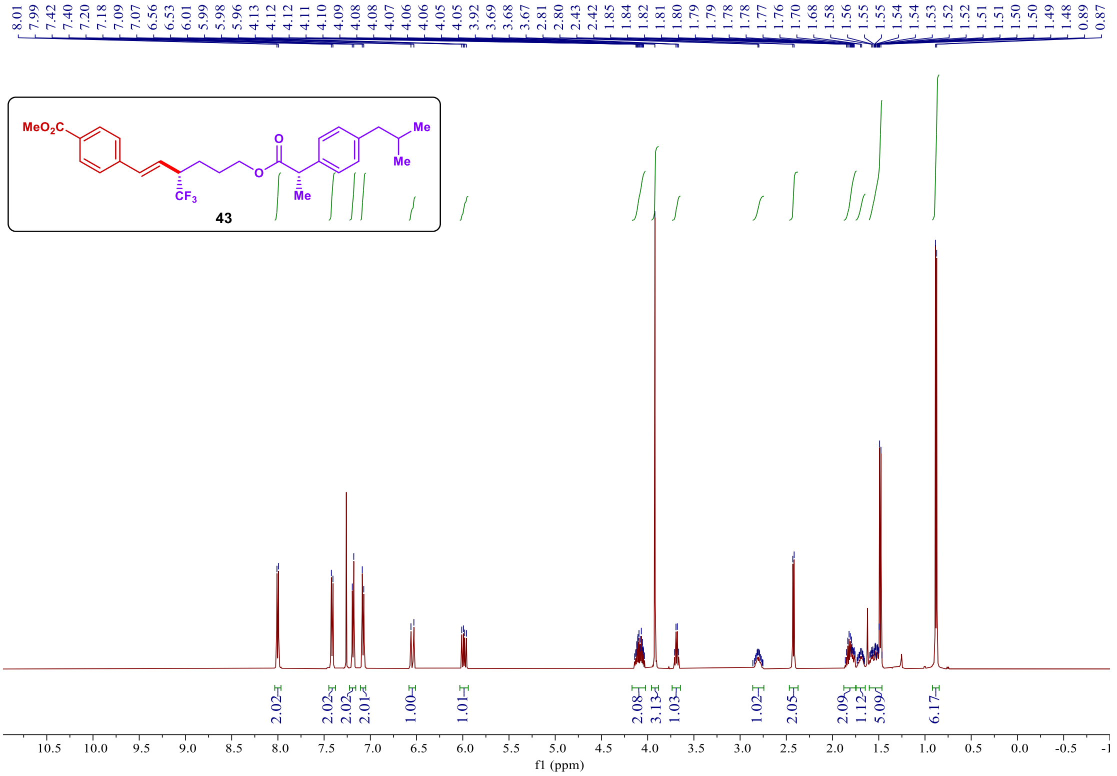

Supplementary Figure 146. ^{1}H NMR Spectrum of Compound **43** (500 MHz, $CDCl_3$)

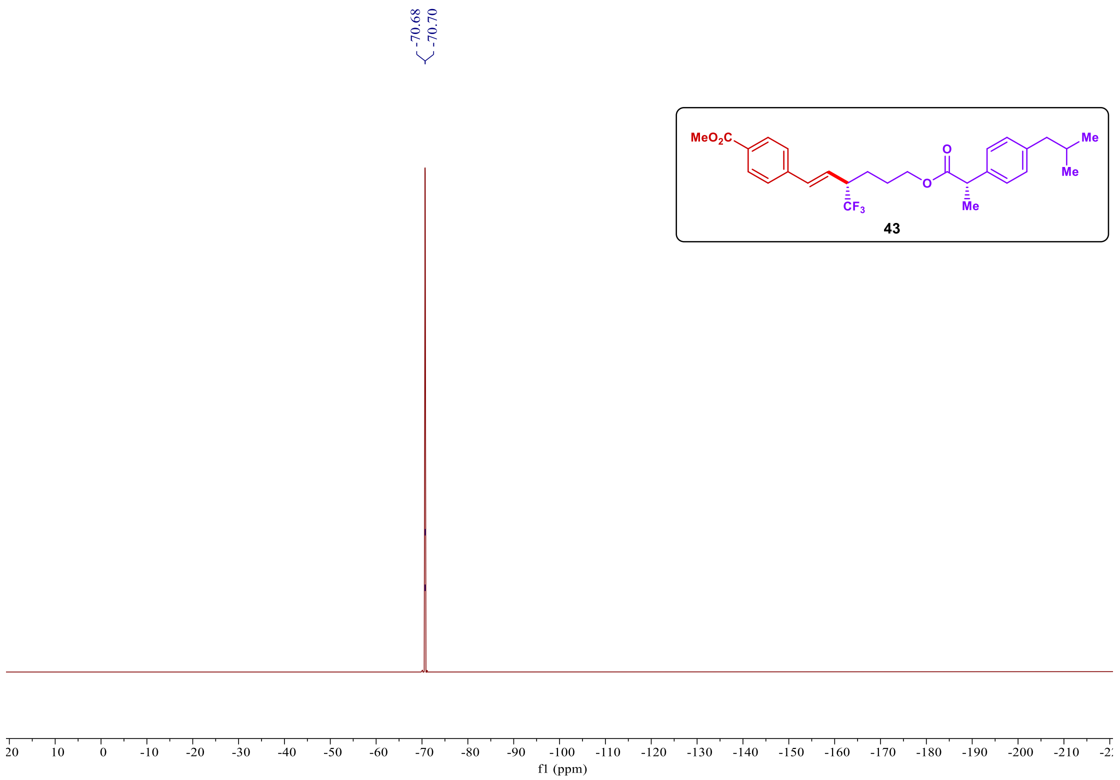

Supplementary Figure 147. ^{19}F NMR Spectrum of Compound **43** (471 MHz, $CDCl_3$)

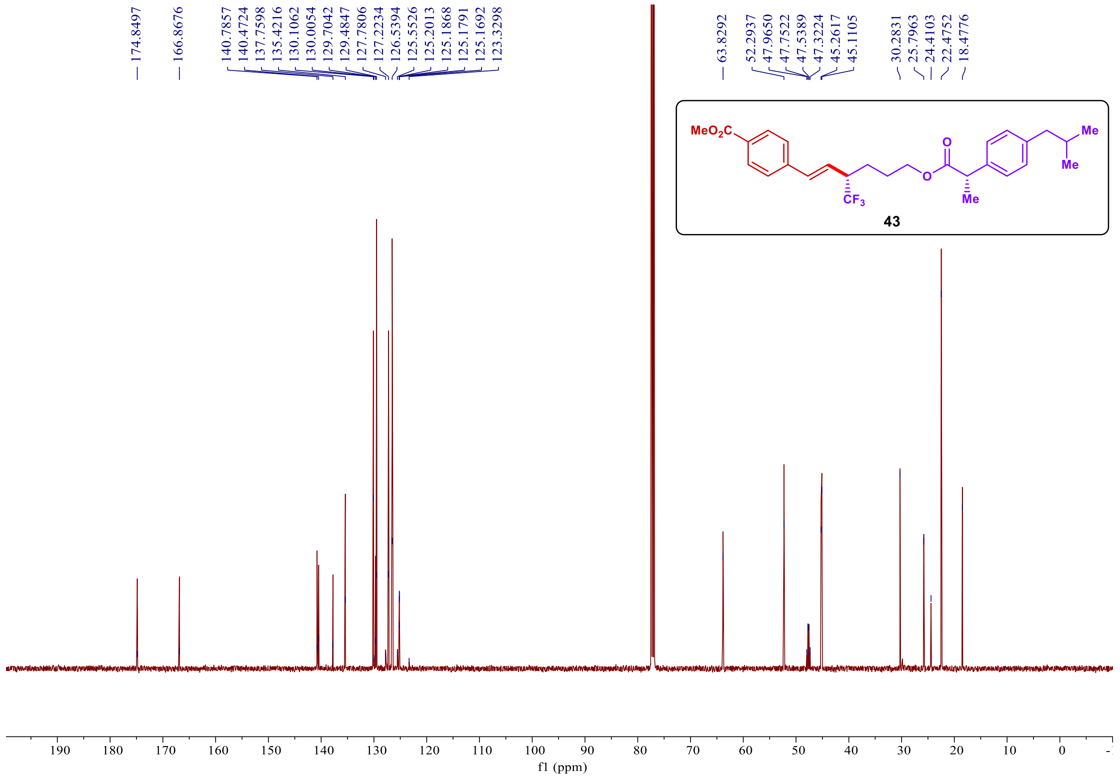

Supplementary Figure 148. ^{13}C NMR Spectrum of Compound **43** (126 MHz, $CDCl_3$)

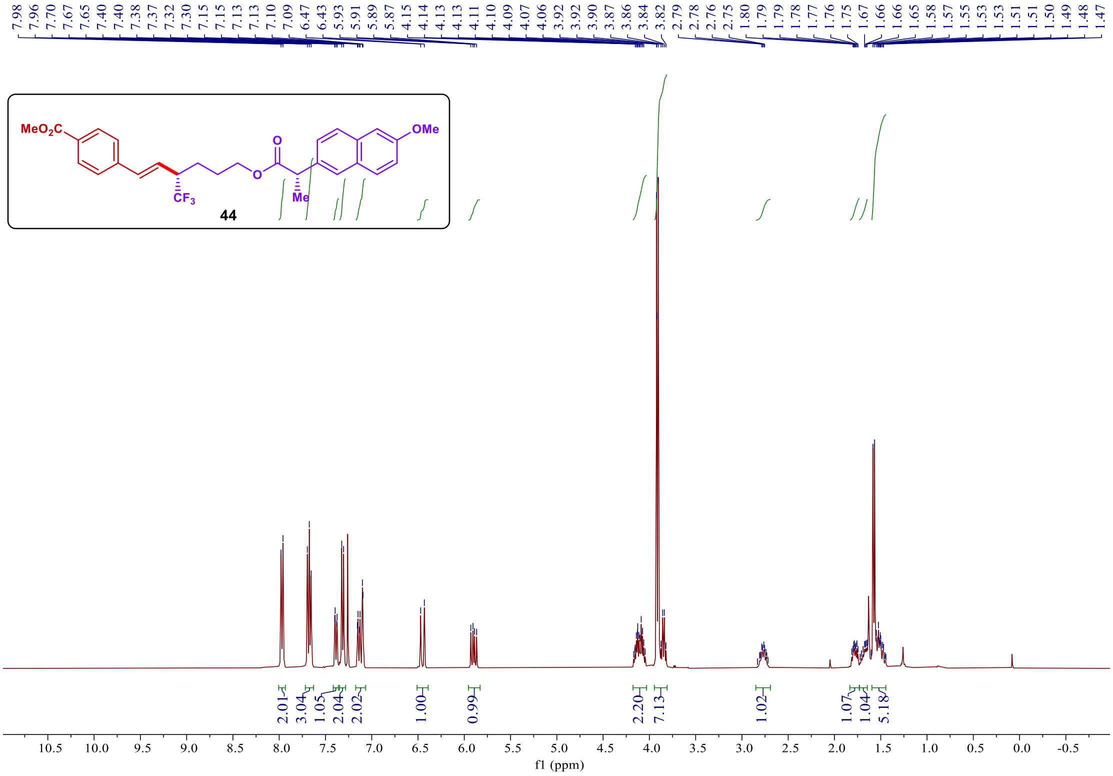

Supplementary Figure 149. ^{1}H NMR Spectrum of Compound **44** (400 MHz, $CDCl_3$)

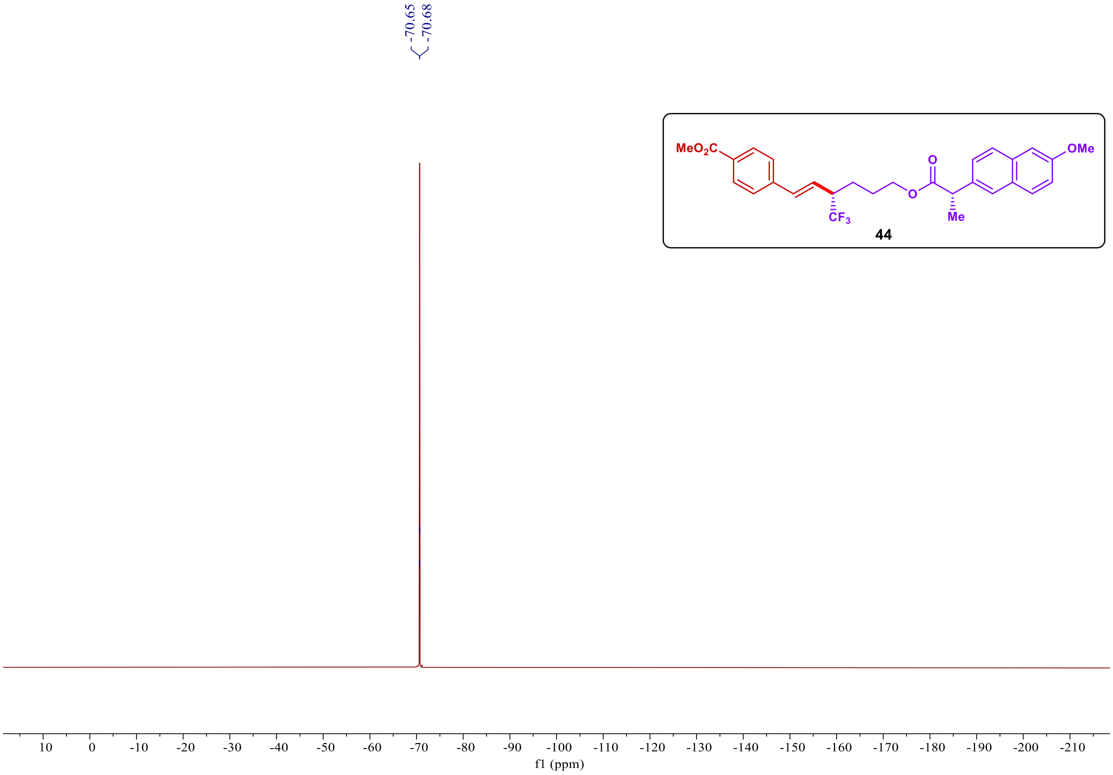

Supplementary Figure 150. ^{19}F NMR Spectrum of Compound **44** (376 MHz, $CDCl_3$)

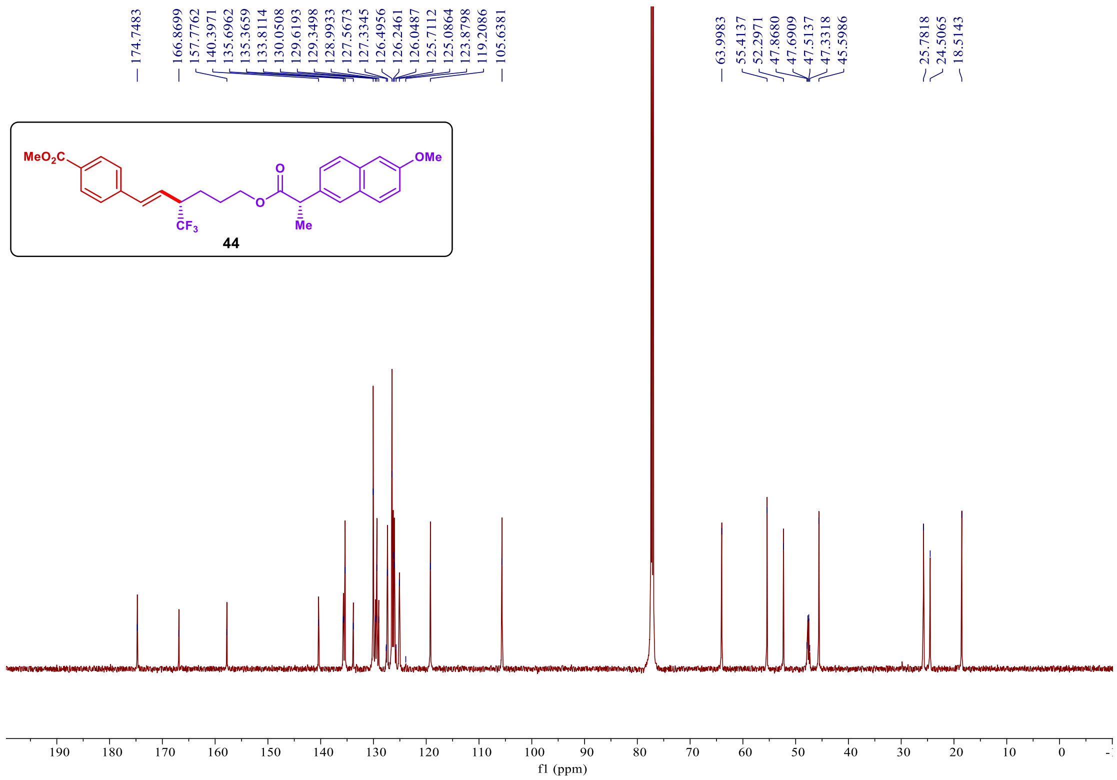

Supplementary Figure 151. ^{13}C NMR Spectrum of Compound **44** (151 MHz, $CDCl_3$)

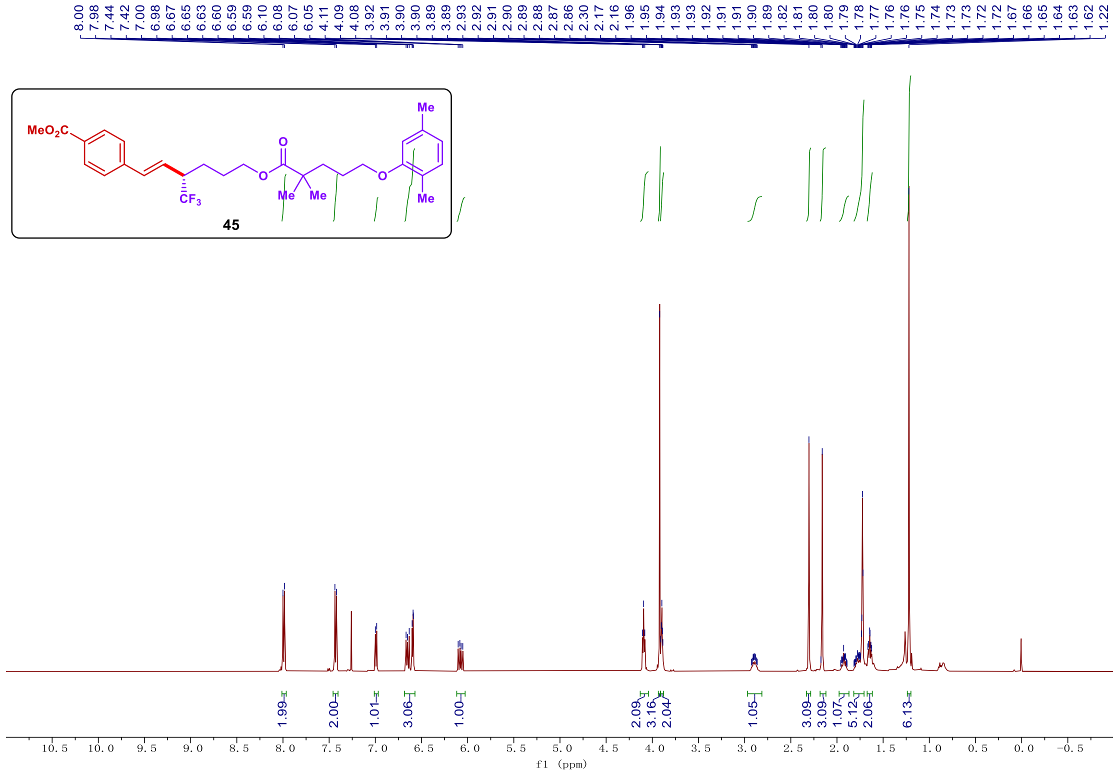

Supplementary Figure 152. ^{1}H NMR Spectrum of Compound **45** (500 MHz, $CDCl_3$)

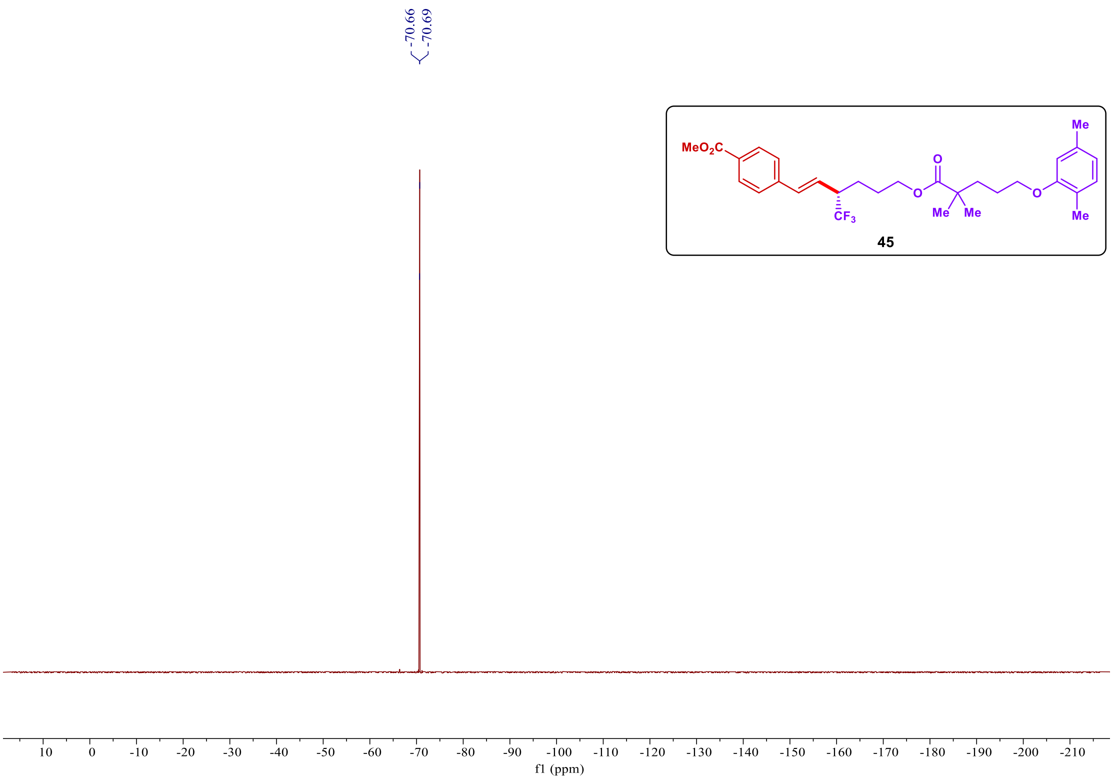

Supplementary Figure 153. ^{19}F NMR Spectrum of Compound **45** (376 MHz, $CDCl_3$)

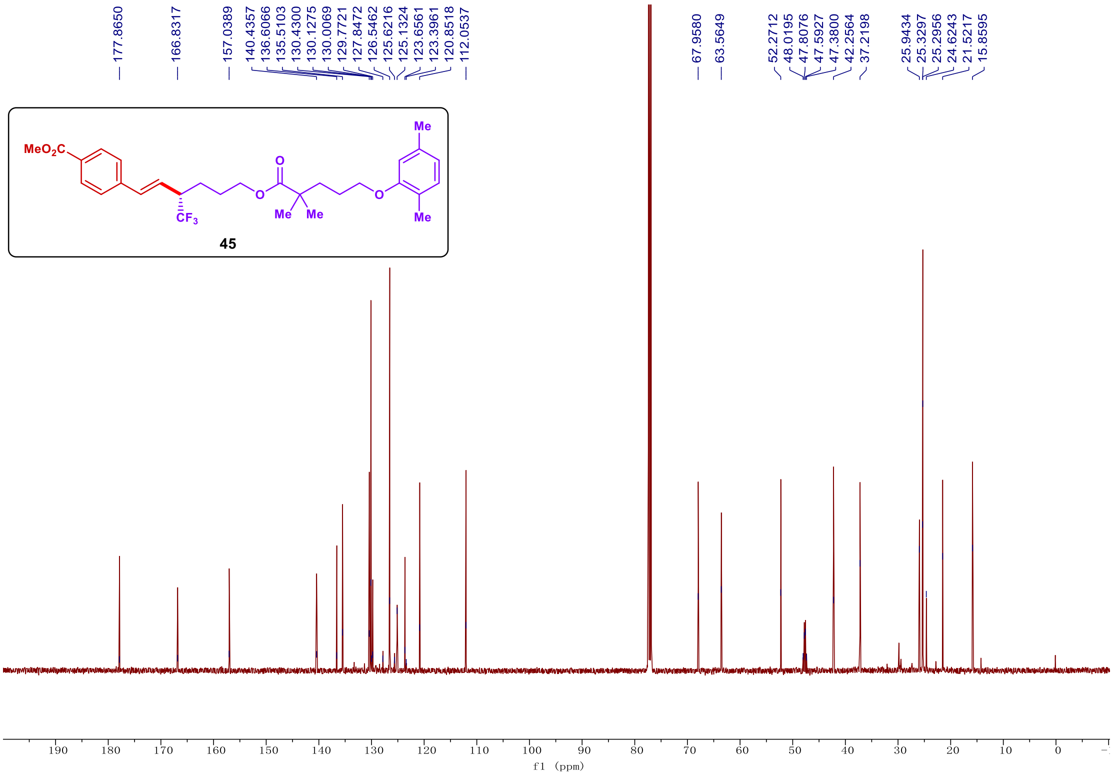

Supplementary Figure 154. ^{13}C NMR Spectrum of Compound **45** (126 MHz, $CDCl_3$)

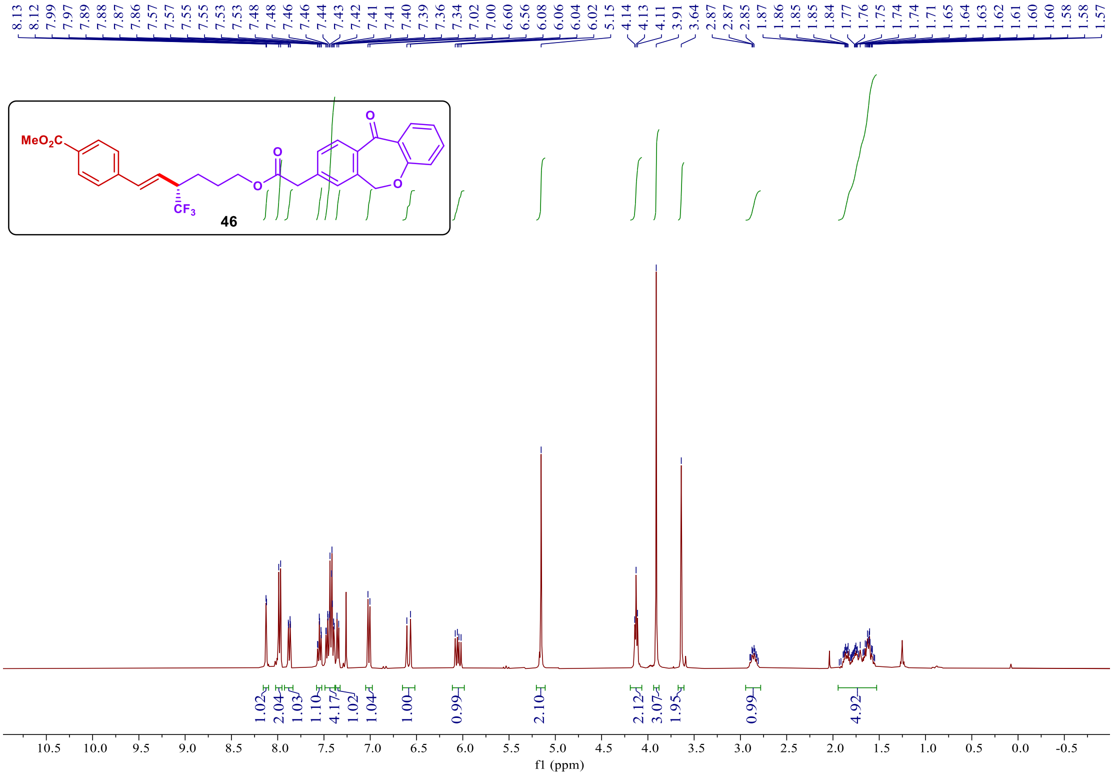

Supplementary Figure 155. ^{1}H NMR Spectrum of Compound **46** (400 MHz, $CDCl_3$)

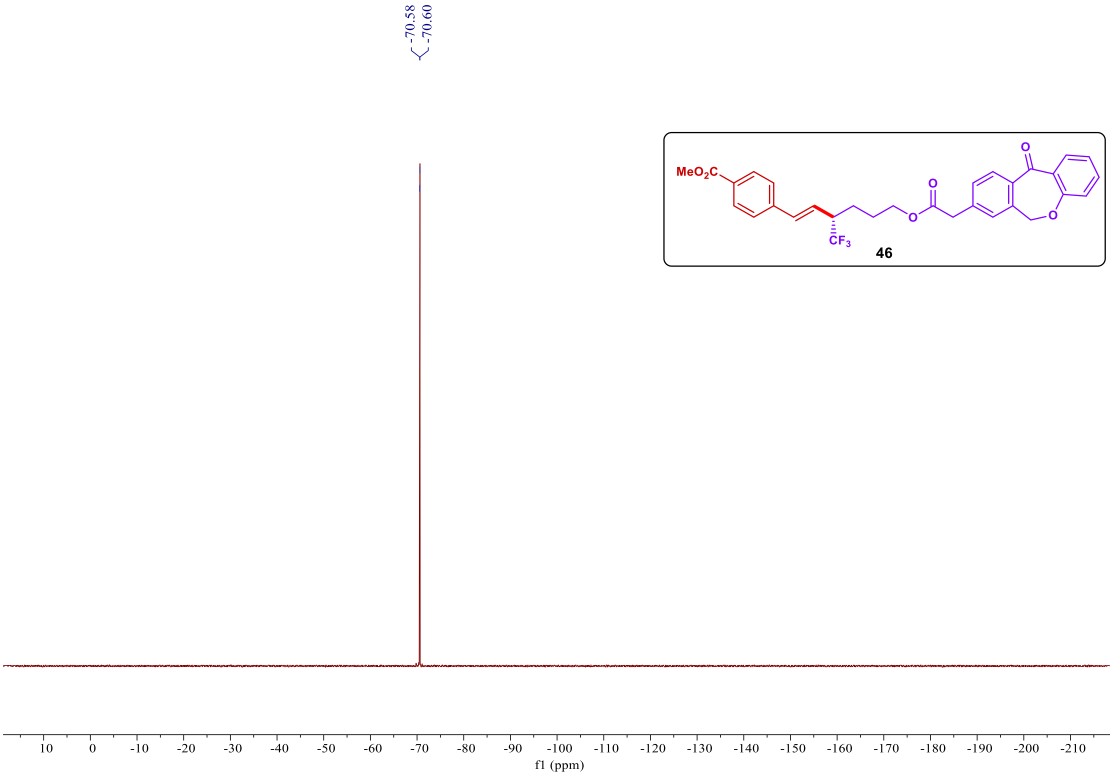

Supplementary Figure 156. ^{19}F NMR Spectrum of Compound **46** (376 MHz, $CDCl_3$)

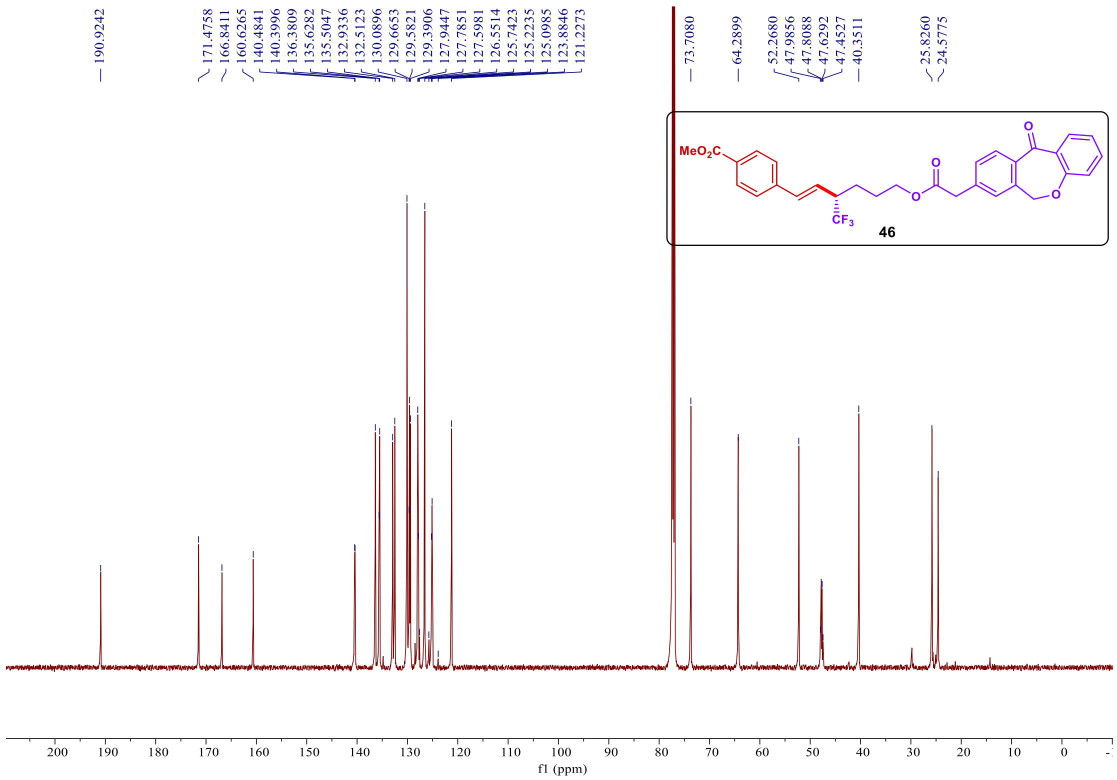

Supplementary Figure 157. ^{13}C NMR Spectrum of Compound **46** (151 MHz, $CDCl_3$)

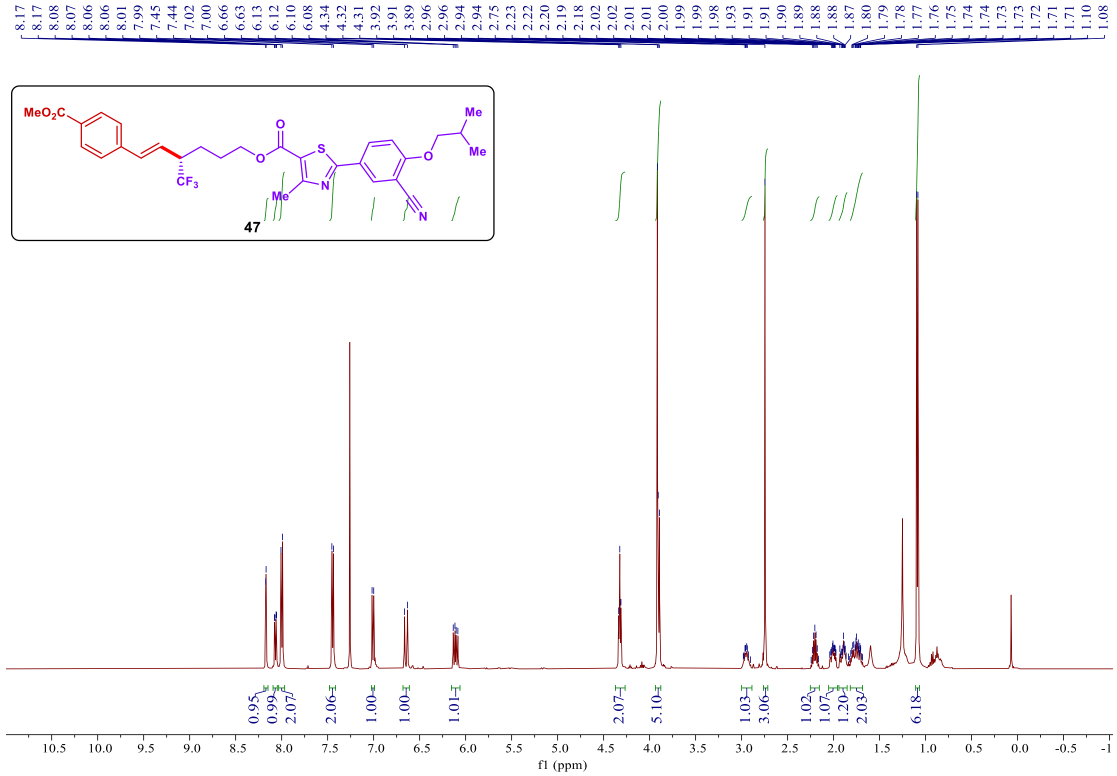

Supplementary Figure 158. ^{1}H NMR Spectrum of Compound **47** (500 MHz, $CDCl_3$)

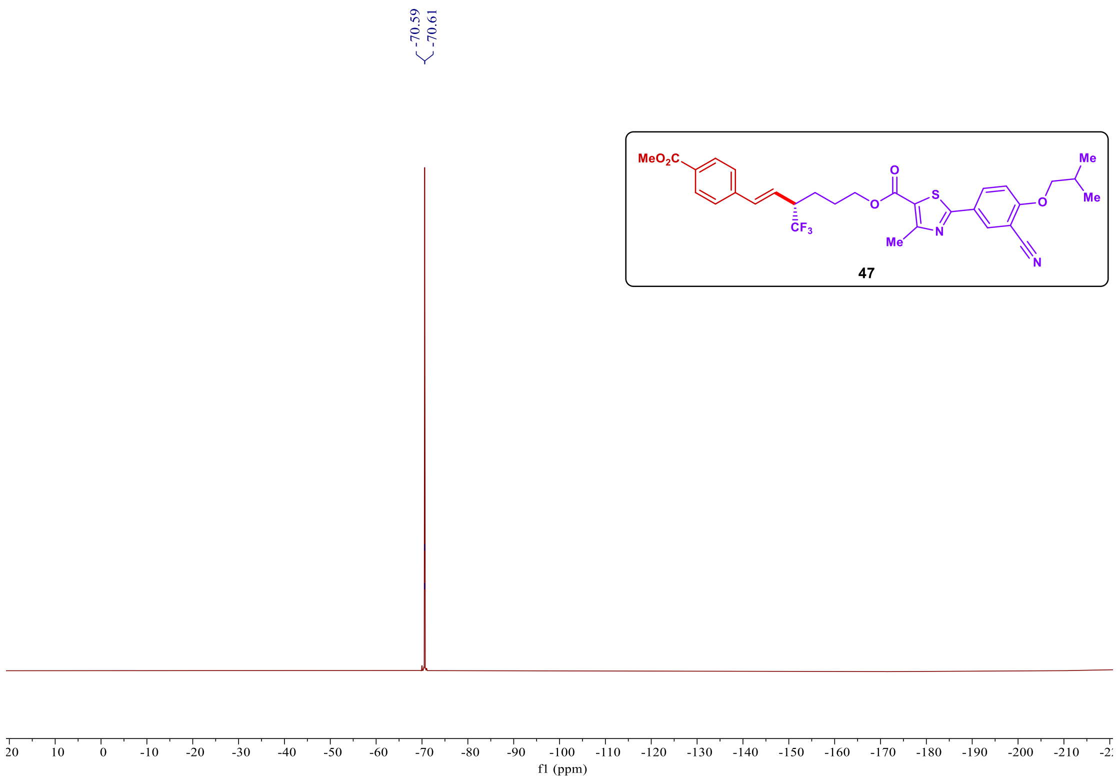

Supplementary Figure 159. ^{19}F NMR Spectrum of Compound **47** (471 MHz, $CDCl_3$)

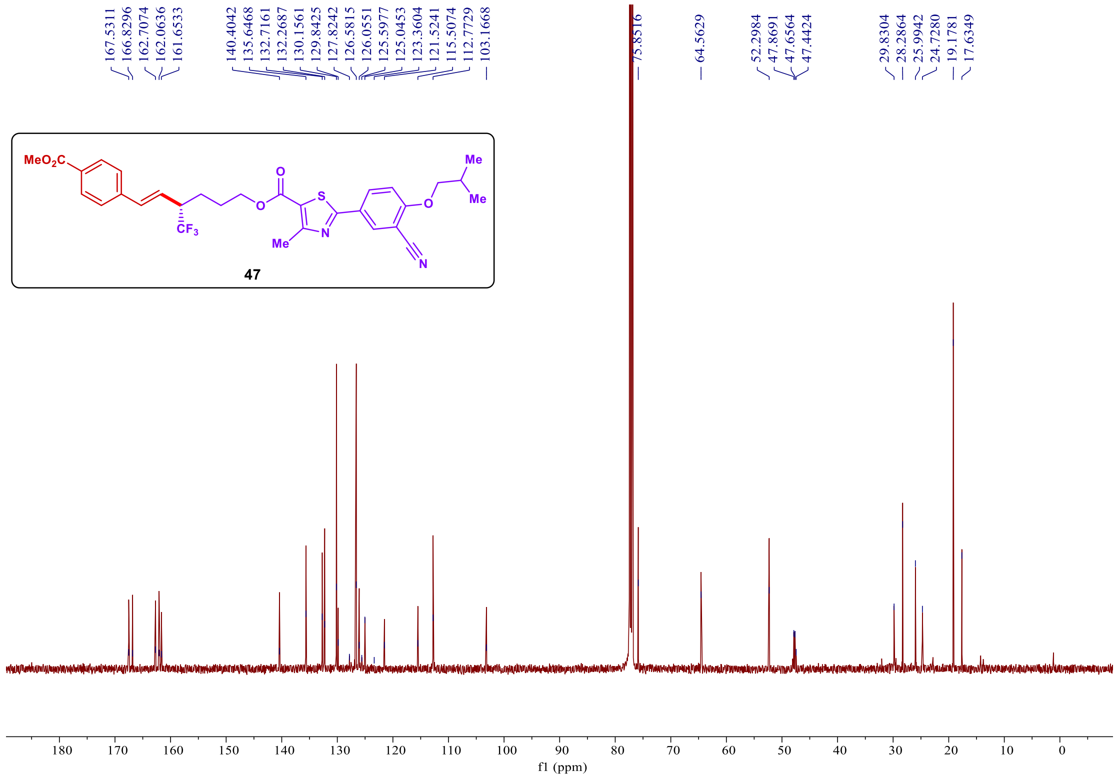

Supplementary Figure 160. ^{13}C NMR Spectrum of Compound **47** (126 MHz, $CDCl_3$)

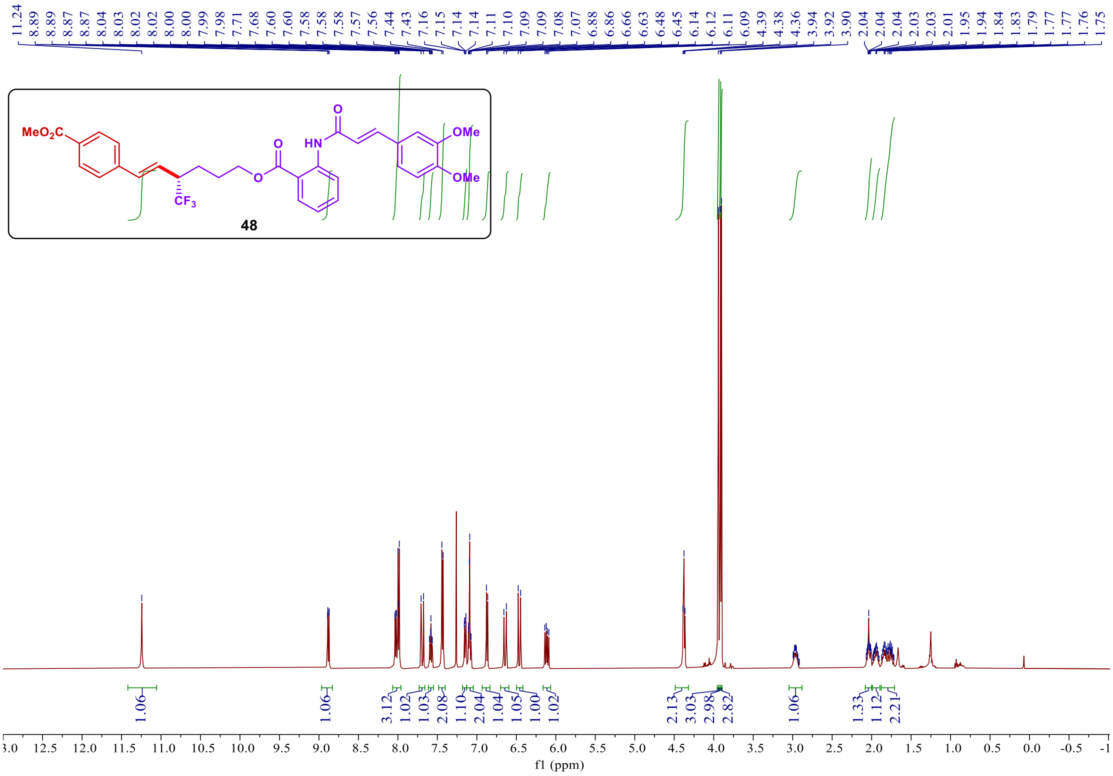

Supplementary Figure 161. ^{1}H NMR Spectrum of Compound **48** (500 MHz, $CDCl_3$)

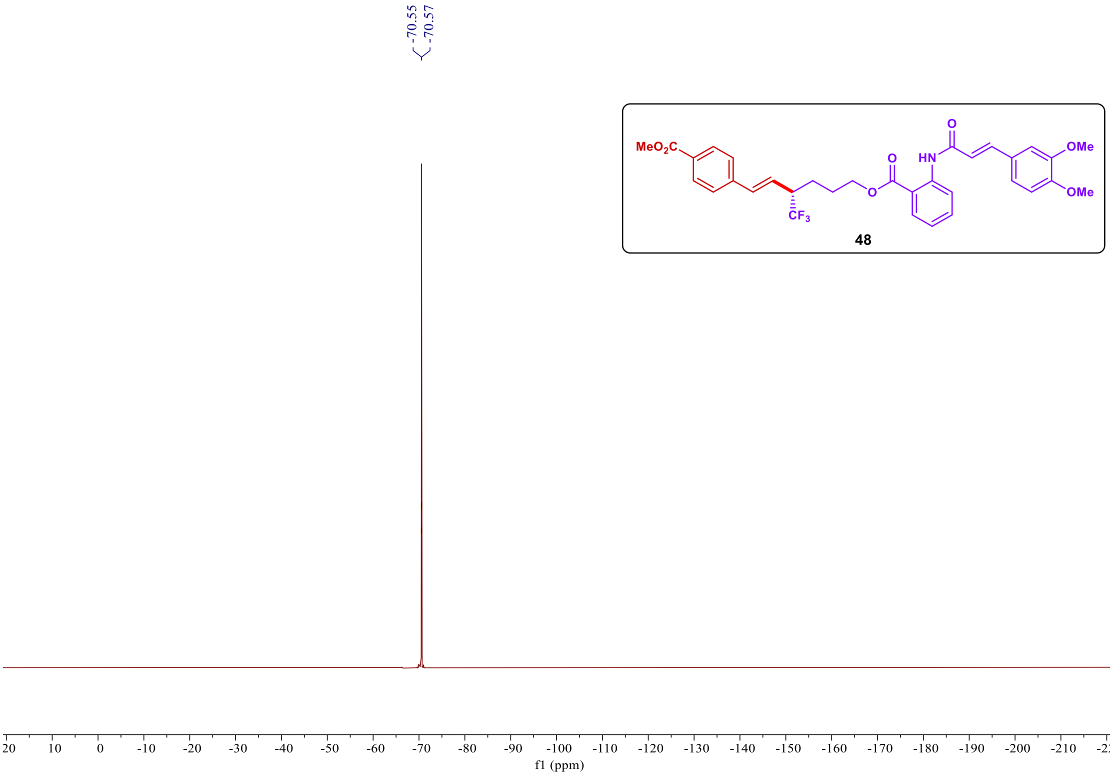

Supplementary Figure 162. ^{19}F NMR Spectrum of Compound **48** (471 MHz, $CDCl_3$)

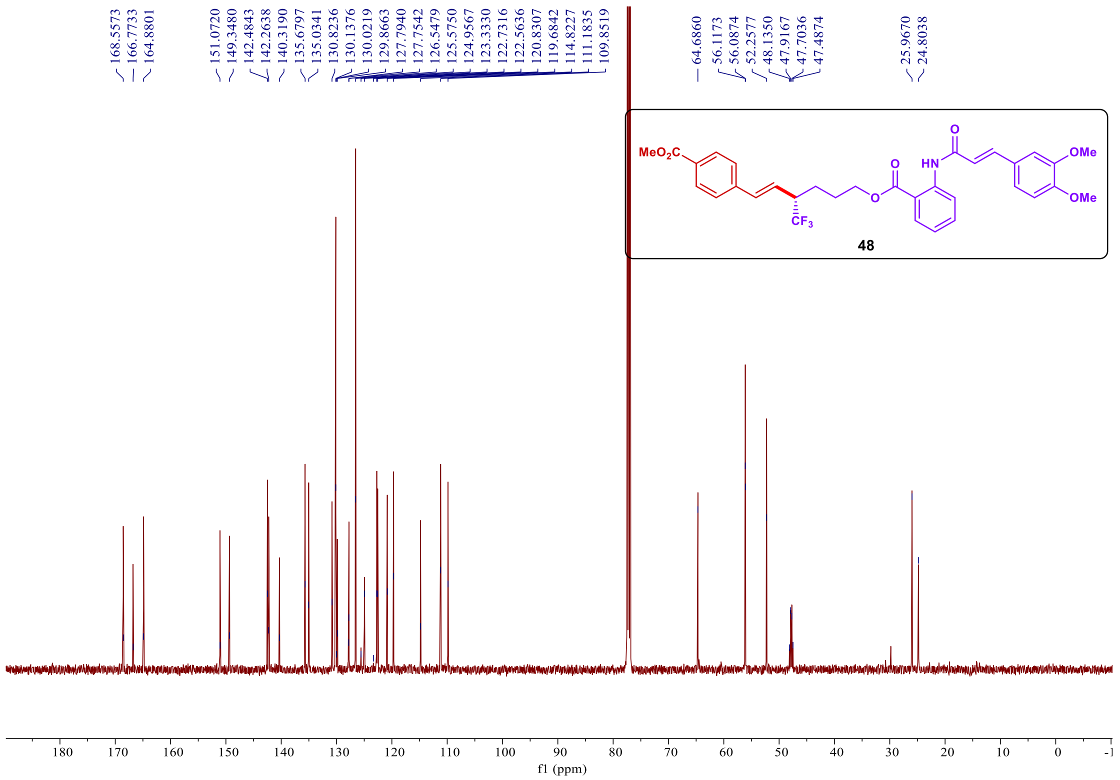

Supplementary Figure 163. ^{13}C NMR Spectrum of Compound **48** (126 MHz, $CDCl_3$)

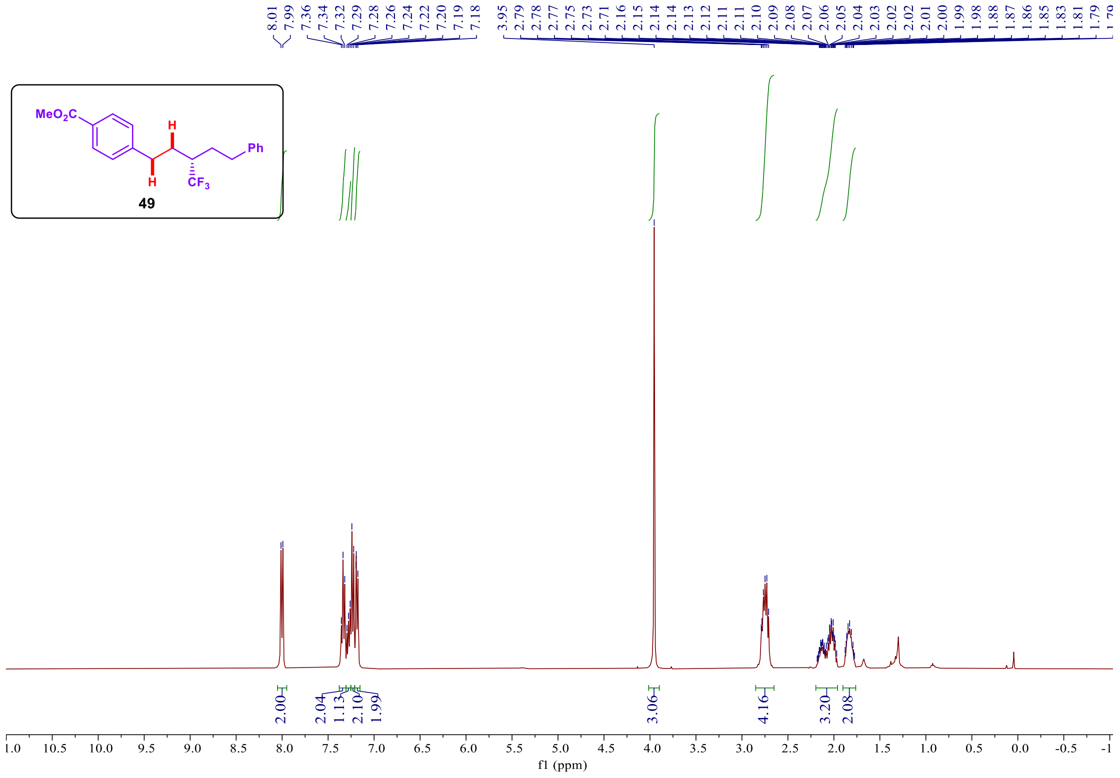

Supplementary Figure 164. ^{1}H NMR Spectrum of Compound **49** (400 MHz, $CDCl_3$)

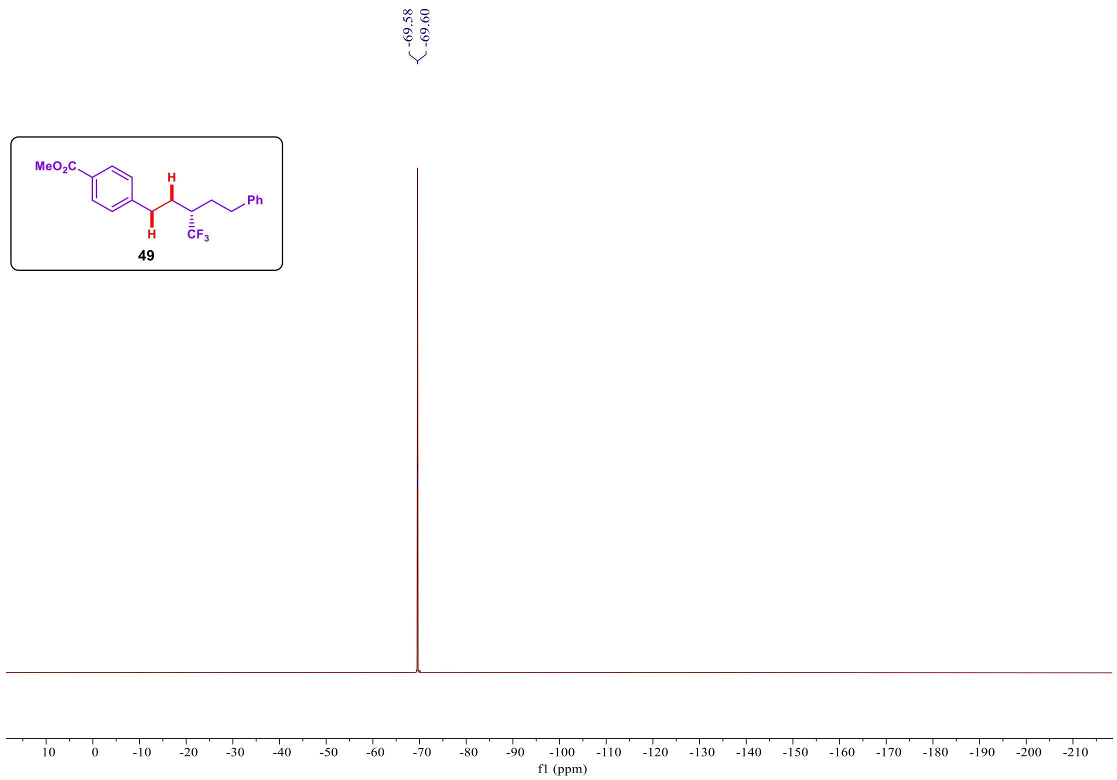

Supplementary Figure 165. ^{19}F NMR Spectrum of Compound **49** (376 MHz, $CDCl_3$)

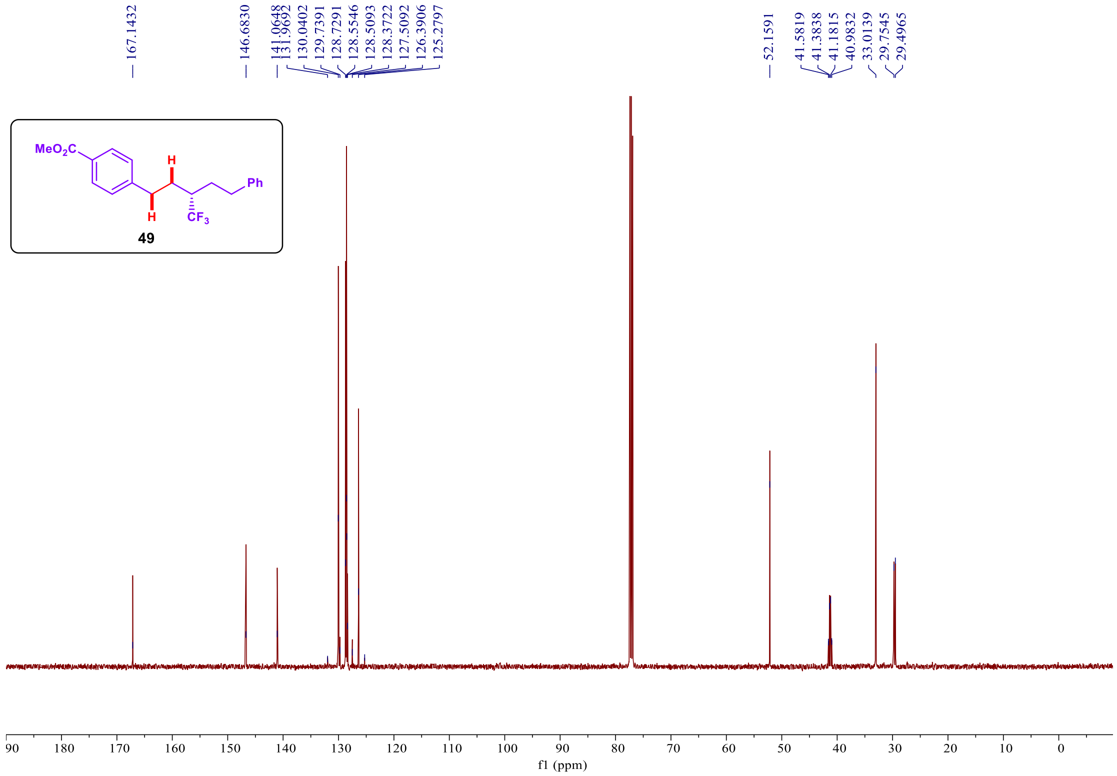

Supplementary Figure 166. ^{13}C NMR Spectrum of Compound **49** (126 MHz, $CDCl_3$)

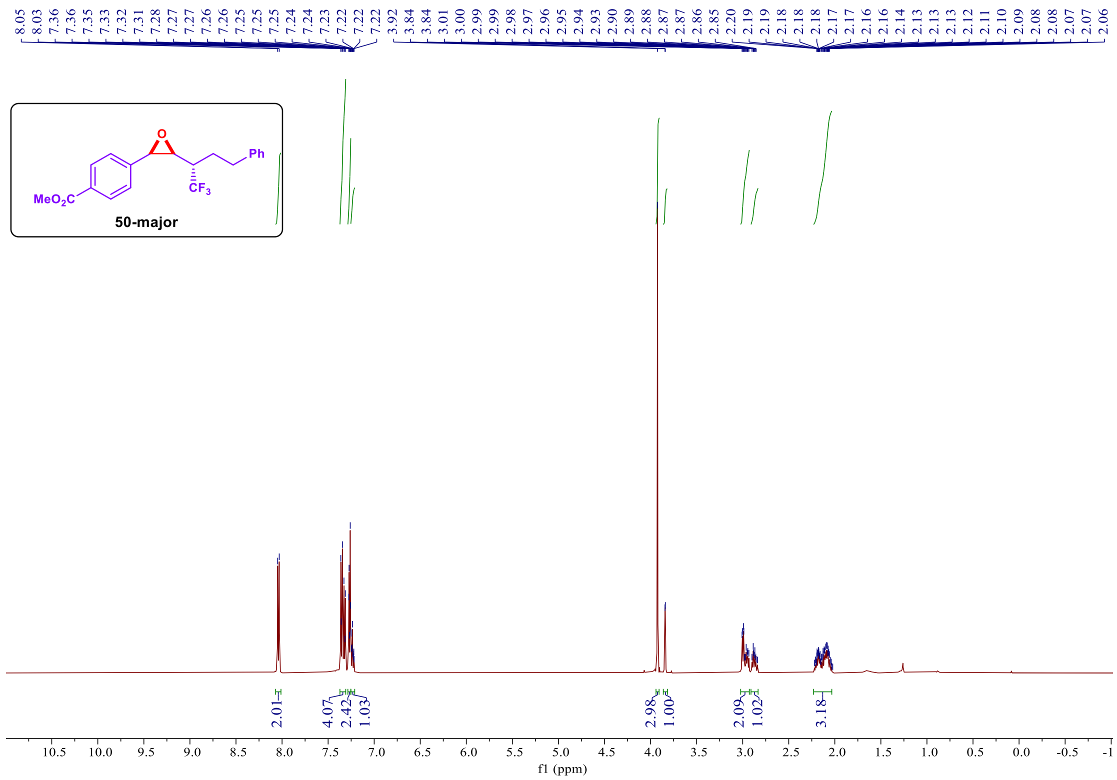

Supplementary Figure 167. ^{1}H NMR Spectrum of Compound **50-major** (500 MHz, $CDCl_3$)

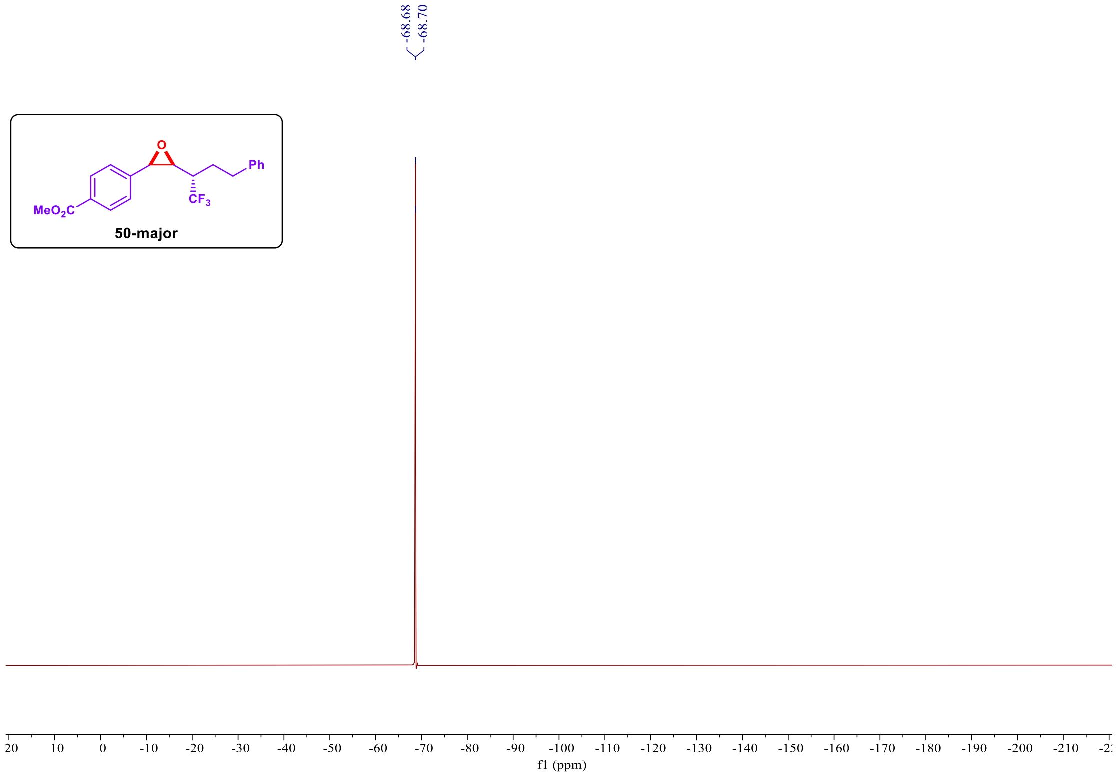

Supplementary Figure 168. ^{19}F NMR Spectrum of Compound **50-major** (471 MHz, $CDCl_3$)

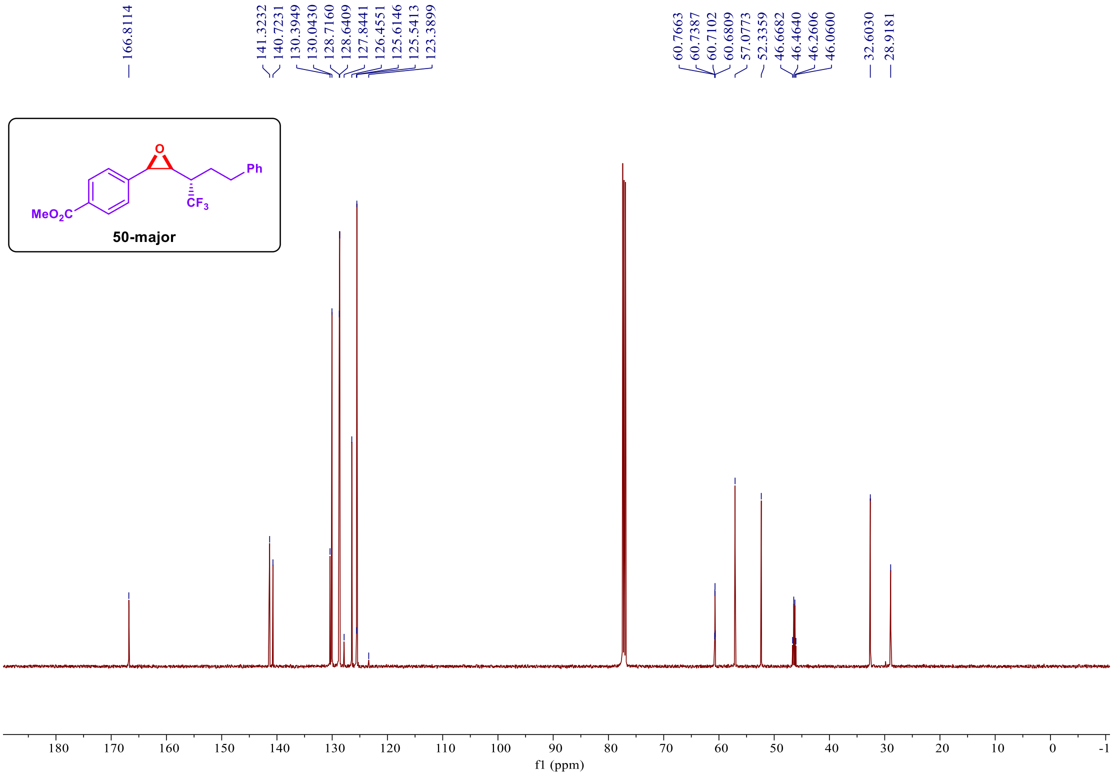

Supplementary Figure 169. ^{13}C NMR Spectrum of Compound **50-major** (126 MHz, $CDCl_3$)

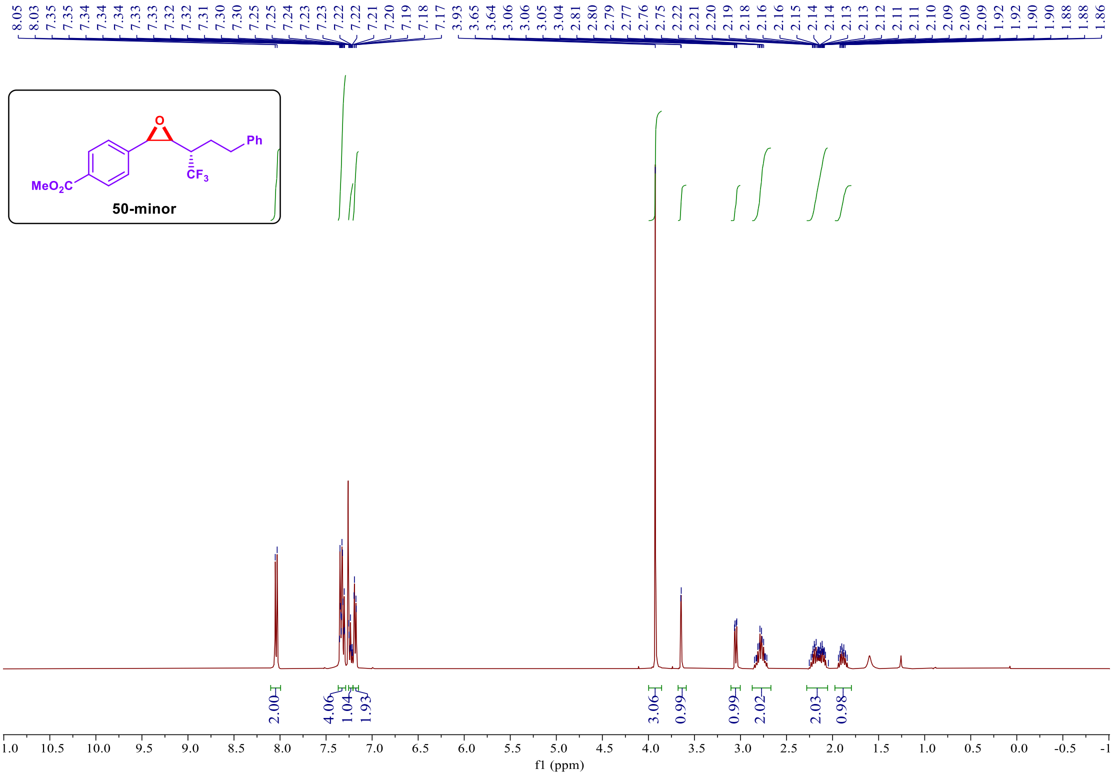

Supplementary Figure 170. ^{1}H NMR Spectrum of Compound **50-minor** (400 MHz, $CDCl_3$)

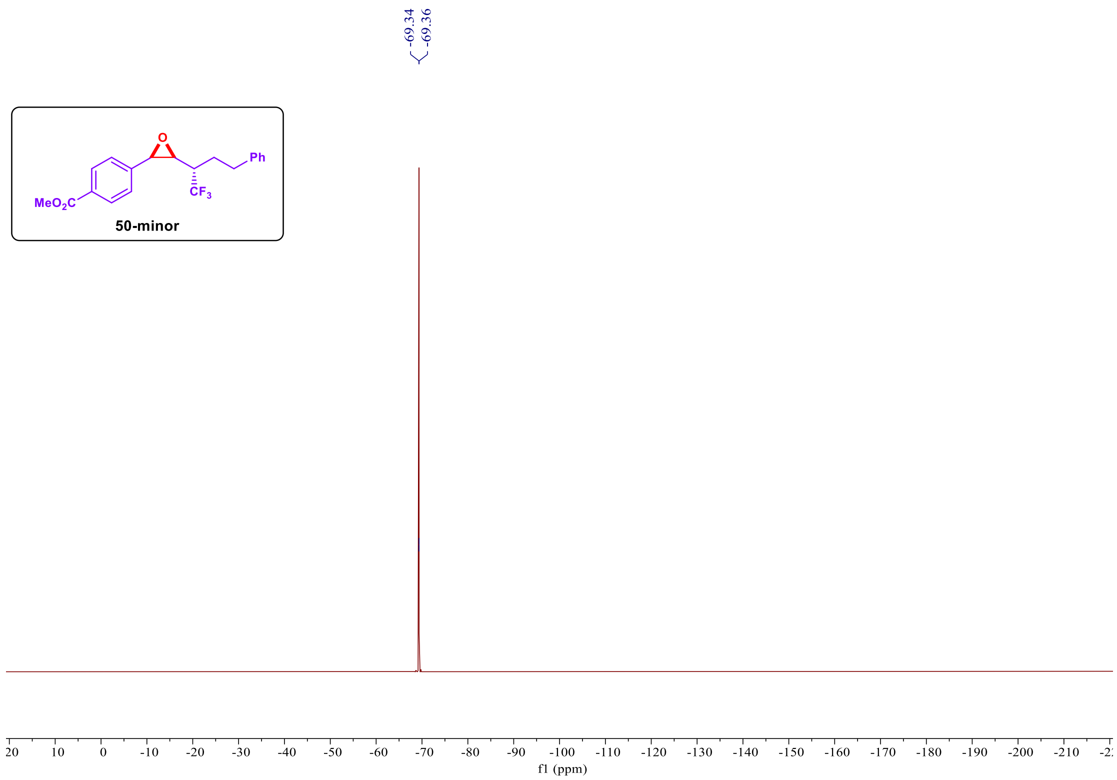

Supplementary Figure 171. ^{19}F NMR Spectrum of Compound **50-minor** (471 MHz, $CDCl_3$)

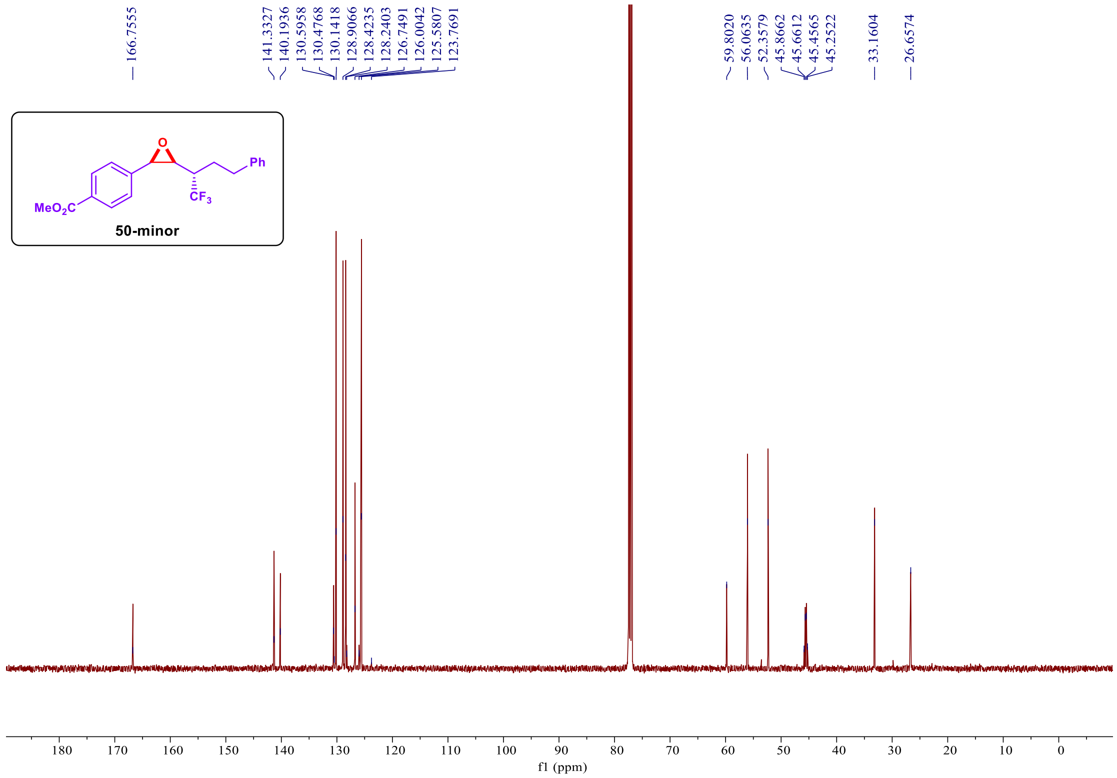

Supplementary Figure 172. ^{13}C NMR Spectrum of Compound **50-minor** (126 MHz, $CDCl_3$)

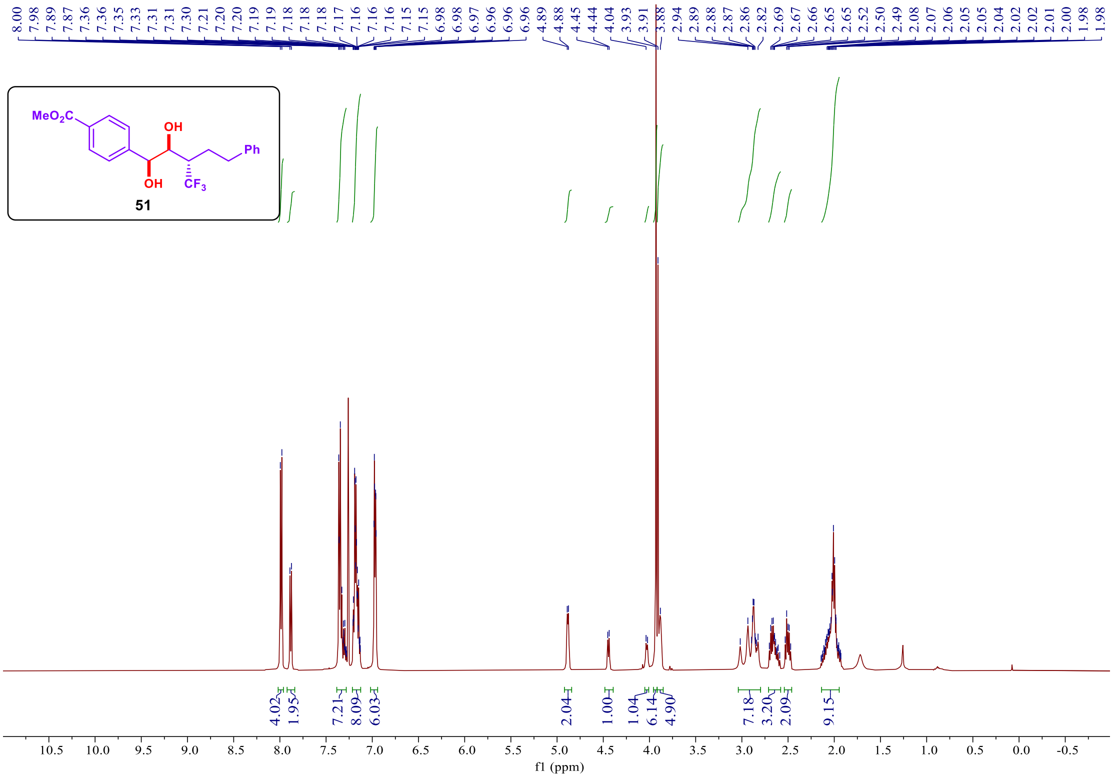

Supplementary Figure 173. ^{1}H NMR Spectrum of Compound **51** (500 MHz, $CDCl_3$)

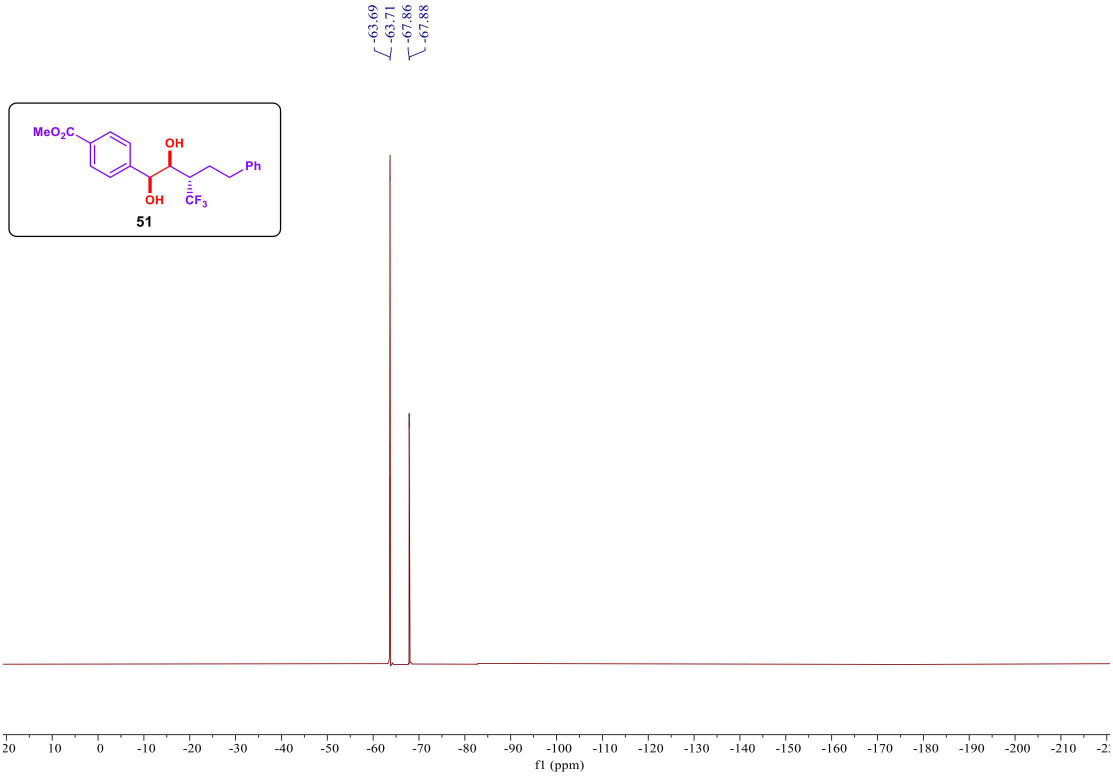

Supplementary Figure 174. ^{19}F NMR Spectrum of Compound **51** (471 MHz, $CDCl_3$)

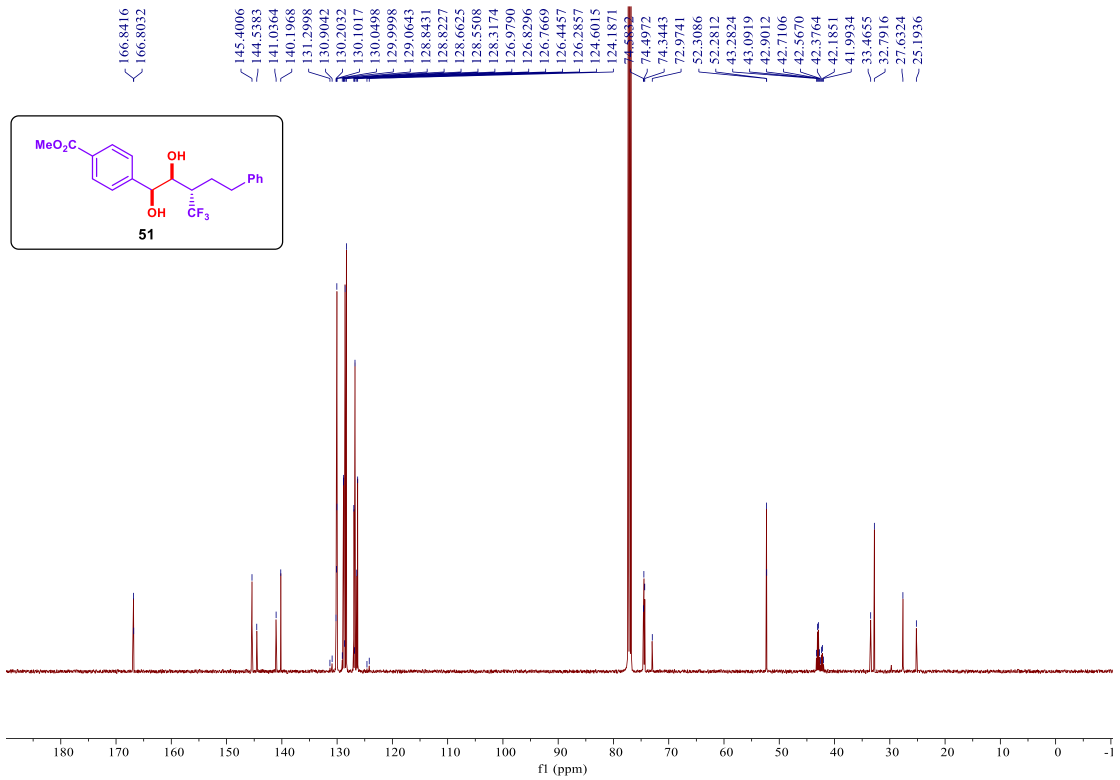

Supplementary Figure 175. ^{13}C NMR Spectrum of Compound **51** (126 MHz, $CDCl_3$)

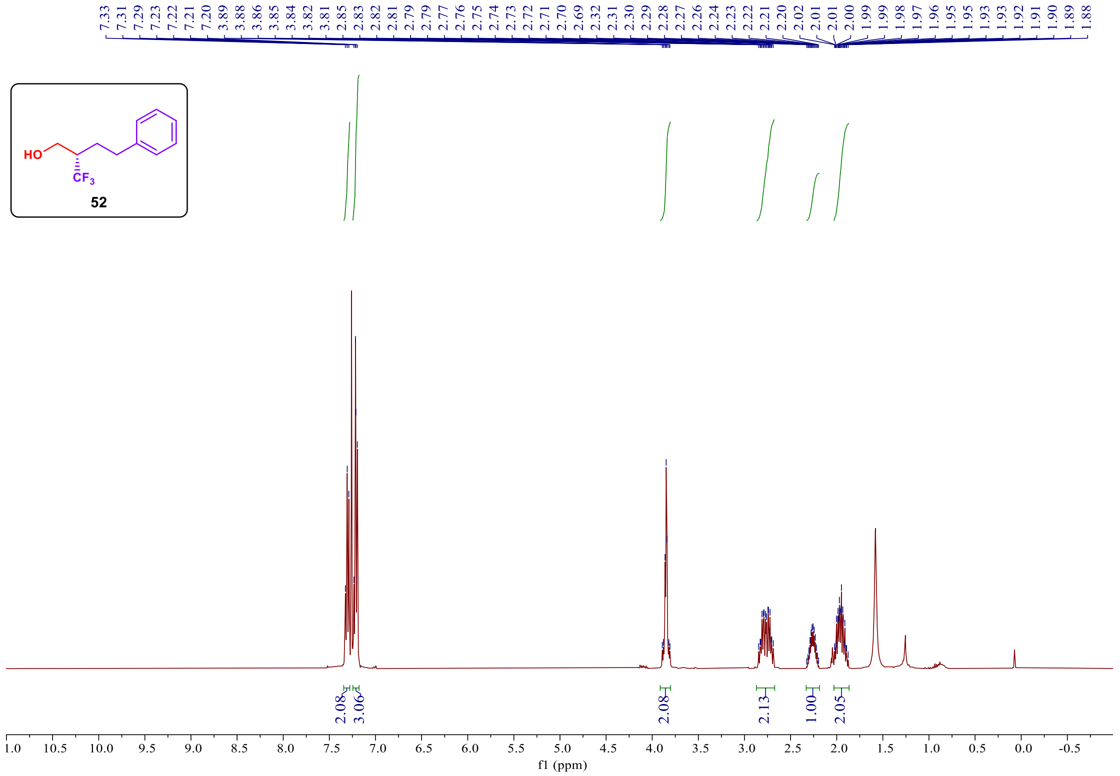

Supplementary Figure 176. ^{1}H NMR Spectrum of Compound **52** (400 MHz, $CDCl_3$)

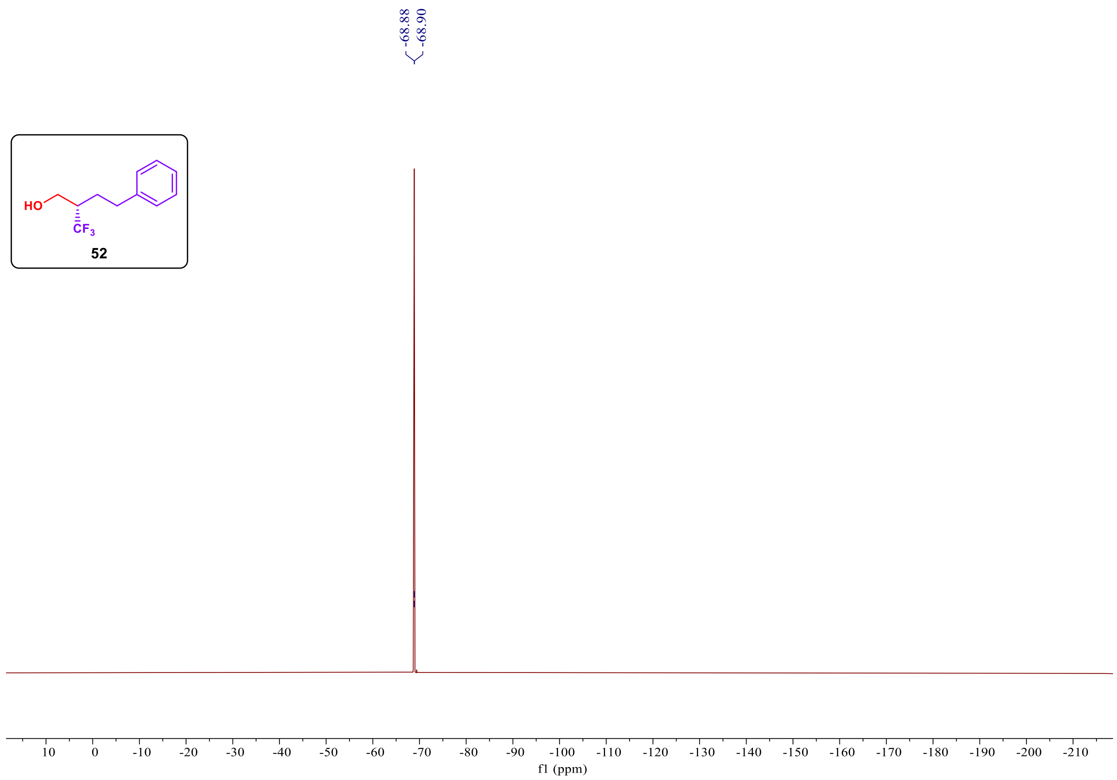

Supplementary Figure 177. ^{19}F NMR Spectrum of Compound **52** (376 MHz, $CDCl_3$)

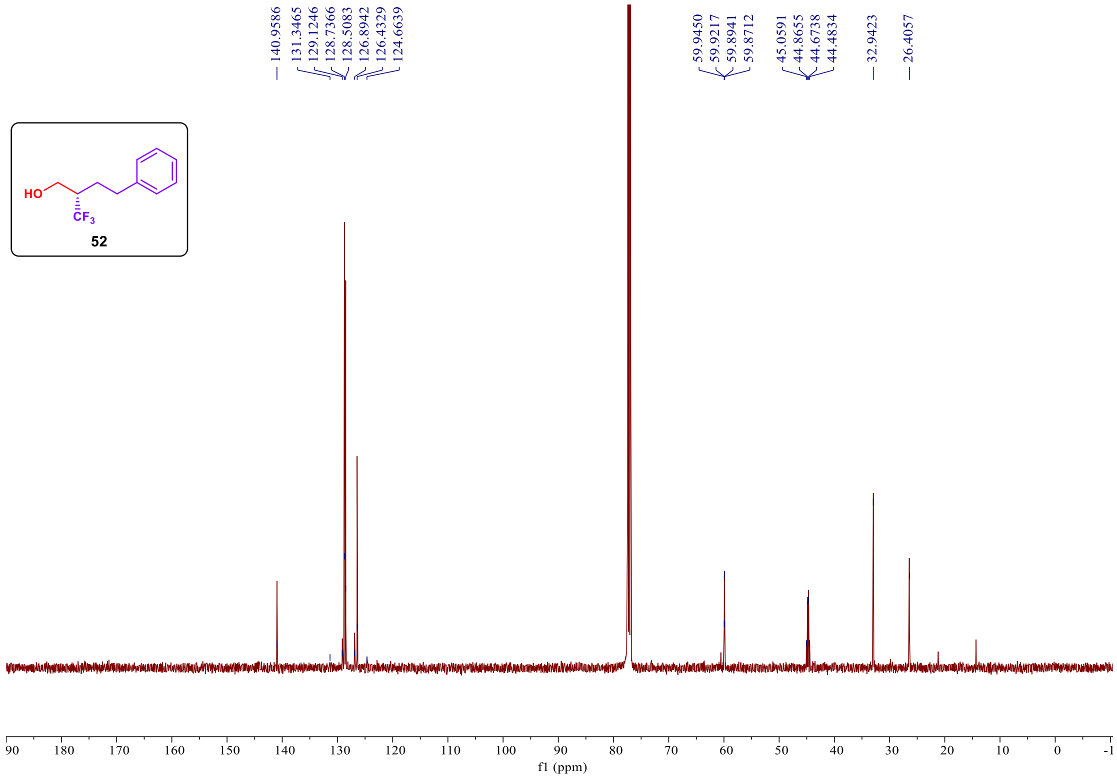

Supplementary Figure 178. ^{13}C NMR Spectrum of Compound **52** (126 MHz, $CDCl_3$)

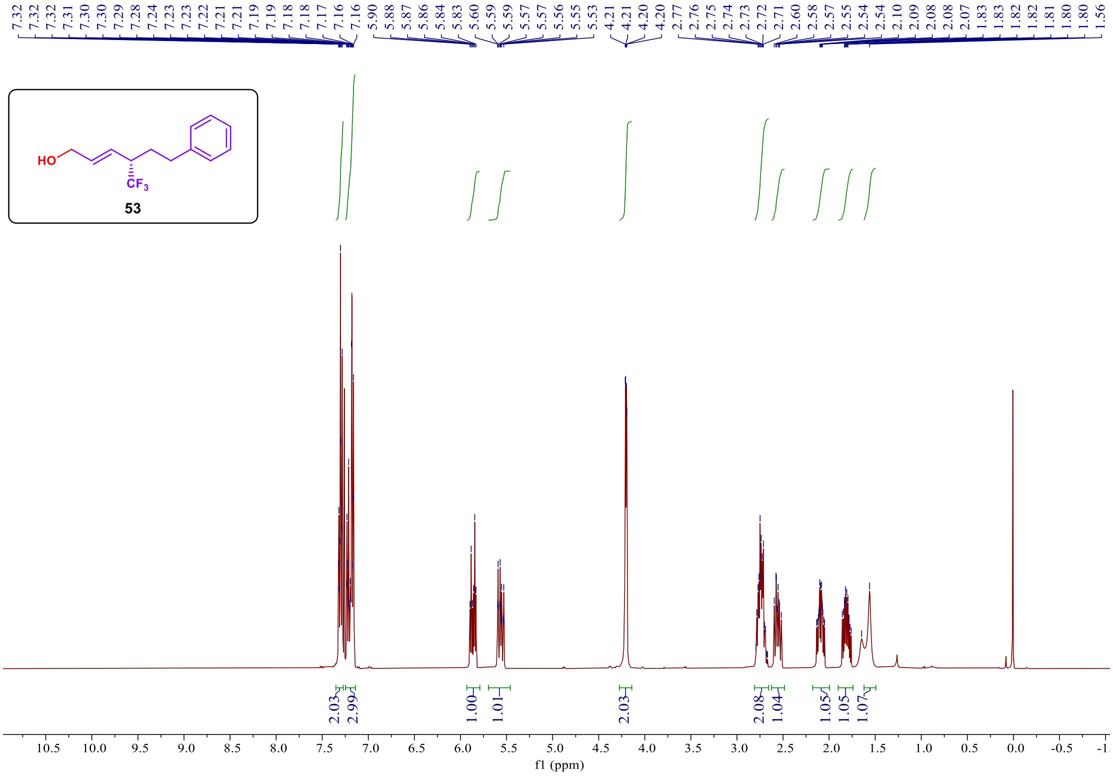

Supplementary Figure 179. ^{1}H NMR Spectrum of Compound **53** (400 MHz, $CDCl_3$)

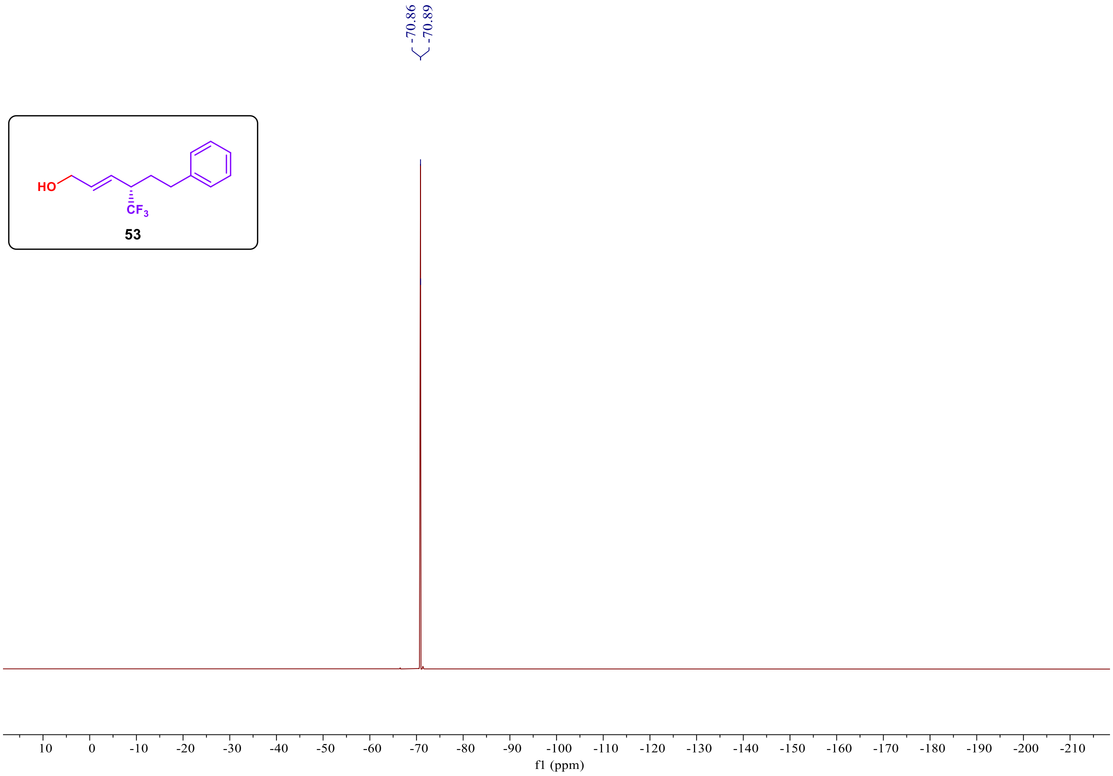

Supplementary Figure 180. ^{19}F NMR Spectrum of Compound **53** (376 MHz, $CDCl_3$)

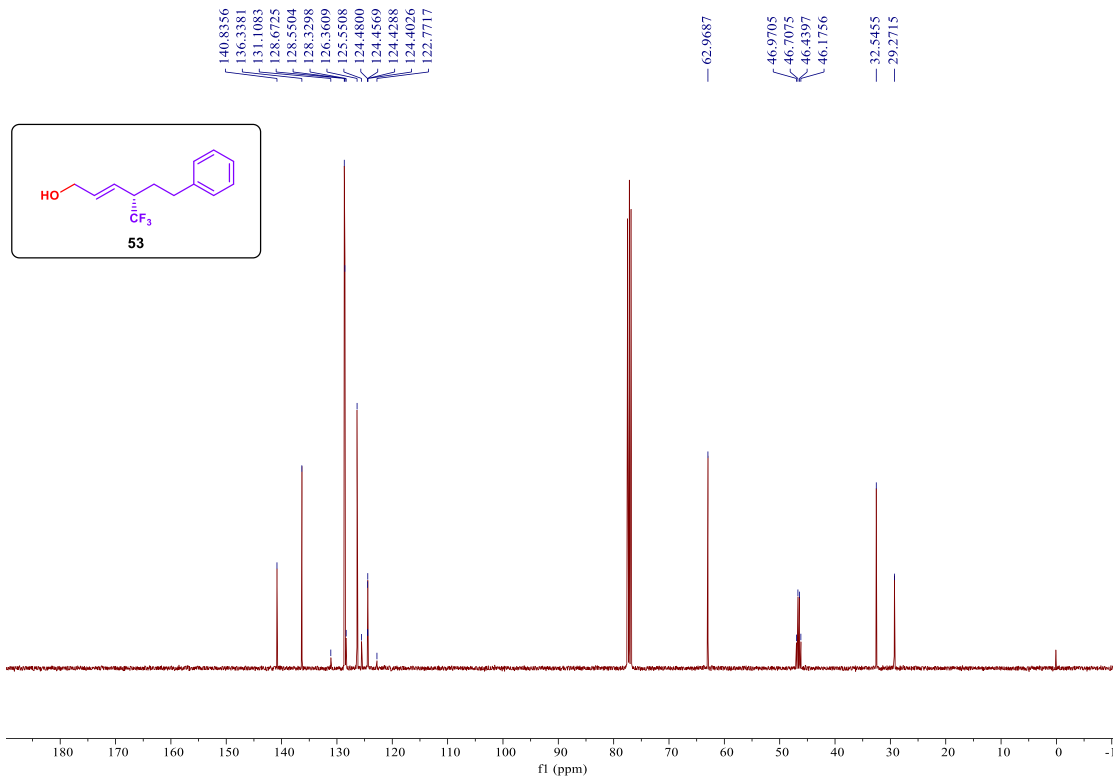

Supplementary Figure 181. ^{13}C NMR Spectrum of Compound **53** (101 MHz, $CDCl_3$)

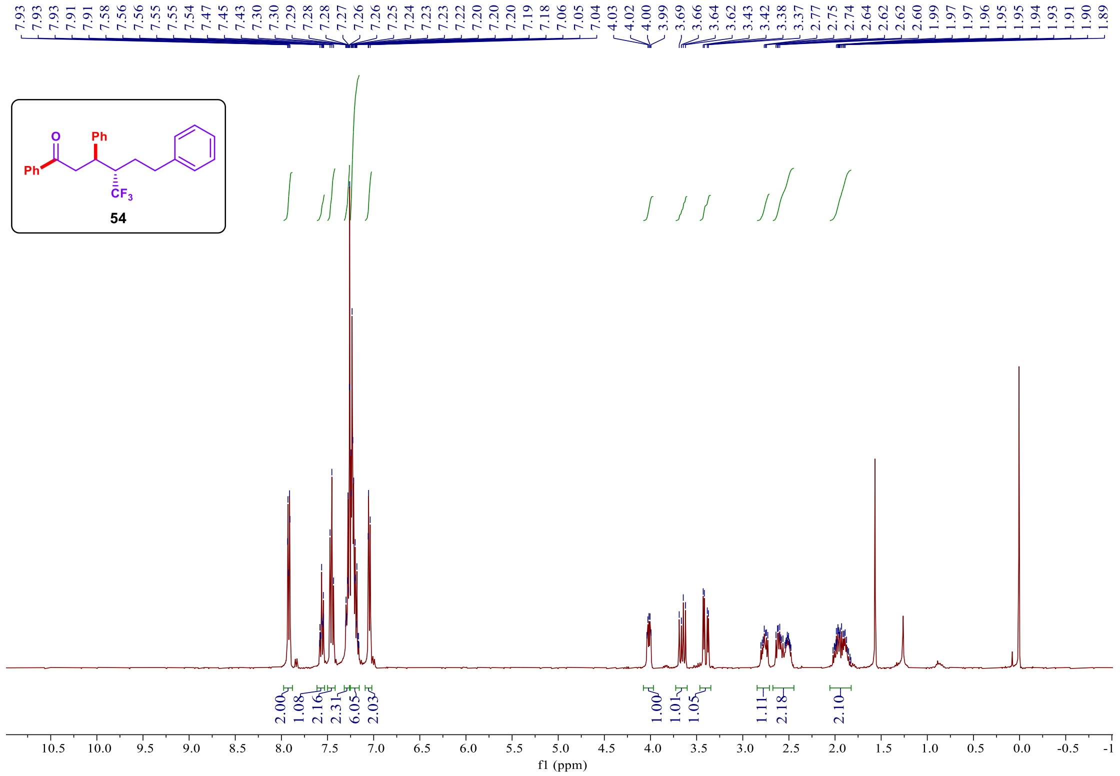

Supplementary Figure 182. ^{1}H NMR Spectrum of Compound **54** (400 MHz, $CDCl_3$)

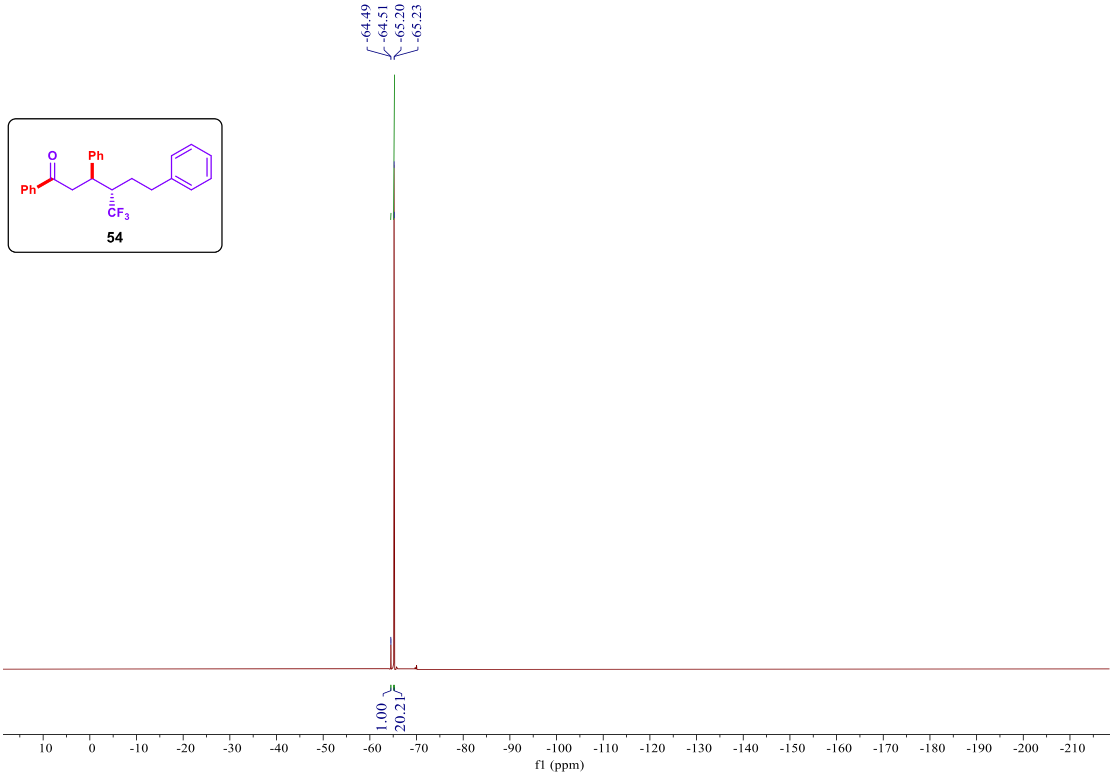

Supplementary Figure 183. ^{19}F NMR Spectrum of Compound **54** (376 MHz, $CDCl_3$)

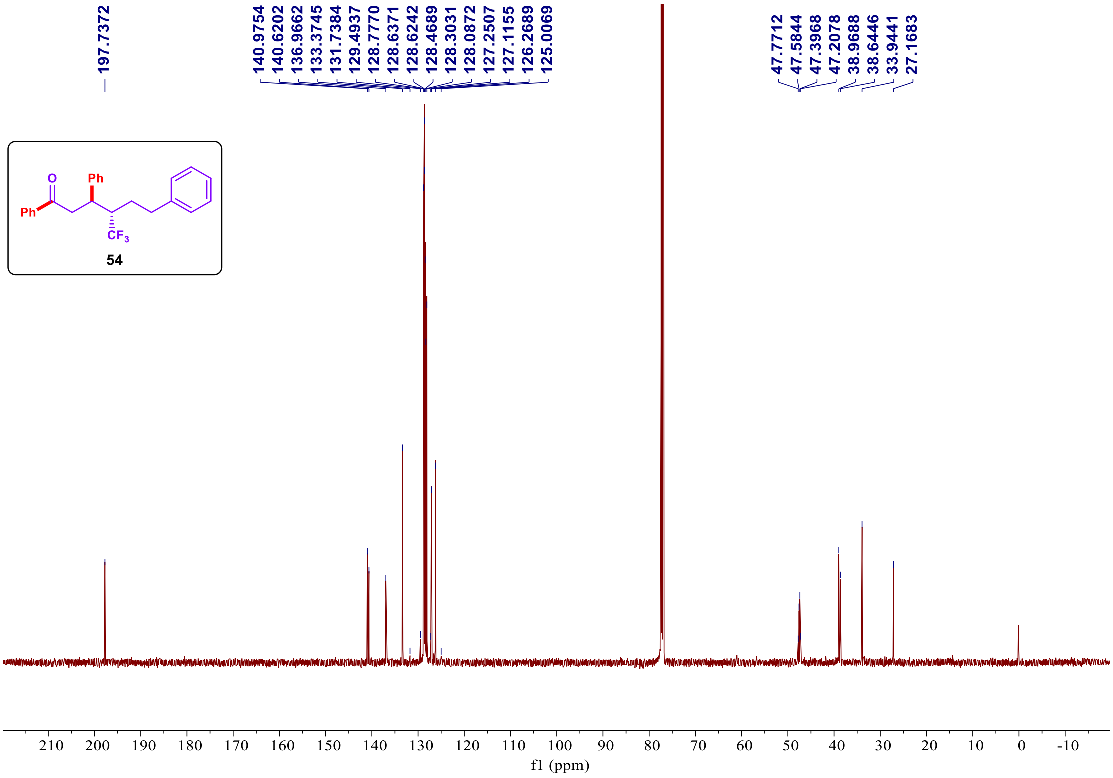

Supplementary Figure 184. ^{13}C NMR Spectrum of Compound **54** (126 MHz, $CDCl_3$)

HPLC Data

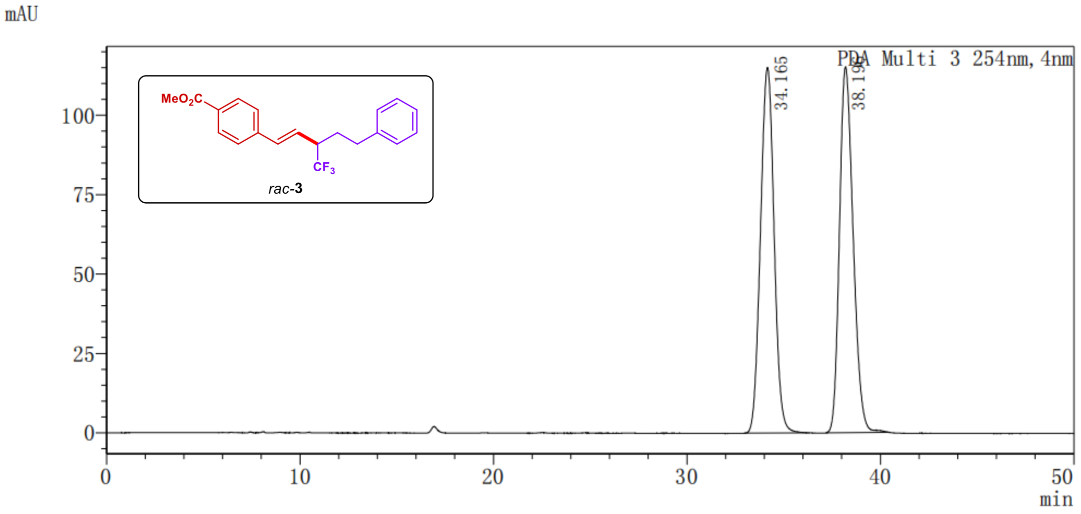

Peak#	Ret. Time	Area	Area#
1	34.165	5626379	49.886
2	38.196	5652194	50.114
Total		11278574	100.000

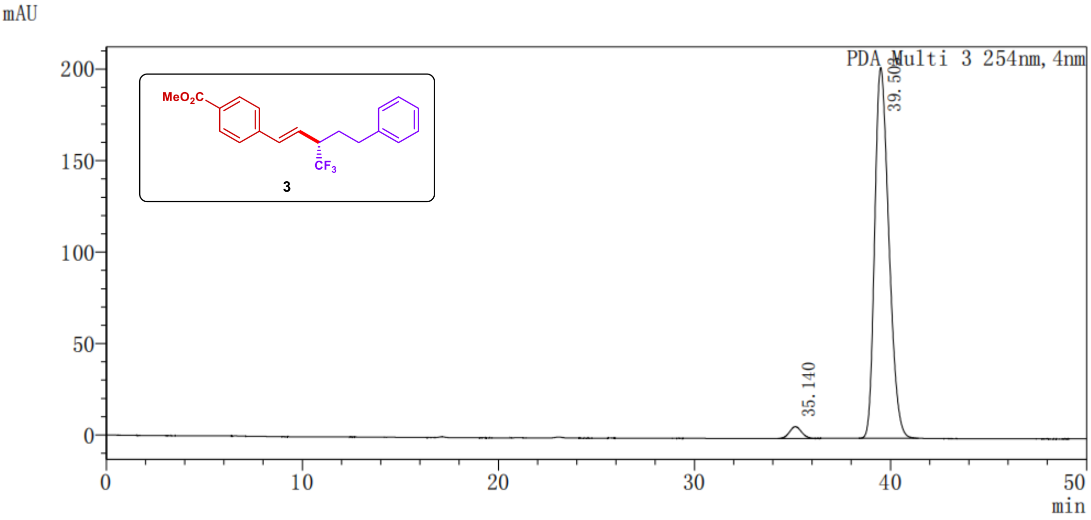

Peak#	Ret. Time	Area	Area#
1	35.140	276747	2.651
2	39.503	10163648	97.349
Total		10440395	100.000

Supplementary Figure 185. Chiral HPLC analysis of Compound **3**

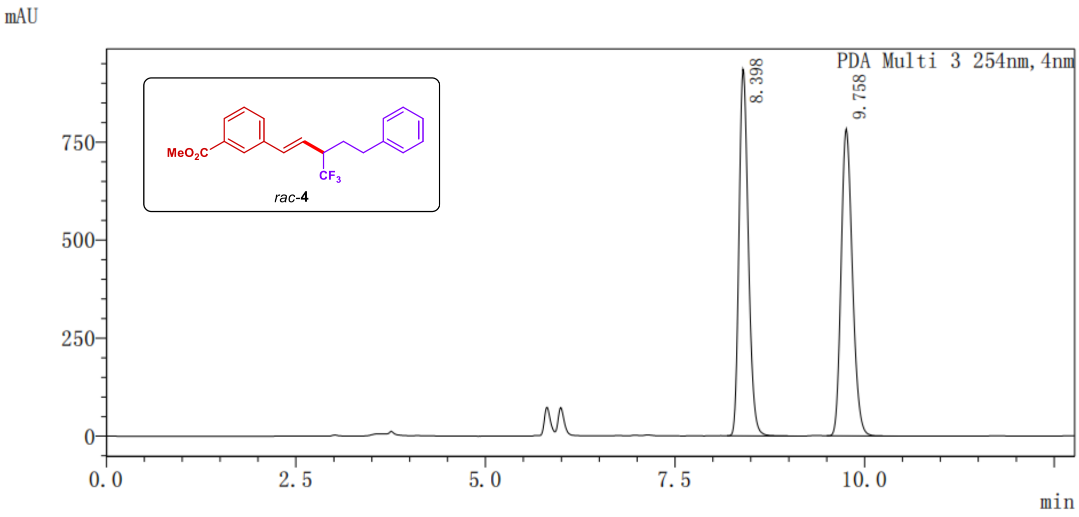

Peak#	Ret. Time	Area	Area#
1	8.398	8035682	49.716
2	9.758	8127597	50.284
Total		16163279	100.000

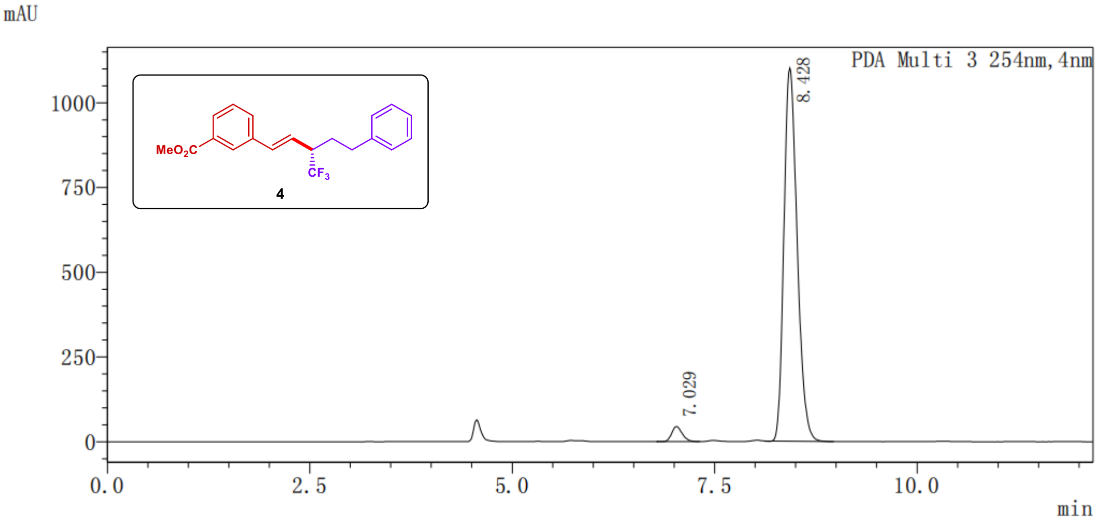

Peak#	Ret. Time	Area	Area#
1	7.029	400386	3.097
2	8.428	12526929	96.903
Total		12927315	100.000

Supplementary Figure 186. Chiral HPLC analysis of Compound **4**

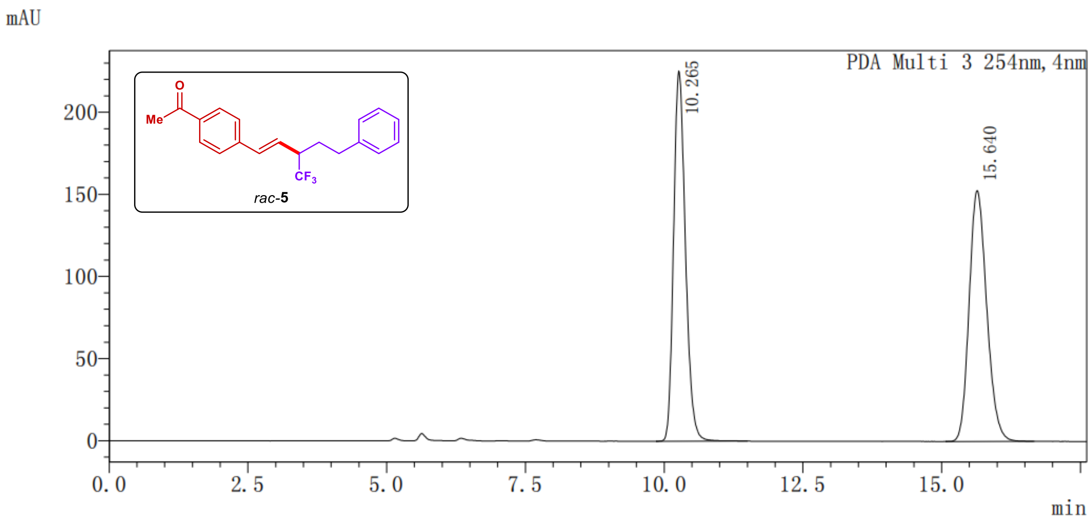

Peak#	Ret. Time	Area	Area#
1	10.265	3344965	49.846
2	15.640	3365651	50.154
Total		6710616	100.000

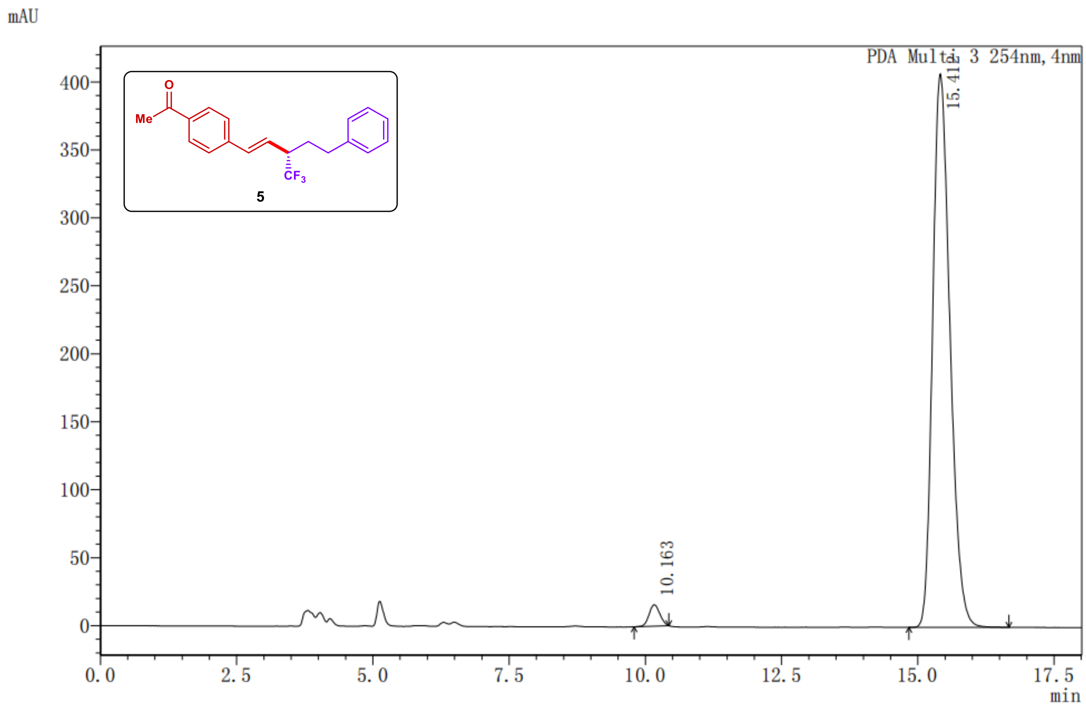

Peak#	Ret. Time	Area	Area#
1	10.163	216988	2.358
2	15.412	8985974	97.642
Total		9202962	100.000

Supplementary Figure 187. Chiral HPLC analysis of Compound **5**

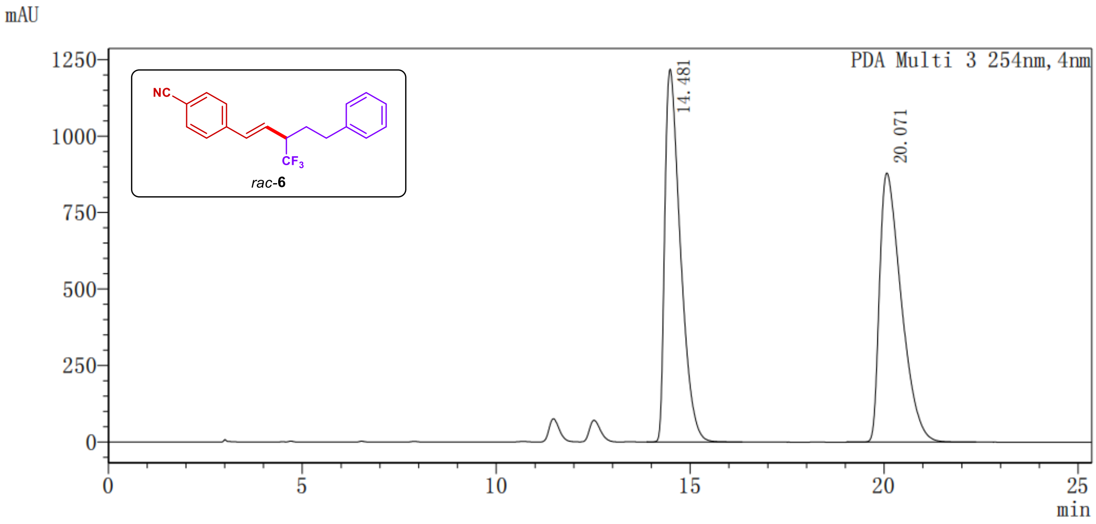

Peak#	Ret. Time	Area	Area#
1	14.481	34199150	49.953
2	20.071	34263567	50.047
Total		68462717	100.000

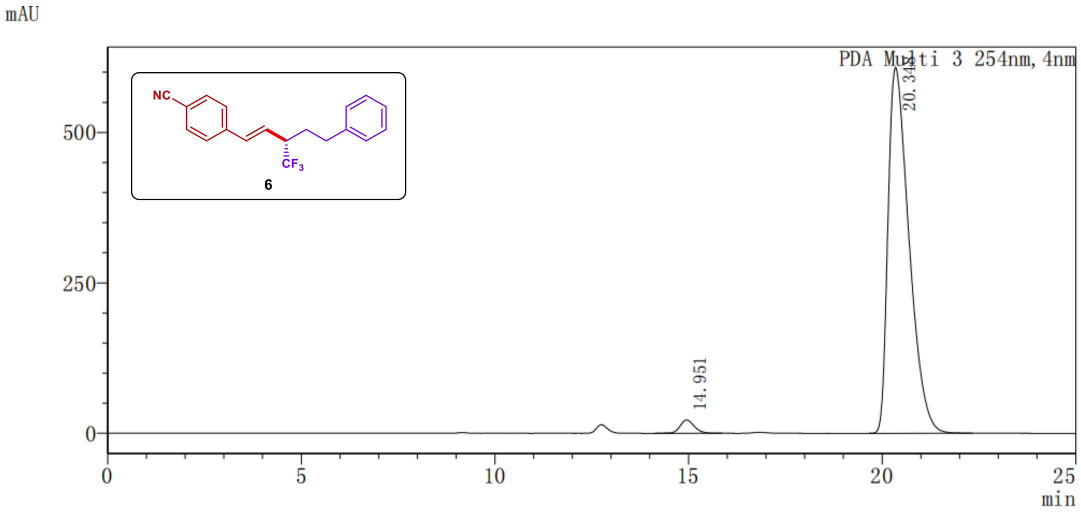

Peak#	Ret. Time	Area	Area#
1	14.951	573312	2.440
2	20.348	22921705	97.560
Total		23495017	100.000

Supplementary Figure 188. Chiral HPLC analysis of Compound **6**

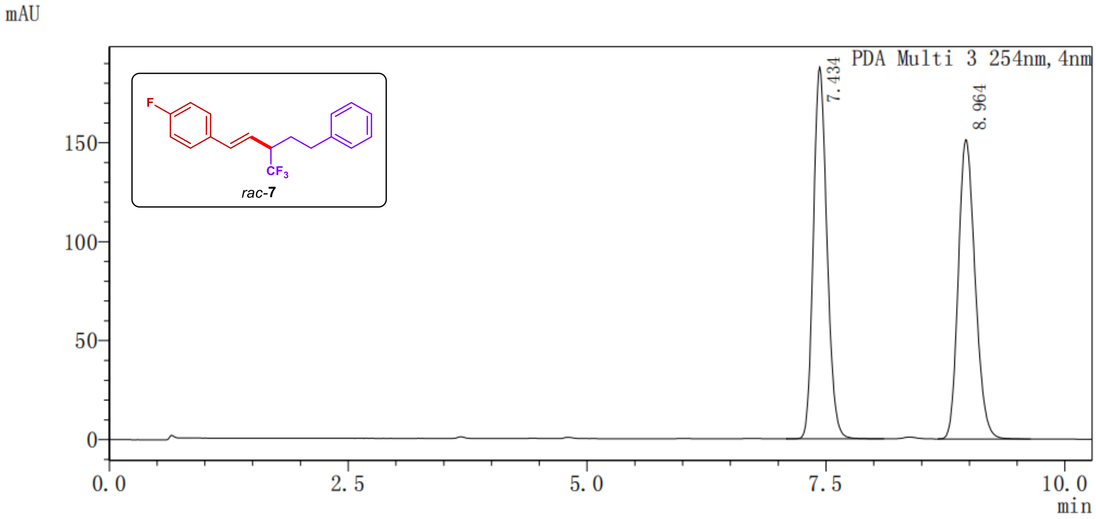

Peak#	Ret. Time	Area	Area#
1	7.434	1859711	50.075
2	8.964	1854129	49.925
Total		3713840	100.000

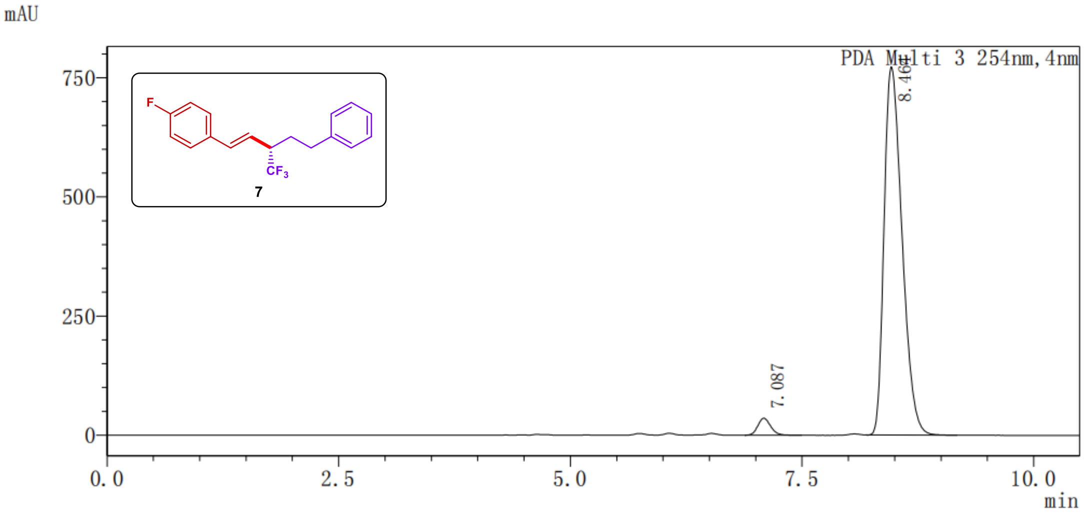

Peak#	Ret. Time	Area	Area#
1	7.087	344968	3.204
2	8.464	10423404	96.796
Total		10768373	100.000

Supplementary Figure 189. Chiral HPLC analysis of Compound **7**

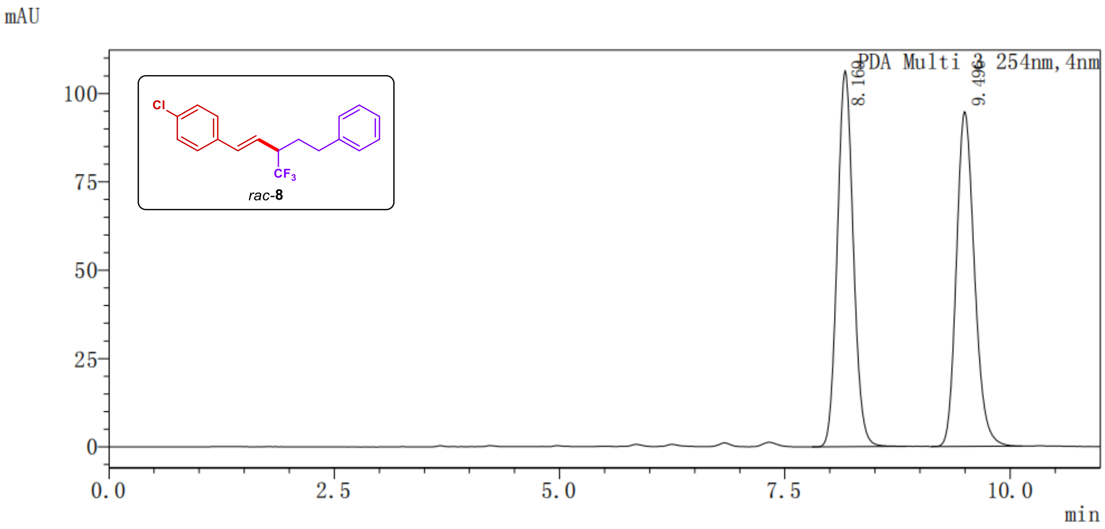

Peak#	Ret. Time	Area	Area#
1	8.169	1310475	49.799
2	9.496	1321032	50.201
Total		2631507	100.000

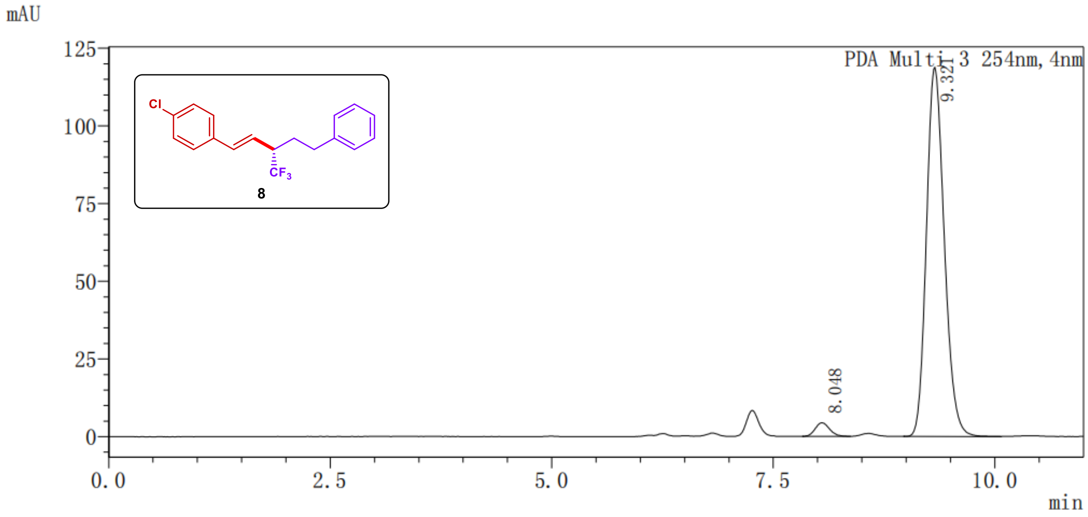

Peak#	Ret. Time	Area	Area#
1	8.048	48948	2.855
2	9.321	1665215	97.145
Total		1714163	100.000

Supplementary Figure 190. Chiral HPLC analysis of Compound **8**

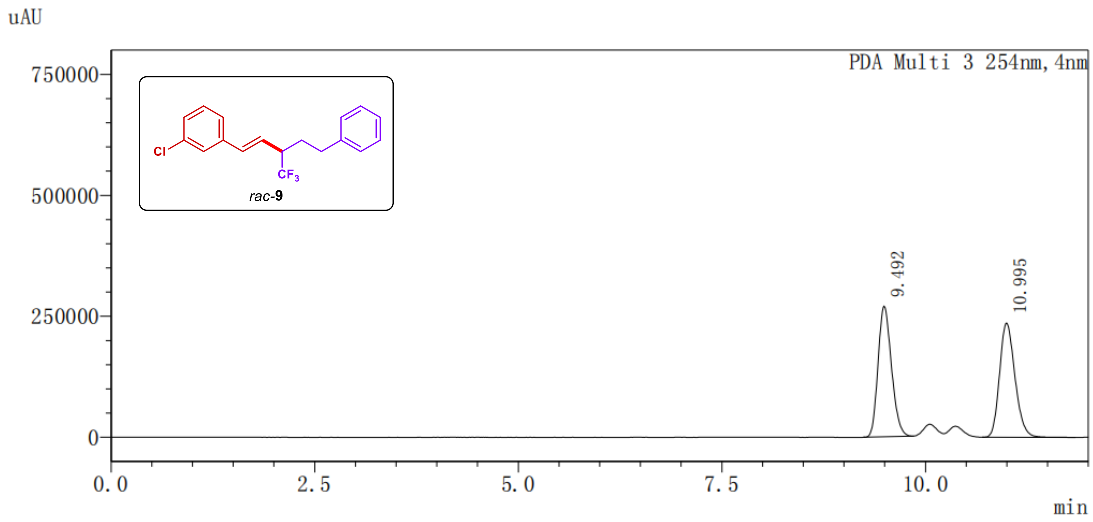

Peak#	Ret. Time	Area	Area#
1	9.492	3099415	49.962
2	10.995	3104175	50.038
Total		6203590	100.000

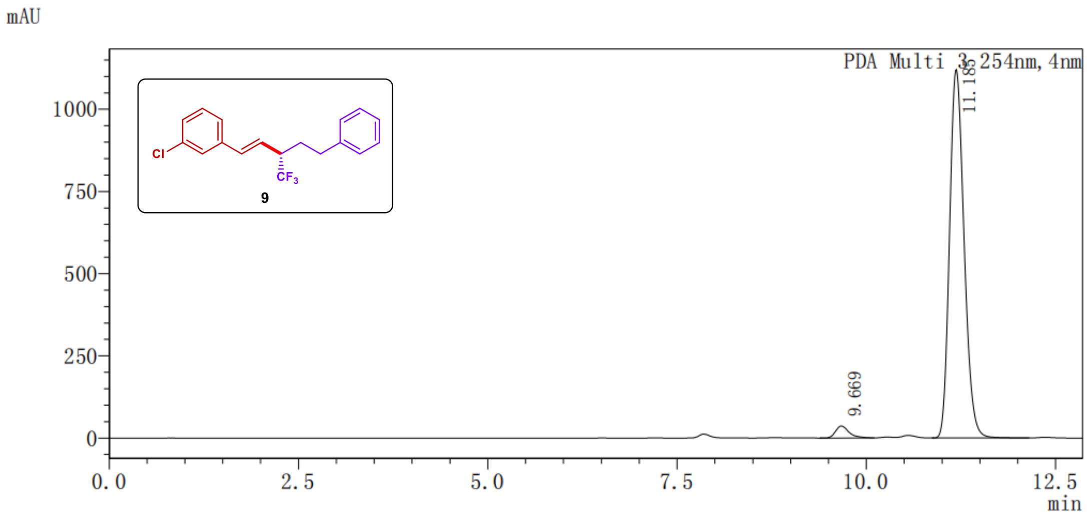

Peak#	Ret. Time	Area	Area#
1	9.669	449254	2.925
2	11.185	14909908	97.075
Total		15359162	100.000

Supplementary Figure 191. Chiral HPLC analysis of Compound **9**

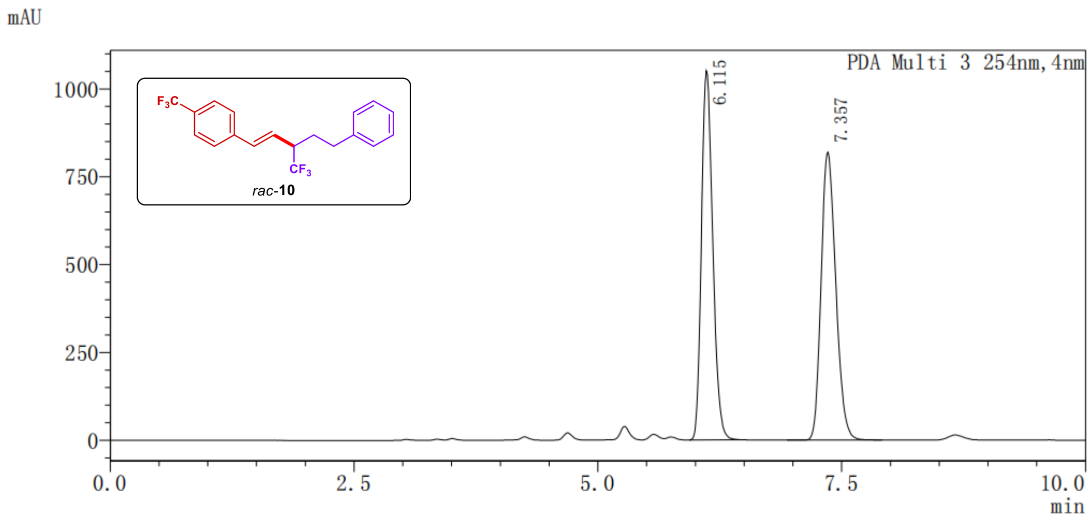

Peak#	Ret. Time	Area	Area#
1	6.115	8465721	49.702
2	7.357	8567130	50.298
Total		17032851	100.000

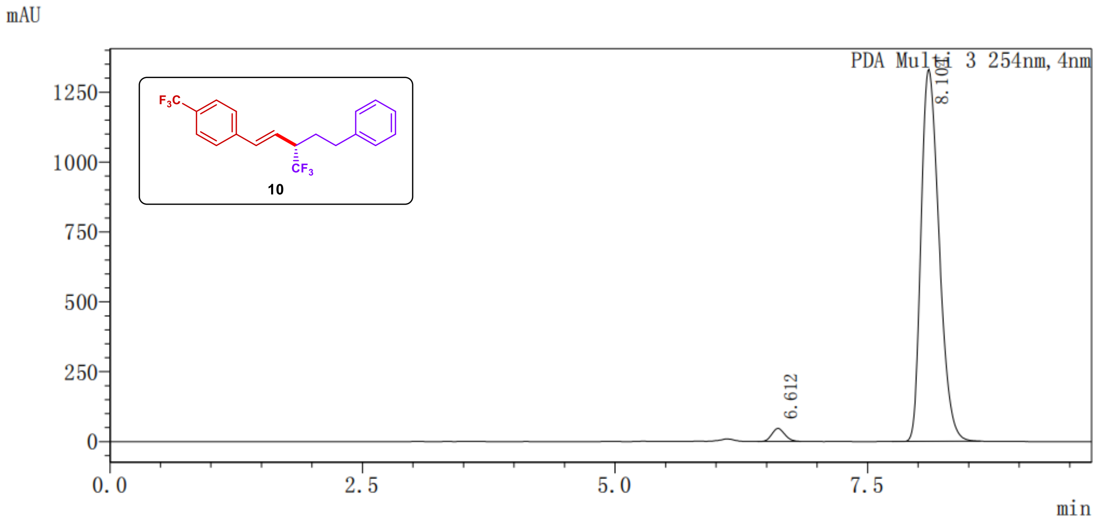

Peak#	Ret. Time	Area	Area#
1	6.612	411026	2.433
2	8.104	16481241	97.567
Total		16892267	100.000

Supplementary Figure 192. Chiral HPLC analysis of Compound **10**

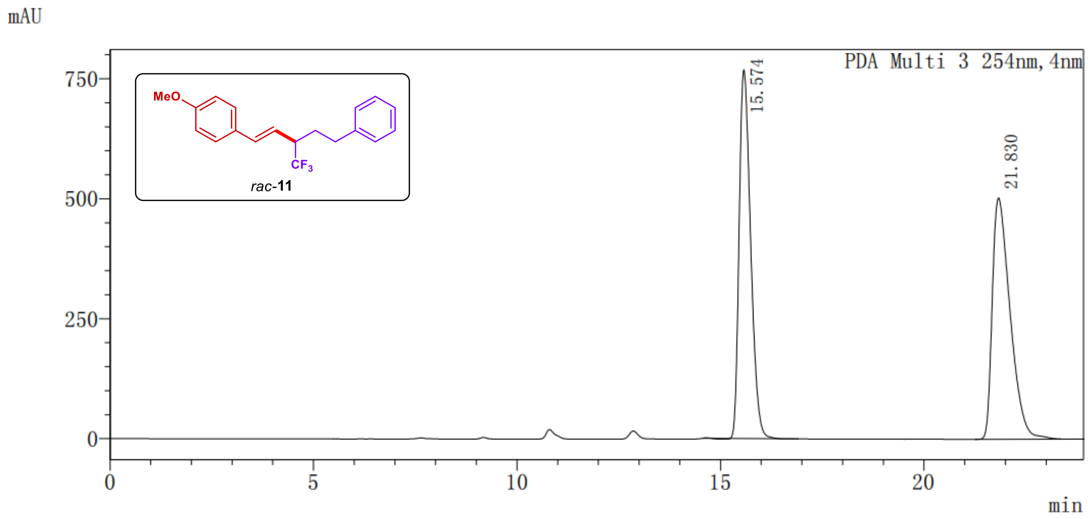

Peak#	Ret. Time	Area	Area#
1	15.574	15043059	49.579
2	21.830	15298264	50.421
Total		30341323	100.000

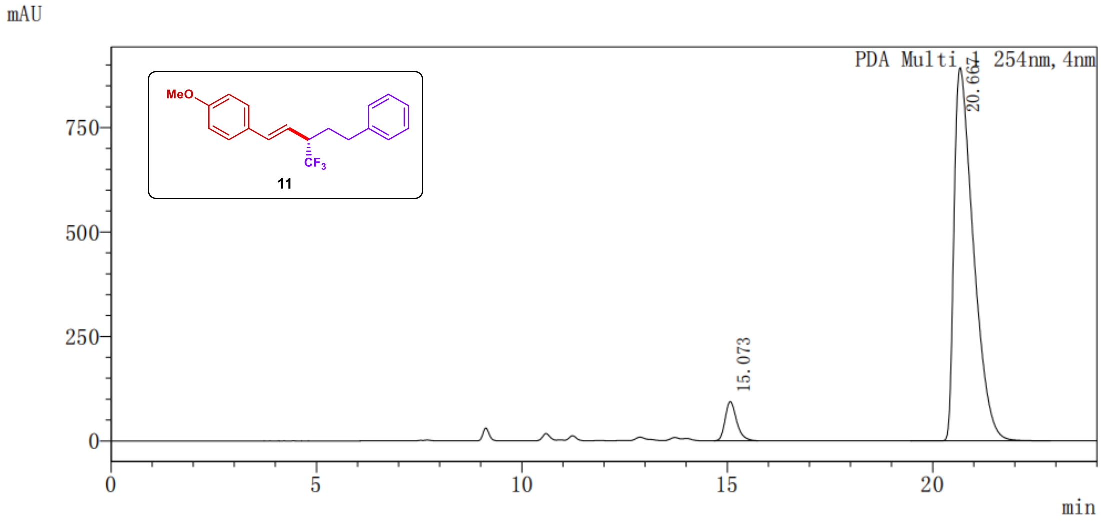

Peak#	Ret. Time	Area	Area#
1	15.073	1814305	5.848
2	20.667	29208003	94.152
Total		31022308	100.000

Supplementary Figure 193. Chiral HPLC analysis of Compound **11**

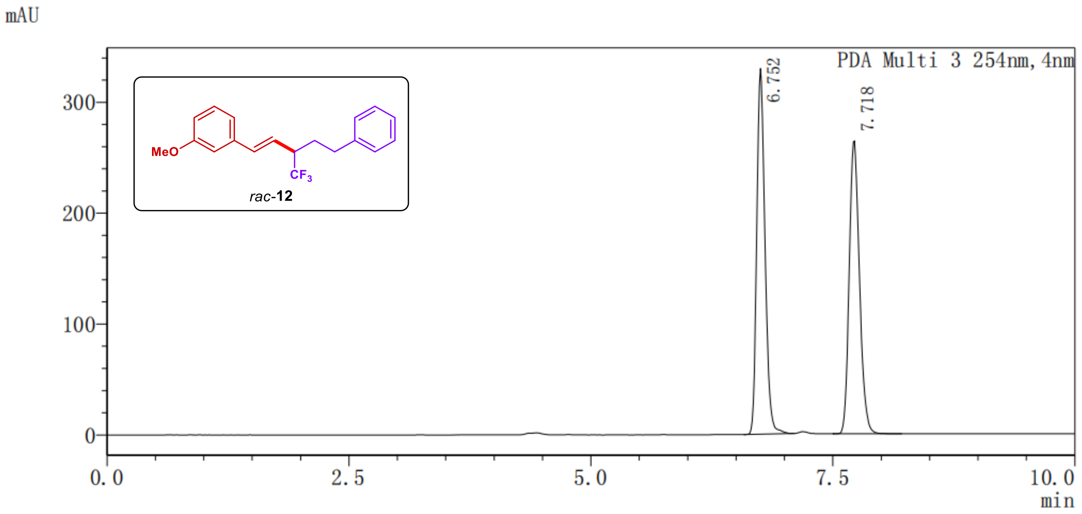

Peak#	Ret. Time	Area	Area#
1	6.752	1955398	50.171
2	7.718	1942084	49.829
Total		3897482	100.000

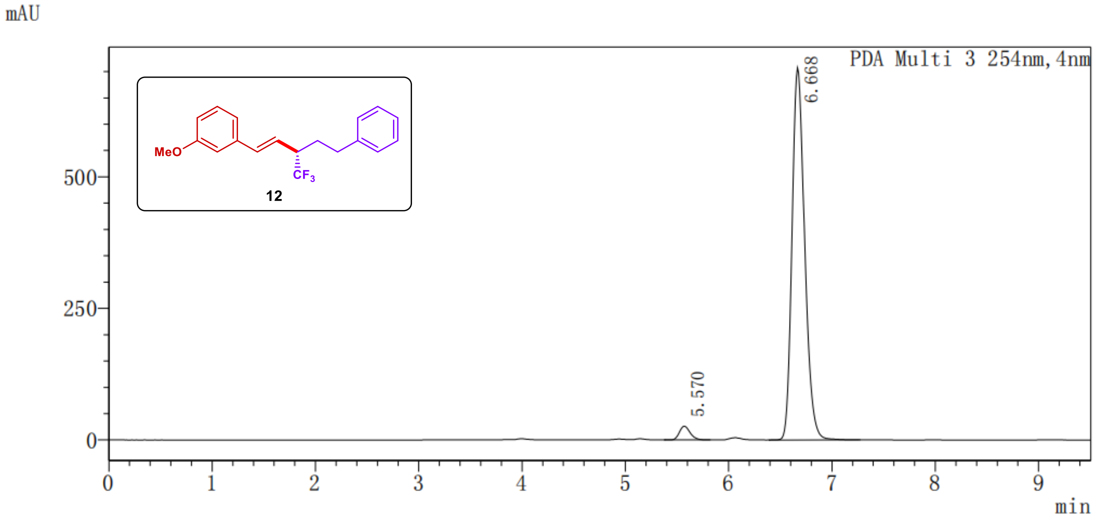

Peak#	Ret. Time	Area	Area#
1	5.570	185250	2.984
2	6.668	6023164	97.016
Total		6208413	100.000

Supplementary Figure 194. Chiral HPLC analysis of Compound **12**

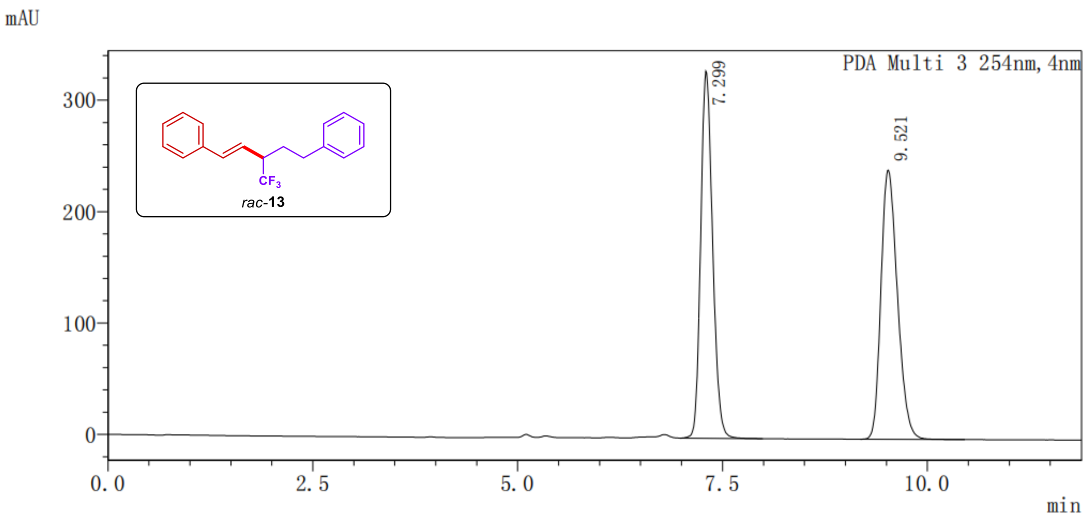

Peak#	Ret. Time	Area	Area#
1	7.299	3332884	49.691
2	9.521	3374351	50.309
Total		6707235	100.000

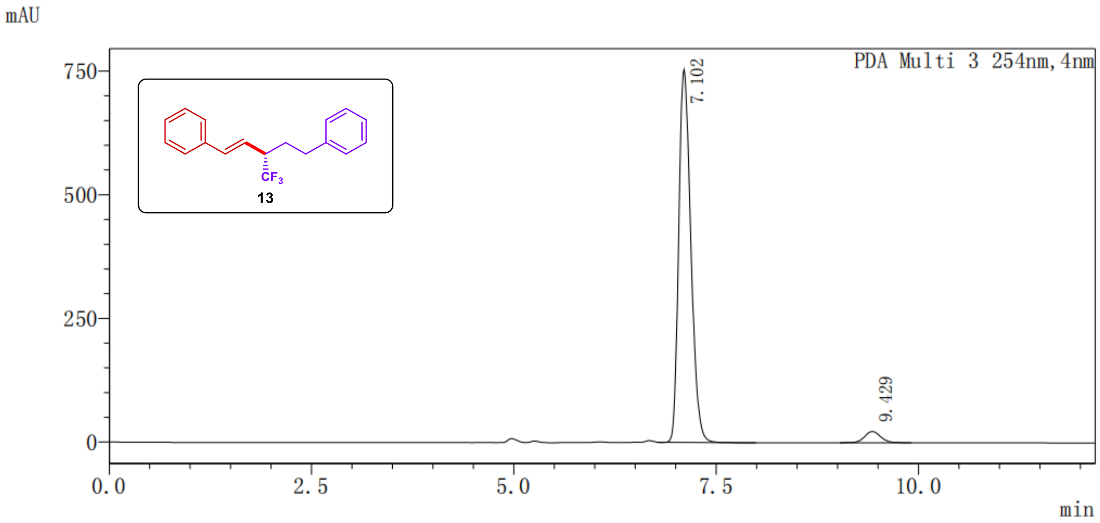

Peak#	Ret. Time	Area	Area#
1	7.102	7825199	96.130
2	9.429	315057	3.870
Total		8140256	100.000

Supplementary Figure 195. Chiral HPLC analysis of Compound **13**

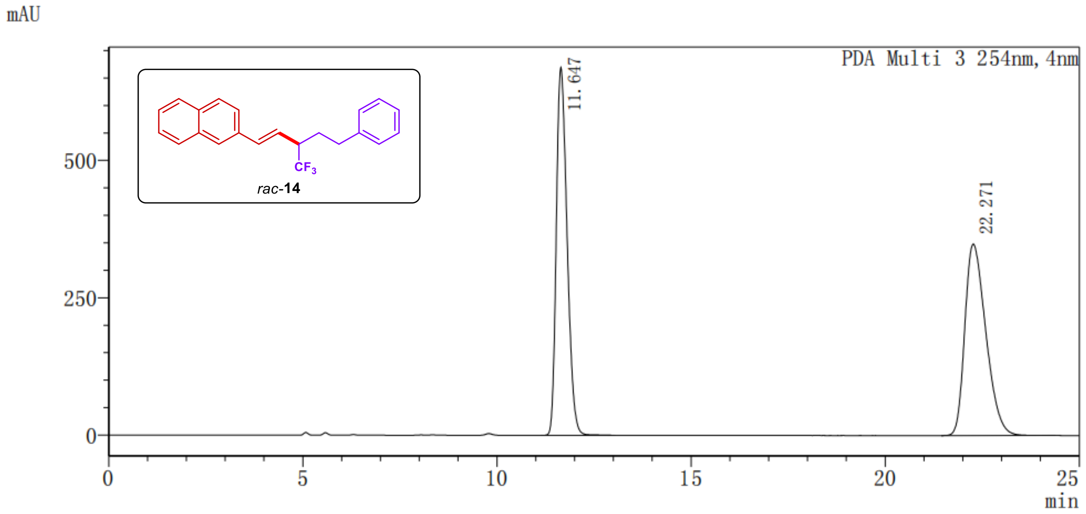

Peak#	Ret. Time	Area	Area#
1	11.647	12925962	49.584
2	22.271	13142992	50.416
Total		26068955	100.000

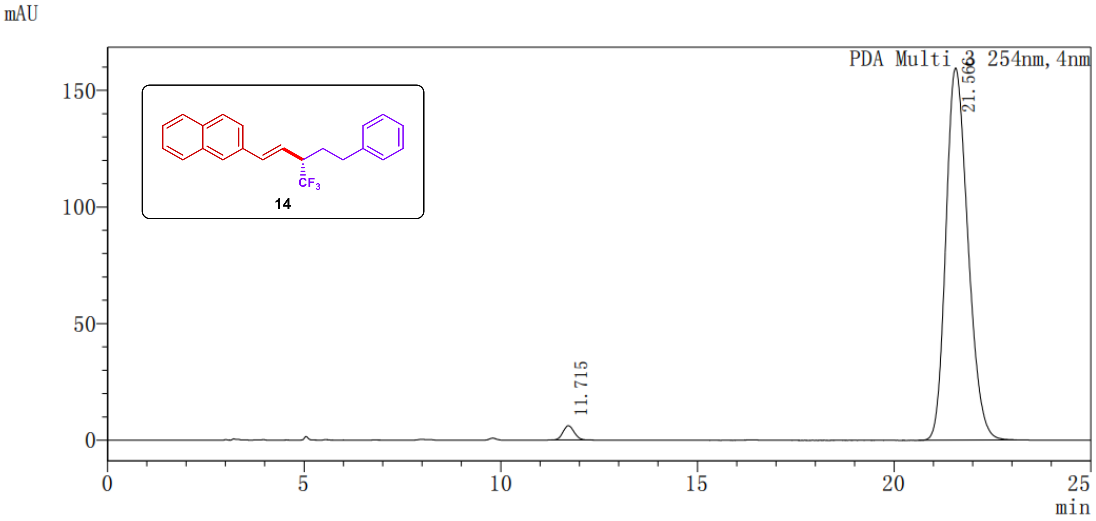

Peak#	Ret. Time	Area	Area#
1	11.715	121589	1.923
2	21.566	6201518	98.077
Total		6323107	100.000

Supplementary Figure 196. Chiral HPLC analysis of Compound **14**

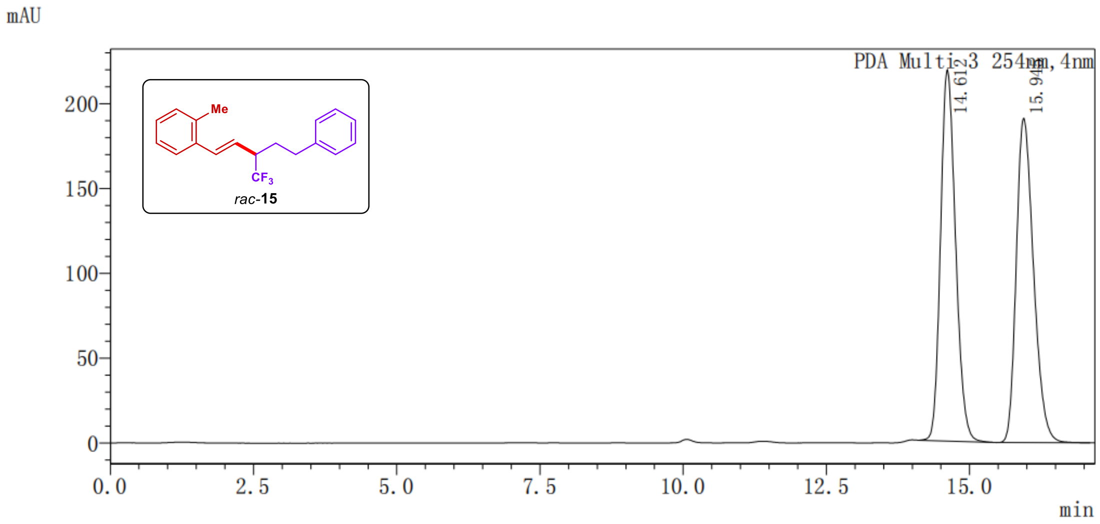

Peak#	Ret. Time	Area	Area#
1	14.612	3994816	49.892
2	15.945	4012156	50.108
Total		8006972	100.000

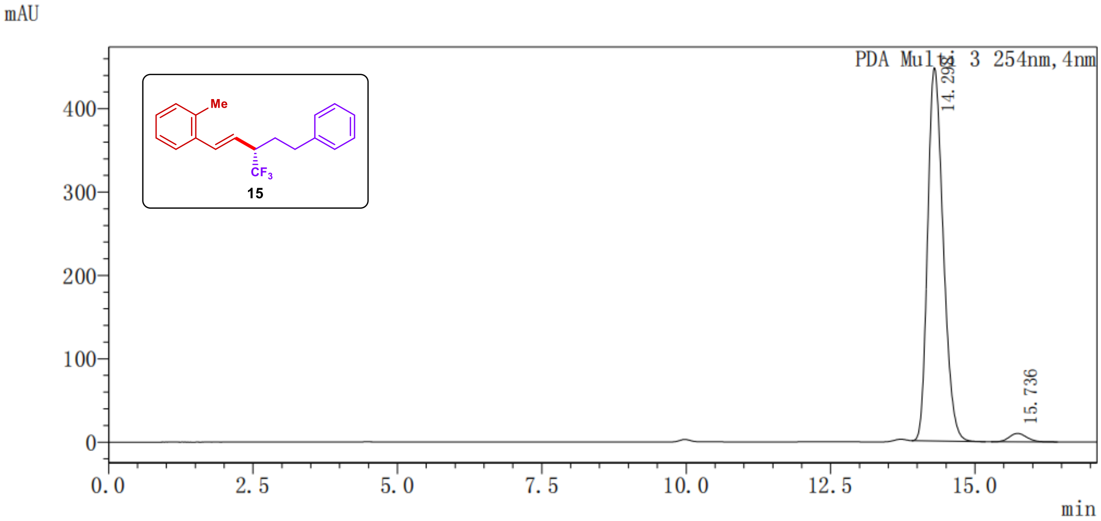

Peak#	Ret. Time	Area	Area#
1	14.298	8386992	97.531
2	15.736	212293	2.469
Total		8599285	100.000

Supplementary Figure 197. Chiral HPLC analysis of Compound **15**

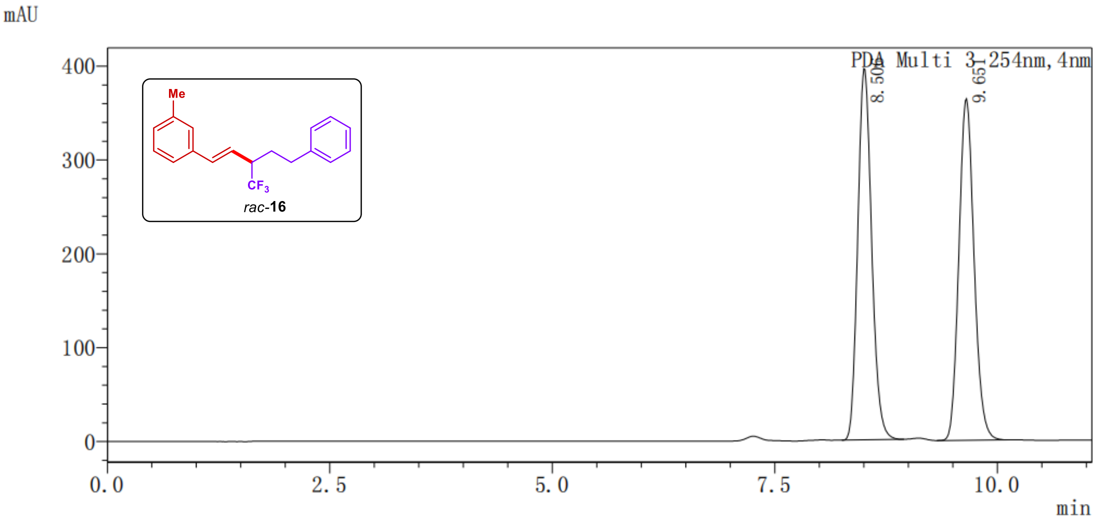

Peak#	Ret. Time	Area	Area#
1	8.506	4295845	49.867
2	9.651	4318674	50.133
Total		8614518	100.000

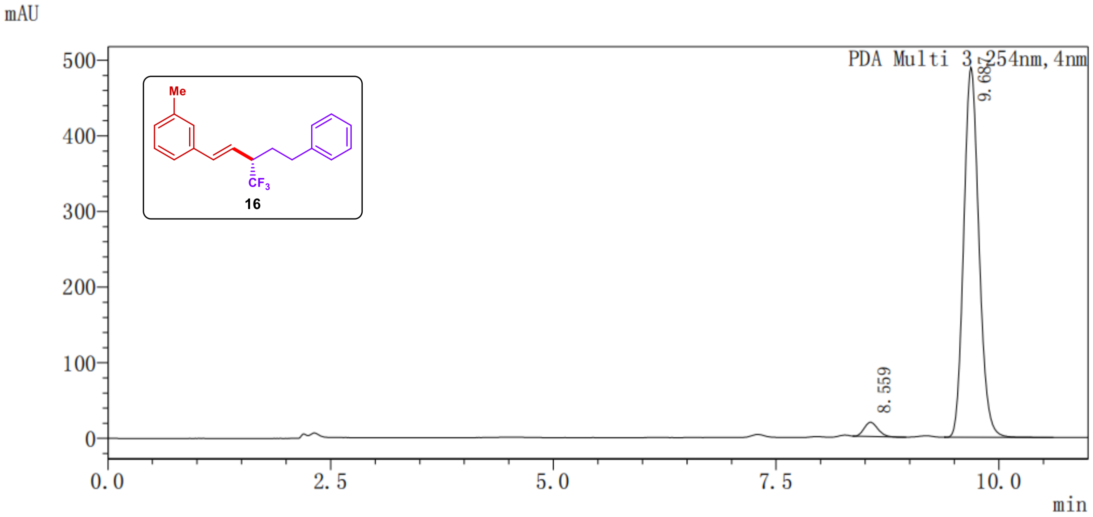

Peak#	Ret. Time	Area	Area#
1	8.559	200395	3.335
2	9.687	5808411	96.665
Total		6008806	100.000

Supplementary Figure 198. Chiral HPLC analysis of Compound **16**

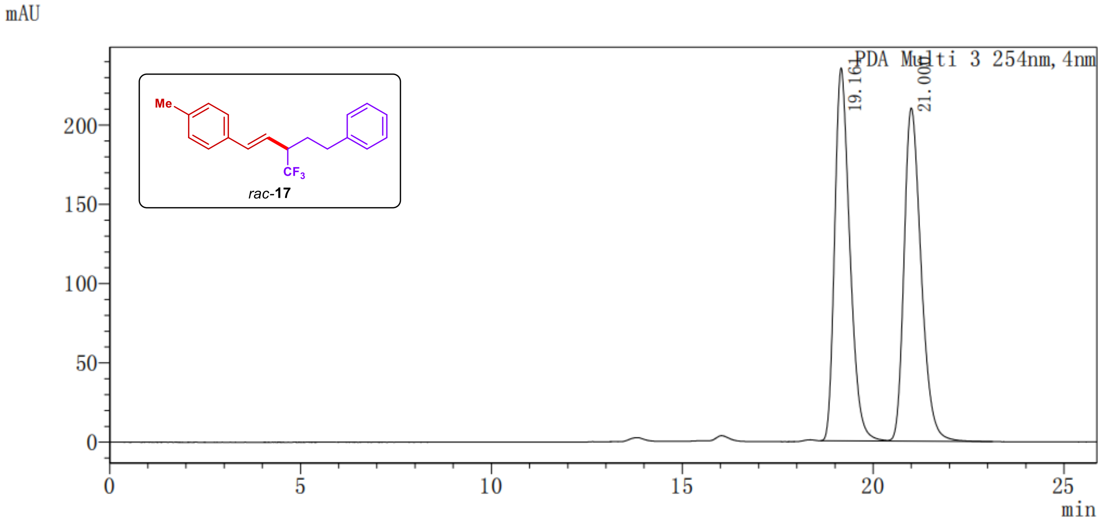

Peak#	Ret. Time	Area	Area#
1	19.161	6489108	49.776
2	21.000	6547627	50.224
Total		13036734	100.000

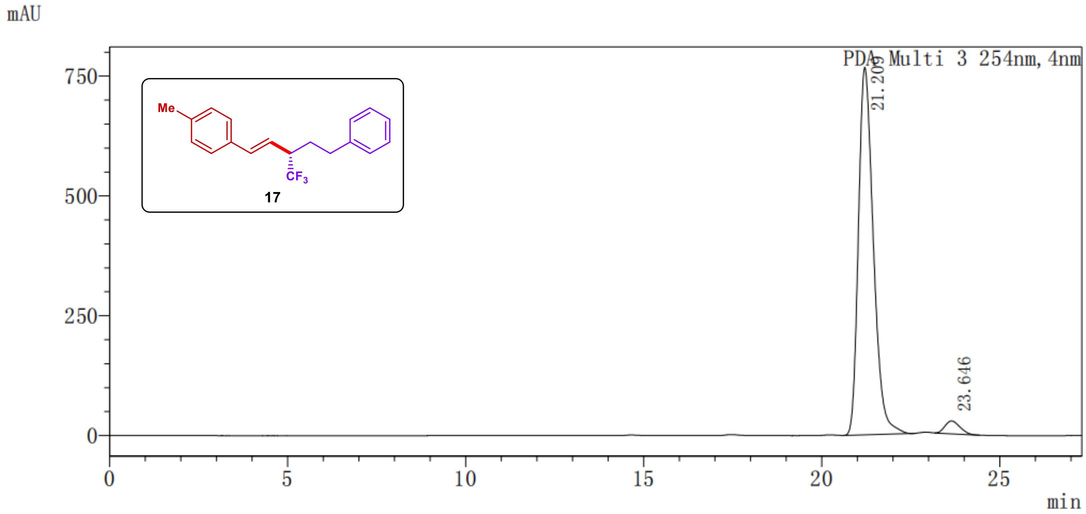

Peak#	Ret. Time	Area	Area#
1	21.209	22301313	96.616
2	23.646	781170	3.384
Total		23082484	100.000

Supplementary Figure 199. Chiral HPLC analysis of Compound **17**

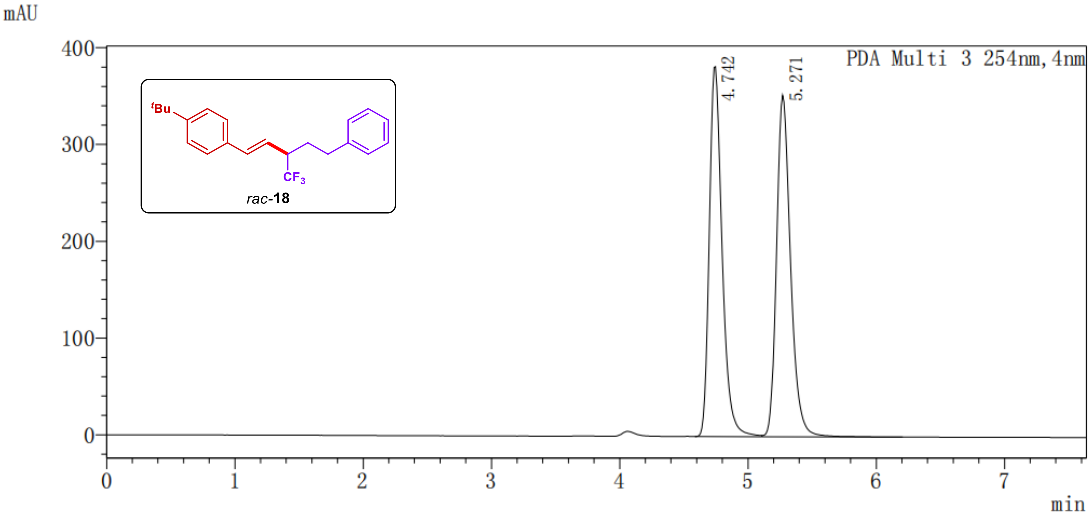

Peak#	Ret. Time	Area	Area#
1	4.742	2671966	49.850
2	5.271	2688042	50.150
Total		5360008	100.000

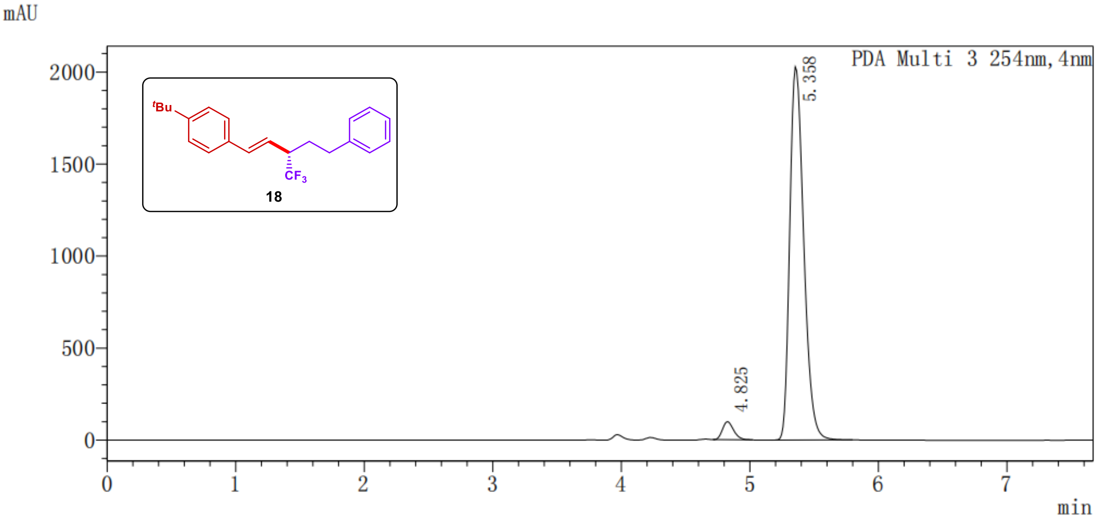

Peak#	Ret. Time	Area	Area#
1	4.825	597287	3.798
2	5.358	15130772	96.202
Total		15728059	100.000

Supplementary Figure 200. Chiral HPLC analysis of Compound **18**

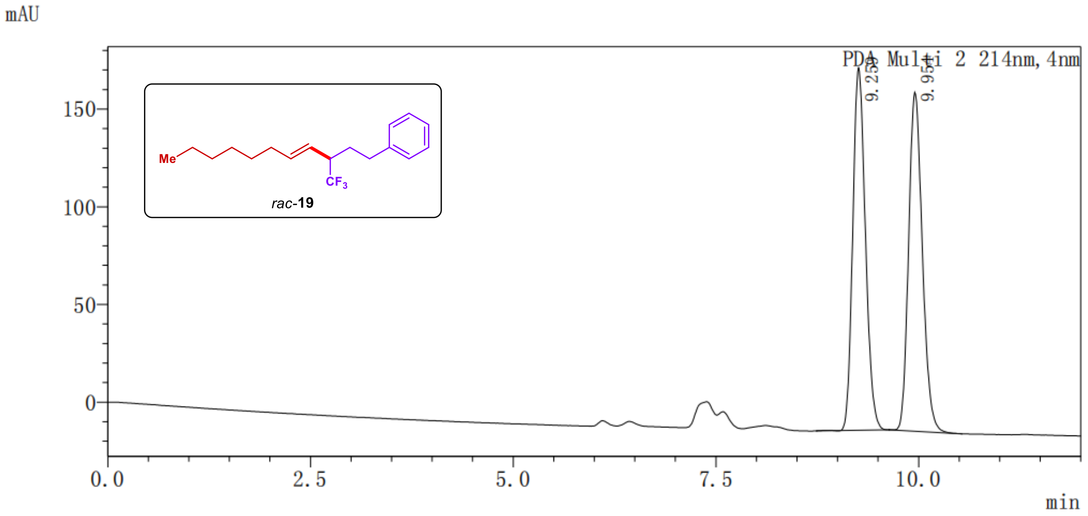

Peak#	Ret. Time	Area	Area#
1	9.259	2018012	49.686
2	9.954	2043492	50.314
Total		4061504	100.000

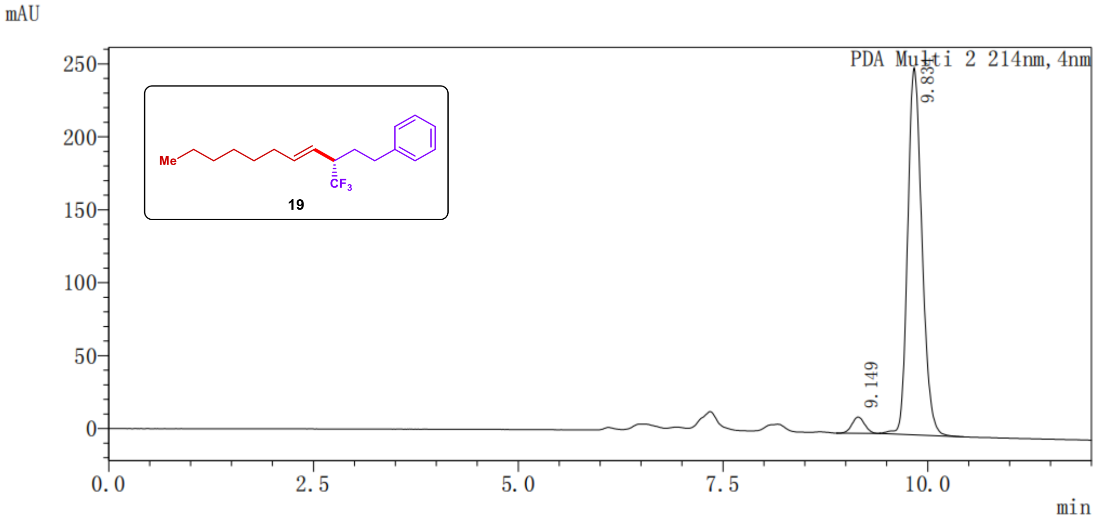

Peak#	Ret. Time	Area	Area#
1	9.149	120969	3.803
2	9.834	3059532	96.197
Total		3180501	100.000

Supplementary Figure 201. Chiral HPLC analysis of Compound **19**

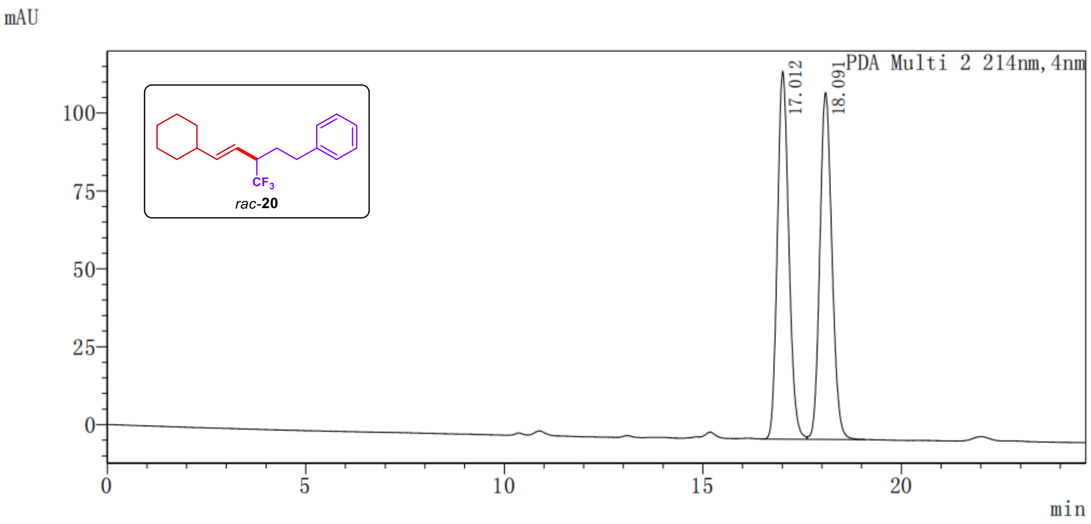

Peak#	Ret. Time	Area	Area#
1	17.012	2361471	50.052
2	18.091	2356553	49.948
Total		4718023	100.000

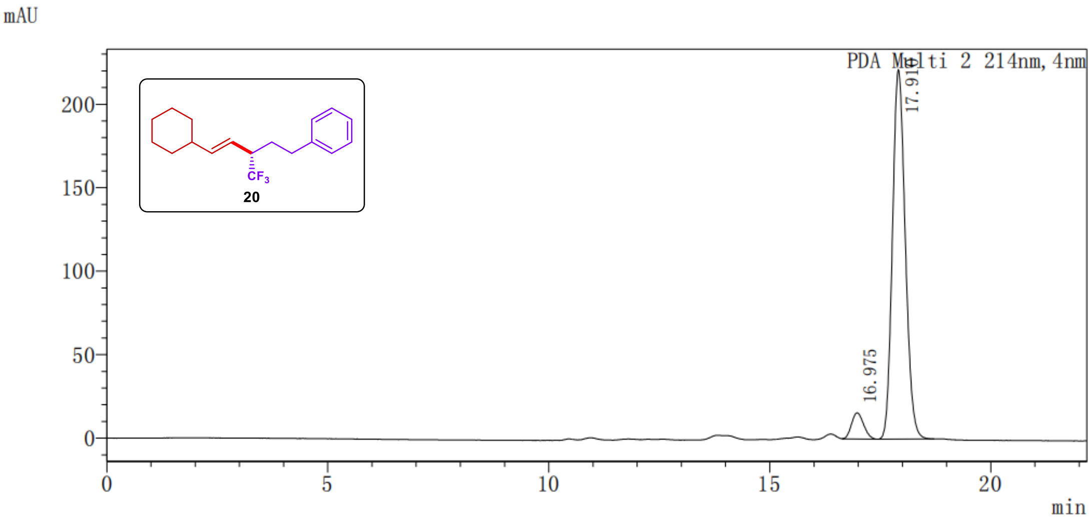

Peak#	Ret. Time	Area	Area#
1	16.975	297515	6.251
2	17.910	4461999	93.749
Total		4759514	100.000

Supplementary Figure 202. Chiral HPLC analysis of Compound **20**

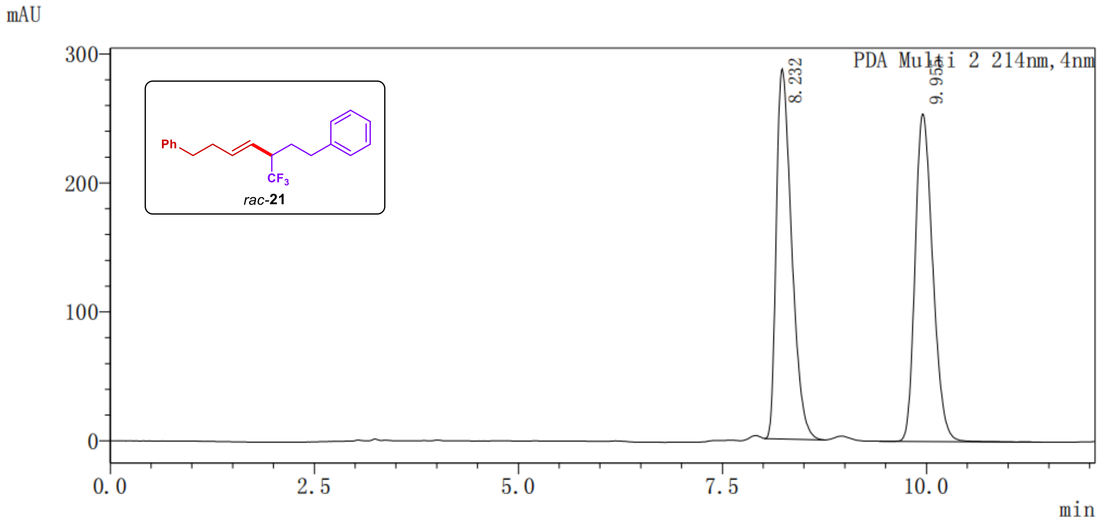

Peak#	Ret. Time	Area	Area#
1	8.232	3758044	49.596
2	9.955	3819232	50.404
Total		7577276	100.000

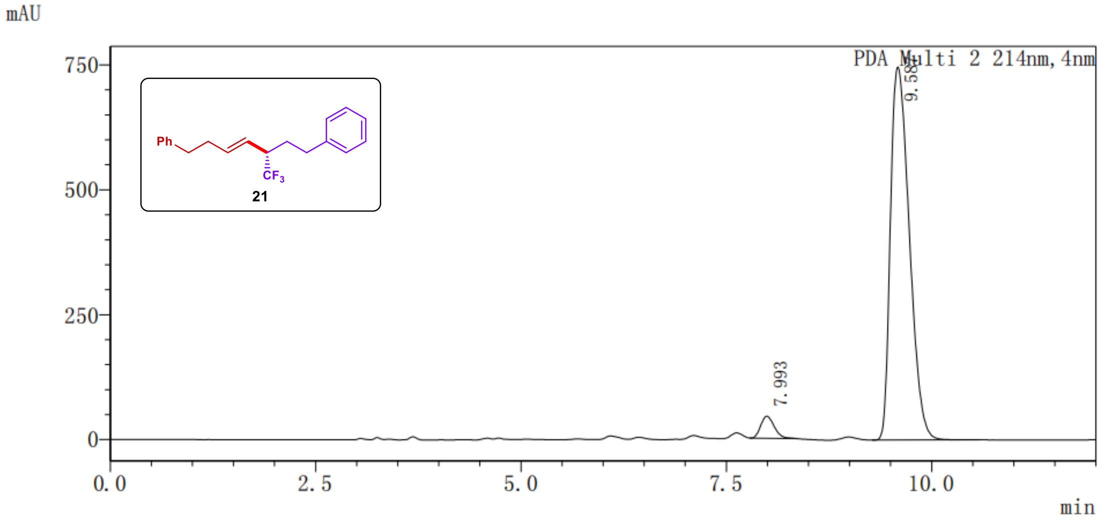

Peak#	Ret. Time	Area	Area#
1	7.993	506047	4.067
2	9.587	11936045	95.933
Total		12442092	100.000

Supplementary Figure 203. Chiral HPLC analysis of Compound **21**

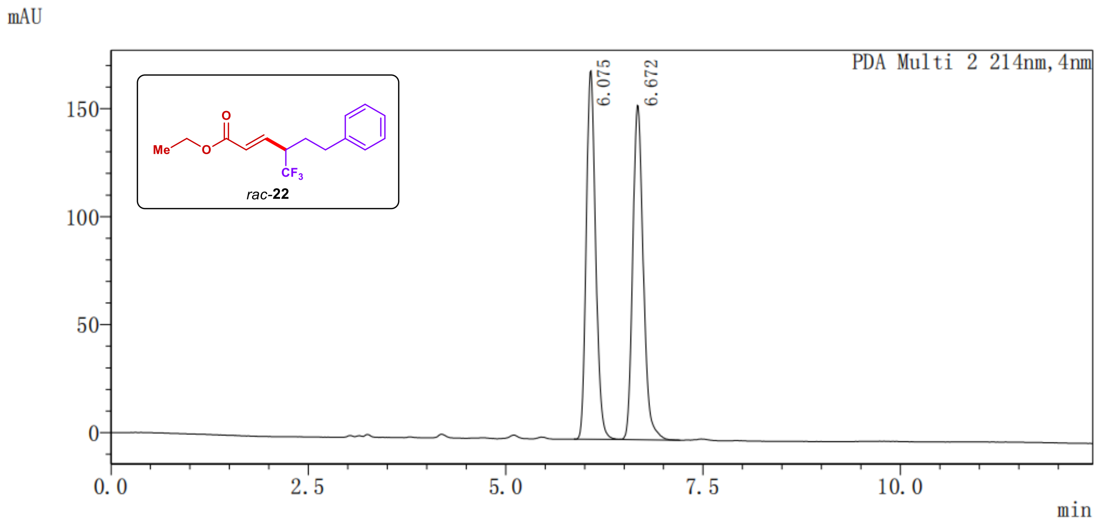

Peak#	Ret. Time	Area	Area#
1	6.075	1415000	49.572
2	6.672	1439445	50.428
Total		2854445	100.000

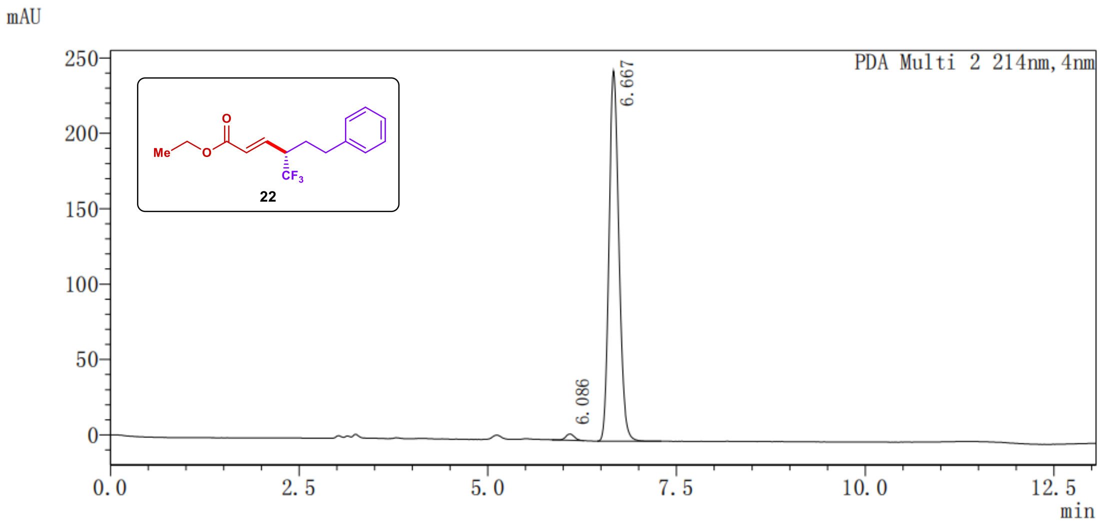

Peak#	Ret. Time	Area	Area#
1	6.086	32043	1.410
2	6.667	2239689	98.590
Total		2271731	100.000

Supplementary Figure 204. Chiral HPLC analysis of Compound **22**

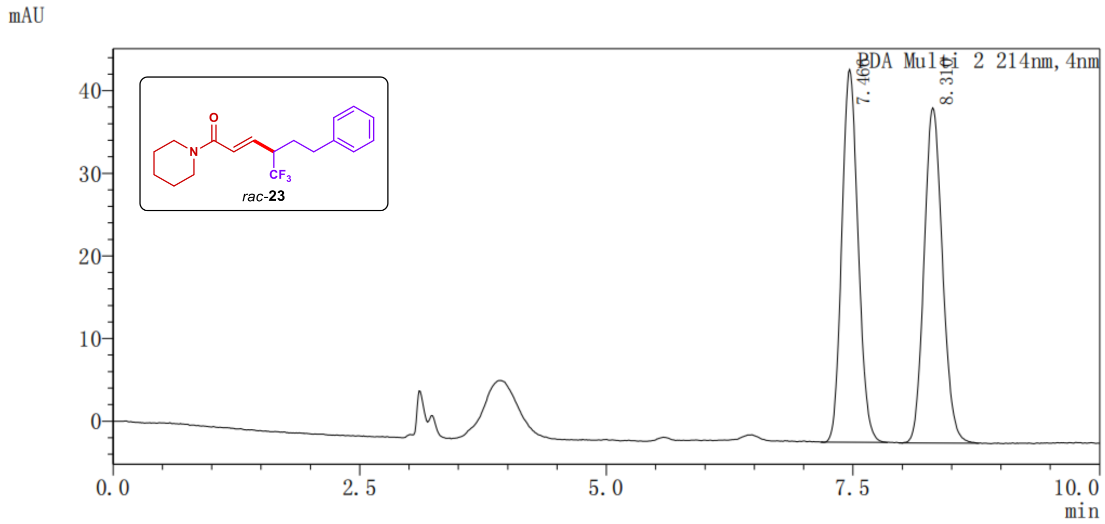

Peak#	Ret. Time	Area	Area#
1	7.466	518296	49.619
2	8.310	526258	50.381
Total		1044554	100.000

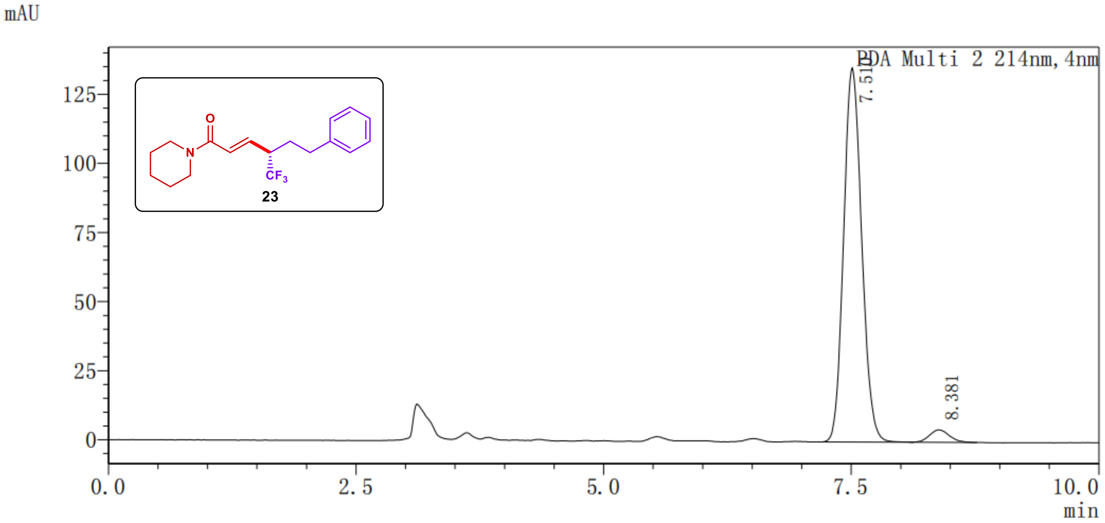

Peak#	Ret. Time	Area	Area#
1	7.510	1717366	96.414
2	8.381	63870	3.586
Total		1781236	100.000

Supplementary Figure 205. Chiral HPLC analysis of Compound **23**

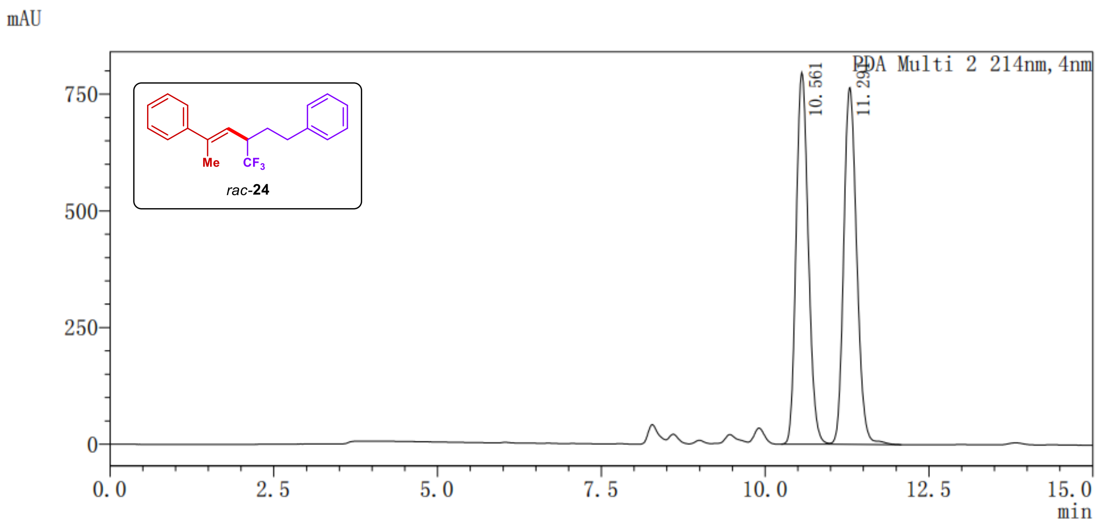

Peak#	Ret. Time	Area	Area#
1	10.561	10289828	49.346
2	11.291	10562687	50.654
Total		20852515	100.000

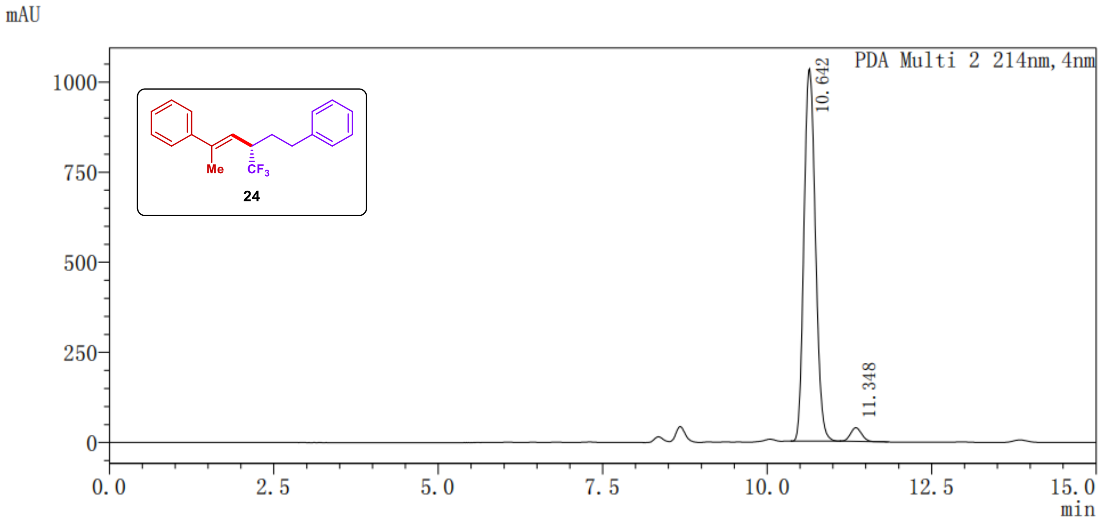

Peak#	Ret. Time	Area	Area#
1	10.642	12531071	96.547
2	11.348	448202	3.453
Total		12979273	100.000

Supplementary Figure 206. Chiral HPLC analysis of Compound **24**

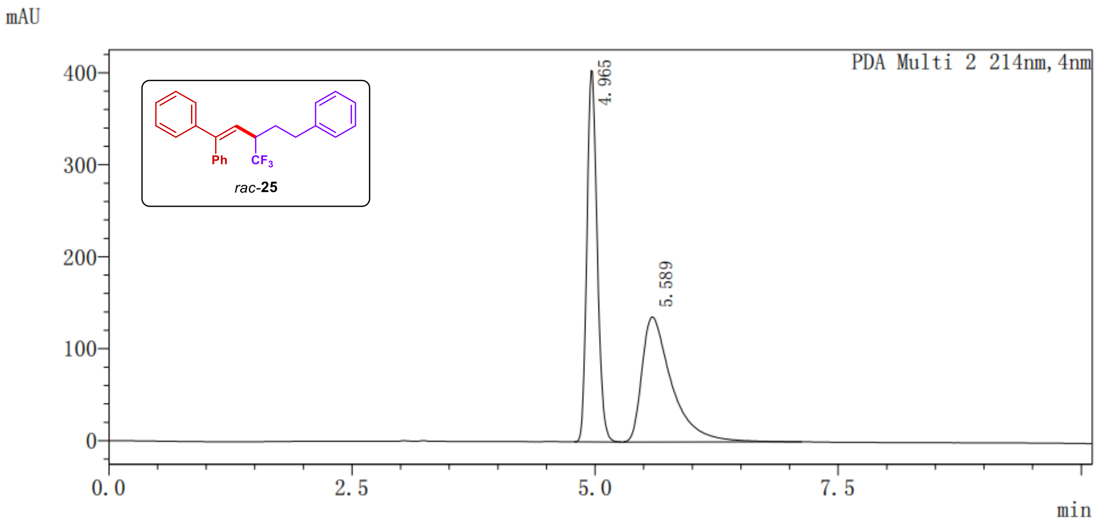

Peak#	Ret. Time	Area	Area#
1	4.965	2968081	50.535
2	5.589	2905217	49.465
Total		5873298	100.000

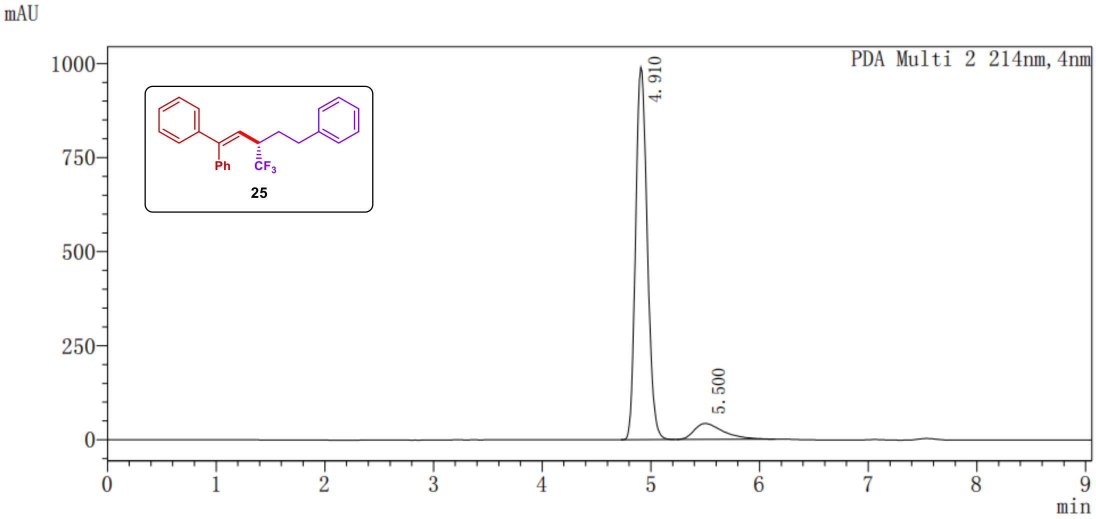

Peak#	Ret. Time	Area	Area#
1	4.910	7394148	90.319
2	5.500	792517	9.681
Total		8186665	100.000

Supplementary Figure 207. Chiral HPLC analysis of Compound **25**

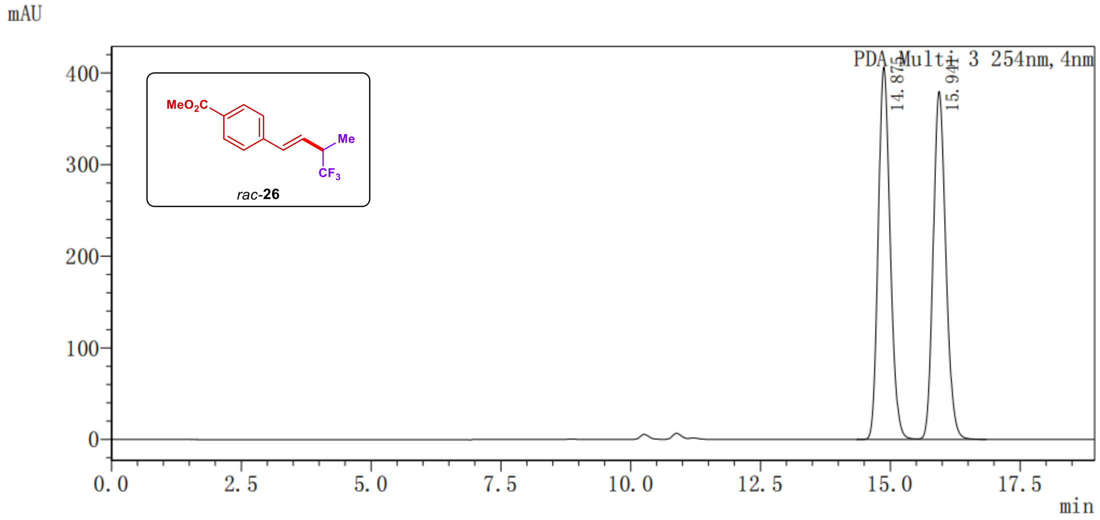

Peak#	Ret. Time	Area	Area#
1	14.875	6502124	50.253
2	15.941	6436708	49.747
Total		12938832	100.000

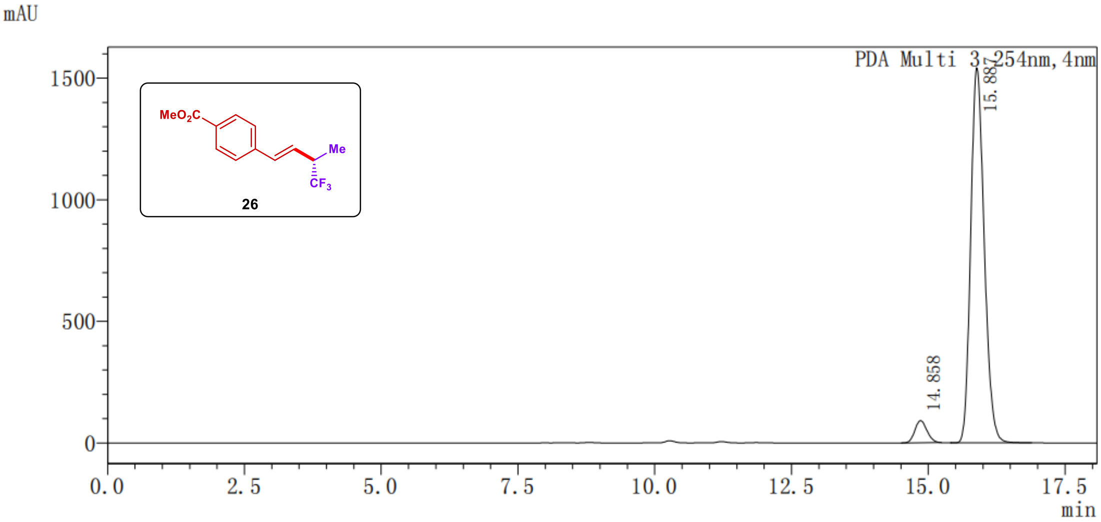

Peak#	Ret. Time	Area	Area#
1	14.858	1403644	5.034
2	15.887	26478207	94.966
Total		27881851	100.000

Supplementary Figure 208. Chiral HPLC analysis of Compound **26**

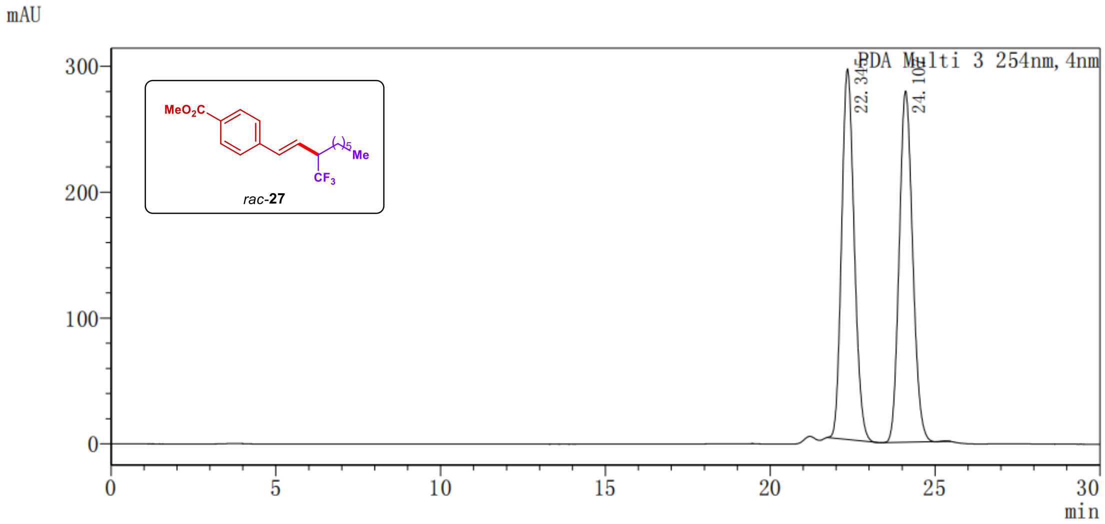

Peak#	Ret. Time	Area	Area#
1	22.345	7836214	49.667
2	24.107	7941404	50.333
Total		15777618	100.000

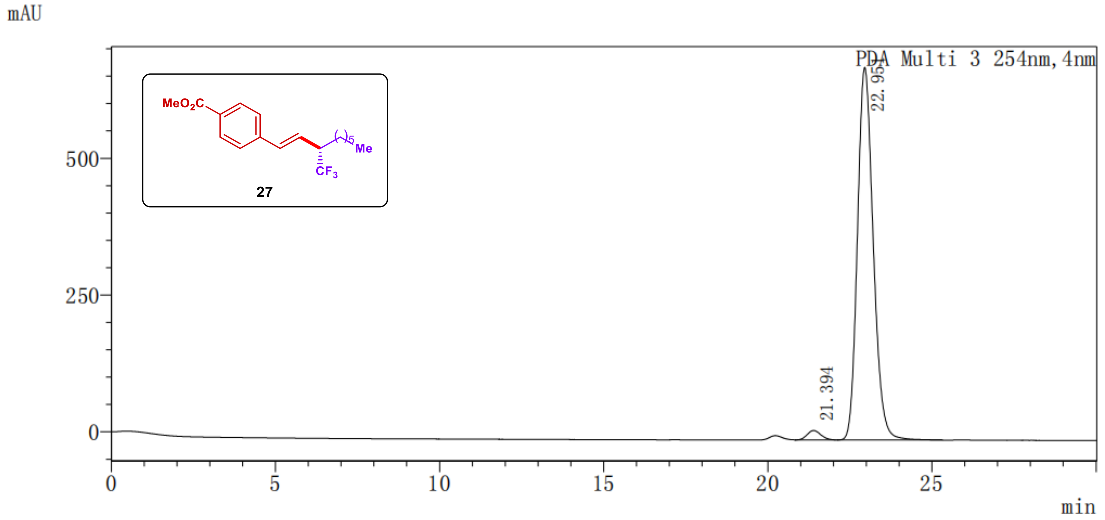

Peak#	Ret. Time	Area	Area#
1	21.394	489399	2.102
2	22.951	22795824	97.898
Total		23285223	100.000

Supplementary Figure 209. Chiral HPLC analysis of Compound **27**

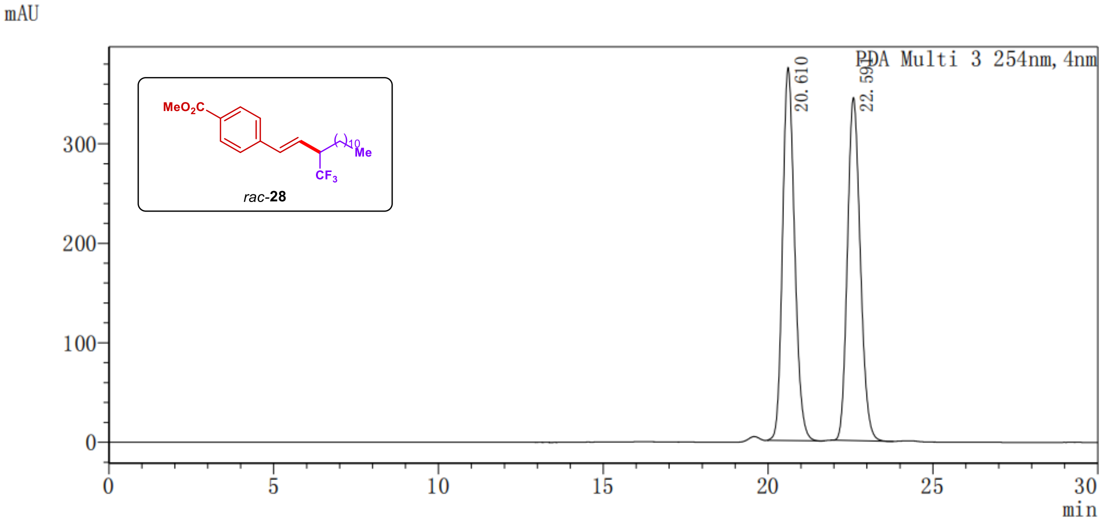

Peak#	Ret. Time	Area	Area#
1	20.610	9489315	50.324
2	22.591	9367093	49.676
Total		18856408	100.000

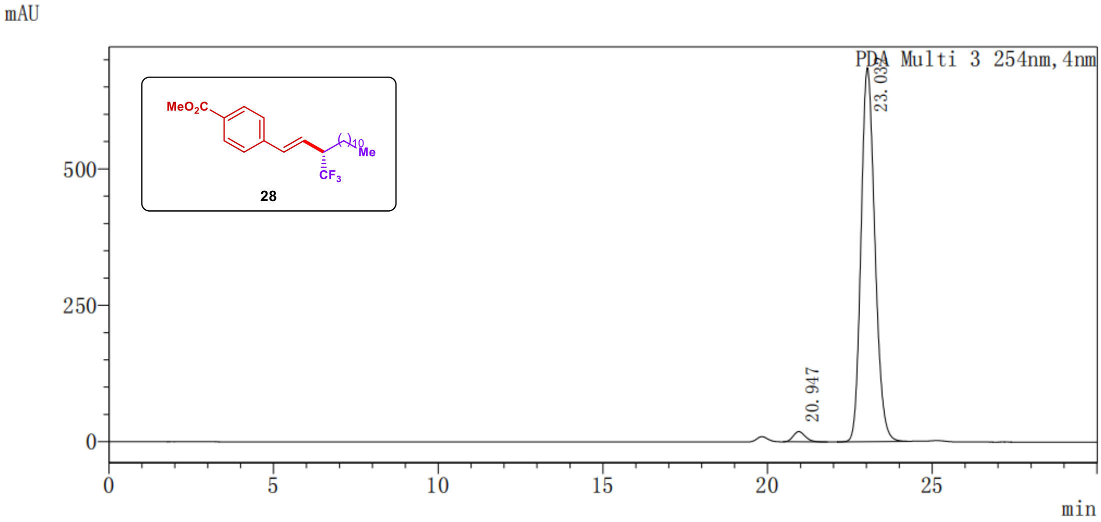

Peak#	Ret. Time	Area	Area#
1	20.947	476226	2.320
2	23.032	20050028	97.680
Total		20526254	100.000

Supplementary Figure 210. Chiral HPLC analysis of Compound **28**

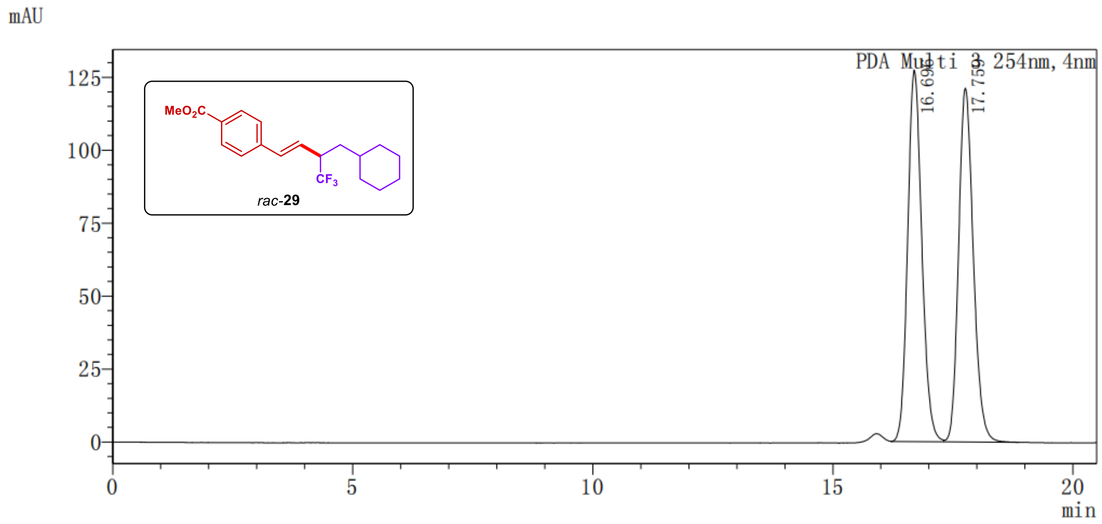

Peak#	Ret. Time	Area	Area#
1	16.696	2607833	50.257
2	17.759	2581145	49.743
Total		5188978	100.000

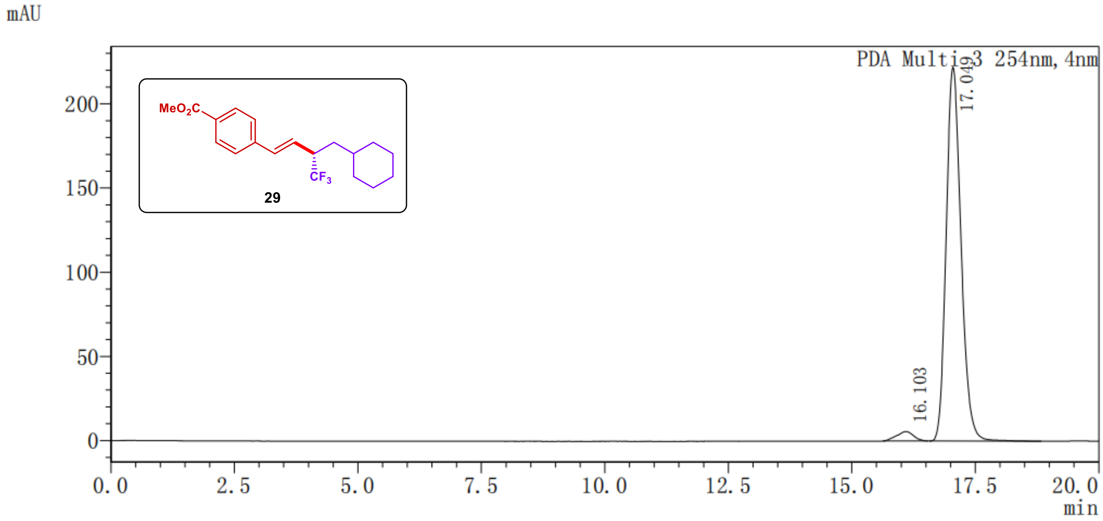

Peak#	Ret. Time	Area	Area#
1	16.103	132428	2.772
2	17.049	4644245	97.228
Total		4776673	100.000

Supplementary Figure 211. Chiral HPLC analysis of Compound **29**

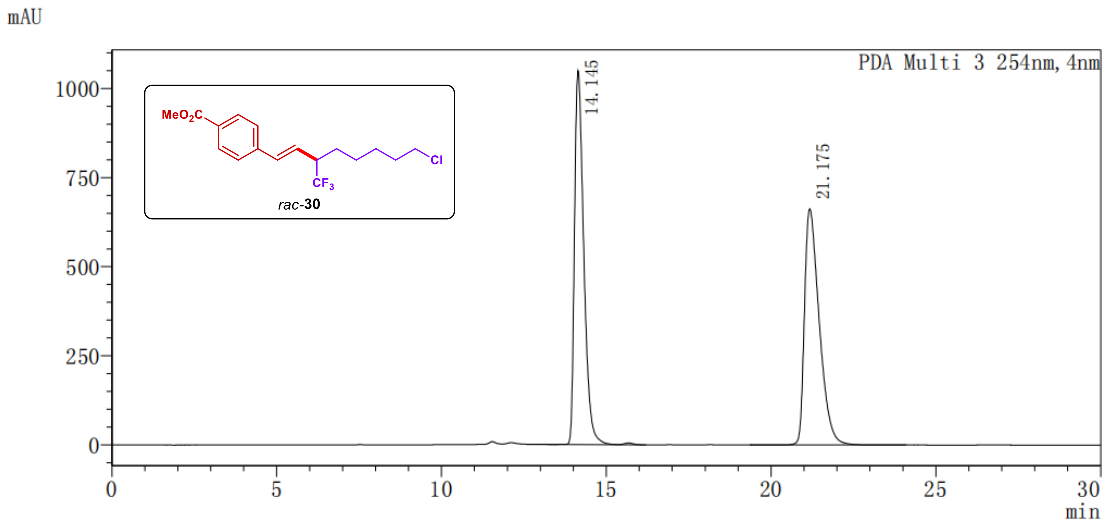

Peak#	Ret. Time	Area	Area#
1	14.145	20599755	49.931
2	21.175	20656662	50.069
Total		41256417	100.000

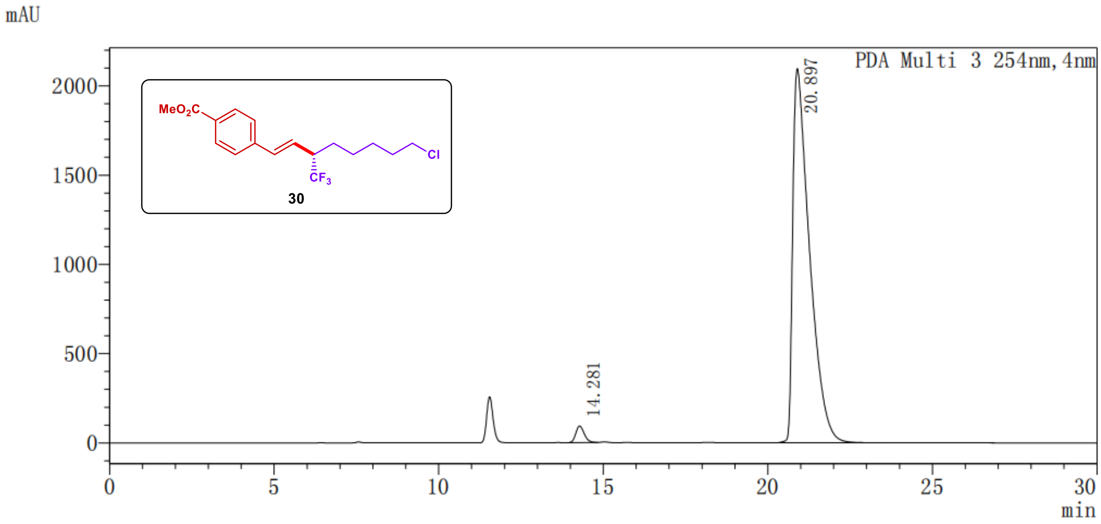

Peak#	Ret. Time	Area	Area#
1	14.281	1662489	2.187
2	20.897	74353451	97.813
Total		76015940	100.000

Supplementary Figure 212. Chiral HPLC analysis of Compound **30**

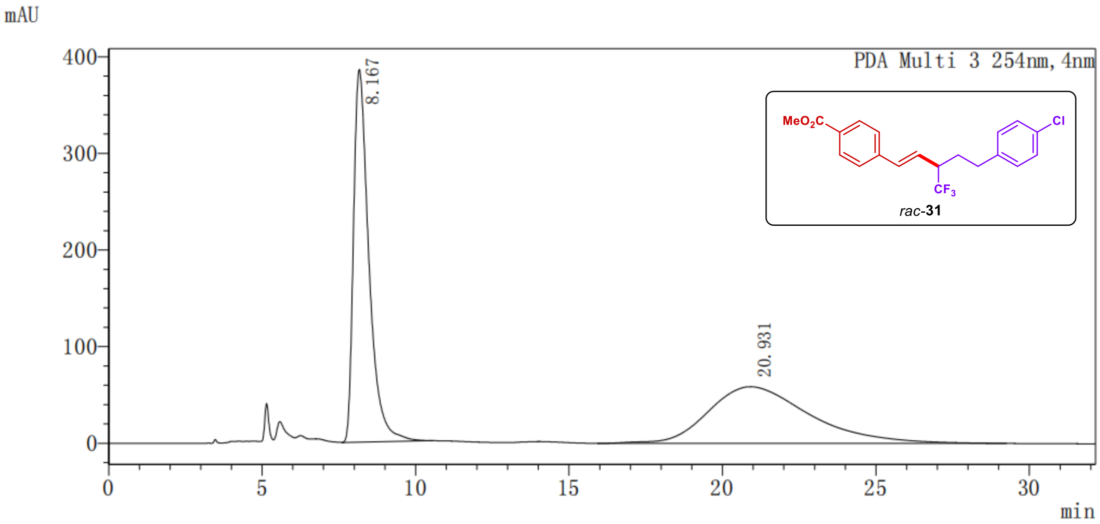

Peak#	Ret. Time	Area	Area#
1	8.167	13485600	49.921
2	20.931	13528529	50.079
Total		27014129	100.000

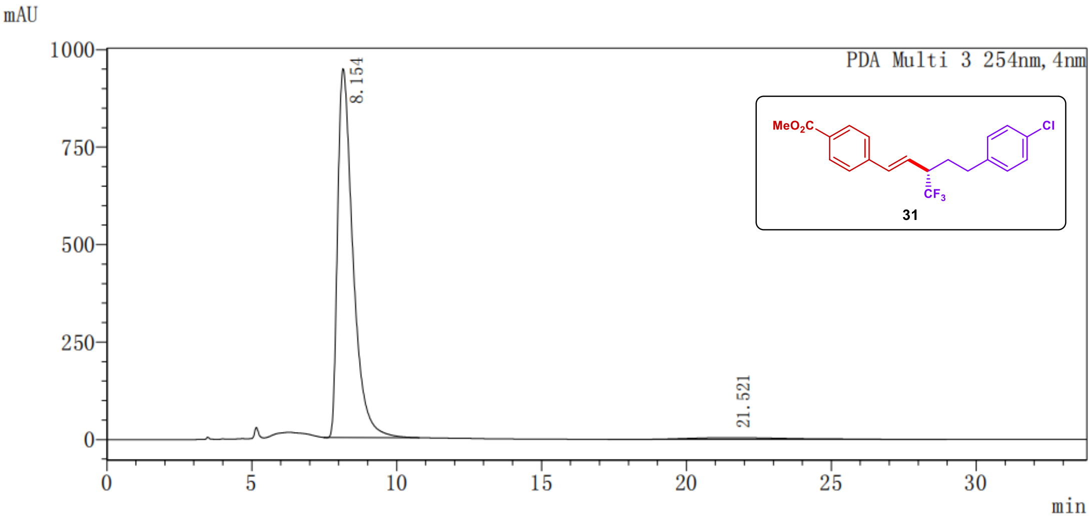

Peak#	Ret. Time	Area	Area#
1	8.154	34830380	96.851
2	21.521	1132354	3.149
Total		35962735	100.000

Supplementary Figure 213. Chiral HPLC analysis of Compound **31**

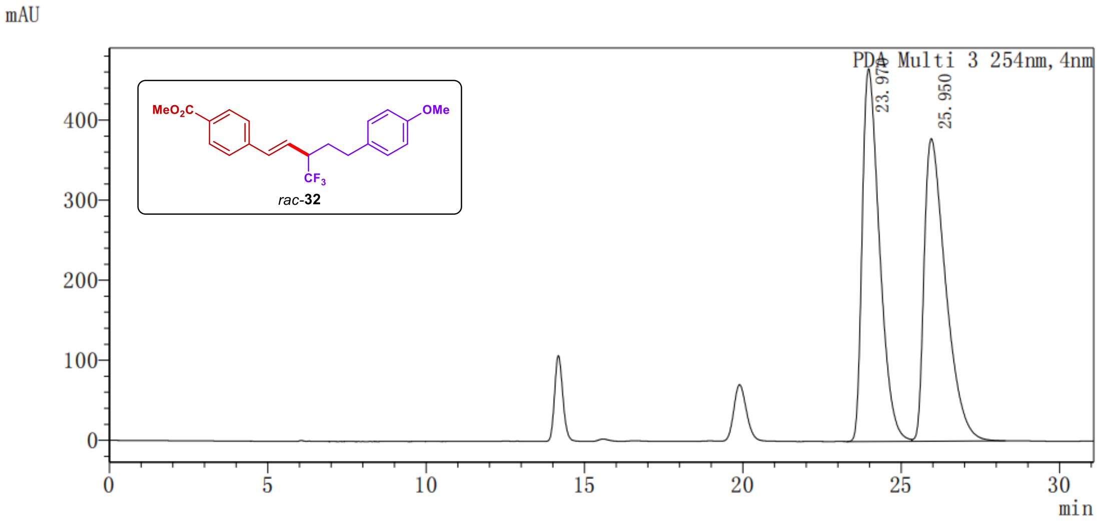

Peak#	Ret. Time	Area	Area#
1	23.970	17666673	49.675
2	25.950	17897874	50.325
Total		35564548	100.000

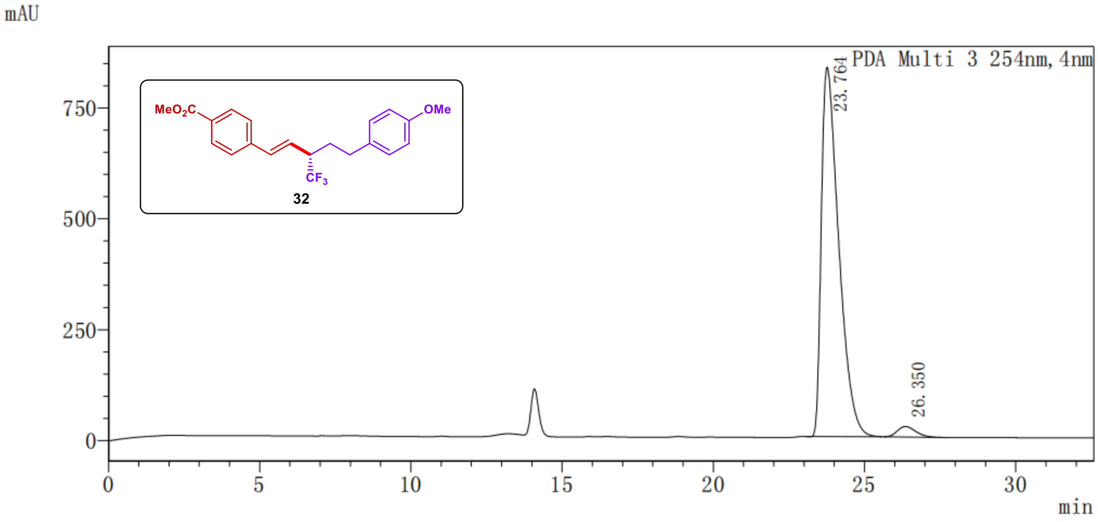

Peak#	Ret. Time	Area	Area#
1	23.764	33326410	97.074
2	26.350	1004644	2.926
Total		34331053	100.000

Supplementary Figure 214. Chiral HPLC analysis of Compound **32**

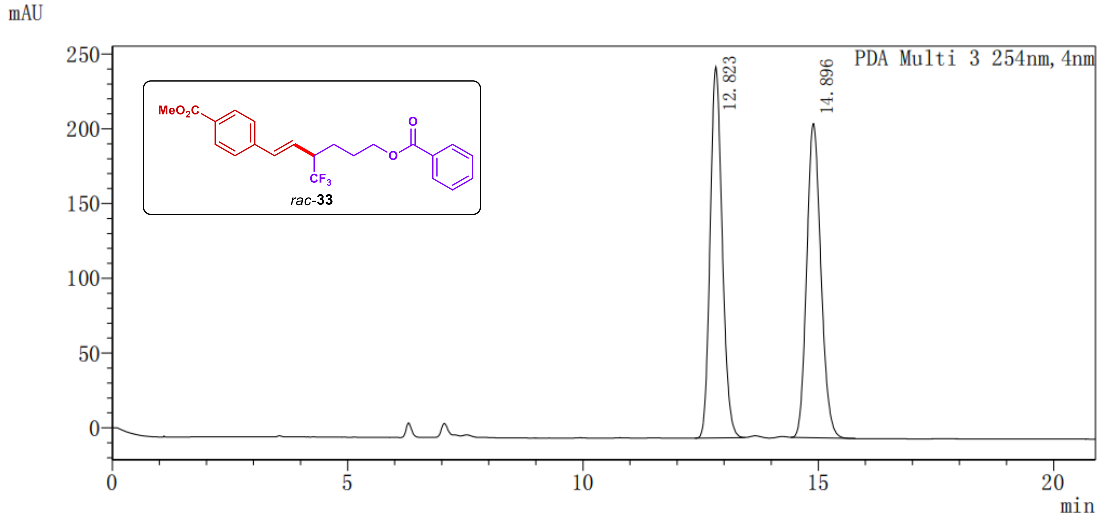

Peak#	Ret. Time	Area	Area#
1	12.823	4417416	50.024
2	14.896	4413211	49.976
Total		8830627	100.000

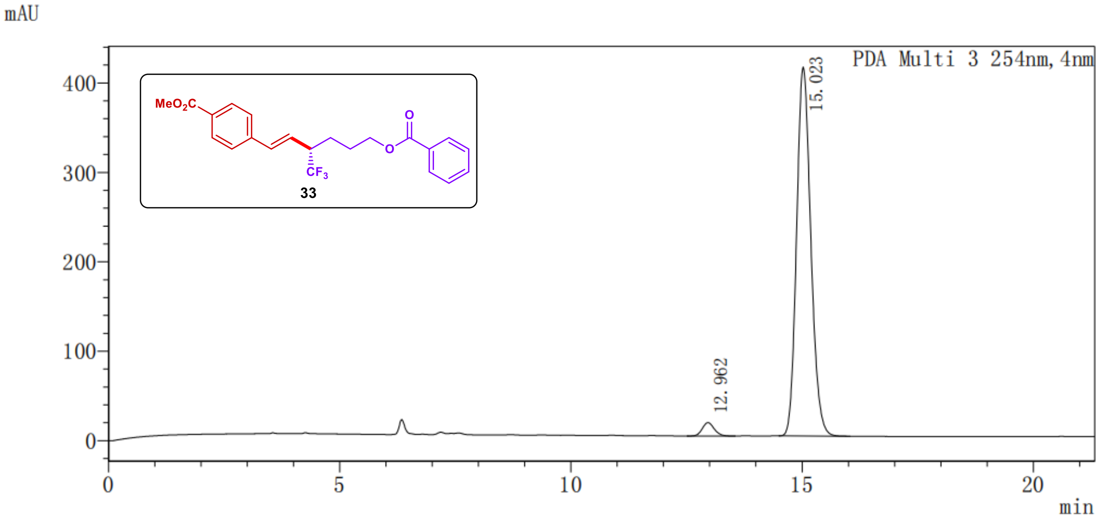

Peak#	Ret. Time	Area	Area#
1	12.962	269612	2.971
2	15.023	8804494	97.029
Total		9074106	100.000

Supplementary Figure 215. Chiral HPLC analysis of Compound **33**

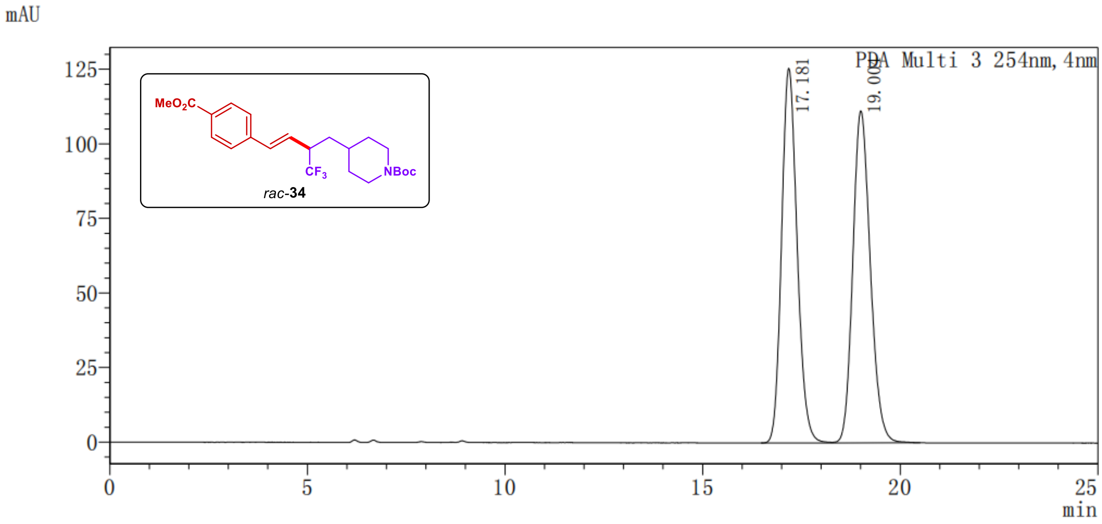

Peak#	Ret. Time	Area	Area#
1	17.181	3423703	49.964
2	19.004	3428602	50.036
Total		6852305	100.000

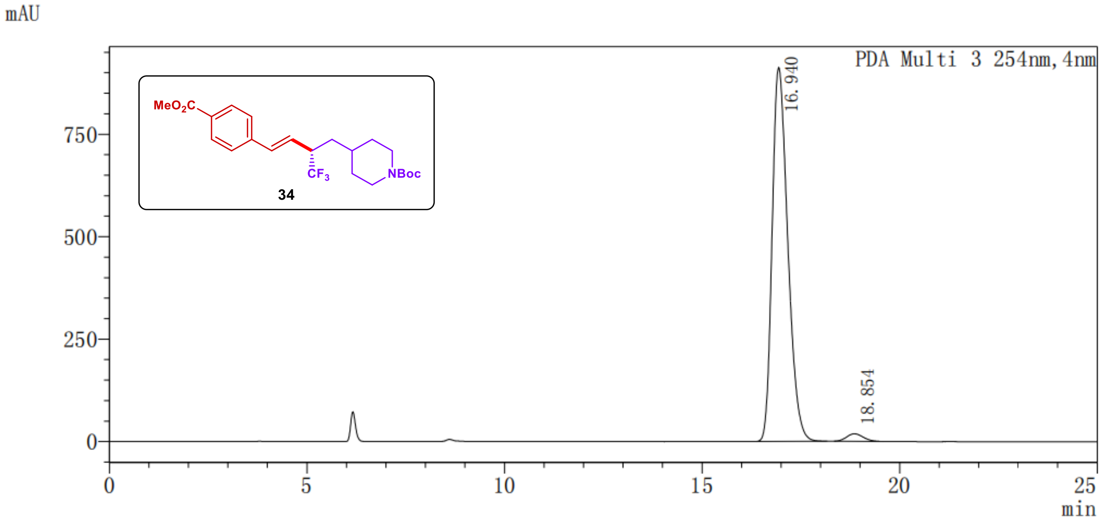

Peak#	Ret. Time	Area	Area#
1	16.940	25104090	97.883
2	18.854	543044	2.117
Total		25647135	100.000

Supplementary Figure 216. Chiral HPLC analysis of Compound **34**

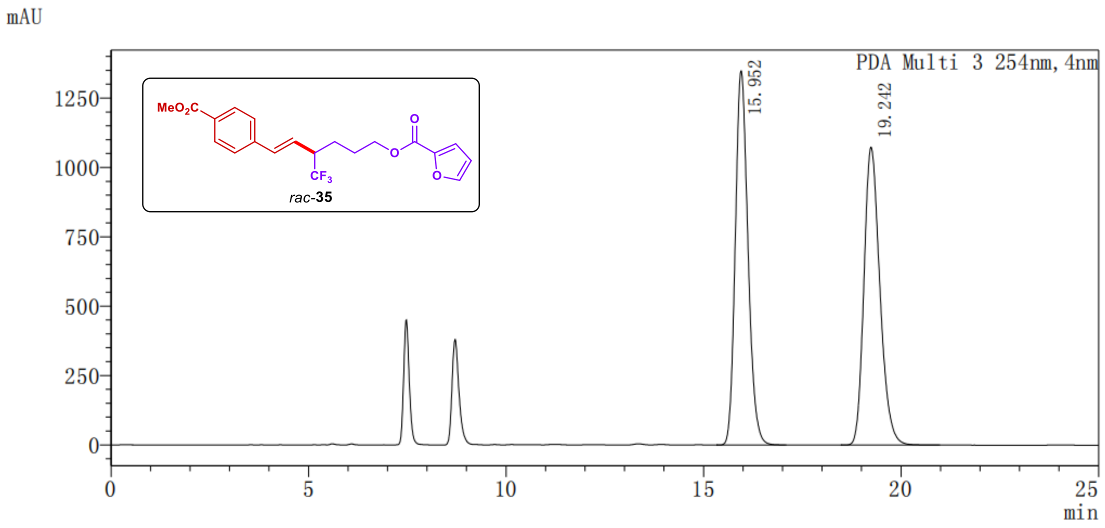

Peak#	Ret. Time	Area	Area#
1	15.952	30106112	49.941
2	19.242	30176807	50.059
Total		60282919	100.000

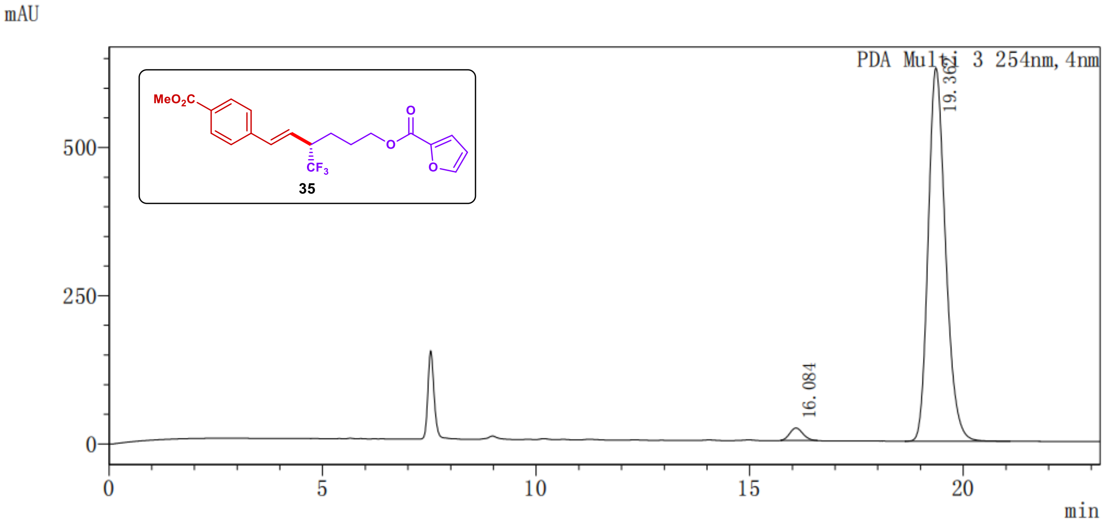

Peak#	Ret. Time	Area	Area#
1	16.084	448656	2.465
2	19.362	17755048	97.535
Total		18203704	100.000

Supplementary Figure 217. Chiral HPLC analysis of Compound **35**

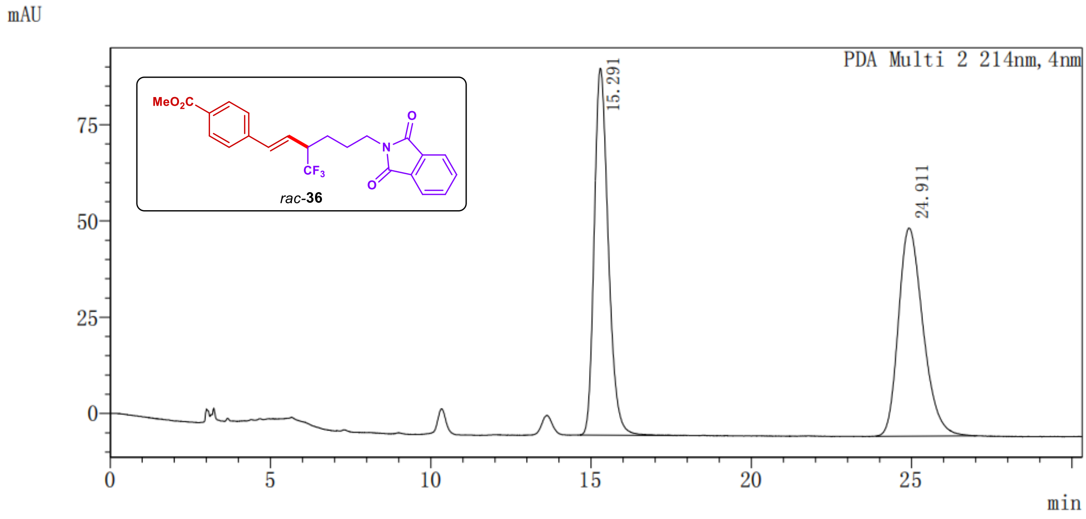

Peak#	Ret. Time	Area	Area#
1	15.291	2907792	50.180
2	24.911	2886910	49.820
Total		5794702	100.000

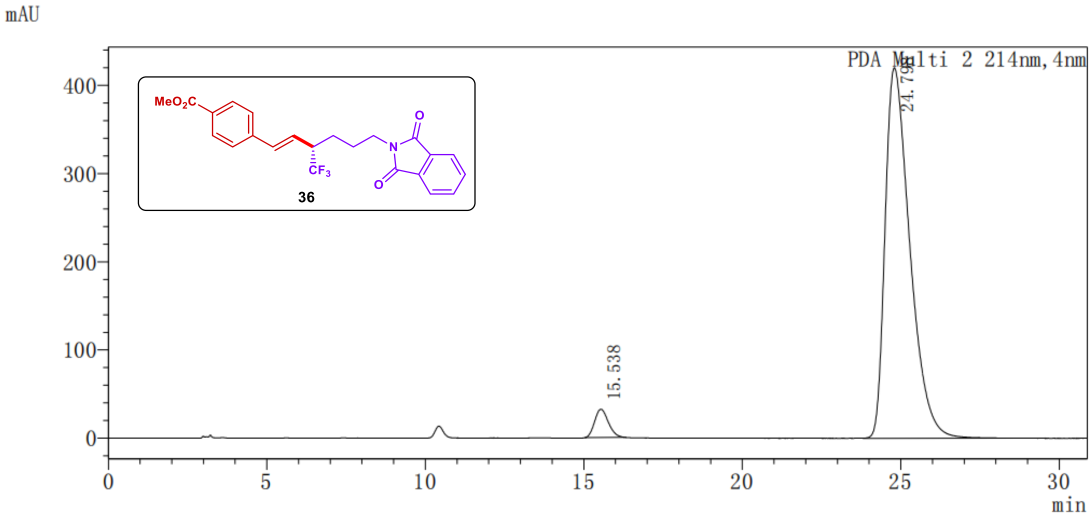

Peak#	Ret. Time	Area	Area#
1	15.538	964868	4.062
2	24.798	22788125	95.938
Total		23752993	100.000

Supplementary Figure 218. Chiral HPLC analysis of Compound **36**

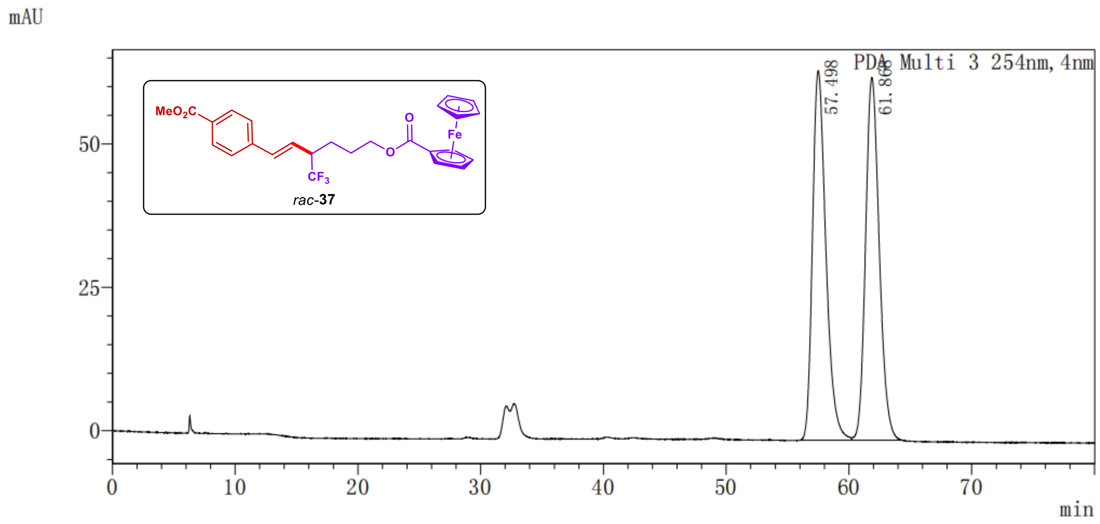

Peak#	Ret. Time	Area	Area#
1	57.498	4881519	49.895
2	61.868	4902062	50.105
Total		9783581	100.000

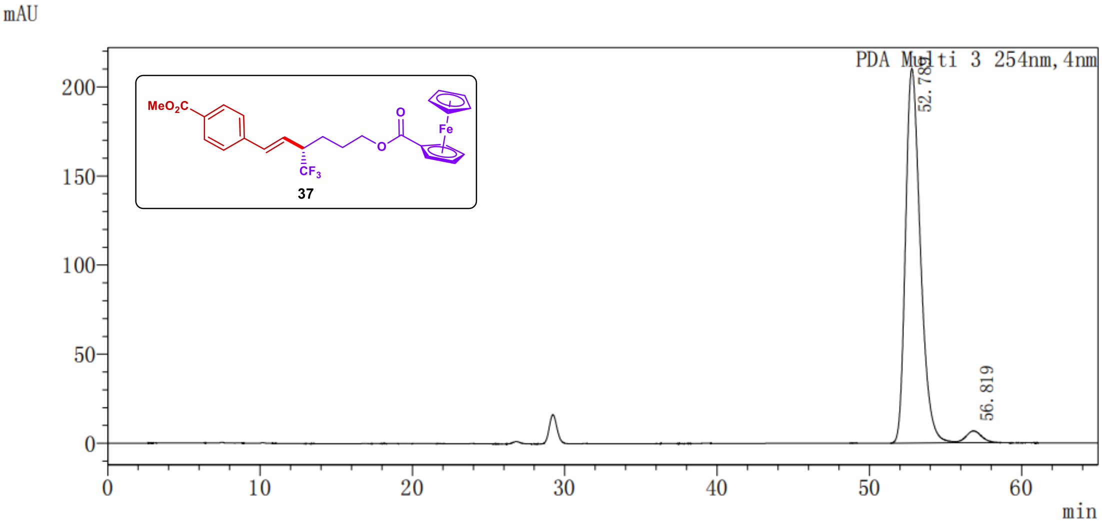

Peak#	Ret. Time	Area	Area#
1	52.789	14230159	96.752
2	56.819	477739	3.248
Total		14707898	100.000

Supplementary Figure 219. Chiral HPLC analysis of Compound **37**

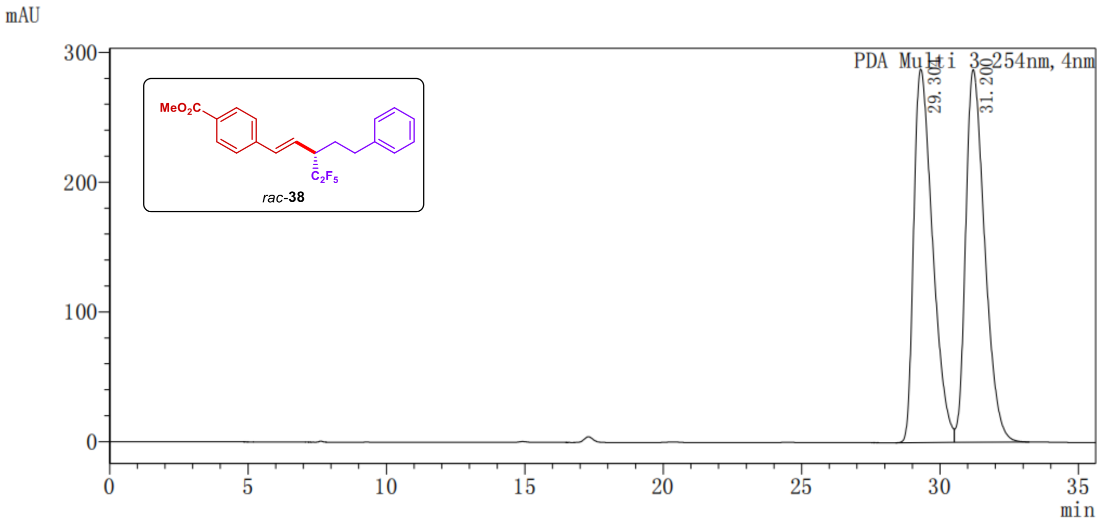

Peak#	Ret. Time	Area	Area#
1	29.304	13636320	49.757
2	31.200	13769469	50.243
Total		27405788	100.000

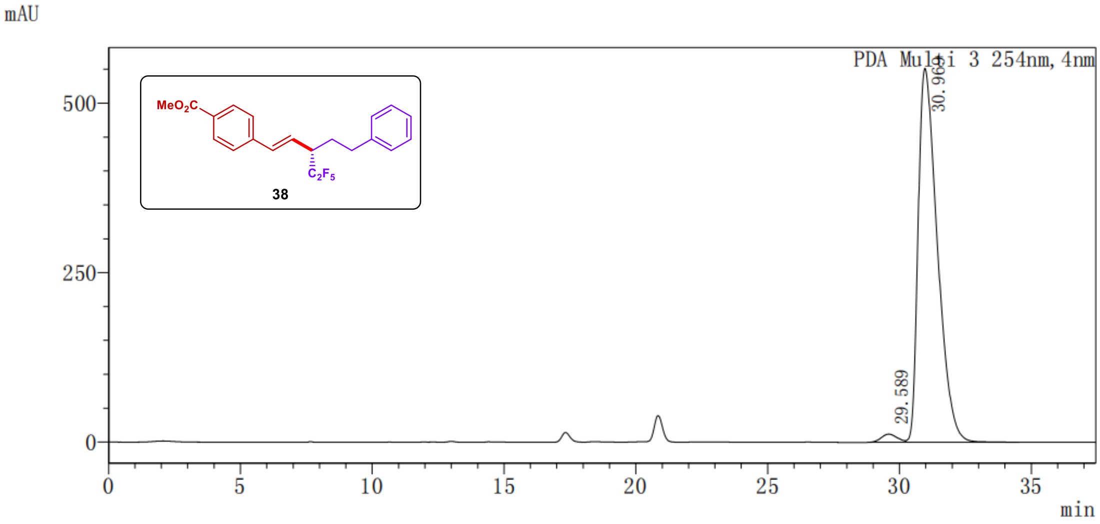

Peak#	Ret. Time	Area	Area#
1	29.589	513078	1.805
2	30.969	27906896	98.195
Total		28419974	100.000

Supplementary Figure 220. Chiral HPLC analysis of Compound **38**

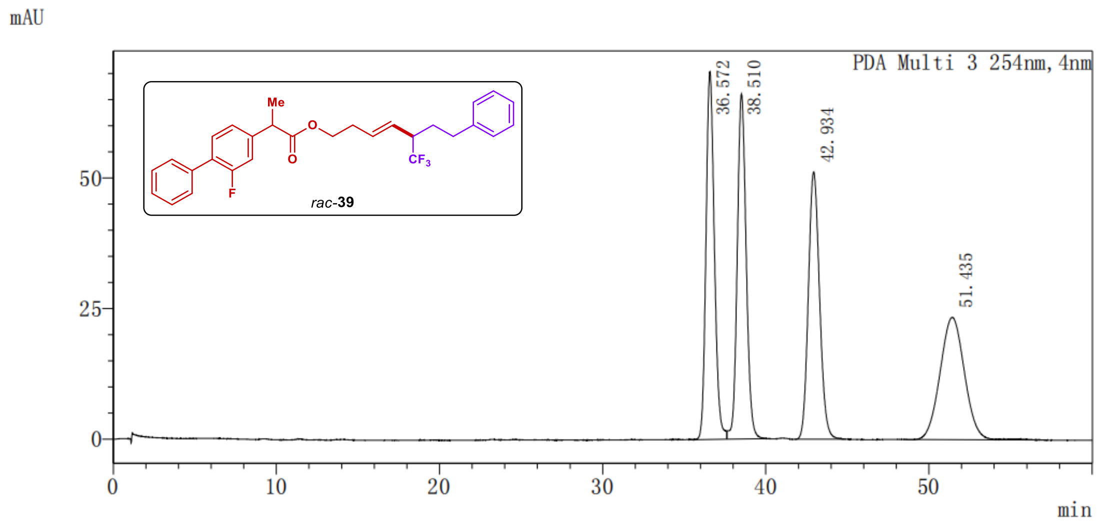

Peak#	Ret. Time	Area	Area#
1	36.572	2511665	25.371
2	38.510	2512981	25.385
3	42.934	2436857	24.616
4	51.435	2438153	24.629
Total		9899655	100.000

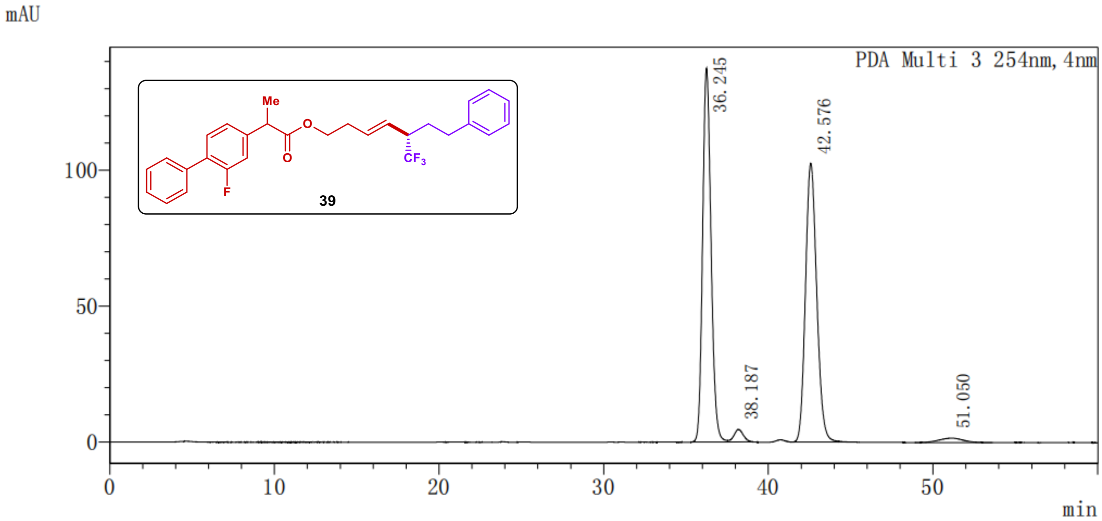

Peak#	Ret. Time	Area	Area#
1	36.245	4877380	48.291
2	38.187	181739	1.799
3	42.576	4879868	48.316
4	51.050	161013	1.594
Total		10100000	100.000

Supplementary Figure 221. Chiral HPLC analysis of Compound **39**

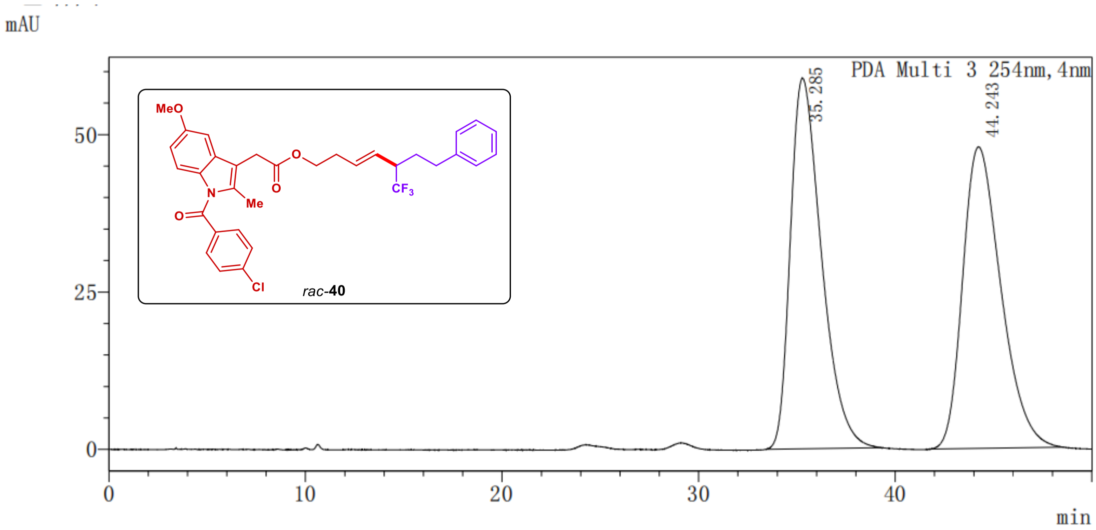

Peak#	Ret. Time	Area	Area#
1	35.285	6468992	49.950
2	44.243	6481827	50.050
Total		12950819	100.000

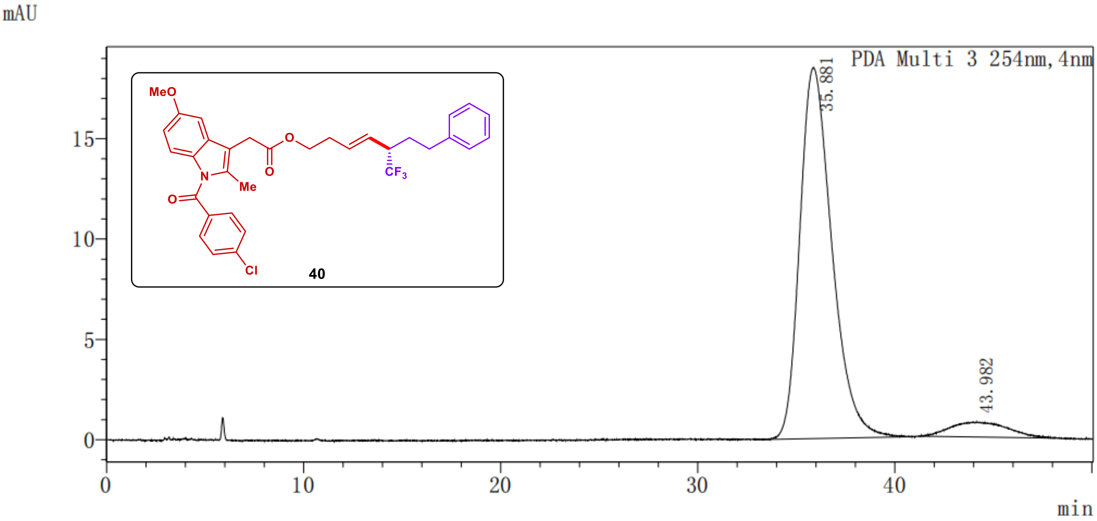

Peak#	Ret. Time	Area	Area#
1	35.881	2089502	93.218
2	43.982	152013	6.782
Total		2241515	100.000

Supplementary Figure 222. Chiral HPLC analysis of Compound **40**

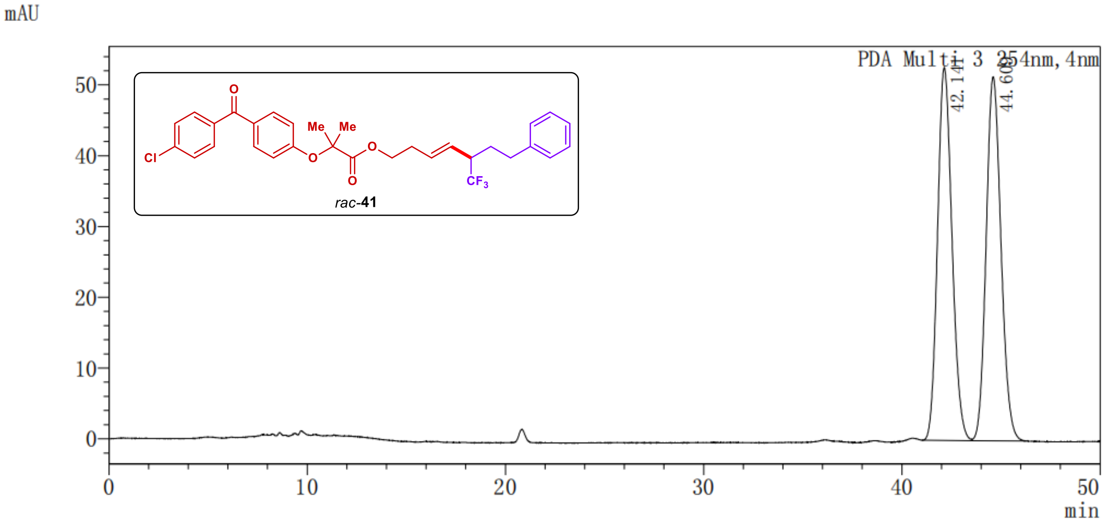

Peak#	Ret. Time	Area	Area#
1	42.141	2664704	49.886
2	44.609	2676866	50.114
Total		5341569	100.000

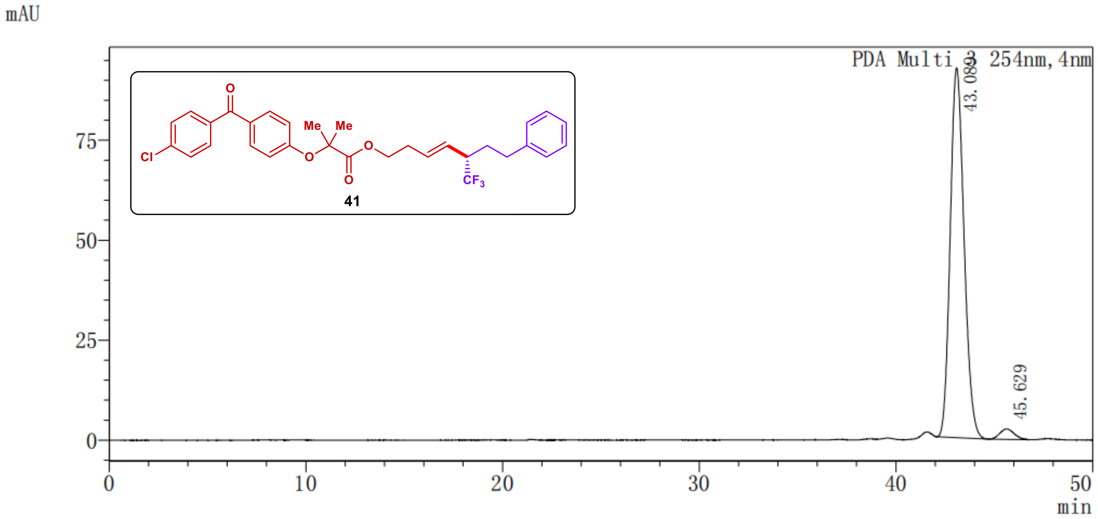

Peak#	Ret. Time	Area	Area#
1	43.089	4517879	97.127
2	45.629	133626	2.873
Total		4651505	100.000

Supplementary Figure 223. Chiral HPLC analysis of Compound **41**

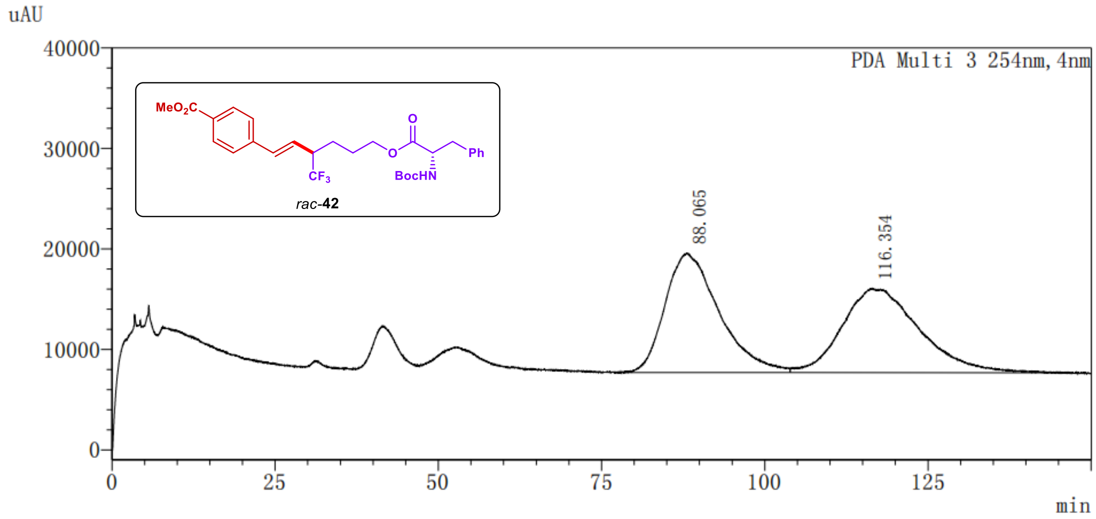

Peak#	Ret. Time	Area	Area#
1	88.065	6833138	49.804
2	116.354	6886839	50.196
Total		13719977	100.000

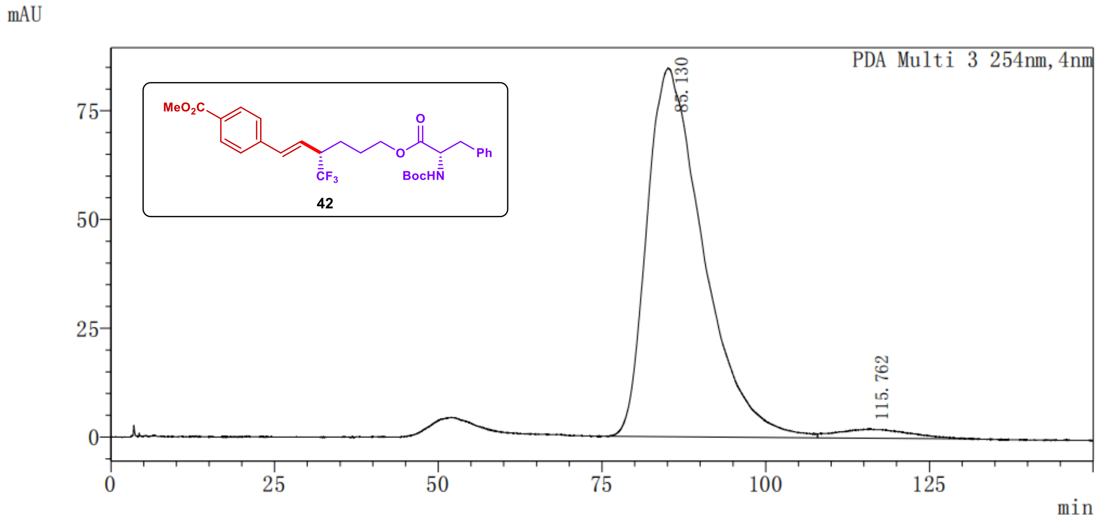

Peak#	Ret. Time	Area	Area#
1	85.130	50682323	96.914
2	115.762	1613770	3.086
Total		52296093	100.000

Supplementary Figure 224. Chiral HPLC analysis of Compound **42**

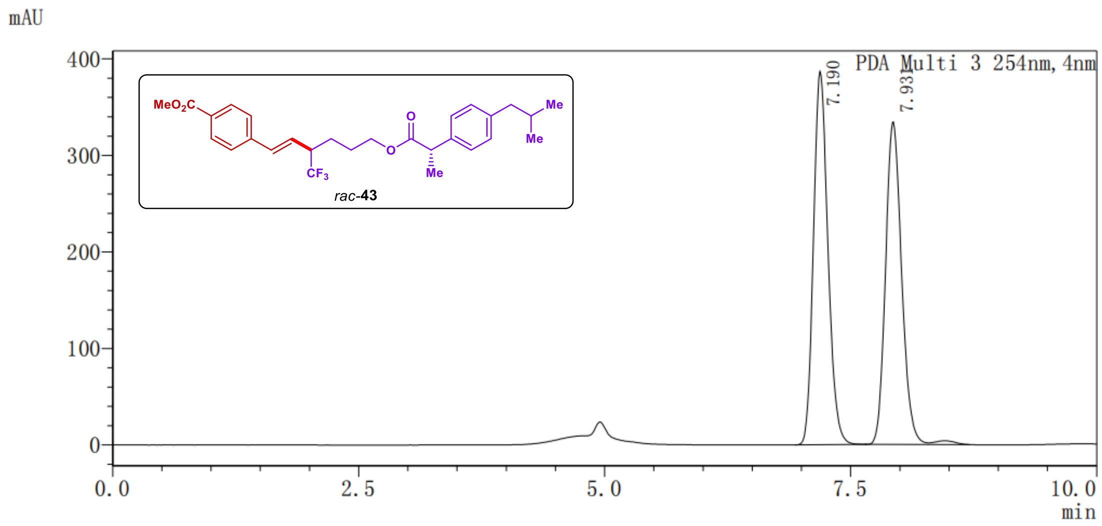

Peak#	Ret. Time	Area	Area#
1	7.190	3941262	50.623
2	7.931	3844190	49.377
Total		7785452	100.000

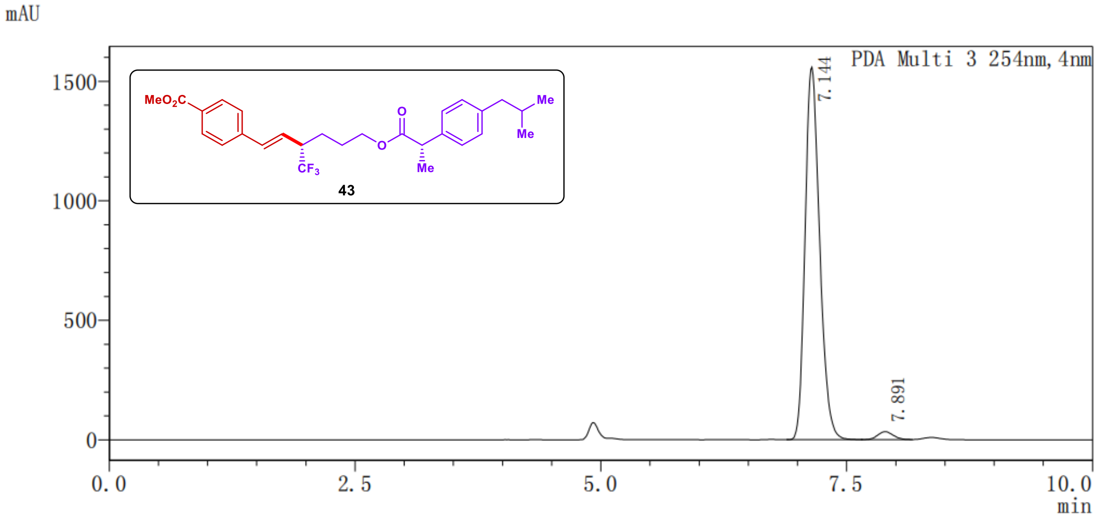

Peak#	Ret. Time	Area	Area#
1	7.144	16092136	97.795
2	7.891	362876	2.205
Total		16455012	100.000

Supplementary Figure 225. Chiral HPLC analysis of Compound **43**

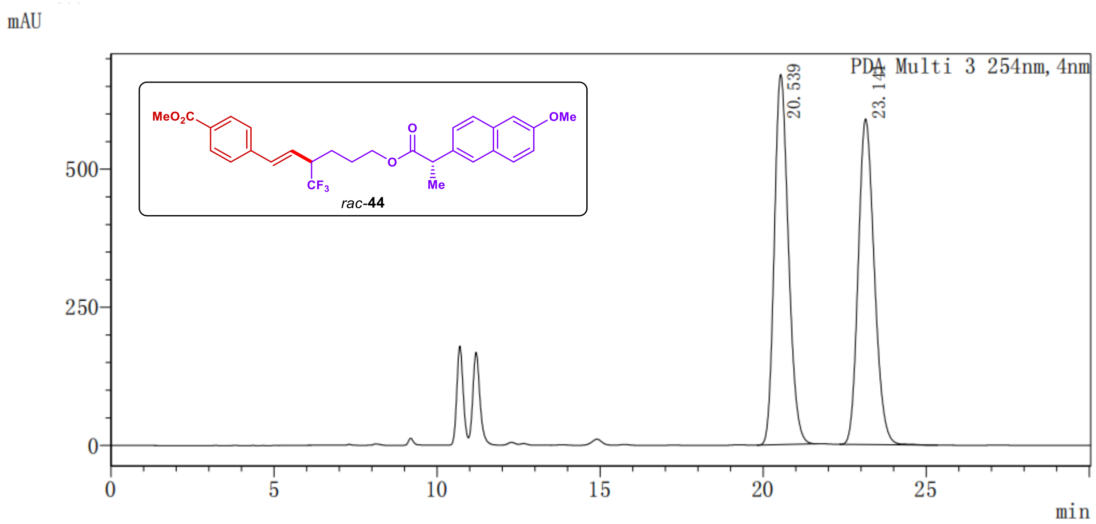

Peak#	Ret. Time	Area	Area#
1	20.539	20155238	49.942
2	23.141	20202068	50.058
Total		40357305	100.000

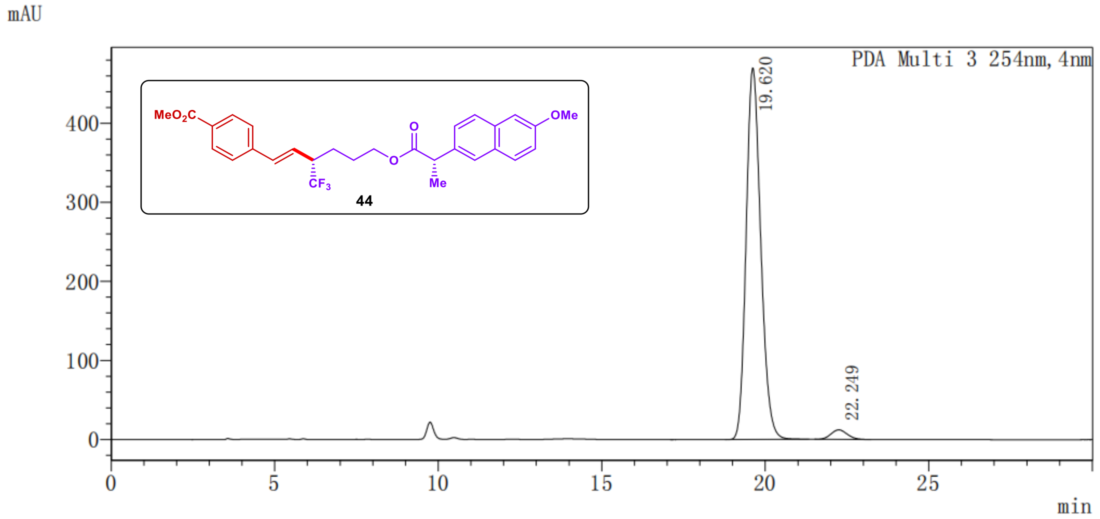

Peak#	Ret. Time	Area	Area#
1	19.620	14593824	97.163
2	22.249	426076	2.837
Total		15019900	100.000

Supplementary Figure 226. Chiral HPLC analysis of Compound **44**

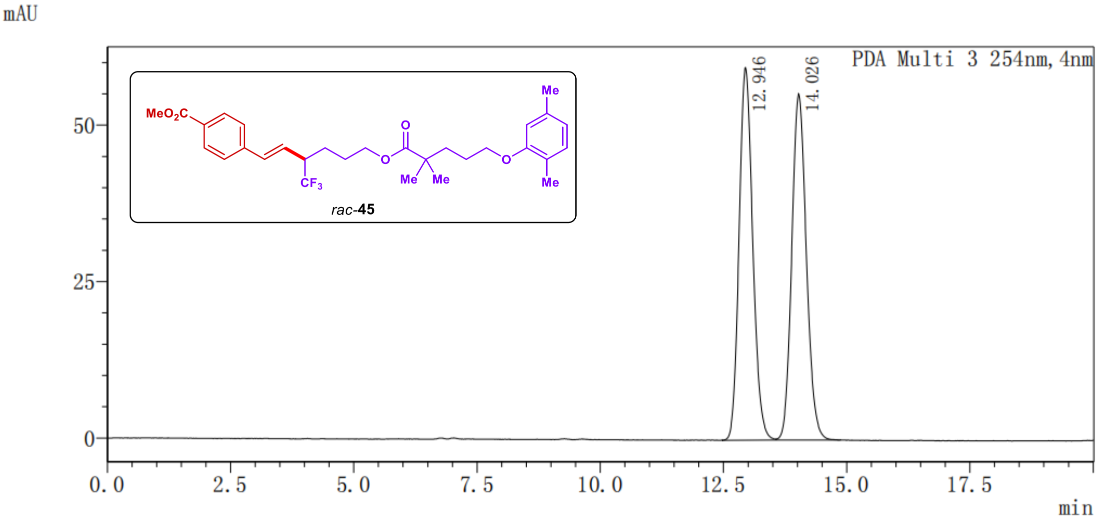

Peak#	Ret. Time	Area	Area#
1	12.946	1146158	50.170
2	14.026	1138392	49.830
Total		2284549	100.000

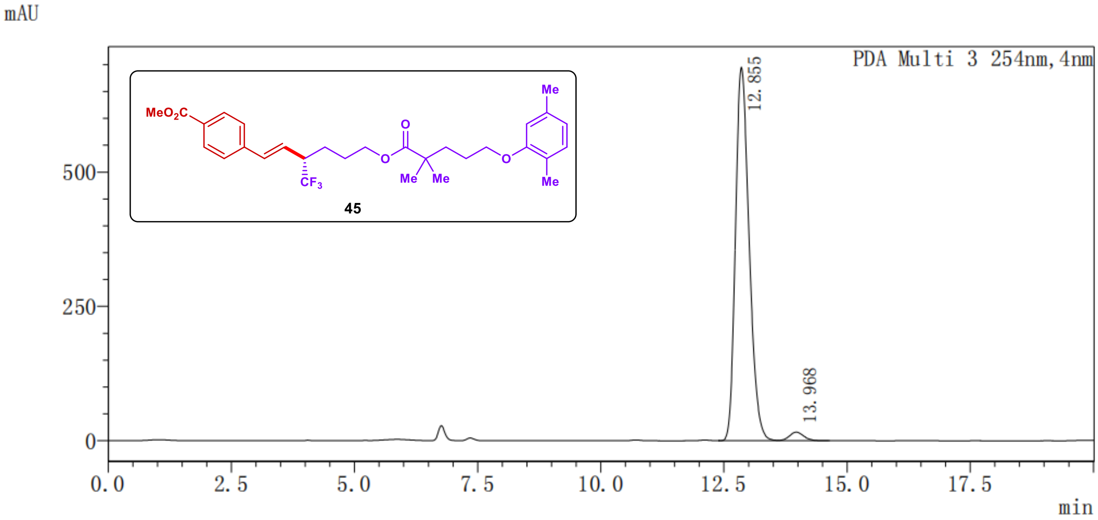

Peak#	Ret. Time	Area	Area#
1	12.855	13416427	97.620
2	13.968	327034	2.380
Total		13743462	100.000

Supplementary Figure 227. Chiral HPLC analysis of Compound **45**

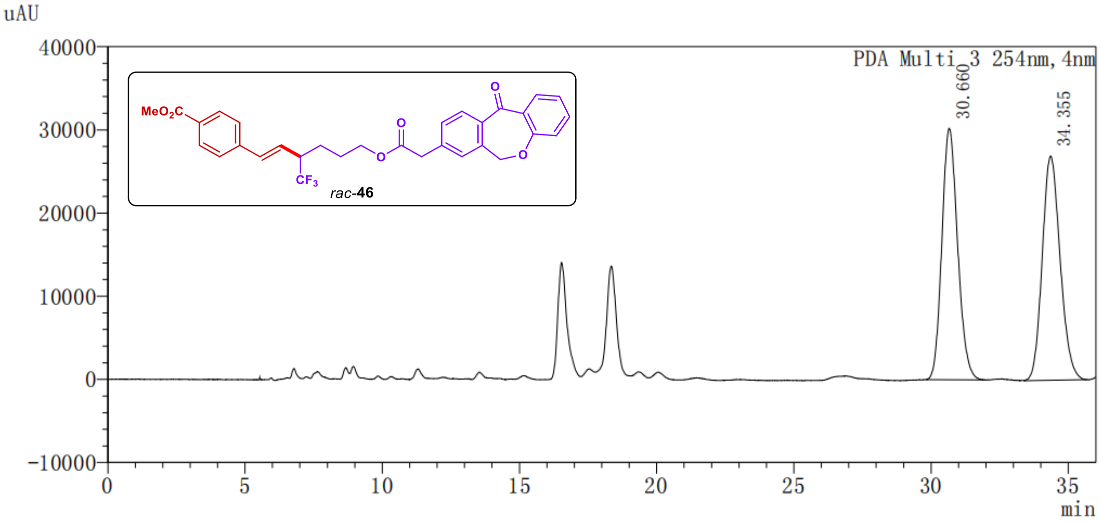

Peak#	Ret. Time	Area	Area#
1	30.660	1205913	49.655
2	34.355	1222656	50.345
Total		2428570	100.000

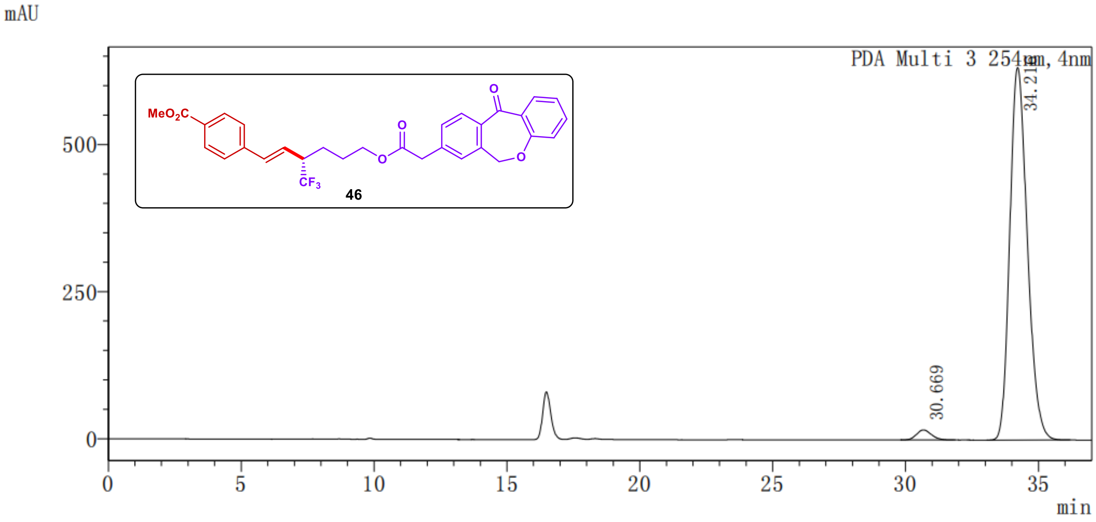

Peak#	Ret. Time	Area	Area#
1	30.669	670054	2.252
2	34.216	29084107	97.748
Total		29754160	100.000

Supplementary Figure 228. Chiral HPLC analysis of Compound **46**

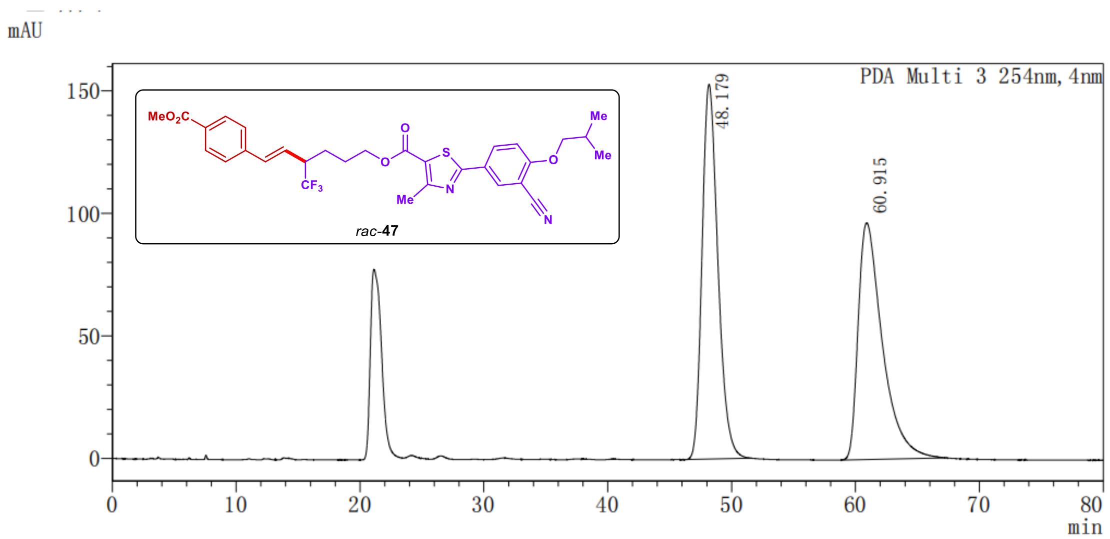

Peak#	Ret. Time	Area	Area#
1	48.179	13578449	50.819
2	60.915	13140645	49.181
Total		26719094	100.000

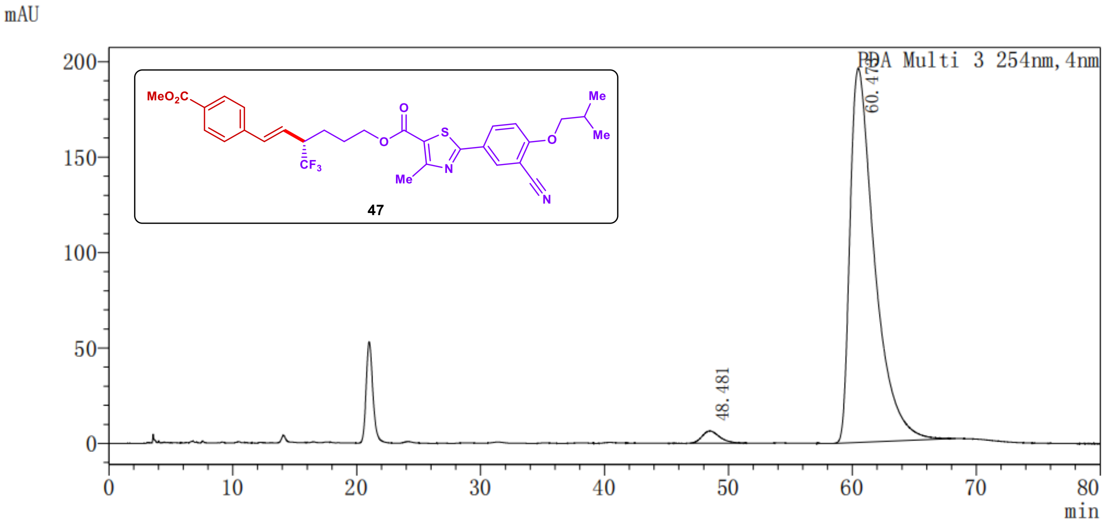

Peak#	Ret. Time	Area	Area#
1	48.481	623421	2.261
2	60.473	26953288	97.739
Total		27576709	100.000

Supplementary Figure 229. Chiral HPLC analysis of Compound **47**

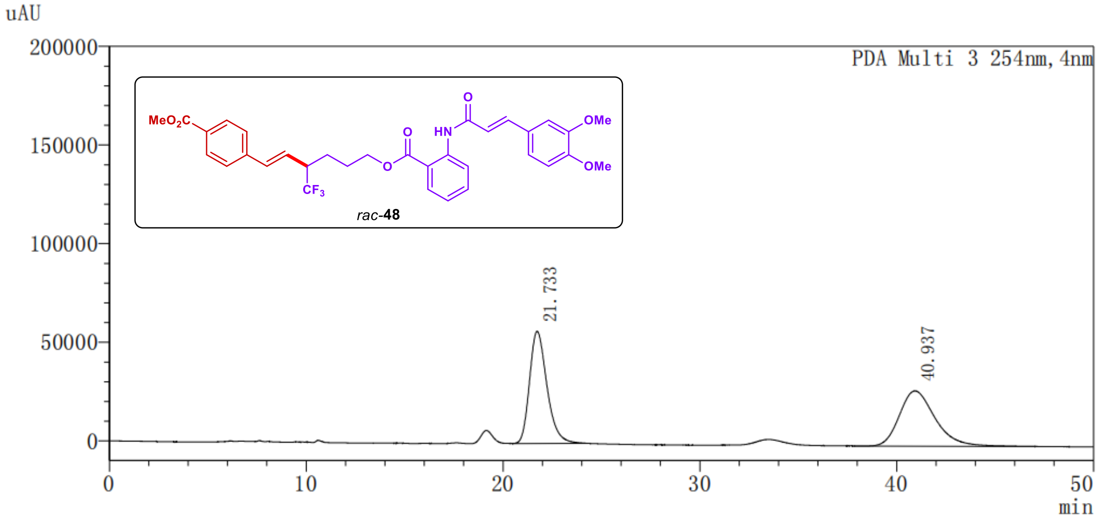

Peak#	Ret. Time	Area	Area#
1	21.733	3499419	49.679
2	40.937	3544595	50.321
Total		7044014	100.000

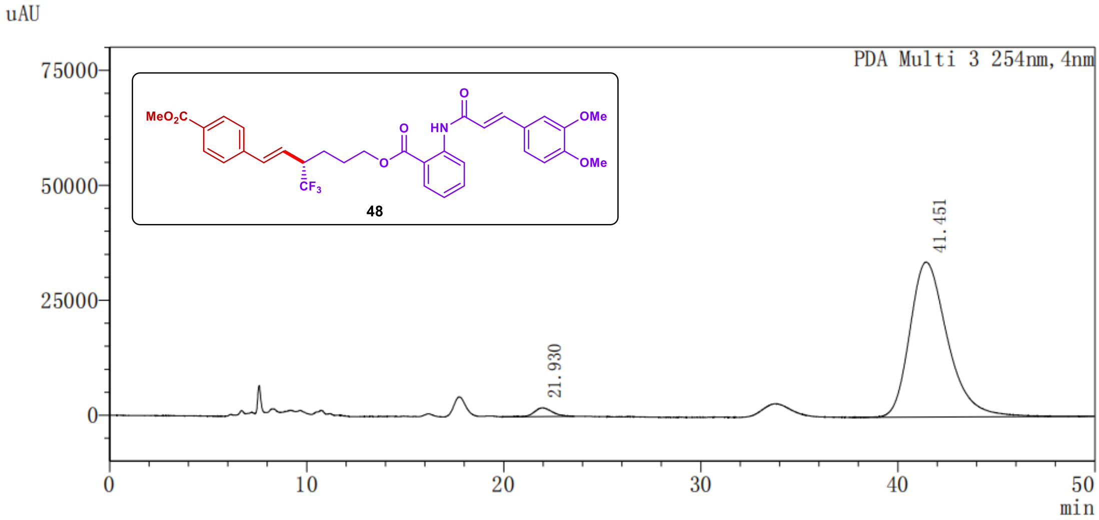

Peak#	Ret. Time	Area	Area#
1	21.930	117719	2.558
2	41.451	4483536	97.442
Total		4601254	100.000

Supplementary Figure 230. Chiral HPLC analysis of Compound **48**

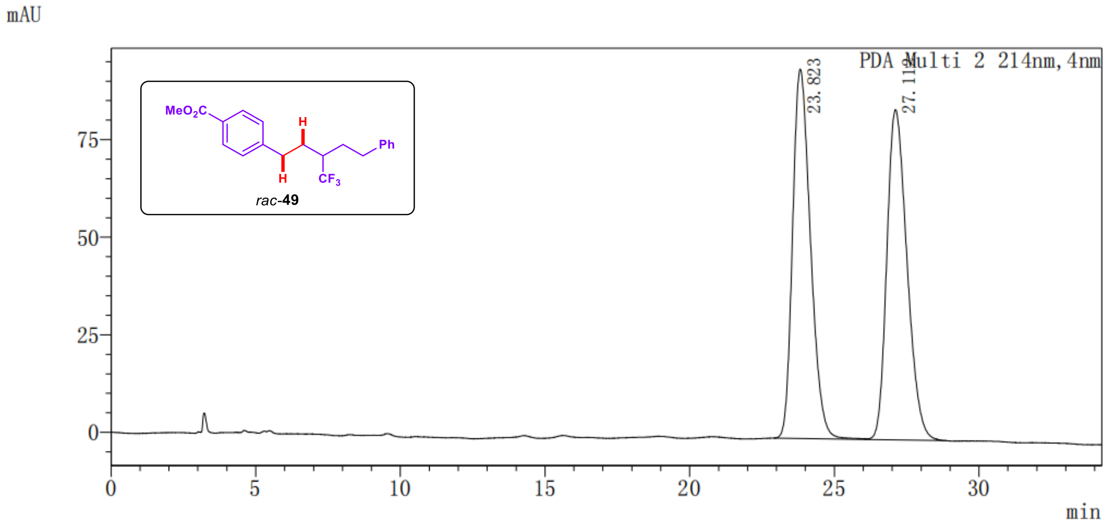

Peak#	Ret. Time	Area	Area#
1	23.823	4062203	49.783
2	27.112	4097661	50.217
Total		8159864	100.000

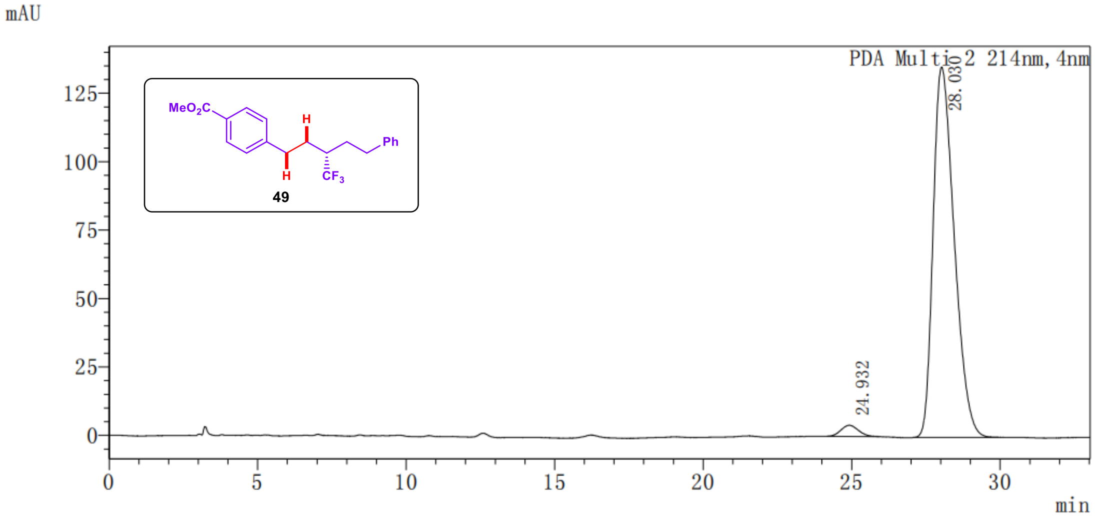

Peak#	Ret. Time	Area	Area#
1	24.932	170330	2.488
2	28.030	6675492	97.512
Total		6845822	100.000

Supplementary Figure 231. Chiral HPLC analysis of Compound **49**

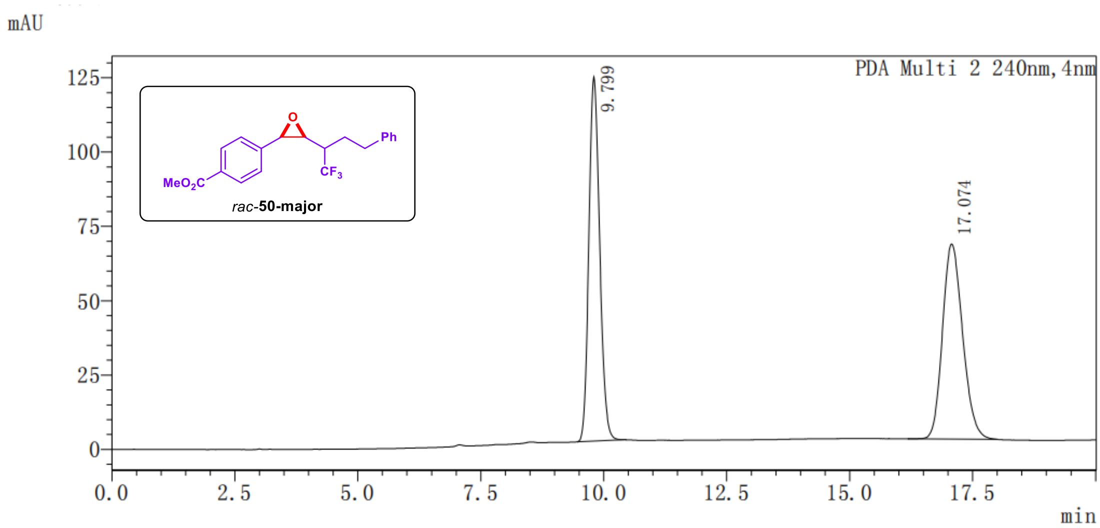

Peak#	Ret. Time	Area	Area#
1	9.799	1871588	50.052
2	17.074	1867687	49.948
Total		3739275	100.000

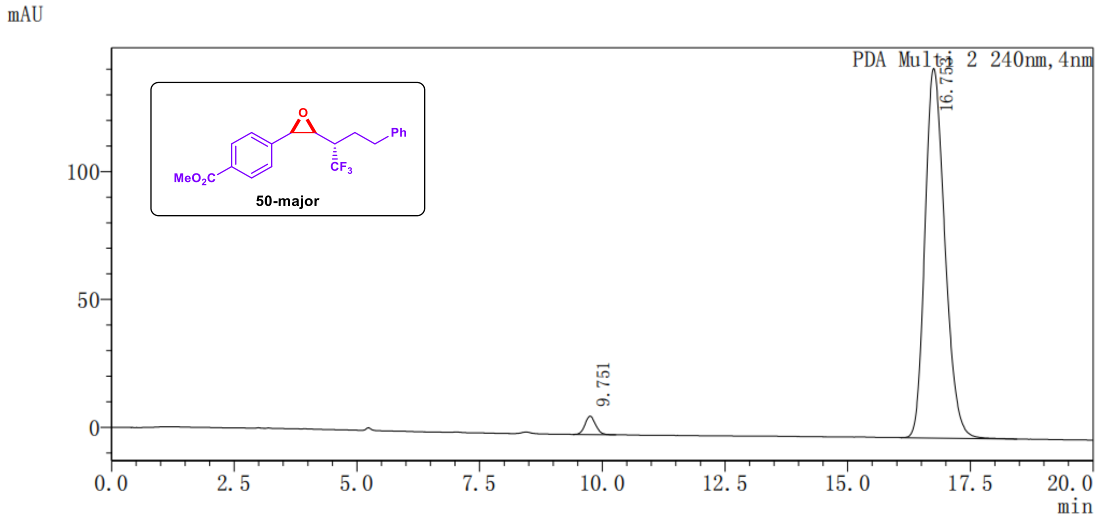

Peak#	Ret. Time	Area	Area#
1	9.751	107852	2.580
2	16.753	4072963	97.420
Total		4180815	100.000

Supplementary Figure 232. Chiral HPLC analysis of Compound **50-major**

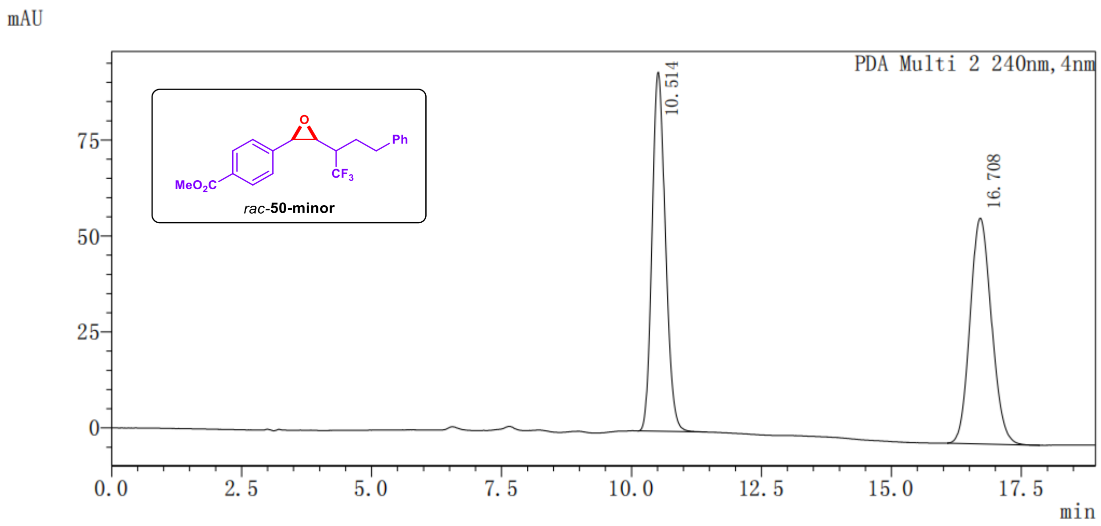

Peak#	Ret. Time	Area	Area#
1	10.514	1674654	49.800
2	16.708	1688120	50.200
Total		3362774	100.000

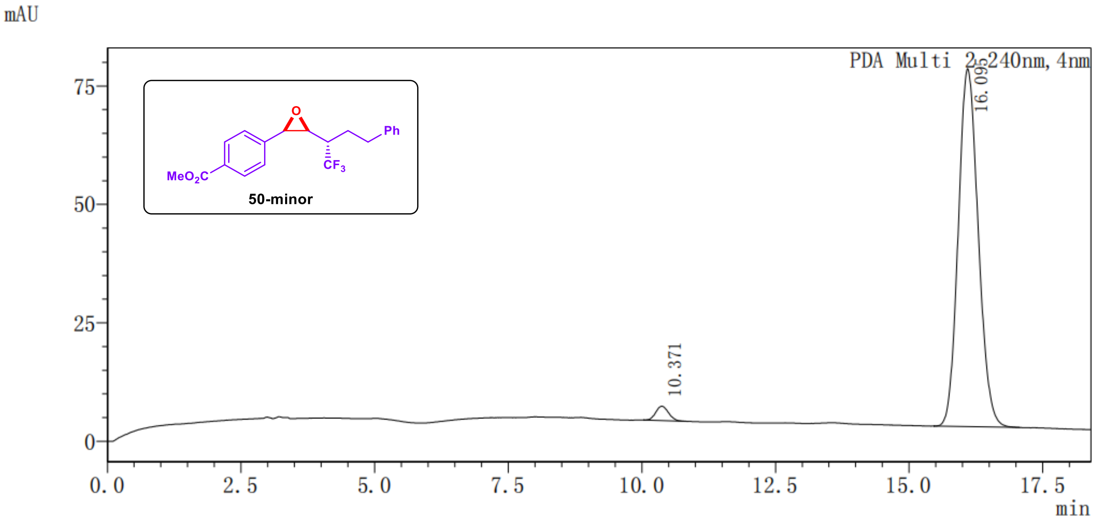

Peak#	Ret. Time	Area	Area#
1	10.371	50383	2.498
2	16.095	1966613	97.502
Total		2016996	100.000

Supplementary Figure 233. Chiral HPLC analysis of Compound **50-minor**

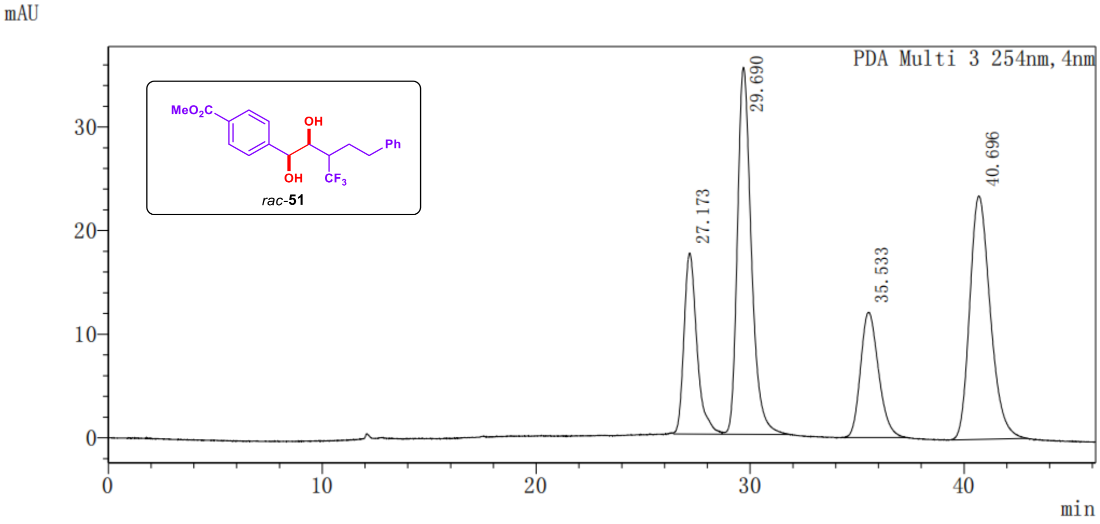

Peak#	Ret. Time	Area	Area#
1	27.173	737981	15.973
2	29.690	1600698	34.646
3	35.533	711088	15.391
4	40.696	1570332	33.989
Total		4620099	100.000

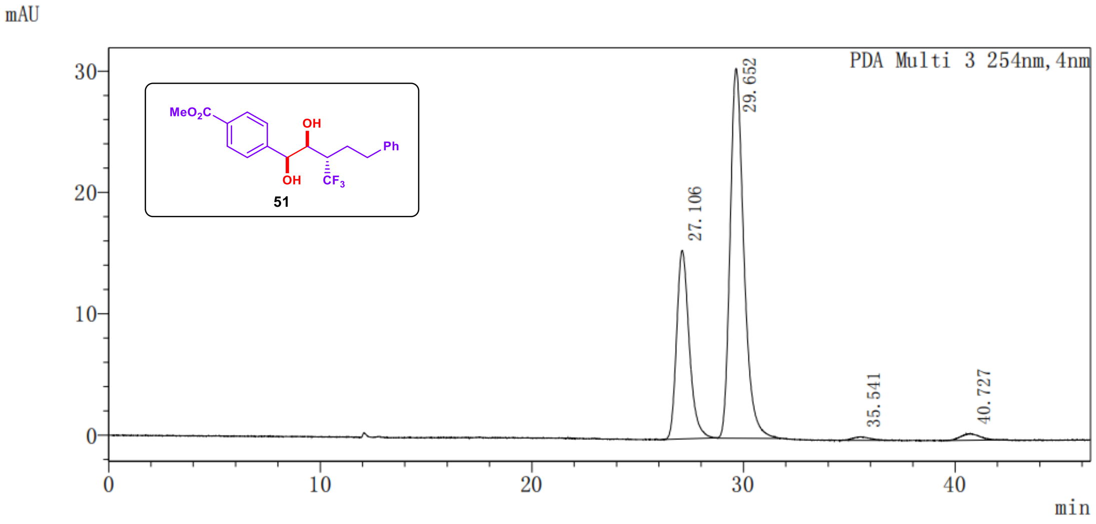

Peak#	Ret. Time	Area	Area#
1	27.106	642109	31.058
2	29.652	1373615	66.439
3	35.541	16601	0.803
4	40.727	35157	1.700
Total		2067481	100.000

Supplementary Figure 234. Chiral HPLC analysis of Compound **51**

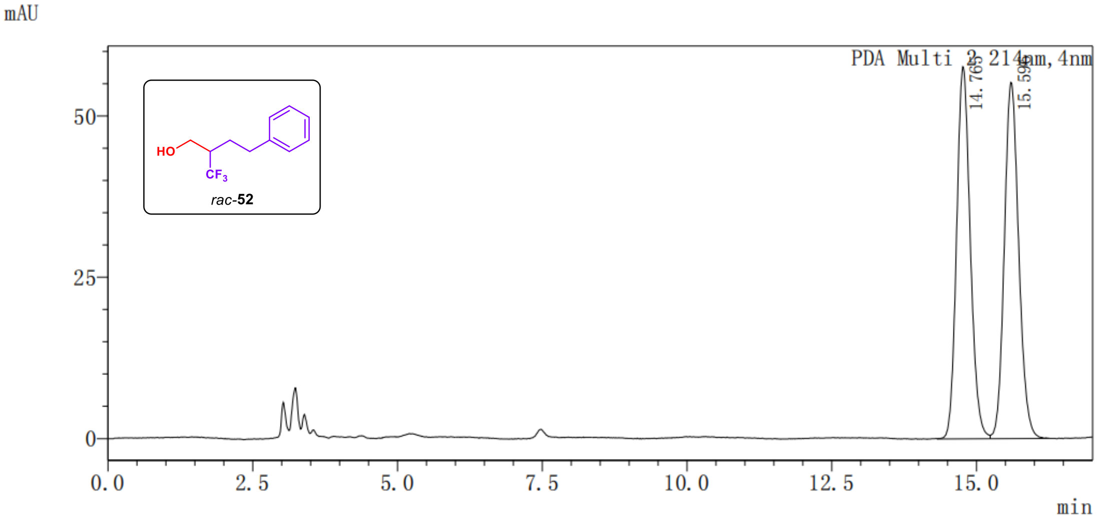

Peak#	Ret. Time	Area	Area#
1	14.765	947817	49.833
2	15.596	954165	50.167
Total		1901983	100.000

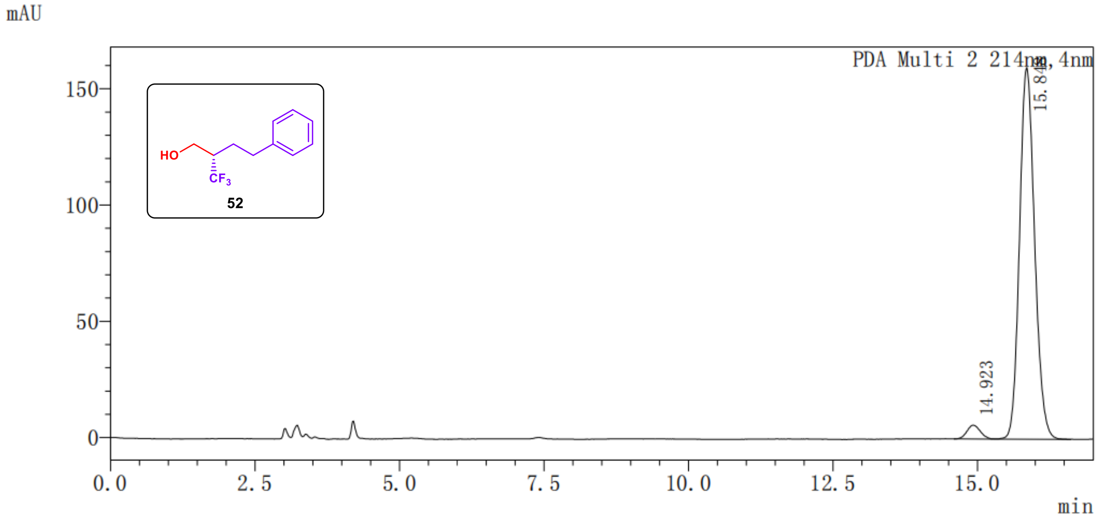

Peak#	Ret. Time	Area	Area#
1	14.923	95405	3.181
2	15.848	2904149	96.819
Total		2999555	100.000

Supplementary Figure 235. Chiral HPLC analysis of Compound **52**

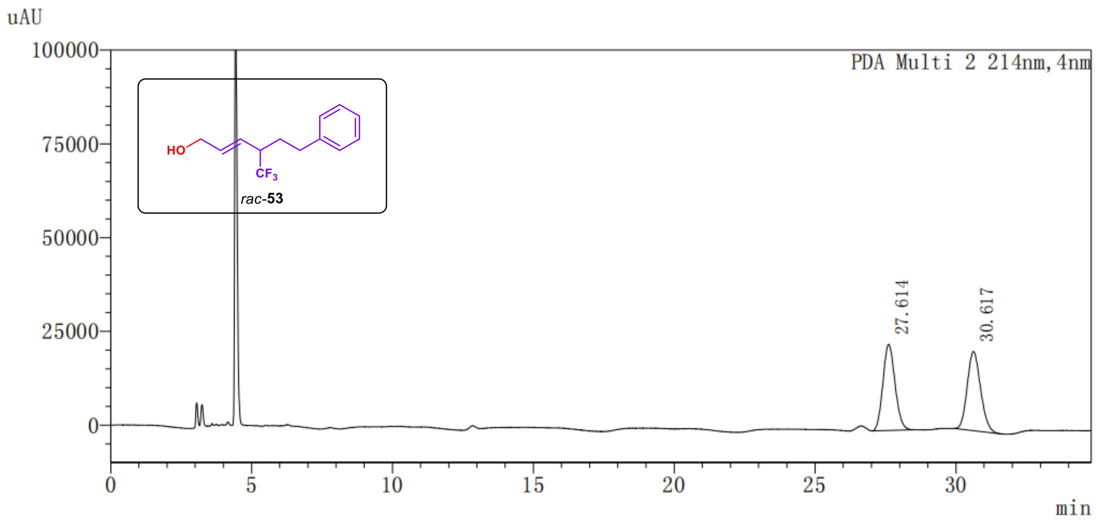

Peak#	Ret. Time	Area	Area#
1	27.614	686302	49.555
2	30.617	698632	50.445
Total		1384934	100.000

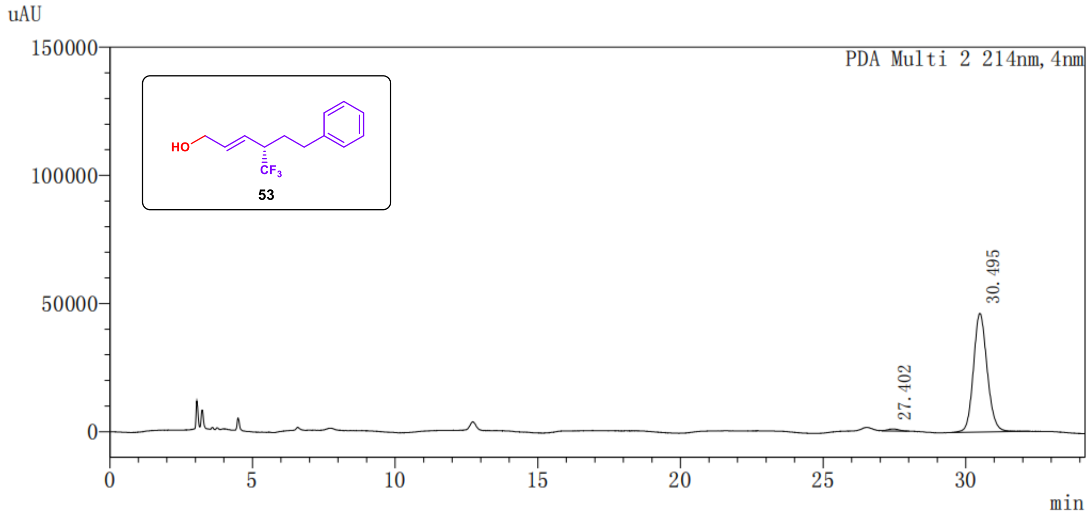

Peak#	Ret. Time	Area	Area#
1	27.402	23095	1.463
2	30.495	1556023	98.537
Total		1579119	100.000

Supplementary Figure 236. Chiral HPLC analysis of Compound **53**

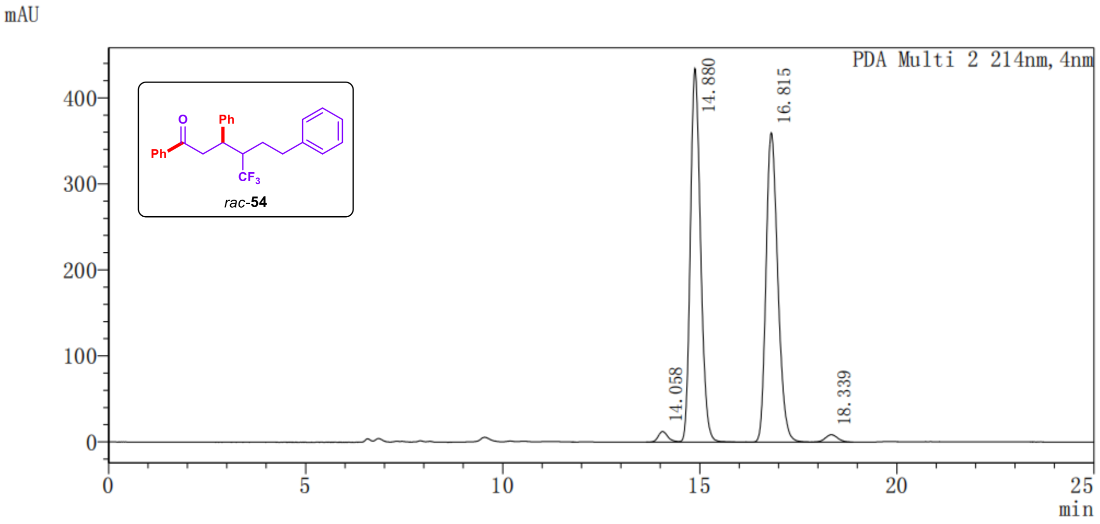

Peak#	Ret. Time	Area	Area#
1	14.058	211373	1.347
2	14.880	7804678	49.721
3	16.815	7488852	47.709
4	18.339	191945	1.223
Total		15696848	100.000

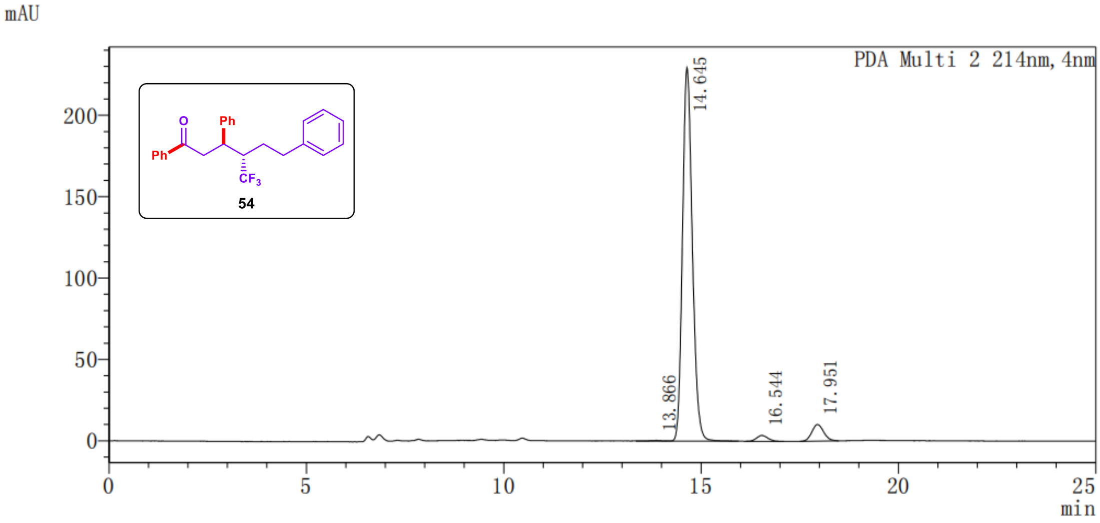

Peak#	Ret. Time	Area	Area#
1	13.866	9438	0.221
2	14.645	3976842	93.039
3	16.544	74134	1.734
4	17.951	213948	5.005
Total		4274362	100.000

Supplementary Figure 237. Chiral HPLC analysis of Compound **54**

IV. Supplementary References

(1) Ding, W., Lu, L.-Q., Zhou, Q.-Q., Wei, Y., Chen, J.-R., Xiao, W.-J. Bifunctional Photocatalysts for Enantioselective Aerobic Oxidation of β-Ketoesters. *J. Am. Chem. Soc*. **139**, 63-66 (2017).

(2) Shrestha, B., Rose, B. T., Olen, C. L., Roth, A., Kwong, A. C., Wang, Y., Denmark. S. B. E. A Unified Strategy for the Asymmetric Synthesis of Highly Substituted 1,2-Amino Alcohols Leading to Highly Substituted Bisoxazoline Ligands. *J. Org. Chem.* **86**, 3490-3534 **(**2021).

(3) Wu, B.-B., Xu, J., Bian, K.-J., Gao, Q., Wang, X.-S. Enantioselective Synthesis of Secondary β-Trifluoromethyl Alcohols via Catalytic Asymmetric Reductive Trifluoroalkylation and Diastereoselective Reduction. *J. Am. Chem. Soc*. **144**, 6543-6550 (2022).

(4) (a) Cox, L. R., DeBoos, G. A., Fullbrook, J. J., Percy, J. M., Spencer, N. S., Tolley, M. Catalytic asymmetric synthesis of a 1-deoxy-1,1-difluoro-D-xylulose. *Org. Lett*. **5**, 337-339 (2003). (b) Dai, Y.-B., Wang, F., Zhu, S.-Q., Chu, L.-L. Selective Ni-catalyzed cross-electrophile coupling of alkynes, fluoroalkyl halides, and vinyl halides. *Chinese Chemical Letters*. **12**, 050 (2021).

(5) Huang, Z.-H., Negishi, E. A Convenient and Genuine Equivalent to $HZrCp_2Cl$ Generated in Situ from $ZrCp_2Cl_2$−DIBAL-H. *Org. Lett*. **8**, 3675-3678 (2006).

(6) Abe, M., Yokoi, Y., Hirata, A., Matsuoka, Y., Kimachi, T., Inamoto, K. Site-selective iodine atom transfer in fluorinated alkyl iodides via 1,5-hydrogen atom transfer. *Chem. Commun*. **58**, 7416 (2022).
